# Identifying Quantum Tunneling at Ambient Temperature through Rate–Driving Force Responsiveness

**DOI:** 10.1021/jacs.6c08355

**Published:** 2026-08-07

**Authors:** Flóra Barasits, Guanqi Qiu

**Affiliations:** Max-Planck-Institut für Kohlenforschung, Kaiser-Wilhelm-Platz 1, 45470, Mülheim an der Ruhr, Germany

## Abstract

Quantum mechanical tunneling can strongly influence chemical reactivity, yet its experimental identification remains limited. Here we derive a new diagnostic principle: tunneling leaves a distinct signature in the rate-driving force relationship. We show that tunneling reshapes the scaling relationship between reaction rate and driving force. Within a Hammond-type interpolation of barrier topology, over-the-barrier reactivity primarily translates the interpolated change in barrier height into rate–driving-force responsiveness. When through-barrier transmission contributes, the interpolated change in barrier width is also expressed in the rate response, producing significantly enhanced responsiveness. This principle enables a simple experimentally anchored diagnostic for tunneling based on responsiveness analysis within a reaction family, complementing existing diagnostics that often rely on cryogenic temperatures or isotopic substitution. We demonstrate the principle using the *syn* elimination of selenoxides as a benchmark model system at room temperature. Because this diagnostic principle relies only on standard kinetic measurements, it should be broadly applicable to reactions with measurable elementary kinetics.

## Graphical abstract

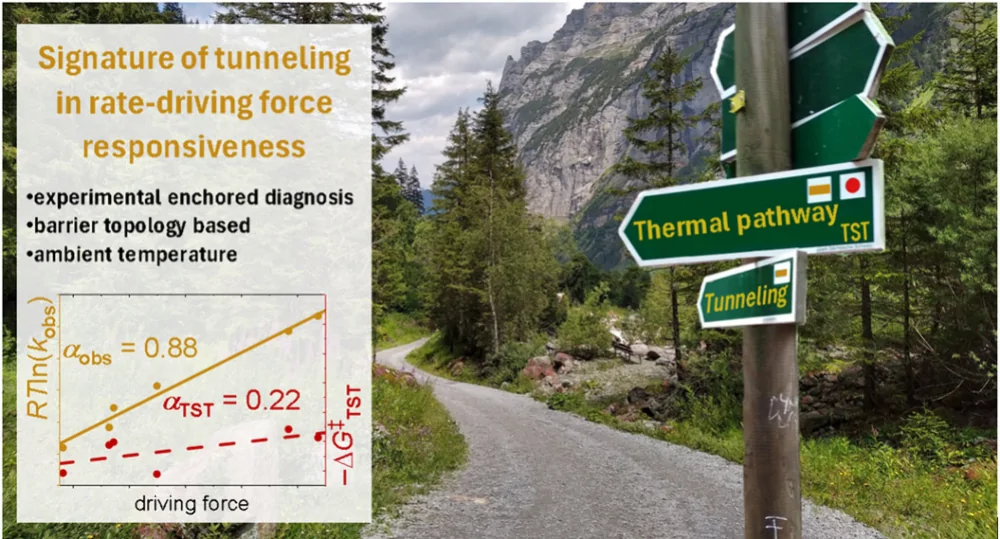

Quantum mechanical tunneling (QMT) allows particles to traverse energy barriers that would be insurmountable under classical mechanics. QMT is present in a broad range of chemical reactions^1–8^ and it opens a new reaction channel that can alter selectivity and enhance reactivity.^9–12^ However, identification of tunneling contributions remains challenging because established experimental diagnostics have limited applicability (Figure 1a). Temperature-based diagnostics probe the role of thermal activation in crossing the reaction barrier, as tunneling can lead to unusually weak temperature dependence of reaction rates.^11,13^ However, because tunneling and over-the-barrier reactivity often coexist under typical reaction conditions, such diagnostics can be difficult to interpret.^13–19^ Practically, temperature-based diagnostics often require cryogenic conditions^11,20–23^ and may not reflect reactivity under the conditions of interest.^6,13,24–27^ Another common diagnostic is based on unusually large KIEs, which probes the dependence of tunneling on nuclear mass.^7,19,28–34^ However, for heavy atoms, isotopic substitution often produces only modest changes in rate.^5,21–23,35–38^ Moreover, a large KIE can be a result of phenomena other than tunneling, such as H-bonding or cumulative effects from multiple steps.^39–44^ Both diagnostics probe tunneling by perturbing a single reaction, and useful signals often require large perturbations that narrow the experimentally relevant window.

**Figure 1:**
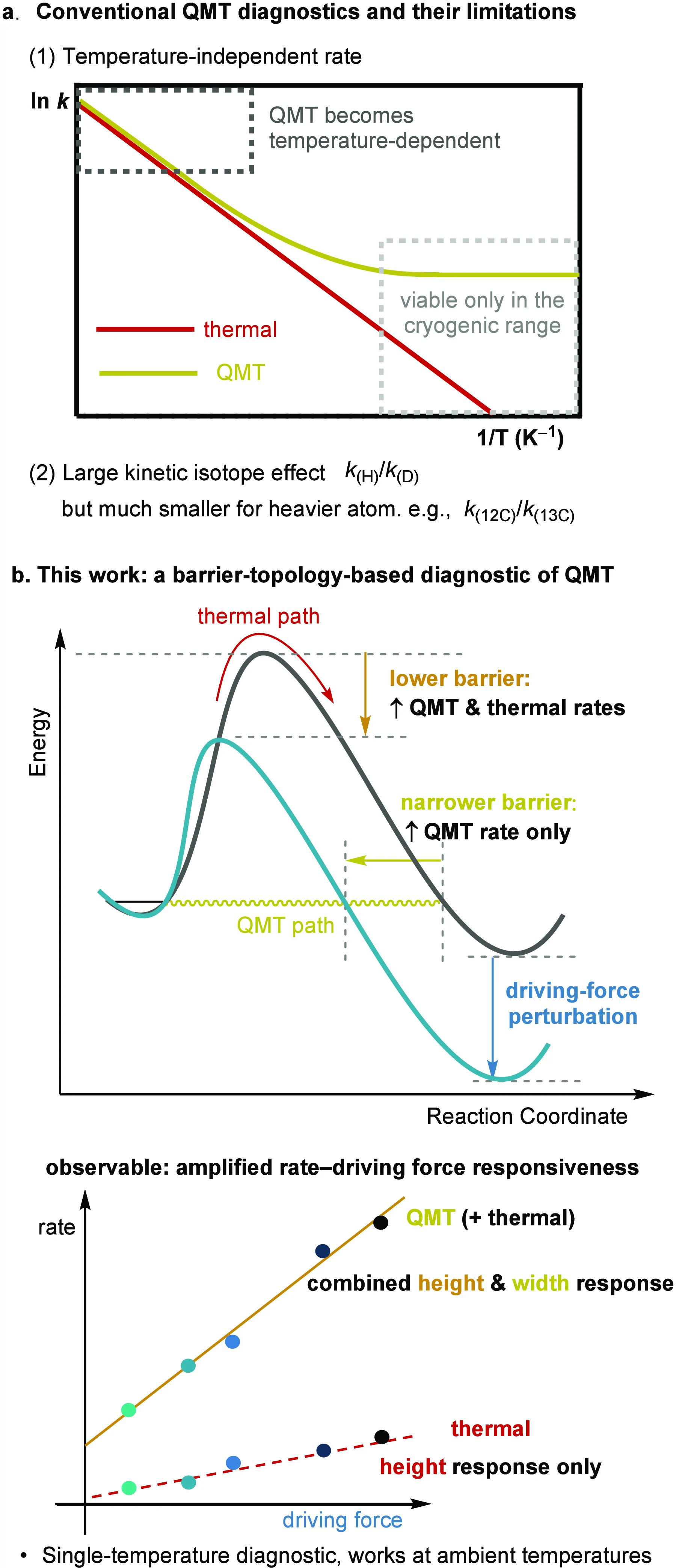
**Diagnostic principles of quantum mechanical tunneling through kinetic–thermodynamic responsiveness.** (a) Common experimental signatures of tunneling and their limitations. (b) This work: Diagnostic principle based on topology of the reaction barrier (top), leading to experimentally anchored detection of tunneling through rate–driving force responsiveness under ambient conditions (bottom): qualitative comparison of the thermal and observed (QMT + thermal) responsiveness, which diverge with increasing tunneling contribution.

These limitations motivate the search for alternative experimental signatures of tunneling. Here we introduce a diagnostic principle, considering a different physical basis: the topology of the reaction barrier, encompassing both barrier height and barrier width (Figure 1b). Whereas classical transition state theory links reaction rates primarily to barrier height (red path), tunneling depends on both components (yellow path). This leads to a diagnostic principle based on whether both aspects of barrier topology are reflected in the responsiveness of reaction rates to thermodynamic driving force, providing a qualitative experimental diagnostic of significant tunneling contributions. Consequently, significant tunneling contributions are expected to produce an enhanced rate–driving force responsiveness relative to the classical over-the-barrier pathway. This responsiveness can be obtained from standard kinetic data across a closely related reaction family, providing an experimentally anchored diagnostic for significant tunneling contributions under the reaction conditions of interest.

To connect this diagnostic to the physical-organic framework of rate–driving-force relationships, we first recall how thermodynamic driving force (−Δ*G*) interpolates the activation barrier within a reaction family. In a Hammond–Leffler description, changes in reaction free energy shift the transition state and change the activation barrier height. Consequently, the responsiveness of reaction rates to thermodynamic driving force is determined by how the barrier height changes with reaction free energy Δ*G*. This coupling arises because changes in reaction thermodynamics generally shift the position and energy of the transition state, consistent with the Hammond postulate:^45^ more exergonic driving forces shift the TS toward the reactant side (Figure 2a). This relationship is commonly expressed through the Brønsted slope α, which reflects how strongly the barrier height responds to thermodynamic perturbations. Within the Leffler approximation,^46^ the activation free energy can be written as Δ*G* ^‡^ = Δ*G*_0_^‡^ + αΔ*G*, where Δ*G*_0_^‡^ is the intrinsic barrier corresponding to a thermoneutral reaction (Δ*G* = 0). The parameter α therefore represents this relationship and is considered an intrinsic property of a reaction family (the black, dark blue, and light blue potentials in Figure 2a represent a family of reactions related by systematic thermodynamic perturbation, as described by the equation above). Under classical transition state theory, this responsiveness is bounded between 0 and 1,^47^ as expected from the Hammond postulate. In the over-the-barrier limit, this barrier-height interpolation is translated into rate responsiveness through the Eyring relationship, where rates depend exponentially on Δ*G* ^‡^.^48^ Thus, experimentally, responsiveness can be evaluated through a Brønsted plot (Figure 2b), in which activation energies (Δ*G* ^‡^) are correlated with driving forces (−Δ*G*) for a family of reactions.

**Figure 2:**
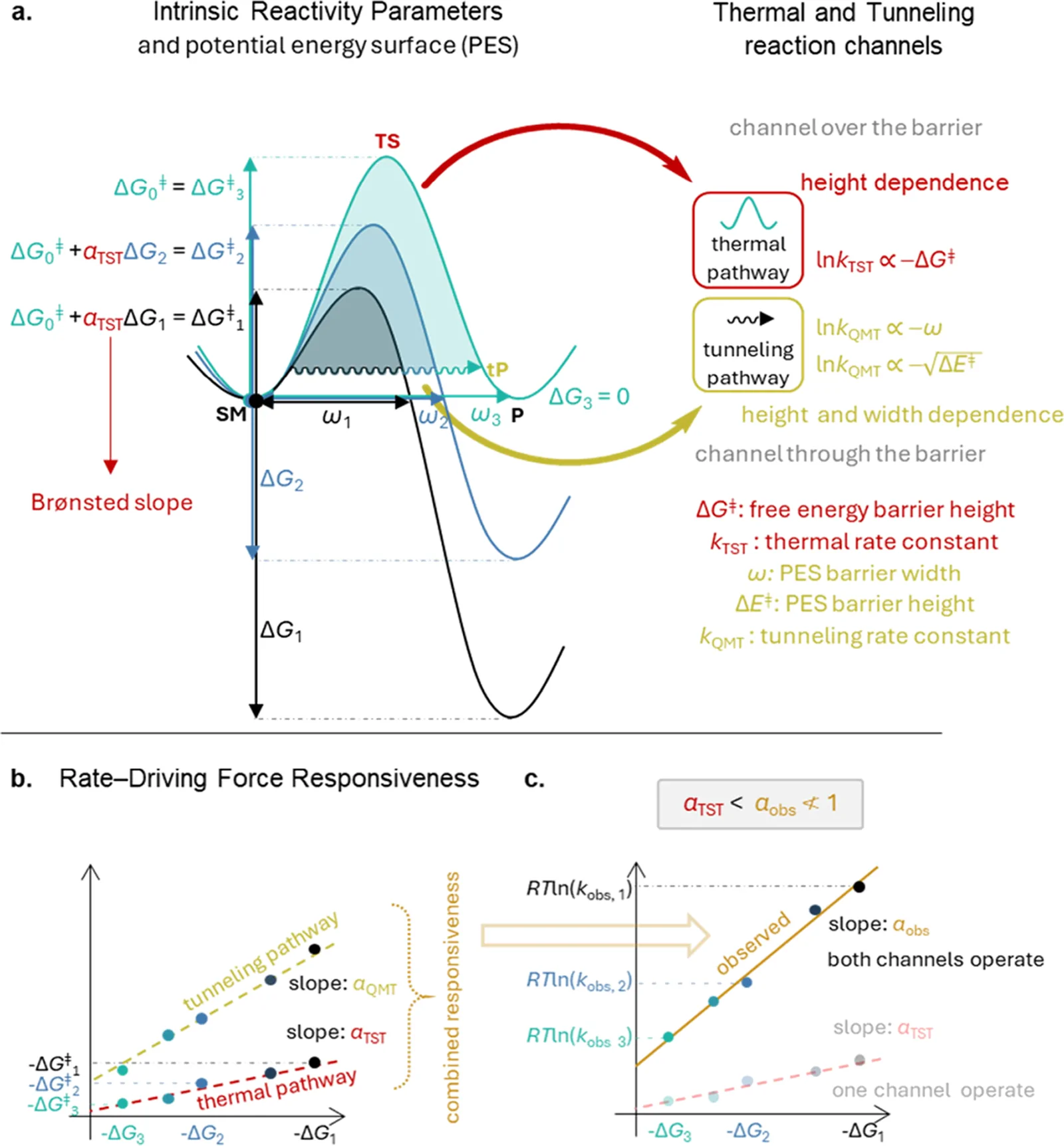
**Relation of QMT to intrinsic reactivity.** (a) Barrier-topology origin of thermal (red) and tunneling (yellow) pathways for a family of reactions (blue scale). SM: starting material ground state; TS: transition state; tP: tunneling product; P: product ground state. (b) Conceptual origin of enhanced responsiveness in case of QMT (yellow) and construction of thermal Brønsted plot (red). (c) Experimental readout of tunneling-enhanced responsiveness by construction of observed Brønsted plot (orange) and comparison with thermal Brønsted plot (red).

Besides height, barrier width (ω) is another crucial component of barrier topology that influences tunneling probabilities even more strongly.^49,50^ In semiclassical descriptions of tunneling, the transmission probability depends exponentially on barrier width, such that even small changes in ω can produce substantial variations in tunneling rates.^51–55^ The tunneling rate depends on both barrier height and barrier width, both of which respond systematically to thermodynamic driving-force perturbation through Bell–Evans–Polanyi/Hammond-type interpolation of the underlying barrier topology.^56,57^ Product stabilization makes the barrier both lower and narrower.^58^ As illustrated in Figure 2a, classical thermal reactivity responds to driving force primarily through changes in barrier height (red channel over the barrier), whereas tunneling introduces an additional contribution arising from barrier-width sensitivity (yellow channel through the barrier). This dual dependence makes the tunneling rate more responsive to thermodynamic perturbations (Figure 2b yellow dashed slope) than the classical over-the-barrier pathway (red dashed slope). With significant tunneling, the combined rate–driving-force responsiveness contains the classical thermal response to barrier height plus additional tunneling responses to both barrier height and barrier width, yielding an enhanced overall slope (Figure 2b, **c**). The additional rate–driving force responsiveness arises when the Bell–Evans–Polanyi-type barrier-width response of the underlying potential energy surface (PES) topology is translated into observable rate responsiveness through significant through-the-barrier reactivity. Because tunneling is defined on the PES, whereas rate–driving force relationships are expressed on an apparent free-energy scale, a comparison shows that the computed potential-energy and free-energy descriptors vary consistently across the substituent series (), in line with the distinction highlighted previously.^59^ Thus, we interpret the observed enhanced responsiveness as an experimental manifestation of PES-level barrier-topology changes. Importantly, the analysis does not assume a particular analytical barrier shape. The practical diagnostic is based on whether the experimentally observed rate–driving force responsiveness exceeds the classical over-the-barrier response expected for the same reaction family.

Other nonclassical effects such as vibrational quantization, dynamic transmission, and mode coupling can influence absolute rates, but can explain enhanced responsiveness only if they vary coherently, with the required sign and sufficient magnitude. This distinction is summarized schematically in Figure 3. Within this framework, tunneling is distinguished because transmission through the same Hammond-evolving barrier provides a direct physical mechanism by which thermodynamic barrier interpolation is transduced into enhanced rate responsiveness. In contrast, the lower-left contributions become viable explanations only if the remote driving-force perturbation also modulates those effects coherently, with the correct sign and sufficient magnitude, which is highly demanding. Representative readouts show no coherent driving-force dependence or only minor variation ().

**Figure 3:**
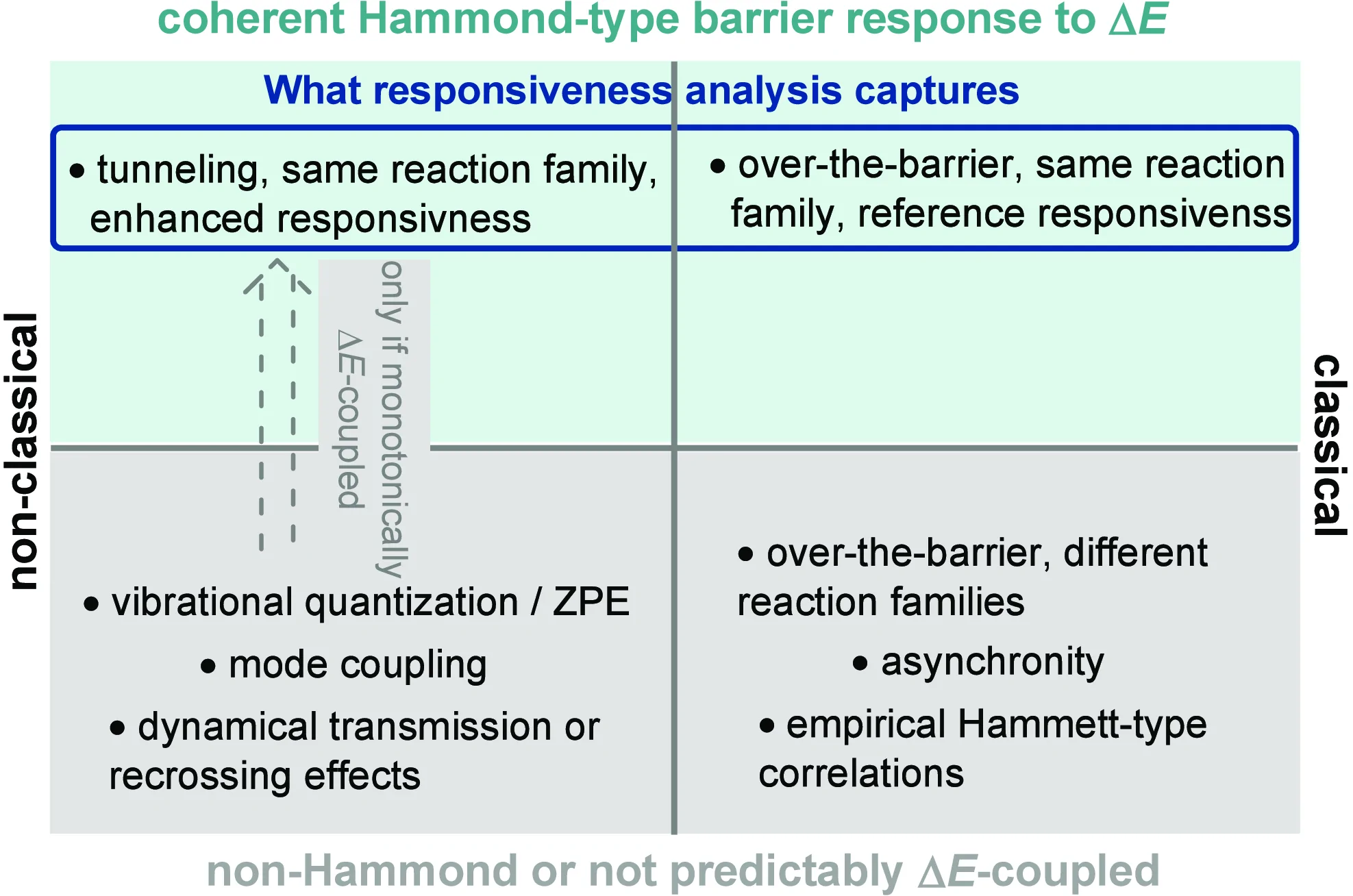
Scope of the responsiveness analysis. Rate–driving-force responsiveness analysis captures contributions that are coherently coupled to a Hammond-type barrier response within a preserved reaction family. Tunneling and over-the-barrier reactivity are compared within a preserved Hammond-type reaction family; other effects contribute only if they are coherently coupled to the driving-force perturbation with sufficient magnitude.

With the two components combined, the classical upper bound of Brønsted slopes no longer applies. As a practical convenience, observation of α_obs_ > 1 immediately indicates that the classical bound has been exceeded, and explicit calculation of the classical slope α_TST_ is no longer necessary. However, this shortcut requires some caution. The classical 0–1 bound applies only when the transition state directly connects the corresponding reactant and product minima along the reaction coordinate. In asynchronous processes, apparent violations can arise if the analyzed transition state is assigned to an incorrect reactant–product pair on the underlying potential energy surface.^60^ A simple intrinsic reaction coordinate (IRC)^61,62^ calculation can verify this connectivity and exclude apparent violations arising from asynchronicity.

Experimentally, variations in thermodynamic driving force can be achieved by introducing substituents with different electronic properties at positions remote from the reaction center. This design allows the modulation of the reaction driving force while only minimally affecting the intrinsic reactivity. Plotting *RT*ln­(*k*_obs_) against the thermodynamic driving force (determined computationally here, but in principle accessible experimentally) for a family of reactions therefore provides a direct experimental measure of α_obs_ by linear fitting (Figure 2c orange line). The classical slope α_TST_ (Figure 2b red line) can be computed from straightforward transition state calculations of the barrier heights of the same family of reactions.

To establish the derived diagnostic experimentally, we applied it to selenoxide *syn* elimination, a mechanistically simple benchmark with well-established tunneling contributions.^63^ This proof-of-concept system allows the predicted responsiveness signature to be evaluated against an independently established tunneling case. Selenides 1a–f were synthesized through diphenyl diselenide intermediates.^64,65^ Driving force was varied by introducing *para* substituents (R_a‑f_ = OMe, Me, H, Cl, CF_3_, CN) on the selenium-bound phenyl ring, while largely preserving the intrinsic reactivity. Broad thermodynamic ranges enable more accurate determination of the responsiveness. For kinetic rate measurements selenoxide **2** was prepared *in situ* from the respective selenide precursors **1**. The formation of **3** was then monitored via ^1^H NMR from the time point when a selenide was fully consumed to track selenoxide elimination at room temperature (Figure 4a). Values of the driving force were calculated by subtracting the free energy of the tunneling product (tP) from the ground state of respective starting materials (SM) computationally. The tunneling product is characterized by the fully formed OH bond of the product selenic acid **4** on the IRC (see Figure 2a and ). The barrier height Δ*G* ^‡^_TST_ was obtained from transition state calculations for the pathway connecting SM and tP.

**Figure 4:**
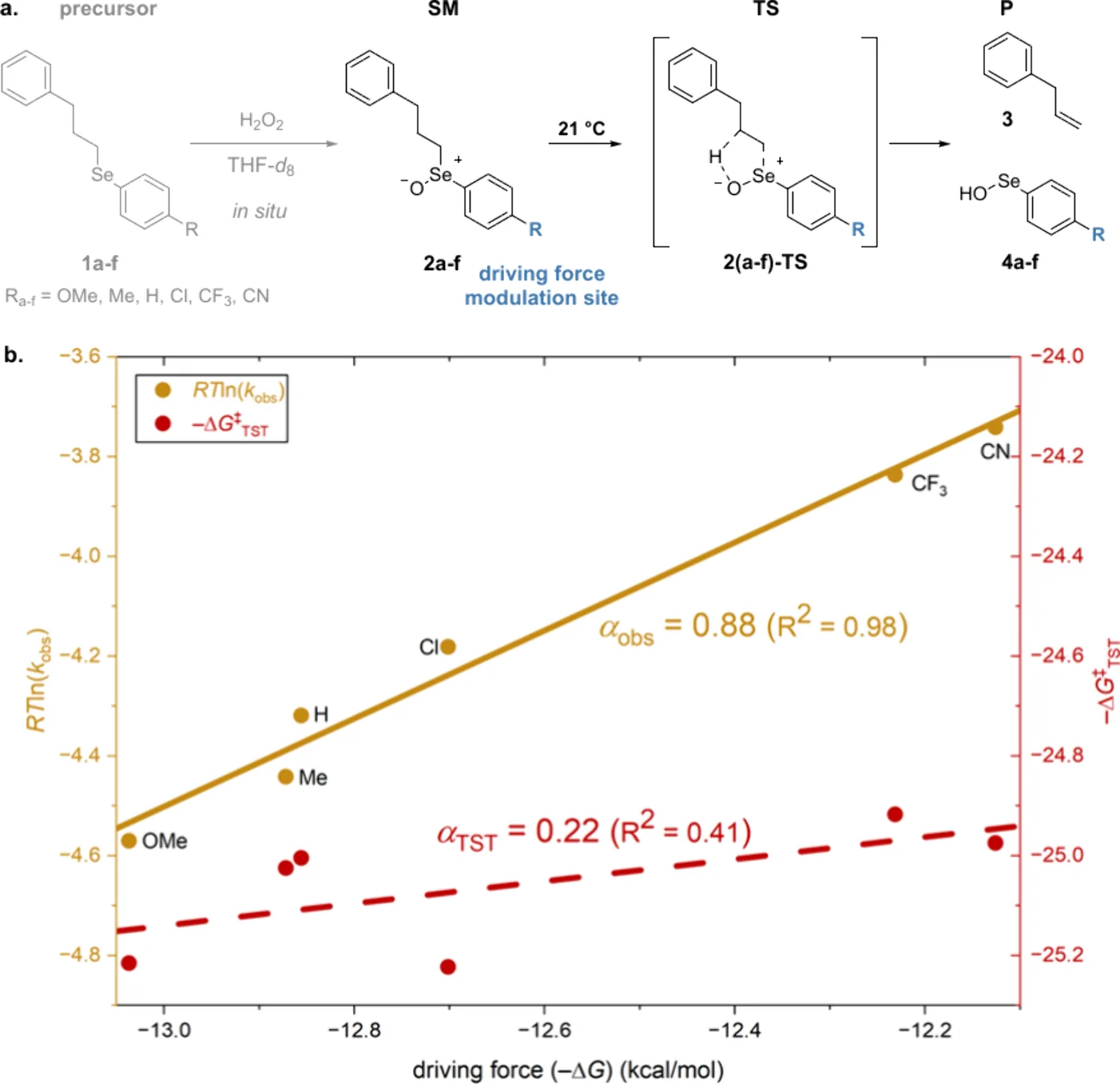
**Experimental demonstration of the new diagnostic.** (a) Model system: *syn* elimination of selenoxides. (b) Comparison graph of observed (α_obs_) and thermal (α_TST_) Brønsted slopes for selenoxide elimination. Observed rate constants are averages of three measurements (−Δ*G* ^‡^_obs_ = *RT*ln­(*k*_obs_)), while the classical barrier height (Δ*G* ^‡^_TST_) and driving force (−Δ*G* = *E*_tP_ – *E*_SM_) are determined computationally for each data point via IRC calculation using B3LYP-D3­(BJ)/def2-TZVP level of theory with universal solvation model (SMD) in THF^66–68^ via ORCA 6.1.^69–74^

The Brønsted plot of selenoxide elimination is shown in Figure 4b, comparing the experimentally observed responsiveness (α_obs_) with the classical thermal prediction (α_TST_). The observed Brønsted slope is close to unity (α_obs_ = 0.88) with good linear correlation (R^2^ = 0.98). In contrast, the computed classical slope, obtained from transition state calculations of the barrier height across the same reaction series, is four times smaller (α_tst_ = 0.22). The small α_TST_ produces a narrow dynamic range and therefore greater scatter (R^2^ = 0.41). α_obs_ remains consistently and significantly higher than α_TST_ across all tested levels of theory (). In case the experimental α_obs_ were only slightly higher than the α_TST_, further assessment would be needed. According to our conceptually derived diagnostic principle, the large difference between α_obs_ and α_TST_ indicates a significant tunneling contribution to the mechanism of selenoxide elimination in alignment with previous knowledge, validating the viability of our new diagnostic for quantum mechanical tunneling. Together, these results establish responsiveness analysis as an experimentally anchored diagnostic for tunneling under ambient conditions.

In conclusion, we establish a new diagnostic principle for quantum tunneling, based on the responsiveness of reaction rates to thermodynamic driving force. By introducing an additional dependence of the rate on barrier width, reactions with significant tunneling contributions exhibit Brønsted slopes that exceed the classical transition state theory prediction. Based on this principle, we established responsiveness analysis as a practical diagnostic for tunneling that operates under any single temperature of choice, relying only on standard kinetic measurements. Because responsiveness analysis is grounded in standard intrinsic reactivity relationships, it is expected to be a broadly relevant diagnostic principle for chemical reactions with measurable elementary kinetics, enabling the identification of previously unrecognized tunneling contributions.

## Supplementary Material





## Notes

IRC output for all substrates (PDF)
General information, synthetic procedures, responsiveness analysis, computational methods, NMR spectra, computational coordinates, references (PDF)
