# Supplementary material for: Identifying Quantum Tunneling at Ambient Temperature through Rate–Driving Force Responsiveness
Source: J Am Chem Soc. 2026 Aug 7;148(32):34038–44. doi: 10.1021/jacs.6c08355 (PMC13495758; doi:10.1021/jacs.6c08355)
Supplement: Supplementary file 1 [file ja6c08355_si_001.pdf]

Identifying Quantum Tunneling at Ambient Temperature through Rate–Driving Force Responsiveness

Flóra Barasits[1], Guanqi Qiu[1]*

[1]*Max-Planck-Institut für Kohlenforschung, Kaiser-Wilhelm-Platz 1, 45470, Mülheim an der Ruhr, Germany*

*Corresponding author. Email: gqiu@kofo.mpg.de

Full IRC output for all substrates

Table of Contents

IRC output for R = OMe 2
IRC output for R = Me 62
IRC output for R = H 121
IRC output for R = Cl 179
IRC output for R = CF_3 237
IRC output for R = CN 298

IRC output for R = OMe

```
! Job execution by suborca V 10.04 !
Job started from head2.mpi-muelheim.mpg.de, running
/hsnfs/software/orca/orca_current_directory/orca_current_20251124/orca on xe30th86.mpi-
muelheim.mpg.de in /usr/scratch/barasits/LQA_TS-OMe__26790259 (/bin/bash)
Job execution start: Mon Nov 24 10:56:53 AM CET 2025
Shared library path: /hsnfs/software/openmpi-
4.1.0/lib:/hsnfs/software/orca/orca_current_directory/orca_current_20251124:/hsnfs/software/the
mis/llvm-14.0.1/lib:/hsnfs/software/orca/orca_stable:/usr/users/openmpi-
2.0.4/lib:/opt/intel/lib/intel64:/opt/intel/mkl/lib/intel64
SLURM Job ID is: 26790259
SLURM_JOB_NODELIST: xe30th86
Maxcore enforced by SLURM: 9600 MB per core
Expanded nodelist (1 nodes):
xe30th86

                                 *****************
                                 * O   R   C   A *
                                 *****************

                                            #,
                                            ###
                                            ####
                                            #####
                                            ######
                                           ########,
                                     ,,################,,,,,
                               ,,#################################,,
                          ,,##########################################,,
                       ,#########################################, ''#####,
                    ,#############################################,,   '####,
                  ,##################################################,,,,####,
                ,###########''''           ''''###############################
              ,#####''   ,,,,##########,,,,          '''####'''          '####
            ,##' ,,,,###########################,,,                        '##
           ' ,,###''''                  '''############,,,
         ,,##''                                '''############,,,,        ,,,,,,###''
      ,#''                                            '''#######################'''
     '                                                          ''''####''''
             ,#######,   #######,   ,#######,      ##         ,####,    ,#,
            ,#'     '#,  ##    ##  ,#'     '#,    #''#        ,####,    ,#,
            ##       ##  ##   ,#'  ##            #'  '#       #'       ,# #
            ##       ##  #######   ##           ,######,      #####,      #
            '#,     ,#'  ##    ##  '#,     ,#' ,#      #,     #,   #      #
             '#######'   ##     ##  '#######'  #'      '#     '####' #    #


                ##########################################################
                #                        -***-                           #
                #          Department of theory and spectroscopy         #
                #                                                        #
                #                      Frank Neese                       #
                #                                                        #
                #     Directorship, Architecture, Infrastructure         #
                #                    SHARK, DRIVERS                      #
                #        Core code/Algorithms in most modules            #
                #                                                        #
                #        Max Planck Institute fuer Kohlenforschung       #
                #                Kaiser Wilhelm Platz 1                  #
                #                 D-45470 Muelheim/Ruhr                  #
                #                      Germany                           #
                #                                                        #
                #                  All rights reserved                   #
```

```
                #                        -***-                        #
                #######################################################


                         Program Version 6.1.x  -  CURRENT   -
                                (GIT: $ce43e543$)
                ($2025-11-21 17:32:59 +0100 $)

                  !                   WARNING:                          !
                  !         This is a DEVELOPMENT version               !

                         (BUILD: $Mon 24 Nov 2025 12:05:30 AM CET$)


 With contributions from (in alphabetic order):
[Max-Planck-Institut fuer Kohlenforschung]
  Daniel Aravena         : Magnetic Suceptibility
  Michael Atanasov       : Ab Initio Ligand Field Theory (pilot matlab implementation)
  Alexander A. Auer      : GIAO ZORA, VPT2 properties, NMR spectrum
  Ute Becker             : All parallelization in ORCA, NUMFREQ, NUMCALC
  Giovanni Bistoni       : ED, misc. LED, open-shell LED, HFLD
  Dmytro Bykov           : pre 5.0 version of the SCF Hessian
  Marcos Casanova-Páez   : Triplet and SCS-CIS(D). UHF-(DLPNO)-IP/EA/STEOM-CCSD. UHF-CVS-
IP/STEOM-CCSD
  Vijay G. Chilkuri      : MRCI spin determinant printing, contributions to CSF-ICE
  Pauline Colinet        : FMM embedding
  Dipayan Datta          : RHF DLPNO-CCSD density
  Achintya Kumar Dutta   : EOM-CC, STEOM-CC
  Nicolas Foglia         : Exact transition moments, OPA infrastructure, MCD improvements
  Dmitry Ganyushin       : Spin-Orbit,Spin-Spin,Magnetic field MRCI
  Miquel Garcia-Rates    : C-PCM and meta-GGA Hessian, CCSD/C-PCM, Gaussian charge scheme
  Tiago L. C. Gouveia    : GS-ROHF, GS-ROCIS
  Yang Guo               : DLPNO-NEVPT2, F12-NEVPT2, CIM, IAO-localization
  Andreas Hansen         : Spin unrestricted coupled pair/coupled cluster methods
  Ingolf Harden          : AUTO-CI MPn and infrastructure
  Benjamin Helmich-Paris : MC-RPA, TRAH-(SCF,CASSCF), AVAS, COSX integrals, SCF dyn. polar.,
MC-PDFT, srDFT
  Lee Huntington         : MR-EOM, pCC
  Robert Izsak           : Overlap fitted RIJCOSX, COSX-SCS-MP3, EOM
  Riya Kayal             : Wick's Theorem for AUTO-CI, AUTO-CI UHF-CCSDT
  Emily Kempfer          : AUTO-CI RHF CISDT and CCSDT, approximate NEVPT4
  Christian Kollmar      : KDIIS, OOCD, Brueckner-CCSD(T), CCSD density, CASPT2, CASPT2-K,
improved NEVPT2
  Axel Koslowski         : Symmetry handling
  Simone Kossmann        : meta-GGA functionals, TD-DFT gradient, OOMP2, (MP2 Hessian;
deprecated post 5.0)
  Lucas Lang             : DCDCAS, Hyperfine gauge corrections, ICE-SOC+SSC
  Marvin Lechner         : AUTO-CI (C++ implementation), FIC-MRCC
  Spencer Leger          : CASSCF response
  Dagmar Lenk            : GEPOL surface, SMD, ORCA-2-JSON
  Dimitrios Liakos       : Extrapolation schemes; Compound Job, Property file
  Dimitrios Manganas     : Further ROCIS development; embedding schemes. LFT, Crystal Embedding
  Hagen Neugebauer       : wr2SCAN, Native XTB
  Dimitrios Pantazis     : SARC Basis sets
  Anastasios Papadopoulos: AUTO-CI, single reference methods and gradients
  Taras Petrenko         : pre 6.0 DFT Hessian and TD-DFT gradient,  ECA, NRVS
  Petra Pikulova         : Analytic Raman intensities
  Peter Pinski           : DLPNO-MP2, DLPNO-MP2 Gradient
  Shashank Vittal Rao    : ES-AILFT, MagRelax
  Christoph Reimann      : Effective Core Potentials
  Marius Retegan         : Local ZFS, SOC
  Christoph Riplinger    : Optimizer, TS searches, QM/MM, DLPNO-CCSD(T), (RO)-DLPNO pert.
Triples
```

```
  Michael Roemelt        : Original ROCIS implementation, recursive CI coupling coefficients
  Masaaki Saitow         : Open-shell DLPNO-CCSD energy and density
  Barbara Sandhoefer     : DKH picture change effects
  Yorick L. A. Schmerwitz: GMF and freeze-and-release deltaSCF, NEB S-IDPP initial path
  Kantharuban Sivalingam : CASSCF convergence/infrastructure, NEVPT2, NEVPT3, NEVPT4(SD), FIC-
MRCI and CEPA variants
  Bernardo de Souza      : ESD, SOC TD-DFT
  Georgi L. Stoychev     : AutoAux, RI-MP2 NMR, DLPNO-MP2 response, X2C
  Van Anh Tran           : RI-MP2 g-tensors
  Willem Van den Heuvel  : Paramagnetic NMR
  Zikuan Wang            : NOTCH, Electric field optimization
  Frank Wennmohs         : Technical directorship and infrastructure
  Hang Xu                : AUTO-CI-Response properties

[FACCTs GmbH]
  Markus Bursch, Nicolas Foglia, Miquel Garcia-Rates, Ingolf Harden, Hagen Neugebauer,
Anastasios Papadopoulos,
  Christoph Riplinger, Bernardo de Souza, Georgi L. Stoychev

  APM, various basis sets, CI-OPT, improved COSX, DLPNO-Multilevel,
  DOCKER, DRACO, updates on ESD, Fragmentator, GOAT, IRC, LR-CPCM, L-BFGS, MBIS, meta-GGA TD-
DFT gradient, ML-optimized integration grids,
  MM, NACMEs, nearIR, NEB, NEB-TS, NL-DFT gradient (VV10), 2- and 3-layer-ONIOM, interface
openCOSMO-RS, QMMM,
  Crystal-QMMM, RESP, rigid body optimization, SF, symmetry and pop. for TD-DFT, various
functionals, SOLVATOR

[Other institutions]
  V. Asgeirsson          : NEB
  Christoph Bannwarth    : sTDA-DFT, sTD-DFT, PBEh-3c, B97-3c, D3
  Giovanni Bistoni       : ETS/NOCV, ADLD/ADEX, COVALED
  Martin Brehm           : Molecular dynamics
  Ronald Cardenas        : ETS/NOCV
  Martina Colucci        : COVALED
  Sebastian Ehlert       : rSCAN, r2SCAN, r2SCAN-3c, D4, dhf basis sets
  Marvin Friede          : D4 for Fr, Ra, Ac-Lr
  Lars Goerigk           : TD-DFT with DH, B97 family of functionals
  Stefan Grimme          : VdW corrections, initial TS optimization, DFT functionals, gCP,
sTDA/sTD-DF
  Waldemar Hujo          : DFT-NL
  H. Jonsson             : NEB
  Holger Kruse           : gCP
  Marcel Mueller         : wB97X-3c, vDZP basis set
  Gianluca Regni         : ADLD/ADEX
  Tobias Risthaus        : pre 6.0 range-separated hybrid DFT and stability analysis
  Lukas Wittmann         : regularized MP2, r2SCAN double-hybrids, wr2SCAN

We gratefully acknowledge several colleagues who have allowed us to
interface, adapt or use parts of their codes:
  Ed Valeev, F. Pavosevic, A. Kumar             : LibInt (2-el integral package), F12 methods
  Garnet Chan, S. Sharma, J. Yang, R. Olivares  : DMRG
  Ulf Ekstrom                                   : XCFun DFT Library
  Mihaly Kallay                                 : mrcc  (arbitrary order and MRCC methods)
  Frank Weinhold                                : gennbo (NPA and NBO analysis)
  Simon Mueller                                 : openCOSMO-RS
  Christopher J. Cramer and Donald G. Truhlar   : smd solvation model
  S Lehtola, MJT Oliveira, MAL Marques          : LibXC Library
  Liviu Ungur et al                             : ANISO software


 Your calculation uses the libint2 library for the computation of 2-el integrals
 For citations please refer to: http://libint.valeyev.net
```

```
 Your ORCA version has been built with support for libXC version: 7.0.0
 For citations please refer to: https://libxc.gitlab.io

 This ORCA versions uses:
   CBLAS   interface :  Fast vector & matrix operations
   LAPACKE interface :  Fast linear algebra routines
   SCALAPACK package :  Parallel linear algebra routines
   Shared memory     :  Shared parallel matrices
   BLAS/LAPACK       :  OpenBLAS 0.3.26  USE64BITINT DYNAMIC_ARCH NO_AFFINITY Haswell
SINGLE_THREADED
        Core in use  :  Haswell
   Copyright (c) 2011-2014, The OpenBLAS Project


  ***********************************
  * Starting time: Mon Nov 24 10:56:53 2025
  * Host name:     xe30th86.mpi-muelheim.mpg.de
  * Process ID:    3401375
  * Working dir.:  /usr/scratch/barasits/LQA_TS-OMe__26790259
  ***********************************

NOTE: MaxCore=8000 MB was set to SCF,MP2,MDCI,CIPSI,MRCI,RASCI and CIS
      => If you want to overwrite this, your respective input block should be placed after the
MaxCore statement
Your calculation utilizes the atom-pairwise dispersion correction
with the Becke-Johnson damping scheme (D3BJ)


Your calculation utilizes the SMD solvation module
Please cite in your paper:
Marenich, A. V.; Cramer, C. J.; Truhlar, D. G. J. Phys. Chem. B 2009, 113, 6378

================================================================================

----- Orbital basis set information -----
Your calculation utilizes the basis: def2-TZVP
   F. Weigend and R. Ahlrichs, Phys. Chem. Chem. Phys. 7, 3297 (2005).

----- AuxJ basis set information -----
Your calculation utilizes the auxiliary basis: def2/J
   H-Rn: F. Weigend, Phys. Chem. Chem. Phys. 8, 1057 (2006).
   Fr-Lr: K. Eichkorn, F. Weigend, O. Treutler, R. Ahlrichs; Theor. Chem. Acc. 97, 119 (1997).

================================================================================
                                        WARNINGS
                       Please study these warnings very carefully!
================================================================================

WARNING: SinglePoint found together with IRC
         Switching on EnGrad - energy plus gradient calculation

================================================================================
                                       INPUT FILE
================================================================================
NAME = LQA_TS-OMe.inp
|  1> #
|  2> #IRC LQA
|  3> #
|  4> ! irc B3LYP D3BJ def2-TZVP def2/J verytightscf
|  5> %pal nprocs 12 end
|  6> %maxcore 8000
|  7> %irc
|  8> printlevel 1
```

```
|  9> method lqa
| 10> maxiter 241
| 11> tolrmsg 1.e-5 # Tighter convergence criteria
| 12> tolmaxg 8.e-5 # Tighter convergence criteria
| 13> follow_coordtype massweighted
| 14> inithess read
| 15> hess_filename "TS-OMe_Compound_2.hess"
| 16> lqahess lqacalc_bofillupdate
| 17> init_displ length
| 18> scale_init_displ 0.04
| 19> adapt_scale_displ false
| 20> scale_displ_sd 0.11
| 21> ENFORCESTRICTCONVERGENCE true
| 22> REPORTPROFILE true
| 23> BSCORRECTOR true
| 24> BSRICHTHRESH 1E-6
| 25> INITBSCORRECTOR false
| 26> end
| 27> %CPCM SMD true
| 28>      SMDSOLVENT "TETRAHYDROFURAN" END
| 29>
| 30> * xyz 0 1
| 31>   C         -1.68315636183039      0.33021797821882     -6.00104503996402
| 32>   C         -1.15437514331283     -0.44500766737758     -4.96804317601567
| 33>   C         -0.86588518632462      1.29977835626813     -6.58635676629494
| 34>   C          0.14874465315648     -0.25102550801711     -4.52460586592678
| 35>   C          0.44130807437946      1.49254053811229     -6.15193709010013
| 36>   C          0.95256833981509      0.71736172100065     -5.11627918694672
| 37>   H         -1.77114517333674     -1.20821614435879     -4.50931618711464
| 38>   H         -1.26209685498840      1.91214895744245     -7.38827498289601
| 39>   H          0.53991373633812     -0.86180714624003     -3.72135378305798
| 40>   H          1.05919323314974      2.24900707476585     -6.62048688542392
| 41>   H          1.97084626672060      0.86146264781351     -4.77722884489171
| 42>   C         -3.09398998414558      0.12223807790296     -6.48307596109776
| 43>   H         -3.48993623341992      1.08173342081102     -6.82984356461734
| 44>   H         -3.71937766911073     -0.18765639425529     -5.64278345830879
| 45>   C         -3.25582524487063     -0.90433612181464     -7.61540188432257
| 46>   H         -3.14948008017975     -2.08585798727212     -7.04926469362890
| 47>   H         -4.28352930379314     -0.96497131388039     -7.97551611887916
| 48>   C         -2.28034716264576     -1.05494097326939     -8.61911657535221
| 49>   H         -2.56983154310193     -1.30876819316868     -9.62957070327348
| 50>   H         -1.29806840325315     -0.61191234282496     -8.51867529265622
| 51>   Se        -1.56164844326017     -3.42208787670288     -8.08812530896050
| 52>   O         -2.75233062577177     -3.31930806276004     -6.85076752101363
| 53>   C          0.07649781719434     -3.06086257644294     -7.13152451784482
| 54>   C          0.30429315864657     -3.64665169196715     -5.89352519512593
| 55>   C          1.06350222235541     -2.26727098625819     -7.71494384255497
| 56>   C          1.50217640999906     -3.43163360834534     -5.22115457685014
| 57>   C          2.26229805530533     -2.05581466482009     -7.05693478573442
| 58>   C          2.48628403457500     -2.62933870508388     -5.80160102559058
| 59>   H         -0.45891406702091     -4.26309371006052     -5.43661504833684
| 60>   H          0.89354158719584     -1.80050255999083     -8.67708819453704
| 61>   H          1.65276260225657     -3.88325647363266     -4.25186781494243
| 62>   H          3.02857914509606     -1.42651655328168     -7.49096203043131
| 63>   O          3.67592534496859     -2.34097932228469     -5.21915029791983
| 64>   C          3.94427180234322     -2.88084797351211     -3.92672778618338
| 65>   H          3.21333116868397     -2.53228366544859     -3.19269104867936
| 66>   H          3.95096359281910     -3.97371028207636     -3.94699089740054
| 67>   H          4.93260688536786     -2.51930227718852     -3.65142337712425
| 68> *
| 69>
| 70>                          ****END OF INPUT****
================================================================================
```

```
                     ******************************
                     * Energy+Gradient Calculation *
                     ******************************

---------------------------------
CARTESIAN COORDINATES (ANGSTROEM)
---------------------------------
  C     -1.683156    0.330218   -6.001045
  C     -1.154375   -0.445008   -4.968043
  C     -0.865885    1.299778   -6.586357
  C      0.148745   -0.251026   -4.524606
  C      0.441308    1.492541   -6.151937
  C      0.952568    0.717362   -5.116279
  H     -1.771145   -1.208216   -4.509316
  H     -1.262097    1.912149   -7.388275
  H      0.539914   -0.861807   -3.721354
  H      1.059193    2.249007   -6.620487
  H      1.970846    0.861463   -4.777229
  C     -3.093990    0.122238   -6.483076
  H     -3.489936    1.081733   -6.829844
  H     -3.719378   -0.187656   -5.642783
  C     -3.255825   -0.904336   -7.615402
  H     -3.149480   -2.085858   -7.049265
  H     -4.283529   -0.964971   -7.975516
  C     -2.280347   -1.054941   -8.619117
  H     -2.569832   -1.308768   -9.629571
  H     -1.298068   -0.611912   -8.518675
  Se    -1.561648   -3.422088   -8.088125
  O     -2.752331   -3.319308   -6.850768
  C      0.076498   -3.060863   -7.131525
  C      0.304293   -3.646652   -5.893525
  C      1.063502   -2.267271   -7.714944
  C      1.502176   -3.431634   -5.221155
  C      2.262298   -2.055815   -7.056935
  C      2.486284   -2.629339   -5.801601
  H     -0.458914   -4.263094   -5.436615
  H      0.893542   -1.800503   -8.677088
  H      1.652763   -3.883256   -4.251868
  H      3.028579   -1.426517   -7.490962
  O      3.675925   -2.340979   -5.219150
  C      3.944272   -2.880848   -3.926728
  H      3.213331   -2.532284   -3.192691
  H      3.950964   -3.973710   -3.946991
  H      4.932607   -2.519302   -3.651423

----------------------------
CARTESIAN COORDINATES (A.U.)
----------------------------
  NO LB      ZA    FRAG     MASS         X           Y           Z
   0 C     6.0000    0    12.011   -3.180705    0.624022  -11.340332
   1 C     6.0000    0    12.011   -2.181453   -0.840943   -9.388241
   2 C     6.0000    0    12.011   -1.636286    2.456225  -12.446411
   3 C     6.0000    0    12.011    0.281087   -0.474369   -8.550266
   4 C     6.0000    0    12.011    0.833951    2.820493  -11.625476
   5 C     6.0000    0    12.011    1.800093    1.355617   -9.668366
   6 H     1.0000    0     1.008   -3.346979   -2.283198   -8.521373
   7 H     1.0000    0     1.008   -2.385017    3.613438  -13.961816
   8 H     1.0000    0     1.008    1.020289   -1.628579   -7.032339
   9 H     1.0000    0     1.008    2.001585    4.250007  -12.510907
  10 H     1.0000    0     1.008    3.724360    1.627928   -9.027654
  11 C     6.0000    0    12.011   -5.846794    0.230996  -12.251238
  12 H     1.0000    0     1.008   -6.595024    2.044180  -12.906534
```

13	H	1.0000	0	1.008	-7.028605	-0.354619	-10.663315
14	C	6.0000	0	12.011	-6.152618	-1.708948	-14.391024
15	H	1.0000	0	1.008	-5.951655	-3.941700	-13.321180
16	H	1.0000	0	1.008	-8.094697	-1.823532	-15.071541
17	C	6.0000	0	12.011	-4.309232	-1.993550	-16.287770
18	H	1.0000	0	1.008	-4.856278	-2.473213	-18.197251
19	H	1.0000	0	1.008	-2.452994	-1.156347	-16.097963
20	Se	34.0000	0	78.960	-2.951088	-6.466809	-15.284342
21	O	8.0000	0	15.999	-5.201151	-6.272583	-12.946074
22	C	6.0000	0	12.011	0.144560	-5.784192	-13.476628
23	C	6.0000	0	12.011	0.575031	-6.891173	-11.137149
24	C	6.0000	0	12.011	2.009728	-4.284521	-14.579131
25	C	6.0000	0	12.011	2.838702	-6.484848	-9.866552
26	C	6.0000	0	12.011	4.275124	-3.884927	-13.335674
27	C	6.0000	0	12.011	4.698396	-4.968730	-10.963437
28	H	1.0000	0	1.008	-0.867222	-8.056080	-10.273714
29	H	1.0000	0	1.008	1.688549	-3.402457	-16.397320
30	H	1.0000	0	1.008	3.123269	-7.338291	-8.034866
31	H	1.0000	0	1.008	5.723185	-2.695726	-14.155867
32	O	8.0000	0	15.999	6.946492	-4.423810	-9.862765
33	C	6.0000	0	12.011	7.453594	-5.444014	-7.420440
34	H	1.0000	0	1.008	6.072316	-4.785323	-6.033312
35	H	1.0000	0	1.008	7.466239	-7.509224	-7.458732
36	H	1.0000	0	1.008	9.321276	-4.760791	-6.900190

INTERNAL COORDINATES (ANGSTROEM)

C	0	0	0	0.000000000000	0.00000000	0.00000000
C	1	0	0	1.395592071333	0.00000000	0.00000000
C	1	2	0	1.396627838036	118.30141620	0.00000000
C	2	1	3	1.390103208427	120.91645811	0.51055659
C	3	1	2	1.390910469673	121.01549055	359.88063881
C	4	2	1	1.390677411683	120.21809764	359.44702430
H	2	1	3	1.083200304476	119.28200599	180.59570554
H	3	1	2	1.083999200039	119.23027009	180.15405128
H	4	2	1	1.082257533982	119.79030419	179.92588306
H	5	3	1	1.083310943914	119.84959584	179.90361127
H	6	4	2	1.082871251802	120.19754652	180.61136066
C	1	2	3	1.505344132655	121.02271885	179.49225392
H	12	1	2	1.094373115636	108.62771061	148.71940428
H	12	1	2	1.092353345133	109.23487531	32.83666634
C	12	1	2	1.536947375660	115.26975086	270.42934830
H	15	12	1	1.314463590707	105.93504821	76.24531672
H	15	12	1	1.090657838725	112.30944984	184.09571714
C	15	12	1	1.407722448202	121.58691170	324.86079369
H	18	15	12	1.081317256277	120.38986486	214.10072944
H	18	15	12	1.082235824493	121.26364206	13.09641713
Se	16	15	12	2.320765176121	112.33961422	232.78089864
O	16	15	12	1.310926353917	159.30333134	227.82410837
C	21	16	15	1.931085695082	104.53358885	91.18152440
C	23	21	16	1.388409856097	120.12993371	78.28924427
C	23	21	16	1.394397151555	119.97723493	254.89483025
C	24	23	21	1.390409762795	120.48379557	177.58258200
C	25	23	21	1.383763398790	120.08142163	182.87613849
C	26	24	23	1.396088467255	119.67712130	359.47757803
H	24	23	21	1.082244387013	120.07069513	356.58950841
H	25	23	21	1.082811680448	120.39825125	3.78708592
H	26	24	23	1.079887142510	119.30861255	178.98830118
H	27	25	23	1.082396632892	120.76267752	181.17433541
O	28	26	24	1.355598182061	124.20880128	178.86027402
C	33	28	26	1.426121990086	118.29333588	359.58514336
H	34	33	28	1.092968992605	111.22738607	299.33440159

```
 H     34  33  28     1.093070628650   111.27453291    61.83892957
 H     34  33  28     1.087802392218   105.93207044   180.59136677

---------------------------
INTERNAL COORDINATES (A.U.)
---------------------------
 C      0   0   0     0.000000000000     0.00000000     0.00000000
 C      1   0   0     2.637286809492     0.00000000     0.00000000
 C      1   2   0     2.639244124898   118.30141620     0.00000000
 C      2   1   3     2.626914361812   120.91645811     0.51055659
 C      3   1   2     2.628439864485   121.01549055   359.88063881
 C      4   2   1     2.627999448711   120.21809764   359.44702430
 H      2   1   3     2.046951923641   119.28200599   180.59570554
 H      3   1   2     2.048461617463   119.23027009   180.15405128
 H      4   2   1     2.045170345599   119.79030419   179.92588306
 H      5   3   1     2.047161001877   119.84959584   179.90361127
 H      6   4   2     2.046330104202   120.19754652   180.61136066
 C      1   2   3     2.844688148024   121.02271885   179.49225392
 H     12   1   2     2.068065476879   108.62771061   148.71940428
 H     12   1   2     2.064248663774   109.23487531    32.83666634
 C     12   1   2     2.904409622245   115.26975086   270.42934830
 H     15  12   1     2.483976199447   105.93504821    76.24531672
 H     15  12   1     2.061044621005   112.30944984   184.09571714
 C     15  12   1     2.660209899675   121.58691170   324.86079369
 H     18  15  12     2.043393478247   120.38986486   214.10072944
 H     18  15  12     2.045129320611   121.26364206    13.09641713
 Se    16  15  12     4.385610604011   112.33961422   232.78089864
 O     16  15  12     2.477291790643   159.30333134   227.82410837
 C     21  16  15     3.649223104839   104.53358885    91.18152440
 C     23  21  16     2.623714389660   120.12993371    78.28924427
 C     23  21  16     2.635028738359   119.97723493   254.89483025
 C     24  23  21     2.627493665613   120.48379557   177.58258200
 C     25  23  21     2.614933857858   120.08142163   182.87613849
 C     26  24  23     2.638224861838   119.67712130   359.47757803
 H     24  23  21     2.045145501428   120.07069513   356.58950841
 H     25  23  21     2.046217530658   120.39825125     3.78708592
 H     26  24  23     2.040690954887   119.30861255   178.98830118
 H     27  25  23     2.045433204444   120.76267752   181.17433541
 O     28  26  24     2.561709311737   124.20880128   178.86027402
 C     33  28  26     2.694979994825   118.29333588   359.58514336
 H     34  33  28     2.065412068891   111.22738607   299.33440159
 H     34  33  28     2.065604133181   111.27453291    61.83892957
 H     34  33  28     2.055648609116   105.93207044   180.59136677

---------------------
BASIS SET INFORMATION
---------------------
There are 4 groups of distinct atoms

 Group   1 Type C   : 11s6p2d1f contracted to 5s3p2d1f pattern {62111/411/11/1}
 Group   2 Type H   : 5s1p contracted to 3s1p pattern {311/1}
 Group   3 Type Se  : 17s13p8d1f contracted to 6s5p4d1f pattern {842111/64111/5111/1}
 Group   4 Type O   : 11s6p2d1f contracted to 5s3p2d1f pattern {62111/411/11/1}

Atom   0C    basis set group =>   1
Atom   1C    basis set group =>   1
Atom   2C    basis set group =>   1
Atom   3C    basis set group =>   1
Atom   4C    basis set group =>   1
Atom   5C    basis set group =>   1
Atom   6H    basis set group =>   2
Atom   7H    basis set group =>   2
Atom   8H    basis set group =>   2
```

```
Atom   9H    basis set group =>   2
Atom  10H    basis set group =>   2
Atom  11C    basis set group =>   1
Atom  12H    basis set group =>   2
Atom  13H    basis set group =>   2
Atom  14C    basis set group =>   1
Atom  15H    basis set group =>   2
Atom  16H    basis set group =>   2
Atom  17C    basis set group =>   1
Atom  18H    basis set group =>   2
Atom  19H    basis set group =>   2
Atom  20Se   basis set group =>   3
Atom  21O    basis set group =>   4
Atom  22C    basis set group =>   1
Atom  23C    basis set group =>   1
Atom  24C    basis set group =>   1
Atom  25C    basis set group =>   1
Atom  26C    basis set group =>   1
Atom  27C    basis set group =>   1
Atom  28H    basis set group =>   2
Atom  29H    basis set group =>   2
Atom  30H    basis set group =>   2
Atom  31H    basis set group =>   2
Atom  32O    basis set group =>   4
Atom  33C    basis set group =>   1
Atom  34H    basis set group =>   2
Atom  35H    basis set group =>   2
Atom  36H    basis set group =>   2
---------------------------------
AUXILIARY/J BASIS SET INFORMATION
---------------------------------
There are 4 groups of distinct atoms

 Group   1 Type C   : 12s5p4d2f1g contracted to 6s4p3d1f1g pattern {711111/2111/211/2/1}
 Group   2 Type H   : 5s2p1d contracted to 3s1p1d pattern {311/2/1}
 Group   3 Type Se  : 19s5p5d3f1g contracted to 8s4p3d2f1g pattern {121111111/2111/311/21/1}
 Group   4 Type O   : 12s5p4d2f1g contracted to 6s4p3d1f1g pattern {711111/2111/211/2/1}

Atom   0C    basis set group =>   1
Atom   1C    basis set group =>   1
Atom   2C    basis set group =>   1
Atom   3C    basis set group =>   1
Atom   4C    basis set group =>   1
Atom   5C    basis set group =>   1
Atom   6H    basis set group =>   2
Atom   7H    basis set group =>   2
Atom   8H    basis set group =>   2
Atom   9H    basis set group =>   2
Atom  10H    basis set group =>   2
Atom  11C    basis set group =>   1
Atom  12H    basis set group =>   2
Atom  13H    basis set group =>   2
Atom  14C    basis set group =>   1
Atom  15H    basis set group =>   2
Atom  16H    basis set group =>   2
Atom  17C    basis set group =>   1
Atom  18H    basis set group =>   2
Atom  19H    basis set group =>   2
Atom  20Se   basis set group =>   3
Atom  21O    basis set group =>   4
Atom  22C    basis set group =>   1
Atom  23C    basis set group =>   1
Atom  24C    basis set group =>   1
```

```
Atom  25C    basis set group =>   1
Atom  26C    basis set group =>   1
Atom  27C    basis set group =>   1
Atom  28H    basis set group =>   2
Atom  29H    basis set group =>   2
Atom  30H    basis set group =>   2
Atom  31H    basis set group =>   2
Atom  32O    basis set group =>   4
Atom  33C    basis set group =>   1
Atom  34H    basis set group =>   2
Atom  35H    basis set group =>   2
Atom  36H    basis set group =>   2


           ************************************************************
           *        Program running with 12 parallel MPI-processes    *
           *              working on a common directory               *
           ************************************************************
------------------------------------------------------------------------------
                            ORCA STARTUP CALCULATIONS
                          -- RI-GTO INTEGRALS CHOSEN --
------------------------------------------------------------------------------
------------------------------------------------------------------------------
                   ___
                  /   \      - P O W E R E D   B Y -
                 /     \
                 |  |  |   _    _      __       _____    __    __
                 |  |  |  | |  | |    /  \     |  _  \  |  |  /  |
                  \  \/   | |  | |   /    \    | | | |  |  | /  /
                 / \  \   | |__| |  /  /\  \   | |_| |  |  |/  /
                |  |  |   |  __  | /  /__\  \  |    /   |      \
                |  |  |   | |  | | |   __   |  |    \   |  |\   \
                \     /   | |  | | |  |  |  |  | |\  \  |  |  \  \
                 \___/    |_|  |_| |__|  |__|  |_| \__\ |__|   \__/

                      - O R C A' S   B I G   F R I E N D -
                                      &
                       - I N T E G R A L   F E E D E R -

 v1 FN, 2020, v2 2021, v3 2022-2024
------------------------------------------------------------------------------


----------------------
SHARK INTEGRAL PACKAGE
----------------------

Number of atoms                             ...     37
Number of basis functions                   ...    714
Number of shells                            ...    286
Maximum angular momentum                    ...      3
Integral batch strategy                     ... SHARK/LIBINT Hybrid
RI-J (if used) integral strategy            ... SPLIT-RIJ (Revised 2003 algorithm where
possible)
Printlevel                                  ...      1
Contraction scheme used                     ... SEGMENTED contraction
Prescreening option                         ... SCHWARTZ
   Thresh                                   ... 1.000e-12
   Tcut                                     ... 1.000e-14
   Tpresel                                  ... 1.000e-14
Coulomb Range Separation                    ... NOT USED
Exchange Range Separation                   ... NOT USED
Multipole approximations                    ... NOT USED
```

```
Finite Nucleus Model                         ... NOT USED
CABS basis                                   ... NOT available
Auxiliary Coulomb fitting basis              ... AVAILABLE
   # of basis functions in Aux-J             ...   1138
   # of shells in Aux-J                      ...    378
   Maximum angular momentum in Aux-J         ...      4
Auxiliary J/K fitting basis                  ... NOT available
Auxiliary Correlation fitting basis          ... NOT available
Auxiliary 'external' fitting basis           ... NOT available

Checking pre-screening integrals             ... done (  0.0 sec) Dimension = 286
   => SHARK Basis and OBASIS are compatible. Storing Pre-screening
Shell pair information
Shell pair cut-off parameter TPreSel         ...   1.0e-14
Total number of shell pairs                  ...     41041
Shell pairs after pre-screening              ...     33504
Total number of primitive shell pairs        ...    129294
Primitive shell pairs kept                   ...     71134
          la=0 lb=0:   8848 shell pairs
          la=1 lb=0:   9578 shell pairs
          la=1 lb=1:   2634 shell pairs
          la=2 lb=0:   4852 shell pairs
          la=2 lb=1:   2650 shell pairs
          la=2 lb=2:    699 shell pairs
          la=3 lb=0:   2191 shell pairs
          la=3 lb=1:   1248 shell pairs
          la=3 lb=2:    645 shell pairs
          la=3 lb=3:    159 shell pairs

Checking whether 4 symmetric matrices of dimension 714 fit in memory:
Max Core in MB      =   8000.00
 MB in use           =     44.56
 MB left             =   7955.44
 MB needed           =      7.79
 Data fit in memory  = YES
Calculating RI/J V-Matrix + Cholesky decomp.... done (  0.1 sec)
Calculating Nuclear repulsion                ... done (  0.0 sec) ENN=   1907.685046068582 Eh

Diagonalization of the overlap matrix:
Smallest eigenvalue                         ... 6.192e-06
Time for diagonalization                    ...    0.097 sec
Threshold for overlap eigenvalues           ... 1.000e-07
Number of eigenvalues below threshold       ... 0
Time for construction of square roots       ...    0.056 sec
Total time needed                           ...    0.158 sec

-------------------
DFT GRID GENERATION
-------------------

General Integration Accuracy     IntAcc      ... 4.388
Radial Grid Type                 RadialGrid  ... OptM3 with GC (2021)
Angular Grid (max. ang.)         AngularGrid ... 4 (Lebedev-302)
Angular grid pruning method      GridPruning ... 4 (adaptive)
Weight generation scheme         WeightScheme... mBecke (2022)
Basis function cutoff            BFCut       ... 1.0000e-12
Integration weight cutoff        WCut        ... 1.0000e-14
Partially contracted basis set               ... off
Rotationally invariant grid construction     ... off
Angular grids for H and He will be reduced by one unit
Core-polarized basis detected: some atoms will have their core
                               angular grid increased.
```

```
Total number of grid points                  ...   175709
Total number of batches                      ...     2764
Average number of points per batch           ...       63
Average number of grid points per atom       ...     4749

--------------------
COSX GRID GENERATION
--------------------

GRIDX 1
-------
General Integration Accuracy     IntAcc      ... 3.816
Radial Grid Type                 RadialGrid  ... OptM3 with GC (2021)
Angular Grid (max. ang.)         AngularGrid ... 1 (Lebedev-50)
Angular grid pruning method      GridPruning ... 4 (adaptive)
Weight generation scheme         WeightScheme... mBecke (2022)
Basis function cutoff            BFCut       ... 1.0000e-12
Integration weight cutoff        WCut        ... 1.0000e-14
Partially contracted basis set               ... on
Rotationally invariant grid construction     ... off
Angular grids for H and He will be reduced by one unit
Core-polarized basis detected: some atoms will have their core
                               angular grid increased.

Total number of grid points                  ...    26804
Total number of batches                      ...      229
Average number of points per batch           ...      117
Average number of grid points per atom       ...      724
UseSFitting                                  ... on

GRIDX 2
-------
General Integration Accuracy     IntAcc      ... 4.020
Radial Grid Type                 RadialGrid  ... OptM3 with GC (2021)
Angular Grid (max. ang.)         AngularGrid ... 2 (Lebedev-110)
Angular grid pruning method      GridPruning ... 4 (adaptive)
Weight generation scheme         WeightScheme... mBecke (2022)
Basis function cutoff            BFCut       ... 1.0000e-12
Integration weight cutoff        WCut        ... 1.0000e-14
Partially contracted basis set               ... on
Rotationally invariant grid construction     ... off
Angular grids for H and He will be reduced by one unit
Core-polarized basis detected: some atoms will have their core
                               angular grid increased.

Total number of grid points                  ...    50594
Total number of batches                      ...      415
Average number of points per batch           ...      121
Average number of grid points per atom       ...     1367
UseSFitting                                  ... on

GRIDX 3
-------
General Integration Accuracy     IntAcc      ... 4.338
Radial Grid Type                 RadialGrid  ... OptM3 with GC (2021)
Angular Grid (max. ang.)         AngularGrid ... 3 (Lebedev-194)
Angular grid pruning method      GridPruning ... 4 (adaptive)
Weight generation scheme         WeightScheme... mBecke (2022)
Basis function cutoff            BFCut       ... 1.0000e-12
Integration weight cutoff        WCut        ... 1.0000e-14
Partially contracted basis set               ... on
Rotationally invariant grid construction     ... off
Angular grids for H and He will be reduced by one unit
```

```
Core-polarized basis detected: some atoms will have their core
                               angular grid increased.

Total number of grid points                  ...   103309
Total number of batches                      ...      824
Average number of points per batch           ...      125
Average number of grid points per atom       ...     2792
UseSFitting                                  ... on
Grids setup in   2.7 sec
Initializing property integral containers    ... done (  0.0 sec)

SHARK setup successfully completed in   3.5 seconds

Maximum memory used throughout the entire STARTUP-calculation: 87.0 MB


           ************************************************************
           *        Program running with 12 parallel MPI-processes    *
           *              working on a common directory               *
           ************************************************************
------------------------------------------------------------------------------
                                 ORCA GUESS
                   Start orbitals & Density for SCF / CASSCF
------------------------------------------------------------------------------


------------
SCF SETTINGS
------------
Hamiltonian:
 Density Functional     Method          .... DFT(GTOs)
 Exchange Functional    Exchange        .... B88
   X-Alpha parameter    XAlpha          ....  0.666667
   Becke's b parameter  XBeta           ....  0.004200
 Correlation Functional Correlation     .... LYP
 LDA part of GGA corr.  LDAOpt          .... VWN-5
 Gradients option       PostSCFGGA      .... off
 Hybrid DFT is turned on
   Fraction HF Exchange ScalHFX         ....  0.200000
   Scaling of DF-GGA-X  ScalDFX         ....  0.720000
   Scaling of DF-GGA-C  ScalDFC         ....  0.810000
   Scaling of DF-LDA-C  ScalLDAC        ....  1.000000
   Perturbative correction              ....  0.000000
   NL short-range parameter             ....  4.800000
 RI-approximation to the Coulomb term is turned on
   Number of AuxJ basis functions       .... 1138
   RIJ-COSX (HFX calculated with COS-X)).... on


General Settings:
 Integral files         IntName         .... LQA_TS-OMe
 Hartree-Fock type      HFTyp           .... RHF
 Total Charge           Charge          ....    0
 Multiplicity           Mult            ....    1
 Number of Electrons    NEL             ....  164
 Basis Dimension        Dim             ....  714
 Nuclear Repulsion      ENuc            ....   1907.6850460686 Eh

Convergence Acceleration:
 AO-DIIS                CNVDIIS         .... on
   Start iteration      DIISMaxIt       ....    12
   Startup error        DIISStart       ....  0.200000
   # of expansion vecs  DIISMaxEq       ....     5
   Bias factor          DIISBfac        ....   1.050
```

```
   Max. coefficient     DIISMaxC        ....  10.000
 MO-DIIS                CNVKDIIS        .... off
 Trust-Rad. Augm. Hess. CNVTRAH         .... auto
   Auto Start mean grad. ratio tolernc. ....  1.125000
   Auto Start start iteration           ....    50
   Auto Start num. interpolation iter.  ....    10
   Max. Number of Micro iterations      ....    24
   Max. Number of Macro iterations      .... Maxiter - #DIIS iter
   Number of Davidson start vectors     ....     2
   Converg. threshold    (grad. norm)   ....   2.000e-06
   Grad. Scal. Fac. for Micro threshold ....   0.100
   Minimum threshold for Micro iter.    ....   1.000e-02
   NR start threshold (gradient norm)   ....   1.000e-04
   Initial trust radius                 ....   0.400
   Minimum AH scaling param. (alpha)    ....   1.000
   Maximum AH scaling param. (alpha)    .... 1000.000
   Quad. conv. algorithm                .... NR
   White noise on init. David. guess    .... on
   Maximum white noise                  ....   0.010
   Pseudo random numbers                .... off
   Inactive MOs                         .... canonical
   Orbital update algorithm             .... Taylor
   Preconditioner                       .... Diag
   Full preconditioner red. dimension   ....   250
 SOSCF                 CNVSOSCF           .... on
   Start iteration     SOSCFMaxIt         ....   150
   Startup grad/error  SOSCFStart         ....  0.003300
   Hessian update      SOSCFHessUp        .... L-BFGS
   Autom. constraints  SOSCFAutoConstrain .... off
 Level Shifting         CNVShift        .... on
   Level shift para.    LevelShift      ....    0.2500
   Turn off err/grad.   ShiftErr        ....    0.0010
 Zerner damping         CNVZerner       .... off
 Static damping         CNVDamp         .... on
   Fraction old density DampFac         ....    0.7000
   Max. Damping (<1)    DampMax         ....    0.9800
   Min. Damping (>=0)   DampMin         ....    0.0000
   Turn off err/grad.   DampErr         ....    0.1000

SCF Procedure:
 Maximum # iterations   MaxIter         ....   125
 SCF integral mode      SCFMode         .... Direct
   Integral package                     .... SHARK and LIBINT hybrid scheme
 Reset frequency        DirectResetFreq ....    20
 Integral Threshold     Thresh          ....  1.000e-12 Eh
 Primitive CutOff       TCut            ....  1.000e-14 Eh

Convergence Tolerance:
 Convergence Check Mode ConvCheckMode   .... Total+1el-Energy
 Convergence forced     ConvForced      .... 0
 Energy Change          TolE            ....  1.000e-09 Eh
 1-El. energy change                    ....  1.000e-06 Eh
 Orbital Gradient       TolG            ....  2.000e-06
 Orbital Rotation angle TolX            ....  2.000e-06
 DIIS Error             TolErr          ....  1.000e-08

------------------------------
INITIAL GUESS: MODEL POTENTIAL
------------------------------
Loading Hartree-Fock densities                     ... done
Calculating cut-offs                               ... done
Initializing the effective Hamiltonian             ... done
Setting up the integral package (SHARK)            ... done
```

```
Starting the Coulomb interaction                  ... done (   0.4 sec)
Making the grid                                   ... done (   0.4 sec)
Mapping shells                                    ... done
Starting the XC term evaluation                   ... done (   0.3 sec)
  promolecular density results
     # of electrons  =    163.988199338
     EX              =   -189.629096546
     EC              =     -6.022979005
     EX+EC           =   -195.652075551
Transforming the Hamiltonian                      ... done (   0.1 sec)
Diagonalizing the Hamiltonian                     ... done (   0.1 sec)
Back transforming the eigenvectors                ... done (   0.0 sec)
Now organizing SCF variables                      ... done
                      ------------------
                      INITIAL GUESS DONE (   1.3 sec)
                      ------------------
             **** ENERGY FILE WAS UPDATED (LQA_TS-OMe.en.tmp) ****
Finished Guess after   1.7 sec
Maximum memory used throughout the entire GUESS-calculation: 51.0 MB


           ************************************************************
           *        Program running with 12 parallel MPI-processes    *
           *              working on a common directory               *
           ************************************************************

--------------------------------------------------------------------------------------------
                                     ORCA LEAN-SCF
                              memory conserving SCF solver
--------------------------------------------------------------------------------------------

--------------------
CPCM SOLVATION MODEL
--------------------
CPCM parameters:
  Epsilon                                         ...       7.4257
  Refrac                                          ...       1.4050
  Rsolv                                           ...       1.3000
  Surface type                                    ... GAUSSIAN VDW
  Discretization scheme                           ... Constant charge density
    Threshold for H atoms                         ...       5.0000 (charges/Ang^2)
    Threshold for non-H atoms                     ...       5.0000 (charges/Ang^2)
  Epsilon function type                           ...         CPCM
Solvent:                                          ... TETRAHYDROFURAN
SMD-CDS solvent descriptors:
  Soln                                            ...       1.4050
  Soln25                                          ...       1.4044
  Sola                                            ...       0.0000
  Solb                                            ...       0.4800
  Solg                                            ...      39.4400
  Solc                                            ...       0.0000
  Solh                                            ...       0.0000
Radii:
  Scheme                                          ... Element-dependent radii
  Radius for C  used is    3.4960 Bohr (=   1.8500 Ang.)
  Radius for H  used is    2.2677 Bohr (=   1.2000 Ang.)
  Radius for Se used is    3.5905 Bohr (=   1.9000 Ang.)
  Radius for O  used is    4.3350 Bohr (=   2.2940 Ang.)
Calculating surface                               ...        done! (  0.0s)
Cavity surface points                             ...         2879
Cavity Volume                                     ...    2133.4587
Cavity Surface-area                               ...    1152.1038
Calculating surface distance matrix               ...        done! (  0.1s)
```

```
Performing Cholesky decomposition & store         ...        done! (  0.2s)
Overall time for CPCM initialization              ...               0.3s

                      ----------------------------------------D-I-I-S--------------------------------------------
Iteration    Energy (Eh)           Delta-E    RMSDP     MaxDP     DIISErr   Damp  Time(sec)
---------------------------------------------------------------------------------------------
               ***  Starting incremental Fock matrix formation  ***
    1    -3171.5270887173355732     0.00e+00  7.36e-03  2.30e-01  3.16e-01  0.700  10.5
    2    -3171.8342378958459449    -3.07e-01  6.15e-03  1.96e-01  1.13e-01  0.700   6.6
                               ***Turning on AO-DIIS***
    3    -3171.9490933995330124    -1.15e-01  2.05e-03  5.77e-02  4.39e-02  0.700   6.3
    4    -3172.0187830054819642    -6.97e-02  3.16e-03  9.17e-02  2.07e-02  0.000   6.1
    5    -3172.1731275857773653    -1.54e-01  1.26e-03  3.89e-02  7.69e-03  0.000   6.3
                              *** Initializing SOSCF ***
---------------------------------------S-O-S-C-F--------------------------------------
Iteration    Energy (Eh)           Delta-E    RMSDP     MaxDP     MaxGrad    Time(sec)
--------------------------------------------------------------------------------------
    6    -3172.1743679181045081    -1.24e-03  4.54e-04  1.32e-02  3.11e-03     6.1
               *** Restarting incremental Fock matrix formation ***
    7    -3172.1744507973917280    -8.29e-05  4.94e-04  1.72e-02  4.95e-04    11.3
    8    -3172.1744370304959375     1.38e-05  1.53e-04  3.96e-03  6.41e-04     8.8
    9    -3172.1744684751556633    -3.14e-05  9.40e-05  3.10e-03  2.53e-04     7.8
   10    -3172.1744662462069755     2.23e-06  3.95e-05  1.43e-03  2.68e-04     8.0
   11    -3172.1744710856692109    -4.84e-06  1.22e-05  3.39e-04  3.07e-05     7.5
   12    -3172.1744710029188354     8.28e-08  6.10e-06  1.62e-04  3.99e-05     7.1
   13    -3172.1744711740384446    -1.71e-07  2.21e-06  7.61e-05  8.33e-06     6.7
   14    -3172.1744711724286390     1.61e-09  1.48e-06  5.55e-05  8.24e-06     6.0
   15    -3172.1744711763649320    -3.94e-09  3.89e-07  8.74e-06  1.97e-06     5.6
   16    -3172.1744711762130464     1.52e-10  2.86e-07  8.60e-06  2.20e-06     4.9
                 **** Energy Check signals convergence ****

               *****************************************************
               *                     SUCCESS                       *
               *           SCF CONVERGED AFTER  16 CYCLES          *
               *****************************************************

Recomputing exchange energy using gridx3       ... done (   15.020 sec)
Old exchange energy                            :    -38.249437329 Eh
New exchange energy                            :    -38.249442972 Eh
Exchange energy change after final integration :     -0.000005644 Eh
Total energy after final integration           :  -3172.174476820 Eh

SMD CDS free energy correction energy :                 -3.74720     Kcal/mol
Total Energy after SMD CDS correction =           -3172.180448366 Eh
             **** ENERGY FILE WAS UPDATED (LQA_TS-OMe.en.tmp) ****

----------------
TOTAL SCF ENERGY
----------------

Total Energy       :      -3172.18044836599620 Eh          -86319.41839 eV

Components:
Nuclear Repulsion  :       1907.68504606858164 Eh           51910.74920 eV
Electronic Energy  :      -5079.84176360686251 Eh         -138229.52184 eV
One Electron Energy:      -8185.70613342774504 Eh         -222744.38800 eV
Two Electron Energy:       3105.86436982088298 Eh           84514.86616 eV
CPCM Dielectric    :         -0.01775363795643 Eh              -0.48310 eV
SMD CDS (Gcds)     :         -0.00597154625304 Eh              -0.16249 eV

Virial components:
Potential Energy   :      -6337.29756206525508 Eh         -172446.63368 eV
Kinetic Energy     :       3165.11711369925888 Eh           86127.21529 eV
```

```
Virial Ratio       :          2.00223161874048

DFT components:
N(Alpha)           :       81.999861445439 electrons
N(Beta)            :       81.999861445439 electrons
N(Total)           :      163.999722890879 electrons
E(X)               :     -152.151287983879 Eh
E(C)               :       -7.082407878576 Eh
E(XC)              :     -159.233695862455 Eh

CPCM Solvation Model Properties:
Surface-charge          :         -0.05798445459651
Corrected charge        :          0.00000000000000
Outlying charge corr.   :          0.00016878400683 Eh              0.00459 eV
Free-energy (cav+disp)  :         -0.00597154625304 Eh             -0.16249 eV

---------------
SCF CONVERGENCE
---------------

  Last Energy change         ...   -1.5189e-10  Tolerance :   1.0000e-09
  Last MAX-Density change    ...    8.5974e-06  Tolerance :   1.0000e-08
  Last RMS-Density change    ...    2.8642e-07  Tolerance :   1.0000e-09
  Last DIIS Error            ...    3.1100e-03  Tolerance :   1.0000e-08
  Last Orbital Gradient      ...    2.2048e-06  Tolerance :   2.0000e-06
  Last Orbital Rotation      ...    1.6358e-06  Tolerance :   2.0000e-06


----------------
ORBITAL ENERGIES
----------------

  NO   OCC          E(Eh)            E(eV)
   0   2.0000    -453.925332    -12351.9362
   1   2.0000     -58.105639     -1581.1348
   2   2.0000     -52.158080     -1419.2935
   3   2.0000     -52.157299     -1419.2723
   4   2.0000     -52.156291     -1419.2448
   5   2.0000     -19.156557      -521.2764
   6   2.0000     -19.107493      -519.9413
   7   2.0000     -10.234535      -278.4959
   8   2.0000     -10.212595      -277.8988
   9   2.0000     -10.189105      -277.2597
  10   2.0000     -10.183041      -277.0946
  11   2.0000     -10.178947      -276.9832
  12   2.0000     -10.177625      -276.9473
  13   2.0000     -10.172605      -276.8107
  14   2.0000     -10.170953      -276.7657
  15   2.0000     -10.170116      -276.7429
  16   2.0000     -10.165693      -276.6226
  17   2.0000     -10.164857      -276.5998
  18   2.0000     -10.164158      -276.5808
  19   2.0000     -10.163860      -276.5727
  20   2.0000     -10.163395      -276.5600
  21   2.0000     -10.162464      -276.5347
  22   2.0000     -10.152527      -276.2643
  23   2.0000      -7.843971      -213.4453
  24   2.0000      -5.787921      -157.4973
  25   2.0000      -5.784005      -157.3908
  26   2.0000      -5.780365      -157.2917
  27   2.0000      -2.141406       -58.2706
  28   2.0000      -2.140609       -58.2489
  29   2.0000      -2.136996       -58.1506
```

30	2.0000	-2.133615	-58.0586
31	2.0000	-2.133282	-58.0495
32	2.0000	-1.066861	-29.0308
33	2.0000	-0.959275	-26.1032
34	2.0000	-0.859290	-23.3825
35	2.0000	-0.853843	-23.2343
36	2.0000	-0.792076	-21.5535
37	2.0000	-0.763677	-20.7807
38	2.0000	-0.753101	-20.4929
39	2.0000	-0.742077	-20.1929
40	2.0000	-0.739044	-20.1104
41	2.0000	-0.702722	-19.1220
42	2.0000	-0.675596	-18.3839
43	2.0000	-0.647742	-17.6259
44	2.0000	-0.613811	-16.7027
45	2.0000	-0.599488	-16.3129
46	2.0000	-0.590768	-16.0756
47	2.0000	-0.577295	-15.7090
48	2.0000	-0.552118	-15.0239
49	2.0000	-0.532806	-14.4984
50	2.0000	-0.502676	-13.6785
51	2.0000	-0.487188	-13.2571
52	2.0000	-0.467044	-12.7089
53	2.0000	-0.465904	-12.6779
54	2.0000	-0.462705	-12.5909
55	2.0000	-0.456891	-12.4326
56	2.0000	-0.448244	-12.1973
57	2.0000	-0.442466	-12.0401
58	2.0000	-0.437684	-11.9100
59	2.0000	-0.426291	-11.6000
60	2.0000	-0.415287	-11.3005
61	2.0000	-0.413051	-11.2397
62	2.0000	-0.410236	-11.1631
63	2.0000	-0.390822	-10.6348
64	2.0000	-0.387784	-10.5521
65	2.0000	-0.376205	-10.2370
66	2.0000	-0.368143	-10.0177
67	2.0000	-0.364968	-9.9313
68	2.0000	-0.363224	-9.8838
69	2.0000	-0.353755	-9.6262
70	2.0000	-0.346942	-9.4408
71	2.0000	-0.331949	-9.0328
72	2.0000	-0.328675	-8.9437
73	2.0000	-0.324930	-8.8418
74	2.0000	-0.323729	-8.8091
75	2.0000	-0.268878	-7.3165
76	2.0000	-0.263352	-7.1662
77	2.0000	-0.253571	-6.9000
78	2.0000	-0.250202	-6.8083
79	2.0000	-0.242097	-6.5878
80	2.0000	-0.237516	-6.4631
81	2.0000	-0.214672	-5.8415
82	0.0000	-0.035785	-0.9738
83	0.0000	-0.021986	-0.5983
84	0.0000	-0.014647	-0.3986
85	0.0000	-0.005871	-0.1598
86	0.0000	-0.001113	-0.0303
87	0.0000	0.022769	0.6196
88	0.0000	0.036641	0.9971
89	0.0000	0.046388	1.2623
90	0.0000	0.059449	1.6177
91	0.0000	0.067172	1.8278
92	0.0000	0.069589	1.8936

*Only the first 10 virtual orbitals were printed (use %output print[p_orben] 2 end to see all).

```
                    ********************************
                    * MULLIKEN POPULATION ANALYSIS *
                    ********************************

-----------------------
MULLIKEN ATOMIC CHARGES
-----------------------
   0 C :    0.185422
   1 C :   -0.239834
   2 C :   -0.241924
   3 C :   -0.100582
   4 C :   -0.126036
   5 C :   -0.168315
   6 H :    0.120742
   7 H :    0.133463
   8 H :    0.141111
   9 H :    0.136180
  10 H :    0.135405
  11 C :   -0.178959
  12 H :    0.110479
  13 H :    0.115186
  14 C :   -0.266602
  15 H :    0.256275
  16 H :    0.124958
  17 C :   -0.305958
  18 H :    0.161284
  19 H :    0.160527
  20 Se:    0.408217
  21 O :   -0.573134
  22 C :    0.038920
  23 C :   -0.202031
  24 C :   -0.168714
  25 C :   -0.285730
  26 C :   -0.234192
  27 C :    0.362267
  28 H :    0.139581
  29 H :    0.153525
  30 H :    0.152112
  31 H :    0.129701
  32 O :   -0.288816
  33 C :   -0.208034
  34 H :    0.137140
  35 H :    0.137784
  36 H :    0.148584
Sum of atomic charges:   -0.0000000

--------------------------------
MULLIKEN REDUCED ORBITAL CHARGES
--------------------------------
  0 C s       :     3.152368  s :     3.152368
      pz      :     0.886612  p :     2.527822
      px      :     0.780410
      py      :     0.860800
      dz2     :     0.023671  d :     0.124557
      dxz     :     0.028712
      dyz     :     0.018751
      dx2y2   :     0.025553
      dxy     :     0.027869
      f0      :     0.001043  f :     0.009832
      f+1     :     0.001324
      f-1     :     0.001277
```

```
    f+2     :     0.001934
    f-2     :     0.001347
    f+3     :     0.001205
    f-3     :     0.001702

1 C s       :     3.278017  s :     3.278017
    pz      :     0.978252  p :     2.872147
    px      :     0.967446
    py      :     0.926449
    dz2     :     0.016831  d :     0.082358
    dxz     :     0.020465
    dyz     :     0.010054
    dx2y2   :     0.012920
    dxy     :     0.022087
    f0      :     0.000989  f :     0.007312
    f+1     :     0.001060
    f-1     :     0.000716
    f+2     :     0.001360
    f-2     :     0.001223
    f+3     :     0.000901
    f-3     :     0.001064

2 C s       :     3.271099  s :     3.271099
    pz      :     0.970046  p :     2.881219
    px      :     0.944565
    py      :     0.966608
    dz2     :     0.011472  d :     0.082332
    dxz     :     0.019240
    dyz     :     0.011333
    dx2y2   :     0.018786
    dxy     :     0.021502
    f0      :     0.000334  f :     0.007273
    f+1     :     0.001234
    f-1     :     0.000981
    f+2     :     0.001125
    f-2     :     0.001158
    f+3     :     0.000982
    f-3     :     0.001459

3 C s       :     3.192817  s :     3.192817
    pz      :     0.945133  p :     2.818373
    px      :     0.917302
    py      :     0.955938
    dz2     :     0.011372  d :     0.082126
    dxz     :     0.019034
    dyz     :     0.010445
    dx2y2   :     0.020054
    dxy     :     0.021222
    f0      :     0.000326  f :     0.007266
    f+1     :     0.001246
    f-1     :     0.001037
    f+2     :     0.001154
    f-2     :     0.001151
    f+3     :     0.000925
    f-3     :     0.001427

4 C s       :     3.211645  s :     3.211645
    pz      :     0.948611  p :     2.824671
    px      :     0.922693
    py      :     0.953366
    dz2     :     0.017004  d :     0.082456
    dxz     :     0.020886
    dyz     :     0.009592
```

```
      dx2y2   :     0.013205
      dxy     :     0.021769
      f0      :     0.001030  f :     0.007264
      f+1     :     0.000991
      f-1     :     0.000721
      f+2     :     0.001316
      f-2     :     0.001237
      f+3     :     0.000882
      f-3     :     0.001087

  5 C s       :     3.233987  s :     3.233987
      pz      :     0.948521  p :     2.846288
      px      :     0.965423
      py      :     0.932344
      dz2     :     0.019212  d :     0.080809
      dxz     :     0.014285
      dyz     :     0.014738
      dx2y2   :     0.021187
      dxy     :     0.011388
      f0      :     0.000888  f :     0.007231
      f+1     :     0.000805
      f-1     :     0.001188
      f+2     :     0.001433
      f-2     :     0.001083
      f+3     :     0.000929
      f-3     :     0.000903

  6 H s       :     0.856963  s :     0.856963
      pz      :     0.006014  p :     0.022296
      px      :     0.007040
      py      :     0.009241

  7 H s       :     0.844369  s :     0.844369
      pz      :     0.009682  p :     0.022169
      px      :     0.005038
      py      :     0.007448

  8 H s       :     0.836472  s :     0.836472
      pz      :     0.009936  p :     0.022417
      px      :     0.005056
      py      :     0.007425

  9 H s       :     0.841649  s :     0.841649
      pz      :     0.006007  p :     0.022171
      px      :     0.007041
      py      :     0.009123

 10 H s       :     0.842331  s :     0.842331
      pz      :     0.005147  p :     0.022264
      px      :     0.012794
      py      :     0.004323

 11 C s       :     3.235916  s :     3.235916
      pz      :     0.970470  p :     2.850318
      px      :     0.904708
      py      :     0.975141
      dz2     :     0.018953  d :     0.086965
      dxz     :     0.024191
      dyz     :     0.010004
      dx2y2   :     0.016180
      dxy     :     0.017637
      f0      :     0.000594  f :     0.005760
      f+1     :     0.000833
```

```
      f-1     :     0.000803
      f+2     :     0.001309
      f-2     :     0.000823
      f+3     :     0.000734
      f-3     :     0.000665

 12 H s       :     0.867805  s :     0.867805
      pz      :     0.005083  p :     0.021716
      px      :     0.005657
      py      :     0.010976

 13 H s       :     0.863055  s :     0.863055
      pz      :     0.009496  p :     0.021759
      px      :     0.007378
      py      :     0.004885

 14 C s       :     3.201211  s :     3.201211
      pz      :     0.977563  p :     2.964020
      px      :     0.952966
      py      :     1.033491
      dz2     :     0.027552  d :     0.094974
      dxz     :     0.013942
      dyz     :     0.018910
      dx2y2   :     0.022927
      dxy     :     0.011642
      f0      :     0.001191  f :     0.006398
      f+1     :     0.000710
      f-1     :     0.000732
      f+2     :     0.001641
      f-2     :     0.001064
      f+3     :     0.000407
      f-3     :     0.000653

 15 H s       :     0.691055  s :     0.691055
      pz      :     0.008854  p :     0.052670
      px      :     0.007316
      py      :     0.036501

 16 H s       :     0.852398  s :     0.852398
      pz      :     0.005365  p :     0.022644
      px      :     0.012050
      py      :     0.005230

 17 C s       :     3.302791  s :     3.302791
      pz      :     1.005574  p :     2.927960
      px      :     1.008349
      py      :     0.914037
      dz2     :     0.024573  d :     0.070287
      dxz     :     0.006004
      dyz     :     0.010829
      dx2y2   :     0.014337
      dxy     :     0.014544
      f0      :     0.000736  f :     0.004920
      f+1     :     0.001219
      f-1     :     0.000626
      f+2     :     0.000400
      f-2     :     0.000870
      f+3     :     0.000335
      f-3     :     0.000734

 18 H s       :     0.816362  s :     0.816362
      pz      :     0.012456  p :     0.022354
      px      :     0.004541
```

```
     py      :     0.005358

19 H s       :     0.816663  s :     0.816663
     pz      :     0.004048  p :     0.022810
     px      :     0.012241
     py      :     0.006521

20 Ses       :     7.838758  s :     7.838758
     pz      :     5.101438  p :    15.480996
     px      :     4.810593
     py      :     5.568966
     dz2     :     2.052368  d :    10.231600
     dxz     :     2.047104
     dyz     :     2.036641
     dx2y2   :     2.059971
     dxy     :     2.035517
     f0      :     0.006926  f :     0.040429
     f+1     :     0.008979
     f-1     :     0.002274
     f+2     :     0.007624
     f-2     :     0.007510
     f+3     :     0.003415
     f-3     :     0.003702

21 O s       :     3.865453  s :     3.865453
     pz      :     1.552076  p :     4.680959
     px      :     1.528798
     py      :     1.600084
     dz2     :     0.005903  d :     0.025756
     dxz     :     0.006928
     dyz     :     0.003318
     dx2y2   :     0.005599
     dxy     :     0.004008
     f0      :     0.000107  f :     0.000966
     f+1     :     0.000243
     f-1     :     0.000068
     f+2     :     0.000200
     f-2     :     0.000154
     f+3     :     0.000104
     f-3     :     0.000089

22 C s       :     3.166616  s :     3.166616
     pz      :     0.837677  p :     2.662400
     px      :     0.895512
     py      :     0.929212
     dz2     :     0.023453  d :     0.123803
     dxz     :     0.035478
     dyz     :     0.023271
     dx2y2   :     0.023567
     dxy     :     0.018035
     f0      :     0.001311  f :     0.008260
     f+1     :     0.001412
     f-1     :     0.000891
     f+2     :     0.001332
     f-2     :     0.001309
     f+3     :     0.001160
     f-3     :     0.000845

23 C s       :     3.281211  s :     3.281211
     pz      :     0.926999  p :     2.821255
     px      :     0.928155
     py      :     0.966101
     dz2     :     0.024243  d :     0.091919
```

```
      dxz     :     0.024983
      dyz     :     0.014698
      dx2y2   :     0.008084
      dxy     :     0.019911
      f0      :     0.001346  f :     0.007647
      f+1     :     0.001587
      f-1     :     0.000618
      f+2     :     0.000967
      f-2     :     0.001476
      f+3     :     0.000738
      f-3     :     0.000915

 24 C s       :     3.242446  s :     3.242446
      pz      :     0.949495  p :     2.829531
      px      :     0.917570
      py      :     0.962466
      dz2     :     0.020003  d :     0.089257
      dxz     :     0.017032
      dyz     :     0.010226
      dx2y2   :     0.020732
      dxy     :     0.021265
      f0      :     0.000445  f :     0.007480
      f+1     :     0.001593
      f-1     :     0.000913
      f+2     :     0.001004
      f-2     :     0.001231
      f+3     :     0.001205
      f-3     :     0.001088

 25 C s       :     3.316862  s :     3.316862
      pz      :     0.970422  p :     2.890033
      px      :     0.906736
      py      :     1.012875
      dz2     :     0.013634  d :     0.071580
      dxz     :     0.017759
      dyz     :     0.009514
      dx2y2   :     0.014599
      dxy     :     0.016075
      f0      :     0.000511  f :     0.007255
      f+1     :     0.001457
      f-1     :     0.000862
      f+2     :     0.000960
      f-2     :     0.001187
      f+3     :     0.001238
      f-3     :     0.001040

 26 C s       :     3.306588  s :     3.306588
      pz      :     0.928494  p :     2.848455
      px      :     0.956570
      py      :     0.963391
      dz2     :     0.020202  d :     0.071707
      dxz     :     0.015118
      dyz     :     0.012870
      dx2y2   :     0.008505
      dxy     :     0.015012
      f0      :     0.001284  f :     0.007442
      f+1     :     0.001594
      f-1     :     0.000629
      f+2     :     0.000938
      f-2     :     0.001373
      f+3     :     0.000702
      f-3     :     0.000922
```

```
27 C s       :      2.993196  s :      2.993196
     pz      :      0.831731  p :      2.463763
     px      :      0.730258
     py      :      0.901775
     dz2     :      0.035763  d :      0.165068
     dxz     :      0.036688
     dyz     :      0.023726
     dx2y2   :      0.033577
     dxy     :      0.035314
     f0      :      0.001733  f :      0.015706
     f+1     :      0.003603
     f-1     :      0.001112
     f+2     :      0.002428
     f-2     :      0.002297
     f+3     :      0.002376
     f-3     :      0.002157

28 H s       :      0.837974  s :      0.837974
     pz      :      0.005581  p :      0.022446
     px      :      0.009220
     py      :      0.007644

29 H s       :      0.824168  s :      0.824168
     pz      :      0.012156  p :      0.022307
     px      :      0.003973
     py      :      0.006179

30 H s       :      0.824843  s :      0.824843
     pz      :      0.012369  p :      0.023045
     px      :      0.004152
     py      :      0.006523

31 H s       :      0.847920  s :      0.847920
     pz      :      0.005398  p :      0.022378
     px      :      0.009024
     py      :      0.007956

32 O s       :      3.758149  s :      3.758149
     pz      :      1.354373  p :      4.487547
     px      :      1.449628
     py      :      1.683547
     dz2     :      0.010179  d :      0.041029
     dxz     :      0.008815
     dyz     :      0.007794
     dx2y2   :      0.007737
     dxy     :      0.006504
     f0      :      0.000329  f :      0.002091
     f+1     :      0.000424
     f-1     :      0.000308
     f+2     :      0.000360
     f-2     :      0.000227
     f+3     :      0.000219
     f-3     :      0.000223

33 C s       :      3.271826  s :      3.271826
     pz      :      0.727576  p :      2.843714
     px      :      1.092701
     py      :      1.023437
     dz2     :      0.025321  d :      0.084651
     dxz     :      0.020855
     dyz     :      0.019158
     dx2y2   :      0.013755
     dxy     :      0.005562
```

```
     f0      :     0.002285  f :     0.007843
     f+1     :     0.001427
     f-1     :     0.001678
     f+2     :     0.000818
     f-2     :     0.000795
     f+3     :     0.000089
     f-3     :     0.000750

 34 H s       :     0.842623  s :     0.842623
      pz      :     0.007718  p :     0.020237
      px      :     0.007762
      py      :     0.004757

 35 H s       :     0.842023  s :     0.842023
      pz      :     0.002894  p :     0.020194
      px      :     0.004189
      py      :     0.013111

 36 H s       :     0.830607  s :     0.830607
      pz      :     0.003822  p :     0.020809
      px      :     0.011979
      py      :     0.005007


                     *******************************
                     * LOEWDIN POPULATION ANALYSIS *
                     *******************************

----------------------
LOEWDIN ATOMIC CHARGES
----------------------
   0 C :   -0.147859
   1 C :   -0.114892
   2 C :   -0.124116
   3 C :   -0.142982
   4 C :   -0.153040
   5 C :   -0.154053
   6 H :    0.150394
   7 H :    0.151157
   8 H :    0.147693
   9 H :    0.147619
  10 H :    0.146942
  11 C :   -0.211337
  12 H :    0.146947
  13 H :    0.146170
  14 C :   -0.314269
  15 H :    0.185191
  16 H :    0.143134
  17 C :   -0.233176
  18 H :    0.124586
  19 H :    0.127781
  20 Se:    0.641165
  21 O :   -0.410458
  22 C :   -0.343095
  23 C :   -0.139531
  24 C :   -0.142665
  25 C :   -0.136015
  26 C :   -0.118519
  27 C :   -0.255427
  28 H :    0.152163
  29 H :    0.154469
  30 H :    0.156825
```

```
  31 H :    0.153862
  32 O :    0.233945
  33 C :   -0.312029
  34 H :    0.112639
  35 H :    0.112856
  36 H :    0.117922

-------------------------------
LOEWDIN REDUCED ORBITAL CHARGES
-------------------------------
  0 C s       :     2.710936  s :     2.710936
      pz      :     0.944073  p :     2.859990
      px      :     0.969366
      py      :     0.946551
      dz2     :     0.093454  d :     0.519171
      dxz     :     0.113588
      dyz     :     0.081982
      dx2y2   :     0.120368
      dxy     :     0.109779
      f0      :     0.006063  f :     0.057762
      f+1     :     0.009026
      f-1     :     0.006565
      f+2     :     0.010443
      f-2     :     0.007595
      f+3     :     0.006914
      f-3     :     0.011157

  1 C s       :     2.759634  s :     2.759634
      pz      :     0.964249  p :     2.929247
      px      :     1.019960
      py      :     0.945038
      dz2     :     0.065233  d :     0.383232
      dxz     :     0.101270
      dyz     :     0.051514
      dx2y2   :     0.067420
      dxy     :     0.097795
      f0      :     0.005687  f :     0.042779
      f+1     :     0.006169
      f-1     :     0.004545
      f+2     :     0.008143
      f-2     :     0.005341
      f+3     :     0.005564
      f-3     :     0.007329

  2 C s       :     2.760317  s :     2.760317
      pz      :     0.945643  p :     2.936945
      px      :     1.032552
      py      :     0.958751
      dz2     :     0.048987  d :     0.384240
      dxz     :     0.087112
      dyz     :     0.057424
      dx2y2   :     0.090808
      dxy     :     0.099908
      f0      :     0.002846  f :     0.042614
      f+1     :     0.007670
      f-1     :     0.004196
      f+2     :     0.007111
      f-2     :     0.005388
      f+3     :     0.005527
      f-3     :     0.009876

  3 C s       :     2.765623  s :     2.765623
      pz      :     0.944970  p :     2.945053
```

```
      px      :     1.041428
      py      :     0.958655
      dz2     :     0.050594  d :     0.388825
      dxz     :     0.087989
      dyz     :     0.057415
      dx2y2   :     0.091812
      dxy     :     0.101016
      f0      :     0.002834  f :     0.043480
      f+1     :     0.007831
      f-1     :     0.004451
      f+2     :     0.007326
      f-2     :     0.005492
      f+3     :     0.005551
      f-3     :     0.009995

  4 C s       :     2.766411  s :     2.766411
      pz      :     0.977231  p :     2.954480
      px      :     1.026358
      py      :     0.950891
      dz2     :     0.068493  d :     0.388876
      dxz     :     0.103166
      dyz     :     0.052032
      dx2y2   :     0.065993
      dxy     :     0.099193
      f0      :     0.005966  f :     0.043273
      f+1     :     0.006077
      f-1     :     0.004700
      f+2     :     0.008199
      f-2     :     0.005394
      f+3     :     0.005576
      f-3     :     0.007359

  5 C s       :     2.766631  s :     2.766631
      pz      :     0.985511  p :     2.957078
      px      :     0.984163
      py      :     0.987404
      dz2     :     0.079019  d :     0.386893
      dxz     :     0.072060
      dyz     :     0.074668
      dx2y2   :     0.100740
      dxy     :     0.060406
      f0      :     0.005128  f :     0.043451
      f+1     :     0.005977
      f-1     :     0.006090
      f+2     :     0.007642
      f-2     :     0.006498
      f+3     :     0.005383
      f-3     :     0.006733

  6 H s       :     0.790228  s :     0.790228
      pz      :     0.016325  p :     0.059378
      px      :     0.018825
      py      :     0.024228

  7 H s       :     0.789682  s :     0.789682
      pz      :     0.025292  p :     0.059162
      px      :     0.014124
      py      :     0.019745

  8 H s       :     0.792997  s :     0.792997
      pz      :     0.025177  p :     0.059310
      px      :     0.014274
      py      :     0.019859
```

```
  9 H s       :     0.793322  s :     0.793322
      pz      :     0.016455  p :     0.059059
      px      :     0.018864
      py      :     0.023739

 10 H s       :     0.793725  s :     0.793725
      pz      :     0.014423  p :     0.059332
      px      :     0.032353
      py      :     0.012557

 11 C s       :     2.766176  s :     2.766176
      pz      :     1.010272  p :     3.016986
      px      :     0.994730
      py      :     1.011983
      dz2     :     0.072315  d :     0.389986
      dxz     :     0.098941
      dyz     :     0.062066
      dx2y2   :     0.082484
      dxy     :     0.074179
      f0      :     0.005189  f :     0.038189
      f+1     :     0.006841
      f-1     :     0.004437
      f+2     :     0.005360
      f-2     :     0.005396
      f+3     :     0.005677
      f-3     :     0.005289

 12 H s       :     0.794727  s :     0.794727
      pz      :     0.015156  p :     0.058326
      px      :     0.014431
      py      :     0.028739

 13 H s       :     0.795712  s :     0.795712
      pz      :     0.024850  p :     0.058119
      px      :     0.018550
      py      :     0.014719

 14 C s       :     2.787770  s :     2.787770
      pz      :     1.042840  p :     3.071284
      px      :     1.030939
      py      :     0.997505
      dz2     :     0.097480  d :     0.413357
      dxz     :     0.090254
      dyz     :     0.087367
      dx2y2   :     0.097997
      dxy     :     0.040258
      f0      :     0.008964  f :     0.041858
      f+1     :     0.006207
      f-1     :     0.004841
      f+2     :     0.007048
      f-2     :     0.006396
      f+3     :     0.004311
      f-3     :     0.004091

 15 H s       :     0.653764  s :     0.653764
      pz      :     0.022401  p :     0.161045
      px      :     0.018434
      py      :     0.120211

 16 H s       :     0.794929  s :     0.794929
      pz      :     0.016130  p :     0.061937
      px      :     0.030396
```

```
     py      :     0.015411

17 C s       :     2.871521  s :     2.871521
     pz      :     1.055513  p :     3.030090
     px      :     1.071771
     py      :     0.902805
     dz2     :     0.106324  d :     0.303034
     dxz     :     0.050714
     dyz     :     0.030880
     dx2y2   :     0.053040
     dxy     :     0.062077
     f0      :     0.003749  f :     0.028531
     f+1     :     0.006419
     f-1     :     0.003096
     f+2     :     0.004170
     f-2     :     0.005400
     f+3     :     0.002048
     f-3     :     0.003650

18 H s       :     0.810496  s :     0.810496
     pz      :     0.034524  p :     0.064918
     px      :     0.015084
     py      :     0.015309

19 H s       :     0.805704  s :     0.805704
     pz      :     0.012875  p :     0.066516
     px      :     0.035115
     py      :     0.018526

20 Ses       :     7.324825  s :     7.324825
     pz      :     5.125764  p :    15.379598
     px      :     4.809707
     py      :     5.444127
     dz2     :     2.111513  d :    10.547517
     dxz     :     2.147836
     dyz     :     2.077071
     dx2y2   :     2.136362
     dxy     :     2.074735
     f0      :     0.016229  f :     0.106895
     f+1     :     0.027457
     f-1     :     0.005165
     f+2     :     0.023387
     f-2     :     0.017584
     f+3     :     0.008167
     f-3     :     0.008906

21 O s       :     3.626291  s :     3.626291
     pz      :     1.613762  p :     4.720549
     px      :     1.557085
     py      :     1.549702
     dz2     :     0.012706  d :     0.060814
     dxz     :     0.019515
     dyz     :     0.006193
     dx2y2   :     0.011055
     dxy     :     0.011346
     f0      :     0.000229  f :     0.002804
     f+1     :     0.000714
     f-1     :     0.000275
     f+2     :     0.000547
     f-2     :     0.000417
     f+3     :     0.000305
     f-3     :     0.000317
```

```
22 C s       :     2.782273  s :     2.782273
     pz      :     1.040700  p :     3.024309
     px      :     1.003716
     py      :     0.979893
     dz2     :     0.095202  d :     0.487099
     dxz     :     0.142145
     dyz     :     0.086929
     dx2y2   :     0.086054
     dxy     :     0.076769
     f0      :     0.007074  f :     0.049413
     f+1     :     0.010215
     f-1     :     0.006990
     f+2     :     0.006408
     f-2     :     0.007477
     f+3     :     0.005311
     f-3     :     0.005938

23 C s       :     2.764411  s :     2.764411
     pz      :     1.012349  p :     2.929974
     px      :     1.001884
     py      :     0.915741
     dz2     :     0.096742  d :     0.400636
     dxz     :     0.120053
     dyz     :     0.068183
     dx2y2   :     0.039951
     dxy     :     0.075708
     f0      :     0.007022  f :     0.044511
     f+1     :     0.010733
     f-1     :     0.005435
     f+2     :     0.006249
     f-2     :     0.005930
     f+3     :     0.003864
     f-3     :     0.005277

24 C s       :     2.763333  s :     2.763333
     pz      :     0.971863  p :     2.935572
     px      :     1.038443
     py      :     0.925265
     dz2     :     0.084176  d :     0.399304
     dxz     :     0.087220
     dyz     :     0.050858
     dx2y2   :     0.090207
     dxy     :     0.086843
     f0      :     0.002759  f :     0.044457
     f+1     :     0.010775
     f-1     :     0.004291
     f+2     :     0.006900
     f-2     :     0.006911
     f+3     :     0.005202
     f-3     :     0.007618

25 C s       :     2.759428  s :     2.759428
     pz      :     0.981706  p :     2.967733
     px      :     1.026441
     py      :     0.959586
     dz2     :     0.078809  d :     0.366057
     dxz     :     0.085372
     dyz     :     0.047780
     dx2y2   :     0.075004
     dxy     :     0.079091
     f0      :     0.002903  f :     0.042796
     f+1     :     0.010119
     f-1     :     0.004011
```

```
    f+2     :     0.006422
    f-2     :     0.006781
    f+3     :     0.005238
    f-3     :     0.007323

 26 C s       :     2.761629  s :     2.761629
      pz      :     1.001599  p :     2.947509
      px      :     1.006752
      py      :     0.939158
      dz2     :     0.092961  d :     0.366502
      dxz     :     0.102165
      dyz     :     0.061099
      dx2y2   :     0.039922
      dxy     :     0.070356
      f0      :     0.006799  f :     0.042879
      f+1     :     0.010226
      f-1     :     0.005462
      f+2     :     0.006085
      f-2     :     0.005436
      f+3     :     0.003630
      f-3     :     0.005240

 27 C s       :     2.713092  s :     2.713092
      pz      :     0.977672  p :     2.765722
      px      :     0.864096
      py      :     0.923954
      dz2     :     0.143213  d :     0.679532
      dxz     :     0.162148
      dyz     :     0.101464
      dx2y2   :     0.140080
      dxy     :     0.132626
      f0      :     0.010132  f :     0.097081
      f+1     :     0.023986
      f-1     :     0.008282
      f+2     :     0.014078
      f-2     :     0.013018
      f+3     :     0.012286
      f-3     :     0.015299

 28 H s       :     0.787883  s :     0.787883
      pz      :     0.015507  p :     0.059954
      px      :     0.024140
      py      :     0.020306

 29 H s       :     0.785939  s :     0.785939
      pz      :     0.031167  p :     0.059592
      px      :     0.011404
      py      :     0.017021

 30 H s       :     0.780945  s :     0.780945
      pz      :     0.032184  p :     0.062230
      px      :     0.011992
      py      :     0.018054

 31 H s       :     0.785896  s :     0.785896
      pz      :     0.015241  p :     0.060242
      px      :     0.023530
      py      :     0.021470

 32 O s       :     3.244265  s :     3.244265
      pz      :     1.371530  p :     4.403954
      px      :     1.442913
      py      :     1.589511
```

```
     dz2     :     0.029167  d :     0.111237
     dxz     :     0.029979
     dyz     :     0.019289
     dx2y2   :     0.017967
     dxy     :     0.014835
     f0      :     0.001085  f :     0.006600
     f+1     :     0.001450
     f-1     :     0.000913
     f+2     :     0.000984
     f-2     :     0.000752
     f+3     :     0.000663
     f-3     :     0.000753

33 C s       :     2.861922  s :     2.861922
     pz      :     0.852126  p :     3.020341
     px      :     1.095660
     py      :     1.072555
     dz2     :     0.112265  d :     0.375510
     dxz     :     0.090525
     dyz     :     0.083260
     dx2y2   :     0.069480
     dxy     :     0.019980
     f0      :     0.014431  f :     0.054257
     f+1     :     0.008135
     f-1     :     0.014441
     f+2     :     0.004834
     f-2     :     0.008084
     f+3     :     0.001073
     f-3     :     0.003258

34 H s       :     0.825589  s :     0.825589
     pz      :     0.022186  p :     0.061772
     px      :     0.024551
     py      :     0.015035

35 H s       :     0.825421  s :     0.825421
     pz      :     0.008922  p :     0.061723
     px      :     0.013139
     py      :     0.039662

36 H s       :     0.821319  s :     0.821319
     pz      :     0.010686  p :     0.060759
     px      :     0.034539
     py      :     0.015534


                      *****************************
                      * MAYER POPULATION ANALYSIS *
                      *****************************

  NA   - Mulliken gross atomic population
  ZA   - Total nuclear charge
  QA   - Mulliken gross atomic charge
  VA   - Mayer's total valence
  BVA  - Mayer's bonded valence
  FA   - Mayer's free valence

  ATOM       NA         ZA         QA         VA         BVA        FA
  0 C      5.8146     6.0000     0.1854     3.5730     3.5730    -0.0000
  1 C      6.2398     6.0000    -0.2398     3.9269     3.9269     0.0000
  2 C      6.2419     6.0000    -0.2419     3.8824     3.8824    -0.0000
  3 C      6.1006     6.0000    -0.1006     3.8261     3.8261    -0.0000
```

```
  4 C      6.1260     6.0000    -0.1260     3.8301     3.8301    -0.0000
  5 C      6.1683     6.0000    -0.1683     3.8435     3.8435    -0.0000
  6 H      0.8793     1.0000     0.1207     0.9906     0.9906     0.0000
  7 H      0.8665     1.0000     0.1335     0.9703     0.9703    -0.0000
  8 H      0.8589     1.0000     0.1411     0.9673     0.9673    -0.0000
  9 H      0.8638     1.0000     0.1362     0.9650     0.9650     0.0000
 10 H      0.8646     1.0000     0.1354     0.9690     0.9690    -0.0000
 11 C      6.1790     6.0000    -0.1790     3.8440     3.8440    -0.0000
 12 H      0.8895     1.0000     0.1105     0.9616     0.9616     0.0000
 13 H      0.8848     1.0000     0.1152     0.9636     0.9636    -0.0000
 14 C      6.2666     6.0000    -0.2666     3.7526     3.7526    -0.0000
 15 H      0.7437     1.0000     0.2563     1.0165     1.0165    -0.0000
 16 H      0.8750     1.0000     0.1250     0.9630     0.9630    -0.0000
 17 C      6.3060     6.0000    -0.3060     3.8971     3.8971    -0.0000
 18 H      0.8387     1.0000     0.1613     0.9603     0.9603    -0.0000
 19 H      0.8395     1.0000     0.1605     0.9633     0.9633     0.0000
 20 Se    33.5918    34.0000     0.4082     2.9551     2.9551    -0.0000
 21 O      8.5731     8.0000    -0.5731     1.8147     1.8147    -0.0000
 22 C      5.9611     6.0000     0.0389     3.6629     3.6629    -0.0000
 23 C      6.2020     6.0000    -0.2020     3.8212     3.8212    -0.0000
 24 C      6.1687     6.0000    -0.1687     3.8318     3.8318    -0.0000
 25 C      6.2857     6.0000    -0.2857     3.7964     3.7964     0.0000
 26 C      6.2342     6.0000    -0.2342     3.8793     3.8793    -0.0000
 27 C      5.6377     6.0000     0.3623     3.7201     3.7201     0.0000
 28 H      0.8604     1.0000     0.1396     0.9936     0.9936    -0.0000
 29 H      0.8465     1.0000     0.1535     0.9627     0.9627    -0.0000
 30 H      0.8479     1.0000     0.1521     0.9842     0.9842     0.0000
 31 H      0.8703     1.0000     0.1297     0.9891     0.9891    -0.0000
 32 O      8.2888     8.0000    -0.2888     2.1047     2.1047    -0.0000
 33 C      6.2080     6.0000    -0.2080     3.8288     3.8288     0.0000
 34 H      0.8629     1.0000     0.1371     0.9574     0.9574     0.0000
 35 H      0.8622     1.0000     0.1378     0.9574     0.9574     0.0000
 36 H      0.8514     1.0000     0.1486     0.9626     0.9626    -0.0000

  Mayer bond orders larger than 0.100000
B(  0-C ,  1-C ) :   1.3437 B(  0-C ,  2-C ) :   1.3369 B(  0-C , 11-C ) :   0.9094
B(  1-C ,  3-C ) :   1.3984 B(  1-C ,  6-H ) :   0.9796 B(  2-C ,  4-C ) :   1.3896
B(  2-C ,  7-H ) :   0.9911 B(  3-C ,  5-C ) :   1.3717 B(  3-C ,  8-H ) :   0.9678
B(  4-C ,  5-C ) :   1.3738 B(  4-C ,  9-H ) :   0.9834 B(  5-C , 10-H ) :   0.9803
B( 11-C , 12-H ) :   0.9537 B( 11-C , 13-H ) :   0.9778 B( 11-C , 14-C ) :   0.9542
B( 14-C , 15-H ) :   0.5416 B( 14-C , 16-H ) :   0.9431 B( 14-C , 17-C ) :   1.2980
B( 15-H , 21-O ) :   0.4004 B( 17-C , 18-H ) :   0.9574 B( 17-C , 19-H ) :   0.9530
B( 17-C , 20-Se) :   0.5090 B( 20-Se, 21-O ) :   1.2570 B( 20-Se, 22-C ) :   0.9629
B( 22-C , 23-C ) :   1.3199 B( 22-C , 24-C ) :   1.3089 B( 23-C , 25-C ) :   1.3549
B( 23-C , 28-H ) :   0.9686 B( 24-C , 26-C ) :   1.4216 B( 24-C , 29-H ) :   0.9783
B( 25-C , 27-C ) :   1.2607 B( 25-C , 30-H ) :   0.9765 B( 26-C , 27-C ) :   1.3094
B( 26-C , 31-H ) :   0.9957 B( 27-C , 32-O ) :   1.1290 B( 32-O , 33-C ) :   0.9112
B( 33-C , 34-H ) :   0.9627 B( 33-C , 35-H ) :   0.9640 B( 33-C , 36-H ) :   0.9682


-------
TIMINGS
-------

Total SCF time: 0 days 0 hours 2 min 16 sec

Total time                  ....     136.035 sec
Sum of individual times     ....     131.781 sec  ( 96.9%)

SCF preparation             ....       0.740 sec  (  0.5%)
Fock matrix formation       ....     126.195 sec  ( 92.8%)
  Startup                   ....       0.056 sec  (  0.0% of F)
  Split-RI-J                ....      10.422 sec  (  8.3% of F)
```

```
  Chain of spheres X        ....      93.337 sec  ( 74.0% of F)
  XC integration            ....      12.898 sec  ( 10.2% of F)
    Basis function eval.    ....       3.258 sec  ( 25.3% of XC)
    Density eval.           ....       4.299 sec  ( 33.3% of XC)
    XC-Functional eval.     ....       0.117 sec  (  0.9% of XC)
    XC-Potential eval.      ....       4.364 sec  ( 33.8% of XC)
  CPCM terms                ....      12.912 sec  ( 10.2% of F)
Diagonalization             ....       0.000 sec  (  0.0%)
Density matrix formation    ....       0.475 sec  (  0.3%)
Total Energy calculation    ....       0.216 sec  (  0.2%)
Population analysis         ....       0.161 sec  (  0.1%)
Orbital Transformation      ....       0.436 sec  (  0.3%)
Orbital Orthonormalization  ....       0.000 sec  (  0.0%)
DIIS solution               ....       1.802 sec  (  1.3%)
SOSCF solution              ....       1.756 sec  (  1.3%)
Finished LeanSCF after 136.3 sec

Maximum memory used throughout the entire LEANSCF-calculation: 108.1 MB


-------------------------------------------------------------------------------
                          DFT DISPERSION CORRECTION

                              DFTD3 V3.1  Rev 1
                          USING Becke-Johnson damping
-------------------------------------------------------------------------------
The B3LYP functional is recognized
Active option DFTDOPT                   ...         4

molecular C6(AA) [au] = 16963.604940


            DFT-D V3
 parameters
 s6 scaling factor         :     1.0000
 a1 scaling factor         :     0.3981
 s8 scaling factor         :     1.9889
 a2 scaling factor         :     4.4211
 ad hoc parameters k1-k3   :    16.0000     1.3333    -4.0000

 Edisp/kcal,au: -55.865973339458  -0.089028085932
 E6   /kcal   : -26.012529699
 E8   /kcal   : -29.853443640

-------------------------   ----------------
Dispersion correction           -0.089028086
-------------------------   ----------------


-------------------------   --------------------
FINAL SINGLE POINT ENERGY     -3172.269476451928
-------------------------   --------------------


           ************************************************************
           *        Program running with 12 parallel MPI-processes    *
           *              working on a common directory               *
           ************************************************************
------------------------------------------------------------------------------
                         ORCA SCF GRADIENT CALCULATION
------------------------------------------------------------------------------
```

```
Nuc. rep. gradient        (SHARK) ... done (  0.0 sec)
HCore & Overlap gradient  (SHARK) ... done (  0.5 sec)
Split-RIJ-J gradient      (SHARK) ... done (  2.4 sec)
COSX-gradient                     ... done ( 42.1 sec)
XC gradient                       ... done (  6.6 sec)
CPCM gradient                     ... done (  2.4 sec)
SMD gradient                      ... done (  0.0 sec)
Dispersion correction             ... done (  0.0 sec)

-------------------
DISPERSION GRADIENT
-------------------

   1   C   :   -0.000224710    0.000890594    0.000545501
   2   C   :   -0.000166854    0.000923706    0.001090407
   3   C   :    0.000193540    0.000860708    0.000269843
   4   C   :   -0.000235930    0.000959791    0.000908998
   5   C   :    0.000193104    0.000764331    0.000169708
   6   C   :   -0.000007443    0.000921878    0.000458332
   7   H   :   -0.000198698    0.000056455    0.000570611
   8   H   :    0.000036455    0.000436475   -0.000146890
   9   H   :   -0.000095813    0.000210382    0.000508101
  10   H   :    0.000167459    0.000292496   -0.000095318
  11   H   :    0.000213966    0.000325508    0.000190986
  12   C   :   -0.000665354    0.000425576    0.000049113
  13   H   :   -0.000343744    0.000263723    0.000024430
  14   H   :   -0.000394657    0.000128007    0.000198800
  15   C   :   -0.000760844   -0.000356669   -0.000123592
  16   H   :   -0.000048688   -0.001017516   -0.000137365
  17   H   :   -0.000365220    0.000088427   -0.000142333
  18   C   :   -0.000133742   -0.001628326   -0.000586702
  19   H   :   -0.000090398    0.000118593   -0.000292373
  20   H   :   -0.000217919    0.000122179   -0.000632869
  21   Se  :   -0.001108628   -0.000257977   -0.001595417
  22   O   :   -0.000994356    0.000521553   -0.000270854
  23   C   :    0.000607287   -0.000992396   -0.000332447
  24   C   :    0.000347385   -0.000773392   -0.000199405
  25   C   :    0.000683534   -0.000742969   -0.000436606
  26   C   :    0.000258172   -0.000699963    0.000007942
  27   C   :    0.000696709   -0.000376556   -0.000554444
  28   C   :    0.000405353   -0.000534610   -0.000257135
  29   H   :   -0.000021769   -0.000390208    0.000243634
  30   H   :    0.000332453    0.000011045   -0.000382156
  31   H   :   -0.000007637   -0.000295035    0.000232339
  32   H   :    0.000311077    0.000067560   -0.000269969
  33   O   :    0.000539045   -0.000075018    0.000098835
  34   C   :    0.000422100   -0.000099576    0.000269863
  35   H   :    0.000236929   -0.000039169    0.000300793
  36   H   :    0.000228055   -0.000117583    0.000177695
  37   H   :    0.000209781    0.000007978    0.000139945

Difference to translation invariance:
           :    0.0000000000   -0.0000000000   -0.0000000000

Difference to rotation invariance:
           :    0.0000000000   -0.0000000000   -0.0000000000

Norm of the Dispersion gradient    ...    0.0052540597
RMS gradient                       ...    0.0004986933
MAX gradient                       ...    0.0016283257

------------------
CARTESIAN GRADIENT
```

```
------------------

   1   C   :   -0.000007978   -0.000001351    0.000015772
   2   C   :   -0.000002870   -0.000000222   -0.000000448
   3   C   :   -0.000003976    0.000003727    0.000002964
   4   C   :    0.000007120    0.000001106   -0.000004000
   5   C   :   -0.000002581   -0.000001089   -0.000003398
   6   C   :    0.000002142   -0.000001762   -0.000005632
   7   H   :    0.000001227    0.000001390    0.000002663
   8   H   :   -0.000000688   -0.000000259   -0.000002793
   9   H   :    0.000096360   -0.000045361    0.000021605
  10   H   :   -0.000001838    0.000000008   -0.000004181
  11   H   :   -0.000001318    0.000003681   -0.000002887
  12   C   :    0.000004136   -0.000002628   -0.000007667
  13   H   :    0.000007311   -0.000000438   -0.000006082
  14   H   :   -0.000006561    0.000004183   -0.000001978
  15   C   :   -0.000028823    0.000006129   -0.000018828
  16   H   :    0.000017273   -0.000008775    0.000001944
  17   H   :   -0.000003303   -0.000014463    0.000010303
  18   C   :   -0.000000763    0.000007506    0.000008502
  19   H   :    0.000003643   -0.000006842    0.000000138
  20   H   :   -0.000001627   -0.000002886    0.000000729
  21   Se  :    0.000001338   -0.000012785    0.000000232
  22   O   :    0.000005331    0.000000888    0.000006642
  23   C   :   -0.000005536    0.000010670    0.000007701
  24   C   :    0.000005358    0.000000941    0.000004289
  25   C   :    0.000000146   -0.000012089   -0.000004981
  26   C   :    0.000001846    0.000002865   -0.000002007
  27   C   :    0.000002843    0.000002445    0.000007036
  28   C   :    0.000002078   -0.000008831    0.000003203
  29   H   :    0.000001886    0.000000237    0.000004135
  30   H   :   -0.000002335   -0.000000852    0.000000621
  31   H   :    0.000005129   -0.000004809    0.000010363
  32   H   :   -0.000000324   -0.000000897   -0.000002415
  33   O   :    0.000000593    0.000027412   -0.000004518
  34   C   :    0.000000371   -0.000022832   -0.000017940
  35   H   :   -0.000098758    0.000069773   -0.000017452
  36   H   :   -0.000000289    0.000002981    0.000000184
  37   H   :    0.000003438    0.000003228   -0.000001818

Difference to translation invariance:
           :   -0.0000000001   -0.0000000004   -0.0000000010

Difference to rotation invariance:
           :   -0.0004158374    0.0002555727    0.0004128533

Norm of the Cartesian gradient     ...    0.0001797994
RMS gradient                       ...    0.0000170658
MAX gradient                       ...    0.0000987583

-------
TIMINGS
-------

Total SCF gradient time     ....       54.124 sec

Densities                   ....        0.008 sec  (  0.0%)
One electron gradient       ....        0.537 sec  (  1.0%)
RI-J Coulomb gradient       ....        2.411 sec  (  4.5%)
COSX gradient               ....       42.109 sec  ( 77.8%)
XC gradient                 ....        6.641 sec  ( 12.3%)
CPCM gradient               ....        2.364 sec  (  4.4%)
   A-Matrix (El+Nuc)        ....        0.025 sec  (  0.0%)
```

```
   Potential                ....       2.339 sec  (  4.3%)
SMD gradient                ....       0.003 sec  (  0.0%)

Maximum memory used throughout the entire SCFGRAD-calculation: 124.1 MB

           ************************************************************
           *        Program running with 12 parallel MPI-processes    *
           *              working on a common directory               *
           ************************************************************

-------------------------------------------------------------------------------
                              ORCA PROPERTY CALCULATIONS
-------------------------------------------------------------------------------

GBWName                                ... LQA_TS-OMe.gbw
Number of atoms                        ...     37
Number of basis functions              ...    714
Max core memory                        ...   8000 MB

Electric properties:
Dipole moment                          ... YES
Quadrupole moment                      ...  NO
General moments                        ...  NO (MaxL=10)
Static polarizability (Dipole/Dipole)  ...  NO
Static polarizability (Dipole/Quad.)   ...  NO
Static polarizability (Quad./Quad.)    ...  NO
Static polarizability (Velocity)       ...  NO
Static hyperpolarizability             ...  NO

Atomic electric properties:
Dipole moment                          ...  NO
Quadrupole moment                      ...  NO
Static polarizability                  ...  NO

Choice of electric origin              ... Center of mass
Position of electric origin            ...    -0.582945    -3.568287   -12.521182

General magnetic properties:
Magnetizability                        ...  NO

EPR properties:
g-Tensor (aka g-matrix)                ...  NO
Zero-Field splitting spin-orbit        ...  NO
Zero-field splitting spin-spin         ...  NO
Hyperfine couplings                    ...  NO (   0 nuclei)
Quadrupole couplings                   ...  NO (   0 nuclei)
Contact density                        ...  NO (   0 nuclei)

NMR properties:
Chemical shifts                        ...  NO (   0 nuclei)
Spin-rotation constants                ...  NO (   0 nuclei)
Spin-spin couplings                    ...  NO (   0 nuclei,    0 pairs)

Choice of magnetic origin              ... GIAO
Position of magnetic origin            ...     0.000000     0.000000     0.000000

Properties with geometric perturbations:
SCF Hessian                            ...  NO
IR spectrum                            ...  NO
Raman spectrum                         ...  NO
Raman option                           ... 0
VCD spectrum                           ...  NO
```

```
X-ray spectroscopy properties:
SCF XES/XAS/RIXS spectra                ...  NO

SCF SOC stabilization energy            ...  NO
Diagonal Born-Oppenheimer correction    ...  NO

-------------
DIPOLE MOMENT
-------------

Method             : SCF
Type of density    : Electron Density
Multiplicity       :   1
Irrep              :   0
Energy             : -3172.1804483659961988 Eh
Basis              : AO
                                X               Y               Z
Electronic contribution:    -16.198263124     -27.055073758     -24.433559484
Nuclear contribution   :     17.410341865      27.445708474      24.400811703
                        -----------------------------------------
Total Dipole Moment    :      1.212078741       0.390634715      -0.032747781
                        -----------------------------------------
Magnitude (a.u.)       :      1.273892763
Magnitude (Debye)      :      3.237978078



--------------------
Rotational spectrum
--------------------

Rotational constants in cm-1:            0.013681             0.008893             0.006312
Rotational constants in MHz :          410.153638           266.607310           189.236172

Dipole components along the rotational axes:
x,y,z [a.u.] :     1.062502    -0.167875    -0.682430
x,y,z [Debye]:     2.700665    -0.426705    -1.734600



Dipole moment calculation done in   0.0 sec

Maximum memory used throughout the entire PROP-calculation: 64.2 MB


--------------------------------------------------------------------------------
                   Intrinsic Reaction Coordinate Calculation
--------------------------------------------------------------------------------

IRC Settings:
  Nr. of atoms                        .... 37
  Algorithm                           .... LQA
  Coordinate system following type    .... Mass-Weighted
  Max. no of cycles (MaxIter)         .... 241
  Direction                           .... Forward and backward
  Steep Lenght                        .... 0.110 bohr Dalton^(1/2)

Initial displacement:
  Hessian type                        .... Read
  Hessian Filename                    .... TS-OMe_Compound_2.hess
  Following Hessian mode              .... 0
  Initial displacement type           .... Length
    Scaling normalized TS mode by     .... 0.040
```

```
Convergence Tolerances:
  Energy Change                      ....  5.0000e-06 Eh
  Max. Gradient                      ....  8.0000e-05 Eh/bohr
  RMS Gradient                       ....  1.0000e-05 Eh/bohr
  Max. Displacement                  ....  4.0000e-03 bohr
  RMS Displacement                   ....  2.0000e-03 bohr
  Strict Convergency criteria enforced

LQA Settings:
  Hessian update type                .... Bofill
  Update steep lenght                .... NO
  t Stepsize                         .... Gradient-based adjusted
  t MAX int. Steps                   .... 1000000
  Bulirsch-Stoer Correction          .... YES
    Euler Initial Steps              .... 100
    Richardson Max extrapolations    .... 20
    Richardson error threshould      ....  1.0000e-06
    Apply BS correction on TS disp.  .... NO
    Keep LQA direction               .... NO
    LQA direction Theshould          ....  1.0000e-01
    Update Hessian and Gradient      .... YES

Storing full IRC trajectory in       .... LQA_TS-OMe_IRC_Full_trj.xyz
Storing forward trajectory in        .... LQA_TS-OMe_IRC_F_trj.xyz
Storing backward trajectory in       .... LQA_TS-OMe_IRC_B_trj.xyz
Storing IRC profile in               .... LQA_TS-OMe.IRC.out


         *************************************************************
         *                         FORWARD IRC                       *
         *************************************************************

Iteration    E(Eh)      dE(kcal/mol)  max(|G|)  RMS(G)   max(|S|)  RMS(S)   MW-Displ.
Convergence thresholds    0.003138    0.000080  0.000010  0.004000  0.002000
    0     -3172.269520   -0.027450    0.002342  0.000484  0.035612  0.003797  0.046517
    1     -3172.269916   -0.275883    0.007553  0.001402  0.076319  0.008455  0.110003
    2     -3172.270525   -0.658260    0.011792  0.002046  0.063441  0.007632  0.110005
    3     -3172.271212   -1.088884    0.014613  0.002453  0.045508  0.006609  0.110004
    4     -3172.271930   -1.539583    0.016344  0.002712  0.031224  0.005837  0.110003
    5     -3172.272675   -2.007063    0.017837  0.002888  0.029518  0.005333  0.110002
    6     -3172.273438   -2.485716    0.019266  0.003018  0.028205  0.005020  0.110003
    7     -3172.274215   -2.973169    0.020448  0.003120  0.026990  0.004803  0.110002
    8     -3172.275003   -3.468231    0.021453  0.003203  0.025872  0.004647  0.110001
    9     -3172.275805   -3.971246    0.022324  0.003273  0.024830  0.004530  0.110001
   10     -3172.276613   -4.477928    0.023089  0.003333  0.023856  0.004436  0.110000
   11     -3172.277428   -4.989673    0.023767  0.003385  0.022945  0.004360  0.110003
   12     -3172.278250   -5.505355    0.024370  0.003431  0.022103  0.004297  0.110000
   13     -3172.279077   -6.024395    0.024905  0.003471  0.021333  0.004246  0.110003
   14     -3172.279907   -6.545423    0.025375  0.003505  0.020637  0.004203  0.110001
   15     -3172.280743   -7.069892    0.025785  0.003533  0.020014  0.004169  0.110000
   16     -3172.281581   -7.595477    0.026129  0.003556  0.019481  0.004143  0.110002
   17     -3172.282421   -8.122543    0.026407  0.003572  0.019014  0.004123  0.110001
   18     -3172.283261   -8.650125    0.026619  0.003582  0.018622  0.004109  0.110002
   19     -3172.284102   -9.177559    0.026760  0.003585  0.018305  0.004101  0.110002
   20     -3172.284941   -9.704168    0.026829  0.003581  0.018060  0.004097  0.110004
   21     -3172.285778   -10.229219    0.026825  0.003570  0.017893  0.004097  0.110001
   22     -3172.286610   -10.751701    0.026747  0.003550  0.017759  0.004100  0.110002
   23     -3172.287438   -11.270838    0.026591  0.003521  0.017555  0.004102  0.110000
   24     -3172.288244   -11.776484    0.026353  0.003483  0.017321  0.004103  0.110001
   25     -3172.289048   -12.281275    0.026042  0.003436  0.016985  0.004100  0.110001
   26     -3172.289837   -12.776349    0.025657  0.003381  0.016627  0.004091  0.110002
   27     -3172.290605   -13.258248    0.025197  0.003319  0.016219  0.004066  0.110004
```

28	-3172.291349	-13.725015	0.024650	0.003248	0.015952	0.004024	0.110002
29	-3172.292073	-14.179629	0.024014	0.003166	0.015721	0.004000	0.110003
30	-3172.292778	-14.621653	0.023300	0.003075	0.015530	0.003997	0.110000
31	-3172.293459	-15.049011	0.022512	0.002976	0.015294	0.003987	0.110002
32	-3172.294113	-15.459845	0.021649	0.002869	0.014989	0.003965	0.110003
33	-3172.294741	-15.853747	0.020714	0.002754	0.014636	0.003940	0.110003
34	-3172.295340	-16.229863	0.019709	0.002633	0.014536	0.003915	0.110001
35	-3172.295910	-16.587208	0.018638	0.002505	0.014689	0.003887	0.110000
36	-3172.296448	-16.925115	0.017510	0.002371	0.014876	0.003856	0.110000
37	-3172.296956	-17.243356	0.016329	0.002231	0.015074	0.003823	0.110002
38	-3172.297430	-17.541369	0.015104	0.002088	0.015304	0.003790	0.110000
39	-3172.297874	-17.819385	0.013845	0.001943	0.015560	0.003756	0.110002
40	-3172.298285	-18.077364	0.012563	0.001797	0.015840	0.003723	0.110001
41	-3172.298664	-18.315574	0.011276	0.001651	0.016119	0.003694	0.110000
42	-3172.299014	-18.534798	0.009998	0.001507	0.016359	0.003675	0.110000
43	-3172.299333	-18.735511	0.008750	0.001369	0.016584	0.003666	0.110001
44	-3172.299625	-18.918287	0.007548	0.001228	0.016706	0.003676	0.110002
45	-3172.299889	-19.084003	0.006885	0.001110	0.016583	0.003719	0.110000
46	-3172.300135	-19.238446	0.006349	0.000982	0.016621	0.003774	0.110000
47	-3172.300346	-19.370954	0.005821	0.000946	0.016589	0.003849	0.110000
48	-3172.300570	-19.511349	0.005171	0.000744	0.016004	0.004070	0.110002
49	-3172.300731	-19.612245	0.005042	0.001052	0.023603	0.005175	0.110002
50	-3172.300948	-19.748362	0.004342	0.000617	0.016334	0.004240	0.110003
51	-3172.301124	-19.859111	0.003901	0.000618	0.021143	0.004892	0.110003
52	-3172.301296	-19.966749	0.003543	0.000568	0.020805	0.004889	0.110001
53	-3172.301463	-20.071945	0.003239	0.000526	0.021640	0.005149	0.110001
54	-3172.301627	-20.174425	0.002981	0.000502	0.023310	0.005444	0.110000
55	-3172.301790	-20.276826	0.002769	0.000488	0.024365	0.005679	0.110001
56	-3172.301951	-20.378092	0.002594	0.000477	0.024638	0.005819	0.110001
57	-3172.302114	-20.480290	0.002451	0.000465	0.024591	0.005920	0.110001
58	-3172.302277	-20.582891	0.002332	0.000453	0.025143	0.006005	0.110001
59	-3172.302442	-20.686139	0.002233	0.000441	0.025476	0.006072	0.110000
60	-3172.302604	-20.787523	0.002148	0.000434	0.025536	0.006102	0.110001
61	-3172.302760	-20.885541	0.002073	0.000454	0.025186	0.006062	0.110001
62	-3172.302909	-20.979413	0.002007	0.000489	0.024255	0.005929	0.110001
63	-3172.303059	-21.073287	0.001948	0.000483	0.023488	0.005809	0.110001
64	-3172.303207	-21.166351	0.001904	0.000474	0.023808	0.005784	0.110000
65	-3172.303353	-21.257830	0.001863	0.000468	0.023618	0.005783	0.110001
66	-3172.303497	-21.348073	0.001825	0.000468	0.023385	0.005775	0.110000
67	-3172.303638	-21.436713	0.001868	0.000469	0.023156	0.005752	0.110001
68	-3172.303776	-21.522957	0.001918	0.000470	0.022942	0.005712	0.110001
69	-3172.303910	-21.607203	0.001957	0.000468	0.022749	0.005665	0.110001
70	-3172.304041	-21.689396	0.001988	0.000466	0.022565	0.005620	0.110001
71	-3172.304168	-21.769523	0.002016	0.000465	0.022377	0.005582	0.110001
72	-3172.304293	-21.847549	0.002045	0.000463	0.022190	0.005549	0.110001
73	-3172.304414	-21.923440	0.002074	0.000461	0.022011	0.005518	0.110000
74	-3172.304532	-21.997394	0.002098	0.000459	0.021860	0.005489	0.110001
75	-3172.304646	-22.069206	0.002125	0.000457	0.021688	0.005456	0.110000
76	-3172.304757	-22.139030	0.002151	0.000456	0.021584	0.005422	0.110001
77	-3172.304865	-22.206891	0.002173	0.000454	0.021649	0.005389	0.110001
78	-3172.304971	-22.272873	0.002193	0.000453	0.021713	0.005356	0.110000
79	-3172.305073	-22.336936	0.002212	0.000451	0.021753	0.005322	0.110000
80	-3172.305172	-22.399168	0.002226	0.000450	0.021768	0.005285	0.110000
81	-3172.305270	-22.460633	0.002136	0.000432	0.022000	0.005296	0.110000
82	-3172.305361	-22.518131	0.002291	0.000453	0.021894	0.005200	0.110000
83	-3172.305451	-22.574296	0.002221	0.000439	0.021401	0.005143	0.110000
84	-3172.305540	-22.629862	0.002255	0.000444	0.021564	0.005112	0.110001
85	-3172.305622	-22.681619	0.002195	0.000438	0.021230	0.005043	0.110001
86	-3172.305706	-22.734156	0.002068	0.000432	0.021167	0.005001	0.110001
87	-3172.305785	-22.783925	0.001767	0.000425	0.020812	0.004926	0.110000
88	-3172.305857	-22.828921	0.001701	0.000405	0.020572	0.004825	0.110000
89	-3172.305930	-22.874698	0.001892	0.000410	0.020064	0.004701	0.110000
90	-3172.305998	-22.917544	0.001860	0.000420	0.019239	0.004530	0.110001

91	-3172.306061	-22.956985	0.002035	0.000455	0.018421	0.004431	0.110000
92	-3172.306121	-22.995019	0.002480	0.000470	0.017533	0.004246	0.110000
93	-3172.306177	-23.029916	0.003041	0.000519	0.016963	0.004222	0.110000
94	-3172.306239	-23.068804	0.002381	0.000429	0.016087	0.004107	0.110000
95	-3172.306294	-23.103430	0.001465	0.000396	0.016973	0.004556	0.110001
96	-3172.306352	-23.139559	0.001126	0.000297	0.023486	0.005278	0.110001
97	-3172.306391	-23.164018	0.001971	0.000418	0.020456	0.005145	0.110000
98	-3172.306442	-23.196127	0.001305	0.000281	0.018352	0.004985	0.110000
99	-3172.306475	-23.216693	0.002004	0.000419	0.020148	0.005226	0.110000
100	-3172.306535	-23.254604	0.000950	0.000210	0.016635	0.004161	0.110001
101	-3172.306573	-23.278136	0.001508	0.000352	0.018199	0.004604	0.110000
102	-3172.306619	-23.307042	0.000983	0.000274	0.014795	0.004075	0.110001
103	-3172.306642	-23.321666	0.002956	0.000516	0.016882	0.004332	0.110000
104	-3172.306652	-23.328253	0.002741	0.000478	0.013302	0.002202	0.110000
105	-3172.306698	-23.357007	0.001055	0.000223	0.016040	0.004147	0.110002
106	-3172.306733	-23.378823	0.001353	0.000283	0.015086	0.004201	0.110000
107	-3172.306770	-23.402101	0.001396	0.000258	0.014797	0.003904	0.110000
108	-3172.306804	-23.423514	0.001449	0.000261	0.015274	0.003985	0.110000
109	-3172.306828	-23.438332	0.001486	0.000277	0.014354	0.003891	0.110000
110	-3172.306868	-23.463653	0.001341	0.000267	0.013861	0.003842	0.110000
111	-3172.306902	-23.484635	0.001242	0.000273	0.014453	0.003954	0.110000
112	-3172.306930	-23.502376	0.001317	0.000280	0.014114	0.003910	0.110000
113	-3172.306961	-23.521911	0.001426	0.000273	0.013835	0.003846	0.110000
114	-3172.306989	-23.539670	0.001539	0.000272	0.013668	0.003825	0.110000
115	-3172.307018	-23.557920	0.001568	0.000268	0.013103	0.003766	0.110000
116	-3172.307047	-23.575739	0.001553	0.000266	0.013012	0.003764	0.110000
117	-3172.307075	-23.593264	0.001539	0.000264	0.012872	0.003757	0.110000
118	-3172.307102	-23.610245	0.001547	0.000266	0.012964	0.003764	0.110000
119	-3172.307129	-23.626983	0.001539	0.000266	0.012883	0.003746	0.110000
120	-3172.307155	-23.643362	0.001518	0.000270	0.013150	0.003761	0.110000
121	-3172.307182	-23.660528	0.001496	0.000272	0.013246	0.003758	0.110000
122	-3172.307208	-23.676756	0.001506	0.000274	0.013388	0.003764	0.110000
123	-3172.307232	-23.691960	0.001540	0.000272	0.013314	0.003747	0.110000
124	-3172.307258	-23.707921	0.001585	0.000271	0.013379	0.003752	0.110000
125	-3172.307283	-23.723662	0.001608	0.000271	0.013270	0.003750	0.110000
126	-3172.307307	-23.739106	0.001600	0.000271	0.013106	0.003739	0.110000
127	-3172.307332	-23.754446	0.001584	0.000269	0.012948	0.003729	0.110000
128	-3172.307356	-23.769513	0.001604	0.000272	0.013093	0.003754	0.110000
129	-3172.307380	-23.784473	0.001599	0.000274	0.013086	0.003750	0.110000
130	-3172.307403	-23.799329	0.001596	0.000275	0.013124	0.003751	0.110000
131	-3172.307428	-23.814997	0.001602	0.000275	0.013178	0.003749	0.110000
132	-3172.307450	-23.828885	0.001598	0.000274	0.013091	0.003738	0.110000
133	-3172.307467	-23.839212	0.001628	0.000275	0.013176	0.003751	0.110000
134	-3172.307498	-23.858850	0.001637	0.000272	0.013231	0.003763	0.110000
135	-3172.307521	-23.872971	0.001671	0.000283	0.013336	0.003802	0.110000
136	-3172.307529	-23.878511	0.001287	0.000254	0.015026	0.002318	0.110000
137	-3172.307551	-23.892143	0.000998	0.000186	0.015767	0.004146	0.110002
138	-3172.307578	-23.908893	0.000590	0.000124	0.016775	0.004635	0.110000
139	-3172.307600	-23.923145	0.000717	0.000163	0.011154	0.004320	0.110000
140	-3172.307625	-23.938456	0.000857	0.000178	0.014621	0.004277	0.110000
141	-3172.307649	-23.953514	0.000902	0.000189	0.012157	0.004010	0.110000
142	-3172.307673	-23.968722	0.000857	0.000184	0.012318	0.004004	0.110000
143	-3172.307698	-23.984053	0.000825	0.000181	0.012632	0.004069	0.110000
144	-3172.307721	-23.998661	0.000818	0.000190	0.013192	0.004123	0.110000
145	-3172.307746	-24.014430	0.000839	0.000203	0.013432	0.004141	0.110000
146	-3172.307771	-24.029934	0.001086	0.000219	0.013772	0.004166	0.110000
147	-3172.307794	-24.044800	0.001342	0.000237	0.013963	0.004179	0.110000
148	-3172.307823	-24.062645	0.001594	0.000254	0.013981	0.004183	0.110000
149	-3172.307843	-24.075050	0.001470	0.000225	0.014931	0.004108	0.110000
150	-3172.307866	-24.089929	0.001607	0.000232	0.014145	0.004319	0.110000
151	-3172.307891	-24.105358	0.001927	0.000280	0.014236	0.004290	0.110000
152	-3172.307908	-24.116225	0.001937	0.000296	0.013867	0.004131	0.110000
153	-3172.307937	-24.134587	0.001805	0.000291	0.013999	0.004066	0.110000

154	-3172.307959	-24.148155	0.001608	0.000281	0.013754	0.004109	0.110000
155	-3172.307983	-24.163473	0.001441	0.000274	0.014442	0.004143	0.110000
156	-3172.308011	-24.180691	0.001230	0.000260	0.014616	0.004160	0.110000
157	-3172.308034	-24.194997	0.000942	0.000240	0.014253	0.004159	0.110000
158	-3172.308061	-24.212012	0.000944	0.000222	0.014166	0.004165	0.110000
159	-3172.308085	-24.226917	0.000982	0.000215	0.013873	0.004165	0.110000
160	-3172.308108	-24.241381	0.000948	0.000219	0.013699	0.004171	0.110000
161	-3172.308132	-24.256476	0.000877	0.000226	0.013499	0.004132	0.110000
162	-3172.308150	-24.267753	0.001098	0.000234	0.012902	0.004109	0.110000
163	-3172.308179	-24.286279	0.001206	0.000237	0.012713	0.004084	0.110000
164	-3172.308196	-24.296699	0.001363	0.000252	0.012620	0.004120	0.110000
165	-3172.308228	-24.317134	0.001517	0.000266	0.011967	0.004057	0.110000
166	-3172.308251	-24.331458	0.000758	0.000159	0.012870	0.003895	0.110000
167	-3172.308276	-24.347264	0.000645	0.000149	0.013969	0.004529	0.110000
168	-3172.308297	-24.360256	0.001057	0.000234	0.012000	0.004206	0.110000
169	-3172.308321	-24.375402	0.000961	0.000223	0.011861	0.003952	0.110000
170	-3172.308345	-24.390213	0.000843	0.000206	0.012618	0.004095	0.110000
171	-3172.308368	-24.404723	0.000880	0.000204	0.013338	0.004172	0.110000
172	-3172.308391	-24.419146	0.000960	0.000205	0.013183	0.004116	0.110000
173	-3172.308414	-24.433522	0.000957	0.000204	0.012566	0.004057	0.110000
174	-3172.308436	-24.447678	0.000910	0.000211	0.012085	0.004058	0.110000
175	-3172.308459	-24.461774	0.000836	0.000221	0.011696	0.004064	0.110000
176	-3172.308481	-24.475854	0.000940	0.000228	0.011608	0.004075	0.110000
177	-3172.308503	-24.489780	0.000958	0.000228	0.012044	0.004090	0.110000
178	-3172.308526	-24.503991	0.000823	0.000219	0.011979	0.004094	0.110000
179	-3172.308549	-24.518220	0.000833	0.000210	0.011811	0.004089	0.110000
180	-3172.308572	-24.532506	0.000924	0.000207	0.011363	0.004058	0.110000
181	-3172.308594	-24.546529	0.000994	0.000210	0.011791	0.004051	0.110000
182	-3172.308616	-24.560264	0.001008	0.000212	0.012377	0.004051	0.110000
183	-3172.308638	-24.573916	0.000992	0.000210	0.012788	0.004076	0.110000
184	-3172.308659	-24.587482	0.000969	0.000207	0.012708	0.004084	0.110000
185	-3172.308681	-24.600900	0.000940	0.000206	0.012139	0.004073	0.110000
186	-3172.308702	-24.614215	0.000910	0.000209	0.011489	0.004058	0.110000
187	-3172.308723	-24.627389	0.000892	0.000210	0.011098	0.004049	0.110000
188	-3172.308744	-24.640476	0.000894	0.000210	0.010931	0.004043	0.110000
189	-3172.308764	-24.653513	0.000914	0.000209	0.010901	0.004037	0.110000
190	-3172.308785	-24.666489	0.000939	0.000208	0.010962	0.004029	0.110000
191	-3172.308806	-24.679407	0.000955	0.000209	0.011149	0.004024	0.110000
192	-3172.308826	-24.692309	0.000956	0.000209	0.011371	0.004024	0.110000
193	-3172.308847	-24.705247	0.000940	0.000208	0.011494	0.004026	0.110000
194	-3172.308868	-24.718340	0.000909	0.000207	0.011444	0.004026	0.110000
195	-3172.308884	-24.728426	0.000869	0.000205	0.011334	0.004021	0.110000
196	-3172.308911	-24.745773	0.000831	0.000207	0.010124	0.004048	0.110000
197	-3172.308923	-24.753338	0.000836	0.000195	0.011900	0.003889	0.110000
198	-3172.308946	-24.767228	0.000870	0.000204	0.010023	0.004111	0.110000
199	-3172.308966	-24.779858	0.000905	0.000217	0.011063	0.004029	0.110000
200	-3172.308982	-24.790090	0.000844	0.000196	0.010234	0.003856	0.110000
201	-3172.309003	-24.803530	0.000864	0.000211	0.011179	0.004125	0.110000
202	-3172.309022	-24.814944	0.000889	0.000207	0.010772	0.003934	0.110000
203	-3172.309037	-24.824595	0.000918	0.000213	0.010408	0.004026	0.110000
204	-3172.309061	-24.839560	0.000931	0.000214	0.009899	0.003987	0.110000
205	-3172.309083	-24.853545	0.000964	0.000210	0.010623	0.003924	0.110000
206	-3172.309098	-24.863134	0.000918	0.000185	0.010264	0.003799	0.110000
207	-3172.309102	-24.865655	0.000677	0.000159	0.008161	0.001642	0.110000
208	-3172.309119	-24.876012	0.000739	0.000148	0.014573	0.004005	0.110000
209	-3172.309141	-24.890018	0.000878	0.000176	0.013492	0.004575	0.110000
210	-3172.309157	-24.900062	0.001024	0.000248	0.015380	0.004619	0.110000
211	-3172.309176	-24.911742	0.000557	0.000135	0.007613	0.003419	0.110000
212	-3172.309194	-24.923315	0.000792	0.000176	0.013598	0.004772	0.110000
213	-3172.309210	-24.933344	0.000695	0.000157	0.015665	0.004109	0.110000
214	-3172.309229	-24.944852	0.000419	0.000079	0.010178	0.004134	0.110000
215	-3172.309247	-24.956385	0.000810	0.000192	0.012082	0.004702	0.110000
216	-3172.309267	-24.968634	0.000742	0.000181	0.014333	0.004327	0.110000

```
 217     -3172.309287   -24.981480    0.000602  0.000152  0.013340  0.004397  0.110000
 218     -3172.309307   -24.993997    0.000597  0.000140  0.012217  0.004480  0.110000
 219     -3172.309326   -25.006188    0.000594  0.000141  0.011471  0.004471  0.110000
 220     -3172.309346   -25.018195    0.000620  0.000149  0.011072  0.004437  0.110000
 221     -3172.309364   -25.029968    0.000673  0.000161  0.010981  0.004388  0.110000
 222     -3172.309383   -25.041429    0.000701  0.000167  0.010862  0.004346  0.110000
 223     -3172.309402   -25.053292    0.000702  0.000168  0.010960  0.004334  0.110000
 224     -3172.309420   -25.064910    0.000700  0.000166  0.010918  0.004349  0.110000
 225     -3172.309438   -25.076376    0.000713  0.000166  0.010887  0.004360  0.110000
 226     -3172.309456   -25.087679    0.000738  0.000170  0.010871  0.004360  0.110000
 227     -3172.309474   -25.098781    0.000766  0.000175  0.010886  0.004349  0.110000
 228     -3172.309491   -25.109601    0.000783  0.000184  0.010916  0.004332  0.110000
 229     -3172.309508   -25.120192    0.000766  0.000196  0.010934  0.004313  0.110000
 230     -3172.309525   -25.130556    0.000908  0.000211  0.010775  0.004299  0.110000
 231     -3172.309533   -25.135778    0.000990  0.000200  0.009965  0.004207  0.110000
 232     -3172.309559   -25.151818    0.001182  0.000212  0.010646  0.004282  0.110000
 233     -3172.309576   -25.162573    0.001370  0.000228  0.010827  0.004326  0.110000
 234     -3172.309583   -25.167308    0.000980  0.000191  0.006958  0.002045  0.110000
 235     -3172.309590   -25.171777    0.001527  0.000260  0.017305  0.004013  0.110001
 236     -3172.309591   -25.172178    0.002084  0.000364  0.010598  0.003950  0.110000
 237     -3172.309611   -25.184941    0.001341  0.000215  0.019317  0.003864  0.110000
 238     -3172.309627   -25.194614    0.000661  0.000137  0.011530  0.004007  0.110000
 239     -3172.309644   -25.205572    0.000520  0.000128  0.012593  0.004599  0.110000
 240     -3172.309658   -25.214316    0.000798  0.000185  0.011215  0.004333  0.110000

         *************************************************************
         *  MAXIMUM NUMBER OF ITERATIONS REACHED - STOPPING IRC RUN  *
         *************************************************************


         *************************************************************
         *                        BACKWARD IRC                       *
         *************************************************************

Iteration    E(Eh)      dE(kcal/mol)  max(|G|)   RMS(G)   max(|S|)   RMS(S)   MW-Displ.
Convergence thresholds    0.003138    0.000080  0.000010  0.004000  0.002000
    0     -3172.269527   -0.031829    0.002335  0.000521  0.035612  0.003797  0.046517
    1     -3172.270200   -0.453997    0.008592  0.002012  0.088475  0.009271  0.110006
    2     -3172.271809   -1.463629    0.015217  0.003521  0.090696  0.009446  0.110002
    3     -3172.274013   -2.846443    0.020356  0.004475  0.088826  0.009325  0.110003
    4     -3172.276198   -4.218062    0.024112  0.004907  0.080643  0.008774  0.110001
    5     -3172.278019   -5.360220    0.026211  0.004989  0.060348  0.007494  0.110001
    6     -3172.279527   -6.306953    0.026220  0.004860  0.033134  0.006035  0.110001
    7     -3172.280870   -7.149669    0.025112  0.004652  0.024337  0.005252  0.110004
    8     -3172.282097   -7.919631    0.025170  0.004441  0.020127  0.004803  0.110003
    9     -3172.283229   -8.630129    0.025366  0.004246  0.020215  0.004504  0.110004
   10     -3172.284298   -9.300954    0.025420  0.004071  0.020150  0.004304  0.110003
   11     -3172.285303   -9.931461    0.025356  0.003915  0.019930  0.004181  0.110001
   12     -3172.286258   -10.530706    0.025193  0.003773  0.020348  0.004113  0.110001
   13     -3172.287168   -11.101548    0.024950  0.003643  0.021082  0.004081  0.110000
   14     -3172.288042   -11.650139    0.024642  0.003523  0.021473  0.004072  0.110001
   15     -3172.288884   -12.178252    0.024285  0.003411  0.021613  0.004075  0.110003
   16     -3172.289700   -12.690541    0.023893  0.003304  0.021577  0.004084  0.110001
   17     -3172.290477   -13.177974    0.023467  0.003201  0.021434  0.004096  0.110003
   18     -3172.291234   -13.653250    0.023021  0.003103  0.021211  0.004109  0.110001
   19     -3172.291959   -14.107695    0.022562  0.003008  0.020939  0.004124  0.110001
   20     -3172.292662   -14.549030    0.022093  0.002916  0.020638  0.004138  0.110002
   21     -3172.293339   -14.973978    0.021618  0.002828  0.020323  0.004152  0.110001
   22     -3172.293993   -15.384106    0.021143  0.002745  0.020004  0.004166  0.110002
   23     -3172.294620   -15.777838    0.020677  0.002669  0.019673  0.004179  0.110002
   24     -3172.295219   -16.153428    0.020235  0.002612  0.019256  0.004180  0.110000
   25     -3172.295786   -16.509265    0.019811  0.002574  0.018602  0.004112  0.110003
   26     -3172.296324   -16.847249    0.019360  0.002501  0.017912  0.004001  0.110001
```

27	-3172.296844	-17.173649	0.018911	0.002432	0.017634	0.004002	0.110001
28	-3172.297345	-17.487510	0.018470	0.002368	0.017336	0.003992	0.110001
29	-3172.297826	-17.789561	0.018048	0.002306	0.017479	0.003976	0.110002
30	-3172.298289	-18.080404	0.017615	0.002244	0.017603	0.003962	0.110002
31	-3172.298736	-18.360340	0.017190	0.002183	0.017733	0.003949	0.110001
32	-3172.299166	-18.630192	0.016777	0.002126	0.017847	0.003939	0.110001
33	-3172.299580	-18.890169	0.016369	0.002069	0.017945	0.003925	0.110001
34	-3172.299979	-19.140601	0.015969	0.002014	0.018029	0.003912	0.110001
35	-3172.300364	-19.381939	0.015578	0.001961	0.018100	0.003898	0.110000
36	-3172.300734	-19.614546	0.015195	0.001910	0.018160	0.003885	0.110000
37	-3172.301092	-19.838784	0.014817	0.001860	0.018208	0.003871	0.110002
38	-3172.301436	-20.054875	0.014444	0.001811	0.018250	0.003857	0.110002
39	-3172.301768	-20.263227	0.014074	0.001763	0.018281	0.003843	0.110001
40	-3172.302088	-20.463947	0.013673	0.001714	0.018305	0.003829	0.110001
41	-3172.302396	-20.657464	0.013333	0.001670	0.018326	0.003816	0.110001
42	-3172.302693	-20.843911	0.013000	0.001628	0.018321	0.003800	0.110001
43	-3172.302980	-21.023998	0.012675	0.001586	0.018313	0.003782	0.110001
44	-3172.303257	-21.197323	0.012358	0.001548	0.018298	0.003767	0.110001
45	-3172.303524	-21.365200	0.012047	0.001506	0.018273	0.003747	0.110002
46	-3172.303781	-21.526148	0.011744	0.001475	0.018236	0.003730	0.110000
47	-3172.304031	-21.683327	0.011447	0.001427	0.018191	0.003709	0.110001
48	-3172.304268	-21.831796	0.011160	0.001414	0.018129	0.003693	0.110001
49	-3172.304505	-21.980935	0.010872	0.001341	0.018067	0.003682	0.110000
50	-3172.304719	-22.115194	0.010597	0.001378	0.017947	0.003674	0.110000
51	-3172.304953	-22.261765	0.010309	0.001229	0.017888	0.003679	0.110001
52	-3172.305182	-22.405791	0.009985	0.001161	0.018968	0.003919	0.110001
53	-3172.305395	-22.539349	0.009690	0.001184	0.018946	0.003941	0.110001
54	-3172.305595	-22.664370	0.009473	0.001213	0.018828	0.003769	0.110000
55	-3172.305789	-22.786722	0.009244	0.001148	0.017689	0.003612	0.110001
56	-3172.305959	-22.893029	0.009003	0.001203	0.017652	0.003576	0.110001
57	-3172.306156	-23.016553	0.008760	0.001050	0.017376	0.003573	0.110000
58	-3172.306341	-23.132924	0.008473	0.000989	0.018289	0.003791	0.110000
59	-3172.306504	-23.235178	0.008227	0.001080	0.018129	0.003779	0.110000
60	-3172.306683	-23.347457	0.008033	0.000984	0.017858	0.003634	0.110000
61	-3172.306835	-23.442728	0.007804	0.001035	0.017241	0.003601	0.110001
62	-3172.306992	-23.541617	0.007608	0.000977	0.017207	0.003528	0.110001
63	-3172.307132	-23.629060	0.007401	0.001027	0.016663	0.003470	0.110000
64	-3172.307283	-23.723857	0.007206	0.000918	0.016547	0.003452	0.110000
65	-3172.307416	-23.807542	0.006988	0.000963	0.016586	0.003482	0.110000
66	-3172.307554	-23.894222	0.006808	0.000904	0.016482	0.003446	0.110001
67	-3172.307678	-23.971599	0.006617	0.000950	0.016013	0.003403	0.110001
68	-3172.307810	-24.054793	0.006443	0.000856	0.015918	0.003390	0.110000
69	-3172.307926	-24.127615	0.006248	0.000911	0.016005	0.003406	0.110000
70	-3172.308050	-24.205114	0.006085	0.000828	0.015906	0.003387	0.110001
71	-3172.308158	-24.272858	0.005903	0.000882	0.015871	0.003390	0.110000
72	-3172.308271	-24.343988	0.005748	0.000797	0.015886	0.003383	0.110000
73	-3172.308376	-24.409836	0.005570	0.000850	0.015830	0.003382	0.110000
74	-3172.308482	-24.476370	0.005428	0.000775	0.015613	0.003360	0.110000
75	-3172.308581	-24.538371	0.005264	0.000828	0.015592	0.003359	0.110001
76	-3172.308681	-24.601085	0.005134	0.000750	0.015268	0.003336	0.110001
77	-3172.308774	-24.659496	0.004978	0.000805	0.015374	0.003346	0.110000
78	-3172.308803	-24.677824	0.005304	0.000888	0.019997	0.002374	0.110001
79	-3172.308909	-24.744514	0.004795	0.000537	0.010579	0.003166	0.110002
80	-3172.309019	-24.812994	0.004606	0.000519	0.017393	0.003912	0.110001
81	-3172.309109	-24.869568	0.004449	0.000528	0.017469	0.003836	0.110001
82	-3172.309202	-24.928007	0.004320	0.000521	0.016569	0.003754	0.110000
83	-3172.309291	-24.983913	0.004203	0.000513	0.017104	0.003748	0.110001
84	-3172.309378	-25.038288	0.004094	0.000507	0.017207	0.003743	0.110001
85	-3172.309462	-25.090986	0.003990	0.000497	0.017164	0.003729	0.110001
86	-3172.309543	-25.141897	0.003890	0.000489	0.016979	0.003718	0.110000
87	-3172.309622	-25.191529	0.003796	0.000480	0.016660	0.003699	0.110001
88	-3172.309699	-25.239765	0.003705	0.000472	0.016283	0.003681	0.110000
89	-3172.309773	-25.286603	0.003618	0.000465	0.015927	0.003663	0.110000

90	-3172.309846	-25.332168	0.003535	0.000458	0.015603	0.003648	0.110000
91	-3172.309917	-25.376494	0.003456	0.000451	0.015299	0.003635	0.110000
92	-3172.309985	-25.419636	0.003379	0.000445	0.015012	0.003622	0.110001
93	-3172.310052	-25.461612	0.003304	0.000439	0.014737	0.003602	0.110000
94	-3172.310117	-25.502580	0.003233	0.000431	0.014548	0.003598	0.110000
95	-3172.310181	-25.542360	0.003160	0.000428	0.014233	0.003587	0.110000
96	-3172.310243	-25.581336	0.003091	0.000420	0.014065	0.003570	0.110001
97	-3172.310303	-25.618992	0.003021	0.000418	0.013787	0.003570	0.110000
98	-3172.310362	-25.656030	0.002955	0.000408	0.013638	0.003551	0.110000
99	-3172.310418	-25.691260	0.002883	0.000407	0.013183	0.003560	0.110000
100	-3172.310474	-25.726108	0.002820	0.000394	0.013070	0.003559	0.110000
101	-3172.310527	-25.759579	0.002743	0.000394	0.012430	0.003582	0.110000
102	-3172.310578	-25.791500	0.002682	0.000384	0.012362	0.003596	0.110000
103	-3172.310629	-25.823573	0.002608	0.000377	0.011751	0.003569	0.110000
104	-3172.310678	-25.854127	0.002540	0.000374	0.012457	0.003505	0.110000
105	-3172.310725	-25.883793	0.002464	0.000368	0.010998	0.003493	0.110000
106	-3172.310771	-25.912445	0.002405	0.000365	0.012546	0.003469	0.110000
107	-3172.310815	-25.940251	0.002331	0.000355	0.010281	0.003501	0.110000
108	-3172.310858	-25.967177	0.002274	0.000360	0.012376	0.003447	0.110000
109	-3172.310908	-25.998452	0.002210	0.000341	0.010564	0.003508	0.110000
110	-3172.310940	-26.018635	0.002147	0.000353	0.011247	0.003417	0.110000
111	-3172.310978	-26.042285	0.002093	0.000329	0.010578	0.003547	0.110000
112	-3172.311019	-26.068563	0.002028	0.000337	0.010990	0.003439	0.110000
113	-3172.311058	-26.092636	0.001972	0.000325	0.010025	0.003474	0.110000
114	-3172.311095	-26.115670	0.001913	0.000328	0.010435	0.003415	0.110000
115	-3172.311139	-26.143891	0.001860	0.000326	0.009983	0.003483	0.110000
116	-3172.311164	-26.159550	0.001803	0.000311	0.009799	0.003392	0.110000
117	-3172.311198	-26.180362	0.001753	0.000323	0.010284	0.003531	0.110000
118	-3172.311231	-26.201556	0.001699	0.000300	0.009966	0.003398	0.110000
119	-3172.311264	-26.221947	0.001767	0.000316	0.009994	0.003524	0.110000
120	-3172.311307	-26.248850	0.001605	0.000288	0.009976	0.003409	0.110000
121	-3172.311325	-26.260355	0.001570	0.000242	0.010609	0.003243	0.110000
122	-3172.311356	-26.279729	0.001491	0.000243	0.011709	0.003913	0.110000
123	-3172.311387	-26.299509	0.001566	0.000311	0.010792	0.003761	0.110000
124	-3172.311419	-26.319531	0.001409	0.000259	0.010434	0.003498	0.110000
125	-3172.311444	-26.335225	0.001604	0.000286	0.010749	0.003693	0.110000
126	-3172.311472	-26.352379	0.001394	0.000258	0.010453	0.003520	0.110000
127	-3172.311495	-26.367179	0.001553	0.000297	0.010528	0.003657	0.110000
128	-3172.311526	-26.386236	0.001304	0.000283	0.010646	0.003477	0.110000
129	-3172.311544	-26.398000	0.002156	0.000327	0.010385	0.003640	0.110000
130	-3172.311577	-26.418298	0.002181	0.000312	0.011030	0.003635	0.110000
131	-3172.311593	-26.428414	0.001742	0.000297	0.012264	0.003818	0.110000
132	-3172.311620	-26.445193	0.001189	0.000256	0.020312	0.004399	0.110000
133	-3172.311640	-26.457910	0.001448	0.000276	0.018577	0.004240	0.110000
134	-3172.311664	-26.473194	0.001507	0.000279	0.020393	0.004169	0.110000
135	-3172.311676	-26.480802	0.001783	0.000265	0.013161	0.002197	0.110000
136	-3172.311686	-26.487010	0.001114	0.000210	0.011246	0.003239	0.110001
137	-3172.311712	-26.502848	0.000990	0.000186	0.013215	0.004084	0.110000
138	-3172.311723	-26.509983	0.001487	0.000243	0.014145	0.003879	0.110000
139	-3172.311750	-26.527147	0.000954	0.000117	0.011543	0.004202	0.110000
140	-3172.311771	-26.540375	0.000929	0.000133	0.011931	0.004221	0.110000
141	-3172.311791	-26.552735	0.001104	0.000208	0.011601	0.003998	0.110000
142	-3172.311809	-26.564014	0.001262	0.000230	0.010693	0.003630	0.110000
143	-3172.311828	-26.576161	0.000870	0.000180	0.009505	0.003387	0.110000
144	-3172.311847	-26.587870	0.000813	0.000154	0.011473	0.003810	0.110000
145	-3172.311860	-26.596222	0.000791	0.000142	0.011343	0.004082	0.110000
146	-3172.311882	-26.610014	0.000773	0.000131	0.014338	0.004533	0.110000
147	-3172.311896	-26.618449	0.000993	0.000199	0.012752	0.004301	0.110000
148	-3172.311912	-26.628935	0.001133	0.000222	0.013901	0.004050	0.110000
149	-3172.311928	-26.638556	0.001045	0.000202	0.012344	0.003862	0.110000
150	-3172.311943	-26.648193	0.000873	0.000181	0.012383	0.003944	0.110000
151	-3172.311958	-26.657255	0.000794	0.000163	0.012584	0.003927	0.110000
152	-3172.311973	-26.666718	0.000813	0.000171	0.012958	0.003907	0.110000

153	-3172.311987	-26.675848	0.000741	0.000174	0.012333	0.003893	0.110000
154	-3172.312001	-26.684801	0.000668	0.000155	0.012048	0.003953	0.110000
155	-3172.312013	-26.691865	0.000810	0.000152	0.011766	0.003891	0.110000
156	-3172.312024	-26.699106	0.000822	0.000149	0.011569	0.003889	0.110000
157	-3172.312037	-26.706941	0.000855	0.000152	0.011546	0.003777	0.110000
158	-3172.312044	-26.711517	0.000867	0.000155	0.011561	0.003742	0.110000
159	-3172.312061	-26.721946	0.000783	0.000145	0.011997	0.003852	0.110000
160	-3172.312072	-26.728780	0.000893	0.000162	0.011948	0.003743	0.110000
161	-3172.312081	-26.734634	0.000791	0.000147	0.012114	0.003769	0.110000
162	-3172.312093	-26.742203	0.000841	0.000150	0.012027	0.003756	0.110000
163	-3172.312105	-26.749709	0.000760	0.000137	0.012568	0.003896	0.110000
164	-3172.312114	-26.755294	0.000915	0.000159	0.011700	0.003719	0.110000
165	-3172.312123	-26.761080	0.000749	0.000136	0.011970	0.003721	0.110000
166	-3172.312132	-26.766791	0.000800	0.000142	0.011895	0.003672	0.110000
167	-3172.312139	-26.770823	0.000790	0.000143	0.012567	0.003730	0.110000
168	-3172.312153	-26.779623	0.000657	0.000123	0.012844	0.003929	0.110000
169	-3172.312162	-26.785455	0.000706	0.000127	0.011546	0.003733	0.110000
170	-3172.312164	-26.787028	0.000817	0.000142	0.012225	0.003920	0.110000
171	-3172.312183	-26.798731	0.000637	0.000113	0.011321	0.003669	0.110000
172	-3172.312179	-26.795946	0.000408	0.000076	0.010341	0.001798	0.110000
173	-3172.312186	-26.800785	0.000436	0.000087	0.017105	0.003751	0.110001
174	-3172.312197	-26.807689	0.000342	0.000074	0.011997	0.004066	0.110000
175	-3172.312205	-26.812387	0.000420	0.000093	0.014060	0.004158	0.110000
176	-3172.312212	-26.817134	0.000448	0.000111	0.013003	0.004038	0.110000
177	-3172.312218	-26.820825	0.000703	0.000141	0.014012	0.004101	0.110000
178	-3172.312227	-26.826390	0.000341	0.000074	0.008202	0.003286	0.110000
179	-3172.312233	-26.830228	0.000371	0.000084	0.015353	0.004383	0.110000
180	-3172.312238	-26.833462	0.000649	0.000137	0.013374	0.003846	0.110000
181	-3172.312246	-26.838444	0.000675	0.000140	0.015437	0.003917	0.110000
182	-3172.312252	-26.842166	0.000259	0.000063	0.010655	0.003335	0.102558
183	-3172.312258	-26.845875	0.000648	0.000131	0.012528	0.004068	0.110000
184	-3172.312257	-26.845249	0.000734	0.000149	0.006857	0.001957	0.110000
185	-3172.312256	-26.844794	0.001265	0.000258	0.010381	0.003814	0.110000
186	-3172.312261	-26.847358	0.000914	0.000192	0.009831	0.002411	0.060072
187	-3172.312260	-26.846759	0.001183	0.000241	0.010165	0.003389	0.095600
188	-3172.312269	-26.852869	0.000966	0.000169	0.012524	0.003661	0.110000
189	-3172.312273	-26.855462	0.000893	0.000150	0.009363	0.003576	0.110000
190	-3172.312279	-26.859080	0.000215	0.000053	0.012804	0.003851	0.110000
191	-3172.312285	-26.862960	0.000320	0.000078	0.015046	0.004294	0.110000
192	-3172.312291	-26.866495	0.000475	0.000102	0.011501	0.003865	0.110000
193	-3172.312292	-26.866855	0.000658	0.000111	0.012505	0.004003	0.110000
194	-3172.312295	-26.868745	0.000637	0.000128	0.014184	0.004098	0.110000
195	-3172.312301	-26.872905	0.000514	0.000095	0.014573	0.003950	0.110000
196	-3172.312304	-26.874702	0.000332	0.000066	0.010876	0.002848	0.076229
197	-3172.312309	-26.877934	0.000228	0.000072	0.014578	0.003813	0.096050
198	-3172.312313	-26.880200	0.000371	0.000092	0.015760	0.004094	0.104094
199	-3172.312317	-26.882623	0.000363	0.000091	0.015293	0.003809	0.097812
200	-3172.312321	-26.885007	0.000350	0.000077	0.012997	0.003169	0.082113
201	-3172.312323	-26.886752	0.000379	0.000071	0.011883	0.002958	0.076145
202	-3172.312326	-26.888631	0.000324	0.000063	0.011850	0.003021	0.080532
203	-3172.312330	-26.890706	0.000333	0.000073	0.015306	0.004204	0.110000
204	-3172.312334	-26.893219	0.000295	0.000067	0.014886	0.003800	0.101286
205	-3172.312323	-26.886329	0.000338	0.000075	0.013139	0.003237	0.083030
206	-3172.312338	-26.895665	0.000359	0.000078	0.013501	0.003278	0.081324
207	-3172.312342	-26.898747	0.000335	0.000063	0.011281	0.002887	0.072917
208	-3172.312345	-26.900385	0.000263	0.000066	0.012278	0.003304	0.083206
209	-3172.312347	-26.901542	0.000660	0.000115	0.014824	0.004251	0.110000
210	-3172.312353	-26.905433	0.000600	0.000101	0.008403	0.001833	0.110000
211	-3172.312350	-26.903232	0.000723	0.000138	0.010765	0.003928	0.110000
212	-3172.312348	-26.902529	0.001060	0.000178	0.010504	0.002313	0.058652
213	-3172.312349	-26.902710	0.001130	0.000195	0.010776	0.002556	0.066098
214	-3172.312350	-26.903511	0.000776	0.000175	0.008178	0.003105	0.092374
215	-3172.312349	-26.903185	0.000895	0.000168	0.010522	0.002892	0.083258

216	-3172.312346	-26.901109	0.001607	0.000297	0.010491	0.003637	0.106073
217	-3172.312347	-26.901476	0.001490	0.000286	0.020164	0.003307	0.079089
218	-3172.312352	-26.904931	0.000513	0.000090	0.008985	0.001273	0.029429
219	-3172.312357	-26.907704	0.000342	0.000082	0.013423	0.003553	0.092366
220	-3172.312359	-26.909096	0.000239	0.000056	0.010996	0.003809	0.110000
221	-3172.312361	-26.910117	0.000239	0.000056	0.010586	0.003131	0.083111
222	-3172.312362	-26.911198	0.000339	0.000079	0.014301	0.003750	0.092801
223	-3172.312365	-26.913211	0.000418	0.000085	0.011025	0.002852	0.072188
224	-3172.312367	-26.914196	0.000363	0.000086	0.013992	0.003577	0.087713
225	-3172.312370	-26.916076	0.000280	0.000065	0.009092	0.002355	0.061498
226	-3172.312371	-26.916930	0.000148	0.000044	0.009951	0.002458	0.060730
227	-3172.312374	-26.918288	0.000198	0.000051	0.013404	0.003249	0.082192
228	-3172.312375	-26.919104	0.000163	0.000050	0.010246	0.002957	0.080792
229	-3172.312377	-26.920258	0.000174	0.000045	0.009702	0.002680	0.073066
230	-3172.312377	-26.920722	0.000188	0.000051	0.008701	0.002332	0.061105
231	-3172.312378	-26.921210	0.000212	0.000054	0.007868	0.002162	0.056683
232	-3172.312381	-26.922838	0.000363	0.000073	0.016033	0.004355	0.106843
233	-3172.312381	-26.922990	0.000200	0.000055	0.009592	0.002434	0.062798
234	-3172.312390	-26.928705	0.000174	0.000038	0.013281	0.003424	0.086957
235	-3172.312385	-26.925172	0.000132	0.000035	0.002272	0.000523	0.078603
236	-3172.312384	-26.924527	0.000346	0.000099	0.014331	0.004223	0.110001
237	-3172.312385	-26.925754	0.000395	0.000076	0.009061	0.002167	0.055873
238	-3172.312388	-26.927399	0.000409	0.000083	0.005156	0.001760	0.053018
239	-3172.312386	-26.925811	0.000501	0.000112	0.005270	0.001858	0.051814
240	-3172.312389	-26.927969	0.000573	0.000115	0.004812	0.001259	0.032487

```
*************************************************************
*  MAXIMUM NUMBER OF ITERATIONS REACHED - STOPPING IRC RUN  *
*************************************************************
```

IRC PATH SUMMARY

All gradients are in Eh/Bohr.

Step	E(Eh)	dE(kcal/mol)	max(|G|)	RMS(G)	SMW-Displ.
1	-3172.312389	-26.927969	0.000573	0.000115	-25.067209
2	-3172.312386	-26.925811	0.000501	0.000112	-25.034722
3	-3172.312388	-26.927399	0.000409	0.000083	-24.982908
4	-3172.312385	-26.925754	0.000395	0.000076	-24.929890
5	-3172.312384	-26.924527	0.000346	0.000099	-24.874017
6	-3172.312385	-26.925172	0.000132	0.000035	-24.764016
7	-3172.312390	-26.928705	0.000174	0.000038	-24.685414
8	-3172.312381	-26.922990	0.000200	0.000055	-24.598457
9	-3172.312381	-26.922838	0.000363	0.000073	-24.535659
10	-3172.312378	-26.921210	0.000212	0.000054	-24.428816
11	-3172.312377	-26.920722	0.000188	0.000051	-24.372134
12	-3172.312377	-26.920258	0.000174	0.000045	-24.311028
13	-3172.312375	-26.919104	0.000163	0.000050	-24.237963
14	-3172.312374	-26.918288	0.000198	0.000051	-24.157171
15	-3172.312371	-26.916930	0.000148	0.000044	-24.074979
16	-3172.312370	-26.916076	0.000280	0.000065	-24.014249
17	-3172.312367	-26.914196	0.000363	0.000086	-23.952752
18	-3172.312365	-26.913211	0.000418	0.000085	-23.865039
19	-3172.312362	-26.911198	0.000339	0.000079	-23.792850
20	-3172.312361	-26.910117	0.000239	0.000056	-23.700050
21	-3172.312359	-26.909096	0.000239	0.000056	-23.616939
22	-3172.312357	-26.907704	0.000342	0.000082	-23.506939
23	-3172.312352	-26.904931	0.000513	0.000090	-23.414573
24	-3172.312347	-26.901476	0.001490	0.000286	-23.385144
25	-3172.312346	-26.901109	0.001607	0.000297	-23.306055
26	-3172.312349	-26.903185	0.000895	0.000168	-23.199981

27	-3172.312350	-26.903511	0.000776	0.000175	-23.116724
28	-3172.312349	-26.902710	0.001130	0.000195	-23.024350
29	-3172.312348	-26.902529	0.001060	0.000178	-22.958252
30	-3172.312350	-26.903232	0.000723	0.000138	-22.899600
31	-3172.312353	-26.905433	0.000600	0.000101	-22.789600
32	-3172.312347	-26.901542	0.000660	0.000115	-22.679600
33	-3172.312345	-26.900385	0.000263	0.000066	-22.569600
34	-3172.312342	-26.898747	0.000335	0.000063	-22.486394
35	-3172.312338	-26.895665	0.000359	0.000078	-22.413477
36	-3172.312323	-26.886329	0.000338	0.000075	-22.332153
37	-3172.312334	-26.893219	0.000295	0.000067	-22.249123
38	-3172.312330	-26.890706	0.000333	0.000073	-22.147837
39	-3172.312326	-26.888631	0.000324	0.000063	-22.037837
40	-3172.312323	-26.886752	0.000379	0.000071	-21.957306
41	-3172.312321	-26.885007	0.000350	0.000077	-21.881161
42	-3172.312317	-26.882623	0.000363	0.000091	-21.799048
43	-3172.312313	-26.880200	0.000371	0.000092	-21.701236
44	-3172.312309	-26.877934	0.000228	0.000072	-21.597143
45	-3172.312304	-26.874702	0.000332	0.000066	-21.501092
46	-3172.312301	-26.872905	0.000514	0.000095	-21.424863
47	-3172.312295	-26.868745	0.000637	0.000128	-21.314863
48	-3172.312292	-26.866855	0.000658	0.000111	-21.204863
49	-3172.312291	-26.866495	0.000475	0.000102	-21.094863
50	-3172.312285	-26.862960	0.000320	0.000078	-20.984863
51	-3172.312279	-26.859080	0.000215	0.000053	-20.874863
52	-3172.312273	-26.855462	0.000893	0.000150	-20.764863
53	-3172.312269	-26.852869	0.000966	0.000169	-20.654863
54	-3172.312260	-26.846759	0.001183	0.000241	-20.544862
55	-3172.312261	-26.847358	0.000914	0.000192	-20.449262
56	-3172.312256	-26.844794	0.001265	0.000258	-20.389190
57	-3172.312257	-26.845249	0.000734	0.000149	-20.279190
58	-3172.312258	-26.845875	0.000648	0.000131	-20.169190
59	-3172.312252	-26.842166	0.000259	0.000063	-20.059189
60	-3172.312246	-26.838444	0.000675	0.000140	-19.956632
61	-3172.312238	-26.833462	0.000649	0.000137	-19.846632
62	-3172.312233	-26.830228	0.000371	0.000084	-19.736632
63	-3172.312227	-26.826390	0.000341	0.000074	-19.626631
64	-3172.312218	-26.820825	0.000703	0.000141	-19.516631
65	-3172.312212	-26.817134	0.000448	0.000111	-19.406631
66	-3172.312205	-26.812387	0.000420	0.000093	-19.296631
67	-3172.312197	-26.807689	0.000342	0.000074	-19.186631
68	-3172.312186	-26.800785	0.000436	0.000087	-19.076631
69	-3172.312179	-26.795946	0.000408	0.000076	-18.966630
70	-3172.312183	-26.798731	0.000637	0.000113	-18.856630
71	-3172.312164	-26.787028	0.000817	0.000142	-18.746630
72	-3172.312162	-26.785455	0.000706	0.000127	-18.636630
73	-3172.312153	-26.779623	0.000657	0.000123	-18.526630
74	-3172.312139	-26.770823	0.000790	0.000143	-18.416630
75	-3172.312132	-26.766791	0.000800	0.000142	-18.306630
76	-3172.312123	-26.761080	0.000749	0.000136	-18.196630
77	-3172.312114	-26.755294	0.000915	0.000159	-18.086630
78	-3172.312105	-26.749709	0.000760	0.000137	-17.976630
79	-3172.312093	-26.742203	0.000841	0.000150	-17.866630
80	-3172.312081	-26.734634	0.000791	0.000147	-17.756629
81	-3172.312072	-26.728780	0.000893	0.000162	-17.646629
82	-3172.312061	-26.721946	0.000783	0.000145	-17.536629
83	-3172.312044	-26.711517	0.000867	0.000155	-17.426629
84	-3172.312037	-26.706941	0.000855	0.000152	-17.316629
85	-3172.312024	-26.699106	0.000822	0.000149	-17.206629
86	-3172.312013	-26.691865	0.000810	0.000152	-17.096629
87	-3172.312001	-26.684801	0.000668	0.000155	-16.986629
88	-3172.311987	-26.675848	0.000741	0.000174	-16.876629
89	-3172.311973	-26.666718	0.000813	0.000171	-16.766629

90	-3172.311958	-26.657255	0.000794	0.000163	-16.656629
91	-3172.311943	-26.648193	0.000873	0.000181	-16.546629
92	-3172.311928	-26.638556	0.001045	0.000202	-16.436629
93	-3172.311912	-26.628935	0.001133	0.000222	-16.326629
94	-3172.311896	-26.618449	0.000993	0.000199	-16.216628
95	-3172.311882	-26.610014	0.000773	0.000131	-16.106628
96	-3172.311860	-26.596222	0.000791	0.000142	-15.996628
97	-3172.311847	-26.587870	0.000813	0.000154	-15.886628
98	-3172.311828	-26.576161	0.000870	0.000180	-15.776628
99	-3172.311809	-26.564014	0.001262	0.000230	-15.666628
100	-3172.311791	-26.552735	0.001104	0.000208	-15.556628
101	-3172.311771	-26.540375	0.000929	0.000133	-15.446627
102	-3172.311750	-26.527147	0.000954	0.000117	-15.336627
103	-3172.311723	-26.509983	0.001487	0.000243	-15.226627
104	-3172.311712	-26.502848	0.000990	0.000186	-15.116627
105	-3172.311686	-26.487010	0.001114	0.000210	-15.006626
106	-3172.311676	-26.480802	0.001783	0.000265	-14.896626
107	-3172.311664	-26.473194	0.001507	0.000279	-14.786625
108	-3172.311640	-26.457910	0.001448	0.000276	-14.676625
109	-3172.311620	-26.445193	0.001189	0.000256	-14.566625
110	-3172.311593	-26.428414	0.001742	0.000297	-14.456625
111	-3172.311577	-26.418298	0.002181	0.000312	-14.346625
112	-3172.311544	-26.398000	0.002156	0.000327	-14.236625
113	-3172.311526	-26.386236	0.001304	0.000283	-14.126624
114	-3172.311495	-26.367179	0.001553	0.000297	-14.016624
115	-3172.311472	-26.352379	0.001394	0.000258	-13.906624
116	-3172.311444	-26.335225	0.001604	0.000286	-13.796624
117	-3172.311419	-26.319531	0.001409	0.000259	-13.686624
118	-3172.311387	-26.299509	0.001566	0.000311	-13.576624
119	-3172.311356	-26.279729	0.001491	0.000243	-13.466624
120	-3172.311325	-26.260355	0.001570	0.000242	-13.356623
121	-3172.311307	-26.248850	0.001605	0.000288	-13.246623
122	-3172.311264	-26.221947	0.001767	0.000316	-13.136623
123	-3172.311231	-26.201556	0.001699	0.000300	-13.026623
124	-3172.311198	-26.180362	0.001753	0.000323	-12.916623
125	-3172.311164	-26.159550	0.001803	0.000311	-12.806622
126	-3172.311139	-26.143891	0.001860	0.000326	-12.696622
127	-3172.311095	-26.115670	0.001913	0.000328	-12.586622
128	-3172.311058	-26.092636	0.001972	0.000325	-12.476622
129	-3172.311019	-26.068563	0.002028	0.000337	-12.366622
130	-3172.310978	-26.042285	0.002093	0.000329	-12.256622
131	-3172.310940	-26.018635	0.002147	0.000353	-12.146622
132	-3172.310908	-25.998452	0.002210	0.000341	-12.036622
133	-3172.310858	-25.967177	0.002274	0.000360	-11.926621
134	-3172.310815	-25.940251	0.002331	0.000355	-11.816621
135	-3172.310771	-25.912445	0.002405	0.000365	-11.706621
136	-3172.310725	-25.883793	0.002464	0.000368	-11.596621
137	-3172.310678	-25.854127	0.002540	0.000374	-11.486620
138	-3172.310629	-25.823573	0.002608	0.000377	-11.376620
139	-3172.310578	-25.791500	0.002682	0.000384	-11.266620
140	-3172.310527	-25.759579	0.002743	0.000394	-11.156619
141	-3172.310474	-25.726108	0.002820	0.000394	-11.046619
142	-3172.310418	-25.691260	0.002883	0.000407	-10.936619
143	-3172.310362	-25.656030	0.002955	0.000408	-10.826618
144	-3172.310303	-25.618992	0.003021	0.000418	-10.716618
145	-3172.310243	-25.581336	0.003091	0.000420	-10.606618
146	-3172.310181	-25.542360	0.003160	0.000428	-10.496618
147	-3172.310117	-25.502580	0.003233	0.000431	-10.386617
148	-3172.310052	-25.461612	0.003304	0.000439	-10.276617
149	-3172.309985	-25.419636	0.003379	0.000445	-10.166616
150	-3172.309917	-25.376494	0.003456	0.000451	-10.056616
151	-3172.309846	-25.332168	0.003535	0.000458	-9.946616
152	-3172.309773	-25.286603	0.003618	0.000465	-9.836616

153	-3172.309699	-25.239765	0.003705	0.000472	-9.726615
154	-3172.309622	-25.191529	0.003796	0.000480	-9.616615
155	-3172.309543	-25.141897	0.003890	0.000489	-9.506615
156	-3172.309462	-25.090986	0.003990	0.000497	-9.396615
157	-3172.309378	-25.038288	0.004094	0.000507	-9.286614
158	-3172.309291	-24.983913	0.004203	0.000513	-9.176613
159	-3172.309202	-24.928007	0.004320	0.000521	-9.066613
160	-3172.309109	-24.869568	0.004449	0.000528	-8.956612
161	-3172.309019	-24.812994	0.004606	0.000519	-8.846611
162	-3172.308909	-24.744514	0.004795	0.000537	-8.736611
163	-3172.308803	-24.677824	0.005304	0.000888	-8.626609
164	-3172.308774	-24.659496	0.004978	0.000805	-8.516608
165	-3172.308681	-24.601085	0.005134	0.000750	-8.406608
166	-3172.308581	-24.538371	0.005264	0.000828	-8.296607
167	-3172.308482	-24.476370	0.005428	0.000775	-8.186606
168	-3172.308376	-24.409836	0.005570	0.000850	-8.076606
169	-3172.308271	-24.343988	0.005748	0.000797	-7.966606
170	-3172.308158	-24.272858	0.005903	0.000882	-7.856605
171	-3172.308050	-24.205114	0.006085	0.000828	-7.746605
172	-3172.307926	-24.127615	0.006248	0.000911	-7.636605
173	-3172.307810	-24.054793	0.006443	0.000856	-7.526605
174	-3172.307678	-23.971599	0.006617	0.000950	-7.416604
175	-3172.307554	-23.894222	0.006808	0.000904	-7.306604
176	-3172.307416	-23.807542	0.006988	0.000963	-7.196603
177	-3172.307283	-23.723857	0.007206	0.000918	-7.086603
178	-3172.307132	-23.629060	0.007401	0.001027	-6.976602
179	-3172.306992	-23.541617	0.007608	0.000977	-6.866602
180	-3172.306835	-23.442728	0.007804	0.001035	-6.756601
181	-3172.306683	-23.347457	0.008033	0.000984	-6.646600
182	-3172.306504	-23.235178	0.008227	0.001080	-6.536600
183	-3172.306341	-23.132924	0.008473	0.000989	-6.426600
184	-3172.306156	-23.016553	0.008760	0.001050	-6.316599
185	-3172.305959	-22.893029	0.009003	0.001203	-6.206599
186	-3172.305789	-22.786722	0.009244	0.001148	-6.096598
187	-3172.305595	-22.664370	0.009473	0.001213	-5.986597
188	-3172.305395	-22.539349	0.009690	0.001184	-5.876597
189	-3172.305182	-22.405791	0.009985	0.001161	-5.766596
190	-3172.304953	-22.261765	0.010309	0.001229	-5.656595
191	-3172.304719	-22.115194	0.010597	0.001378	-5.546594
192	-3172.304505	-21.980935	0.010872	0.001341	-5.436594
193	-3172.304268	-21.831796	0.011160	0.001414	-5.326594
194	-3172.304031	-21.683327	0.011447	0.001427	-5.216592
195	-3172.303781	-21.526148	0.011744	0.001475	-5.106591
196	-3172.303524	-21.365200	0.012047	0.001506	-4.996591
197	-3172.303257	-21.197323	0.012358	0.001548	-4.886589
198	-3172.302980	-21.023998	0.012675	0.001586	-4.776588
199	-3172.302693	-20.843911	0.013000	0.001628	-4.666587
200	-3172.302396	-20.657464	0.013333	0.001670	-4.556586
201	-3172.302088	-20.463947	0.013673	0.001714	-4.446586
202	-3172.301768	-20.263227	0.014074	0.001763	-4.336585
203	-3172.301436	-20.054875	0.014444	0.001811	-4.226584
204	-3172.301092	-19.838784	0.014817	0.001860	-4.116582
205	-3172.300734	-19.614546	0.015195	0.001910	-4.006581
206	-3172.300364	-19.381939	0.015578	0.001961	-3.896580
207	-3172.299979	-19.140601	0.015969	0.002014	-3.786580
208	-3172.299580	-18.890169	0.016369	0.002069	-3.676579
209	-3172.299166	-18.630192	0.016777	0.002126	-3.566578
210	-3172.298736	-18.360340	0.017190	0.002183	-3.456577
211	-3172.298289	-18.080404	0.017615	0.002244	-3.346577
212	-3172.297826	-17.789561	0.018048	0.002306	-3.236574
213	-3172.297345	-17.487510	0.018470	0.002368	-3.126572
214	-3172.296844	-17.173649	0.018911	0.002432	-3.016572
215	-3172.296324	-16.847249	0.019360	0.002501	-2.906570

216	-3172.295786	-16.509265	0.019811	0.002574	-2.796569	
217	-3172.295219	-16.153428	0.020235	0.002612	-2.686566	
218	-3172.294620	-15.777838	0.020677	0.002669	-2.576566	
219	-3172.293993	-15.384106	0.021143	0.002745	-2.466564	
220	-3172.293339	-14.973978	0.021618	0.002828	-2.356562	
221	-3172.292662	-14.549030	0.022093	0.002916	-2.246561	
222	-3172.291959	-14.107695	0.022562	0.003008	-2.136559	
223	-3172.291234	-13.653250	0.023021	0.003103	-2.026558	
224	-3172.290477	-13.177974	0.023467	0.003201	-1.916557	
225	-3172.289700	-12.690541	0.023893	0.003304	-1.806554	
226	-3172.288884	-12.178252	0.024285	0.003411	-1.696553	
227	-3172.288042	-11.650139	0.024642	0.003523	-1.586550	
228	-3172.287168	-11.101548	0.024950	0.003643	-1.476549	
229	-3172.286258	-10.530706	0.025193	0.003773	-1.366548	
230	-3172.285303	-9.931461	0.025356	0.003915	-1.256548	
231	-3172.284298	-9.300954	0.025420	0.004071	-1.146546	
232	-3172.283229	-8.630129	0.025366	0.004246	-1.036543	
233	-3172.282097	-7.919631	0.025170	0.004441	-0.926539	
234	-3172.280870	-7.149669	0.025112	0.004652	-0.816536	
235	-3172.279527	-6.306953	0.026220	0.004860	-0.706531	
236	-3172.278019	-5.360220	0.026211	0.004989	-0.596530	
237	-3172.276198	-4.218062	0.024112	0.004907	-0.486529	
238	-3172.274013	-2.846443	0.020356	0.004475	-0.376528	
239	-3172.271809	-1.463629	0.015217	0.003521	-0.266525	
240	-3172.270200	-0.453997	0.008592	0.002012	-0.156523	
241	-3172.269527	-0.031829	0.002335	0.000521	-0.046517	
242	-3172.269476	0.000000	0.000099	0.000017	0.000000	<= TS
243	-3172.269520	-0.027450	0.002342	0.000484	0.046517	
244	-3172.269916	-0.275883	0.007553	0.001402	0.156520	
245	-3172.270525	-0.658260	0.011792	0.002046	0.266525	
246	-3172.271212	-1.088884	0.014613	0.002453	0.376528	
247	-3172.271930	-1.539583	0.016344	0.002712	0.486531	
248	-3172.272675	-2.007063	0.017837	0.002888	0.596533	
249	-3172.273438	-2.485716	0.019266	0.003018	0.706537	
250	-3172.274215	-2.973169	0.020448	0.003120	0.816539	
251	-3172.275003	-3.468231	0.021453	0.003203	0.926540	
252	-3172.275805	-3.971246	0.022324	0.003273	1.036541	
253	-3172.276613	-4.477928	0.023089	0.003333	1.146541	
254	-3172.277428	-4.989673	0.023767	0.003385	1.256545	
255	-3172.278250	-5.505355	0.024370	0.003431	1.366545	
256	-3172.279077	-6.024395	0.024905	0.003471	1.476548	
257	-3172.279907	-6.545423	0.025375	0.003505	1.586550	
258	-3172.280743	-7.069892	0.025785	0.003533	1.696550	
259	-3172.281581	-7.595477	0.026129	0.003556	1.806552	
260	-3172.282421	-8.122543	0.026407	0.003572	1.916553	
261	-3172.283261	-8.650125	0.026619	0.003582	2.026555	
262	-3172.284102	-9.177559	0.026760	0.003585	2.136557	
263	-3172.284941	-9.704168	0.026829	0.003581	2.246560	
264	-3172.285778	-10.229219	0.026825	0.003570	2.356562	
265	-3172.286610	-10.751701	0.026747	0.003550	2.466564	
266	-3172.287438	-11.270838	0.026591	0.003521	2.576564	
267	-3172.288244	-11.776484	0.026353	0.003483	2.686565	
268	-3172.289048	-12.281275	0.026042	0.003436	2.796566	
269	-3172.289837	-12.776349	0.025657	0.003381	2.906568	
270	-3172.290605	-13.258248	0.025197	0.003319	3.016572	
271	-3172.291349	-13.725015	0.024650	0.003248	3.126573	
272	-3172.292073	-14.179629	0.024014	0.003166	3.236577	
273	-3172.292778	-14.621653	0.023300	0.003075	3.346577	
274	-3172.293459	-15.049011	0.022512	0.002976	3.456580	
275	-3172.294113	-15.459845	0.021649	0.002869	3.566583	
276	-3172.294741	-15.853747	0.020714	0.002754	3.676585	
277	-3172.295340	-16.229863	0.019709	0.002633	3.786587	
278	-3172.295910	-16.587208	0.018638	0.002505	3.896587	

279	-3172.296448	-16.925115	0.017510	0.002371	4.006587
280	-3172.296956	-17.243356	0.016329	0.002231	4.116589
281	-3172.297430	-17.541369	0.015104	0.002088	4.226589
282	-3172.297874	-17.819385	0.013845	0.001943	4.336591
283	-3172.298285	-18.077364	0.012563	0.001797	4.446593
284	-3172.298664	-18.315574	0.011276	0.001651	4.556593
285	-3172.299014	-18.534798	0.009998	0.001507	4.666593
286	-3172.299333	-18.735511	0.008750	0.001369	4.776594
287	-3172.299625	-18.918287	0.007548	0.001228	4.886596
288	-3172.299889	-19.084003	0.006885	0.001110	4.996596
289	-3172.300135	-19.238446	0.006349	0.000982	5.106596
290	-3172.300346	-19.370954	0.005821	0.000946	5.216597
291	-3172.300570	-19.511349	0.005171	0.000744	5.326598
292	-3172.300731	-19.612245	0.005042	0.001052	5.436601
293	-3172.300948	-19.748362	0.004342	0.000617	5.546604
294	-3172.301124	-19.859111	0.003901	0.000618	5.656607
295	-3172.301296	-19.966749	0.003543	0.000568	5.766608
296	-3172.301463	-20.071945	0.003239	0.000526	5.876609
297	-3172.301627	-20.174425	0.002981	0.000502	5.986609
298	-3172.301790	-20.276826	0.002769	0.000488	6.096610
299	-3172.301951	-20.378092	0.002594	0.000477	6.206611
300	-3172.302114	-20.480290	0.002451	0.000465	6.316611
301	-3172.302277	-20.582891	0.002332	0.000453	6.426612
302	-3172.302442	-20.686139	0.002233	0.000441	6.536613
303	-3172.302604	-20.787523	0.002148	0.000434	6.646613
304	-3172.302760	-20.885541	0.002073	0.000454	6.756614
305	-3172.302909	-20.979413	0.002007	0.000489	6.866615
306	-3172.303059	-21.073287	0.001948	0.000483	6.976616
307	-3172.303207	-21.166351	0.001904	0.000474	7.086617
308	-3172.303353	-21.257830	0.001863	0.000468	7.196618
309	-3172.303497	-21.348073	0.001825	0.000468	7.306618
310	-3172.303638	-21.436713	0.001868	0.000469	7.416619
311	-3172.303776	-21.522957	0.001918	0.000470	7.526620
312	-3172.303910	-21.607203	0.001957	0.000468	7.636620
313	-3172.304041	-21.689396	0.001988	0.000466	7.746621
314	-3172.304168	-21.769523	0.002016	0.000465	7.856622
315	-3172.304293	-21.847549	0.002045	0.000463	7.966622
316	-3172.304414	-21.923440	0.002074	0.000461	8.076623
317	-3172.304532	-21.997394	0.002098	0.000459	8.186624
318	-3172.304646	-22.069206	0.002125	0.000457	8.296624
319	-3172.304757	-22.139030	0.002151	0.000456	8.406624
320	-3172.304865	-22.206891	0.002173	0.000454	8.516625
321	-3172.304971	-22.272873	0.002193	0.000453	8.626625
322	-3172.305073	-22.336936	0.002212	0.000451	8.736625
323	-3172.305172	-22.399168	0.002226	0.000450	8.846626
324	-3172.305270	-22.460633	0.002136	0.000432	8.956626
325	-3172.305361	-22.518131	0.002291	0.000453	9.066626
326	-3172.305451	-22.574296	0.002221	0.000439	9.176626
327	-3172.305540	-22.629862	0.002255	0.000444	9.286627
328	-3172.305622	-22.681619	0.002195	0.000438	9.396628
329	-3172.305706	-22.734156	0.002068	0.000432	9.506628
330	-3172.305785	-22.783925	0.001767	0.000425	9.616629
331	-3172.305857	-22.828921	0.001701	0.000405	9.726629
332	-3172.305930	-22.874698	0.001892	0.000410	9.836629
333	-3172.305998	-22.917544	0.001860	0.000420	9.946630
334	-3172.306061	-22.956985	0.002035	0.000455	10.056630
335	-3172.306121	-22.995019	0.002480	0.000470	10.166630
336	-3172.306177	-23.029916	0.003041	0.000519	10.276630
337	-3172.306239	-23.068804	0.002381	0.000429	10.386631
338	-3172.306294	-23.103430	0.001465	0.000396	10.496631
339	-3172.306352	-23.139559	0.001126	0.000297	10.606632
340	-3172.306391	-23.164018	0.001971	0.000418	10.716632
341	-3172.306442	-23.196127	0.001305	0.000281	10.826633

342	-3172.306475	-23.216693	0.002004	0.000419	10.936633
343	-3172.306535	-23.254604	0.000950	0.000210	11.046634
344	-3172.306573	-23.278136	0.001508	0.000352	11.156635
345	-3172.306619	-23.307042	0.000983	0.000274	11.266635
346	-3172.306642	-23.321666	0.002956	0.000516	11.376636
347	-3172.306652	-23.328253	0.002741	0.000478	11.486636
348	-3172.306698	-23.357007	0.001055	0.000223	11.596638
349	-3172.306733	-23.378823	0.001353	0.000283	11.706638
350	-3172.306770	-23.402101	0.001396	0.000258	11.816639
351	-3172.306804	-23.423514	0.001449	0.000261	11.926639
352	-3172.306828	-23.438332	0.001486	0.000277	12.036639
353	-3172.306868	-23.463653	0.001341	0.000267	12.146639
354	-3172.306902	-23.484635	0.001242	0.000273	12.256640
355	-3172.306930	-23.502376	0.001317	0.000280	12.366640
356	-3172.306961	-23.521911	0.001426	0.000273	12.476640
357	-3172.306989	-23.539670	0.001539	0.000272	12.586640
358	-3172.307018	-23.557920	0.001568	0.000268	12.696640
359	-3172.307047	-23.575739	0.001553	0.000266	12.806640
360	-3172.307075	-23.593264	0.001539	0.000264	12.916640
361	-3172.307102	-23.610245	0.001547	0.000266	13.026641
362	-3172.307129	-23.626983	0.001539	0.000266	13.136641
363	-3172.307155	-23.643362	0.001518	0.000270	13.246641
364	-3172.307182	-23.660528	0.001496	0.000272	13.356641
365	-3172.307208	-23.676756	0.001506	0.000274	13.466641
366	-3172.307232	-23.691960	0.001540	0.000272	13.576641
367	-3172.307258	-23.707921	0.001585	0.000271	13.686642
368	-3172.307283	-23.723662	0.001608	0.000271	13.796642
369	-3172.307307	-23.739106	0.001600	0.000271	13.906642
370	-3172.307332	-23.754446	0.001584	0.000269	14.016642
371	-3172.307356	-23.769513	0.001604	0.000272	14.126642
372	-3172.307380	-23.784473	0.001599	0.000274	14.236642
373	-3172.307403	-23.799329	0.001596	0.000275	14.346643
374	-3172.307428	-23.814997	0.001602	0.000275	14.456643
375	-3172.307450	-23.828885	0.001598	0.000274	14.566643
376	-3172.307467	-23.839212	0.001628	0.000275	14.676643
377	-3172.307498	-23.858850	0.001637	0.000272	14.786643
378	-3172.307521	-23.872971	0.001671	0.000283	14.896643
379	-3172.307529	-23.878511	0.001287	0.000254	15.006643
380	-3172.307551	-23.892143	0.000998	0.000186	15.116645
381	-3172.307578	-23.908893	0.000590	0.000124	15.226645
382	-3172.307600	-23.923145	0.000717	0.000163	15.336645
383	-3172.307625	-23.938456	0.000857	0.000178	15.446646
384	-3172.307649	-23.953514	0.000902	0.000189	15.556646
385	-3172.307673	-23.968722	0.000857	0.000184	15.666646
386	-3172.307698	-23.984053	0.000825	0.000181	15.776646
387	-3172.307721	-23.998661	0.000818	0.000190	15.886646
388	-3172.307746	-24.014430	0.000839	0.000203	15.996647
389	-3172.307771	-24.029934	0.001086	0.000219	16.106647
390	-3172.307794	-24.044800	0.001342	0.000237	16.216647
391	-3172.307823	-24.062645	0.001594	0.000254	16.326647
392	-3172.307843	-24.075050	0.001470	0.000225	16.436647
393	-3172.307866	-24.089929	0.001607	0.000232	16.546648
394	-3172.307891	-24.105358	0.001927	0.000280	16.656648
395	-3172.307908	-24.116225	0.001937	0.000296	16.766648
396	-3172.307937	-24.134587	0.001805	0.000291	16.876648
397	-3172.307959	-24.148155	0.001608	0.000281	16.986648
398	-3172.307983	-24.163473	0.001441	0.000274	17.096648
399	-3172.308011	-24.180691	0.001230	0.000260	17.206648
400	-3172.308034	-24.194997	0.000942	0.000240	17.316648
401	-3172.308061	-24.212012	0.000944	0.000222	17.426648
402	-3172.308085	-24.226917	0.000982	0.000215	17.536648
403	-3172.308108	-24.241381	0.000948	0.000219	17.646649
404	-3172.308132	-24.256476	0.000877	0.000226	17.756649

405	-3172.308150	-24.267753	0.001098	0.000234	17.866649
406	-3172.308179	-24.286279	0.001206	0.000237	17.976649
407	-3172.308196	-24.296699	0.001363	0.000252	18.086649
408	-3172.308228	-24.317134	0.001517	0.000266	18.196649
409	-3172.308251	-24.331458	0.000758	0.000159	18.306650
410	-3172.308276	-24.347264	0.000645	0.000149	18.416650
411	-3172.308297	-24.360256	0.001057	0.000234	18.526650
412	-3172.308321	-24.375402	0.000961	0.000223	18.636650
413	-3172.308345	-24.390213	0.000843	0.000206	18.746650
414	-3172.308368	-24.404723	0.000880	0.000204	18.856650
415	-3172.308391	-24.419146	0.000960	0.000205	18.966651
416	-3172.308414	-24.433522	0.000957	0.000204	19.076651
417	-3172.308436	-24.447678	0.000910	0.000211	19.186651
418	-3172.308459	-24.461774	0.000836	0.000221	19.296651
419	-3172.308481	-24.475854	0.000940	0.000228	19.406651
420	-3172.308503	-24.489780	0.000958	0.000228	19.516651
421	-3172.308526	-24.503991	0.000823	0.000219	19.626652
422	-3172.308549	-24.518220	0.000833	0.000210	19.736652
423	-3172.308572	-24.532506	0.000924	0.000207	19.846652
424	-3172.308594	-24.546529	0.000994	0.000210	19.956652
425	-3172.308616	-24.560264	0.001008	0.000212	20.066652
426	-3172.308638	-24.573916	0.000992	0.000210	20.176652
427	-3172.308659	-24.587482	0.000969	0.000207	20.286652
428	-3172.308681	-24.600900	0.000940	0.000206	20.396653
429	-3172.308702	-24.614215	0.000910	0.000209	20.506653
430	-3172.308723	-24.627389	0.000892	0.000210	20.616653
431	-3172.308744	-24.640476	0.000894	0.000210	20.726653
432	-3172.308764	-24.653513	0.000914	0.000209	20.836653
433	-3172.308785	-24.666489	0.000939	0.000208	20.946653
434	-3172.308806	-24.679407	0.000955	0.000209	21.056653
435	-3172.308826	-24.692309	0.000956	0.000209	21.166653
436	-3172.308847	-24.705247	0.000940	0.000208	21.276653
437	-3172.308868	-24.718340	0.000909	0.000207	21.386653
438	-3172.308884	-24.728426	0.000869	0.000205	21.496653
439	-3172.308911	-24.745773	0.000831	0.000207	21.606653
440	-3172.308923	-24.753338	0.000836	0.000195	21.716653
441	-3172.308946	-24.767228	0.000870	0.000204	21.826654
442	-3172.308966	-24.779858	0.000905	0.000217	21.936654
443	-3172.308982	-24.790090	0.000844	0.000196	22.046654
444	-3172.309003	-24.803530	0.000864	0.000211	22.156654
445	-3172.309022	-24.814944	0.000889	0.000207	22.266654
446	-3172.309037	-24.824595	0.000918	0.000213	22.376654
447	-3172.309061	-24.839560	0.000931	0.000214	22.486654
448	-3172.309083	-24.853545	0.000964	0.000210	22.596654
449	-3172.309098	-24.863134	0.000918	0.000185	22.706655
450	-3172.309102	-24.865655	0.000677	0.000159	22.816655
451	-3172.309119	-24.876012	0.000739	0.000148	22.926655
452	-3172.309141	-24.890018	0.000878	0.000176	23.036655
453	-3172.309157	-24.900062	0.001024	0.000248	23.146655
454	-3172.309176	-24.911742	0.000557	0.000135	23.256655
455	-3172.309194	-24.923315	0.000792	0.000176	23.366655
456	-3172.309210	-24.933344	0.000695	0.000157	23.476656
457	-3172.309229	-24.944852	0.000419	0.000079	23.586656
458	-3172.309247	-24.956385	0.000810	0.000192	23.696656
459	-3172.309267	-24.968634	0.000742	0.000181	23.806656
460	-3172.309287	-24.981480	0.000602	0.000152	23.916657
461	-3172.309307	-24.993997	0.000597	0.000140	24.026657
462	-3172.309326	-25.006188	0.000594	0.000141	24.136657
463	-3172.309346	-25.018195	0.000620	0.000149	24.246657
464	-3172.309364	-25.029968	0.000673	0.000161	24.356657
465	-3172.309383	-25.041429	0.000701	0.000167	24.466657
466	-3172.309402	-25.053292	0.000702	0.000168	24.576657
467	-3172.309420	-25.064910	0.000700	0.000166	24.686657

```
 468     -3172.309438   -25.076376    0.000713  0.000166  24.796657
 469     -3172.309456   -25.087679    0.000738  0.000170  24.906657
 470     -3172.309474   -25.098781    0.000766  0.000175  25.016657
 471     -3172.309491   -25.109601    0.000783  0.000184  25.126657
 472     -3172.309508   -25.120192    0.000766  0.000196  25.236658
 473     -3172.309525   -25.130556    0.000908  0.000211  25.346658
 474     -3172.309533   -25.135778    0.000990  0.000200  25.456658
 475     -3172.309559   -25.151818    0.001182  0.000212  25.566658
 476     -3172.309576   -25.162573    0.001370  0.000228  25.676658
 477     -3172.309583   -25.167308    0.000980  0.000191  25.786658
 478     -3172.309590   -25.171777    0.001527  0.000260  25.896659
 479     -3172.309591   -25.172178    0.002084  0.000364  26.006659
 480     -3172.309611   -25.184941    0.001341  0.000215  26.116659
 481     -3172.309627   -25.194614    0.000661  0.000137  26.226659
 482     -3172.309644   -25.205572    0.000520  0.000128  26.336660
 483     -3172.309658   -25.214316    0.000798  0.000185  26.446660

-------------------------------
SUGGESTED CITATIONS FOR THIS RUN
-------------------------------
```

Below you find a list of papers that are relevant to this ORCA run
We neither can nor want to force you to cite these papers, but we appreciate if you do
You receive ORCA, which is the product of decades of hard work by many enthusiastic individuals, for free
The only thing we kindly ask in return is that you cite our papers,
We deeply appreciate it, if you show your appreciation for ORCA by not just citing the generic ORCA reference.

Please note that relegating all ORCA citations to the supporting information does *not* help us.
SI sections are not indexed - citations you put there will not count into any citation statistics
But we need these citations in order to attract the funding resources that allow us to do what we are doing

Therefore, if you are a happy ORCA user, please consider citing a few of the papers listed below in the main body of your paper

In addition to the list printed below, the program has created the file LQA_TS-OMe.bibtex that contains the list in bibtex format
You can import this file easily into all common literature databanks and citation aid programs

It goes without saying that in many instances, there are alternative algorithms to achieve similar
results as the ones you have gotten from ORCA. It is, of course, also the case that in some instances
ORCA just re-implements algorithms worked out by others. We are fully aware of that and we are also
fully appreciative of our colleagues work. Hence this citation list should not be read as indicating
that the listed papers, which are focused on our own work, are the only ones worth citing. It simply
meant to make it easier for users to cite ORCA specific papers. It is not a substitute for doing your
own literature research and citing the relevant literature in a scientifically appropriate manner.

List of essential papers. We consider these as the minimum necessary citations

1. Neese, F.
   Software update: the ORCA program system, version 6.0
   WIRES Comput. Molec. Sci. 2025 15(1), e70019

doi.org/10.1002/wcms.70019

List of papers to cite with high priority. The work reported in these papers was absolutely necessary for this run to complete.
Our perspective: the developers of density functionals and basis sets usually get cited in chemistry papers
Good! But without the algorithms to do something with them, the functionals or basis sets would not do anything.
Hence, in our opinion, the algorithm design and method developments papers are equally worthy of getting cited

1. Page, M.; Doubleday, C.; McIver, J.
   Following steepest descent reaction paths. The use of higher energy derivatives with abinitio electronic structure methods
   J. Chem. Phys. 1990 93(8), 5634-5642
   doi.org/10.1063/1.459634
2. Neese, F.
   An improvement of the resolution of the identity approximation for the formation of the Coulomb matrix
   J. Comp. Chem. 2003 24(14), 1740-1747
   doi.org/10.1002/jcc.10318
3. Hratchian, H.; Schlegel, B.
   Using Hessian Updating To Increase the Efficiency of a Hessian Based Predictor-Corrector Reaction Path Following Method
   J. Chem. Theory Comput. 2005 1(1), 61-69
   doi.org/10.1021/ct0499783
4. Neese, F.; Wennmohs, F.; Hansen, A.; Becker, U.
   Efficient, approximate and parallel Hartree-Fock and hybrid DFT calculations. A 'chain-of-spheres' algorithm for the Hartree-Fock exchange
   Chem. Phys. 2009 356(1-3), 98-109
   doi.org/10.1016/j.chemphys.2008.10.036
5. Grimme, S.; Antony, J.; Ehrlich, S.; Krieg, H.
   A consistent and accurate ab initio parametrization of density functional dispersion correction (DFT-D) for the 94 elements H-Pu
   J. Chem. Phys. 2010 132 , 154104
   doi.org/10.1063/1.3382344
6. Grimme, S.; Ehrlich, S.; Goerigk, L.
   Effect of the damping function in dispersion corrected density functional theory
   J. Comput. Chem. 2011 32(7), 1456-1465
   doi.org/10.1002/jcc.21759
7. Garcia-Rates, M.; Neese, F.
   Effect of the Solute Cavity on the Solvation Energy and its Derivatives within the Framework of the Gaussian Charge Scheme
   J. Comput. Chem. 2020 41 , 922-939
   doi.org/10.1002/jcc.26139
8. Helmich-Paris, B.; de Souza, B.; Neese, F.; Izsák, R.
   An improved chain of spheres for exchange algorithm
   J. Chem. Phys. 2021 155(10), 104109
   doi.org/10.1063/5.0058766
9. Neese, F.
   The SHARK Integral Generation and Digestion System
   J. Comp. Chem. 2022 44(3), 381
   doi.org/10.1002/jcc.26942

List of suggested additional citations. These are papers that are important in the 'surrounding' of
of this run, or papers that preceded the highly important papers. If you like your results we are grateful for a citation.

1. Izsak, R.; Neese, F.
   An overlap fitted chain of spheres exchange method
   J. Chem. Phys. 2011 135 , 144105
   doi.org/10.1063/1.3646921

```
  2. Izsak, R.; Hansen, A.; Neese, F.
     The resolution of identity and chain of spheres approximations for the LPNO-CCSD singles
Fock term
     Molec. Phys. 2012 110 , 2413-2417
     doi.org/10.1080/00268976.2012.687466
  3. Neese, F.
     The ORCA program system
     WIRES Comput. Molec. Sci. 2012 2(1), 73-78
     doi.org/10.1002/wcms.81
  4. Izsak, R.; Neese, F.; Klopper, W.
     Robust fitting techniques in the chain of spheres approximation to the Fock exchange: The
role of the complementary space
     J. Chem. Phys. 2013 139 ,
     doi.org/10.1063/1.4819264
  5. Neese, F.
     Software update: the ORCA program system, version 4.0
     WIRES Comput. Molec. Sci. 2018 8(1), 1-6
     doi.org/10.1002/wcms.1327
  6. Neese, F.; Wennmohs, F.; Becker, U.; Riplinger, C.
     The ORCA quantum chemistry program package
     J. Chem. Phys. 2020 152(22), 224108
     doi.org/10.1063/5.0004608
  7. Neese, F.
     Software update: The ORCA program system—Version 5.0
     WIRES Comput. Molec. Sci. 2022 12(1), e1606
     doi.org/10.1002/wcms.1606

List of optional additional citations

  1. Neese, F.
     Approximate second-order SCF convergence for spin unrestricted wavefunctions
     Chem. Phys. Lett. 2000 325(1-3), 93-98
     doi.org/10.1016/s0009-2614(00)00662-x

Timings for individual modules:

Sum of individual times          ...   145399.373 sec (=2423.323 min)
Startup calculation              ...     5270.104 sec (=  87.835 min)   3.6 %
SCF iterations                   ...    85807.883 sec (=1430.131 min)  59.0 %
Property calculations            ...        0.448 sec (=   0.007 min)   0.0 %
SCF Gradient evaluation          ...    54320.939 sec (= 905.349 min)  37.4 %
                             ****ORCA TERMINATED NORMALLY****
TOTAL RUN TIME: 1 days 16 hours 44 minutes 39 seconds 49 msec
'LQA_TS-OMe.bibtex' -> '/hsnfs/users/barasits/Tunneling_selenoxide/OMe/IRC/LQA_TS-OMe.bibtex'
'LQA_TS-OMe.cpcm' -> '/hsnfs/users/barasits/Tunneling_selenoxide/OMe/IRC/LQA_TS-OMe.cpcm'
'LQA_TS-OMe.cpcm_corr' -> '/hsnfs/users/barasits/Tunneling_selenoxide/OMe/IRC/LQA_TS-
OMe.cpcm_corr'
'LQA_TS-OMe.densities' -> '/hsnfs/users/barasits/Tunneling_selenoxide/OMe/IRC/LQA_TS-
OMe.densities'
'LQA_TS-OMe.densitiesinfo' -> '/hsnfs/users/barasits/Tunneling_selenoxide/OMe/IRC/LQA_TS-
OMe.densitiesinfo'
'LQA_TS-OMe.engrad' -> '/hsnfs/users/barasits/Tunneling_selenoxide/OMe/IRC/LQA_TS-OMe.engrad'
'LQA_TS-OMe.err' -> '/hsnfs/users/barasits/Tunneling_selenoxide/OMe/IRC/LQA_TS-OMe.err'
'LQA_TS-OMe.gbw' -> '/hsnfs/users/barasits/Tunneling_selenoxide/OMe/IRC/LQA_TS-OMe.gbw'
'LQA_TS-OMe_IRC_B.cpcm' -> '/hsnfs/users/barasits/Tunneling_selenoxide/OMe/IRC/LQA_TS-
OMe_IRC_B.cpcm'
'LQA_TS-OMe_IRC_B.cpcm_corr' -> '/hsnfs/users/barasits/Tunneling_selenoxide/OMe/IRC/LQA_TS-
OMe_IRC_B.cpcm_corr'
'LQA_TS-OMe_IRC_B.densities' -> '/hsnfs/users/barasits/Tunneling_selenoxide/OMe/IRC/LQA_TS-
OMe_IRC_B.densities'
'LQA_TS-OMe_IRC_B.densitiesinfo' -> '/hsnfs/users/barasits/Tunneling_selenoxide/OMe/IRC/LQA_TS-
OMe_IRC_B.densitiesinfo'
```

```
'LQA_TS-OMe_IRC_B.gbw' -> '/hsnfs/users/barasits/Tunneling_selenoxide/OMe/IRC/LQA_TS-
OMe_IRC_B.gbw'
'LQA_TS-OMe_IRC_B.smd' -> '/hsnfs/users/barasits/Tunneling_selenoxide/OMe/IRC/LQA_TS-
OMe_IRC_B.smd'
'LQA_TS-OMe_IRC_B.smd.grd' -> '/hsnfs/users/barasits/Tunneling_selenoxide/OMe/IRC/LQA_TS-
OMe_IRC_B.smd.grd'
'LQA_TS-OMe_IRC_B_trj.xyz' -> '/hsnfs/users/barasits/Tunneling_selenoxide/OMe/IRC/LQA_TS-
OMe_IRC_B_trj.xyz'
'LQA_TS-OMe_IRC_B.xyz' -> '/hsnfs/users/barasits/Tunneling_selenoxide/OMe/IRC/LQA_TS-
OMe_IRC_B.xyz'
'LQA_TS-OMe_IRC_F.cpcm' -> '/hsnfs/users/barasits/Tunneling_selenoxide/OMe/IRC/LQA_TS-
OMe_IRC_F.cpcm'
'LQA_TS-OMe_IRC_F.cpcm_corr' -> '/hsnfs/users/barasits/Tunneling_selenoxide/OMe/IRC/LQA_TS-
OMe_IRC_F.cpcm_corr'
'LQA_TS-OMe_IRC_F.densities' -> '/hsnfs/users/barasits/Tunneling_selenoxide/OMe/IRC/LQA_TS-
OMe_IRC_F.densities'
'LQA_TS-OMe_IRC_F.densitiesinfo' -> '/hsnfs/users/barasits/Tunneling_selenoxide/OMe/IRC/LQA_TS-
OMe_IRC_F.densitiesinfo'
'LQA_TS-OMe_IRC_F.gbw' -> '/hsnfs/users/barasits/Tunneling_selenoxide/OMe/IRC/LQA_TS-
OMe_IRC_F.gbw'
'LQA_TS-OMe_IRC_F.smd' -> '/hsnfs/users/barasits/Tunneling_selenoxide/OMe/IRC/LQA_TS-
OMe_IRC_F.smd'
'LQA_TS-OMe_IRC_F.smd.grd' -> '/hsnfs/users/barasits/Tunneling_selenoxide/OMe/IRC/LQA_TS-
OMe_IRC_F.smd.grd'
'LQA_TS-OMe_IRC_F_trj.xyz' -> '/hsnfs/users/barasits/Tunneling_selenoxide/OMe/IRC/LQA_TS-
OMe_IRC_F_trj.xyz'
'LQA_TS-OMe_IRC_Full_trj.xyz' -> '/hsnfs/users/barasits/Tunneling_selenoxide/OMe/IRC/LQA_TS-
OMe_IRC_Full_trj.xyz'
'LQA_TS-OMe_IRC_F.xyz' -> '/hsnfs/users/barasits/Tunneling_selenoxide/OMe/IRC/LQA_TS-
OMe_IRC_F.xyz'
'LQA_TS-OMe.job' -> '/hsnfs/users/barasits/Tunneling_selenoxide/OMe/IRC/LQA_TS-OMe.job'
'LQA_TS-OMe.property.txt' -> '/hsnfs/users/barasits/Tunneling_selenoxide/OMe/IRC/LQA_TS-
OMe.property.txt'
'LQA_TS-OMe.smd' -> '/hsnfs/users/barasits/Tunneling_selenoxide/OMe/IRC/LQA_TS-OMe.smd'
'LQA_TS-OMe.smd.grd' -> '/hsnfs/users/barasits/Tunneling_selenoxide/OMe/IRC/LQA_TS-OMe.smd.grd'
'TS-OMe_Compound_2.hess' -> '/hsnfs/users/barasits/Tunneling_selenoxide/OMe/IRC/TS-
OMe_Compound_2.hess'
cp: cannot stat 'LQA_TS-OMe.asa.inp': No such file or directory
cp: missing destination file operand after
'/hsnfs/users/barasits/Tunneling_selenoxide/OMe/IRC/'
Try 'cp --help' for more information.
cp: cannot stat 'LQA_TS-OMe.pc.tmp': No such file or directory
cp: cannot stat 'LQA_TS-OMe.QM2.pc.tmp': No such file or directory
```

IRC output for R = Me

```
! Job execution by suborca V 10.06 !
Job started from head2.mpi-muelheim.mpg.de, running
/hsnfs/software/orca/orca_current_directory/orca_current_20251216/orca on xe30th96.mpi-
muelheim.mpg.de in /usr/scratch/barasits/Me-IRC__26969140 (/bin/bash)
Job execution start: Tue Dec 16 12:08:19 PM CET 2025
Shared library path: /hsnfs/software/openmpi-
4.1.0/lib:/hsnfs/software/orca/orca_current_directory/orca_current_20251216:/hsnfs/software/the
mis/llvm-14.0.1/lib:/hsnfs/software/orca/orca_stable:/usr/users/openmpi-
2.0.4/lib:/opt/intel/lib/intel64:/opt/intel/mkl/lib/intel64
SLURM Job ID is: 26969140
SLURM_JOB_NODELIST: xe30th96
Maxcore enforced by SLURM: 9600 MB per core
Expanded nodelist (1 nodes):
xe30th96

                                 *****************
                                 * O   R   C   A *
                                 *****************

                                            #,
                                            ###
                                            ####
                                            #####
                                            ######
                                           ########,
                                     ,,################,,,,,
                                ,,#################################,,
                           ,,##########################################,,
                        ,#########################################, ''#####,
                     ,#############################################,,   '####,
                   ,##################################################,,,,####,
                 ,###########''''           ''''###############################
               ,#####''   ,,,,##########,,,,          '''####'''          '####
             ,##' ,,,,###########################,,,                        '##
            ' ,,###''''                  '''############,,,
          ,,##''                                '''############,,,,        ,,,,,,###''
        ,#''                                            '''#######################'''
       '                                                          ''''####''''
             ,#######,   #######,   ,#######,      ##
            ,#'     '#,  ##    ##  ,#'     '#,    #''#        ,####,    ,#,
            ##       ##  ##   ,#'  ##            #'  '#       #'       ,# #
            ##       ##  #######   ##           ,######,      #####,      #
            '#,     ,#'  ##    ##  '#,     ,#' ,#      #,     #,   #      #
             '#######'   ##     ##  '#######'  #'      '#     '####' #    #


                  #######################################################
                  #                        -***-                        #
                  #          Department of theory and spectroscopy      #
                  #                                                     #
                  #                      Frank Neese                    #
                  #                                                     #
                  #     Directorship, Architecture, Infrastructure      #
                  #                    SHARK, DRIVERS                   #
                  #        Core code/Algorithms in most modules         #
                  #                                                     #
                  #        Max Planck Institute fuer Kohlenforschung    #
                  #                Kaiser Wilhelm Platz 1               #
                  #                 D-45470 Muelheim/Ruhr               #
                  #                      Germany                        #
                  #                                                     #
                  #                  All rights reserved                #
```

```
                  #                         -***-                          #
                  ##########################################################


                           Program Version 6.1.x  -  CURRENT   -
                                  (GIT: $2a5ddc4e$)
                  ($2025-12-15 18:08:28 +0100 $)

                    !                    WARNING:                          !
                    !         This is a DEVELOPMENT version                !

                           (BUILD: $Tue 16 Dec 2025 12:05:34 AM CET$)


 With contributions from (in alphabetic order):
[Max-Planck-Institut fuer Kohlenforschung]
  Daniel Aravena         : Magnetic Suceptibility
  Michael Atanasov       : Ab Initio Ligand Field Theory (pilot matlab implementation)
  Alexander A. Auer      : GIAO ZORA, VPT2 properties, NMR spectrum
  Ute Becker             : All parallelization in ORCA, NUMFREQ, NUMCALC
  Giovanni Bistoni       : ED, misc. LED, open-shell LED, HFLD
  Dmytro Bykov           : pre 5.0 version of the SCF Hessian
  Marcos Casanova-Páez   : Triplet and SCS-CIS(D). UHF-(DLPNO)-IP/EA/STEOM-CCSD. UHF-CVS-
IP/STEOM-CCSD
  Vijay G. Chilkuri      : MRCI spin determinant printing, contributions to CSF-ICE
  Pauline Colinet        : FMM embedding
  Dipayan Datta          : RHF DLPNO-CCSD density
  Achintya Kumar Dutta   : EOM-CC, STEOM-CC
  Nicolas Foglia         : Exact transition moments, OPA infrastructure, MCD improvements
  Dmitry Ganyushin       : Spin-Orbit,Spin-Spin,Magnetic field MRCI
  Miquel Garcia-Rates    : C-PCM and meta-GGA Hessian, CCSD/C-PCM, Gaussian charge scheme
  Tiago L. C. Gouveia    : GS-ROHF, GS-ROCIS
  Yang Guo               : DLPNO-NEVPT2, F12-NEVPT2, CIM, IAO-localization
  Andreas Hansen         : Spin unrestricted coupled pair/coupled cluster methods
  Ingolf Harden          : AUTO-CI MPn and infrastructure
  Benjamin Helmich-Paris : MC-RPA, TRAH-(SCF,CASSCF), AVAS, COSX integrals, SCF dyn. polar.,
MC-PDFT, srDFT
  Lee Huntington         : MR-EOM, pCC
  Robert Izsak           : Overlap fitted RIJCOSX, COSX-SCS-MP3, EOM
  Riya Kayal             : Wick's Theorem for AUTO-CI, AUTO-CI UHF-CCSDT
  Emily Kempfer          : AUTO-CI RHF CISDT and CCSDT, approximate NEVPT4
  Christian Kollmar      : KDIIS, OOCD, Brueckner-CCSD(T), CCSD density, CASPT2, CASPT2-K,
improved NEVPT2
  Axel Koslowski         : Symmetry handling
  Simone Kossmann        : meta-GGA functionals, TD-DFT gradient, OOMP2, (MP2 Hessian;
deprecated post 5.0)
  Lucas Lang             : DCDCAS, Hyperfine gauge corrections, ICE-SOC+SSC
  Marvin Lechner         : AUTO-CI (C++ implementation), FIC-MRCC
  Spencer Leger          : CASSCF response
  Dagmar Lenk            : GEPOL surface, SMD, ORCA-2-JSON
  Dimitrios Liakos       : Extrapolation schemes; Compound Job, Property file
  Dimitrios Manganas     : Further ROCIS development; embedding schemes. LFT, Crystal Embedding
  Hagen Neugebauer       : wr2SCAN, Native XTB
  Dimitrios Pantazis     : SARC Basis sets
  Anastasios Papadopoulos: AUTO-CI, single reference methods and gradients
  Taras Petrenko         : pre 6.0 DFT Hessian and TD-DFT gradient,  ECA, NRVS
  Petra Pikulova         : Analytic Raman intensities
  Peter Pinski           : DLPNO-MP2, DLPNO-MP2 Gradient
  Shashank Vittal Rao    : ES-AILFT, MagRelax
  Christoph Reimann      : Effective Core Potentials
  Marius Retegan         : Local ZFS, SOC
  Christoph Riplinger    : Optimizer, TS searches, QM/MM, DLPNO-CCSD(T), (RO)-DLPNO pert.
Triples
```

```
  Michael Roemelt        : Original ROCIS implementation, recursive CI coupling coefficients
  Masaaki Saitow         : Open-shell DLPNO-CCSD energy and density
  Barbara Sandhoefer     : DKH picture change effects
  Yorick L. A. Schmerwitz: GMF and freeze-and-release deltaSCF, NEB S-IDPP initial path
  Kantharuban Sivalingam : CASSCF convergence/infrastructure, NEVPT2, NEVPT3, NEVPT4(SD), FIC-
MRCI and CEPA variants
  Bernardo de Souza      : ESD, SOC TD-DFT
  Georgi L. Stoychev     : AutoAux, RI-MP2 NMR, DLPNO-MP2 response, X2C
  Van Anh Tran           : RI-MP2 g-tensors
  Willem Van den Heuvel  : Paramagnetic NMR
  Zikuan Wang            : NOTCH, Electric field optimization
  Frank Wennmohs         : Technical directorship and infrastructure
  Hang Xu                : AUTO-CI-Response properties

[FACCTs GmbH]
  Markus Bursch, Nicolas Foglia, Miquel Garcia-Rates, Ingolf Harden, Hagen Neugebauer,
Anastasios Papadopoulos,
  Christoph Riplinger, Bernardo de Souza, Georgi L. Stoychev

  APM, various basis sets, CI-OPT, improved COSX, DLPNO-Multilevel,
  DOCKER, DRACO, updates on ESD, Fragmentator, GOAT, IRC, LR-CPCM, L-BFGS, MBIS, meta-GGA TD-
DFT gradient, ML-optimized integration grids,
  MM, NACMEs, nearIR, NEB, NEB-TS, NL-DFT gradient (VV10), 2- and 3-layer-ONIOM, interface
openCOSMO-RS, QMMM,
  Crystal-QMMM, RESP, rigid body optimization, SF, symmetry and pop. for TD-DFT, various
functionals, SOLVATOR

[Other institutions]
  V. Asgeirsson          : NEB
  Christoph Bannwarth    : sTDA-DFT, sTD-DFT, PBEh-3c, B97-3c, D3
  Giovanni Bistoni       : ETS/NOCV, ADLD/ADEX, COVALED
  Martin Brehm           : Molecular dynamics
  Ronald Cardenas        : ETS/NOCV
  Martina Colucci        : COVALED
  Sebastian Ehlert       : rSCAN, r2SCAN, r2SCAN-3c, D4, dhf basis sets
  Marvin Friede          : D4 for Fr, Ra, Ac-Lr
  Lars Goerigk           : TD-DFT with DH, B97 family of functionals
  Stefan Grimme          : VdW corrections, initial TS optimization, DFT functionals, gCP,
sTDA/sTD-DF
  Waldemar Hujo          : DFT-NL
  H. Jonsson             : NEB
  Holger Kruse           : gCP
  Marcel Mueller         : wB97X-3c, vDZP basis set
  Gianluca Regni         : ADLD/ADEX
  Tobias Risthaus        : pre 6.0 range-separated hybrid DFT and stability analysis
  Lukas Wittmann         : regularized MP2, r2SCAN double-hybrids, wr2SCAN

We gratefully acknowledge several colleagues who have allowed us to
interface, adapt or use parts of their codes:
  Ed Valeev, F. Pavosevic, A. Kumar             : LibInt (2-el integral package), F12 methods
  Garnet Chan, S. Sharma, J. Yang, R. Olivares  : DMRG
  Ulf Ekstrom                                   : XCFun DFT Library
  Mihaly Kallay                                 : mrcc  (arbitrary order and MRCC methods)
  Frank Weinhold                                : gennbo (NPA and NBO analysis)
  Simon Mueller                                 : openCOSMO-RS
  Christopher J. Cramer and Donald G. Truhlar   : smd solvation model
  S Lehtola, MJT Oliveira, MAL Marques          : LibXC Library
  Liviu Ungur et al                             : ANISO software


 Your calculation uses the libint2 library for the computation of 2-el integrals
 For citations please refer to: http://libint.valeyev.net
```

```
 Your ORCA version has been built with support for libXC version: 7.0.0
 For citations please refer to: https://libxc.gitlab.io

 This ORCA versions uses:
   CBLAS   interface :  Fast vector & matrix operations
   LAPACKE interface :  Fast linear algebra routines
   SCALAPACK package :  Parallel linear algebra routines
   Shared memory     :  Shared parallel matrices
   BLAS/LAPACK       :  OpenBLAS 0.3.26  USE64BITINT DYNAMIC_ARCH NO_AFFINITY Haswell
SINGLE_THREADED
        Core in use  :  Haswell
   Copyright (c) 2011-2014, The OpenBLAS Project


  ***********************************
  * Starting time: Tue Dec 16 12:08:19 2025
  * Host name:     xe30th96.mpi-muelheim.mpg.de
  * Process ID:    4172949
  * Working dir.:  /usr/scratch/barasits/Me-IRC__26969140
  ***********************************

NOTE: MaxCore=8000 MB was set to SCF,MP2,MDCI,CIPSI,MRCI,RASCI and CIS
      => If you want to overwrite this, your respective input block should be placed after the
MaxCore statement
Your calculation utilizes the atom-pairwise dispersion correction
with the Becke-Johnson damping scheme (D3BJ)


Your calculation utilizes the SMD solvation module
Please cite in your paper:
Marenich, A. V.; Cramer, C. J.; Truhlar, D. G. J. Phys. Chem. B 2009, 113, 6378

================================================================================

----- Orbital basis set information -----
Your calculation utilizes the basis: def2-TZVP
   F. Weigend and R. Ahlrichs, Phys. Chem. Chem. Phys. 7, 3297 (2005).

----- AuxJ basis set information -----
Your calculation utilizes the auxiliary basis: def2/J
   H-Rn: F. Weigend, Phys. Chem. Chem. Phys. 8, 1057 (2006).
   Fr-Lr: K. Eichkorn, F. Weigend, O. Treutler, R. Ahlrichs; Theor. Chem. Acc. 97, 119 (1997).

================================================================================
                                        WARNINGS
                       Please study these warnings very carefully!
================================================================================

WARNING: SinglePoint found together with IRC
         Switching on EnGrad - energy plus gradient calculation

================================================================================
                                       INPUT FILE
================================================================================
NAME = Me-IRC.inp
|  1> #
|  2> #IRC LQA
|  3> #
|  4> ! irc B3LYP D3BJ def2-TZVP def2/J verytightscf
|  5> %pal nprocs 12 end
|  6> %maxcore 8000
|  7> %irc
|  8> printlevel 1
```

```
|  9> method lqa
| 10> maxiter 241
| 11> tolrmsg 1.e-5 # Tighter convergence criteria
| 12> tolmaxg 8.e-5 # Tighter convergence criteria
| 13> follow_coordtype massweighted
| 14> inithess read
| 15> hess_filename "TS-Me_Compound_2.hess"
| 16> lqahess lqacalc_bofillupdate
| 17> init_displ length
| 18> scale_init_displ 0.04
| 19> adapt_scale_displ false
| 20> scale_displ_sd 0.11
| 21> ENFORCESTRICTCONVERGENCE true
| 22> REPORTPROFILE true
| 23> BSCORRECTOR true
| 24> BSRICHTHRESH 1E-6
| 25> INITBSCORRECTOR false
| 26> end
| 27> %CPCM SMD true
| 28>      SMDSOLVENT "TETRAHYDROFURAN" END
| 29> 
| 30> * xyz 0 1
| 31>   C         -1.81283034501624      0.40036353131771     -6.09065807280438
| 32>   C         -1.36913824802058     -0.31916294778936     -4.98031895066750
| 33>   C         -0.95114289260033      1.34209917758946     -6.65708547552200
| 34>   C         -0.10557955144969     -0.09868071402935     -4.44469042752773
| 35>   C          0.31696476094881      1.56160869042788     -6.12962145613200
| 36>   C          0.74343371443124      0.84146808101715     -5.01860089173867
| 37>   H         -2.02038133629528     -1.06107676836100     -4.53443308194206
| 38>   H         -1.28152482652021      1.91180205925317     -7.51813068079703
| 39>   H          0.21964802189795     -0.66700753600396     -3.58280617466352
| 40>   H          0.97023616252612      2.29628570717498     -6.58462757069959
| 41>   H          1.73045566962436      1.00832175145658     -4.60567204001317
| 42>   C         -3.17984713610389      0.16011594237349     -6.67314808496554
| 43>   H         -3.54290640533545      1.09399379540067     -7.11314148420678
| 44>   H         -3.87363245200985     -0.09635728965787     -5.86931662036954
| 45>   C         -3.24964759745324     -0.93916418569348     -7.74436987915005
| 46>   H         -3.15849886251497     -2.07933229749626     -7.09565663322275
| 47>   H         -4.24986601774073     -1.04516708225485     -8.16605252651890
| 48>   C         -2.20738604701034     -1.13240396942470     -8.67025859956160
| 49>   H         -2.42448387325747     -1.45742059259920     -9.67841840083874
| 50>   H         -1.24124454856501     -0.66379338748976     -8.53507795830517
| 51>   Se        -1.48792043753358     -3.44781291274028     -7.94141321574191
| 52>   O         -2.75249794501672     -3.28711325219179     -6.78708021541796
| 53>   C          0.09151608114451     -2.99807628351897     -6.91958134422662
| 54>   C          0.26522549253531     -3.51765610314150     -5.63926781031102
| 55>   C          1.09026638876560     -2.22606992428159     -7.49958190698987
| 56>   C          1.43424126525941     -3.24945023496838     -4.94330921253935
| 57>   C          2.26121802443337     -1.97009554379583     -6.79300322057610
| 58>   C          2.45142936870627     -2.47283481270712     -5.50697463914098
| 59>   H         -0.51307043924443     -4.11874933654515     -5.18730163508738
| 60>   H          0.95770448939781     -1.81302154102349     -8.49181335997033
| 61>   H          1.56073936222195     -3.64788127404618     -3.94294522103732
| 62>   H          3.03090960717658     -1.35588574575990     -7.24513434923701
| 63>   C          3.69900651846576     -2.16629911989596     -4.72805397478829
| 64>   H          3.47236699990727     -1.53938660241269     -3.86077982218942
| 65>   H          4.16505768749041     -3.07943679364883     -4.35004023329163
| 66>   H          4.42966439675532     -1.63853545453357     -5.34154835980777
| 67> *
| 68> 
| 69> 
| 70>                          ****END OF INPUT****
================================================================================
```

```
                     ******************************
                     * Energy+Gradient Calculation *
                     ******************************

---------------------------------
CARTESIAN COORDINATES (ANGSTROEM)
---------------------------------
  C     -1.812830    0.400364   -6.090658
  C     -1.369138   -0.319163   -4.980319
  C     -0.951143    1.342099   -6.657085
  C     -0.105580   -0.098681   -4.444690
  C      0.316965    1.561609   -6.129621
  C      0.743434    0.841468   -5.018601
  H     -2.020381   -1.061077   -4.534433
  H     -1.281525    1.911802   -7.518131
  H      0.219648   -0.667008   -3.582806
  H      0.970236    2.296286   -6.584628
  H      1.730456    1.008322   -4.605672
  C     -3.179847    0.160116   -6.673148
  H     -3.542906    1.093994   -7.113141
  H     -3.873632   -0.096357   -5.869317
  C     -3.249648   -0.939164   -7.744370
  H     -3.158499   -2.079332   -7.095657
  H     -4.249866   -1.045167   -8.166053
  C     -2.207386   -1.132404   -8.670259
  H     -2.424484   -1.457421   -9.678418
  H     -1.241245   -0.663793   -8.535078
  Se    -1.487920   -3.447813   -7.941413
  O     -2.752498   -3.287113   -6.787080
  C      0.091516   -2.998076   -6.919581
  C      0.265225   -3.517656   -5.639268
  C      1.090266   -2.226070   -7.499582
  C      1.434241   -3.249450   -4.943309
  C      2.261218   -1.970096   -6.793003
  C      2.451429   -2.472835   -5.506975
  H     -0.513070   -4.118749   -5.187302
  H      0.957704   -1.813022   -8.491813
  H      1.560739   -3.647881   -3.942945
  H      3.030910   -1.355886   -7.245134
  C      3.699007   -2.166299   -4.728054
  H      3.472367   -1.539387   -3.860780
  H      4.165058   -3.079437   -4.350040
  H      4.429664   -1.638535   -5.341548

----------------------------
CARTESIAN COORDINATES (A.U.)
----------------------------
  NO LB      ZA    FRAG     MASS         X           Y           Z
   0 C     6.0000    0    12.011   -3.425753    0.756577  -11.509676
   1 C     6.0000    0    12.011   -2.587296   -0.603131   -9.411439
   2 C     6.0000    0    12.011   -1.797400    2.536200  -12.580068
   3 C     6.0000    0    12.011   -0.199516   -0.186480   -8.399248
   4 C     6.0000    0    12.011    0.598977    2.951013  -11.583306
   5 C     6.0000    0    12.011    1.404886    1.590144   -9.483781
   6 H     1.0000    0     1.008   -3.817967   -2.005144   -8.568837
   7 H     1.0000    0     1.008   -2.421731    3.612782  -14.207208
   8 H     1.0000    0     1.008    0.415075   -1.260462   -6.770522
   9 H     1.0000    0     1.008    1.833481    4.339351  -12.443143
  10 H     1.0000    0     1.008    3.270087    1.905452   -8.703459
  11 C     6.0000    0    12.011   -6.009040    0.302575  -12.610422
  12 H     1.0000    0     1.008   -6.695123    2.067349  -13.441889
  13 H     1.0000    0     1.008   -7.320104   -0.182089  -11.091401
```

```
  14 C     6.0000    0    12.011   -6.140944   -1.774763  -14.634738
  15 H     1.0000    0     1.008   -5.968698   -3.929369  -13.408848
  16 H     1.0000    0     1.008   -8.031083   -1.975080  -15.431603
  17 C     6.0000    0    12.011   -4.171355   -2.139933  -16.384414
  18 H     1.0000    0     1.008   -4.581611   -2.754126  -18.289560
  19 H     1.0000    0     1.008   -2.345612   -1.254388  -16.128960
  20 Se   34.0000    0    78.960   -2.811762   -6.515422  -15.007096
  21 O     8.0000    0    15.999   -5.201467   -6.211744  -12.825723
  22 C     6.0000    0    12.011    0.172940   -5.665543  -13.076114
  23 C     6.0000    0    12.011    0.501204   -6.647407  -10.656672
  24 C     6.0000    0    12.011    2.060305   -4.206663  -14.172156
  25 C     6.0000    0    12.011    2.710323   -6.140571   -9.341501
  26 C     6.0000    0    12.011    4.273083   -3.722941  -12.836916
  27 C     6.0000    0    12.011    4.632530   -4.672981  -10.406674
  28 H     1.0000    0     1.008   -0.969563   -7.783308   -9.802579
  29 H     1.0000    0     1.008    1.809799   -3.426114  -16.047202
  30 H     1.0000    0     1.008    2.949370   -6.893497   -7.451087
  31 H     1.0000    0     1.008    5.727589   -2.562253  -13.691320
  32 C     6.0000    0    12.011    6.990109   -4.093712   -8.934727
  33 H     1.0000    0     1.008    6.561823   -2.909019   -7.295817
  34 H     1.0000    0     1.008    7.870818   -5.819292   -8.220385
  35 H     1.0000    0     1.008    8.370853   -3.096383  -10.094064

--------------------------------
INTERNAL COORDINATES (ANGSTROEM)
--------------------------------
 C      0   0   0     0.000000000000     0.00000000     0.00000000
 C      1   0   0     1.395504925545     0.00000000     0.00000000
 C      1   2   0     1.396499658310   118.30977481     0.00000000
 C      2   1   3     1.389996730170   120.91987341     0.44814273
 C      3   1   2     1.390862947586   121.00975498   359.90332347
 C      4   2   1     1.390710790711   120.20966638   359.50152397
 H      2   1   3     1.083221069362   119.30823651   180.63581139
 H      3   1   2     1.084026033434   119.24339766   180.19163197
 H      4   2   1     1.082410650528   119.78100569   179.97572101
 H      5   3   1     1.083302546544   119.83876435   179.94831066
 H      6   4   2     1.082849354232   120.21606268   180.43326065
 C      1   2   3     1.505240321469   120.95411876   179.41790958
 H     12   1   2     1.094318997699   108.70440903   149.57875300
 H     12   1   2     1.092365143526   109.23923523    33.63851064
 C     12   1   2     1.536491144362   115.16595547   271.36188571
 H     15  12   1     1.314960185897   105.85790343    74.56836800
 H     15  12   1     1.090637317101   112.38068959   182.51524042
 C     15  12   1     1.407451837945   121.54922957   323.31671738
 H     18  15  12     1.081273996919   120.39451096   213.98721022
 H     18  15  12     1.082265715197   121.29581946    13.11015174
 Se    16  15  12     2.319240320706   112.43580814   232.65256620
 O     16  15  12     1.311026691246   159.45102558   228.19549334
 C     21  16  15     1.934172465382   104.97200058    91.09234667
 C     23  21  16     1.392602202243   120.05374882    79.34183646
 C     23  21  16     1.389207201422   119.71763798   255.21282883
 C     24  23  21     1.386683321603   119.63104417   176.45975602
 C     25  23  21     1.391367692823   119.68686399   183.84708531
 C     27  25  23     1.393842401516   121.13391526   359.84117856
 H     24  23  21     1.082280488697   120.09591093   355.82975050
 H     25  23  21     1.082914992213   120.43094694     4.60388229
 H     26  24  23     1.084194252397   119.36445202   179.39026854
 H     27  25  23     1.083559581220   119.35317175   180.84296727
 C     28  27  25     1.502374878958   121.20394224   181.69264706
 H     33  28  27     1.093868928033   110.85130140   112.98514392
 H     33  28  27     1.092665773298   111.28716311   231.99323355
 H     33  28  27     1.090307654763   111.31663378   352.85908610
```

```
---------------------------
INTERNAL COORDINATES (A.U.)
---------------------------
 C      0   0   0     0.000000000000     0.00000000     0.00000000
 C      1   0   0     2.637122127818     0.00000000     0.00000000
 C      1   2   0     2.639001900320   118.30977481     0.00000000
 C      2   1   3     2.626713147068   120.91987341     0.44814273
 C      3   1   2     2.628350060756   121.00975498   359.90332347
 C      4   2   1     2.628062525933   120.20966638   359.50152397
 H      2   1   3     2.046991163588   119.30823651   180.63581139
 H      3   1   2     2.048512325232   119.24339766   180.19163197
 H      4   2   1     2.045459693938   119.78100569   179.97572101
 H      5   3   1     2.047145133148   119.83876435   179.94831066
 H      6   4   2     2.046288723791   120.21606268   180.43326065
 C      1   2   3     2.844491973312   120.95411876   179.41790958
 H     12   1   2     2.067963208799   108.70440903   149.57875300
 H     12   1   2     2.064270959505   109.23923523    33.63851064
 C     12   1   2     2.903547470040   115.16595547   271.36188571
 H     15  12   1     2.484914628356   105.85790343    74.56836800
 H     15  12   1     2.061005840756   112.38068959   182.51524042
 C     15  12   1     2.659698520399   121.54922957   323.31671738
 H     18  15  12     2.043311729906   120.39451096   213.98721022
 H     18  15  12     2.045185805854   121.29581946    13.11015174
 Se    16  15  12     4.382729044881   112.43580814   232.65256620
 O     16  15  12     2.477481400716   159.45102558   228.19549334
 C     21  16  15     3.655056255343   104.97200058    91.09234667
 C     23  21  16     2.631636775735   120.05374882    79.34183646
 C     23  21  16     2.625221153958   119.71763798   255.21282883
 C     24  23  21     2.620451712306   119.63104417   176.45975602
 C     25  23  21     2.629303891022   119.68686399   183.84708531
 C     27  25  23     2.633980412712   121.13391526   359.84117856
 H     24  23  21     2.045213723724   120.09591093   355.82975050
 H     25  23  21     2.046412761600   120.43094694     4.60388229
 H     26  24  23     2.048830213001   119.36445202   179.39026854
 H     27  25  23     2.047630858292   119.35317175   180.84296727
 C     28  27  25     2.839077071714   121.20394224   181.69264706
 H     33  28  27     2.067112700388   110.85130140   112.98514392
 H     33  28  27     2.064839067442   111.28716311   231.99323355
 H     33  28  27     2.060382869219   111.31663378   352.85908610

---------------------
BASIS SET INFORMATION
---------------------
There are 4 groups of distinct atoms

 Group   1 Type C   : 11s6p2d1f contracted to 5s3p2d1f pattern {62111/411/11/1}
 Group   2 Type H   : 5s1p contracted to 3s1p pattern {311/1}
 Group   3 Type Se  : 17s13p8d1f contracted to 6s5p4d1f pattern {842111/64111/5111/1}
 Group   4 Type O   : 11s6p2d1f contracted to 5s3p2d1f pattern {62111/411/11/1}

Atom   0C    basis set group =>   1
Atom   1C    basis set group =>   1
Atom   2C    basis set group =>   1
Atom   3C    basis set group =>   1
Atom   4C    basis set group =>   1
Atom   5C    basis set group =>   1
Atom   6H    basis set group =>   2
Atom   7H    basis set group =>   2
Atom   8H    basis set group =>   2
Atom   9H    basis set group =>   2
Atom  10H    basis set group =>   2
Atom  11C    basis set group =>   1
Atom  12H    basis set group =>   2
```

```
Atom  13H    basis set group =>   2
Atom  14C    basis set group =>   1
Atom  15H    basis set group =>   2
Atom  16H    basis set group =>   2
Atom  17C    basis set group =>   1
Atom  18H    basis set group =>   2
Atom  19H    basis set group =>   2
Atom  20Se   basis set group =>   3
Atom  21O    basis set group =>   4
Atom  22C    basis set group =>   1
Atom  23C    basis set group =>   1
Atom  24C    basis set group =>   1
Atom  25C    basis set group =>   1
Atom  26C    basis set group =>   1
Atom  27C    basis set group =>   1
Atom  28H    basis set group =>   2
Atom  29H    basis set group =>   2
Atom  30H    basis set group =>   2
Atom  31H    basis set group =>   2
Atom  32C    basis set group =>   1
Atom  33H    basis set group =>   2
Atom  34H    basis set group =>   2
Atom  35H    basis set group =>   2
---------------------------------
AUXILIARY/J BASIS SET INFORMATION
---------------------------------
There are 4 groups of distinct atoms

 Group   1 Type C   : 12s5p4d2f1g contracted to 6s4p3d1f1g pattern {711111/2111/211/2/1}
 Group   2 Type H   : 5s2p1d contracted to 3s1p1d pattern {311/2/1}
 Group   3 Type Se  : 19s5p5d3f1g contracted to 8s4p3d2f1g pattern {121111111/2111/311/21/1}
 Group   4 Type O   : 12s5p4d2f1g contracted to 6s4p3d1f1g pattern {711111/2111/211/2/1}

Atom   0C    basis set group =>   1
Atom   1C    basis set group =>   1
Atom   2C    basis set group =>   1
Atom   3C    basis set group =>   1
Atom   4C    basis set group =>   1
Atom   5C    basis set group =>   1
Atom   6H    basis set group =>   2
Atom   7H    basis set group =>   2
Atom   8H    basis set group =>   2
Atom   9H    basis set group =>   2
Atom  10H    basis set group =>   2
Atom  11C    basis set group =>   1
Atom  12H    basis set group =>   2
Atom  13H    basis set group =>   2
Atom  14C    basis set group =>   1
Atom  15H    basis set group =>   2
Atom  16H    basis set group =>   2
Atom  17C    basis set group =>   1
Atom  18H    basis set group =>   2
Atom  19H    basis set group =>   2
Atom  20Se   basis set group =>   3
Atom  21O    basis set group =>   4
Atom  22C    basis set group =>   1
Atom  23C    basis set group =>   1
Atom  24C    basis set group =>   1
Atom  25C    basis set group =>   1
Atom  26C    basis set group =>   1
Atom  27C    basis set group =>   1
Atom  28H    basis set group =>   2
Atom  29H    basis set group =>   2
```

```
Atom  30H    basis set group =>   2
Atom  31H    basis set group =>   2
Atom  32C    basis set group =>   1
Atom  33H    basis set group =>   2
Atom  34H    basis set group =>   2
Atom  35H    basis set group =>   2


           ************************************************************
           *        Program running with 12 parallel MPI-processes    *
           *              working on a common directory               *
           ************************************************************
------------------------------------------------------------------------------
                            ORCA STARTUP CALCULATIONS
                          -- RI-GTO INTEGRALS CHOSEN --
------------------------------------------------------------------------------
------------------------------------------------------------------------------
                   ___
                  /   \      - P O W E R E D   B Y -
                 /     \
                 |  |  |   _    _      __       _____    __    __
                 |  |  |  | |  | |    /  \     |  _  \  |  |  /  |
                  \  \/   | |  | |   /    \    | | | |  |  | /  /
                 / \  \   | |__| |  /  /\  \   | |_| |  |  |/  /
                |  |  |   |  __  | /  /__\  \  |    /   |      \
                |  |  |   | |  | | |   __   |  |    \   |  |\   \
                \     /   | |  | | |  |  |  |  | |\  \  |  |  \  \
                 \___/    |_|  |_| |__|  |__|  |_| \__\ |__|   \__/

                      - O R C A' S   B I G   F R I E N D -
                                      &
                       - I N T E G R A L   F E E D E R -

 v1 FN, 2020, v2 2021, v3 2022-2024
------------------------------------------------------------------------------


----------------------
SHARK INTEGRAL PACKAGE
----------------------

Number of atoms                             ...     36
Number of basis functions                   ...    683
Number of shells                            ...    275
Maximum angular momentum                    ...      3
Integral batch strategy                     ... SHARK/LIBINT Hybrid
RI-J (if used) integral strategy            ... SPLIT-RIJ (Revised 2003 algorithm where
possible)
Printlevel                                  ...      1
Contraction scheme used                     ... SEGMENTED contraction
Prescreening option                         ... SCHWARTZ
   Thresh                                   ... 1.000e-12
   Tcut                                     ... 1.000e-14
   Tpresel                                  ... 1.000e-14
Coulomb Range Separation                    ... NOT USED
Exchange Range Separation                   ... NOT USED
Multipole approximations                    ... NOT USED
Finite Nucleus Model                        ... NOT USED
CABS basis                                  ... NOT available
Auxiliary Coulomb fitting basis             ... AVAILABLE
   # of basis functions in Aux-J            ...   1089
   # of shells in Aux-J                     ...    363
   Maximum angular momentum in Aux-J        ...      4
```

```
Auxiliary J/K fitting basis                 ... NOT available
Auxiliary Correlation fitting basis         ... NOT available
Auxiliary 'external' fitting basis          ... NOT available

Checking pre-screening integrals            ... done (  0.0 sec) Dimension = 275
   => SHARK Basis and OBASIS are compatible. Storing Pre-screening
Shell pair information
Shell pair cut-off parameter TPreSel        ...   1.0e-14
Total number of shell pairs                 ...     37950
Shell pairs after pre-screening             ...     31721
Total number of primitive shell pairs       ...    119322
Primitive shell pairs kept                  ...     67656
          la=0 lb=0:   8474 shell pairs
          la=1 lb=0:   9058 shell pairs
          la=1 lb=1:   2473 shell pairs
          la=2 lb=0:   4578 shell pairs
          la=2 lb=1:   2488 shell pairs
          la=2 lb=2:    662 shell pairs
          la=3 lb=0:   2074 shell pairs
          la=3 lb=1:   1163 shell pairs
          la=3 lb=2:    599 shell pairs
          la=3 lb=3:    152 shell pairs

Checking whether 4 symmetric matrices of dimension 683 fit in memory:
Max Core in MB      =   8000.00
 MB in use           =     42.42
 MB left             =   7957.58
 MB needed           =      7.13
 Data fit in memory  = YES
Calculating RI/J V-Matrix + Cholesky decomp.... done (  0.1 sec)
Calculating Nuclear repulsion               ... done (  0.0 sec) ENN=   1761.151007088133 Eh

Diagonalization of the overlap matrix:
Smallest eigenvalue                        ... 5.895e-06
Time for diagonalization                   ...    0.082 sec
Threshold for overlap eigenvalues          ... 1.000e-07
Number of eigenvalues below threshold      ... 0
Time for construction of square roots      ...    0.212 sec
Total time needed                          ...    0.299 sec

-------------------
DFT GRID GENERATION
-------------------

General Integration Accuracy     IntAcc      ... 4.388
Radial Grid Type                 RadialGrid  ... OptM3 with GC (2021)
Angular Grid (max. ang.)         AngularGrid ... 4 (Lebedev-302)
Angular grid pruning method      GridPruning ... 4 (adaptive)
Weight generation scheme         WeightScheme... mBecke (2022)
Basis function cutoff            BFCut       ... 1.0000e-12
Integration weight cutoff        WCut        ... 1.0000e-14
Partially contracted basis set               ... off
Rotationally invariant grid construction     ... off
Angular grids for H and He will be reduced by one unit
Core-polarized basis detected: some atoms will have their core
                               angular grid increased.

Total number of grid points                  ...   169668
Total number of batches                      ...     2671
Average number of points per batch           ...       63
Average number of grid points per atom       ...     4713

-------------------
```

```
COSX GRID GENERATION
--------------------

GRIDX 1
-------
General Integration Accuracy     IntAcc      ... 3.816
Radial Grid Type                 RadialGrid  ... OptM3 with GC (2021)
Angular Grid (max. ang.)         AngularGrid ... 1 (Lebedev-50)
Angular grid pruning method      GridPruning ... 4 (adaptive)
Weight generation scheme         WeightScheme... mBecke (2022)
Basis function cutoff            BFCut       ... 1.0000e-12
Integration weight cutoff        WCut        ... 1.0000e-14
Partially contracted basis set               ... on
Rotationally invariant grid construction     ... off
Angular grids for H and He will be reduced by one unit
Core-polarized basis detected: some atoms will have their core
                               angular grid increased.

Total number of grid points                  ...    26073
Total number of batches                      ...      222
Average number of points per batch           ...      117
Average number of grid points per atom       ...      724
UseSFitting                                  ... on

GRIDX 2
-------
General Integration Accuracy     IntAcc      ... 4.020
Radial Grid Type                 RadialGrid  ... OptM3 with GC (2021)
Angular Grid (max. ang.)         AngularGrid ... 2 (Lebedev-110)
Angular grid pruning method      GridPruning ... 4 (adaptive)
Weight generation scheme         WeightScheme... mBecke (2022)
Basis function cutoff            BFCut       ... 1.0000e-12
Integration weight cutoff        WCut        ... 1.0000e-14
Partially contracted basis set               ... on
Rotationally invariant grid construction     ... off
Angular grids for H and He will be reduced by one unit
Core-polarized basis detected: some atoms will have their core
                               angular grid increased.

Total number of grid points                  ...    48854
Total number of batches                      ...      403
Average number of points per batch           ...      121
Average number of grid points per atom       ...     1357
UseSFitting                                  ... on

GRIDX 3
-------
General Integration Accuracy     IntAcc      ... 4.338
Radial Grid Type                 RadialGrid  ... OptM3 with GC (2021)
Angular Grid (max. ang.)         AngularGrid ... 3 (Lebedev-194)
Angular grid pruning method      GridPruning ... 4 (adaptive)
Weight generation scheme         WeightScheme... mBecke (2022)
Basis function cutoff            BFCut       ... 1.0000e-12
Integration weight cutoff        WCut        ... 1.0000e-14
Partially contracted basis set               ... on
Rotationally invariant grid construction     ... off
Angular grids for H and He will be reduced by one unit
Core-polarized basis detected: some atoms will have their core
                               angular grid increased.

Total number of grid points                  ...    99401
Total number of batches                      ...      794
Average number of points per batch           ...      125
```

```
Average number of grid points per atom       ...     2761
UseSFitting                                  ... on
Grids setup in   2.7 sec
Initializing property integral containers    ... done (  0.0 sec)

SHARK setup successfully completed in   3.7 seconds

Maximum memory used throughout the entire STARTUP-calculation: 83.4 MB


           ************************************************************
           *        Program running with 12 parallel MPI-processes    *
           *              working on a common directory               *
           ************************************************************
--------------------------------------------------------------------------------
                                 ORCA GUESS
                   Start orbitals & Density for SCF / CASSCF
--------------------------------------------------------------------------------


------------
SCF SETTINGS
------------
Hamiltonian:
 Density Functional     Method          .... DFT(GTOs)
 Exchange Functional    Exchange        .... B88
   X-Alpha parameter    XAlpha          ....  0.666667
   Becke's b parameter  XBeta           ....  0.004200
 Correlation Functional Correlation     .... LYP
 LDA part of GGA corr.  LDAOpt          .... VWN-5
 Gradients option       PostSCFGGA      .... off
 Hybrid DFT is turned on
   Fraction HF Exchange ScalHFX         ....  0.200000
   Scaling of DF-GGA-X  ScalDFX         ....  0.720000
   Scaling of DF-GGA-C  ScalDFC         ....  0.810000
   Scaling of DF-LDA-C  ScalLDAC        ....  1.000000
   Perturbative correction              ....  0.000000
   NL short-range parameter             ....  4.800000
 RI-approximation to the Coulomb term is turned on
   Number of AuxJ basis functions       .... 1089
   RIJ-COSX (HFX calculated with COS-X)).... on


General Settings:
 Integral files         IntName         .... Me-IRC
 Hartree-Fock type      HFTyp           .... RHF
 Total Charge           Charge          ....    0
 Multiplicity           Mult            ....    1
 Number of Electrons    NEL             ....  156
 Basis Dimension        Dim             ....  683
 Nuclear Repulsion      ENuc            ....   1761.1510070881 Eh

Convergence Acceleration:
 AO-DIIS                CNVDIIS         .... on
   Start iteration      DIISMaxIt       ....    12
   Startup error        DIISStart       ....  0.200000
   # of expansion vecs  DIISMaxEq       ....     5
   Bias factor          DIISBfac        ....   1.050
   Max. coefficient     DIISMaxC        ....  10.000
 MO-DIIS                CNVKDIIS        .... off
 Trust-Rad. Augm. Hess. CNVTRAH         .... auto
   Auto Start mean grad. ratio tolernc. ....  1.125000
   Auto Start start iteration           ....    50
   Auto Start num. interpolation iter.  ....    10
```

```
   Max. Number of Micro iterations      ....    24
   Max. Number of Macro iterations      .... Maxiter - #DIIS iter
   Number of Davidson start vectors     ....     2
   Converg. threshold    (grad. norm)   ....   2.000e-06
   Grad. Scal. Fac. for Micro threshold ....   0.100
   Minimum threshold for Micro iter.    ....   1.000e-02
   NR start threshold (gradient norm)   ....   1.000e-04
   Initial trust radius                 ....   0.400
   Minimum AH scaling param. (alpha)    ....   1.000
   Maximum AH scaling param. (alpha)    .... 1000.000
   Quad. conv. algorithm                .... NR
   White noise on init. David. guess    .... on
   Maximum white noise                  ....   0.010
   Pseudo random numbers                .... off
   Inactive MOs                         .... canonical
   Orbital update algorithm             .... Taylor
   Preconditioner                       .... Diag
   Full preconditioner red. dimension   ....   250
 SOSCF                 CNVSOSCF           .... on
   Start iteration     SOSCFMaxIt         ....   150
   Startup grad/error  SOSCFStart         ....  0.003300
   Hessian update      SOSCFHessUp        .... L-BFGS
   Autom. constraints  SOSCFAutoConstrain .... off
 Level Shifting         CNVShift        .... on
   Level shift para.    LevelShift      ....    0.2500
   Turn off err/grad.   ShiftErr        ....    0.0010
 Zerner damping         CNVZerner       .... off
 Static damping         CNVDamp         .... on
   Fraction old density DampFac         ....    0.7000
   Max. Damping (<1)    DampMax         ....    0.9800
   Min. Damping (>=0)   DampMin         ....    0.0000
   Turn off err/grad.   DampErr         ....    0.1000

SCF Procedure:
 Maximum # iterations   MaxIter         ....   125
 SCF integral mode      SCFMode         .... Direct
   Integral package                     .... SHARK and LIBINT hybrid scheme
 Reset frequency        DirectResetFreq ....    20
 Integral Threshold     Thresh          ....  1.000e-12 Eh
 Primitive CutOff       TCut            ....  1.000e-14 Eh

Convergence Tolerance:
 Convergence Check Mode ConvCheckMode   .... Total+1el-Energy
 Convergence forced     ConvForced      .... 0
 Energy Change          TolE            ....  1.000e-09 Eh
 1-El. energy change                    ....  1.000e-06 Eh
 Orbital Gradient       TolG            ....  2.000e-06
 Orbital Rotation angle TolX            ....  2.000e-06
 DIIS Error             TolErr          ....  1.000e-08

------------------------------
INITIAL GUESS: MODEL POTENTIAL
------------------------------
Loading Hartree-Fock densities                     ... done
Calculating cut-offs                               ... done
Initializing the effective Hamiltonian             ... done
Setting up the integral package (SHARK)            ... done
Starting the Coulomb interaction                   ... done (   0.4 sec)
Making the grid                                    ... done (   0.3 sec)
Mapping shells                                     ... done
Starting the XC term evaluation                    ... done (   0.2 sec)
  promolecular density results
     # of electrons  =    155.988179286
```

```
     EX             =   -181.417039061
     EC             =     -5.718628036
     EX+EC          =   -187.135667096
Transforming the Hamiltonian                       ... done (   0.1 sec)
Diagonalizing the Hamiltonian                      ... done (   0.1 sec)
Back transforming the eigenvectors                 ... done (   0.0 sec)
Now organizing SCF variables                       ... done
                      ------------------
                      INITIAL GUESS DONE (   1.2 sec)
                      ------------------
             **** ENERGY FILE WAS UPDATED (Me-IRC.en.tmp) ****
Finished Guess after   1.5 sec
Maximum memory used throughout the entire GUESS-calculation: 48.4 MB


           ************************************************************
           *        Program running with 12 parallel MPI-processes    *
           *              working on a common directory               *
           ************************************************************

--------------------------------------------------------------------------------------------
                                      ORCA LEAN-SCF
                               memory conserving SCF solver
--------------------------------------------------------------------------------------------

--------------------
CPCM SOLVATION MODEL
--------------------
CPCM parameters:
  Epsilon                                         ...       7.4257
  Refrac                                          ...       1.4050
  Rsolv                                           ...       1.3000
  Surface type                                    ... GAUSSIAN VDW
  Discretization scheme                           ... Constant charge density
    Threshold for H atoms                         ...       5.0000 (charges/Ang^2)
    Threshold for non-H atoms                     ...       5.0000 (charges/Ang^2)
  Epsilon function type                           ...         CPCM
Solvent:                                          ... TETRAHYDROFURAN
SMD-CDS solvent descriptors:
  Soln                                            ...       1.4050
  Soln25                                          ...       1.4044
  Sola                                            ...       0.0000
  Solb                                            ...       0.4800
  Solg                                            ...      39.4400
  Solc                                            ...       0.0000
  Solh                                            ...       0.0000
Radii:
  Scheme                                          ... Element-dependent radii
  Radius for C  used is    3.4960 Bohr (=   1.8500 Ang.)
  Radius for H  used is    2.2677 Bohr (=   1.2000 Ang.)
  Radius for Se used is    3.5905 Bohr (=   1.9000 Ang.)
  Radius for O  used is    4.3350 Bohr (=   2.2940 Ang.)
Calculating surface                               ...        done! (  0.0s)
Cavity surface points                             ...         2787
Cavity Volume                                     ...    1976.0524
Cavity Surface-area                               ...    1094.4191
Calculating surface distance matrix               ...        done! (  0.1s)
Performing Cholesky decomposition & store         ...        done! (  0.2s)
Overall time for CPCM initialization              ...                 0.3s

----------------------------------------D-I-I-S--------------------------------------------
Iteration    Energy (Eh)           Delta-E    RMSDP     MaxDP     DIISErr   Damp  Time(sec)
--------------------------------------------------------------------------------------------
```

```
               ***  Starting incremental Fock matrix formation  ***
    1    -3096.3137412449323165     0.00e+00  6.07e-03  1.79e-01  3.14e-01  0.700  11.2
    2    -3096.6227197728358078    -3.09e-01  4.47e-03  1.44e-01  1.07e-01  0.700   6.3
                               ***Turning on AO-DIIS***
    3    -3096.7385002614437326    -1.16e-01  1.46e-03  3.35e-02  4.17e-02  0.700   6.0
    4    -3096.8083986165011083    -6.99e-02  2.79e-03  9.21e-02  1.97e-02  0.000   5.9
    5    -3096.9629359329201179    -1.55e-01  8.69e-04  2.29e-02  8.99e-03  0.000   6.0
                              *** Initializing SOSCF ***
---------------------------------------S-O-S-C-F--------------------------------------
Iteration    Energy (Eh)           Delta-E    RMSDP     MaxDP     MaxGrad    Time(sec)
--------------------------------------------------------------------------------------
    6    -3096.9642557372526426    -1.32e-03  3.98e-04  1.08e-02  3.12e-03     5.9
               *** Restarting incremental Fock matrix formation ***
    7    -3096.9643454778524756    -8.97e-05  4.07e-04  1.30e-02  4.58e-04    10.0
    8    -3096.9643413262638205     4.15e-06  1.26e-04  2.23e-03  4.60e-04     8.1
    9    -3096.9643615400618728    -2.02e-05  7.25e-05  8.18e-04  2.53e-04     7.5
   10    -3096.9643591597941850     2.38e-06  3.05e-05  7.56e-04  2.82e-04     7.5
   11    -3096.9643640678050360    -4.91e-06  1.59e-05  4.13e-04  3.55e-05     7.1
   12    -3096.9643639366086063     1.31e-07  7.65e-06  1.84e-04  4.90e-05     6.8
   13    -3096.9643641964812559    -2.60e-07  1.80e-06  3.02e-05  1.03e-05     6.6
   14    -3096.9643641928141733     3.67e-09  8.86e-07  1.18e-05  9.96e-06     5.7
   15    -3096.9643641992033736    -6.39e-09  3.92e-07  7.63e-06  2.52e-06     5.4
   16    -3096.9643641982256668     9.78e-10  1.96e-07  4.80e-06  2.68e-06     4.6
                 **** Energy Check signals convergence ****

               *****************************************************
               *                     SUCCESS                       *
               *           SCF CONVERGED AFTER  16 CYCLES          *
               *****************************************************

Recomputing exchange energy using gridx3       ... done (   14.027 sec)
Old exchange energy                            :    -36.603335573 Eh
New exchange energy                            :    -36.603315143 Eh
Exchange energy change after final integration :      0.000020430 Eh
Total energy after final integration           :  -3096.964343770 Eh

SMD CDS free energy correction energy :                -4.08324     Kcal/mol
Total Energy after SMD CDS correction =             -3096.970850824 Eh
             **** ENERGY FILE WAS UPDATED (Me-IRC.en.tmp) ****

----------------
TOTAL SCF ENERGY
----------------

Total Energy       :      -3096.97085082431568 Eh          -84272.86120 eV

Components:
Nuclear Repulsion  :       1761.15100708813338 Eh           47923.35528 eV
Electronic Energy  :      -4858.09960478397898 Eh         -132195.61094 eV
One Electron Energy:      -7789.61623266193055 Eh         -211966.23385 eV
Two Electron Energy:       2931.51662787795158 Eh           79770.62290 eV
CPCM Dielectric    :         -0.01576650329494 Eh              -0.42903 eV
SMD CDS (Gcds)     :         -0.00650705470658 Eh              -0.17707 eV

Virial components:
Potential Energy   :      -6187.14938617411644 Eh         -168360.89410 eV
Kinetic Energy     :       3090.17853534980122 Eh           84088.03290 eV
Virial Ratio       :          2.00219803334882

DFT components:
N(Alpha)           :       78.000073486788 electrons
N(Beta)            :       78.000073486788 electrons
N(Total)           :      156.000146973576 electrons
```

```
E(X)               :     -145.576754308839 Eh
E(C)               :       -6.728912779684 Eh
E(XC)              :     -152.305667088523 Eh

CPCM Solvation Model Properties:
Surface-charge          :         -0.06325579849761
Corrected charge        :          0.00000000000000
Outlying charge corr.   :          0.00020676074054 Eh               0.00563 eV
Free-energy (cav+disp)  :         -0.00650705470658 Eh              -0.17707 eV

---------------
SCF CONVERGENCE
---------------

  Last Energy change         ...   -9.7771e-10  Tolerance :   1.0000e-09
  Last MAX-Density change    ...    4.7990e-06  Tolerance :   1.0000e-08
  Last RMS-Density change    ...    1.9623e-07  Tolerance :   1.0000e-09
  Last DIIS Error            ...    3.1165e-03  Tolerance :   1.0000e-08
  Last Orbital Gradient      ...    2.6780e-06  Tolerance :   2.0000e-06
  Last Orbital Rotation      ...    1.8918e-06  Tolerance :   2.0000e-06


----------------
ORBITAL ENERGIES
----------------

  NO   OCC          E(Eh)            E(eV)
   0   2.0000    -453.925935    -12351.9527
   1   2.0000     -58.106286     -1581.1524
   2   2.0000     -52.158728     -1419.3112
   3   2.0000     -52.157943     -1419.2898
   4   2.0000     -52.156933     -1419.2623
   5   2.0000     -19.108357      -519.9648
   6   2.0000     -10.192586      -277.3544
   7   2.0000     -10.183946      -277.1193
   8   2.0000     -10.181392      -277.0498
   9   2.0000     -10.176185      -276.9081
  10   2.0000     -10.174493      -276.8620
  11   2.0000     -10.172744      -276.8144
  12   2.0000     -10.169623      -276.7295
  13   2.0000     -10.169029      -276.7133
  14   2.0000     -10.165918      -276.6287
  15   2.0000     -10.165015      -276.6041
  16   2.0000     -10.164413      -276.5877
  17   2.0000     -10.164124      -276.5799
  18   2.0000     -10.163577      -276.5650
  19   2.0000     -10.162504      -276.5358
  20   2.0000     -10.158259      -276.4203
  21   2.0000     -10.153067      -276.2790
  22   2.0000      -7.844620      -213.4630
  23   2.0000      -5.788573      -157.5151
  24   2.0000      -5.784669      -157.4088
  25   2.0000      -5.781005      -157.3092
  26   2.0000      -2.142069       -58.2887
  27   2.0000      -2.141277       -58.2671
  28   2.0000      -2.137642       -58.1682
  29   2.0000      -2.134267       -58.0763
  30   2.0000      -2.133925       -58.0671
  31   2.0000      -0.960146       -26.1269
  32   2.0000      -0.862357       -23.4659
  33   2.0000      -0.854142       -23.2424
  34   2.0000      -0.792596       -21.5676
  35   2.0000      -0.778318       -21.1791
```

```
  36   2.0000      -0.748006       -20.3543
  37   2.0000      -0.742448       -20.2030
  38   2.0000      -0.739196       -20.1145
  39   2.0000      -0.699910       -19.0455
  40   2.0000      -0.674843       -18.3634
  41   2.0000      -0.640267       -17.4226
  42   2.0000      -0.608979       -16.5712
  43   2.0000      -0.599602       -16.3160
  44   2.0000      -0.590238       -16.0612
  45   2.0000      -0.572980       -15.5916
  46   2.0000      -0.552372       -15.0308
  47   2.0000      -0.503177       -13.6921
  48   2.0000      -0.491698       -13.3798
  49   2.0000      -0.470090       -12.7918
  50   2.0000      -0.463862       -12.6223
  51   2.0000      -0.461846       -12.5675
  52   2.0000      -0.447516       -12.1775
  53   2.0000      -0.445421       -12.1205
  54   2.0000      -0.437352       -11.9009
  55   2.0000      -0.421404       -11.4670
  56   2.0000      -0.418846       -11.3974
  57   2.0000      -0.413893       -11.2626
  58   2.0000      -0.412074       -11.2131
  59   2.0000      -0.398713       -10.8495
  60   2.0000      -0.388261       -10.5651
  61   2.0000      -0.381867       -10.3911
  62   2.0000      -0.377444       -10.2708
  63   2.0000      -0.372421       -10.1341
  64   2.0000      -0.365083        -9.9344
  65   2.0000      -0.350750        -9.5444
  66   2.0000      -0.346367        -9.4251
  67   2.0000      -0.342313        -9.3148
  68   2.0000      -0.339490        -9.2380
  69   2.0000      -0.331950        -9.0328
  70   2.0000      -0.324301        -8.8247
  71   2.0000      -0.271646        -7.3919
  72   2.0000      -0.261959        -7.1283
  73   2.0000      -0.261040        -7.1033
  74   2.0000      -0.249663        -6.7937
  75   2.0000      -0.242173        -6.5899
  76   2.0000      -0.239862        -6.5270
  77   2.0000      -0.223636        -6.0854
  78   0.0000      -0.038105        -1.0369
  79   0.0000      -0.023472        -0.6387
  80   0.0000      -0.016929        -0.4607
  81   0.0000      -0.005899        -0.1605
  82   0.0000      -0.001963        -0.0534
  83   0.0000       0.019706         0.5362
  84   0.0000       0.034910         0.9500
  85   0.0000       0.047755         1.2995
  86   0.0000       0.060078         1.6348
  87   0.0000       0.070222         1.9108
  88   0.0000       0.075847         2.0639
*Only the first 10 virtual orbitals were printed (use %output print[p_orben] 2 end to see all).

                    ********************************
                    * MULLIKEN POPULATION ANALYSIS *
                    ********************************

-----------------------
MULLIKEN ATOMIC CHARGES
-----------------------
   0 C :    0.180440
```

```
   1 C :   -0.233650
   2 C :   -0.240204
   3 C :   -0.095665
   4 C :   -0.127687
   5 C :   -0.171955
   6 H :    0.121047
   7 H :    0.133521
   8 H :    0.137073
   9 H :    0.136252
  10 H :    0.138803
  11 C :   -0.180565
  12 H :    0.110469
  13 H :    0.115996
  14 C :   -0.265623
  15 H :    0.257184
  16 H :    0.125924
  17 C :   -0.305312
  18 H :    0.161835
  19 H :    0.160284
  20 Se:    0.408663
  21 O :   -0.572402
  22 C :    0.052641
  23 C :   -0.173325
  24 C :   -0.175349
  25 C :   -0.231459
  26 C :   -0.220743
  27 C :    0.181354
  28 H :    0.135337
  29 H :    0.152929
  30 H :    0.132635
  31 H :    0.134131
  32 C :   -0.384578
  33 H :    0.134601
  34 H :    0.135574
  35 H :    0.131821
Sum of atomic charges:   -0.0000000

--------------------------------
MULLIKEN REDUCED ORBITAL CHARGES
--------------------------------
  0 C s       :     3.155871  s :     3.155871
      pz      :     0.878807  p :     2.529472
      px      :     0.783737
      py      :     0.866928
      dz2     :     0.024933  d :     0.124383
      dxz     :     0.028906
      dyz     :     0.020214
      dx2y2   :     0.024946
      dxy     :     0.025384
      f0      :     0.001201  f :     0.009835
      f+1     :     0.001478
      f-1     :     0.001140
      f+2     :     0.001841
      f-2     :     0.001498
      f+3     :     0.001188
      f-3     :     0.001490

  1 C s       :     3.274967  s :     3.274967
      pz      :     0.965735  p :     2.869184
      px      :     0.975241
      py      :     0.928208
      dz2     :     0.018915  d :     0.082188
      dxz     :     0.019885
```

```
    dyz     :     0.011362
    dx2y2   :     0.011601
    dxy     :     0.020424
    f0      :     0.001102  f :     0.007311
    f+1     :     0.001217
    f-1     :     0.000617
    f+2     :     0.001216
    f-2     :     0.001369
    f+3     :     0.000861
    f-3     :     0.000929

2 C s       :     3.269310  s :     3.269310
    pz      :     0.972345  p :     2.881229
    px      :     0.943265
    py      :     0.965619
    dz2     :     0.013388  d :     0.082393
    dxz     :     0.017918
    dyz     :     0.011765
    dx2y2   :     0.019259
    dxy     :     0.020064
    f0      :     0.000293  f :     0.007272
    f+1     :     0.001389
    f-1     :     0.000963
    f+2     :     0.001040
    f-2     :     0.001223
    f+3     :     0.001042
    f-3     :     0.001322

3 C s       :     3.192390  s :     3.192390
    pz      :     0.946957  p :     2.813908
    px      :     0.913597
    py      :     0.953355
    dz2     :     0.013121  d :     0.082105
    dxz     :     0.017854
    dyz     :     0.010911
    dx2y2   :     0.020385
    dxy     :     0.019834
    f0      :     0.000285  f :     0.007262
    f+1     :     0.001402
    f-1     :     0.001011
    f+2     :     0.001073
    f-2     :     0.001210
    f+3     :     0.000987
    f-3     :     0.001293

4 C s       :     3.212394  s :     3.212394
    pz      :     0.940136  p :     2.825470
    px      :     0.928382
    py      :     0.956953
    dz2     :     0.018692  d :     0.082556
    dxz     :     0.020882
    dyz     :     0.010982
    dx2y2   :     0.011754
    dxy     :     0.020246
    f0      :     0.001138  f :     0.007267
    f+1     :     0.001159
    f-1     :     0.000613
    f+2     :     0.001170
    f-2     :     0.001383
    f+3     :     0.000851
    f-3     :     0.000952

5 C s       :     3.230665  s :     3.230665
```

```
      pz      :     0.944249  p :     2.853284
      px      :     0.969480
      py      :     0.939554
      dz2     :     0.019079  d :     0.080773
      dxz     :     0.016454
      dyz     :     0.015074
      dx2y2   :     0.019526
      dxy     :     0.010640
      f0      :     0.001096  f :     0.007234
      f+1     :     0.000812
      f-1     :     0.001040
      f+2     :     0.001396
      f-2     :     0.001158
      f+3     :     0.000910
      f-3     :     0.000823

  6 H s       :     0.856653  s :     0.856653
      pz      :     0.005817  p :     0.022300
      px      :     0.007467
      py      :     0.009016

  7 H s       :     0.844306  s :     0.844306
      pz      :     0.010446  p :     0.022173
      px      :     0.004664
      py      :     0.007062

  8 H s       :     0.840564  s :     0.840564
      pz      :     0.010689  p :     0.022363
      px      :     0.004667
      py      :     0.007007

  9 H s       :     0.841574  s :     0.841574
      pz      :     0.005801  p :     0.022174
      px      :     0.007477
      py      :     0.008896

 10 H s       :     0.838922  s :     0.838922
      pz      :     0.005549  p :     0.022275
      px      :     0.012275
      py      :     0.004451

 11 C s       :     3.237943  s :     3.237943
      pz      :     0.970824  p :     2.849775
      px      :     0.911180
      py      :     0.967771
      dz2     :     0.018266  d :     0.087080
      dxz     :     0.023014
      dyz     :     0.011639
      dx2y2   :     0.017035
      dxy     :     0.017127
      f0      :     0.000473  f :     0.005768
      f+1     :     0.000824
      f-1     :     0.000731
      f+2     :     0.001513
      f-2     :     0.000960
      f+3     :     0.000652
      f-3     :     0.000614

 12 H s       :     0.867818  s :     0.867818
      pz      :     0.005588  p :     0.021713
      px      :     0.005477
      py      :     0.010648
```

```
13 H s       :     0.862253  s :     0.862253
     pz      :     0.009000  p :     0.021751
     px      :     0.008062
     py      :     0.004689

14 C s       :     3.200059  s :     3.200059
     pz      :     0.996493  p :     2.964149
     px      :     0.946970
     py      :     1.020686
     dz2     :     0.024857  d :     0.095018
     dxz     :     0.014562
     dyz     :     0.019463
     dx2y2   :     0.022289
     dxy     :     0.013848
     f0      :     0.000952  f :     0.006396
     f+1     :     0.000717
     f-1     :     0.000725
     f+2     :     0.001795
     f-2     :     0.001046
     f+3     :     0.000454
     f-3     :     0.000707

15 H s       :     0.690172  s :     0.690172
     pz      :     0.010550  p :     0.052644
     px      :     0.007259
     py      :     0.034835

16 H s       :     0.851440  s :     0.851440
     pz      :     0.005821  p :     0.022636
     px      :     0.011618
     py      :     0.005198

17 C s       :     3.303327  s :     3.303327
     pz      :     1.005373  p :     2.926706
     px      :     1.000614
     py      :     0.920719
     dz2     :     0.022794  d :     0.070352
     dxz     :     0.007374
     dyz     :     0.010299
     dx2y2   :     0.014502
     dxy     :     0.015384
     f0      :     0.000652  f :     0.004926
     f+1     :     0.001175
     f-1     :     0.000589
     f+2     :     0.000450
     f-2     :     0.000892
     f+3     :     0.000367
     f-3     :     0.000802

18 H s       :     0.815815  s :     0.815815
     pz      :     0.012458  p :     0.022351
     px      :     0.004257
     py      :     0.005636

19 H s       :     0.816929  s :     0.816929
     pz      :     0.004131  p :     0.022787
     px      :     0.011982
     py      :     0.006674

20 Ses       :     7.840039  s :     7.840039
     pz      :     5.125510  p :    15.480582
     px      :     4.792786
     py      :     5.562286
```

```
     dz2     :     2.048525  d :    10.230444
     dxz     :     2.048580
     dyz     :     2.036454
     dx2y2   :     2.059706
     dxy     :     2.037179
     f0      :     0.005826  f :     0.040272
     f+1     :     0.009472
     f-1     :     0.001713
     f+2     :     0.007450
     f-2     :     0.007948
     f+3     :     0.003760
     f-3     :     0.004102

 21 O s      :     3.865913  s :     3.865913
     pz      :     1.583575  p :     4.679709
     px      :     1.491449
     py      :     1.604684
     dz2     :     0.005346  d :     0.025812
     dxz     :     0.007086
     dyz     :     0.003396
     dx2y2   :     0.005739
     dxy     :     0.004245
     f0      :     0.000109  f :     0.000967
     f+1     :     0.000226
     f-1     :     0.000040
     f+2     :     0.000223
     f-2     :     0.000157
     f+3     :     0.000115
     f-3     :     0.000096

 22 C s      :     3.167711  s :     3.167711
     pz      :     0.829549  p :     2.647664
     px      :     0.899171
     py      :     0.918944
     dz2     :     0.023572  d :     0.123709
     dxz     :     0.037147
     dyz     :     0.024664
     dx2y2   :     0.021525
     dxy     :     0.016802
     f0      :     0.001296  f :     0.008275
     f+1     :     0.001592
     f-1     :     0.000984
     f+2     :     0.001186
     f-2     :     0.001230
     f+3     :     0.001200
     f-3     :     0.000787

 23 C s      :     3.265726  s :     3.265726
     pz      :     0.925145  p :     2.809700
     px      :     0.917361
     py      :     0.967194
     dz2     :     0.024415  d :     0.090381
     dxz     :     0.025441
     dyz     :     0.015230
     dx2y2   :     0.008105
     dxy     :     0.017191
     f0      :     0.001308  f :     0.007518
     f+1     :     0.001698
     f-1     :     0.000726
     f+2     :     0.000844
     f-2     :     0.001357
     f+3     :     0.000746
     f-3     :     0.000838
```

```
24 C s       :      3.240671  s :     3.240671
     pz      :      0.951945  p :     2.838481
     px      :      0.919364
     py      :      0.967172
     dz2     :      0.022083  d :     0.088744
     dxz     :      0.016842
     dyz     :      0.009865
     dx2y2   :      0.020134
     dxy     :      0.019819
     f0      :      0.000572  f :     0.007452
     f+1     :      0.001573
     f-1     :      0.000875
     f+2     :      0.000985
     f-2     :      0.001203
     f+3     :      0.001274
     f-3     :      0.000970

25 C s       :      3.307497  s :     3.307497
     pz      :      0.950821  p :     2.838986
     px      :      0.912252
     py      :      0.975913
     dz2     :      0.018742  d :     0.077704
     dxz     :      0.015840
     dyz     :      0.009779
     dx2y2   :      0.016615
     dxy     :      0.016728
     f0      :      0.000582  f :     0.007273
     f+1     :      0.001536
     f-1     :      0.000791
     f+2     :      0.000911
     f-2     :      0.001183
     f+3     :      0.001322
     f-3     :      0.000948

26 C s       :      3.264748  s :     3.264748
     pz      :      0.936508  p :     2.866398
     px      :      0.976200
     py      :      0.953689
     dz2     :      0.021626  d :     0.082258
     dxz     :      0.021742
     dyz     :      0.015016
     dx2y2   :      0.008663
     dxy     :      0.015211
     f0      :      0.001290  f :     0.007339
     f+1     :      0.001681
     f-1     :      0.000693
     f+2     :      0.000822
     f-2     :      0.001343
     f+3     :      0.000708
     f-3     :      0.000802

27 C s       :      3.153734  s :     3.153734
     pz      :      0.840909  p :     2.525545
     px      :      0.802126
     py      :      0.882509
     dz2     :      0.027312  d :     0.129545
     dxz     :      0.034110
     dyz     :      0.024672
     dx2y2   :      0.020592
     dxy     :      0.022859
     f0      :      0.001351  f :     0.009823
     f+1     :      0.002280
```

```
      f-1     :     0.001092
      f+2     :     0.001455
      f-2     :     0.001442
      f+3     :     0.001227
      f-3     :     0.000976

 28 H s       :     0.842203  s :     0.842203
      pz      :     0.005508  p :     0.022459
      px      :     0.009420
      py      :     0.007532

 29 H s       :     0.824729  s :     0.824729
      pz      :     0.012591  p :     0.022341
      px      :     0.003933
      py      :     0.005817

 30 H s       :     0.845128  s :     0.845128
      pz      :     0.012558  p :     0.022237
      px      :     0.003881
      py      :     0.005798

 31 H s       :     0.843593  s :     0.843593
      pz      :     0.005552  p :     0.022275
      px      :     0.009047
      py      :     0.007676

 32 C s       :     3.321939  s :     3.321939
      pz      :     1.033368  p :     3.012237
      px      :     0.927711
      py      :     1.051158
      dz2     :     0.008637  d :     0.046738
      dxz     :     0.008235
      dyz     :     0.009957
      dx2y2   :     0.006908
      dxy     :     0.013002
      f0      :     0.000128  f :     0.003664
      f+1     :     0.000690
      f-1     :     0.000260
      f+2     :     0.000770
      f-2     :     0.001108
      f+3     :     0.000490
      f-3     :     0.000218

 33 H s       :     0.843922  s :     0.843922
      pz      :     0.009747  p :     0.021477
      px      :     0.004514
      py      :     0.007216

 34 H s       :     0.843101  s :     0.843101
      pz      :     0.005468  p :     0.021324
      px      :     0.006033
      py      :     0.009823

 35 H s       :     0.846577  s :     0.846577
      pz      :     0.006631  p :     0.021602
      px      :     0.008555
      py      :     0.006417


                    ******************************
                    * LOEWDIN POPULATION ANALYSIS *
                    ******************************
```

```
----------------------
LOEWDIN ATOMIC CHARGES
----------------------
   0 C :   -0.147878
   1 C :   -0.114451
   2 C :   -0.124240
   3 C :   -0.142646
   4 C :   -0.153031
   5 C :   -0.154218
   6 H :    0.150473
   7 H :    0.151096
   8 H :    0.148274
   9 H :    0.147591
  10 H :    0.147012
  11 C :   -0.211289
  12 H :    0.146896
  13 H :    0.146498
  14 C :   -0.313847
  15 H :    0.185757
  16 H :    0.143343
  17 C :   -0.231243
  18 H :    0.124927
  19 H :    0.127899
  20 Se:    0.643182
  21 O :   -0.409535
  22 C :   -0.331490
  23 C :   -0.140758
  24 C :   -0.143524
  25 C :   -0.113024
  26 C :   -0.116802
  27 C :   -0.166713
  28 H :    0.150759
  29 H :    0.152866
  30 H :    0.153416
  31 H :    0.152548
  32 C :   -0.252054
  33 H :    0.132365
  34 H :    0.132138
  35 H :    0.129707

-------------------------------
LOEWDIN REDUCED ORBITAL CHARGES
-------------------------------
  0 C s       :     2.710830  s :     2.710830
      pz      :     0.959044  p :     2.860089
      px      :     0.965029
      py      :     0.936016
      dz2     :     0.099730  d :     0.519178
      dxz     :     0.119678
      dyz     :     0.086042
      dx2y2   :     0.112159
      dxy     :     0.101569
      f0      :     0.006392  f :     0.057782
      f+1     :     0.010410
      f-1     :     0.006907
      f+2     :     0.009623
      f-2     :     0.007961
      f+3     :     0.006664
      f-3     :     0.009824

  1 C s       :     2.759570  s :     2.759570
      pz      :     0.977574  p :     2.928865
```

```
    px       :      1.011679
    py       :      0.939612
    dz2      :      0.074455  d :      0.383222
    dxz      :      0.104688
    dyz      :      0.056086
    dx2y2    :      0.057923
    dxy      :      0.090070
    f0       :      0.005820  f :      0.042794
    f+1      :      0.007550
    f-1      :      0.004845
    f+2      :      0.007666
    f-2      :      0.005515
    f+3      :      0.005001
    f-3      :      0.006397

2 C s        :      2.760276  s :      2.760276
    pz       :      0.953475  p :      2.937061
    px       :      1.028793
    py       :      0.954793
    dz2      :      0.058591  d :      0.384279
    dxz      :      0.084833
    dyz      :      0.059275
    dx2y2    :      0.088689
    dxy      :      0.092891
    f0       :      0.002535  f :      0.042624
    f+1      :      0.008704
    f-1      :      0.004391
    f+2      :      0.006862
    f-2      :      0.005780
    f+3      :      0.005261
    f-3      :      0.009091

3 C s        :      2.765619  s :      2.765619
    pz       :      0.953857  p :      2.944868
    px       :      1.037247
    py       :      0.953765
    dz2      :      0.060354  d :      0.388681
    dxz      :      0.085130
    dyz      :      0.058909
    dx2y2    :      0.090075
    dxy      :      0.094213
    f0       :      0.002525  f :      0.043477
    f+1      :      0.008860
    f-1      :      0.004635
    f+2      :      0.007068
    f-2      :      0.005876
    f+3      :      0.005296
    f-3      :      0.009218

4 C s        :      2.766403  s :      2.766403
    pz       :      0.992479  p :      2.954414
    px       :      1.016801
    py       :      0.945134
    dz2      :      0.077845  d :      0.388936
    dxz      :      0.106066
    dyz      :      0.057106
    dx2y2    :      0.056535
    dxy      :      0.091384
    f0       :      0.006086  f :      0.043277
    f+1      :      0.007484
    f-1      :      0.004967
    f+2      :      0.007709
    f-2      :      0.005582
```

```
     f+3     :     0.005026
     f-3     :     0.006423

  5 C s      :     2.766582  s :     2.766582
     pz      :     0.996310  p :     2.957292
     px      :     0.981531
     py      :     0.979451
     dz2     :     0.080716  d :     0.386894
     dxz     :     0.083896
     dyz     :     0.075965
     dx2y2   :     0.090457
     dxy     :     0.055861
     f0      :     0.005812  f :     0.043450
     f+1     :     0.006487
     f-1     :     0.006429
     f+2     :     0.007093
     f-2     :     0.006419
     f+3     :     0.005103
     f-3     :     0.006106

  6 H s      :     0.790146  s :     0.790146
     pz      :     0.015834  p :     0.059381
     px      :     0.019833
     py      :     0.023714

  7 H s      :     0.789743  s :     0.789743
     pz      :     0.027079  p :     0.059161
     px      :     0.013233
     py      :     0.018850

  8 H s      :     0.792484  s :     0.792484
     pz      :     0.026994  p :     0.059243
     px      :     0.013368
     py      :     0.018880

  9 H s      :     0.793349  s :     0.793349
     pz      :     0.015997  p :     0.059060
     px      :     0.019872
     py      :     0.023190

 10 H s      :     0.793664  s :     0.793664
     pz      :     0.015340  p :     0.059324
     px      :     0.031146
     py      :     0.012838

 11 C s      :     2.766052  s :     2.766052
     pz      :     1.010377  p :     3.016950
     px      :     0.996036
     py      :     1.010537
     dz2     :     0.064668  d :     0.390067
     dxz     :     0.100584
     dyz     :     0.069473
     dx2y2   :     0.085481
     dxy     :     0.069861
     f0      :     0.005310  f :     0.038220
     f+1     :     0.006613
     f-1     :     0.004543
     f+2     :     0.005322
     f-2     :     0.005476
     f+3     :     0.005652
     f-3     :     0.005304

 12 H s      :     0.794776  s :     0.794776
```

```
      pz      :     0.016396  p :     0.058327
      px      :     0.014071
      py      :     0.027861

 13 H s       :     0.795381  s :     0.795381
      pz      :     0.023880  p :     0.058121
      px      :     0.020145
      py      :     0.014095

 14 C s       :     2.787744  s :     2.787744
      pz      :     1.038772  p :     3.070930
      px      :     1.036226
      py      :     0.995932
      dz2     :     0.085293  d :     0.413317
      dxz     :     0.092626
      dyz     :     0.087909
      dx2y2   :     0.099116
      dxy     :     0.048374
      f0      :     0.007798  f :     0.041857
      f+1     :     0.006132
      f-1     :     0.004913
      f+2     :     0.007265
      f-2     :     0.006388
      f+3     :     0.004841
      f-3     :     0.004519

 15 H s       :     0.653333  s :     0.653333
      pz      :     0.028341  p :     0.160910
      px      :     0.018180
      py      :     0.114388

 16 H s       :     0.794724  s :     0.794724
      pz      :     0.017464  p :     0.061932
      px      :     0.029302
      py      :     0.015167

 17 C s       :     2.871672  s :     2.871672
      pz      :     1.041972  p :     3.028255
      px      :     1.075772
      py      :     0.910511
      dz2     :     0.099909  d :     0.302791
      dxz     :     0.049830
      dyz     :     0.030362
      dx2y2   :     0.055253
      dxy     :     0.067437
      f0      :     0.003063  f :     0.028525
      f+1     :     0.006576
      f-1     :     0.002734
      f+2     :     0.004147
      f-2     :     0.005668
      f+3     :     0.002380
      f-3     :     0.003956

 18 H s       :     0.810155  s :     0.810155
      pz      :     0.034582  p :     0.064918
      px      :     0.014153
      py      :     0.016184

 19 H s       :     0.805626  s :     0.805626
      pz      :     0.013161  p :     0.066475
      px      :     0.034214
      py      :     0.019100
```

```
20 Ses       :     7.326083  s :     7.326083
     pz      :     5.138462  p :    15.379279
     px      :     4.795136
     py      :     5.445681
     dz2     :     2.099716  d :    10.544966
     dxz     :     2.151484
     dyz     :     2.077984
     dx2y2   :     2.136655
     dxy     :     2.079128
     f0      :     0.014288  f :     0.106490
     f+1     :     0.026846
     f-1     :     0.003725
     f+2     :     0.023681
     f-2     :     0.019094
     f+3     :     0.009045
     f-3     :     0.009811

21 O s       :     3.626118  s :     3.626118
     pz      :     1.630822  p :     4.719685
     px      :     1.533480
     py      :     1.555383
     dz2     :     0.010866  d :     0.060924
     dxz     :     0.020070
     dyz     :     0.006068
     dx2y2   :     0.011633
     dxy     :     0.012287
     f0      :     0.000270  f :     0.002808
     f+1     :     0.000615
     f-1     :     0.000165
     f+2     :     0.000637
     f-2     :     0.000463
     f+3     :     0.000321
     f-3     :     0.000336

22 C s       :     2.784147  s :     2.784147
     pz      :     1.045379  p :     3.008407
     px      :     0.999471
     py      :     0.963557
     dz2     :     0.099187  d :     0.489492
     dxz     :     0.148400
     dyz     :     0.090955
     dx2y2   :     0.078145
     dxy     :     0.072805
     f0      :     0.007371  f :     0.049443
     f+1     :     0.011141
     f-1     :     0.007309
     f+2     :     0.005681
     f-2     :     0.007304
     f+3     :     0.005062
     f-3     :     0.005574

23 C s       :     2.765156  s :     2.765156
     pz      :     1.020772  p :     2.937860
     px      :     1.001994
     py      :     0.915093
     dz2     :     0.099828  d :     0.393614
     dxz     :     0.121084
     dyz     :     0.069445
     dx2y2   :     0.035980
     dxy     :     0.067277
     f0      :     0.007147  f :     0.044128
     f+1     :     0.011528
     f-1     :     0.005723
```

```
     f+2     :     0.005825
     f-2     :     0.005574
     f+3     :     0.003479
     f-3     :     0.004852

24 C s       :     2.764217  s :     2.764217
     pz      :     0.979126  p :     2.941728
     px      :     1.035801
     py      :     0.926801
     dz2     :     0.093150  d :     0.393620
     dxz     :     0.085175
     dyz     :     0.048480
     dx2y2   :     0.085208
     dxy     :     0.081608
     f0      :     0.003097  f :     0.043960
     f+1     :     0.010759
     f-1     :     0.004207
     f+2     :     0.006820
     f-2     :     0.007060
     f+3     :     0.004974
     f-3     :     0.007043

25 C s       :     2.759831  s :     2.759831
     pz      :     0.974356  p :     2.928676
     px      :     1.021825
     py      :     0.932495
     dz2     :     0.092973  d :     0.381722
     dxz     :     0.084471
     dyz     :     0.048291
     dx2y2   :     0.078532
     dxy     :     0.077455
     f0      :     0.003178  f :     0.042795
     f+1     :     0.010743
     f-1     :     0.003982
     f+2     :     0.006389
     f-2     :     0.006892
     f+3     :     0.004808
     f-3     :     0.006803

26 C s       :     2.759991  s :     2.759991
     pz      :     1.014775  p :     2.931202
     px      :     0.993089
     py      :     0.923338
     dz2     :     0.094673  d :     0.382843
     dxz     :     0.119568
     dyz     :     0.066167
     dx2y2   :     0.036893
     dxy     :     0.065543
     f0      :     0.006702  f :     0.042765
     f+1     :     0.011388
     f-1     :     0.005478
     f+2     :     0.005706
     f-2     :     0.005379
     f+3     :     0.003336
     f-3     :     0.004776

27 C s       :     2.716529  s :     2.716529
     pz      :     1.005912  p :     2.867473
     px      :     0.969346
     py      :     0.892215
     dz2     :     0.120514  d :     0.524665
     dxz     :     0.140768
     dyz     :     0.094163
```

```
     dx2y2   :     0.086764
     dxy     :     0.082456
     f0      :     0.007423  f :     0.058047
     f+1     :     0.015057
     f-1     :     0.007824
     f+2     :     0.006854
     f-2     :     0.008261
     f+3     :     0.005787
     f-3     :     0.006841

28 H s       :     0.789201  s :     0.789201
     pz      :     0.015291  p :     0.060040
     px      :     0.024668
     py      :     0.020082

29 H s       :     0.787348  s :     0.787348
     pz      :     0.032278  p :     0.059786
     px      :     0.011330
     py      :     0.016177

30 H s       :     0.787401  s :     0.787401
     pz      :     0.031841  p :     0.059184
     px      :     0.011362
     py      :     0.015981

31 H s       :     0.788061  s :     0.788061
     pz      :     0.015264  p :     0.059392
     px      :     0.023488
     py      :     0.020639

32 C s       :     2.819091  s :     2.819091
     pz      :     1.056214  p :     3.158289
     px      :     1.033621
     py      :     1.068455
     dz2     :     0.036350  d :     0.249433
     dxz     :     0.058207
     dyz     :     0.055751
     dx2y2   :     0.037276
     dxy     :     0.061849
     f0      :     0.003237  f :     0.025241
     f+1     :     0.005303
     f-1     :     0.002334
     f+2     :     0.003139
     f-2     :     0.003891
     f+3     :     0.004337
     f-3     :     0.003001

33 H s       :     0.806770  s :     0.806770
     pz      :     0.026653  p :     0.060865
     px      :     0.013636
     py      :     0.020576

34 H s       :     0.807009  s :     0.807009
     pz      :     0.015725  p :     0.060853
     px      :     0.016213
     py      :     0.028916

35 H s       :     0.809714  s :     0.809714
     pz      :     0.020139  p :     0.060579
     px      :     0.021900
     py      :     0.018540
```

```
                    ****************************
                    * MAYER POPULATION ANALYSIS *
                    ****************************

  NA   - Mulliken gross atomic population
  ZA   - Total nuclear charge
  QA   - Mulliken gross atomic charge
  VA   - Mayer's total valence
  BVA  - Mayer's bonded valence
  FA   - Mayer's free valence

  ATOM       NA         ZA         QA         VA         BVA        FA
  0 C      5.8196     6.0000     0.1804     3.5789     3.5789     0.0000
  1 C      6.2336     6.0000    -0.2336     3.9214     3.9214    -0.0000
  2 C      6.2402     6.0000    -0.2402     3.8823     3.8823    -0.0000
  3 C      6.0957     6.0000    -0.0957     3.8165     3.8165    -0.0000
  4 C      6.1277     6.0000    -0.1277     3.8294     3.8294    -0.0000
  5 C      6.1720     6.0000    -0.1720     3.8451     3.8451    -0.0000
  6 H      0.8790     1.0000     0.1210     0.9899     0.9899    -0.0000
  7 H      0.8665     1.0000     0.1335     0.9702     0.9702    -0.0000
  8 H      0.8629     1.0000     0.1371     0.9697     0.9697    -0.0000
  9 H      0.8637     1.0000     0.1363     0.9652     0.9652    -0.0000
 10 H      0.8612     1.0000     0.1388     0.9659     0.9659    -0.0000
 11 C      6.1806     6.0000    -0.1806     3.8455     3.8455     0.0000
 12 H      0.8895     1.0000     0.1105     0.9619     0.9619     0.0000
 13 H      0.8840     1.0000     0.1160     0.9635     0.9635     0.0000
 14 C      6.2656     6.0000    -0.2656     3.7534     3.7534    -0.0000
 15 H      0.7428     1.0000     0.2572     1.0155     1.0155    -0.0000
 16 H      0.8741     1.0000     0.1259     0.9629     0.9629    -0.0000
 17 C      6.3053     6.0000    -0.3053     3.8966     3.8966    -0.0000
 18 H      0.8382     1.0000     0.1618     0.9605     0.9605    -0.0000
 19 H      0.8397     1.0000     0.1603     0.9639     0.9639     0.0000
 20 Se    33.5913    34.0000     0.4087     2.9503     2.9503     0.0000
 21 O      8.5724     8.0000    -0.5724     1.8170     1.8170     0.0000
 22 C      5.9474     6.0000     0.0526     3.6533     3.6533    -0.0000
 23 C      6.1733     6.0000    -0.1733     3.8013     3.8013     0.0000
 24 C      6.1753     6.0000    -0.1753     3.8256     3.8256    -0.0000
 25 C      6.2315     6.0000    -0.2315     3.8621     3.8621     0.0000
 26 C      6.2207     6.0000    -0.2207     3.8812     3.8812    -0.0000
 27 C      5.8186     6.0000     0.1814     3.5692     3.5692    -0.0000
 28 H      0.8647     1.0000     0.1353     0.9912     0.9912    -0.0000
 29 H      0.8471     1.0000     0.1529     0.9644     0.9644    -0.0000
 30 H      0.8674     1.0000     0.1326     0.9702     0.9702     0.0000
 31 H      0.8659     1.0000     0.1341     0.9716     0.9716     0.0000
 32 C      6.3846     6.0000    -0.3846     3.8410     3.8410    -0.0000
 33 H      0.8654     1.0000     0.1346     0.9587     0.9587     0.0000
 34 H      0.8644     1.0000     0.1356     0.9605     0.9605     0.0000
 35 H      0.8682     1.0000     0.1318     0.9708     0.9708    -0.0000

  Mayer bond orders larger than 0.100000
B(  0-C ,  1-C ) :   1.3434 B(  0-C ,  2-C ) :   1.3373 B(  0-C , 11-C ) :   0.9088
B(  1-C ,  3-C ) :   1.3983 B(  1-C ,  6-H ) :   0.9807 B(  2-C ,  4-C ) :   1.3897
B(  2-C ,  7-H ) :   0.9909 B(  3-C ,  5-C ) :   1.3712 B(  3-C ,  8-H ) :   0.9718
B(  4-C ,  5-C ) :   1.3725 B(  4-C ,  9-H ) :   0.9831 B(  5-C , 10-H ) :   0.9781
B( 11-C , 12-H ) :   0.9544 B( 11-C , 13-H ) :   0.9778 B( 11-C , 14-C ) :   0.9542
B( 14-C , 15-H ) :   0.5412 B( 14-C , 16-H ) :   0.9425 B( 14-C , 17-C ) :   1.2998
B( 15-H , 21-O ) :   0.4004 B( 17-C , 18-H ) :   0.9572 B( 17-C , 19-H ) :   0.9523
B( 17-C , 20-Se) :   0.5074 B( 20-Se, 21-O ) :   1.2584 B( 20-Se, 22-C ) :   0.9534
B( 22-C , 23-C ) :   1.2839 B( 22-C , 24-C ) :   1.3360 B( 23-C , 25-C ) :   1.3953
B( 23-C , 28-H ) :   0.9847 B( 24-C , 26-C ) :   1.3748 B( 24-C , 29-H ) :   0.9757
B( 25-C , 27-C ) :   1.2959 B( 25-C , 30-H ) :   1.0036 B( 26-C , 27-C ) :   1.3620
B( 26-C , 31-H ) :   0.9830 B( 27-C , 32-C ) :   0.9153 B( 32-C , 33-H ) :   0.9581
```

```
B( 32-C , 34-H ) :   0.9679 B( 32-C , 35-H ) :   0.9897

-------
TIMINGS
-------

Total SCF time: 0 days 0 hours 2 min 9 sec

Total time                  ....     129.994 sec
Sum of individual times     ....     125.873 sec  ( 96.8%)

SCF preparation             ....       0.753 sec  (  0.6%)
Fock matrix formation       ....     120.451 sec  ( 92.7%)
  Startup                   ....       0.057 sec  (  0.0% of F)
  Split-RI-J                ....       8.803 sec  (  7.3% of F)
  Chain of spheres X        ....      90.002 sec  ( 74.7% of F)
  XC integration            ....      12.782 sec  ( 10.6% of F)
    XC Preparation          ....       0.000 sec  (  0.0% of XC)
    Basis function eval.    ....       3.119 sec  ( 24.4% of XC)
    Density eval.           ....       4.211 sec  ( 32.9% of XC)
    XC-Functional eval.     ....       0.113 sec  (  0.9% of XC)
    XC-Potential eval.      ....       4.243 sec  ( 33.2% of XC)
  CPCM terms                ....      12.261 sec  ( 10.2% of F)
Diagonalization             ....       0.000 sec  (  0.0%)
Density matrix formation    ....       0.480 sec  (  0.4%)
Total Energy calculation    ....       0.204 sec  (  0.2%)
Population analysis         ....       0.159 sec  (  0.1%)
Orbital Transformation      ....       0.412 sec  (  0.3%)
Orbital Orthonormalization  ....       0.000 sec  (  0.0%)
DIIS solution               ....       1.780 sec  (  1.4%)
SOSCF solution              ....       1.634 sec  (  1.3%)
Finished LeanSCF after 130.3 sec

Maximum memory used throughout the entire LEANSCF-calculation: 102.7 MB


-------------------------------------------------------------------------------
                          DFT DISPERSION CORRECTION

                              DFTD3 V3.1  Rev 1
                          USING Becke-Johnson damping
-------------------------------------------------------------------------------
The B3LYP functional is recognized
Active option DFTDOPT                   ...         4

molecular C6(AA) [au] = 16168.304070


            DFT-D V3
 parameters
 s6 scaling factor         :     1.0000
 a1 scaling factor         :     0.3981
 s8 scaling factor         :     1.9889
 a2 scaling factor         :     4.4211
 ad hoc parameters k1-k3   :    16.0000     1.3333    -4.0000

 Edisp/kcal,au: -54.870692791390  -0.087442005589
 E6   /kcal   : -25.505620375
 E8   /kcal   : -29.365072416

-------------------------   ----------------
Dispersion correction           -0.087442006
-------------------------   ----------------
```

```
-------------------------   --------------------
FINAL SINGLE POINT ENERGY     -3097.058292829905
-------------------------   --------------------


           *****************************************************
           *        Program running with 12 parallel MPI-processes    *
           *              working on a common directory              *
           *****************************************************
------------------------------------------------------------------------------
                         ORCA SCF GRADIENT CALCULATION
------------------------------------------------------------------------------

Nuc. rep. gradient        (SHARK) ... done (  0.0 sec)
HCore & Overlap gradient  (SHARK) ... done (  0.5 sec)
Split-RIJ-J gradient      (SHARK) ... done (  2.2 sec)
COSX-gradient                     ... done ( 41.2 sec)
XC gradient                       ... done (  6.3 sec)
CPCM gradient                     ... done (  2.2 sec)
SMD gradient                      ... done (  0.0 sec)
Dispersion correction             ... done (  0.0 sec)

-------------------
DISPERSION GRADIENT
-------------------

   1   C   :   -0.000271017    0.000913987    0.000483939
   2   C   :   -0.000237622    0.000953455    0.001036576
   3   C   :    0.000163793    0.000881714    0.000244375
   4   C   :   -0.000306428    0.000955368    0.000873152
   5   C   :    0.000160260    0.000785749    0.000151870
   6   C   :   -0.000091803    0.000954282    0.000433004
   7   H   :   -0.000233660    0.000074010    0.000551365
   8   H   :    0.000045780    0.000428642   -0.000166059
   9   H   :   -0.000108656    0.000191311    0.000505517
  10   H   :    0.000170703    0.000293000   -0.000095623
  11   H   :    0.000169565    0.000351252    0.000205195
  12   C   :   -0.000669392    0.000419390   -0.000027969
  13   H   :   -0.000344341    0.000263697   -0.000019195
  14   H   :   -0.000409084    0.000135919    0.000157046
  15   C   :   -0.000739246   -0.000375762   -0.000150818
  16   H   :   -0.000018969   -0.001022287   -0.000073452
  17   H   :   -0.000355991    0.000073057   -0.000172603
  18   C   :   -0.000073414   -0.001640660   -0.000493393
  19   H   :   -0.000073898    0.000099895   -0.000304828
  20   H   :   -0.000174778    0.000084254   -0.000650859
  21   Se  :   -0.001004878   -0.000396585   -0.001658915
  22   O   :   -0.000991352    0.000475094   -0.000376408
  23   C   :    0.000657798   -0.001003028   -0.000221962
  24   C   :    0.000334819   -0.000827272   -0.000069714
  25   C   :    0.000642386   -0.000740805   -0.000442341
  26   C   :    0.000332602   -0.000684857    0.000136650
  27   C   :    0.000640035   -0.000439589   -0.000417788
  28   C   :    0.000555638   -0.000507722   -0.000065871
  29   H   :   -0.000021245   -0.000378752    0.000272629
  30   H   :    0.000347106   -0.000002014   -0.000364622
  31   H   :    0.000003391   -0.000287351    0.000282750
  32   H   :    0.000291249    0.000068506   -0.000288002
  33   C   :    0.000642702   -0.000098895    0.000248255
  34   H   :    0.000363477    0.000017344    0.000257165
```

```
  35   H   :    0.000287090   -0.000057594    0.000152316
  36   H   :    0.000317383    0.000043247    0.000068617

Difference to translation invariance:
           :    0.0000000000   -0.0000000000   -0.0000000000

Difference to rotation invariance:
           :   -0.0000000000    0.0000000000   -0.0000000000

Norm of the Dispersion gradient    ...    0.0052548913
RMS gradient                       ...    0.0005056522
MAX gradient                       ...    0.0016589146

------------------
CARTESIAN GRADIENT
------------------

   1   C   :   -0.000004244    0.000025188   -0.000007352
   2   C   :    0.000006414   -0.000018410   -0.000003930
   3   C   :    0.000013714   -0.000036595   -0.000001575
   4   C   :   -0.000002705   -0.000001139    0.000003958
   5   C   :   -0.000006758    0.000005239    0.000004239
   6   C   :   -0.000001015   -0.000019345   -0.000003249
   7   H   :   -0.000001091   -0.000006645    0.000000595
   8   H   :   -0.000010621   -0.000006521    0.000004306
   9   H   :   -0.000018613    0.000042987   -0.000000595
  10   H   :   -0.000010601    0.000004170    0.000006240
  11   H   :    0.000068694   -0.000099301    0.000047630
  12   C   :   -0.000016355   -0.000009417    0.000012939
  13   H   :   -0.000025503   -0.000019080    0.000000352
  14   H   :    0.000013457   -0.000009094    0.000020555
  15   C   :    0.000043177   -0.000040363    0.000046214
  16   H   :   -0.000056313   -0.000001135   -0.000014205
  17   H   :    0.000007107    0.000019420   -0.000024815
  18   C   :   -0.000000465   -0.000009094    0.000007811
  19   H   :   -0.000005341   -0.000006075    0.000003599
  20   H   :   -0.000002910    0.000004701   -0.000003776
  21   Se  :    0.000019603    0.000005095   -0.000021568
  22   O   :    0.000016503   -0.000023071    0.000007222
  23   C   :   -0.000004645    0.000010936    0.000001820
  24   C   :    0.000008735    0.000002868    0.000003909
  25   C   :   -0.000013778    0.000028678   -0.000004583
  26   C   :   -0.000011056    0.000020919    0.000004375
  27   C   :    0.000003841   -0.000011280   -0.000008809
  28   C   :   -0.000014067    0.000057743   -0.000023411
  29   H   :    0.000008636   -0.000000589   -0.000004072
  30   H   :   -0.000003175    0.000013496   -0.000000226
  31   H   :    0.000027376   -0.000041637   -0.000005741
  32   H   :   -0.000000219    0.000014090   -0.000002319
  33   C   :    0.000026741   -0.000012194   -0.000008128
  34   H   :   -0.000066989    0.000091147   -0.000009952
  35   H   :    0.000020607    0.000005664   -0.000026433
  36   H   :   -0.000008141    0.000018644   -0.000001028

Difference to translation invariance:
           :   -0.0000000003   -0.0000000013   -0.0000000018

Difference to rotation invariance:
           :    0.0004080957    0.0006624644    0.0014598785

Norm of the Cartesian gradient     ...    0.0002477687
RMS gradient                       ...    0.0000238416
MAX gradient                       ...    0.0000993006
```

```
-------
TIMINGS
-------

Total SCF gradient time     ....       52.524 sec

Densities                   ....        0.007 sec  (  0.0%)
One electron gradient       ....        0.500 sec  (  1.0%)
RI-J Coulomb gradient       ....        2.233 sec  (  4.3%)
COSX gradient               ....       41.222 sec  ( 78.5%)
XC gradient                 ....        6.340 sec  ( 12.1%)
CPCM gradient               ....        2.177 sec  (  4.1%)
   A-Matrix (El+Nuc)        ....        0.024 sec  (  0.0%)
   Potential                ....        2.153 sec  (  4.1%)
SMD gradient                ....        0.003 sec  (  0.0%)

Maximum memory used throughout the entire SCFGRAD-calculation: 118.5 MB


           ************************************************************
           *        Program running with 12 parallel MPI-processes    *
           *              working on a common directory               *
           ************************************************************

-------------------------------------------------------------------------------
                              ORCA PROPERTY CALCULATIONS
-------------------------------------------------------------------------------

GBWName                                     ... Me-IRC.gbw
Number of atoms                             ...     36
Number of basis functions                   ...    683
Max core memory                             ...   8000 MB

Electric properties:
Dipole moment                               ... YES
Quadrupole moment                           ...  NO
General moments                             ...  NO (MaxL=10)
Static polarizability (Dipole/Dipole)       ...  NO
Static polarizability (Dipole/Quad.)        ...  NO
Static polarizability (Quad./Quad.)         ...  NO
Static polarizability (Velocity)            ...  NO
Static hyperpolarizability                  ...  NO

Atomic electric properties:
Dipole moment                               ...  NO
Quadrupole moment                           ...  NO
Static polarizability                       ...  NO

Choice of electric origin                   ... Center of mass
Position of electric origin                 ...    -1.049724    -3.370676   -12.569630

General magnetic properties:
Magnetizability                             ...  NO

EPR properties:
g-Tensor (aka g-matrix)                     ...  NO
Zero-Field splitting spin-orbit             ...  NO
Zero-field splitting spin-spin              ...  NO
Hyperfine couplings                         ...  NO (   0 nuclei)
Quadrupole couplings                        ...  NO (   0 nuclei)
Contact density                             ...  NO (   0 nuclei)
```

```
NMR properties:
Chemical shifts                         ...  NO (   0 nuclei)
Spin-rotation constants                 ...  NO (   0 nuclei)
Spin-spin couplings                     ...  NO (   0 nuclei,    0 pairs)

Choice of magnetic origin               ... GIAO
Position of magnetic origin             ...     0.000000     0.000000     0.000000

Properties with geometric perturbations:
SCF Hessian                             ...  NO
IR spectrum                             ...  NO
Raman spectrum                          ...  NO
Raman option                            ... 0
VCD spectrum                            ...  NO
X-ray spectroscopy properties:
SCF XES/XAS/RIXS spectra                ...  NO

SCF SOC stabilization energy            ...  NO
Diagonal Born-Oppenheimer correction    ...  NO

-------------
DIPOLE MOMENT
-------------

Method             : SCF
Type of density    : Electron Density
Multiplicity       :   1
Irrep              :   0
Energy             : -3096.9708508243156757 Eh
Basis              : AO
                                X               Y               Z
Electronic contribution:    -15.975280997     -29.664380142     -21.362704953
Nuclear contribution   :     17.280951928      30.375919992      20.796246142
                        -----------------------------------------
Total Dipole Moment    :      1.305670932       0.711539850      -0.566458811
                        -----------------------------------------
Magnitude (a.u.)       :      1.591207442
Magnitude (Debye)      :      4.044527894



--------------------
Rotational spectrum
--------------------

Rotational constants in cm-1:            0.014132           0.011144           0.007415
Rotational constants in MHz :          423.661083         334.091583         222.297784

Dipole components along the rotational axes:
x,y,z [a.u.] :     0.970071    -0.104794    -1.256950
x,y,z [Debye]:     2.465724    -0.266364    -3.194912



Dipole moment calculation done in   0.0 sec

Maximum memory used throughout the entire PROP-calculation: 60.5 MB


--------------------------------------------------------------------------------
                   Intrinsic Reaction Coordinate Calculation
--------------------------------------------------------------------------------
```

```
IRC Settings:
  Nr. of atoms                          .... 36
  Algorithm                             .... LQA
  Coordinate system following type      .... Mass-Weighted
  Max. no of cycles (MaxIter)           .... 241
  Direction                             .... Forward and backward
  Steep Lenght                          .... 0.110 bohr Dalton^(1/2)

Initial displacement:
  Hessian type                          .... Read
  Hessian Filename                      .... TS-Me_Compound_2.hess
  Following Hessian mode                .... 0
  Initial displacement type             .... Length
    Scaling normalized TS mode by       .... 0.040

Convergence Tolerances:
  Energy Change                         ....  5.0000e-06 Eh
  Max. Gradient                         ....  8.0000e-05 Eh/bohr
  RMS Gradient                          ....  1.0000e-05 Eh/bohr
  Max. Displacement                     ....  4.0000e-03 bohr
  RMS Displacement                      ....  2.0000e-03 bohr
  Strict Convergency criteria enforced

LQA Settings:
  Hessian update type                   .... Bofill
  Update steep lenght                   .... NO
  t Stepsize                            .... Gradient-based adjusted
  t MAX int. Steps                      .... 1000000
  Bulirsch-Stoer Correction             .... YES
    Euler Initial Steps                 .... 100
    Richardson Max extrapolations       .... 20
    Richardson error threshould         ....  1.0000e-06
    Apply BS correction on TS disp.     .... NO
    Keep LQA direction                  .... NO
    LQA direction Theshould             ....  1.0000e-01
    Update Hessian and Gradient         .... YES

Storing full IRC trajectory in        .... Me-IRC_IRC_Full_trj.xyz
Storing forward trajectory in         .... Me-IRC_IRC_F_trj.xyz
Storing backward trajectory in        .... Me-IRC_IRC_B_trj.xyz
Storing IRC profile in                .... Me-IRC.IRC.out


         *************************************************************
         *                          FORWARD IRC                      *
         *************************************************************

Iteration    E(Eh)      dE(kcal/mol)  max(|G|)  RMS(G)   max(|S|)  RMS(S)   MW-Displ.
Convergence thresholds    0.003138    0.000080  0.000010  0.004000  0.002000
    0     -3097.058338   -0.028205    0.002297  0.000485  0.034673  0.003849  0.046469
    1     -3097.058726   -0.271691    0.007435  0.001411  0.074765  0.008553  0.110006
    2     -3097.059330   -0.651100    0.011607  0.002061  0.062084  0.007733  0.110003
    3     -3097.060012   -1.078589    0.014359  0.002467  0.044546  0.006696  0.110003
    4     -3097.060725   -1.526087    0.016021  0.002722  0.031324  0.005913  0.110001
    5     -3097.061460   -1.987438    0.017548  0.002895  0.029828  0.005408  0.110002
    6     -3097.062214   -2.460374    0.018915  0.003022  0.028390  0.005082  0.110002
    7     -3097.062980   -2.941503    0.020056  0.003123  0.027142  0.004864  0.110000
    8     -3097.063759   -3.429774    0.021041  0.003208  0.026067  0.004711  0.110003
    9     -3097.064551   -3.927065    0.021911  0.003282  0.025121  0.004597  0.110004
   10     -3097.065348   -4.427122    0.022692  0.003348  0.024277  0.004509  0.110002
   11     -3097.066161   -4.937202    0.023400  0.003408  0.023513  0.004439  0.110002
   12     -3097.066979   -5.450717    0.024043  0.003462  0.022820  0.004383  0.110003
   13     -3097.067804   -5.968158    0.024623  0.003511  0.022185  0.004337  0.110002
```

14	-3097.068636	-6.490622	0.025144	0.003554	0.021601	0.004300	0.110002
15	-3097.069477	-7.018081	0.025603	0.003591	0.021066	0.004270	0.110002
16	-3097.070320	-7.547309	0.025996	0.003622	0.020574	0.004246	0.110001
17	-3097.071167	-8.078877	0.026321	0.003645	0.020116	0.004227	0.110001
18	-3097.072017	-8.611793	0.026574	0.003660	0.019697	0.004213	0.110003
19	-3097.072867	-9.145211	0.026752	0.003668	0.019314	0.004201	0.110001
20	-3097.073713	-9.676069	0.026852	0.003666	0.018969	0.004193	0.110002
21	-3097.074563	-10.209582	0.026877	0.003657	0.018646	0.004187	0.110002
22	-3097.075405	-10.737945	0.026819	0.003638	0.018359	0.004183	0.110002
23	-3097.076241	-11.262727	0.026681	0.003608	0.018026	0.004177	0.110002
24	-3097.077055	-11.773408	0.026455	0.003569	0.017630	0.004170	0.110003
25	-3097.077866	-12.282601	0.026153	0.003519	0.017142	0.004159	0.110004
26	-3097.078661	-12.781444	0.025774	0.003462	0.016657	0.004143	0.110001
27	-3097.079434	-13.266467	0.025319	0.003397	0.016120	0.004112	0.110002
28	-3097.080183	-13.736300	0.024775	0.003323	0.015699	0.004064	0.110002
29	-3097.080911	-14.192831	0.024145	0.003238	0.015493	0.004037	0.110000
30	-3097.081617	-14.635927	0.023437	0.003144	0.015241	0.004027	0.110003
31	-3097.082299	-15.063906	0.022654	0.003041	0.014848	0.004013	0.110001
32	-3097.082954	-15.475366	0.021798	0.002932	0.014450	0.003993	0.110002
33	-3097.083583	-15.869497	0.020870	0.002814	0.014191	0.003973	0.110000
34	-3097.084182	-16.245936	0.019873	0.002689	0.014286	0.003952	0.110001
35	-3097.084752	-16.603456	0.018814	0.002558	0.014404	0.003930	0.110003
36	-3097.085291	-16.941675	0.017695	0.002421	0.014545	0.003908	0.110001
37	-3097.085798	-17.259745	0.016525	0.002279	0.014711	0.003885	0.110003
38	-3097.086274	-17.558338	0.015308	0.002132	0.014895	0.003861	0.110001
39	-3097.086717	-17.836650	0.014058	0.001985	0.015117	0.003841	0.110002
40	-3097.087130	-18.095298	0.012780	0.001832	0.015324	0.003822	0.110001
41	-3097.087509	-18.333155	0.011499	0.001689	0.015585	0.003810	0.110001
42	-3097.087862	-18.554796	0.010214	0.001531	0.015764	0.003803	0.110001
43	-3097.088175	-18.751238	0.008982	0.001421	0.016091	0.003816	0.110002
44	-3097.088483	-18.944585	0.007741	0.001229	0.016001	0.003875	0.110001
45	-3097.088756	-19.115782	0.006837	0.001237	0.017835	0.004608	0.110001
46	-3097.089022	-19.282581	0.006352	0.000988	0.016101	0.003992	0.110001
47	-3097.089241	-19.420408	0.005794	0.000995	0.017848	0.004505	0.110001
48	-3097.089474	-19.566485	0.005345	0.000768	0.015933	0.004309	0.110000
49	-3097.089670	-19.689591	0.004899	0.000882	0.020412	0.005040	0.110000
50	-3097.089866	-19.812136	0.004528	0.000739	0.018092	0.004501	0.110001
51	-3097.090058	-19.932935	0.004121	0.000615	0.020407	0.004930	0.110001
52	-3097.090244	-20.049320	0.003746	0.000530	0.022033	0.005269	0.110001
53	-3097.090413	-20.155819	0.003258	0.000527	0.022619	0.005461	0.110001
54	-3097.090576	-20.257955	0.003016	0.000520	0.022071	0.005621	0.110000
55	-3097.090736	-20.358588	0.002809	0.000508	0.023312	0.005713	0.110000
56	-3097.090893	-20.457033	0.002641	0.000506	0.023151	0.005747	0.110000
57	-3097.091047	-20.553698	0.002501	0.000509	0.023136	0.005692	0.110000
58	-3097.091200	-20.649564	0.002387	0.000504	0.023502	0.005624	0.110001
59	-3097.091352	-20.745107	0.002290	0.000494	0.023873	0.005631	0.110000
60	-3097.091503	-20.839804	0.002205	0.000483	0.024094	0.005681	0.110001
61	-3097.091651	-20.932383	0.002128	0.000486	0.024075	0.005704	0.110000
62	-3097.091795	-21.022595	0.002058	0.000497	0.024142	0.005694	0.110001
63	-3097.091935	-21.110956	0.002023	0.000487	0.024432	0.005714	0.110000
64	-3097.092075	-21.198326	0.001974	0.000474	0.024562	0.005753	0.110001
65	-3097.092210	-21.283488	0.002027	0.000473	0.024112	0.005764	0.110001
66	-3097.092343	-21.366554	0.002099	0.000476	0.023623	0.005691	0.110001
67	-3097.092472	-21.447929	0.002129	0.000476	0.023422	0.005627	0.110001
68	-3097.092599	-21.527526	0.002164	0.000476	0.023247	0.005598	0.110001
69	-3097.092723	-21.605458	0.002186	0.000474	0.023138	0.005586	0.110000
70	-3097.092845	-21.681698	0.002205	0.000472	0.022976	0.005570	0.110001
71	-3097.092964	-21.756202	0.002231	0.000470	0.022756	0.005544	0.110000
72	-3097.093080	-21.828951	0.002257	0.000468	0.022543	0.005513	0.110001
73	-3097.093193	-21.899973	0.002281	0.000467	0.022356	0.005488	0.110001
74	-3097.093303	-21.969280	0.002302	0.000465	0.022196	0.005468	0.110001
75	-3097.093411	-22.036799	0.002324	0.000463	0.022022	0.005445	0.110000
76	-3097.093516	-22.102553	0.002345	0.000462	0.021848	0.005417	0.110000

77	-3097.093617	-22.166453	0.002364	0.000460	0.021663	0.005384	0.110000
78	-3097.093716	-22.228534	0.002381	0.000458	0.021604	0.005345	0.110000
79	-3097.093812	-22.288649	0.002395	0.000456	0.021603	0.005301	0.110000
80	-3097.093904	-22.346309	0.002404	0.000454	0.021567	0.005253	0.110000
81	-3097.093993	-22.402335	0.002405	0.000451	0.021493	0.005200	0.110000
82	-3097.094079	-22.456194	0.002404	0.000449	0.021367	0.005138	0.110001
83	-3097.094162	-22.507890	0.002383	0.000446	0.021181	0.005071	0.110000
84	-3097.094240	-22.557367	0.002329	0.000441	0.020954	0.004999	0.110000
85	-3097.094318	-22.606068	0.002189	0.000433	0.020687	0.004926	0.110000
86	-3097.094391	-22.651953	0.001867	0.000422	0.020290	0.004847	0.110000
87	-3097.094457	-22.693046	0.001586	0.000395	0.019979	0.004736	0.110000
88	-3097.094523	-22.734617	0.001792	0.000398	0.019481	0.004613	0.110000
89	-3097.094583	-22.772248	0.001745	0.000403	0.018665	0.004439	0.110000
90	-3097.094637	-22.806336	0.001939	0.000442	0.018172	0.004358	0.110000
91	-3097.094689	-22.839016	0.002408	0.000453	0.017004	0.004134	0.110000
92	-3097.094734	-22.867372	0.003106	0.000522	0.017061	0.004136	0.110000
93	-3097.094752	-22.878375	0.002644	0.000448	0.011309	0.002158	0.110000
94	-3097.094798	-22.907480	0.001346	0.000372	0.020695	0.004089	0.110002
95	-3097.094855	-22.942941	0.001069	0.000244	0.023576	0.005181	0.110001
96	-3097.094901	-22.972128	0.001018	0.000206	0.021347	0.005669	0.110000
97	-3097.094932	-22.991591	0.001903	0.000381	0.019492	0.004916	0.110001
98	-3097.094977	-23.019919	0.000947	0.000241	0.016038	0.003900	0.110001
99	-3097.095018	-23.045397	0.000789	0.000179	0.017563	0.004322	0.110000
100	-3097.095048	-23.064406	0.001412	0.000338	0.016622	0.004219	0.110001
101	-3097.095086	-23.088025	0.000994	0.000250	0.012833	0.003949	0.110001
102	-3097.095115	-23.106195	0.001047	0.000286	0.015254	0.004347	0.110000
103	-3097.095148	-23.126837	0.001237	0.000270	0.014835	0.004179	0.110000
104	-3097.095178	-23.145453	0.001249	0.000261	0.014075	0.004155	0.110000
105	-3097.095206	-23.163259	0.001380	0.000273	0.013546	0.003996	0.110000
106	-3097.095235	-23.181218	0.001339	0.000274	0.013212	0.003927	0.110000
107	-3097.095261	-23.197613	0.001182	0.000284	0.012640	0.003863	0.110000
108	-3097.095288	-23.214501	0.001257	0.000304	0.013255	0.003910	0.110000
109	-3097.095313	-23.230617	0.001287	0.000292	0.011703	0.003771	0.110000
110	-3097.095339	-23.246886	0.001155	0.000267	0.012053	0.003757	0.110000
111	-3097.095363	-23.261592	0.000818	0.000233	0.010172	0.003514	0.110000
112	-3097.095387	-23.276679	0.001143	0.000263	0.013650	0.004018	0.110000
113	-3097.095411	-23.292265	0.001292	0.000265	0.011351	0.003693	0.110000
114	-3097.095438	-23.308588	0.001260	0.000260	0.012231	0.003790	0.110000
115	-3097.095461	-23.323458	0.001113	0.000260	0.011759	0.003757	0.110000
116	-3097.095467	-23.327357	0.000975	0.000257	0.009484	0.002097	0.110000
117	-3097.095492	-23.343018	0.001035	0.000194	0.012553	0.004326	0.110001
118	-3097.095509	-23.353416	0.001194	0.000298	0.012153	0.004117	0.110000
119	-3097.095537	-23.370935	0.000943	0.000195	0.011021	0.003663	0.110000
120	-3097.095563	-23.387606	0.000865	0.000147	0.012309	0.004440	0.110000
121	-3097.095582	-23.399276	0.001104	0.000297	0.014818	0.004452	0.110001
122	-3097.095609	-23.416400	0.000934	0.000223	0.013079	0.003858	0.110000
123	-3097.095636	-23.433183	0.000792	0.000164	0.014618	0.004154	0.110000
124	-3097.095660	-23.447986	0.000732	0.000167	0.013999	0.004183	0.110000
125	-3097.095683	-23.462908	0.000734	0.000170	0.012810	0.004281	0.110000
126	-3097.095709	-23.478823	0.000661	0.000178	0.012568	0.004124	0.110000
127	-3097.095731	-23.492920	0.000937	0.000231	0.013016	0.004174	0.110000
128	-3097.095756	-23.508511	0.001125	0.000264	0.012099	0.004050	0.110000
129	-3097.095778	-23.522217	0.000827	0.000213	0.011070	0.003931	0.110000
130	-3097.095804	-23.538794	0.000651	0.000182	0.014554	0.004233	0.110000
131	-3097.095829	-23.553946	0.000716	0.000189	0.014894	0.004309	0.110000
132	-3097.095852	-23.568696	0.000819	0.000211	0.014824	0.004244	0.110000
133	-3097.095876	-23.583712	0.001076	0.000240	0.014470	0.004211	0.110000
134	-3097.095897	-23.597178	0.001168	0.000246	0.013200	0.004247	0.110000
135	-3097.095920	-23.611290	0.001062	0.000253	0.013324	0.004045	0.110000
136	-3097.095943	-23.625857	0.001124	0.000259	0.012724	0.004038	0.110000
137	-3097.095967	-23.640704	0.001051	0.000254	0.012221	0.003954	0.110000
138	-3097.095992	-23.656537	0.001077	0.000252	0.012614	0.004015	0.110000
139	-3097.096015	-23.671235	0.001103	0.000254	0.012731	0.004026	0.110000

140	-3097.096039	-23.686135	0.001082	0.000249	0.012958	0.004021	0.110000
141	-3097.096062	-23.700607	0.001065	0.000246	0.013352	0.004056	0.110000
142	-3097.096085	-23.715162	0.001099	0.000250	0.013508	0.004074	0.110000
143	-3097.096109	-23.729657	0.001110	0.000253	0.013134	0.004045	0.110000
144	-3097.096129	-23.742706	0.001118	0.000255	0.012877	0.004024	0.110000
145	-3097.096152	-23.757042	0.001165	0.000261	0.012689	0.004027	0.110000
146	-3097.096177	-23.772623	0.001173	0.000261	0.012320	0.004008	0.110000
147	-3097.096198	-23.786030	0.001123	0.000253	0.011874	0.003981	0.110000
148	-3097.096221	-23.800093	0.001180	0.000259	0.012233	0.004024	0.110000
149	-3097.096244	-23.814657	0.001183	0.000258	0.012025	0.004009	0.110000
150	-3097.096267	-23.828969	0.001171	0.000256	0.012219	0.004009	0.110000
151	-3097.096290	-23.843843	0.001168	0.000256	0.012296	0.004019	0.110000
152	-3097.096311	-23.857019	0.001091	0.000245	0.011856	0.003980	0.110000
153	-3097.096333	-23.870791	0.001204	0.000261	0.012760	0.004070	0.110000
154	-3097.096355	-23.884493	0.001284	0.000260	0.014634	0.004294	0.110000
155	-3097.096378	-23.898527	0.001221	0.000261	0.012294	0.004053	0.110000
156	-3097.096401	-23.912953	0.001257	0.000266	0.012336	0.004026	0.110000
157	-3097.096424	-23.927328	0.001295	0.000268	0.011839	0.004050	0.110000
158	-3097.096445	-23.940940	0.001253	0.000257	0.012116	0.004140	0.110000
159	-3097.096468	-23.955436	0.001306	0.000267	0.012370	0.004121	0.110000
160	-3097.096492	-23.970028	0.001291	0.000268	0.012270	0.004028	0.110000
161	-3097.096515	-23.984540	0.001321	0.000269	0.011939	0.004046	0.110000
162	-3097.096538	-23.999121	0.001276	0.000267	0.011898	0.003999	0.110000
163	-3097.096561	-24.013555	0.001347	0.000274	0.011903	0.004040	0.110000
164	-3097.096583	-24.027439	0.001252	0.000257	0.012470	0.004129	0.110000
165	-3097.096607	-24.042537	0.001361	0.000272	0.012343	0.004112	0.110000
166	-3097.096633	-24.058869	0.001322	0.000268	0.011681	0.003998	0.110000
167	-3097.096639	-24.062651	0.001025	0.000251	0.010087	0.002247	0.110000
168	-3097.096663	-24.077840	0.000826	0.000150	0.011826	0.004171	0.110001
169	-3097.096691	-24.095023	0.000742	0.000167	0.012610	0.004679	0.110000
170	-3097.096715	-24.110009	0.000947	0.000204	0.015122	0.004294	0.110000
171	-3097.096738	-24.124497	0.000887	0.000206	0.015755	0.004491	0.110000
172	-3097.096762	-24.139888	0.000798	0.000189	0.014114	0.004345	0.110000
173	-3097.096788	-24.155780	0.000950	0.000217	0.013751	0.004348	0.110000
174	-3097.096812	-24.171223	0.001001	0.000220	0.014166	0.004256	0.110000
175	-3097.096837	-24.186627	0.000995	0.000223	0.013332	0.004271	0.110000
176	-3097.096861	-24.202120	0.000955	0.000215	0.013458	0.004221	0.110000
177	-3097.096886	-24.217587	0.000931	0.000218	0.012885	0.004262	0.110000
178	-3097.096907	-24.230541	0.000747	0.000192	0.012803	0.004159	0.110000
179	-3097.096931	-24.245487	0.000728	0.000189	0.014469	0.004250	0.110000
180	-3097.096954	-24.260447	0.000841	0.000202	0.014184	0.004246	0.110000
181	-3097.096973	-24.272342	0.000834	0.000205	0.014044	0.004099	0.110000
182	-3097.096995	-24.285630	0.000803	0.000201	0.014608	0.004216	0.110000
183	-3097.097015	-24.298626	0.000781	0.000203	0.014640	0.004232	0.110000
184	-3097.097036	-24.311448	0.000764	0.000203	0.014552	0.004238	0.110000
185	-3097.097056	-24.324083	0.000777	0.000204	0.014375	0.004227	0.110000
186	-3097.097068	-24.331763	0.000738	0.000202	0.014011	0.004259	0.110000
187	-3097.097098	-24.350739	0.000774	0.000204	0.015445	0.004312	0.110000
188	-3097.097119	-24.363650	0.000822	0.000208	0.012799	0.004287	0.110000
189	-3097.097137	-24.375157	0.000738	0.000198	0.012145	0.004158	0.110000
190	-3097.097158	-24.388352	0.000760	0.000200	0.012774	0.004254	0.110000
191	-3097.097181	-24.402507	0.000809	0.000200	0.013664	0.004279	0.110000
192	-3097.097184	-24.404603	0.000610	0.000159	0.008073	0.001534	0.110000
193	-3097.097204	-24.416879	0.000620	0.000127	0.012344	0.004281	0.110002
194	-3097.097225	-24.430191	0.000734	0.000196	0.013212	0.004615	0.110000
195	-3097.097247	-24.443894	0.000931	0.000211	0.014121	0.004334	0.110000
196	-3097.097268	-24.457375	0.000906	0.000204	0.013310	0.004330	0.110000
197	-3097.097290	-24.471171	0.000877	0.000200	0.012760	0.004358	0.110000
198	-3097.097312	-24.484845	0.000877	0.000202	0.012685	0.004377	0.110000
199	-3097.097334	-24.498554	0.000895	0.000205	0.012977	0.004384	0.110000
200	-3097.097356	-24.512253	0.000910	0.000206	0.013277	0.004397	0.110000
201	-3097.097378	-24.526058	0.000921	0.000207	0.013407	0.004415	0.110000
202	-3097.097400	-24.539780	0.000933	0.000209	0.013452	0.004430	0.110000

```
  203     -3097.097420   -24.552839    0.000946  0.000212  0.013469  0.004448  0.110000
  204     -3097.097444   -24.567644    0.000961  0.000215  0.013568  0.004501  0.110000
  205     -3097.097466   -24.581703    0.000966  0.000216  0.013171  0.004526  0.110000
  206     -3097.097489   -24.595683    0.000975  0.000218  0.013100  0.004554  0.110000
  207     -3097.097510   -24.608954    0.000985  0.000220  0.012974  0.004572  0.110000
  208     -3097.097532   -24.622999    0.001000  0.000223  0.013606  0.004599  0.110000
  209     -3097.097555   -24.637121    0.001011  0.000225  0.013947  0.004619  0.110000
  210     -3097.097577   -24.651219    0.001017  0.000224  0.012853  0.004549  0.110000
  211     -3097.097601   -24.665892    0.001024  0.000224  0.012822  0.004472  0.110000
  212     -3097.097621   -24.678722    0.000994  0.000227  0.011967  0.004378  0.110000
  213     -3097.097644   -24.692939    0.001053  0.000228  0.013147  0.004367  0.110000
  214     -3097.097666   -24.706808    0.001054  0.000230  0.012672  0.004430  0.110000
  215     -3097.097689   -24.721462    0.001065  0.000232  0.012846  0.004431  0.110000
  216     -3097.097711   -24.735137    0.001050  0.000229  0.012399  0.004421  0.110000
  217     -3097.097732   -24.748427    0.001033  0.000236  0.011856  0.004347  0.110000
  218     -3097.097753   -24.761654    0.001054  0.000227  0.012659  0.004334  0.110000
  219     -3097.097775   -24.775662    0.001089  0.000239  0.012287  0.004438  0.110000
  220     -3097.097796   -24.788580    0.001053  0.000227  0.012324  0.004397  0.110000
  221     -3097.097816   -24.801014    0.001059  0.000243  0.011390  0.004331  0.110000
  222     -3097.097838   -24.814649    0.001090  0.000234  0.012714  0.004314  0.110000
  223     -3097.097846   -24.819782    0.001109  0.000244  0.011801  0.004409  0.110000
  224     -3097.097878   -24.840164    0.001151  0.000246  0.014105  0.004467  0.110000
  225     -3097.097898   -24.852686    0.001064  0.000237  0.010672  0.004435  0.110000
  226     -3097.097918   -24.865056    0.001084  0.000246  0.010867  0.004329  0.110000
  227     -3097.097939   -24.878214    0.001071  0.000230  0.011166  0.004322  0.110000
  228     -3097.097957   -24.889897    0.001113  0.000244  0.012498  0.004480  0.110000
  229     -3097.097978   -24.902927    0.001084  0.000239  0.011117  0.004317  0.110000
  230     -3097.097996   -24.914157    0.001071  0.000230  0.011144  0.004386  0.110000
  231     -3097.098015   -24.926058    0.001126  0.000251  0.010919  0.004362  0.110000
  232     -3097.098034   -24.937999    0.000980  0.000203  0.011468  0.004317  0.110000
  233     -3097.098052   -24.949414    0.001036  0.000241  0.011270  0.004462  0.110000
  234     -3097.098071   -24.960951    0.001040  0.000221  0.011451  0.004384  0.110000
  235     -3097.098086   -24.970660    0.001116  0.000257  0.011291  0.004400  0.110000
  236     -3097.098105   -24.982531    0.001004  0.000212  0.011959  0.004391  0.110000
  237     -3097.098122   -24.993086    0.001046  0.000245  0.012161  0.004458  0.110000
  238     -3097.098139   -25.003945    0.001036  0.000228  0.010812  0.004274  0.110000
  239     -3097.098155   -25.013595    0.001101  0.000248  0.013063  0.004376  0.110000
  240     -3097.098172   -25.024398    0.001019  0.000224  0.012594  0.004391  0.110000

         *************************************************************
         *  MAXIMUM NUMBER OF ITERATIONS REACHED - STOPPING IRC RUN  *
         *************************************************************


         *************************************************************
         *                         BACKWARD IRC                      *
         *************************************************************

Iteration     E(Eh)      dE(kcal/mol)  max(|G|)   RMS(G)   max(|S|)   RMS(S)   MW-Displ.
Convergence thresholds     0.003138    0.000080  0.000010  0.004000  0.002000
    0     -3097.058345   -0.032703    0.002344  0.000533  0.034673  0.003849  0.046469
    1     -3097.059022   -0.457303    0.008372  0.002043  0.085688  0.009405  0.110004
    2     -3097.060632   -1.467818    0.014788  0.003568  0.088115  0.009579  0.110008
    3     -3097.062835   -2.850525    0.019365  0.004530  0.086242  0.009455  0.110000
    4     -3097.065016   -4.218794    0.022961  0.004964  0.078075  0.008894  0.110003
    5     -3097.066827   -5.355343    0.025032  0.005045  0.057802  0.007581  0.110002
    6     -3097.068328   -6.296977    0.025126  0.004914  0.030947  0.006091  0.110005
    7     -3097.069665   -7.136017    0.024847  0.004703  0.025132  0.005301  0.110002
    8     -3097.070887   -7.902858    0.025103  0.004488  0.020779  0.004849  0.110004
    9     -3097.072019   -8.613423    0.025190  0.004289  0.019560  0.004544  0.110002
   10     -3097.073081   -9.279404    0.025147  0.004111  0.019519  0.004340  0.110001
   11     -3097.074082   -9.908124    0.025001  0.003950  0.019325  0.004215  0.110001
   12     -3097.075034   -10.505322    0.024770  0.003804  0.019299  0.004149  0.110001
```

13	-3097.075942	-11.074687	0.024471	0.003672	0.020035	0.004123	0.110002
14	-3097.076817	-11.624192	0.024121	0.003549	0.020470	0.004122	0.110001
15	-3097.077659	-12.152388	0.023734	0.003435	0.020680	0.004136	0.110001
16	-3097.078468	-12.659800	0.023319	0.003327	0.020738	0.004156	0.110001
17	-3097.079249	-13.150025	0.022884	0.003223	0.020691	0.004178	0.110002
18	-3097.080004	-13.623840	0.022438	0.003123	0.020571	0.004201	0.110002
19	-3097.080732	-14.080737	0.021986	0.003028	0.020389	0.004223	0.110001
20	-3097.081435	-14.521929	0.021529	0.002936	0.020166	0.004244	0.110003
21	-3097.082114	-14.948014	0.021071	0.002849	0.019915	0.004262	0.110003
22	-3097.082770	-15.359316	0.020617	0.002766	0.019642	0.004277	0.110001
23	-3097.083398	-15.753407	0.020179	0.002693	0.019327	0.004289	0.110001
24	-3097.083995	-16.128243	0.019773	0.002643	0.018879	0.004280	0.110001
25	-3097.084562	-16.483804	0.019367	0.002599	0.018188	0.004187	0.110001
26	-3097.085102	-16.823079	0.018936	0.002524	0.017570	0.004095	0.110002
27	-3097.085624	-17.150325	0.018506	0.002454	0.017278	0.004097	0.110001
28	-3097.086125	-17.464931	0.018081	0.002389	0.017415	0.004083	0.110002
29	-3097.086607	-17.767386	0.017663	0.002326	0.017550	0.004062	0.110001
30	-3097.087071	-18.058307	0.017252	0.002263	0.017668	0.004044	0.110000
31	-3097.087517	-18.338302	0.016849	0.002202	0.017790	0.004026	0.110002
32	-3097.087946	-18.607734	0.016455	0.002144	0.017891	0.004011	0.110001
33	-3097.088359	-18.867056	0.016075	0.002087	0.017974	0.003992	0.110001
34	-3097.088757	-19.116643	0.015684	0.002032	0.018041	0.003975	0.110001
35	-3097.089140	-19.356933	0.015300	0.001977	0.018097	0.003957	0.110001
36	-3097.089509	-19.588179	0.014922	0.001925	0.018139	0.003940	0.110002
37	-3097.089864	-19.810863	0.014549	0.001873	0.018170	0.003922	0.110001
38	-3097.090205	-20.025101	0.014183	0.001824	0.018192	0.003905	0.110001
39	-3097.090534	-20.231480	0.013822	0.001775	0.018209	0.003887	0.110000
40	-3097.090850	-20.430006	0.013467	0.001729	0.018214	0.003869	0.110001
41	-3097.091155	-20.621471	0.013117	0.001682	0.018210	0.003850	0.110000
42	-3097.091449	-20.805464	0.012774	0.001640	0.018202	0.003833	0.110002
43	-3097.091732	-20.983295	0.012436	0.001593	0.018186	0.003813	0.110001
44	-3097.092003	-21.153612	0.012094	0.001557	0.018171	0.003798	0.110001
45	-3097.092267	-21.319191	0.011695	0.001500	0.018150	0.003779	0.110001
46	-3097.092517	-21.475690	0.011378	0.001481	0.018154	0.003773	0.110001
47	-3097.092765	-21.631509	0.011060	0.001408	0.018112	0.003749	0.110001
48	-3097.092991	-21.773423	0.010755	0.001431	0.018109	0.003752	0.110001
49	-3097.093229	-21.922949	0.010438	0.001295	0.017987	0.003729	0.110001
50	-3097.093452	-22.062964	0.010108	0.001286	0.018643	0.003875	0.110001
51	-3097.093664	-22.195874	0.009836	0.001305	0.018580	0.003792	0.110001
52	-3097.093875	-22.328276	0.009560	0.001217	0.018010	0.003698	0.110000
53	-3097.094061	-22.444648	0.009281	0.001277	0.018086	0.003710	0.110000
54	-3097.094268	-22.574637	0.009001	0.001112	0.017842	0.003681	0.110001
55	-3097.094470	-22.701677	0.008680	0.001032	0.019202	0.003971	0.110000
56	-3097.094676	-22.830460	0.008369	0.000989	0.019718	0.004099	0.110002
57	-3097.094846	-22.937652	0.008104	0.001075	0.019385	0.004047	0.110001
58	-3097.095022	-23.048028	0.007913	0.001029	0.019090	0.003810	0.110002
59	-3097.095170	-23.140924	0.007697	0.001114	0.017763	0.003665	0.110000
60	-3097.095339	-23.246958	0.007475	0.000949	0.017531	0.003605	0.110001
61	-3097.095494	-23.344258	0.007211	0.000905	0.018532	0.003811	0.110001
62	-3097.095630	-23.429381	0.006995	0.001044	0.018500	0.003784	0.110000
63	-3097.095785	-23.526463	0.006804	0.000866	0.017430	0.003626	0.110000
64	-3097.095932	-23.619177	0.006555	0.000782	0.018692	0.003886	0.110001
65	-3097.096084	-23.714069	0.006316	0.000745	0.019352	0.004032	0.110000
66	-3097.096210	-23.793520	0.006105	0.000811	0.019127	0.003992	0.110000
67	-3097.096322	-23.863705	0.005974	0.000931	0.018694	0.003765	0.110001
68	-3097.096449	-23.943553	0.005834	0.000798	0.016845	0.003570	0.110001
69	-3097.096562	-24.013995	0.005635	0.000817	0.017214	0.003693	0.110000
70	-3097.096668	-24.081021	0.005490	0.000865	0.017216	0.003594	0.110000
71	-3097.096778	-24.150000	0.005359	0.000799	0.016387	0.003511	0.110001
72	-3097.096880	-24.213788	0.005206	0.000836	0.016338	0.003501	0.110001
73	-3097.096980	-24.276220	0.005079	0.000780	0.016113	0.003474	0.110000
74	-3097.097075	-24.336427	0.004941	0.000813	0.015949	0.003469	0.110000
75	-3097.097169	-24.395033	0.004827	0.000758	0.015699	0.003443	0.110000

76	-3097.097259	-24.451743	0.004698	0.000792	0.015561	0.003442	0.110000
77	-3097.097288	-24.469762	0.005060	0.000874	0.019080	0.002398	0.110000
78	-3097.097390	-24.533638	0.004544	0.000521	0.014353	0.003491	0.110003
79	-3097.097488	-24.595150	0.004367	0.000504	0.017866	0.004051	0.110000
80	-3097.097580	-24.653082	0.004226	0.000513	0.017198	0.003920	0.110001
81	-3097.097668	-24.708040	0.004105	0.000512	0.016597	0.003880	0.110001
82	-3097.097752	-24.761238	0.003998	0.000507	0.016884	0.003850	0.110001
83	-3097.097834	-24.812431	0.003899	0.000499	0.017273	0.003865	0.110000
84	-3097.097914	-24.862310	0.003800	0.000492	0.017412	0.003842	0.110000
85	-3097.097990	-24.910501	0.003704	0.000484	0.017082	0.003818	0.110001
86	-3097.098065	-24.957255	0.003609	0.000475	0.016578	0.003784	0.110001
87	-3097.098137	-25.002487	0.003514	0.000467	0.016095	0.003762	0.110000
88	-3097.098207	-25.046362	0.003421	0.000458	0.015658	0.003740	0.110000
89	-3097.098274	-25.088802	0.003329	0.000451	0.015351	0.003728	0.110001
90	-3097.098340	-25.129967	0.003239	0.000443	0.015050	0.003713	0.110000
91	-3097.098403	-25.169750	0.003150	0.000436	0.014800	0.003705	0.110000
92	-3097.098465	-25.208515	0.003062	0.000428	0.014510	0.003692	0.110000
93	-3097.098524	-25.245648	0.002975	0.000423	0.014251	0.003687	0.110000
94	-3097.098582	-25.281763	0.002891	0.000413	0.013993	0.003675	0.110000
95	-3097.098637	-25.316607	0.002806	0.000410	0.013832	0.003679	0.110000
96	-3097.098692	-25.350566	0.002726	0.000398	0.013574	0.003664	0.110000
97	-3097.098743	-25.383100	0.002643	0.000399	0.013442	0.003680	0.110000
98	-3097.098794	-25.415078	0.002568	0.000382	0.013127	0.003654	0.110000
99	-3097.098843	-25.445425	0.002486	0.000390	0.013029	0.003700	0.110000
100	-3097.098891	-25.475756	0.002418	0.000363	0.012561	0.003639	0.110000
101	-3097.098936	-25.504153	0.002333	0.000379	0.012577	0.003791	0.110000
102	-3097.098982	-25.532864	0.002275	0.000346	0.011420	0.003595	0.110000
103	-3097.099025	-25.559880	0.002188	0.000342	0.012208	0.003943	0.110000
104	-3097.099064	-25.584532	0.002134	0.000364	0.010665	0.003677	0.110000
105	-3097.099107	-25.610973	0.002064	0.000305	0.010797	0.003789	0.110000
106	-3097.099160	-25.644339	0.002009	0.000266	0.011080	0.003891	0.110000
107	-3097.099186	-25.660728	0.001923	0.000331	0.014662	0.004118	0.110000
108	-3097.099231	-25.688833	0.001877	0.000286	0.010443	0.003680	0.110000
109	-3097.099269	-25.712902	0.001808	0.000265	0.010668	0.003928	0.110000
110	-3097.099303	-25.734360	0.001744	0.000307	0.010839	0.003912	0.110000
111	-3097.099340	-25.757435	0.001689	0.000301	0.010571	0.003742	0.110000
112	-3097.099371	-25.776826	0.001635	0.000311	0.010837	0.003779	0.110000
113	-3097.099404	-25.797421	0.001587	0.000311	0.011591	0.003774	0.110000
114	-3097.099434	-25.816673	0.001663	0.000325	0.012242	0.003808	0.110000
115	-3097.099465	-25.835939	0.001973	0.000333	0.012378	0.003815	0.110000
116	-3097.099494	-25.854252	0.002266	0.000349	0.012487	0.003838	0.110000
117	-3097.099523	-25.872301	0.002238	0.000343	0.011776	0.003866	0.110000
118	-3097.099549	-25.888876	0.002202	0.000350	0.011796	0.003854	0.110000
119	-3097.099577	-25.906202	0.001918	0.000325	0.014155	0.003937	0.110000
120	-3097.099603	-25.922392	0.002011	0.000340	0.014497	0.003929	0.110000
121	-3097.099630	-25.939721	0.001895	0.000313	0.015656	0.004079	0.110000
122	-3097.099654	-25.954748	0.002114	0.000329	0.014609	0.004043	0.110000
123	-3097.099681	-25.971244	0.001848	0.000293	0.013835	0.004194	0.110000
124	-3097.099703	-25.985496	0.001826	0.000300	0.013938	0.004087	0.110000
125	-3097.099728	-26.000749	0.001571	0.000276	0.013883	0.004069	0.110000
126	-3097.099753	-26.016288	0.001106	0.000227	0.015260	0.004348	0.110000
127	-3097.099776	-26.030822	0.001099	0.000185	0.025513	0.005621	0.110000
128	-3097.099796	-26.043380	0.001079	0.000236	0.023802	0.005701	0.110000
129	-3097.099814	-26.054780	0.001057	0.000174	0.032265	0.006638	0.110000
130	-3097.099829	-26.064501	0.001035	0.000295	0.022929	0.005700	0.110000
131	-3097.099849	-26.076596	0.001007	0.000136	0.031961	0.006551	0.110000
132	-3097.099865	-26.086996	0.001269	0.000261	0.031044	0.006470	0.110000
133	-3097.099882	-26.097240	0.000956	0.000128	0.011442	0.004002	0.110000
134	-3097.099895	-26.105471	0.002318	0.000339	0.014491	0.004473	0.110000
135	-3097.099900	-26.108955	0.001736	0.000327	0.013931	0.004367	0.110000
136	-3097.099899	-26.108112	0.003834	0.000533	0.022657	0.004527	0.110000
137	-3097.099922	-26.122492	0.002525	0.000353	0.018711	0.004358	0.110000
138	-3097.099925	-26.124273	0.002010	0.000366	0.017169	0.004707	0.110000

139	-3097.099950	-26.140280	0.000884	0.000156	0.011874	0.004192	0.110000
140	-3097.099961	-26.146931	0.000869	0.000176	0.013687	0.004339	0.110000
141	-3097.099981	-26.159688	0.000816	0.000132	0.010906	0.003949	0.110000
142	-3097.099995	-26.168426	0.001199	0.000198	0.011785	0.003974	0.110000
143	-3097.100008	-26.176391	0.001240	0.000201	0.009981	0.003545	0.110000
144	-3097.100023	-26.185846	0.001247	0.000199	0.010071	0.003696	0.110000
145	-3097.100034	-26.192945	0.001225	0.000195	0.009664	0.003596	0.110000
146	-3097.100051	-26.203350	0.001188	0.000193	0.009523	0.003568	0.110000
147	-3097.100055	-26.206274	0.000952	0.000166	0.009392	0.001944	0.110000
148	-3097.100066	-26.212863	0.000668	0.000145	0.010373	0.004002	0.110001
149	-3097.100077	-26.219947	0.000880	0.000162	0.012028	0.004353	0.110000
150	-3097.100089	-26.227490	0.000512	0.000110	0.013176	0.003990	0.110000
151	-3097.100102	-26.235825	0.000528	0.000075	0.011231	0.004219	0.110000
152	-3097.100114	-26.243134	0.000502	0.000091	0.012656	0.004355	0.110000
153	-3097.100125	-26.250178	0.000524	0.000100	0.013590	0.004272	0.110000
154	-3097.100135	-26.256366	0.000448	0.000094	0.012992	0.004279	0.110000
155	-3097.100147	-26.263862	0.000416	0.000069	0.012317	0.004313	0.110000
156	-3097.100159	-26.271627	0.000399	0.000057	0.011804	0.004373	0.110000
157	-3097.100170	-26.278265	0.000381	0.000069	0.012295	0.004372	0.110000
158	-3097.100177	-26.282654	0.000373	0.000094	0.011389	0.004249	0.110000
159	-3097.100186	-26.288090	0.000356	0.000099	0.010729	0.004087	0.110000
160	-3097.100195	-26.293751	0.000342	0.000077	0.011527	0.004111	0.110000
161	-3097.100203	-26.298842	0.000329	0.000066	0.011046	0.004273	0.110000
162	-3097.100212	-26.304426	0.000305	0.000052	0.012332	0.004403	0.110000
163	-3097.100220	-26.309373	0.000291	0.000055	0.012333	0.004538	0.110000
164	-3097.100228	-26.314746	0.000272	0.000051	0.012656	0.004589	0.110000
165	-3097.100234	-26.318454	0.000267	0.000069	0.013900	0.004499	0.110000
166	-3097.100239	-26.321504	0.000449	0.000093	0.014380	0.004558	0.110000
167	-3097.100248	-26.327051	0.000486	0.000095	0.014432	0.004492	0.110000
168	-3097.100255	-26.331572	0.000426	0.000092	0.013854	0.004598	0.110000
169	-3097.100261	-26.335390	0.000387	0.000110	0.014682	0.004586	0.110000
170	-3097.100267	-26.339243	0.000595	0.000132	0.014363	0.004401	0.110000
171	-3097.100273	-26.343093	0.000683	0.000135	0.012278	0.004179	0.110000
172	-3097.100294	-26.356368	0.000538	0.000104	0.009743	0.003375	0.093795
173	-3097.100280	-26.347274	0.000462	0.000086	0.003866	0.000868	0.088683
174	-3097.100284	-26.349951	0.000463	0.000114	0.011243	0.003887	0.110003
175	-3097.100286	-26.351152	0.000617	0.000129	0.012431	0.004120	0.110000
176	-3097.100292	-26.355115	0.000242	0.000072	0.012511	0.003931	0.110000
177	-3097.100297	-26.357734	0.000741	0.000150	0.012202	0.004210	0.110000
178	-3097.100296	-26.357636	0.000848	0.000157	0.012372	0.004165	0.110000
179	-3097.100281	-26.347994	0.001915	0.000397	0.012614	0.004176	0.110000
180	-3097.100285	-26.350353	0.001944	0.000379	0.024291	0.004998	0.096662
181	-3097.100299	-26.359114	0.000385	0.000079	0.008072	0.001469	0.032657
182	-3097.100301	-26.360548	0.000479	0.000109	0.013681	0.004344	0.110000
183	-3097.100306	-26.363378	0.000301	0.000084	0.013077	0.004190	0.110000
184	-3097.100311	-26.366855	0.000304	0.000070	0.013454	0.004381	0.110000
185	-3097.100315	-26.368981	0.000396	0.000085	0.014340	0.004363	0.110000
186	-3097.100319	-26.371561	0.000460	0.000085	0.014087	0.003992	0.101633
187	-3097.100323	-26.374006	0.000387	0.000079	0.013030	0.003804	0.097622
188	-3097.100330	-26.378641	0.000341	0.000060	0.011346	0.003279	0.084245
189	-3097.100329	-26.377937	0.000232	0.000049	0.004240	0.001480	0.096801
190	-3097.100331	-26.379386	0.000213	0.000055	0.010013	0.004017	0.110001
191	-3097.100334	-26.381372	0.000260	0.000066	0.011146	0.003992	0.107149
192	-3097.100339	-26.384297	0.000424	0.000091	0.012332	0.004195	0.110000
193	-3097.100340	-26.385055	0.000434	0.000085	0.005423	0.001348	0.079592
194	-3097.100340	-26.384839	0.000587	0.000107	0.012154	0.003707	0.110001
195	-3097.100334	-26.381439	0.000460	0.000085	0.006493	0.001830	0.048839
196	-3097.100344	-26.387525	0.000192	0.000046	0.011588	0.003777	0.098785
197	-3097.100346	-26.388924	0.000387	0.000091	0.012124	0.003968	0.110000
198	-3097.100349	-26.390650	0.000363	0.000063	0.009951	0.003595	0.098106
199	-3097.100351	-26.392088	0.000175	0.000042	0.014335	0.004168	0.110000
200	-3097.100352	-26.392255	0.000203	0.000039	0.003693	0.001061	0.086537
201	-3097.100352	-26.392394	0.000433	0.000095	0.014994	0.004042	0.110001

202	-3097.100356	-26.395170	0.000336	0.000061	0.006882	0.002070	0.056163
203	-3097.100356	-26.394841	0.000392	0.000100	0.013933	0.004014	0.110000
204	-3097.100358	-26.396425	0.000344	0.000088	0.005262	0.002193	0.064629
205	-3097.100358	-26.396414	0.000860	0.000156	0.008975	0.002778	0.076246
206	-3097.100350	-26.391369	0.001729	0.000279	0.012914	0.004143	0.104593
207	-3097.100355	-26.394512	0.001514	0.000225	0.008019	0.001859	0.046339
208	-3097.100348	-26.389944	0.002419	0.000361	0.019519	0.004429	0.110000
209	-3097.100355	-26.394554	0.000924	0.000180	0.007352	0.002172	0.062367
210	-3097.100356	-26.394767	0.000702	0.000182	0.006978	0.002294	0.053635
211	-3097.100359	-26.396729	0.000615	0.000148	0.007163	0.002096	0.053927
212	-3097.100358	-26.396125	0.000753	0.000151	0.008701	0.002530	0.061444
213	-3097.100358	-26.396227	0.000707	0.000156	0.008129	0.002453	0.066546
214	-3097.100357	-26.395518	0.000817	0.000196	0.012037	0.003567	0.085607
215	-3097.100361	-26.398095	0.000681	0.000156	0.009425	0.002638	0.073186
216	-3097.100363	-26.399094	0.000132	0.000031	0.008552	0.002446	0.064605
217	-3097.100362	-26.398786	0.000457	0.000125	0.010869	0.004072	0.110000
218	-3097.100363	-26.399214	0.000198	0.000057	0.003940	0.001434	0.042207
219	-3097.100362	-26.398952	0.000459	0.000123	0.006043	0.002341	0.062496
220	-3097.100361	-26.398194	0.000606	0.000158	0.010654	0.004083	0.110000
221	-3097.100362	-26.398657	0.000672	0.000168	0.010791	0.003024	0.080465
222	-3097.100363	-26.399112	0.000536	0.000143	0.008866	0.001928	0.046966
223	-3097.100363	-26.399509	0.000365	0.000089	0.005069	0.001190	0.031697
224	-3097.100364	-26.399845	0.000214	0.000062	0.003863	0.001438	0.038273
225	-3097.100365	-26.400471	0.000274	0.000090	0.015351	0.004403	0.110000
226	-3097.100366	-26.401287	0.000170	0.000054	0.004252	0.001289	0.084260
227	-3097.100367	-26.401847	0.000328	0.000089	0.018157	0.004190	0.110000
228	-3097.100370	-26.403656	0.000136	0.000044	0.007691	0.001947	0.047853
229	-3097.100369	-26.403261	0.000217	0.000058	0.015290	0.004271	0.110000
230	-3097.100372	-26.405146	0.000223	0.000054	0.009637	0.003214	0.077363
231	-3097.100372	-26.404749	0.000255	0.000061	0.004408	0.001872	0.063497
232	-3097.100370	-26.403897	0.000461	0.000112	0.006354	0.002526	0.079773
233	-3097.100370	-26.403949	0.000467	0.000112	0.007543	0.001653	0.046066
234	-3097.100371	-26.404417	0.000349	0.000088	0.004396	0.000923	0.024446
235	-3097.100371	-26.404098	0.000229	0.000071	0.003227	0.001059	0.028848
236	-3097.100371	-26.404659	0.000246	0.000064	0.004076	0.001275	0.037834
237	-3097.100371	-26.404120	0.000347	0.000080	0.004953	0.001577	0.050615
238	-3097.100370	-26.403829	0.000391	0.000090	0.005843	0.001528	0.041854
239	-3097.100370	-26.403492	0.000474	0.000104	0.003270	0.001229	0.037424
240	-3097.100374	-26.406212	0.000474	0.000090	0.006232	0.001359	0.032567

```
**************************************************************
*  MAXIMUM NUMBER OF ITERATIONS REACHED - STOPPING IRC RUN  *
**************************************************************
```

```
---------------------------------------------------------------
                       IRC PATH SUMMARY
---------------------------------------------------------------
```

All gradients are in Eh/Bohr.

Step	E(Eh)	dE(kcal/mol)	max(|G|)	RMS(G)	SMW-Displ.
1	-3097.100374	-26.406212	0.000474	0.000090	-24.421489
2	-3097.100370	-26.403492	0.000474	0.000104	-24.388922
3	-3097.100370	-26.403829	0.000391	0.000090	-24.351498
4	-3097.100371	-26.404120	0.000347	0.000080	-24.309645
5	-3097.100371	-26.404659	0.000246	0.000064	-24.259030
6	-3097.100371	-26.404098	0.000229	0.000071	-24.221196
7	-3097.100371	-26.404417	0.000349	0.000088	-24.192347
8	-3097.100370	-26.403949	0.000467	0.000112	-24.167902
9	-3097.100370	-26.403897	0.000461	0.000112	-24.121836
10	-3097.100372	-26.404749	0.000255	0.000061	-24.042063
11	-3097.100372	-26.405146	0.000223	0.000054	-23.978566
12	-3097.100369	-26.403261	0.000217	0.000058	-23.901203

13	-3097.100370	-26.403656	0.000136	0.000044	-23.791203
14	-3097.100367	-26.401847	0.000328	0.000089	-23.743351
15	-3097.100366	-26.401287	0.000170	0.000054	-23.633350
16	-3097.100365	-26.400471	0.000274	0.000090	-23.549091
17	-3097.100364	-26.399845	0.000214	0.000062	-23.439091
18	-3097.100363	-26.399509	0.000365	0.000089	-23.400817
19	-3097.100363	-26.399112	0.000536	0.000143	-23.369120
20	-3097.100362	-26.398657	0.000672	0.000168	-23.322154
21	-3097.100361	-26.398194	0.000606	0.000158	-23.241689
22	-3097.100362	-26.398952	0.000459	0.000123	-23.131689
23	-3097.100363	-26.399214	0.000198	0.000057	-23.069193
24	-3097.100362	-26.398786	0.000457	0.000125	-23.026986
25	-3097.100363	-26.399094	0.000132	0.000031	-22.916986
26	-3097.100361	-26.398095	0.000681	0.000156	-22.852382
27	-3097.100357	-26.395518	0.000817	0.000196	-22.779196
28	-3097.100358	-26.396227	0.000707	0.000156	-22.693589
29	-3097.100358	-26.396125	0.000753	0.000151	-22.627044
30	-3097.100359	-26.396729	0.000615	0.000148	-22.565600
31	-3097.100356	-26.394767	0.000702	0.000182	-22.511673
32	-3097.100355	-26.394554	0.000924	0.000180	-22.458038
33	-3097.100348	-26.389944	0.002419	0.000361	-22.395672
34	-3097.100355	-26.394512	0.001514	0.000225	-22.285672
35	-3097.100350	-26.391369	0.001729	0.000279	-22.239332
36	-3097.100358	-26.396414	0.000860	0.000156	-22.134739
37	-3097.100358	-26.396425	0.000344	0.000088	-22.058493
38	-3097.100356	-26.394841	0.000392	0.000100	-21.993864
39	-3097.100356	-26.395170	0.000336	0.000061	-21.883864
40	-3097.100352	-26.392394	0.000433	0.000095	-21.827701
41	-3097.100352	-26.392255	0.000203	0.000039	-21.717701
42	-3097.100351	-26.392088	0.000175	0.000042	-21.631164
43	-3097.100349	-26.390650	0.000363	0.000063	-21.521163
44	-3097.100346	-26.388924	0.000387	0.000091	-21.423057
45	-3097.100344	-26.387525	0.000192	0.000046	-21.313057
46	-3097.100334	-26.381439	0.000460	0.000085	-21.214272
47	-3097.100340	-26.384839	0.000587	0.000107	-21.165433
48	-3097.100340	-26.385055	0.000434	0.000085	-21.055431
49	-3097.100339	-26.384297	0.000424	0.000091	-20.975839
50	-3097.100334	-26.381372	0.000260	0.000066	-20.865839
51	-3097.100331	-26.379386	0.000213	0.000055	-20.758690
52	-3097.100329	-26.377937	0.000232	0.000049	-20.648689
53	-3097.100330	-26.378641	0.000341	0.000060	-20.551888
54	-3097.100323	-26.374006	0.000387	0.000079	-20.467643
55	-3097.100319	-26.371561	0.000460	0.000085	-20.370021
56	-3097.100315	-26.368981	0.000396	0.000085	-20.268388
57	-3097.100311	-26.366855	0.000304	0.000070	-20.158388
58	-3097.100306	-26.363378	0.000301	0.000084	-20.048388
59	-3097.100301	-26.360548	0.000479	0.000109	-19.938388
60	-3097.100299	-26.359114	0.000385	0.000079	-19.828388
61	-3097.100285	-26.350353	0.001944	0.000379	-19.795731
62	-3097.100281	-26.347994	0.001915	0.000397	-19.699069
63	-3097.100296	-26.357636	0.000848	0.000157	-19.589069
64	-3097.100297	-26.357734	0.000741	0.000150	-19.479069
65	-3097.100292	-26.355115	0.000242	0.000072	-19.369068
66	-3097.100286	-26.351152	0.000617	0.000129	-19.259068
67	-3097.100284	-26.349951	0.000463	0.000114	-19.149068
68	-3097.100280	-26.347274	0.000462	0.000086	-19.039066
69	-3097.100294	-26.356368	0.000538	0.000104	-18.950383
70	-3097.100273	-26.343093	0.000683	0.000135	-18.856588
71	-3097.100267	-26.339243	0.000595	0.000132	-18.746588
72	-3097.100261	-26.335390	0.000387	0.000110	-18.636588
73	-3097.100255	-26.331572	0.000426	0.000092	-18.526588
74	-3097.100248	-26.327051	0.000486	0.000095	-18.416588
75	-3097.100239	-26.321504	0.000449	0.000093	-18.306588

76	-3097.100234	-26.318454	0.000267	0.000069	-18.196588
77	-3097.100228	-26.314746	0.000272	0.000051	-18.086588
78	-3097.100220	-26.309373	0.000291	0.000055	-17.976588
79	-3097.100212	-26.304426	0.000305	0.000052	-17.866588
80	-3097.100203	-26.298842	0.000329	0.000066	-17.756587
81	-3097.100195	-26.293751	0.000342	0.000077	-17.646587
82	-3097.100186	-26.288090	0.000356	0.000099	-17.536587
83	-3097.100177	-26.282654	0.000373	0.000094	-17.426587
84	-3097.100170	-26.278265	0.000381	0.000069	-17.316587
85	-3097.100159	-26.271627	0.000399	0.000057	-17.206587
86	-3097.100147	-26.263862	0.000416	0.000069	-17.096587
87	-3097.100135	-26.256366	0.000448	0.000094	-16.986587
88	-3097.100125	-26.250178	0.000524	0.000100	-16.876587
89	-3097.100114	-26.243134	0.000502	0.000091	-16.766586
90	-3097.100102	-26.235825	0.000528	0.000075	-16.656586
91	-3097.100089	-26.227490	0.000512	0.000110	-16.546586
92	-3097.100077	-26.219947	0.000880	0.000162	-16.436586
93	-3097.100066	-26.212863	0.000668	0.000145	-16.326586
94	-3097.100055	-26.206274	0.000952	0.000166	-16.216585
95	-3097.100051	-26.203350	0.001188	0.000193	-16.106585
96	-3097.100034	-26.192945	0.001225	0.000195	-15.996585
97	-3097.100023	-26.185846	0.001247	0.000199	-15.886584
98	-3097.100008	-26.176391	0.001240	0.000201	-15.776584
99	-3097.099995	-26.168426	0.001199	0.000198	-15.666584
100	-3097.099981	-26.159688	0.000816	0.000132	-15.556584
101	-3097.099961	-26.146931	0.000869	0.000176	-15.446584
102	-3097.099950	-26.140280	0.000884	0.000156	-15.336584
103	-3097.099925	-26.124273	0.002010	0.000366	-15.226583
104	-3097.099922	-26.122492	0.002525	0.000353	-15.116583
105	-3097.099899	-26.108112	0.003834	0.000533	-15.006583
106	-3097.099900	-26.108955	0.001736	0.000327	-14.896583
107	-3097.099895	-26.105471	0.002318	0.000339	-14.786583
108	-3097.099882	-26.097240	0.000956	0.000128	-14.676582
109	-3097.099865	-26.086996	0.001269	0.000261	-14.566582
110	-3097.099849	-26.076596	0.001007	0.000136	-14.456582
111	-3097.099829	-26.064501	0.001035	0.000295	-14.346581
112	-3097.099814	-26.054780	0.001057	0.000174	-14.236581
113	-3097.099796	-26.043380	0.001079	0.000236	-14.126581
114	-3097.099776	-26.030822	0.001099	0.000185	-14.016581
115	-3097.099753	-26.016288	0.001106	0.000227	-13.906581
116	-3097.099728	-26.000749	0.001571	0.000276	-13.796581
117	-3097.099703	-25.985496	0.001826	0.000300	-13.686580
118	-3097.099681	-25.971244	0.001848	0.000293	-13.576580
119	-3097.099654	-25.954748	0.002114	0.000329	-13.466580
120	-3097.099630	-25.939721	0.001895	0.000313	-13.356580
121	-3097.099603	-25.922392	0.002011	0.000340	-13.246580
122	-3097.099577	-25.906202	0.001918	0.000325	-13.136580
123	-3097.099549	-25.888876	0.002202	0.000350	-13.026579
124	-3097.099523	-25.872301	0.002238	0.000343	-12.916579
125	-3097.099494	-25.854252	0.002266	0.000349	-12.806579
126	-3097.099465	-25.835939	0.001973	0.000333	-12.696579
127	-3097.099434	-25.816673	0.001663	0.000325	-12.586579
128	-3097.099404	-25.797421	0.001587	0.000311	-12.476578
129	-3097.099371	-25.776826	0.001635	0.000311	-12.366578
130	-3097.099340	-25.757435	0.001689	0.000301	-12.256578
131	-3097.099303	-25.734360	0.001744	0.000307	-12.146578
132	-3097.099269	-25.712902	0.001808	0.000265	-12.036578
133	-3097.099231	-25.688833	0.001877	0.000286	-11.926578
134	-3097.099186	-25.660728	0.001923	0.000331	-11.816577
135	-3097.099160	-25.644339	0.002009	0.000266	-11.706577
136	-3097.099107	-25.610973	0.002064	0.000305	-11.596577
137	-3097.099064	-25.584532	0.002134	0.000364	-11.486576
138	-3097.099025	-25.559880	0.002188	0.000342	-11.376576

139	-3097.098982	-25.532864	0.002275	0.000346	-11.266576
140	-3097.098936	-25.504153	0.002333	0.000379	-11.156575
141	-3097.098891	-25.475756	0.002418	0.000363	-11.046575
142	-3097.098843	-25.445425	0.002486	0.000390	-10.936575
143	-3097.098794	-25.415078	0.002568	0.000382	-10.826575
144	-3097.098743	-25.383100	0.002643	0.000399	-10.716574
145	-3097.098692	-25.350566	0.002726	0.000398	-10.606574
146	-3097.098637	-25.316607	0.002806	0.000410	-10.496574
147	-3097.098582	-25.281763	0.002891	0.000413	-10.386574
148	-3097.098524	-25.245648	0.002975	0.000423	-10.276574
149	-3097.098465	-25.208515	0.003062	0.000428	-10.166573
150	-3097.098403	-25.169750	0.003150	0.000436	-10.056573
151	-3097.098340	-25.129967	0.003239	0.000443	-9.946573
152	-3097.098274	-25.088802	0.003329	0.000451	-9.836572
153	-3097.098207	-25.046362	0.003421	0.000458	-9.726572
154	-3097.098137	-25.002487	0.003514	0.000467	-9.616572
155	-3097.098065	-24.957255	0.003609	0.000475	-9.506571
156	-3097.097990	-24.910501	0.003704	0.000484	-9.396571
157	-3097.097914	-24.862310	0.003800	0.000492	-9.286570
158	-3097.097834	-24.812431	0.003899	0.000499	-9.176570
159	-3097.097752	-24.761238	0.003998	0.000507	-9.066570
160	-3097.097668	-24.708040	0.004105	0.000512	-8.956569
161	-3097.097580	-24.653082	0.004226	0.000513	-8.846568
162	-3097.097488	-24.595150	0.004367	0.000504	-8.736567
163	-3097.097390	-24.533638	0.004544	0.000521	-8.626567
164	-3097.097288	-24.469762	0.005060	0.000874	-8.516565
165	-3097.097259	-24.451743	0.004698	0.000792	-8.406565
166	-3097.097169	-24.395033	0.004827	0.000758	-8.296564
167	-3097.097075	-24.336427	0.004941	0.000813	-8.186564
168	-3097.096980	-24.276220	0.005079	0.000780	-8.076564
169	-3097.096880	-24.213788	0.005206	0.000836	-7.966564
170	-3097.096778	-24.150000	0.005359	0.000799	-7.856563
171	-3097.096668	-24.081021	0.005490	0.000865	-7.746562
172	-3097.096562	-24.013995	0.005635	0.000817	-7.636562
173	-3097.096449	-23.943553	0.005834	0.000798	-7.526562
174	-3097.096322	-23.863705	0.005974	0.000931	-7.416561
175	-3097.096210	-23.793520	0.006105	0.000811	-7.306560
176	-3097.096084	-23.714069	0.006316	0.000745	-7.196560
177	-3097.095932	-23.619177	0.006555	0.000782	-7.086559
178	-3097.095785	-23.526463	0.006804	0.000866	-6.976559
179	-3097.095630	-23.429381	0.006995	0.001044	-6.866558
180	-3097.095494	-23.344258	0.007211	0.000905	-6.756558
181	-3097.095339	-23.246958	0.007475	0.000949	-6.646557
182	-3097.095170	-23.140924	0.007697	0.001114	-6.536557
183	-3097.095022	-23.048028	0.007913	0.001029	-6.426556
184	-3097.094846	-22.937652	0.008104	0.001075	-6.316555
185	-3097.094676	-22.830460	0.008369	0.000989	-6.206554
186	-3097.094470	-22.701677	0.008680	0.001032	-6.096552
187	-3097.094268	-22.574637	0.009001	0.001112	-5.986552
188	-3097.094061	-22.444648	0.009281	0.001277	-5.876551
189	-3097.093875	-22.328276	0.009560	0.001217	-5.766551
190	-3097.093664	-22.195874	0.009836	0.001305	-5.656551
191	-3097.093452	-22.062964	0.010108	0.001286	-5.546549
192	-3097.093229	-21.922949	0.010438	0.001295	-5.436548
193	-3097.092991	-21.773423	0.010755	0.001431	-5.326547
194	-3097.092765	-21.631509	0.011060	0.001408	-5.216546
195	-3097.092517	-21.475690	0.011378	0.001481	-5.106545
196	-3097.092267	-21.319191	0.011695	0.001500	-4.996544
197	-3097.092003	-21.153612	0.012094	0.001557	-4.886543
198	-3097.091732	-20.983295	0.012436	0.001593	-4.776542
199	-3097.091449	-20.805464	0.012774	0.001640	-4.666542
200	-3097.091155	-20.621471	0.013117	0.001682	-4.556540
201	-3097.090850	-20.430006	0.013467	0.001729	-4.446540

202	-3097.090534	-20.231480	0.013822	0.001775	-4.336539	
203	-3097.090205	-20.025101	0.014183	0.001824	-4.226539	
204	-3097.089864	-19.810863	0.014549	0.001873	-4.116537	
205	-3097.089509	-19.588179	0.014922	0.001925	-4.006536	
206	-3097.089140	-19.356933	0.015300	0.001977	-3.896534	
207	-3097.088757	-19.116643	0.015684	0.002032	-3.786533	
208	-3097.088359	-18.867056	0.016075	0.002087	-3.676532	
209	-3097.087946	-18.607734	0.016455	0.002144	-3.566531	
210	-3097.087517	-18.338302	0.016849	0.002202	-3.456530	
211	-3097.087071	-18.058307	0.017252	0.002263	-3.346528	
212	-3097.086607	-17.767386	0.017663	0.002326	-3.236528	
213	-3097.086125	-17.464931	0.018081	0.002389	-3.126527	
214	-3097.085624	-17.150325	0.018506	0.002454	-3.016525	
215	-3097.085102	-16.823079	0.018936	0.002524	-2.906524	
216	-3097.084562	-16.483804	0.019367	0.002599	-2.796522	
217	-3097.083995	-16.128243	0.019773	0.002643	-2.686521	
218	-3097.083398	-15.753407	0.020179	0.002693	-2.576520	
219	-3097.082770	-15.359316	0.020617	0.002766	-2.466519	
220	-3097.082114	-14.948014	0.021071	0.002849	-2.356517	
221	-3097.081435	-14.521929	0.021529	0.002936	-2.246514	
222	-3097.080732	-14.080737	0.021986	0.003028	-2.136511	
223	-3097.080004	-13.623840	0.022438	0.003123	-2.026510	
224	-3097.079249	-13.150025	0.022884	0.003223	-1.916508	
225	-3097.078468	-12.659800	0.023319	0.003327	-1.806506	
226	-3097.077659	-12.152388	0.023734	0.003435	-1.696506	
227	-3097.076817	-11.624192	0.024121	0.003549	-1.586505	
228	-3097.075942	-11.074687	0.024471	0.003672	-1.476504	
229	-3097.075034	-10.505322	0.024770	0.003804	-1.366502	
230	-3097.074082	-9.908124	0.025001	0.003950	-1.256501	
231	-3097.073081	-9.279404	0.025147	0.004111	-1.146500	
232	-3097.072019	-8.613423	0.025190	0.004289	-1.036499	
233	-3097.070887	-7.902858	0.025103	0.004488	-0.926498	
234	-3097.069665	-7.136017	0.024847	0.004703	-0.816493	
235	-3097.068328	-6.296977	0.025126	0.004914	-0.706491	
236	-3097.066827	-5.355343	0.025032	0.005045	-0.596486	
237	-3097.065016	-4.218794	0.022961	0.004964	-0.486484	
238	-3097.062835	-2.850525	0.019365	0.004530	-0.376481	
239	-3097.060632	-1.467818	0.014788	0.003568	-0.266480	
240	-3097.059022	-0.457303	0.008372	0.002043	-0.156473	
241	-3097.058345	-0.032703	0.002344	0.000533	-0.046469	
242	-3097.058293	0.000000	0.000099	0.000024	0.000000	<= TS
243	-3097.058338	-0.028205	0.002297	0.000485	0.046469	
244	-3097.058726	-0.271691	0.007435	0.001411	0.156475	
245	-3097.059330	-0.651100	0.011607	0.002061	0.266478	
246	-3097.060012	-1.078589	0.014359	0.002467	0.376481	
247	-3097.060725	-1.526087	0.016021	0.002722	0.486482	
248	-3097.061460	-1.987438	0.017548	0.002895	0.596484	
249	-3097.062214	-2.460374	0.018915	0.003022	0.706486	
250	-3097.062980	-2.941503	0.020056	0.003123	0.816487	
251	-3097.063759	-3.429774	0.021041	0.003208	0.926490	
252	-3097.064551	-3.927065	0.021911	0.003282	1.036494	
253	-3097.065348	-4.427122	0.022692	0.003348	1.146495	
254	-3097.066161	-4.937202	0.023400	0.003408	1.256497	
255	-3097.066979	-5.450717	0.024043	0.003462	1.366499	
256	-3097.067804	-5.968158	0.024623	0.003511	1.476502	
257	-3097.068636	-6.490622	0.025144	0.003554	1.586503	
258	-3097.069477	-7.018081	0.025603	0.003591	1.696505	
259	-3097.070320	-7.547309	0.025996	0.003622	1.806506	
260	-3097.071167	-8.078877	0.026321	0.003645	1.916507	
261	-3097.072017	-8.611793	0.026574	0.003660	2.026511	
262	-3097.072867	-9.145211	0.026752	0.003668	2.136512	
263	-3097.073713	-9.676069	0.026852	0.003666	2.246514	
264	-3097.074563	-10.209582	0.026877	0.003657	2.356516	

265	-3097.075405	-10.737945	0.026819	0.003638	2.466518
266	-3097.076241	-11.262727	0.026681	0.003608	2.576520
267	-3097.077055	-11.773408	0.026455	0.003569	2.686524
268	-3097.077866	-12.282601	0.026153	0.003519	2.796527
269	-3097.078661	-12.781444	0.025774	0.003462	2.906528
270	-3097.079434	-13.266467	0.025319	0.003397	3.016531
271	-3097.080183	-13.736300	0.024775	0.003323	3.126532
272	-3097.080911	-14.192831	0.024145	0.003238	3.236533
273	-3097.081617	-14.635927	0.023437	0.003144	3.346536
274	-3097.082299	-15.063906	0.022654	0.003041	3.456537
275	-3097.082954	-15.475366	0.021798	0.002932	3.566539
276	-3097.083583	-15.869497	0.020870	0.002814	3.676539
277	-3097.084182	-16.245936	0.019873	0.002689	3.786540
278	-3097.084752	-16.603456	0.018814	0.002558	3.896543
279	-3097.085291	-16.941675	0.017695	0.002421	4.006543
280	-3097.085798	-17.259745	0.016525	0.002279	4.116546
281	-3097.086274	-17.558338	0.015308	0.002132	4.226548
282	-3097.086717	-17.836650	0.014058	0.001985	4.336550
283	-3097.087130	-18.095298	0.012780	0.001832	4.446550
284	-3097.087509	-18.333155	0.011499	0.001689	4.556552
285	-3097.087862	-18.554796	0.010214	0.001531	4.666553
286	-3097.088175	-18.751238	0.008982	0.001421	4.776555
287	-3097.088483	-18.944585	0.007741	0.001229	4.886555
288	-3097.088756	-19.115782	0.006837	0.001237	4.996557
289	-3097.089022	-19.282581	0.006352	0.000988	5.106557
290	-3097.089241	-19.420408	0.005794	0.000995	5.216559
291	-3097.089474	-19.566485	0.005345	0.000768	5.326559
292	-3097.089670	-19.689591	0.004899	0.000882	5.436559
293	-3097.089866	-19.812136	0.004528	0.000739	5.546560
294	-3097.090058	-19.932935	0.004121	0.000615	5.656562
295	-3097.090244	-20.049320	0.003746	0.000530	5.766563
296	-3097.090413	-20.155819	0.003258	0.000527	5.876564
297	-3097.090576	-20.257955	0.003016	0.000520	5.986564
298	-3097.090736	-20.358588	0.002809	0.000508	6.096564
299	-3097.090893	-20.457033	0.002641	0.000506	6.206564
300	-3097.091047	-20.553698	0.002501	0.000509	6.316565
301	-3097.091200	-20.649564	0.002387	0.000504	6.426565
302	-3097.091352	-20.745107	0.002290	0.000494	6.536565
303	-3097.091503	-20.839804	0.002205	0.000483	6.646567
304	-3097.091651	-20.932383	0.002128	0.000486	6.756567
305	-3097.091795	-21.022595	0.002058	0.000497	6.866568
306	-3097.091935	-21.110956	0.002023	0.000487	6.976568
307	-3097.092075	-21.198326	0.001974	0.000474	7.086569
308	-3097.092210	-21.283488	0.002027	0.000473	7.196570
309	-3097.092343	-21.366554	0.002099	0.000476	7.306571
310	-3097.092472	-21.447929	0.002129	0.000476	7.416571
311	-3097.092599	-21.527526	0.002164	0.000476	7.526572
312	-3097.092723	-21.605458	0.002186	0.000474	7.636572
313	-3097.092845	-21.681698	0.002205	0.000472	7.746573
314	-3097.092964	-21.756202	0.002231	0.000470	7.856574
315	-3097.093080	-21.828951	0.002257	0.000468	7.966574
316	-3097.093193	-21.899973	0.002281	0.000467	8.076575
317	-3097.093303	-21.969280	0.002302	0.000465	8.186576
318	-3097.093411	-22.036799	0.002324	0.000463	8.296576
319	-3097.093516	-22.102553	0.002345	0.000462	8.406576
320	-3097.093617	-22.166453	0.002364	0.000460	8.516576
321	-3097.093716	-22.228534	0.002381	0.000458	8.626576
322	-3097.093812	-22.288649	0.002395	0.000456	8.736576
323	-3097.093904	-22.346309	0.002404	0.000454	8.846577
324	-3097.093993	-22.402335	0.002405	0.000451	8.956577
325	-3097.094079	-22.456194	0.002404	0.000449	9.066578
326	-3097.094162	-22.507890	0.002383	0.000446	9.176578
327	-3097.094240	-22.557367	0.002329	0.000441	9.286578

328	-3097.094318	-22.606068	0.002189	0.000433	9.396578
329	-3097.094391	-22.651953	0.001867	0.000422	9.506579
330	-3097.094457	-22.693046	0.001586	0.000395	9.616579
331	-3097.094523	-22.734617	0.001792	0.000398	9.726579
332	-3097.094583	-22.772248	0.001745	0.000403	9.836579
333	-3097.094637	-22.806336	0.001939	0.000442	9.946580
334	-3097.094689	-22.839016	0.002408	0.000453	10.056580
335	-3097.094734	-22.867372	0.003106	0.000522	10.166580
336	-3097.094752	-22.878375	0.002644	0.000448	10.276580
337	-3097.094798	-22.907480	0.001346	0.000372	10.386582
338	-3097.094855	-22.942941	0.001069	0.000244	10.496583
339	-3097.094901	-22.972128	0.001018	0.000206	10.606583
340	-3097.094932	-22.991591	0.001903	0.000381	10.716584
341	-3097.094977	-23.019919	0.000947	0.000241	10.826584
342	-3097.095018	-23.045397	0.000789	0.000179	10.936585
343	-3097.095048	-23.064406	0.001412	0.000338	11.046586
344	-3097.095086	-23.088025	0.000994	0.000250	11.156586
345	-3097.095115	-23.106195	0.001047	0.000286	11.266586
346	-3097.095148	-23.126837	0.001237	0.000270	11.376587
347	-3097.095178	-23.145453	0.001249	0.000261	11.486587
348	-3097.095206	-23.163259	0.001380	0.000273	11.596587
349	-3097.095235	-23.181218	0.001339	0.000274	11.706587
350	-3097.095261	-23.197613	0.001182	0.000284	11.816587
351	-3097.095288	-23.214501	0.001257	0.000304	11.926587
352	-3097.095313	-23.230617	0.001287	0.000292	12.036587
353	-3097.095339	-23.246886	0.001155	0.000267	12.146588
354	-3097.095363	-23.261592	0.000818	0.000233	12.256588
355	-3097.095387	-23.276679	0.001143	0.000263	12.366588
356	-3097.095411	-23.292265	0.001292	0.000265	12.476588
357	-3097.095438	-23.308588	0.001260	0.000260	12.586588
358	-3097.095461	-23.323458	0.001113	0.000260	12.696588
359	-3097.095467	-23.327357	0.000975	0.000257	12.806589
360	-3097.095492	-23.343018	0.001035	0.000194	12.916589
361	-3097.095509	-23.353416	0.001194	0.000298	13.026590
362	-3097.095537	-23.370935	0.000943	0.000195	13.136590
363	-3097.095563	-23.387606	0.000865	0.000147	13.246590
364	-3097.095582	-23.399276	0.001104	0.000297	13.356591
365	-3097.095609	-23.416400	0.000934	0.000223	13.466591
366	-3097.095636	-23.433183	0.000792	0.000164	13.576591
367	-3097.095660	-23.447986	0.000732	0.000167	13.686591
368	-3097.095683	-23.462908	0.000734	0.000170	13.796591
369	-3097.095709	-23.478823	0.000661	0.000178	13.906592
370	-3097.095731	-23.492920	0.000937	0.000231	14.016592
371	-3097.095756	-23.508511	0.001125	0.000264	14.126592
372	-3097.095778	-23.522217	0.000827	0.000213	14.236592
373	-3097.095804	-23.538794	0.000651	0.000182	14.346592
374	-3097.095829	-23.553946	0.000716	0.000189	14.456592
375	-3097.095852	-23.568696	0.000819	0.000211	14.566592
376	-3097.095876	-23.583712	0.001076	0.000240	14.676593
377	-3097.095897	-23.597178	0.001168	0.000246	14.786593
378	-3097.095920	-23.611290	0.001062	0.000253	14.896593
379	-3097.095943	-23.625857	0.001124	0.000259	15.006593
380	-3097.095967	-23.640704	0.001051	0.000254	15.116593
381	-3097.095992	-23.656537	0.001077	0.000252	15.226593
382	-3097.096015	-23.671235	0.001103	0.000254	15.336594
383	-3097.096039	-23.686135	0.001082	0.000249	15.446594
384	-3097.096062	-23.700607	0.001065	0.000246	15.556594
385	-3097.096085	-23.715162	0.001099	0.000250	15.666594
386	-3097.096109	-23.729657	0.001110	0.000253	15.776594
387	-3097.096129	-23.742706	0.001118	0.000255	15.886594
388	-3097.096152	-23.757042	0.001165	0.000261	15.996595
389	-3097.096177	-23.772623	0.001173	0.000261	16.106595
390	-3097.096198	-23.786030	0.001123	0.000253	16.216595

391	-3097.096221	-23.800093	0.001180	0.000259	16.326595
392	-3097.096244	-23.814657	0.001183	0.000258	16.436595
393	-3097.096267	-23.828969	0.001171	0.000256	16.546595
394	-3097.096290	-23.843843	0.001168	0.000256	16.656595
395	-3097.096311	-23.857019	0.001091	0.000245	16.766596
396	-3097.096333	-23.870791	0.001204	0.000261	16.876596
397	-3097.096355	-23.884493	0.001284	0.000260	16.986596
398	-3097.096378	-23.898527	0.001221	0.000261	17.096596
399	-3097.096401	-23.912953	0.001257	0.000266	17.206596
400	-3097.096424	-23.927328	0.001295	0.000268	17.316596
401	-3097.096445	-23.940940	0.001253	0.000257	17.426596
402	-3097.096468	-23.955436	0.001306	0.000267	17.536596
403	-3097.096492	-23.970028	0.001291	0.000268	17.646596
404	-3097.096515	-23.984540	0.001321	0.000269	17.756596
405	-3097.096538	-23.999121	0.001276	0.000267	17.866597
406	-3097.096561	-24.013555	0.001347	0.000274	17.976597
407	-3097.096583	-24.027439	0.001252	0.000257	18.086597
408	-3097.096607	-24.042537	0.001361	0.000272	18.196597
409	-3097.096633	-24.058869	0.001322	0.000268	18.306597
410	-3097.096639	-24.062651	0.001025	0.000251	18.416597
411	-3097.096663	-24.077840	0.000826	0.000150	18.526598
412	-3097.096691	-24.095023	0.000742	0.000167	18.636598
413	-3097.096715	-24.110009	0.000947	0.000204	18.746599
414	-3097.096738	-24.124497	0.000887	0.000206	18.856599
415	-3097.096762	-24.139888	0.000798	0.000189	18.966599
416	-3097.096788	-24.155780	0.000950	0.000217	19.076599
417	-3097.096812	-24.171223	0.001001	0.000220	19.186600
418	-3097.096837	-24.186627	0.000995	0.000223	19.296600
419	-3097.096861	-24.202120	0.000955	0.000215	19.406600
420	-3097.096886	-24.217587	0.000931	0.000218	19.516600
421	-3097.096907	-24.230541	0.000747	0.000192	19.626600
422	-3097.096931	-24.245487	0.000728	0.000189	19.736600
423	-3097.096954	-24.260447	0.000841	0.000202	19.846601
424	-3097.096973	-24.272342	0.000834	0.000205	19.956601
425	-3097.096995	-24.285630	0.000803	0.000201	20.066601
426	-3097.097015	-24.298626	0.000781	0.000203	20.176601
427	-3097.097036	-24.311448	0.000764	0.000203	20.286601
428	-3097.097056	-24.324083	0.000777	0.000204	20.396601
429	-3097.097068	-24.331763	0.000738	0.000202	20.506601
430	-3097.097098	-24.350739	0.000774	0.000204	20.616601
431	-3097.097119	-24.363650	0.000822	0.000208	20.726601
432	-3097.097137	-24.375157	0.000738	0.000198	20.836601
433	-3097.097158	-24.388352	0.000760	0.000200	20.946601
434	-3097.097181	-24.402507	0.000809	0.000200	21.056602
435	-3097.097184	-24.404603	0.000610	0.000159	21.166602
436	-3097.097204	-24.416879	0.000620	0.000127	21.276603
437	-3097.097225	-24.430191	0.000734	0.000196	21.386603
438	-3097.097247	-24.443894	0.000931	0.000211	21.496604
439	-3097.097268	-24.457375	0.000906	0.000204	21.606604
440	-3097.097290	-24.471171	0.000877	0.000200	21.716604
441	-3097.097312	-24.484845	0.000877	0.000202	21.826604
442	-3097.097334	-24.498554	0.000895	0.000205	21.936604
443	-3097.097356	-24.512253	0.000910	0.000206	22.046604
444	-3097.097378	-24.526058	0.000921	0.000207	22.156604
445	-3097.097400	-24.539780	0.000933	0.000209	22.266605
446	-3097.097420	-24.552839	0.000946	0.000212	22.376605
447	-3097.097444	-24.567644	0.000961	0.000215	22.486605
448	-3097.097466	-24.581703	0.000966	0.000216	22.596605
449	-3097.097489	-24.595683	0.000975	0.000218	22.706605
450	-3097.097510	-24.608954	0.000985	0.000220	22.816605
451	-3097.097532	-24.622999	0.001000	0.000223	22.926605
452	-3097.097555	-24.637121	0.001011	0.000225	23.036605
453	-3097.097577	-24.651219	0.001017	0.000224	23.146606

454	-3097.097601	-24.665892	0.001024	0.000224	23.256606
455	-3097.097621	-24.678722	0.000994	0.000227	23.366606
456	-3097.097644	-24.692939	0.001053	0.000228	23.476606
457	-3097.097666	-24.706808	0.001054	0.000230	23.586606
458	-3097.097689	-24.721462	0.001065	0.000232	23.696606
459	-3097.097711	-24.735137	0.001050	0.000229	23.806607
460	-3097.097732	-24.748427	0.001033	0.000236	23.916607
461	-3097.097753	-24.761654	0.001054	0.000227	24.026607
462	-3097.097775	-24.775662	0.001089	0.000239	24.136607
463	-3097.097796	-24.788580	0.001053	0.000227	24.246607
464	-3097.097816	-24.801014	0.001059	0.000243	24.356607
465	-3097.097838	-24.814649	0.001090	0.000234	24.466608
466	-3097.097846	-24.819782	0.001109	0.000244	24.576608
467	-3097.097878	-24.840164	0.001151	0.000246	24.686608
468	-3097.097898	-24.852686	0.001064	0.000237	24.796608
469	-3097.097918	-24.865056	0.001084	0.000246	24.906608
470	-3097.097939	-24.878214	0.001071	0.000230	25.016608
471	-3097.097957	-24.889897	0.001113	0.000244	25.126608
472	-3097.097978	-24.902927	0.001084	0.000239	25.236608
473	-3097.097996	-24.914157	0.001071	0.000230	25.346608
474	-3097.098015	-24.926058	0.001126	0.000251	25.456608
475	-3097.098034	-24.937999	0.000980	0.000203	25.566609
476	-3097.098052	-24.949414	0.001036	0.000241	25.676609
477	-3097.098071	-24.960951	0.001040	0.000221	25.786609
478	-3097.098086	-24.970660	0.001116	0.000257	25.896609
479	-3097.098105	-24.982531	0.001004	0.000212	26.006609
480	-3097.098122	-24.993086	0.001046	0.000245	26.116609
481	-3097.098139	-25.003945	0.001036	0.000228	26.226609
482	-3097.098155	-25.013595	0.001101	0.000248	26.336609
483	-3097.098172	-25.024398	0.001019	0.000224	26.446610

SUGGESTED CITATIONS FOR THIS RUN

Below you find a list of papers that are relevant to this ORCA run
We neither can nor want to force you to cite these papers, but we appreciate if you do
You receive ORCA, which is the product of decades of hard work by many enthusiastic individuals, for free
The only thing we kindly ask in return is that you cite our papers,
We deeply appreciate it, if you show your appreciation for ORCA by not just citing the generic ORCA reference.

Please note that relegating all ORCA citations to the supporting information does *not* help us.
SI sections are not indexed - citations you put there will not count into any citation statistics
But we need these citations in order to attract the funding resources that allow us to do what we are doing

Therefore, if you are a happy ORCA user, please consider citing a few of the papers listed below in the main body of your paper

In addition to the list printed below, the program has created the file Me-IRC.bibtex that contains the list in bibtex format
You can import this file easily into all common literature databanks and citation aid programs

It goes without saying that in many instances, there are alternative algorithms to achieve similar
results as the ones you have gotten from ORCA. It is, of course, also the case that in some instances
ORCA just re-implements algorithms worked out by others. We are fully aware of that and we are also

fully appreciative of our colleagues work. Hence this citation list should not be read as indicating
that the listed papers, which are focused on our own work, are the only ones worth citing. It simply
meant to make it easier for users to cite ORCA specific papers. It is not a substitute for doing your
own literature research and citing the relevant literature in a scientifically appropriate manner.

List of essential papers. We consider these as the minimum necessary citations

1. Neese, F.
   Software update: the ORCA program system, version 6.0
   WIRES Comput. Molec. Sci. 2025 15(1), e70019
   doi.org/10.1002/wcms.70019

List of papers to cite with high priority. The work reported in these papers was absolutely necessary for this run to complete.
Our perspective: the developers of density functionals and basis sets usually get cited in chemistry papers
Good! But without the algorithms to do something with them, the functionals or basis sets would not do anything.
Hence, in our opinion, the algorithm design and method developments papers are equally worthy of getting cited

1. Page, M.; Doubleday, C.; McIver, J.
   Following steepest descent reaction paths. The use of higher energy derivatives with abinitio electronic structure methods
   J. Chem. Phys. 1990 93(8), 5634-5642
   doi.org/10.1063/1.459634
2. Neese, F.
   An improvement of the resolution of the identity approximation for the formation of the Coulomb matrix
   J. Comp. Chem. 2003 24(14), 1740-1747
   doi.org/10.1002/jcc.10318
3. Hratchian, H.; Schlegel, B.
   Using Hessian Updating To Increase the Efficiency of a Hessian Based Predictor-Corrector Reaction Path Following Method
   J. Chem. Theory Comput. 2005 1(1), 61-69
   doi.org/10.1021/ct0499783
4. Neese, F.; Wennmohs, F.; Hansen, A.; Becker, U.
   Efficient, approximate and parallel Hartree-Fock and hybrid DFT calculations. A 'chain-of-spheres' algorithm for the Hartree-Fock exchange
   Chem. Phys. 2009 356(1-3), 98-109
   doi.org/10.1016/j.chemphys.2008.10.036
5. Grimme, S.; Antony, J.; Ehrlich, S.; Krieg, H.
   A consistent and accurate ab initio parametrization of density functional dispersion correction (DFT-D) for the 94 elements H-Pu
   J. Chem. Phys. 2010 132 , 154104
   doi.org/10.1063/1.3382344
6. Grimme, S.; Ehrlich, S.; Goerigk, L.
   Effect of the damping function in dispersion corrected density functional theory
   J. Comput. Chem. 2011 32(7), 1456-1465
   doi.org/10.1002/jcc.21759
7. Garcia-Rates, M.; Neese, F.
   Effect of the Solute Cavity on the Solvation Energy and its Derivatives within the Framework of the Gaussian Charge Scheme
   J. Comput. Chem. 2020 41 , 922-939
   doi.org/10.1002/jcc.26139
8. Helmich-Paris, B.; de Souza, B.; Neese, F.; Izsák, R.
   An improved chain of spheres for exchange algorithm
   J. Chem. Phys. 2021 155(10), 104109
   doi.org/10.1063/5.0058766

```
  9. Neese, F.
     The SHARK Integral Generation and Digestion System
     J. Comp. Chem. 2022 44(3), 381
     doi.org/10.1002/jcc.26942

List of suggested additional citations. These are papers that are important in the
'surrounding' of
of this run, or papers that preceded the highly important papers. If you like your results we
are grateful for a citation.

  1. Izsak, R.; Neese, F.
     An overlap fitted chain of spheres exchange method
     J. Chem. Phys. 2011 135 , 144105
     doi.org/10.1063/1.3646921
  2. Izsak, R.; Hansen, A.; Neese, F.
     The resolution of identity and chain of spheres approximations for the LPNO-CCSD singles
Fock term
     Molec. Phys. 2012 110 , 2413-2417
     doi.org/10.1080/00268976.2012.687466
  3. Neese, F.
     The ORCA program system
     WIRES Comput. Molec. Sci. 2012 2(1), 73-78
     doi.org/10.1002/wcms.81
  4. Izsak, R.; Neese, F.; Klopper, W.
     Robust fitting techniques in the chain of spheres approximation to the Fock exchange: The
role of the complementary space
     J. Chem. Phys. 2013 139 ,
     doi.org/10.1063/1.4819264
  5. Neese, F.
     Software update: the ORCA program system, version 4.0
     WIRES Comput. Molec. Sci. 2018 8(1), 1-6
     doi.org/10.1002/wcms.1327
  6. Neese, F.; Wennmohs, F.; Becker, U.; Riplinger, C.
     The ORCA quantum chemistry program package
     J. Chem. Phys. 2020 152(22), 224108
     doi.org/10.1063/5.0004608
  7. Neese, F.
     Software update: The ORCA program system—Version 5.0
     WIRES Comput. Molec. Sci. 2022 12(1), e1606
     doi.org/10.1002/wcms.1606

List of optional additional citations

  1. Neese, F.
     Approximate second-order SCF convergence for spin unrestricted wavefunctions
     Chem. Phys. Lett. 2000 325(1-3), 93-98
     doi.org/10.1016/s0009-2614(00)00662-x

Timings for individual modules:

Sum of individual times          ...   130927.988 sec (=2182.133 min)
Startup calculation              ...     3401.878 sec (=  56.698 min)   2.6 %
SCF iterations                   ...    77874.941 sec (=1297.916 min)  59.5 %
Property calculations            ...        0.439 sec (=   0.007 min)   0.0 %
SCF Gradient evaluation          ...    49650.731 sec (= 827.512 min)  37.9 %
                             ****ORCA TERMINATED NORMALLY****
TOTAL RUN TIME: 1 days 12 hours 41 minutes 47 seconds 331 msec
'Me-IRC.bibtex' -> '/hsnfs/users/barasits/Tunneling_selenoxide/Me/IRC/Me-IRC.bibtex'
'Me-IRC.cpcm' -> '/hsnfs/users/barasits/Tunneling_selenoxide/Me/IRC/Me-IRC.cpcm'
'Me-IRC.cpcm_corr' -> '/hsnfs/users/barasits/Tunneling_selenoxide/Me/IRC/Me-IRC.cpcm_corr'
'Me-IRC.densities' -> '/hsnfs/users/barasits/Tunneling_selenoxide/Me/IRC/Me-IRC.densities'
'Me-IRC.densitiesinfo' -> '/hsnfs/users/barasits/Tunneling_selenoxide/Me/IRC/Me-
IRC.densitiesinfo'
```

```
'Me-IRC.engrad' -> '/hsnfs/users/barasits/Tunneling_selenoxide/Me/IRC/Me-IRC.engrad'
'Me-IRC.err' -> '/hsnfs/users/barasits/Tunneling_selenoxide/Me/IRC/Me-IRC.err'
'Me-IRC.gbw' -> '/hsnfs/users/barasits/Tunneling_selenoxide/Me/IRC/Me-IRC.gbw'
'Me-IRC_IRC_B.cpcm' -> '/hsnfs/users/barasits/Tunneling_selenoxide/Me/IRC/Me-IRC_IRC_B.cpcm'
'Me-IRC_IRC_B.cpcm_corr' -> '/hsnfs/users/barasits/Tunneling_selenoxide/Me/IRC/Me-
IRC_IRC_B.cpcm_corr'
'Me-IRC_IRC_B.densities' -> '/hsnfs/users/barasits/Tunneling_selenoxide/Me/IRC/Me-
IRC_IRC_B.densities'
'Me-IRC_IRC_B.densitiesinfo' -> '/hsnfs/users/barasits/Tunneling_selenoxide/Me/IRC/Me-
IRC_IRC_B.densitiesinfo'
'Me-IRC_IRC_B.gbw' -> '/hsnfs/users/barasits/Tunneling_selenoxide/Me/IRC/Me-IRC_IRC_B.gbw'
'Me-IRC_IRC_B.smd' -> '/hsnfs/users/barasits/Tunneling_selenoxide/Me/IRC/Me-IRC_IRC_B.smd'
'Me-IRC_IRC_B.smd.grd' -> '/hsnfs/users/barasits/Tunneling_selenoxide/Me/IRC/Me-
IRC_IRC_B.smd.grd'
'Me-IRC_IRC_B_trj.xyz' -> '/hsnfs/users/barasits/Tunneling_selenoxide/Me/IRC/Me-
IRC_IRC_B_trj.xyz'
'Me-IRC_IRC_B.xyz' -> '/hsnfs/users/barasits/Tunneling_selenoxide/Me/IRC/Me-IRC_IRC_B.xyz'
'Me-IRC_IRC_F.cpcm' -> '/hsnfs/users/barasits/Tunneling_selenoxide/Me/IRC/Me-IRC_IRC_F.cpcm'
'Me-IRC_IRC_F.cpcm_corr' -> '/hsnfs/users/barasits/Tunneling_selenoxide/Me/IRC/Me-
IRC_IRC_F.cpcm_corr'
'Me-IRC_IRC_F.densities' -> '/hsnfs/users/barasits/Tunneling_selenoxide/Me/IRC/Me-
IRC_IRC_F.densities'
'Me-IRC_IRC_F.densitiesinfo' -> '/hsnfs/users/barasits/Tunneling_selenoxide/Me/IRC/Me-
IRC_IRC_F.densitiesinfo'
'Me-IRC_IRC_F.gbw' -> '/hsnfs/users/barasits/Tunneling_selenoxide/Me/IRC/Me-IRC_IRC_F.gbw'
'Me-IRC_IRC_F.smd' -> '/hsnfs/users/barasits/Tunneling_selenoxide/Me/IRC/Me-IRC_IRC_F.smd'
'Me-IRC_IRC_F.smd.grd' -> '/hsnfs/users/barasits/Tunneling_selenoxide/Me/IRC/Me-
IRC_IRC_F.smd.grd'
'Me-IRC_IRC_F_trj.xyz' -> '/hsnfs/users/barasits/Tunneling_selenoxide/Me/IRC/Me-
IRC_IRC_F_trj.xyz'
'Me-IRC_IRC_Full_trj.xyz' -> '/hsnfs/users/barasits/Tunneling_selenoxide/Me/IRC/Me-
IRC_IRC_Full_trj.xyz'
'Me-IRC_IRC_F.xyz' -> '/hsnfs/users/barasits/Tunneling_selenoxide/Me/IRC/Me-IRC_IRC_F.xyz'
'Me-IRC.job' -> '/hsnfs/users/barasits/Tunneling_selenoxide/Me/IRC/Me-IRC.job'
'Me-IRC.property.txt' -> '/hsnfs/users/barasits/Tunneling_selenoxide/Me/IRC/Me-
IRC.property.txt'
'Me-IRC.smd' -> '/hsnfs/users/barasits/Tunneling_selenoxide/Me/IRC/Me-IRC.smd'
'Me-IRC.smd.grd' -> '/hsnfs/users/barasits/Tunneling_selenoxide/Me/IRC/Me-IRC.smd.grd'
'TS-Me_Compound_2.hess' -> '/hsnfs/users/barasits/Tunneling_selenoxide/Me/IRC/TS-
Me_Compound_2.hess'
cp: cannot stat 'Me-IRC.asa.inp': No such file or directory
cp: missing destination file operand after '/hsnfs/users/barasits/Tunneling_selenoxide/Me/IRC/'
Try 'cp --help' for more information.
cp: cannot stat 'Me-IRC.pc.tmp': No such file or directory
cp: cannot stat 'Me-IRC.QM2.pc.tmp': No such file or directory
```

IRC output for R = H

```
! Job execution by suborca V 10.06 !
Job started from head2.mpi-muelheim.mpg.de, running
/hsnfs/software/orca/orca_current_directory/orca_current_20251216/orca on xe30th96.mpi-
muelheim.mpg.de in /usr/scratch/barasits/H-IRC__26969133 (/bin/bash)
Job execution start: Tue Dec 16 12:03:49 PM CET 2025
Shared library path: /hsnfs/software/openmpi-
4.1.0/lib:/hsnfs/software/orca/orca_current_directory/orca_current_20251216:/hsnfs/software/the
mis/llvm-14.0.1/lib:/hsnfs/software/orca/orca_stable:/usr/users/openmpi-
2.0.4/lib:/opt/intel/lib/intel64:/opt/intel/mkl/lib/intel64
SLURM Job ID is: 26969133
SLURM_JOB_NODELIST: xe30th96
Maxcore enforced by SLURM: 9600 MB per core
Expanded nodelist (1 nodes):
xe30th96

                                 *****************
                                 * O   R   C   A *
                                 *****************

                                            #,
                                            ###
                                            ####
                                            #####
                                            ######
                                           ########,
                                     ,,################,,,,,
                               ,,#################################,,
                          ,,##########################################,,
                       ,#########################################, ''#####,
                    ,#############################################,,   '####,
                  ,##################################################,,,,####,
                ,###########''''           '''''###############################
              ,#####''   ,,,,##########,,,,          '''####'''          '####
            ,##' ,,,,###########################,,,                        '##
           ' ,,###''''                  '''############,,,
         ,,##''                                '''############,,,,        ,,,,,,###''
      ,#''                                            '''#######################'''
     '                                                          ''''####''''
             ,#######,   #######,   ,#######,      ##
            ,#'     '#,  ##    ##  ,#'     '#,    #''#        ,####,    ,#,
            ##       ##  ##   ,#'  ##            #'  '#       #'       ,# #
            ##       ##  #######   ##           ,######,      #####,      #
            '#,     ,#'  ##    ##  '#,     ,#' ,#      #,     #,   #      #
             '#######'   ##     ##  '#######'  #'      '#     '####' #    #



                ##########################################################
                #                        -***-                           #
                #          Department of theory and spectroscopy         #
                #                                                        #
                #                      Frank Neese                       #
                #                                                        #
                #     Directorship, Architecture, Infrastructure         #
                #                    SHARK, DRIVERS                      #
                #        Core code/Algorithms in most modules            #
                #                                                        #
                #        Max Planck Institute fuer Kohlenforschung       #
                #                Kaiser Wilhelm Platz 1                  #
                #                 D-45470 Muelheim/Ruhr                  #
                #                      Germany                           #
                #                                                        #
                #                  All rights reserved                   #
```

```
                #                         -***-                            #
                ##########################################################


                         Program Version 6.1.x  -  CURRENT   -
                                (GIT: $2a5ddc4e$)
                ($2025-12-15 18:08:28 +0100 $)

                  !                     WARNING:                          !
                  !         This is a DEVELOPMENT version                 !

                         (BUILD: $Tue 16 Dec 2025 12:05:34 AM CET$)


 With contributions from (in alphabetic order):
[Max-Planck-Institut fuer Kohlenforschung]
  Daniel Aravena         : Magnetic Suceptibility
  Michael Atanasov       : Ab Initio Ligand Field Theory (pilot matlab implementation)
  Alexander A. Auer      : GIAO ZORA, VPT2 properties, NMR spectrum
  Ute Becker             : All parallelization in ORCA, NUMFREQ, NUMCALC
  Giovanni Bistoni       : ED, misc. LED, open-shell LED, HFLD
  Dmytro Bykov           : pre 5.0 version of the SCF Hessian
  Marcos Casanova-Páez   : Triplet and SCS-CIS(D). UHF-(DLPNO)-IP/EA/STEOM-CCSD. UHF-CVS-
IP/STEOM-CCSD
  Vijay G. Chilkuri      : MRCI spin determinant printing, contributions to CSF-ICE
  Pauline Colinet        : FMM embedding
  Dipayan Datta          : RHF DLPNO-CCSD density
  Achintya Kumar Dutta   : EOM-CC, STEOM-CC
  Nicolas Foglia         : Exact transition moments, OPA infrastructure, MCD improvements
  Dmitry Ganyushin       : Spin-Orbit,Spin-Spin,Magnetic field MRCI
  Miquel Garcia-Rates    : C-PCM and meta-GGA Hessian, CCSD/C-PCM, Gaussian charge scheme
  Tiago L. C. Gouveia    : GS-ROHF, GS-ROCIS
  Yang Guo               : DLPNO-NEVPT2, F12-NEVPT2, CIM, IAO-localization
  Andreas Hansen         : Spin unrestricted coupled pair/coupled cluster methods
  Ingolf Harden          : AUTO-CI MPn and infrastructure
  Benjamin Helmich-Paris : MC-RPA, TRAH-(SCF,CASSCF), AVAS, COSX integrals, SCF dyn. polar.,
MC-PDFT, srDFT
  Lee Huntington         : MR-EOM, pCC
  Robert Izsak           : Overlap fitted RIJCOSX, COSX-SCS-MP3, EOM
  Riya Kayal             : Wick's Theorem for AUTO-CI, AUTO-CI UHF-CCSDT
  Emily Kempfer          : AUTO-CI RHF CISDT and CCSDT, approximate NEVPT4
  Christian Kollmar      : KDIIS, OOCD, Brueckner-CCSD(T), CCSD density, CASPT2, CASPT2-K,
improved NEVPT2
  Axel Koslowski         : Symmetry handling
  Simone Kossmann        : meta-GGA functionals, TD-DFT gradient, OOMP2, (MP2 Hessian;
deprecated post 5.0)
  Lucas Lang             : DCDCAS, Hyperfine gauge corrections, ICE-SOC+SSC
  Marvin Lechner         : AUTO-CI (C++ implementation), FIC-MRCC
  Spencer Leger          : CASSCF response
  Dagmar Lenk            : GEPOL surface, SMD, ORCA-2-JSON
  Dimitrios Liakos       : Extrapolation schemes; Compound Job, Property file
  Dimitrios Manganas     : Further ROCIS development; embedding schemes. LFT, Crystal Embedding
  Hagen Neugebauer       : wr2SCAN, Native XTB
  Dimitrios Pantazis     : SARC Basis sets
  Anastasios Papadopoulos: AUTO-CI, single reference methods and gradients
  Taras Petrenko         : pre 6.0 DFT Hessian and TD-DFT gradient,  ECA, NRVS
  Petra Pikulova         : Analytic Raman intensities
  Peter Pinski           : DLPNO-MP2, DLPNO-MP2 Gradient
  Shashank Vittal Rao    : ES-AILFT, MagRelax
  Christoph Reimann      : Effective Core Potentials
  Marius Retegan         : Local ZFS, SOC
  Christoph Riplinger    : Optimizer, TS searches, QM/MM, DLPNO-CCSD(T), (RO)-DLPNO pert.
Triples
```

```
  Michael Roemelt        : Original ROCIS implementation, recursive CI coupling coefficients
  Masaaki Saitow         : Open-shell DLPNO-CCSD energy and density
  Barbara Sandhoefer     : DKH picture change effects
  Yorick L. A. Schmerwitz: GMF and freeze-and-release deltaSCF, NEB S-IDPP initial path
  Kantharuban Sivalingam : CASSCF convergence/infrastructure, NEVPT2, NEVPT3, NEVPT4(SD), FIC-
MRCI and CEPA variants
  Bernardo de Souza      : ESD, SOC TD-DFT
  Georgi L. Stoychev     : AutoAux, RI-MP2 NMR, DLPNO-MP2 response, X2C
  Van Anh Tran           : RI-MP2 g-tensors
  Willem Van den Heuvel  : Paramagnetic NMR
  Zikuan Wang            : NOTCH, Electric field optimization
  Frank Wennmohs         : Technical directorship and infrastructure
  Hang Xu                : AUTO-CI-Response properties

[FACCTs GmbH]
  Markus Bursch, Nicolas Foglia, Miquel Garcia-Rates, Ingolf Harden, Hagen Neugebauer,
Anastasios Papadopoulos,
  Christoph Riplinger, Bernardo de Souza, Georgi L. Stoychev

  APM, various basis sets, CI-OPT, improved COSX, DLPNO-Multilevel,
  DOCKER, DRACO, updates on ESD, Fragmentator, GOAT, IRC, LR-CPCM, L-BFGS, MBIS, meta-GGA TD-
DFT gradient, ML-optimized integration grids,
  MM, NACMEs, nearIR, NEB, NEB-TS, NL-DFT gradient (VV10), 2- and 3-layer-ONIOM, interface
openCOSMO-RS, QMMM,
  Crystal-QMMM, RESP, rigid body optimization, SF, symmetry and pop. for TD-DFT, various
functionals, SOLVATOR

[Other institutions]
  V. Asgeirsson          : NEB
  Christoph Bannwarth    : sTDA-DFT, sTD-DFT, PBEh-3c, B97-3c, D3
  Giovanni Bistoni       : ETS/NOCV, ADLD/ADEX, COVALED
  Martin Brehm           : Molecular dynamics
  Ronald Cardenas        : ETS/NOCV
  Martina Colucci        : COVALED
  Sebastian Ehlert       : rSCAN, r2SCAN, r2SCAN-3c, D4, dhf basis sets
  Marvin Friede          : D4 for Fr, Ra, Ac-Lr
  Lars Goerigk           : TD-DFT with DH, B97 family of functionals
  Stefan Grimme          : VdW corrections, initial TS optimization, DFT functionals, gCP,
sTDA/sTD-DF
  Waldemar Hujo          : DFT-NL
  H. Jonsson             : NEB
  Holger Kruse           : gCP
  Marcel Mueller         : wB97X-3c, vDZP basis set
  Gianluca Regni         : ADLD/ADEX
  Tobias Risthaus        : pre 6.0 range-separated hybrid DFT and stability analysis
  Lukas Wittmann         : regularized MP2, r2SCAN double-hybrids, wr2SCAN

We gratefully acknowledge several colleagues who have allowed us to
interface, adapt or use parts of their codes:
  Ed Valeev, F. Pavosevic, A. Kumar             : LibInt (2-el integral package), F12 methods
  Garnet Chan, S. Sharma, J. Yang, R. Olivares  : DMRG
  Ulf Ekstrom                                   : XCFun DFT Library
  Mihaly Kallay                                 : mrcc  (arbitrary order and MRCC methods)
  Frank Weinhold                                : gennbo (NPA and NBO analysis)
  Simon Mueller                                 : openCOSMO-RS
  Christopher J. Cramer and Donald G. Truhlar   : smd solvation model
  S Lehtola, MJT Oliveira, MAL Marques          : LibXC Library
  Liviu Ungur et al                             : ANISO software


 Your calculation uses the libint2 library for the computation of 2-el integrals
 For citations please refer to: http://libint.valeyev.net
```

```
 Your ORCA version has been built with support for libXC version: 7.0.0
 For citations please refer to: https://libxc.gitlab.io

 This ORCA versions uses:
   CBLAS   interface :  Fast vector & matrix operations
   LAPACKE interface :  Fast linear algebra routines
   SCALAPACK package :  Parallel linear algebra routines
   Shared memory     :  Shared parallel matrices
   BLAS/LAPACK       :  OpenBLAS 0.3.26  USE64BITINT DYNAMIC_ARCH NO_AFFINITY Haswell
SINGLE_THREADED
        Core in use  :  Haswell
   Copyright (c) 2011-2014, The OpenBLAS Project


  ***********************************
  * Starting time: Tue Dec 16 12:03:49 2025
  * Host name:     xe30th96.mpi-muelheim.mpg.de
  * Process ID:    4170383
  * Working dir.:  /usr/scratch/barasits/H-IRC__26969133
  ***********************************

NOTE: MaxCore=8000 MB was set to SCF,MP2,MDCI,CIPSI,MRCI,RASCI and CIS
      => If you want to overwrite this, your respective input block should be placed after the
MaxCore statement
Your calculation utilizes the atom-pairwise dispersion correction
with the Becke-Johnson damping scheme (D3BJ)


Your calculation utilizes the SMD solvation module
Please cite in your paper:
Marenich, A. V.; Cramer, C. J.; Truhlar, D. G. J. Phys. Chem. B 2009, 113, 6378

================================================================================

----- Orbital basis set information -----
Your calculation utilizes the basis: def2-TZVP
   F. Weigend and R. Ahlrichs, Phys. Chem. Chem. Phys. 7, 3297 (2005).

----- AuxJ basis set information -----
Your calculation utilizes the auxiliary basis: def2/J
   H-Rn: F. Weigend, Phys. Chem. Chem. Phys. 8, 1057 (2006).
   Fr-Lr: K. Eichkorn, F. Weigend, O. Treutler, R. Ahlrichs; Theor. Chem. Acc. 97, 119 (1997).

================================================================================
                                        WARNINGS
                       Please study these warnings very carefully!
================================================================================

WARNING: SinglePoint found together with IRC
         Switching on EnGrad - energy plus gradient calculation

================================================================================
                                       INPUT FILE
================================================================================
NAME = H-IRC.inp
|  1> #
|  2> #IRC LQA
|  3> #
|  4> ! irc B3LYP D3BJ def2-TZVP def2/J verytightscf
|  5> %pal nprocs 12 end
|  6> %maxcore 8000
|  7> %irc
|  8> printlevel 1
```

```
|  9> method lqa
| 10> maxiter 241
| 11> tolrmsg 1.e-5 # Tighter convergence criteria
| 12> tolmaxg 8.e-5 # Tighter convergence criteria
| 13> follow_coordtype massweighted
| 14> inithess read
| 15> hess_filename "TS-H_Compound_3.hess"
| 16> lqahess lqacalc_bofillupdate
| 17> init_displ length
| 18> scale_init_displ 0.04
| 19> adapt_scale_displ false
| 20> scale_displ_sd 0.11
| 21> ENFORCESTRICTCONVERGENCE true
| 22> REPORTPROFILE true
| 23> BSCORRECTOR true
| 24> BSRICHTHRESH 1E-6
| 25> INITBSCORRECTOR false
| 26> end
| 27> %CPCM SMD true
| 28>      SMDSOLVENT "TETRAHYDROFURAN" END
| 29>
| 30> * xyz 0 1
| 31>   C         -1.83159688925588      0.41614799120395     -6.10707295233347
| 32>   C         -1.39747712509140     -0.29047635892215     -4.98494689017283
| 33>   C         -0.96325138382784      1.34729917850720     -6.68078470140177
| 34>   C         -0.13552923006917     -0.06909006381721     -4.44602358502525
| 35>   C          0.30297538742128      1.56806246013998     -6.14960105278649
| 36>   C          0.72071730162502      0.85963960584997     -5.02773443666966
| 37>   H         -2.05438046481555     -1.02365089690211     -4.53293579250229
| 38>   H         -1.28688888952695      1.90734271971577     -7.55066942112676
| 39>   H          0.18301660244585     -0.62820980195008     -3.57567170653008
| 40>   H          0.96166546256545      2.29435791140823     -6.61021650881703
| 41>   H          1.70655546373106      1.02686690431794     -4.61228006549880
| 42>   C         -3.19493688296370      0.17150095073100     -6.69586570392663
| 43>   H         -3.55404878626366      1.10039374120779     -7.14940657726161
| 44>   H         -3.89488633096183     -0.07568507928596     -5.89453816843713
| 45>   C         -3.25573787513559     -0.94025121976507     -7.75435894381739
| 46>   H         -3.15939399430542     -2.07197809918528     -7.09154005140956
| 47>   H         -4.25376932229165     -1.05836638027797     -8.17790782676467
| 48>   C         -2.20870150325039     -1.13817911865303     -8.67337445950710
| 49>   H         -2.41907385074580     -1.47845998992063     -9.67790776037587
| 50>   H         -1.24533046461525     -0.66334520842415     -8.53976582854039
| 51>   Se        -1.48139051502624     -3.44264738093027     -7.91551337488970
| 52>   O         -2.74944688962905     -3.27496434139872     -6.76717575410648
| 53>   C          0.09668362690643     -2.98704100098193     -6.88971408574240
| 54>   C          0.24860955196306     -3.47923370213723     -5.59687844905402
| 55>   C          1.10967774880390     -2.24667430407651     -7.49074480379587
| 56>   C          1.42454563653190     -3.22365553058524     -4.90208704849533
| 57>   C          2.28643233854032     -2.00034524662200     -6.79056343566211
| 58>   C          2.44550919917246     -2.48912523615284     -5.49846278224761
| 59>   H         -0.54736019079749     -4.05012368156640     -5.13700910033285
| 60>   H          0.98160189229898     -1.85647081224499     -8.49269460446677
| 61>   H          1.54353665075097     -3.59921111971675     -3.89334099131984
| 62>   H          3.07237585203485     -1.41563788545038     -7.25197912926308
| 63>   H          3.35962217378140     -2.29059980411481     -4.95333185771847
| 64> *
| 65>
| 66>
| 67>                          ****END OF INPUT****
================================================================================

                     *******************************
                     * Energy+Gradient Calculation *
```

****

CARTESIAN COORDINATES (ANGSTROEM)

C	-1.831597	0.416148	-6.107073
C	-1.397477	-0.290476	-4.984947
C	-0.963251	1.347299	-6.680785
C	-0.135529	-0.069090	-4.446024
C	0.302975	1.568062	-6.149601
C	0.720717	0.859640	-5.027734
H	-2.054380	-1.023651	-4.532936
H	-1.286889	1.907343	-7.550669
H	0.183017	-0.628210	-3.575672
H	0.961665	2.294358	-6.610217
H	1.706555	1.026867	-4.612280
C	-3.194937	0.171501	-6.695866
H	-3.554049	1.100394	-7.149407
H	-3.894886	-0.075685	-5.894538
C	-3.255738	-0.940251	-7.754359
H	-3.159394	-2.071978	-7.091540
H	-4.253769	-1.058366	-8.177908
C	-2.208702	-1.138179	-8.673374
H	-2.419074	-1.478460	-9.677908
H	-1.245330	-0.663345	-8.539766
Se	-1.481391	-3.442647	-7.915513
O	-2.749447	-3.274964	-6.767176
C	0.096684	-2.987041	-6.889714
C	0.248610	-3.479234	-5.596878
C	1.109678	-2.246674	-7.490745
C	1.424546	-3.223656	-4.902087
C	2.286432	-2.000345	-6.790563
C	2.445509	-2.489125	-5.498463
H	-0.547360	-4.050124	-5.137009
H	0.981602	-1.856471	-8.492695
H	1.543537	-3.599211	-3.893341
H	3.072376	-1.415638	-7.251979
H	3.359622	-2.290600	-4.953332

CARTESIAN COORDINATES (A.U.)

NO	LB	ZA	FRAG	MASS	X	Y	Z
0	C	6.0000	0	12.011	-3.461217	0.786406	-11.540695
1	C	6.0000	0	12.011	-2.640849	-0.548921	-9.420184
2	C	6.0000	0	12.011	-1.820281	2.546026	-12.624853
3	C	6.0000	0	12.011	-0.256113	-0.130561	-8.401767
4	C	6.0000	0	12.011	0.572541	2.963209	-11.621062
5	C	6.0000	0	12.011	1.361958	1.624483	-9.501041
6	H	1.0000	0	1.008	-3.882216	-1.934420	-8.566007
7	H	1.0000	0	1.008	-2.431868	3.604355	-14.268697
8	H	1.0000	0	1.008	0.345851	-1.187144	-6.757040
9	H	1.0000	0	1.008	1.817284	4.335708	-12.491499
10	H	1.0000	0	1.008	3.224922	1.940497	-8.715946
11	C	6.0000	0	12.011	-6.037556	0.324090	-12.653352
12	H	1.0000	0	1.008	-6.716179	2.079443	-13.510420
13	H	1.0000	0	1.008	-7.360268	-0.143024	-11.139063
14	C	6.0000	0	12.011	-6.152453	-1.776817	-14.653615
15	H	1.0000	0	1.008	-5.970389	-3.915471	-13.401069
16	H	1.0000	0	1.008	-8.038459	-2.000023	-15.454006
17	C	6.0000	0	12.011	-4.173841	-2.150847	-16.390302
18	H	1.0000	0	1.008	-4.571387	-2.793884	-18.288595
19	H	1.0000	0	1.008	-2.353334	-1.253541	-16.137819

20	Se	34.0000	0	78.960	-2.799422	-6.505661	-14.958152
21	O	8.0000	0	15.999	-5.195702	-6.188786	-12.788109
22	C	6.0000	0	12.011	0.182706	-5.644689	-13.019673
23	C	6.0000	0	12.011	0.469804	-6.574799	-10.576567
24	C	6.0000	0	12.011	2.096987	-4.245599	-14.155456
25	C	6.0000	0	12.011	2.692001	-6.091826	-9.263602
26	C	6.0000	0	12.011	4.320731	-3.780105	-12.832305
27	C	6.0000	0	12.011	4.621343	-4.703765	-10.390589
28	H	1.0000	0	1.008	-1.034361	-7.653625	-9.707540
29	H	1.0000	0	1.008	1.854959	-3.508221	-16.048867
30	H	1.0000	0	1.008	2.916862	-6.801523	-7.357348
31	H	1.0000	0	1.008	5.805949	-2.675168	-13.704254
32	H	1.0000	0	1.008	6.348766	-4.328606	-9.360441

INTERNAL COORDINATES (ANGSTROEM)

C	0	0	0	0.000000000000	0.00000000	0.00000000
C	1	0	0	1.395329653240	0.00000000	0.00000000
C	1	2	0	1.396499774949	118.33298051	0.00000000
C	2	1	3	1.389950614318	120.90541830	0.34753760
C	3	1	2	1.390763362798	121.00112527	359.97110574
C	4	2	1	1.390713643163	120.21027970	359.55426949
H	2	1	3	1.083227092211	119.31896439	180.59655329
H	3	1	2	1.084015419058	119.24266697	180.23849284
H	4	2	1	1.082404139581	119.78105146	180.05938893
H	5	3	1	1.083302495133	119.84249026	179.94910211
H	6	4	2	1.082794618449	120.21227951	180.45071536
C	1	2	3	1.505066449425	120.93846652	179.30681273
H	12	1	2	1.094304573269	108.74894101	149.52902008
H	12	1	2	1.092316795770	109.28367216	33.51632083
C	12	1	2	1.536260911482	115.02584542	271.29437162
H	15	12	1	1.315072909427	105.81963760	74.09075048
H	15	12	1	1.090601493168	112.46866514	182.02456051
C	15	12	1	1.407142542633	121.48920115	322.94178082
H	18	15	12	1.081265346016	120.41474497	213.79671374
H	18	15	12	1.082313386526	121.33089690	13.11915938
Se	16	15	12	2.318051335540	112.50343577	232.60317184
O	16	15	12	1.311657243701	159.52404371	227.91538672
C	21	16	15	1.936532817306	105.21764934	91.52668494
C	23	21	16	1.391674935201	119.85727847	78.08764013
C	23	21	16	1.391236091010	119.32139122	253.60765866
C	24	23	21	1.389561429752	119.46103316	175.59841687
C	25	23	21	1.391288365974	119.51525624	184.66756926
C	27	25	23	1.390588157690	120.14180838	359.86127348
H	24	23	21	1.082110446256	120.07128057	355.17805924
H	25	23	21	1.082850679051	120.35661350	5.22032727
H	26	24	23	1.082944813857	119.66646487	179.57698725
H	27	25	23	1.082817781030	119.67391068	180.63693219
H	28	27	25	1.082673820868	119.99851869	180.39672220

INTERNAL COORDINATES (A.U.)

C	0	0	0	0.000000000000	0.00000000	0.00000000
C	1	0	0	2.636790911162	0.00000000	0.00000000
C	1	2	0	2.639002120736	118.33298051	0.00000000
C	2	1	3	2.626626000736	120.90541830	0.34753760
C	3	1	2	2.628161872780	121.00112527	359.97110574
C	4	2	1	2.628067916286	120.21027970	359.55426949
H	2	1	3	2.047002545123	119.31896439	180.59655329
H	3	1	2	2.048492266968	119.24266697	180.23849284
H	4	2	1	2.045447390031	119.78105146	180.05938893

```
 H      5   3   1     2.047145035994   119.84249026   179.94910211
 H      6   4   2     2.046185288153   120.21227951   180.45071536
 C      1   2   3     2.844163402766   120.93846652   179.30681273
 H     12   1   2     2.067935950576   108.74894101   149.52902008
 H     12   1   2     2.064179595488   109.28367216    33.51632083
 C     12   1   2     2.903112392948   115.02584542   271.29437162
 H     15  12   1     2.485127644956   105.81963760    74.09075048
 H     15  12   1     2.060938143334   112.46866514   182.02456051
 C     15  12   1     2.659114036966   121.48920115   322.94178082
 H     18  15  12     2.043295382070   120.41474497   213.79671374
 H     18  15  12     2.045275891611   121.33089690    13.11915938
 Se    16  15  12     4.380482188541   112.50343577   232.60317184
 O     16  15  12     2.478672972168   159.52404371   227.91538672
 C     21  16  15     3.659516674058   105.21764934    91.52668494
 C     23  21  16     2.629884494972   119.85727847    78.08764013
 C     23  21  16     2.629055199636   119.32139122   253.60765866
 C     24  23  21     2.625890548492   119.46103316   175.59841687
 C     25  23  21     2.629153985001   119.51525624   184.66756926
 C     27  25  23     2.627830783109   120.14180838   359.86127348
 H     24  23  21     2.044892390079   120.07128057   355.17805924
 H     25  23  21     2.046291227337   120.35661350     5.22032727
 H     26  24  23     2.046469116340   119.66646487   179.57698725
 H     27  25  23     2.046229059086   119.67391068   180.63693219
 H     28  27  25     2.045957013806   119.99851869   180.39672220

---------------------
BASIS SET INFORMATION
---------------------
There are 4 groups of distinct atoms

 Group   1 Type C   : 11s6p2d1f contracted to 5s3p2d1f pattern {62111/411/11/1}
 Group   2 Type H   : 5s1p contracted to 3s1p pattern {311/1}
 Group   3 Type Se  : 17s13p8d1f contracted to 6s5p4d1f pattern {842111/64111/5111/1}
 Group   4 Type O   : 11s6p2d1f contracted to 5s3p2d1f pattern {62111/411/11/1}

Atom   0C    basis set group =>   1
Atom   1C    basis set group =>   1
Atom   2C    basis set group =>   1
Atom   3C    basis set group =>   1
Atom   4C    basis set group =>   1
Atom   5C    basis set group =>   1
Atom   6H    basis set group =>   2
Atom   7H    basis set group =>   2
Atom   8H    basis set group =>   2
Atom   9H    basis set group =>   2
Atom  10H    basis set group =>   2
Atom  11C    basis set group =>   1
Atom  12H    basis set group =>   2
Atom  13H    basis set group =>   2
Atom  14C    basis set group =>   1
Atom  15H    basis set group =>   2
Atom  16H    basis set group =>   2
Atom  17C    basis set group =>   1
Atom  18H    basis set group =>   2
Atom  19H    basis set group =>   2
Atom  20Se   basis set group =>   3
Atom  21O    basis set group =>   4
Atom  22C    basis set group =>   1
Atom  23C    basis set group =>   1
Atom  24C    basis set group =>   1
Atom  25C    basis set group =>   1
Atom  26C    basis set group =>   1
Atom  27C    basis set group =>   1
```

```
Atom  28H    basis set group =>   2
Atom  29H    basis set group =>   2
Atom  30H    basis set group =>   2
Atom  31H    basis set group =>   2
Atom  32H    basis set group =>   2
---------------------------------
AUXILIARY/J BASIS SET INFORMATION
---------------------------------
There are 4 groups of distinct atoms

 Group   1 Type C   : 12s5p4d2f1g contracted to 6s4p3d1f1g pattern {711111/2111/211/2/1}
 Group   2 Type H   : 5s2p1d contracted to 3s1p1d pattern {311/2/1}
 Group   3 Type Se  : 19s5p5d3f1g contracted to 8s4p3d2f1g pattern {121111111/2111/311/21/1}
 Group   4 Type O   : 12s5p4d2f1g contracted to 6s4p3d1f1g pattern {711111/2111/211/2/1}

Atom   0C    basis set group =>   1
Atom   1C    basis set group =>   1
Atom   2C    basis set group =>   1
Atom   3C    basis set group =>   1
Atom   4C    basis set group =>   1
Atom   5C    basis set group =>   1
Atom   6H    basis set group =>   2
Atom   7H    basis set group =>   2
Atom   8H    basis set group =>   2
Atom   9H    basis set group =>   2
Atom  10H    basis set group =>   2
Atom  11C    basis set group =>   1
Atom  12H    basis set group =>   2
Atom  13H    basis set group =>   2
Atom  14C    basis set group =>   1
Atom  15H    basis set group =>   2
Atom  16H    basis set group =>   2
Atom  17C    basis set group =>   1
Atom  18H    basis set group =>   2
Atom  19H    basis set group =>   2
Atom  20Se   basis set group =>   3
Atom  21O    basis set group =>   4
Atom  22C    basis set group =>   1
Atom  23C    basis set group =>   1
Atom  24C    basis set group =>   1
Atom  25C    basis set group =>   1
Atom  26C    basis set group =>   1
Atom  27C    basis set group =>   1
Atom  28H    basis set group =>   2
Atom  29H    basis set group =>   2
Atom  30H    basis set group =>   2
Atom  31H    basis set group =>   2
Atom  32H    basis set group =>   2


           ************************************************************
           *        Program running with 12 parallel MPI-processes    *
           *              working on a common directory               *
           ************************************************************
------------------------------------------------------------------------------
                            ORCA STARTUP CALCULATIONS
                          -- RI-GTO INTEGRALS CHOSEN --
------------------------------------------------------------------------------
------------------------------------------------------------------------------
                                   ___
                                  /   \      - P O W E R E D   B Y -
                                 /     \
                                 |  |  |   _    _      __       _____    __    __
```

```
                  |  |  |  | |  | |    /  \    |  _  \  |  |  /  |
                   \  \/   | |  | |   /    \   | |_| |  |  | /  /
                  / \  \   | |__| |  /  /\  \  | |_| |  |  |/  /
                 |  |  |   |  __  | /  /__\  \ |    /   |      \
                 |  |  |   | |  | | |   __   | |    \   |  |\   \
                 \     /   | |  | | |  |  |  | |  |\ \  |  | \   \
                  \___/    |_|  |_| |__|  |__| |_|  \__\ |__|  \__/

                       - O R C A ' S   B I G   F R I E N D -
                                       &
                        - I N T E G R A L   F E E D E R -

 v1 FN, 2020, v2 2021, v3 2022-2024
------------------------------------------------------------------------------


----------------------
SHARK INTEGRAL PACKAGE
----------------------

Number of atoms                             ...     33
Number of basis functions                   ...    640
Number of shells                            ...    256
Maximum angular momentum                    ...      3
Integral batch strategy                     ... SHARK/LIBINT Hybrid
RI-J (if used) integral strategy            ... SPLIT-RIJ (Revised 2003 algorithm where
possible)
Printlevel                                  ...      1
Contraction scheme used                     ... SEGMENTED contraction
Prescreening option                         ... SCHWARTZ
   Thresh                                   ... 1.000e-12
   Tcut                                     ... 1.000e-14
   Tpresel                                  ... 1.000e-14
Coulomb Range Separation                    ... NOT USED
Exchange Range Separation                   ... NOT USED
Multipole approximations                    ... NOT USED
Finite Nucleus Model                        ... NOT USED
CABS basis                                  ... NOT available
Auxiliary Coulomb fitting basis             ... AVAILABLE
   # of basis functions in Aux-J            ...   1018
   # of shells in Aux-J                     ...    338
   Maximum angular momentum in Aux-J        ...      4
Auxiliary J/K fitting basis                 ... NOT available
Auxiliary Correlation fitting basis         ... NOT available
Auxiliary 'external' fitting basis          ... NOT available

Checking pre-screening integrals            ... done (  0.0 sec) Dimension = 256
   => SHARK Basis and OBASIS are compatible. Storing Pre-screening
Shell pair information
Shell pair cut-off parameter TPreSel        ...   1.0e-14
Total number of shell pairs                 ...     32896
Shell pairs after pre-screening             ...     28160
Total number of primitive shell pairs       ...    104206
Primitive shell pairs kept                  ...     60738
          la=0 lb=0:   7367 shell pairs
          la=1 lb=0:   7996 shell pairs
          la=1 lb=1:   2203 shell pairs
          la=2 lb=0:   4117 shell pairs
          la=2 lb=1:   2257 shell pairs
          la=2 lb=2:    608 shell pairs
          la=3 lb=0:   1860 shell pairs
          la=3 lb=1:   1058 shell pairs
          la=3 lb=2:    554 shell pairs
```

```
          la=3 lb=3:    140 shell pairs

Checking whether 4 symmetric matrices of dimension 640 fit in memory:
Max Core in MB      =   8000.00
 MB in use           =     38.22
 MB left             =   7961.78
 MB needed           =      6.26
 Data fit in memory  = YES
Calculating RI/J V-Matrix + Cholesky decomp.... done (  0.1 sec)
Calculating Nuclear repulsion               ... done (  0.0 sec) ENN=   1622.207228878682 Eh

Diagonalization of the overlap matrix:
Smallest eigenvalue                        ... 6.625e-06
Time for diagonalization                   ...    0.071 sec
Threshold for overlap eigenvalues          ... 1.000e-07
Number of eigenvalues below threshold      ... 0
Time for construction of square roots      ...    0.042 sec
Total time needed                          ...    0.117 sec

-------------------
DFT GRID GENERATION
-------------------

General Integration Accuracy     IntAcc      ... 4.388
Radial Grid Type                 RadialGrid  ... OptM3 with GC (2021)
Angular Grid (max. ang.)         AngularGrid ... 4 (Lebedev-302)
Angular grid pruning method      GridPruning ... 4 (adaptive)
Weight generation scheme         WeightScheme... mBecke (2022)
Basis function cutoff            BFCut       ... 1.0000e-12
Integration weight cutoff        WCut        ... 1.0000e-14
Partially contracted basis set               ... off
Rotationally invariant grid construction     ... off
Angular grids for H and He will be reduced by one unit
Core-polarized basis detected: some atoms will have their core
                               angular grid increased.

Total number of grid points                  ...   157446
Total number of batches                      ...     2477
Average number of points per batch           ...       63
Average number of grid points per atom       ...     4771

--------------------
COSX GRID GENERATION
--------------------

GRIDX 1
-------
General Integration Accuracy     IntAcc      ... 3.816
Radial Grid Type                 RadialGrid  ... OptM3 with GC (2021)
Angular Grid (max. ang.)         AngularGrid ... 1 (Lebedev-50)
Angular grid pruning method      GridPruning ... 4 (adaptive)
Weight generation scheme         WeightScheme... mBecke (2022)
Basis function cutoff            BFCut       ... 1.0000e-12
Integration weight cutoff        WCut        ... 1.0000e-14
Partially contracted basis set               ... on
Rotationally invariant grid construction     ... off
Angular grids for H and He will be reduced by one unit
Core-polarized basis detected: some atoms will have their core
                               angular grid increased.

Total number of grid points                  ...    24519
Total number of batches                      ...      207
Average number of points per batch           ...      118
```

```
Average number of grid points per atom       ...      743
UseSFitting                                  ... on

GRIDX 2
-------
General Integration Accuracy     IntAcc      ... 4.020
Radial Grid Type                 RadialGrid  ... OptM3 with GC (2021)
Angular Grid (max. ang.)         AngularGrid ... 2 (Lebedev-110)
Angular grid pruning method      GridPruning ... 4 (adaptive)
Weight generation scheme         WeightScheme... mBecke (2022)
Basis function cutoff            BFCut       ... 1.0000e-12
Integration weight cutoff        WCut        ... 1.0000e-14
Partially contracted basis set               ... on
Rotationally invariant grid construction     ... off
Angular grids for H and He will be reduced by one unit
Core-polarized basis detected: some atoms will have their core
                               angular grid increased.

Total number of grid points                  ...    45718
Total number of batches                      ...      377
Average number of points per batch           ...      121
Average number of grid points per atom       ...     1385
UseSFitting                                  ... on

GRIDX 3
-------
General Integration Accuracy     IntAcc      ... 4.338
Radial Grid Type                 RadialGrid  ... OptM3 with GC (2021)
Angular Grid (max. ang.)         AngularGrid ... 3 (Lebedev-194)
Angular grid pruning method      GridPruning ... 4 (adaptive)
Weight generation scheme         WeightScheme... mBecke (2022)
Basis function cutoff            BFCut       ... 1.0000e-12
Integration weight cutoff        WCut        ... 1.0000e-14
Partially contracted basis set               ... on
Rotationally invariant grid construction     ... off
Angular grids for H and He will be reduced by one unit
Core-polarized basis detected: some atoms will have their core
                               angular grid increased.

Total number of grid points                  ...    92680
Total number of batches                      ...      740
Average number of points per batch           ...      125
Average number of grid points per atom       ...     2808
UseSFitting                                  ... on
Grids setup in   2.2 sec
Initializing property integral containers    ... done (  0.0 sec)

SHARK setup successfully completed in   2.7 seconds

Maximum memory used throughout the entire STARTUP-calculation: 76.2 MB


           ************************************************************
           *        Program running with 12 parallel MPI-processes    *
           *              working on a common directory               *
           ************************************************************
------------------------------------------------------------------------------
                                ORCA GUESS
                   Start orbitals & Density for SCF / CASSCF
------------------------------------------------------------------------------

------------
SCF SETTINGS
```

```
------------
Hamiltonian:
 Density Functional     Method          .... DFT(GTOs)
 Exchange Functional    Exchange        .... B88
   X-Alpha parameter    XAlpha          ....  0.666667
   Becke's b parameter  XBeta           ....  0.004200
 Correlation Functional Correlation     .... LYP
 LDA part of GGA corr.  LDAOpt          .... VWN-5
 Gradients option       PostSCFGGA      .... off
 Hybrid DFT is turned on
   Fraction HF Exchange ScalHFX         ....  0.200000
   Scaling of DF-GGA-X  ScalDFX         ....  0.720000
   Scaling of DF-GGA-C  ScalDFC         ....  0.810000
   Scaling of DF-LDA-C  ScalLDAC        ....  1.000000
   Perturbative correction              ....  0.000000
   NL short-range parameter             ....  4.800000
 RI-approximation to the Coulomb term is turned on
   Number of AuxJ basis functions       .... 1018
   RIJ-COSX (HFX calculated with COS-X)).... on


General Settings:
 Integral files         IntName         .... H-IRC
 Hartree-Fock type      HFTyp           .... RHF
 Total Charge           Charge          ....    0
 Multiplicity           Mult            ....    1
 Number of Electrons    NEL             ....  148
 Basis Dimension        Dim             ....  640
 Nuclear Repulsion      ENuc            ....   1622.2072288787 Eh

Convergence Acceleration:
 AO-DIIS                CNVDIIS         .... on
   Start iteration      DIISMaxIt       ....    12
   Startup error        DIISStart       ....  0.200000
   # of expansion vecs  DIISMaxEq       ....     5
   Bias factor          DIISBfac        ....   1.050
   Max. coefficient     DIISMaxC        ....  10.000
 MO-DIIS                CNVKDIIS        .... off
 Trust-Rad. Augm. Hess. CNVTRAH         .... auto
   Auto Start mean grad. ratio tolernc. ....  1.125000
   Auto Start start iteration           ....    50
   Auto Start num. interpolation iter.  ....    10
   Max. Number of Micro iterations      ....    24
   Max. Number of Macro iterations      .... Maxiter - #DIIS iter
   Number of Davidson start vectors     ....     2
   Converg. threshold    (grad. norm)   ....   2.000e-06
   Grad. Scal. Fac. for Micro threshold ....   0.100
   Minimum threshold for Micro iter.    ....   1.000e-02
   NR start threshold (gradient norm)   ....   1.000e-04
   Initial trust radius                 ....   0.400
   Minimum AH scaling param. (alpha)    ....   1.000
   Maximum AH scaling param. (alpha)    .... 1000.000
   Quad. conv. algorithm                .... NR
   White noise on init. David. guess    .... on
   Maximum white noise                  ....   0.010
   Pseudo random numbers                .... off
   Inactive MOs                         .... canonical
   Orbital update algorithm             .... Taylor
   Preconditioner                       .... Diag
   Full preconditioner red. dimension   ....   250
 SOSCF                 CNVSOSCF           .... on
   Start iteration     SOSCFMaxIt         ....   150
   Startup grad/error  SOSCFStart         ....  0.003300
```

```
   Hessian update      SOSCFHessUp        .... L-BFGS
   Autom. constraints  SOSCFAutoConstrain .... off
 Level Shifting         CNVShift        .... on
   Level shift para.    LevelShift      ....    0.2500
   Turn off err/grad.   ShiftErr        ....    0.0010
 Zerner damping         CNVZerner       .... off
 Static damping         CNVDamp         .... on
   Fraction old density DampFac         ....    0.7000
   Max. Damping (<1)    DampMax         ....    0.9800
   Min. Damping (>=0)   DampMin         ....    0.0000
   Turn off err/grad.   DampErr         ....    0.1000

SCF Procedure:
 Maximum # iterations   MaxIter         ....   125
 SCF integral mode      SCFMode         .... Direct
   Integral package                     .... SHARK and LIBINT hybrid scheme
 Reset frequency        DirectResetFreq ....    20
 Integral Threshold     Thresh          ....  1.000e-12 Eh
 Primitive CutOff       TCut            ....  1.000e-14 Eh

Convergence Tolerance:
 Convergence Check Mode ConvCheckMode   .... Total+1el-Energy
 Convergence forced     ConvForced      .... 0
 Energy Change          TolE            ....  1.000e-09 Eh
 1-El. energy change                    ....  1.000e-06 Eh
 Orbital Gradient       TolG            ....  2.000e-06
 Orbital Rotation angle TolX            ....  2.000e-06
 DIIS Error             TolErr          ....  1.000e-08

------------------------------
INITIAL GUESS: MODEL POTENTIAL
------------------------------
Loading Hartree-Fock densities                     ... done
Calculating cut-offs                               ... done
Initializing the effective Hamiltonian             ... done
Setting up the integral package (SHARK)            ... done
Starting the Coulomb interaction                   ... done (   0.3 sec)
Making the grid                                    ... done (   0.3 sec)
Mapping shells                                     ... done
Starting the XC term evaluation                    ... done (   0.2 sec)
  promolecular density results
     # of electrons  =    147.989736454
     EX              =   -175.673266506
     EC              =     -5.460450695
     EX+EC           =   -181.133717201
Transforming the Hamiltonian                       ... done (   0.0 sec)
Diagonalizing the Hamiltonian                      ... done (   0.1 sec)
Back transforming the eigenvectors                 ... done (   0.0 sec)
Now organizing SCF variables                       ... done
                      ------------------
                      INITIAL GUESS DONE (   1.0 sec)
                      ------------------
             **** ENERGY FILE WAS UPDATED (H-IRC.en.tmp) ****
Finished Guess after   1.3 sec
Maximum memory used throughout the entire GUESS-calculation: 43.7 MB


           ************************************************************
           *        Program running with 12 parallel MPI-processes    *
           *              working on a common directory               *
           ************************************************************

--------------------------------------------------------------------------------
```

```
                                 ORCA LEAN-SCF
                         memory conserving SCF solver
--------------------------------------------------------------------------------------------

--------------------
CPCM SOLVATION MODEL
--------------------
CPCM parameters:
  Epsilon                                          ...       7.4257
  Refrac                                           ...       1.4050
  Rsolv                                            ...       1.3000
  Surface type                                     ... GAUSSIAN VDW
  Discretization scheme                            ... Constant charge density
    Threshold for H atoms                          ...       5.0000 (charges/Ang^2)
    Threshold for non-H atoms                      ...       5.0000 (charges/Ang^2)
  Epsilon function type                            ...         CPCM
Solvent:                                           ... TETRAHYDROFURAN
SMD-CDS solvent descriptors:
  Soln                                             ...       1.4050
  Soln25                                           ...       1.4044
  Sola                                             ...       0.0000
  Solb                                             ...       0.4800
  Solg                                             ...      39.4400
  Solc                                             ...       0.0000
  Solh                                             ...       0.0000
Radii:
  Scheme                                           ... Element-dependent radii
  Radius for C  used is    3.4960 Bohr (=   1.8500 Ang.)
  Radius for H  used is    2.2677 Bohr (=   1.2000 Ang.)
  Radius for Se used is    3.5905 Bohr (=   1.9000 Ang.)
  Radius for O  used is    4.3350 Bohr (=   2.2940 Ang.)
Calculating surface                                ...        done! (  0.0s)
Cavity surface points                              ...         2570
Cavity Volume                                      ...    1858.7226
Cavity Surface-area                                ...    1025.1838
Calculating surface distance matrix                ...        done! (  0.1s)
Performing Cholesky decomposition & store          ...        done! (  0.2s)
Overall time for CPCM initialization               ...                 0.3s

----------------------------------------D-I-I-S--------------------------------------------
Iteration    Energy (Eh)           Delta-E    RMSDP     MaxDP     DIISErr   Damp  Time(sec)
--------------------------------------------------------------------------------------------
               ***  Starting incremental Fock matrix formation  ***
    1    -3057.0526584800877572     0.00e+00  6.11e-03  1.69e-01  3.13e-01  0.700   8.5
    2    -3057.3429118459548590    -2.90e-01  4.48e-03  1.36e-01  1.06e-01  0.700   5.4
                               ***Turning on AO-DIIS***
    3    -3057.4502736199397077    -1.07e-01  1.49e-03  3.27e-02  4.14e-02  0.700   5.2
    4    -3057.5155313288546495    -6.53e-02  2.81e-03  9.18e-02  1.93e-02  0.000   5.0
    5    -3057.6604055105103726    -1.45e-01  8.97e-04  2.30e-02  7.81e-03  0.000   5.2
                              *** Initializing SOSCF ***
---------------------------------------S-O-S-C-F--------------------------------------
Iteration    Energy (Eh)           Delta-E    RMSDP     MaxDP     MaxGrad    Time(sec)
--------------------------------------------------------------------------------------
    6    -3057.6616751767401183    -1.27e-03  3.92e-04  9.74e-03  2.95e-03     5.1
               *** Restarting incremental Fock matrix formation ***
    7    -3057.6617613845696724    -8.62e-05  4.25e-04  1.34e-02  4.79e-04     8.3
    8    -3057.6617494289907881     1.20e-05  1.47e-04  3.13e-03  6.59e-04     6.7
    9    -3057.6617784644308813    -2.90e-05  6.79e-05  6.34e-04  2.65e-04     6.3
   10    -3057.6617758649667849     2.60e-06  2.71e-05  3.57e-04  2.84e-04     6.4
   11    -3057.6617810435832325    -5.18e-06  1.21e-05  2.41e-04  3.08e-05     6.1
   12    -3057.6617809502163254     9.34e-08  6.35e-06  1.21e-04  4.01e-05     5.7
   13    -3057.6617811400105893    -1.90e-07  1.42e-06  2.27e-05  9.33e-06     5.5
   14    -3057.6617811371638709     2.85e-09  7.51e-07  1.00e-05  9.13e-06     4.8
```

```
  15    -3057.6617811421097031    -4.95e-09  4.38e-07  1.27e-05  2.00e-06     4.5
  16    -3057.6617811414448624     6.65e-10  1.69e-07  3.96e-06  2.14e-06     3.9
               **** Energy Check signals convergence ****

              *****************************************************
              *                     SUCCESS                       *
              *           SCF CONVERGED AFTER  16 CYCLES          *
              *****************************************************

Recomputing exchange energy using gridx3       ... done (   11.941 sec)
Old exchange energy                            :    -35.421270625 Eh
New exchange energy                            :    -35.421252996 Eh
Exchange energy change after final integration :      0.000017629 Eh
Total energy after final integration           :  -3057.661763513 Eh

SMD CDS free energy correction energy :                 -4.03537     Kcal/mol
Total Energy after SMD CDS correction =             -3057.668194278 Eh
             **** ENERGY FILE WAS UPDATED (H-IRC.en.tmp) ****

----------------
TOTAL SCF ENERGY
----------------

Total Energy       :      -3057.66819427825794 Eh          -83203.38154 eV

Components:
Nuclear Repulsion  :       1622.20722887868169 Eh           44142.50286 eV
Electronic Energy  :      -4679.85357474223292 Eh         -127345.28988 eV
One Electron Energy:      -7459.71704626140308 Eh         -202989.22060 eV
Two Electron Energy:       2779.86347151917016 Eh           75643.93072 eV
CPCM Dielectric    :         -0.01543527827990 Eh              -0.42002 eV
SMD CDS (Gcds)     :         -0.00643076544425 Eh              -0.17499 eV

Virial components:
Potential Energy   :      -6108.74384405083038 Eh         -166227.37083 eV
Kinetic Energy     :       3051.07564977257243 Eh           83023.98929 eV
Virial Ratio       :          2.00216072797349

DFT components:
N(Alpha)           :       74.000102476666 electrons
N(Beta)            :       74.000102476666 electrons
N(Total)           :      148.000204953333 electrons
E(X)               :     -140.894137149814 Eh
E(C)               :       -6.423656640742 Eh
E(XC)              :     -147.317793790556 Eh

CPCM Solvation Model Properties:
Surface-charge          :         -0.06010374328846
Corrected charge        :         -0.00000000000000
Outlying charge corr.   :          0.00019245753872 Eh               0.00524 eV
Free-energy (cav+disp)  :         -0.00643076544425 Eh              -0.17499 eV

---------------
SCF CONVERGENCE
---------------

  Last Energy change         ...   -6.6484e-10  Tolerance :   1.0000e-09
  Last MAX-Density change    ...    3.9633e-06  Tolerance :   1.0000e-08
  Last RMS-Density change    ...    1.6900e-07  Tolerance :   1.0000e-09
  Last DIIS Error            ...    2.9510e-03  Tolerance :   1.0000e-08
  Last Orbital Gradient      ...    2.1388e-06  Tolerance :   2.0000e-06
  Last Orbital Rotation      ...    1.3314e-06  Tolerance :   2.0000e-06
```

ORBITAL ENERGIES

NO	OCC	E(Eh)	E(eV)
0	2.0000	-453.927142	-12351.9855
1	2.0000	-58.107529	-1581.1863
2	2.0000	-52.159970	-1419.3449
3	2.0000	-52.159197	-1419.3239
4	2.0000	-52.158158	-1419.2956
5	2.0000	-19.109600	-519.9986
6	2.0000	-10.196827	-277.4698
7	2.0000	-10.184910	-277.1455
8	2.0000	-10.178398	-276.9683
9	2.0000	-10.176349	-276.9125
10	2.0000	-10.174774	-276.8697
11	2.0000	-10.174053	-276.8500
12	2.0000	-10.173172	-276.8261
13	2.0000	-10.172854	-276.8174
14	2.0000	-10.166022	-276.6315
15	2.0000	-10.165051	-276.6051
16	2.0000	-10.164710	-276.5958
17	2.0000	-10.164157	-276.5808
18	2.0000	-10.163694	-276.5682
19	2.0000	-10.162602	-276.5384
20	2.0000	-10.153745	-276.2975
21	2.0000	-7.845872	-213.4970
22	2.0000	-5.789832	-157.5494
23	2.0000	-5.785951	-157.4437
24	2.0000	-5.782215	-157.3421
25	2.0000	-2.143337	-58.3232
26	2.0000	-2.142571	-58.3023
27	2.0000	-2.138866	-58.2015
28	2.0000	-2.135511	-58.1102
29	2.0000	-2.135155	-58.1005
30	2.0000	-0.961540	-26.1648
31	2.0000	-0.861605	-23.4455
32	2.0000	-0.854329	-23.2475
33	2.0000	-0.793165	-21.5831
34	2.0000	-0.759620	-20.6703
35	2.0000	-0.750404	-20.4195
36	2.0000	-0.742859	-20.2142
37	2.0000	-0.739339	-20.1184
38	2.0000	-0.678384	-18.4598
39	2.0000	-0.659190	-17.9375
40	2.0000	-0.608151	-16.5486
41	2.0000	-0.599904	-16.3242
42	2.0000	-0.592496	-16.1226
43	2.0000	-0.581108	-15.8128
44	2.0000	-0.552989	-15.0476
45	2.0000	-0.513746	-13.9797
46	2.0000	-0.502824	-13.6825
47	2.0000	-0.466812	-12.7026
48	2.0000	-0.464438	-12.6380
49	2.0000	-0.456733	-12.4283
50	2.0000	-0.450003	-12.2452
51	2.0000	-0.446045	-12.1375
52	2.0000	-0.437465	-11.9040
53	2.0000	-0.423822	-11.5328
54	2.0000	-0.417377	-11.3574
55	2.0000	-0.412813	-11.2332
56	2.0000	-0.408719	-11.1218

```
  57   2.0000      -0.388762       -10.5787
  58   2.0000      -0.387551       -10.5458
  59   2.0000      -0.374203       -10.1826
  60   2.0000      -0.365523        -9.9464
  61   2.0000      -0.356398        -9.6981
  62   2.0000      -0.347709        -9.4616
  63   2.0000      -0.347297        -9.4504
  64   2.0000      -0.345552        -9.4030
  65   2.0000      -0.332127        -9.0376
  66   2.0000      -0.324633        -8.8337
  67   2.0000      -0.277742        -7.5578
  68   2.0000      -0.266588        -7.2542
  69   2.0000      -0.262836        -7.1521
  70   2.0000      -0.250036        -6.8038
  71   2.0000      -0.243014        -6.6127
  72   2.0000      -0.241241        -6.5645
  73   2.0000      -0.227024        -6.1776
  74   0.0000      -0.039675        -1.0796
  75   0.0000      -0.026883        -0.7315
  76   0.0000      -0.017109        -0.4656
  77   0.0000      -0.005808        -0.1580
  78   0.0000      -0.002129        -0.0579
  79   0.0000       0.018230         0.4961
  80   0.0000       0.034116         0.9283
  81   0.0000       0.051938         1.4133
  82   0.0000       0.064768         1.7624
  83   0.0000       0.071925         1.9572
  84   0.0000       0.074928         2.0389
*Only the first 10 virtual orbitals were printed (use %output print[p_orben] 2 end to see all).

                    ********************************
                    * MULLIKEN POPULATION ANALYSIS *
                    ********************************

-----------------------
MULLIKEN ATOMIC CHARGES
-----------------------
   0 C :    0.181214
   1 C :   -0.237054
   2 C :   -0.242190
   3 C :   -0.088115
   4 C :   -0.126382
   5 C :   -0.172974
   6 H :    0.121547
   7 H :    0.134012
   8 H :    0.137593
   9 H :    0.136412
  10 H :    0.135516
  11 C :   -0.179735
  12 H :    0.110636
  13 H :    0.116353
  14 C :   -0.266736
  15 H :    0.257709
  16 H :    0.126892
  17 C :   -0.302944
  18 H :    0.162492
  19 H :    0.161067
  20 Se:    0.412092
  21 O :   -0.571497
  22 C :    0.069201
  23 C :   -0.186937
  24 C :   -0.190336
  25 C :   -0.155219
```

```
  26 C :   -0.144175
  27 C :   -0.122756
  28 H :    0.139291
  29 H :    0.155658
  30 H :    0.142354
  31 H :    0.142792
  32 H :    0.144219
Sum of atomic charges:    0.0000000

--------------------------------
MULLIKEN REDUCED ORBITAL CHARGES
--------------------------------
  0 C s       :     3.155358  s :     3.155358
      pz      :     0.875249  p :     2.529356
      px      :     0.784364
      py      :     0.869743
      dz2     :     0.025101  d :     0.124234
      dxz     :     0.029088
      dyz     :     0.020312
      dx2y2   :     0.024727
      dxy     :     0.025005
      f0      :     0.001226  f :     0.009837
      f+1     :     0.001510
      f-1     :     0.001122
      f+2     :     0.001841
      f-2     :     0.001499
      f+3     :     0.001190
      f-3     :     0.001450

  1 C s       :     3.277010  s :     3.277010
      pz      :     0.963597  p :     2.870543
      px      :     0.976088
      py      :     0.930858
      dz2     :     0.018907  d :     0.082194
      dxz     :     0.020123
      dyz     :     0.011595
      dx2y2   :     0.011403
      dxy     :     0.020167
      f0      :     0.001127  f :     0.007306
      f+1     :     0.001226
      f-1     :     0.000604
      f+2     :     0.001203
      f-2     :     0.001376
      f+3     :     0.000847
      f-3     :     0.000922

  2 C s       :     3.269563  s :     3.269563
      pz      :     0.973710  p :     2.882930
      px      :     0.942888
      py      :     0.966331
      dz2     :     0.013709  d :     0.082425
      dxz     :     0.017955
      dyz     :     0.011632
      dx2y2   :     0.019178
      dxy     :     0.019950
      f0      :     0.000295  f :     0.007273
      f+1     :     0.001405
      f-1     :     0.000961
      f+2     :     0.001046
      f-2     :     0.001212
      f+3     :     0.001050
      f-3     :     0.001303
```

```
3 C s       :     3.182320  s :     3.182320
    pz      :     0.946666  p :     2.816365
    px      :     0.913839
    py      :     0.955860
    dz2     :     0.013496  d :     0.082170
    dxz     :     0.017740
    dyz     :     0.010804
    dx2y2   :     0.020335
    dxy     :     0.019794
    f0      :     0.000288  f :     0.007260
    f+1     :     0.001416
    f-1     :     0.001007
    f+2     :     0.001080
    f-2     :     0.001197
    f+3     :     0.000999
    f-3     :     0.001273

4 C s       :     3.210756  s :     3.210756
    pz      :     0.938764  p :     2.825738
    px      :     0.929466
    py      :     0.957507
    dz2     :     0.018726  d :     0.082622
    dxz     :     0.021087
    dyz     :     0.011168
    dx2y2   :     0.011526
    dxy     :     0.020115
    f0      :     0.001159  f :     0.007267
    f+1     :     0.001173
    f-1     :     0.000600
    f+2     :     0.001159
    f-2     :     0.001389
    f+3     :     0.000840
    f-3     :     0.000947

5 C s       :     3.235693  s :     3.235693
    pz      :     0.939948  p :     2.849204
    px      :     0.964316
    py      :     0.944941
    dz2     :     0.019236  d :     0.080842
    dxz     :     0.016739
    dyz     :     0.015067
    dx2y2   :     0.019277
    dxy     :     0.010523
    f0      :     0.001118  f :     0.007236
    f+1     :     0.000831
    f-1     :     0.001023
    f+2     :     0.001399
    f-2     :     0.001148
    f+3     :     0.000919
    f-3     :     0.000798

6 H s       :     0.856155  s :     0.856155
    pz      :     0.005851  p :     0.022298
    px      :     0.007533
    py      :     0.008915

7 H s       :     0.843823  s :     0.843823
    pz      :     0.010560  p :     0.022164
    px      :     0.004625
    py      :     0.006979

8 H s       :     0.840066  s :     0.840066
    pz      :     0.010785  p :     0.022341
```

```
     px      :     0.004628
     py      :     0.006928

  9 H s      :     0.841415  s :     0.841415
     pz      :     0.005831  p :     0.022173
     px      :     0.007540
     py      :     0.008802

 10 H s      :     0.842237  s :     0.842237
     pz      :     0.005537  p :     0.022247
     px      :     0.012257
     py      :     0.004453

 11 C s      :     3.237651  s :     3.237651
     pz      :     0.971691  p :     2.849282
     px      :     0.911538
     py      :     0.966053
     dz2     :     0.018084  d :     0.087031
     dxz     :     0.022797
     dyz     :     0.011860
     dx2y2   :     0.017132
     dxy     :     0.017158
     f0      :     0.000453  f :     0.005771
     f+1     :     0.000821
     f-1     :     0.000730
     f+2     :     0.001539
     f-2     :     0.000966
     f+3     :     0.000649
     f-3     :     0.000613

 12 H s      :     0.867652  s :     0.867652
     pz      :     0.005670  p :     0.021712
     px      :     0.005454
     py      :     0.010588

 13 H s      :     0.861897  s :     0.861897
     pz      :     0.008964  p :     0.021750
     px      :     0.008129
     py      :     0.004657

 14 C s      :     3.200550  s :     3.200550
     pz      :     0.999369  p :     2.964789
     px      :     0.947406
     py      :     1.018014
     dz2     :     0.024515  d :     0.095004
     dxz     :     0.014547
     dyz     :     0.019616
     dx2y2   :     0.022123
     dxy     :     0.014202
     f0      :     0.000919  f :     0.006393
     f+1     :     0.000715
     f-1     :     0.000733
     f+2     :     0.001804
     f-2     :     0.001046
     f+3     :     0.000466
     f-3     :     0.000710

 15 H s      :     0.689697  s :     0.689697
     pz      :     0.010837  p :     0.052594
     px      :     0.007288
     py      :     0.034469

 16 H s      :     0.850475  s :     0.850475
```

```
      pz      :     0.005848  p :     0.022633
      px      :     0.011588
      py      :     0.005198

 17 C s       :     3.302866  s :     3.302866
      pz      :     1.003881  p :     2.924723
      px      :     0.999984
      py      :     0.920858
      dz2     :     0.022517  d :     0.070424
      dxz     :     0.007571
      dyz     :     0.010406
      dx2y2   :     0.014437
      dxy     :     0.015493
      f0      :     0.000634  f :     0.004931
      f+1     :     0.001172
      f-1     :     0.000597
      f+2     :     0.000457
      f-2     :     0.000896
      f+3     :     0.000371
      f-3     :     0.000804

 18 H s       :     0.815164  s :     0.815164
      pz      :     0.012415  p :     0.022344
      px      :     0.004237
      py      :     0.005692

 19 H s       :     0.816173  s :     0.816173
      pz      :     0.004135  p :     0.022760
      px      :     0.011921
      py      :     0.006704

 20 Ses       :     7.840986  s :     7.840986
      pz      :     5.125532  p :    15.476895
      px      :     4.789711
      py      :     5.561652
      dz2     :     2.048113  d :    10.229780
      dxz     :     2.048611
      dyz     :     2.036386
      dx2y2   :     2.059200
      dxy     :     2.037469
      f0      :     0.005785  f :     0.040248
      f+1     :     0.009444
      f-1     :     0.001680
      f+2     :     0.007439
      f-2     :     0.008001
      f+3     :     0.003780
      f-3     :     0.004120

 21 O s       :     3.866254  s :     3.866254
      pz      :     1.584077  p :     4.678397
      px      :     1.488312
      py      :     1.606008
      dz2     :     0.005315  d :     0.025877
      dxz     :     0.007126
      dyz     :     0.003452
      dx2y2   :     0.005710
      dxy     :     0.004275
      f0      :     0.000110  f :     0.000969
      f+1     :     0.000225
      f-1     :     0.000037
      f+2     :     0.000226
      f-2     :     0.000158
      f+3     :     0.000117
```

```
     f-3     :     0.000096

22 C s       :     3.162886  s :     3.162886
     pz      :     0.828566  p :     2.634822
     px      :     0.891709
     py      :     0.914547
     dz2     :     0.024068  d :     0.124804
     dxz     :     0.038170
     dyz     :     0.024722
     dx2y2   :     0.020895
     dxy     :     0.016950
     f0      :     0.001286  f :     0.008287
     f+1     :     0.001684
     f-1     :     0.001012
     f+2     :     0.001169
     f-2     :     0.001174
     f+3     :     0.001205
     f-3     :     0.000757

23 C s       :     3.270340  s :     3.270340
     pz      :     0.919574  p :     2.819998
     px      :     0.927196
     py      :     0.973228
     dz2     :     0.023553  d :     0.089144
     dxz     :     0.026477
     dyz     :     0.015384
     dx2y2   :     0.007884
     dxy     :     0.015846
     f0      :     0.001290  f :     0.007456
     f+1     :     0.001734
     f-1     :     0.000758
     f+2     :     0.000829
     f-2     :     0.001286
     f+3     :     0.000690
     f-3     :     0.000867

24 C s       :     3.249291  s :     3.249291
     pz      :     0.971010  p :     2.846702
     px      :     0.912465
     py      :     0.963227
     dz2     :     0.022257  d :     0.086952
     dxz     :     0.016735
     dyz     :     0.009295
     dx2y2   :     0.019228
     dxy     :     0.019438
     f0      :     0.000607  f :     0.007391
     f+1     :     0.001591
     f-1     :     0.000820
     f+2     :     0.000977
     f-2     :     0.001165
     f+3     :     0.001276
     f-3     :     0.000955

25 C s       :     3.246682  s :     3.246682
     pz      :     0.958618  p :     2.820699
     px      :     0.900896
     py      :     0.961185
     dz2     :     0.018665  d :     0.080579
     dxz     :     0.016834
     dyz     :     0.009618
     dx2y2   :     0.018150
     dxy     :     0.017312
     f0      :     0.000601  f :     0.007259
```

```
      f+1     :     0.001585
      f-1     :     0.000772
      f+2     :     0.000983
      f-2     :     0.001103
      f+3     :     0.001308
      f-3     :     0.000907

 26 C s       :     3.230677  s :     3.230677
      pz      :     0.925481  p :     2.825165
      px      :     0.949884
      py      :     0.949800
      dz2     :     0.020583  d :     0.081055
      dxz     :     0.022828
      dyz     :     0.015286
      dx2y2   :     0.008588
      dxy     :     0.013770
      f0      :     0.001279  f :     0.007279
      f+1     :     0.001691
      f-1     :     0.000702
      f+2     :     0.000796
      f-2     :     0.001294
      f+3     :     0.000685
      f-3     :     0.000831

 27 C s       :     3.213748  s :     3.213748
      pz      :     0.922779  p :     2.821407
      px      :     0.956722
      py      :     0.941906
      dz2     :     0.017879  d :     0.080390
      dxz     :     0.023767
      dyz     :     0.016364
      dx2y2   :     0.014092
      dxy     :     0.008288
      f0      :     0.001257  f :     0.007210
      f+1     :     0.001377
      f-1     :     0.000957
      f+2     :     0.001111
      f-2     :     0.000912
      f+3     :     0.001068
      f-3     :     0.000529

 28 H s       :     0.838234  s :     0.838234
      pz      :     0.005550  p :     0.022475
      px      :     0.009675
      py      :     0.007250

 29 H s       :     0.822020  s :     0.822020
      pz      :     0.012747  p :     0.022322
      px      :     0.003892
      py      :     0.005684

 30 H s       :     0.835385  s :     0.835385
      pz      :     0.012775  p :     0.022261
      px      :     0.003849
      py      :     0.005638

 31 H s       :     0.834946  s :     0.834946
      pz      :     0.005609  p :     0.022262
      px      :     0.009290
      py      :     0.007363

 32 H s       :     0.833579  s :     0.833579
      pz      :     0.006335  p :     0.022202
```

```
      px      :     0.011195
      py      :     0.004672


                     *******************************
                     * LOEWDIN POPULATION ANALYSIS *
                     *******************************

----------------------
LOEWDIN ATOMIC CHARGES
----------------------
   0 C :   -0.148137
   1 C :   -0.114716
   2 C :   -0.124356
   3 C :   -0.143135
   4 C :   -0.153004
   5 C :   -0.154666
   6 H :    0.150473
   7 H :    0.151136
   8 H :    0.148156
   9 H :    0.147640
  10 H :    0.146970
  11 C :   -0.211131
  12 H :    0.146976
  13 H :    0.146591
  14 C :   -0.313191
  15 H :    0.186421
  16 H :    0.143588
  17 C :   -0.229533
  18 H :    0.125273
  19 H :    0.128298
  20 Se:    0.645984
  21 O :   -0.408287
  22 C :   -0.324233
  23 C :   -0.139936
  24 C :   -0.143496
  25 C :   -0.139220
  26 C :   -0.138216
  27 C :   -0.139133
  28 H :    0.150949
  29 H :    0.153097
  30 H :    0.151139
  31 H :    0.151243
  32 H :    0.150453

-------------------------------
LOEWDIN REDUCED ORBITAL CHARGES
-------------------------------
  0 C s       :     2.710721  s :     2.710721
      pz      :     0.962211  p :     2.860288
      px      :     0.964955
      py      :     0.933121
      dz2     :     0.101029  d :     0.519314
      dxz     :     0.121142
      dyz     :     0.086305
      dx2y2   :     0.110701
      dxy     :     0.100138
      f0      :     0.006449  f :     0.057813
      f+1     :     0.010646
      f-1     :     0.006987
      f+2     :     0.009548
      f-2     :     0.007972
```

```
    f+3     :     0.006616
    f-3     :     0.009594

1 C s       :     2.759564  s :     2.759564
    pz      :     0.979382  p :     2.929014
    px      :     1.011506
    py      :     0.938126
    dz2     :     0.075242  d :     0.383326
    dxz     :     0.106038
    dyz     :     0.056560
    dx2y2   :     0.056688
    dxy     :     0.088798
    f0      :     0.005903  f :     0.042811
    f+1     :     0.007725
    f-1     :     0.004885
    f+2     :     0.007582
    f-2     :     0.005489
    f+3     :     0.004916
    f-3     :     0.006312

2 C s       :     2.760275  s :     2.760275
    pz      :     0.954749  p :     2.937136
    px      :     1.028986
    py      :     0.953400
    dz2     :     0.060309  d :     0.384313
    dxz     :     0.085280
    dyz     :     0.058641
    dx2y2   :     0.088120
    dxy     :     0.091964
    f0      :     0.002519  f :     0.042632
    f+1     :     0.008836
    f-1     :     0.004421
    f+2     :     0.006863
    f-2     :     0.005807
    f+3     :     0.005221
    f-3     :     0.008965

3 C s       :     2.765784  s :     2.765784
    pz      :     0.955278  p :     2.945279
    px      :     1.037956
    py      :     0.952044
    dz2     :     0.061985  d :     0.388606
    dxz     :     0.085597
    dyz     :     0.058123
    dx2y2   :     0.089587
    dxy     :     0.093313
    f0      :     0.002510  f :     0.043465
    f+1     :     0.008994
    f-1     :     0.004658
    f+2     :     0.007068
    f-2     :     0.005896
    f+3     :     0.005257
    f-3     :     0.009083

4 C s       :     2.766408  s :     2.766408
    pz      :     0.994359  p :     2.954365
    px      :     1.016372
    py      :     0.943634
    dz2     :     0.078503  d :     0.388949
    dxz     :     0.107276
    dyz     :     0.057660
    dx2y2   :     0.055325
    dxy     :     0.090183
```

```
      f0      :     0.006155  f :     0.043282
      f+1     :     0.007655
      f-1     :     0.004999
      f+2     :     0.007626
      f-2     :     0.005563
      f+3     :     0.004944
      f-3     :     0.006340

  5 C s       :     2.766752  s :     2.766752
      pz      :     0.998092  p :     2.957776
      px      :     0.982429
      py      :     0.977255
      dz2     :     0.081738  d :     0.386710
      dxz     :     0.085498
      dyz     :     0.075801
      dx2y2   :     0.088935
      dxy     :     0.054739
      f0      :     0.005859  f :     0.043428
      f+1     :     0.006640
      f-1     :     0.006497
      f+2     :     0.007027
      f-2     :     0.006398
      f+3     :     0.005054
      f-3     :     0.005952

  6 H s       :     0.790155  s :     0.790155
      pz      :     0.015906  p :     0.059372
      px      :     0.019985
      py      :     0.023481

  7 H s       :     0.789698  s :     0.789698
      pz      :     0.027358  p :     0.059167
      px      :     0.013142
      py      :     0.018667

  8 H s       :     0.792618  s :     0.792618
      pz      :     0.027271  p :     0.059226
      px      :     0.013278
      py      :     0.018676

  9 H s       :     0.793303  s :     0.793303
      pz      :     0.016068  p :     0.059057
      px      :     0.020019
      py      :     0.022970

 10 H s       :     0.793729  s :     0.793729
      pz      :     0.015343  p :     0.059301
      px      :     0.031109
      py      :     0.012849

 11 C s       :     2.765935  s :     2.765935
      pz      :     1.010424  p :     3.016863
      px      :     0.996248
      py      :     1.010191
      dz2     :     0.063501  d :     0.390082
      dxz     :     0.100502
      dyz     :     0.070499
      dx2y2   :     0.086031
      dxy     :     0.069548
      f0      :     0.005275  f :     0.038252
      f+1     :     0.006571
      f-1     :     0.004600
      f+2     :     0.005335
```

```
     f-2     :     0.005451
     f+3     :     0.005686
     f-3     :     0.005333

 12 H s       :     0.794690  s :     0.794690
      pz      :     0.016605  p :     0.058333
      px      :     0.014030
      py      :     0.027698

 13 H s       :     0.795283  s :     0.795283
      pz      :     0.023831  p :     0.058125
      px      :     0.020301
      py      :     0.013993

 14 C s       :     2.787654  s :     2.787654
      pz      :     1.038544  p :     3.070496
      px      :     1.036646
      py      :     0.995306
      dz2     :     0.083909  d :     0.413185
      dxz     :     0.092348
      dyz     :     0.088127
      dx2y2   :     0.099118
      dxy     :     0.049684
      f0      :     0.007627  f :     0.041855
      f+1     :     0.006094
      f-1     :     0.004976
      f+2     :     0.007275
      f-2     :     0.006422
      f+3     :     0.004915
      f-3     :     0.004545

 15 H s       :     0.652944  s :     0.652944
      pz      :     0.029314  p :     0.160635
      px      :     0.018285
      py      :     0.113036

 16 H s       :     0.794471  s :     0.794471
      pz      :     0.017559  p :     0.061941
      px      :     0.029256
      py      :     0.015126

 17 C s       :     2.871816  s :     2.871816
      pz      :     1.039611  p :     3.026441
      px      :     1.075787
      py      :     0.911042
      dz2     :     0.098631  d :     0.302753
      dxz     :     0.049874
      dyz     :     0.030983
      dx2y2   :     0.055097
      dxy     :     0.068168
      f0      :     0.002983  f :     0.028524
      f+1     :     0.006582
      f-1     :     0.002752
      f+2     :     0.004141
      f-2     :     0.005685
      f+3     :     0.002420
      f-3     :     0.003961

 18 H s       :     0.809811  s :     0.809811
      pz      :     0.034472  p :     0.064916
      px      :     0.014072
      py      :     0.016373
```

```
19 H s       :     0.805239  s :     0.805239
     pz      :     0.013150  p :     0.066463
     px      :     0.034086
     py      :     0.019227

20 Ses       :     7.327032  s :     7.327032
     pz      :     5.137809  p :    15.377295
     px      :     4.792565
     py      :     5.446920
     dz2     :     2.098524  d :    10.543276
     dxz     :     2.151527
     dyz     :     2.078016
     dx2y2   :     2.135756
     dxy     :     2.079452
     f0      :     0.014235  f :     0.106413
     f+1     :     0.026683
     f-1     :     0.003628
     f+2     :     0.023677
     f-2     :     0.019261
     f+3     :     0.009097
     f-3     :     0.009832

21 O s       :     3.625772  s :     3.625772
     pz      :     1.630682  p :     4.718672
     px      :     1.531098
     py      :     1.556892
     dz2     :     0.010724  d :     0.061029
     dxz     :     0.020165
     dyz     :     0.006155
     dx2y2   :     0.011580
     dxy     :     0.012404
     f0      :     0.000277  f :     0.002814
     f+1     :     0.000610
     f-1     :     0.000153
     f+2     :     0.000647
     f-2     :     0.000472
     f+3     :     0.000322
     f-3     :     0.000334

22 C s       :     2.785762  s :     2.785762
     pz      :     1.049182  p :     3.000062
     px      :     0.999482
     py      :     0.951398
     dz2     :     0.100399  d :     0.489180
     dxz     :     0.151546
     dyz     :     0.090595
     dx2y2   :     0.075607
     dxy     :     0.071033
     f0      :     0.007397  f :     0.049230
     f+1     :     0.011546
     f-1     :     0.007351
     f+2     :     0.005538
     f-2     :     0.007155
     f+3     :     0.004932
     f-3     :     0.005310

23 C s       :     2.766434  s :     2.766434
     pz      :     1.023642  p :     2.939855
     px      :     1.004212
     py      :     0.912001
     dz2     :     0.099600  d :     0.389722
     dxz     :     0.123745
     dyz     :     0.067301
```

```
     dx2y2   :      0.035784
     dxy     :      0.063292
     f0      :      0.007186  f :     0.043925
     f+1     :      0.011878
     f-1     :      0.005789
     f+2     :      0.005564
     f-2     :      0.005378
     f+3     :      0.003307
     f-3     :      0.004824

24 C s       :      2.765416  s :     2.765416
     pz      :      0.981831  p :     2.944126
     px      :      1.038514
     py      :      0.923782
     dz2     :      0.096237  d :     0.390163
     dxz     :      0.087209
     dyz     :      0.044583
     dx2y2   :      0.082297
     dxy     :      0.079836
     f0      :      0.003151  f :     0.043790
     f+1     :      0.011057
     f-1     :      0.003941
     f+2     :      0.006804
     f-2     :      0.007065
     f+3     :      0.004969
     f-3     :      0.006805

25 C s       :      2.767347  s :     2.767347
     pz      :      0.978941  p :     2.942301
     px      :      1.037381
     py      :      0.925979
     dz2     :      0.096101  d :     0.386180
     dxz     :      0.086669
     dyz     :      0.044785
     dx2y2   :      0.079873
     dxy     :      0.078752
     f0      :      0.003194  f :     0.043392
     f+1     :      0.011237
     f-1     :      0.003879
     f+2     :      0.006616
     f-2     :      0.006962
     f+3     :      0.004888
     f-3     :      0.006617

26 C s       :      2.767219  s :     2.767219
     pz      :      1.031856  p :     2.941711
     px      :      0.995377
     py      :      0.914478
     dz2     :      0.096987  d :     0.385907
     dxz     :      0.122322
     dyz     :      0.067078
     dx2y2   :      0.036160
     dxy     :      0.063359
     f0      :      0.006944  f :     0.043380
     f+1     :      0.011805
     f-1     :      0.005588
     f+2     :      0.005610
     f-2     :      0.005367
     f+3     :      0.003271
     f-3     :      0.004796

27 C s       :      2.767606  s :     2.767606
     pz      :      1.026134  p :     2.944119
```

```
     px      :     0.987271
     py      :     0.930715
     dz2     :     0.088780  d :     0.384133
     dxz     :     0.119886
     dyz     :     0.074142
     dx2y2   :     0.061510
     dxy     :     0.039814
     f0      :     0.007113  f :     0.043274
     f+1     :     0.009938
     f-1     :     0.007145
     f+2     :     0.004601
     f-2     :     0.005669
     f+3     :     0.004561
     f-3     :     0.004247

28 H s       :     0.788788  s :     0.788788
     pz      :     0.015375  p :     0.060263
     px      :     0.025417
     py      :     0.019470

29 H s       :     0.787006  s :     0.787006
     pz      :     0.032790  p :     0.059896
     px      :     0.011240
     py      :     0.015867

30 H s       :     0.789564  s :     0.789564
     pz      :     0.032415  p :     0.059297
     px      :     0.011381
     py      :     0.015501

31 H s       :     0.789432  s :     0.789432
     pz      :     0.015535  p :     0.059325
     px      :     0.024090
     py      :     0.019700

32 H s       :     0.790228  s :     0.790228
     pz      :     0.017310  p :     0.059320
     px      :     0.028673
     py      :     0.013337


                      *****************************
                      * MAYER POPULATION ANALYSIS *
                      *****************************

 NA   - Mulliken gross atomic population
 ZA   - Total nuclear charge
 QA   - Mulliken gross atomic charge
 VA   - Mayer's total valence
 BVA  - Mayer's bonded valence
 FA   - Mayer's free valence

 ATOM       NA         ZA         QA         VA         BVA        FA
 0 C      5.8188     6.0000     0.1812     3.5811     3.5811    -0.0000
 1 C      6.2371     6.0000    -0.2371     3.9187     3.9187     0.0000
 2 C      6.2422     6.0000    -0.2422     3.8816     3.8816     0.0000
 3 C      6.0881     6.0000    -0.0881     3.8002     3.8002     0.0000
 4 C      6.1264     6.0000    -0.1264     3.8279     3.8279     0.0000
 5 C      6.1730     6.0000    -0.1730     3.8536     3.8536    -0.0000
 6 H      0.8785     1.0000     0.1215     0.9890     0.9890     0.0000
 7 H      0.8660     1.0000     0.1340     0.9700     0.9700     0.0000
 8 H      0.8624     1.0000     0.1376     0.9701     0.9701     0.0000
```

```
  9 H      0.8636     1.0000     0.1364     0.9650     0.9650     0.0000
 10 H      0.8645     1.0000     0.1355     0.9691     0.9691    -0.0000
 11 C      6.1797     6.0000    -0.1797     3.8456     3.8456    -0.0000
 12 H      0.8894     1.0000     0.1106     0.9619     0.9619    -0.0000
 13 H      0.8836     1.0000     0.1164     0.9636     0.9636    -0.0000
 14 C      6.2667     6.0000    -0.2667     3.7569     3.7569    -0.0000
 15 H      0.7423     1.0000     0.2577     1.0149     1.0149    -0.0000
 16 H      0.8731     1.0000     0.1269     0.9629     0.9629     0.0000
 17 C      6.3029     6.0000    -0.3029     3.8953     3.8953     0.0000
 18 H      0.8375     1.0000     0.1625     0.9604     0.9604     0.0000
 19 H      0.8389     1.0000     0.1611     0.9636     0.9636     0.0000
 20 Se    33.5879    34.0000     0.4121     2.9470     2.9470     0.0000
 21 O      8.5715     8.0000    -0.5715     1.8193     1.8193    -0.0000
 22 C      5.9308     6.0000     0.0692     3.6654     3.6654    -0.0000
 23 C      6.1869     6.0000    -0.1869     3.8184     3.8184     0.0000
 24 C      6.1903     6.0000    -0.1903     3.8440     3.8440    -0.0000
 25 C      6.1552     6.0000    -0.1552     3.8591     3.8591    -0.0000
 26 C      6.1442     6.0000    -0.1442     3.8485     3.8485     0.0000
 27 C      6.1228     6.0000    -0.1228     3.8264     3.8264    -0.0000
 28 H      0.8607     1.0000     0.1393     0.9910     0.9910     0.0000
 29 H      0.8443     1.0000     0.1557     0.9637     0.9637    -0.0000
 30 H      0.8576     1.0000     0.1424     0.9650     0.9650    -0.0000
 31 H      0.8572     1.0000     0.1428     0.9656     0.9656     0.0000
 32 H      0.8558     1.0000     0.1442     0.9631     0.9631    -0.0000

  Mayer bond orders larger than 0.100000
B(  0-C ,  1-C ) :   1.3444 B(  0-C ,  2-C ) :   1.3359 B(  0-C , 11-C ) :   0.9083
B(  1-C ,  3-C ) :   1.3959 B(  1-C ,  6-H ) :   0.9812 B(  2-C ,  4-C ) :   1.3894
B(  2-C ,  7-H ) :   0.9903 B(  3-C ,  5-C ) :   1.3722 B(  3-C ,  8-H ) :   0.9706
B(  4-C ,  5-C ) :   1.3728 B(  4-C ,  9-H ) :   0.9830 B(  5-C , 10-H ) :   0.9799
B( 11-C , 12-H ) :   0.9544 B( 11-C , 13-H ) :   0.9778 B( 11-C , 14-C ) :   0.9541
B( 14-C , 15-H ) :   0.5414 B( 14-C , 16-H ) :   0.9420 B( 14-C , 17-C ) :   1.3017
B( 15-H , 21-O ) :   0.3995 B( 17-C , 18-H ) :   0.9571 B( 17-C , 19-H ) :   0.9515
B( 17-C , 20-Se) :   0.5054 B( 20-Se, 21-O ) :   1.2610 B( 20-Se, 22-C ) :   0.9535
B( 22-C , 23-C ) :   1.3093 B( 22-C , 24-C ) :   1.3420 B( 23-C , 25-C ) :   1.3800
B( 23-C , 28-H ) :   0.9753 B( 24-C , 26-C ) :   1.3773 B( 24-C , 29-H ) :   0.9688
B( 25-C , 27-C ) :   1.3643 B( 25-C , 30-H ) :   0.9834 B( 26-C , 27-C ) :   1.3800
B( 26-C , 31-H ) :   0.9776 B( 27-C , 32-H ) :   0.9752

-------
TIMINGS
-------

Total SCF time: 0 days 0 hours 1 min 48 sec

Total time                  ....     108.908 sec
Sum of individual times     ....     105.488 sec  ( 96.9%)

SCF preparation             ....       0.621 sec  (  0.6%)
Fock matrix formation       ....     100.997 sec  ( 92.7%)
  Startup                   ....       0.042 sec  (  0.0% of F)
  Split-RI-J                ....       7.257 sec  (  7.2% of F)
  Chain of spheres X        ....      76.012 sec  ( 75.3% of F)
  XC integration            ....      10.586 sec  ( 10.5% of F)
    XC Preparation          ....       0.000 sec  (  0.0% of XC)
    Basis function eval.    ....       2.664 sec  ( 25.2% of XC)
    Density eval.           ....       3.287 sec  ( 31.0% of XC)
    XC-Functional eval.     ....       0.103 sec  (  1.0% of XC)
    XC-Potential eval.      ....       3.540 sec  ( 33.4% of XC)
  CPCM terms                ....       9.967 sec  (  9.9% of F)
Diagonalization             ....       0.000 sec  (  0.0%)
Density matrix formation    ....       0.417 sec  (  0.4%)
Total Energy calculation    ....       0.167 sec  (  0.2%)
```

```
Population analysis         ....       0.134 sec  (  0.1%)
Orbital Transformation      ....       0.336 sec  (  0.3%)
Orbital Orthonormalization  ....       0.000 sec  (  0.0%)
DIIS solution               ....       1.448 sec  (  1.3%)
SOSCF solution              ....       1.368 sec  (  1.3%)
Finished LeanSCF after 109.1 sec

Maximum memory used throughout the entire LEANSCF-calculation: 92.4 MB


-------------------------------------------------------------------------------
                          DFT DISPERSION CORRECTION

                              DFTD3 V3.1  Rev 1
                          USING Becke-Johnson damping
-------------------------------------------------------------------------------
The B3LYP functional is recognized
Active option DFTDOPT                   ...         4

molecular C6(AA) [au] = 14261.009998


            DFT-D V3
 parameters
 s6 scaling factor         :     1.0000
 a1 scaling factor         :     0.3981
 s8 scaling factor         :     1.9889
 a2 scaling factor         :     4.4211
 ad hoc parameters k1-k3   :    16.0000     1.3333    -4.0000

 Edisp/kcal,au: -50.928891344912  -0.081160345807
 E6   /kcal   : -23.541008494
 E8   /kcal   : -27.387882851

-------------------------   ----------------
Dispersion correction           -0.081160346
-------------------------   ----------------


-------------------------   --------------------
FINAL SINGLE POINT ENERGY     -3057.749354624065
-------------------------   --------------------



           ************************************************************
           *        Program running with 12 parallel MPI-processes    *
           *              working on a common directory               *
           ************************************************************
------------------------------------------------------------------------------
                         ORCA SCF GRADIENT CALCULATION
------------------------------------------------------------------------------

Nuc. rep. gradient        (SHARK) ... done (  0.0 sec)
HCore & Overlap gradient  (SHARK) ... done (  0.4 sec)
Split-RIJ-J gradient      (SHARK) ... done (  1.9 sec)
COSX-gradient                     ... done ( 35.4 sec)
XC gradient                       ... done (  5.3 sec)
CPCM gradient                     ... done (  1.9 sec)
SMD gradient                      ... done (  0.0 sec)
Dispersion correction             ... done (  0.0 sec)

-------------------
```

```
DISPERSION GRADIENT
-------------------

   1   C   :   -0.000262013    0.000910945    0.000470020
   2   C   :   -0.000213622    0.000950077    0.001023160
   3   C   :    0.000175532    0.000875128    0.000235326
   4   C   :   -0.000197993    0.000912994    0.000862118
   5   C   :    0.000191794    0.000758040    0.000151676
   6   C   :    0.000020390    0.000863662    0.000442239
   7   H   :   -0.000231864    0.000078429    0.000547157
   8   H   :    0.000049430    0.000425715   -0.000169996
   9   H   :   -0.000046348    0.000175252    0.000490412
  10   H   :    0.000177152    0.000284062   -0.000095634
  11   H   :    0.000226884    0.000274507    0.000207239
  12   C   :   -0.000666495    0.000417085   -0.000037126
  13   H   :   -0.000344022    0.000263751   -0.000024085
  14   H   :   -0.000409638    0.000137219    0.000152566
  15   C   :   -0.000730060   -0.000378901   -0.000148831
  16   H   :   -0.000017056   -0.001014749   -0.000066150
  17   H   :   -0.000355915    0.000069489   -0.000175055
  18   C   :   -0.000061074   -0.001633106   -0.000474826
  19   H   :   -0.000071585    0.000096135   -0.000305571
  20   H   :   -0.000171318    0.000080700   -0.000649498
  21   Se  :   -0.000988384   -0.000423323   -0.001652820
  22   O   :   -0.000986599    0.000457680   -0.000385258
  23   C   :    0.000663633   -0.000999863   -0.000204945
  24   C   :    0.000455299   -0.000803752    0.000003288
  25   C   :    0.000733872   -0.000700673   -0.000342873
  26   C   :    0.000522760   -0.000640821    0.000286256
  27   C   :    0.000819551   -0.000365963   -0.000283872
  28   C   :    0.000599095   -0.000479387   -0.000020541
  29   H   :   -0.000016408   -0.000374238    0.000282009
  30   H   :    0.000351078   -0.000004225   -0.000351033
  31   H   :    0.000111599   -0.000221162    0.000284961
  32   H   :    0.000334048    0.000054240   -0.000181751
  33   H   :    0.000338277   -0.000044946    0.000131440

Difference to translation invariance:
           :   -0.0000000000   -0.0000000000   -0.0000000000

Difference to rotation invariance:
           :   -0.0000000000    0.0000000000   -0.0000000000

Norm of the Dispersion gradient    ...    0.0051481187
RMS gradient                       ...    0.0005174054
MAX gradient                       ...    0.0016528204

------------------
CARTESIAN GRADIENT
------------------

   1   C   :   -0.000026611    0.000020970    0.000024082
   2   C   :    0.000001512   -0.000009484   -0.000000909
   3   C   :   -0.000007066    0.000000896    0.000005928
   4   C   :    0.000000229   -0.000004258    0.000000401
   5   C   :   -0.000007075    0.000001818    0.000003859
   6   C   :   -0.000002845   -0.000000704    0.000001256
   7   H   :    0.000000978   -0.000007829    0.000001092
   8   H   :   -0.000009398    0.000000727    0.000006731
   9   H   :    0.000000841   -0.000001611   -0.000000870
  10   H   :   -0.000009431    0.000004535    0.000004989
  11   H   :   -0.000004050    0.000003037    0.000001836
  12   C   :   -0.000001415   -0.000016293   -0.000000857
```

```
  13   H   :   -0.000004324   -0.000009121    0.000001477
  14   H   :   -0.000004040   -0.000006128    0.000004948
  15   C   :   -0.000004267    0.000002765   -0.000007611
  16   H   :    0.000014552   -0.000014260   -0.000003218
  17   H   :   -0.000001807   -0.000010209    0.000006099
  18   C   :   -0.000007932    0.000003418    0.000013315
  19   H   :    0.000000468    0.000003292   -0.000000075
  20   H   :   -0.000003671    0.000001661    0.000003777
  21   Se  :    0.000000488    0.000011017   -0.000011376
  22   O   :    0.000002315   -0.000010862    0.000011156
  23   C   :    0.000005788    0.000008553   -0.000005475
  24   C   :    0.000004241    0.000005149   -0.000003647
  25   C   :    0.000024928   -0.000023041   -0.000016429
  26   C   :    0.000007824    0.000002478   -0.000006140
  27   C   :    0.000001644    0.000010834   -0.000004228
  28   C   :    0.000003822    0.000007770   -0.000004154
  29   H   :    0.000009076   -0.000002039   -0.000006174
  30   H   :    0.000000226    0.000007828   -0.000003242
  31   H   :    0.000009610    0.000000099   -0.000006001
  32   H   :    0.000000622    0.000011003   -0.000004467
  33   H   :    0.000004768    0.000007986   -0.000006069

Difference to translation invariance:
           :   -0.0000000004   -0.0000000010    0.0000000008

Difference to rotation invariance:
           :    0.0004549683    0.0004821280    0.0011208051

Norm of the Cartesian gradient     ...    0.0000825034
RMS gradient                       ...    0.0000082919
MAX gradient                       ...    0.0000266111

-------
TIMINGS
-------

Total SCF gradient time     ....       44.941 sec

Densities                   ....        0.006 sec  (  0.0%)
One electron gradient       ....        0.403 sec  (  0.9%)
RI-J Coulomb gradient       ....        1.944 sec  (  4.3%)
COSX gradient               ....       35.397 sec  ( 78.8%)
XC gradient                 ....        5.276 sec  ( 11.7%)
CPCM gradient               ....        1.874 sec  (  4.2%)
   A-Matrix (El+Nuc)        ....        0.021 sec  (  0.0%)
   Potential                ....        1.852 sec  (  4.1%)
SMD gradient                ....        0.003 sec  (  0.0%)

Maximum memory used throughout the entire SCFGRAD-calculation: 107.1 MB


           ************************************************************
           *        Program running with 12 parallel MPI-processes    *
           *              working on a common directory               *
           ************************************************************

------------------------------------------------------------------------------
                              ORCA PROPERTY CALCULATIONS
------------------------------------------------------------------------------

GBWName                                 ... H-IRC.gbw
Number of atoms                         ...     33
Number of basis functions               ...    640
```

```
Max core memory                         ...   8000 MB

Electric properties:
Dipole moment                           ... YES
Quadrupole moment                       ...  NO
General moments                         ...  NO (MaxL=10)
Static polarizability (Dipole/Dipole)   ...  NO
Static polarizability (Dipole/Quad.)    ...  NO
Static polarizability (Quad./Quad.)     ...  NO
Static polarizability (Velocity)        ...  NO
Static hyperpolarizability              ...  NO

Atomic electric properties:
Dipole moment                           ...  NO
Quadrupole moment                       ...  NO
Static polarizability                   ...  NO

Choice of electric origin               ... Center of mass
Position of electric origin             ...    -1.452879    -3.324585   -12.733471

General magnetic properties:
Magnetizability                         ...  NO

EPR properties:
g-Tensor (aka g-matrix)                 ...  NO
Zero-Field splitting spin-orbit         ...  NO
Zero-field splitting spin-spin          ...  NO
Hyperfine couplings                     ...  NO (   0 nuclei)
Quadrupole couplings                    ...  NO (   0 nuclei)
Contact density                         ...  NO (   0 nuclei)

NMR properties:
Chemical shifts                         ...  NO (   0 nuclei)
Spin-rotation constants                 ...  NO (   0 nuclei)
Spin-spin couplings                     ...  NO (   0 nuclei,    0 pairs)

Choice of magnetic origin               ... GIAO
Position of magnetic origin             ...     0.000000     0.000000     0.000000

Properties with geometric perturbations:
SCF Hessian                             ...  NO
IR spectrum                             ...  NO
Raman spectrum                          ...  NO
Raman option                            ... 0
VCD spectrum                            ...  NO
X-ray spectroscopy properties:
SCF XES/XAS/RIXS spectra                ...  NO

SCF SOC stabilization energy            ...  NO
Diagonal Born-Oppenheimer correction    ...  NO

-------------
DIPOLE MOMENT
-------------

Method              : SCF
Type of density     : Electron Density
Multiplicity        :   1
Irrep               :   0
Energy              : -3057.6681942782579426 Eh
Basis               : AO
                                X                 Y                 Z
Electronic contribution:     -7.812413242     -30.012151155     -17.223784357
```

```
Nuclear contribution   :      8.890846736      30.678825451      16.492617327
                        -----------------------------------------
Total Dipole Moment    :      1.078433494       0.666674296      -0.731167030
                        -----------------------------------------
Magnitude (a.u.)       :      1.463584177
Magnitude (Debye)      :      3.720135335



--------------------
Rotational spectrum
--------------------

Rotational constants in cm-1:           0.015529            0.013173            0.008725
Rotational constants in MHz :         465.546021          394.902495          261.562763

Dipole components along the rotational axes:
x,y,z [a.u.] :     0.611713     0.327996    -1.288528
x,y,z [Debye]:     1.554850     0.833700    -3.275178



Dipole moment calculation done in   0.0 sec

Maximum memory used throughout the entire PROP-calculation: 54.3 MB


--------------------------------------------------------------------------------
                   Intrinsic Reaction Coordinate Calculation
--------------------------------------------------------------------------------

IRC Settings:
  Nr. of atoms                          .... 33
  Algorithm                             .... LQA
  Coordinate system following type      .... Mass-Weighted
  Max. no of cycles (MaxIter)           .... 241
  Direction                             .... Forward and backward
  Steep Lenght                          .... 0.110 bohr Dalton^(1/2)

Initial displacement:
  Hessian type                          .... Read
  Hessian Filename                      .... TS-H_Compound_3.hess
  Following Hessian mode                .... 0
  Initial displacement type             .... Length
    Scaling normalized TS mode by       .... 0.040

Convergence Tolerances:
  Energy Change                         ....  5.0000e-06 Eh
  Max. Gradient                         ....  8.0000e-05 Eh/bohr
  RMS Gradient                          ....  1.0000e-05 Eh/bohr
  Max. Displacement                     ....  4.0000e-03 bohr
  RMS Displacement                      ....  2.0000e-03 bohr
  Strict Convergency criteria enforced

LQA Settings:
  Hessian update type                   .... Bofill
  Update steep lenght                   .... NO
  t Stepsize                            .... Gradient-based adjusted
  t MAX int. Steps                      .... 1000000
  Bulirsch-Stoer Correction             .... YES
    Euler Initial Steps                 .... 100
    Richardson Max extrapolations       .... 20
    Richardson error threshould         ....  1.0000e-06
```

```
    Apply BS correction on TS disp.   .... NO
    Keep LQA direction                .... NO
    LQA direction Theshould           ....  1.0000e-01
    Update Hessian and Gradient       .... YES

Storing full IRC trajectory in        .... H-IRC_IRC_Full_trj.xyz
Storing forward trajectory in         .... H-IRC_IRC_F_trj.xyz
Storing backward trajectory in        .... H-IRC_IRC_B_trj.xyz
Storing IRC profile in                .... H-IRC.IRC.out


         *************************************************************
         *                          FORWARD IRC                      *
         *************************************************************

Iteration    E(Eh)      dE(kcal/mol)  max(|G|)   RMS(G)    max(|S|)   RMS(S)   MW-Displ.
Convergence thresholds    0.003138    0.000080  0.000010  0.004000  0.002000
    0     -3057.749399   -0.027604    0.002308  0.000510  0.034475  0.004020  0.046448
    1     -3057.749795   -0.276493    0.007462  0.001481  0.074272  0.008971  0.110002
    2     -3057.750405   -0.659232    0.011629  0.002160  0.061828  0.008102  0.110003
    3     -3057.751090   -1.089100    0.014372  0.002583  0.044361  0.007016  0.110001
    4     -3057.751807   -1.538621    0.016021  0.002848  0.031624  0.006193  0.110000
    5     -3057.752543   -2.000746    0.017460  0.003027  0.030054  0.005663  0.110002
    6     -3057.753300   -2.475519    0.018814  0.003158  0.028580  0.005323  0.110003
    7     -3057.754068   -2.957628    0.019943  0.003262  0.027323  0.005096  0.110001
    8     -3057.754848   -3.447422    0.020919  0.003350  0.026251  0.004938  0.110000
    9     -3057.755641   -3.944972    0.021782  0.003427  0.025319  0.004821  0.110003
   10     -3057.756443   -4.448063    0.022559  0.003496  0.024495  0.004732  0.110002
   11     -3057.757255   -4.957290    0.023264  0.003559  0.023752  0.004661  0.110000
   12     -3057.758075   -5.471992    0.023906  0.003616  0.023078  0.004604  0.110001
   13     -3057.758901   -5.990680    0.024488  0.003668  0.022458  0.004558  0.110001
   14     -3057.759737   -6.514734    0.025012  0.003714  0.021884  0.004521  0.110002
   15     -3057.760580   -7.043880    0.025475  0.003753  0.021351  0.004490  0.110001
   16     -3057.761426   -7.574980    0.025873  0.003786  0.020855  0.004465  0.110004
   17     -3057.762276   -8.108550    0.026203  0.003811  0.020385  0.004444  0.110003
   18     -3057.763129   -8.643628    0.026462  0.003828  0.019948  0.004428  0.110002
   19     -3057.763983   -9.179284    0.026646  0.003837  0.019544  0.004415  0.110003
   20     -3057.764836   -9.714456    0.026754  0.003837  0.019175  0.004405  0.110001
   21     -3057.765686   -10.248135    0.026783  0.003827  0.018835  0.004397  0.110002
   22     -3057.766525   -10.774657    0.026729  0.003808  0.018503  0.004390  0.110000
   23     -3057.767373   -11.306416    0.026600  0.003778  0.018131  0.004383  0.110001
   24     -3057.768191   -11.819931    0.026381  0.003737  0.017727  0.004374  0.110003
   25     -3057.769006   -12.331700    0.026087  0.003686  0.017220  0.004362  0.110002
   26     -3057.769806   -12.833416    0.025715  0.003626  0.016730  0.004346  0.110001
   27     -3057.770584   -13.321675    0.025269  0.003559  0.016183  0.004317  0.110002
   28     -3057.771337   -13.794155    0.024739  0.003483  0.015697  0.004266  0.110000
   29     -3057.772068   -14.252724    0.024120  0.003394  0.015432  0.004228  0.110003
   30     -3057.772778   -14.698114    0.023423  0.003296  0.015262  0.004219  0.110001
   31     -3057.773463   -15.128469    0.022652  0.003190  0.014858  0.004208  0.110002
   32     -3057.774123   -15.542458    0.021808  0.003075  0.014453  0.004187  0.110003
   33     -3057.774755   -15.938823    0.020893  0.002953  0.014082  0.004165  0.110003
   34     -3057.775357   -16.316907    0.019910  0.002823  0.014169  0.004144  0.110002
   35     -3057.775930   -16.676550    0.018863  0.002687  0.014283  0.004124  0.110003
   36     -3057.776473   -17.017202    0.017757  0.002544  0.014418  0.004103  0.110002
   37     -3057.776984   -17.337686    0.016598  0.002396  0.014575  0.004082  0.110001
   38     -3057.777463   -17.638172    0.015393  0.002243  0.014749  0.004060  0.110000
   39     -3057.777910   -17.918692    0.014152  0.002089  0.014963  0.004043  0.110001
   40     -3057.778326   -18.179626    0.012885  0.001930  0.015163  0.004024  0.110000
   41     -3057.778708   -18.419377    0.011612  0.001780  0.015417  0.004015  0.110000
   42     -3057.779064   -18.642946    0.010333  0.001614  0.015590  0.004008  0.110002
   43     -3057.779380   -18.841139    0.009104  0.001499  0.015913  0.004026  0.110001
   44     -3057.779691   -19.036341    0.007854  0.001294  0.015822  0.004090  0.110002
   45     -3057.779951   -19.199354    0.006836  0.001372  0.017614  0.004939  0.110000
```

46	-3057.780227	-19.372495	0.006346	0.001033	0.015595	0.004181	0.110004
47	-3057.780458	-19.517758	0.005788	0.000987	0.017776	0.004659	0.110000
48	-3057.780681	-19.657858	0.005303	0.000829	0.016407	0.004470	0.110002
49	-3057.780896	-19.792761	0.004834	0.000715	0.018094	0.004923	0.110001
50	-3057.781096	-19.917963	0.004401	0.000662	0.020030	0.005241	0.110001
51	-3057.781275	-20.030414	0.004055	0.000687	0.020740	0.005406	0.110002
52	-3057.781445	-20.137220	0.003746	0.000699	0.020635	0.005464	0.110000
53	-3057.781608	-20.239523	0.003517	0.000736	0.021053	0.005547	0.110001
54	-3057.781767	-20.339291	0.004044	0.000742	0.021324	0.005438	0.110000
55	-3057.781924	-20.437707	0.004055	0.000703	0.021850	0.005404	0.110001
56	-3057.782077	-20.533629	0.003962	0.000672	0.022334	0.005565	0.110000
57	-3057.782228	-20.628544	0.003934	0.000661	0.022722	0.005714	0.110000
58	-3057.782376	-20.721248	0.003990	0.000674	0.024404	0.005808	0.110001
59	-3057.782518	-20.810140	0.003950	0.000691	0.024852	0.005789	0.110001
60	-3057.782660	-20.899100	0.003785	0.000678	0.023995	0.005758	0.110001
61	-3057.782800	-20.987109	0.003786	0.000671	0.023916	0.005829	0.110000
62	-3057.782937	-21.073427	0.003954	0.000679	0.023962	0.005860	0.110001
63	-3057.783072	-21.158227	0.004073	0.000679	0.023670	0.005815	0.110001
64	-3057.783205	-21.241205	0.004017	0.000668	0.023471	0.005775	0.110001
65	-3057.783334	-21.322369	0.003996	0.000667	0.023696	0.005799	0.110000
66	-3057.783461	-21.401783	0.003962	0.000665	0.023515	0.005770	0.110000
67	-3057.783585	-21.479711	0.003938	0.000661	0.023231	0.005747	0.110001
68	-3057.783706	-21.555970	0.003932	0.000657	0.023054	0.005730	0.110001
69	-3057.783825	-21.630612	0.003920	0.000654	0.022847	0.005705	0.110000
70	-3057.783942	-21.703739	0.003897	0.000649	0.022617	0.005673	0.110000
71	-3057.784056	-21.775582	0.003873	0.000645	0.022448	0.005653	0.110001
72	-3057.784168	-21.845581	0.003858	0.000642	0.022292	0.005635	0.110001
73	-3057.784277	-21.914020	0.003839	0.000639	0.022114	0.005610	0.110001
74	-3057.784384	-21.980904	0.003818	0.000635	0.021948	0.005583	0.110000
75	-3057.784487	-22.046153	0.003796	0.000631	0.021780	0.005555	0.110000
76	-3057.784589	-22.109744	0.003771	0.000626	0.021592	0.005525	0.110001
77	-3057.784687	-22.171632	0.003745	0.000622	0.021397	0.005492	0.110000
78	-3057.784783	-22.231742	0.003717	0.000618	0.021189	0.005455	0.110000
79	-3057.784876	-22.290053	0.003685	0.000613	0.020960	0.005414	0.110000
80	-3057.784966	-22.346459	0.003650	0.000607	0.020827	0.005368	0.110000
81	-3057.785053	-22.400924	0.003609	0.000601	0.020760	0.005316	0.110000
82	-3057.785136	-22.453317	0.003567	0.000595	0.020677	0.005260	0.110000
83	-3057.785216	-22.503580	0.003517	0.000588	0.020483	0.005190	0.110000
84	-3057.785293	-22.551674	0.003463	0.000581	0.020347	0.005123	0.110000
85	-3057.785366	-22.597497	0.003404	0.000573	0.020063	0.005049	0.110000
86	-3057.785436	-22.641087	0.003337	0.000564	0.019801	0.004967	0.110000
87	-3057.785501	-22.682364	0.003270	0.000555	0.019478	0.004882	0.110000
88	-3057.785564	-22.721399	0.003190	0.000545	0.019119	0.004790	0.110000
89	-3057.785622	-22.758083	0.003111	0.000534	0.018690	0.004695	0.110000
90	-3057.785677	-22.792636	0.003014	0.000520	0.018275	0.004596	0.110000
91	-3057.785728	-22.824855	0.002925	0.000507	0.017677	0.004498	0.110000
92	-3057.785777	-22.855427	0.002699	0.000480	0.017051	0.004367	0.110000
93	-3057.785824	-22.884993	0.002123	0.000430	0.016236	0.004127	0.110000
94	-3057.785870	-22.913579	0.001395	0.000368	0.016317	0.004010	0.110000
95	-3057.785907	-22.936919	0.001667	0.000388	0.017417	0.004128	0.110000
96	-3057.785951	-22.964240	0.001154	0.000326	0.018237	0.004209	0.110001
97	-3057.785963	-22.972015	0.003316	0.000587	0.019404	0.004283	0.110000
98	-3057.785973	-22.978303	0.003838	0.000649	0.022699	0.003001	0.110000
99	-3057.786021	-23.008703	0.001942	0.000446	0.018246	0.004349	0.110001
100	-3057.786074	-23.041472	0.000994	0.000181	0.020993	0.004955	0.110001
101	-3057.786097	-23.055941	0.001620	0.000368	0.017979	0.005318	0.110001
102	-3057.786140	-23.083121	0.000879	0.000229	0.012264	0.003944	0.110001
103	-3057.786178	-23.107220	0.000868	0.000171	0.014206	0.004417	0.110000
104	-3057.786206	-23.124766	0.001054	0.000321	0.014986	0.004586	0.110000
105	-3057.786239	-23.145223	0.000870	0.000285	0.012571	0.004110	0.110000
106	-3057.786268	-23.163336	0.001083	0.000269	0.012135	0.004144	0.110000
107	-3057.786295	-23.180611	0.001582	0.000298	0.012292	0.004122	0.110000
108	-3057.786324	-23.198559	0.001492	0.000282	0.011576	0.003943	0.110000

109	-3057.786350	-23.215096	0.001369	0.000284	0.011535	0.003929	0.110000
110	-3057.786377	-23.231629	0.001271	0.000282	0.011257	0.003849	0.110000
111	-3057.786403	-23.248090	0.001323	0.000280	0.011402	0.003843	0.110000
112	-3057.786428	-23.263677	0.001444	0.000278	0.010849	0.003865	0.110000
113	-3057.786452	-23.278628	0.001540	0.000285	0.011878	0.003868	0.110000
114	-3057.786477	-23.294401	0.001542	0.000284	0.011306	0.003874	0.110000
115	-3057.786501	-23.309525	0.001544	0.000285	0.011759	0.003866	0.110000
116	-3057.786525	-23.324627	0.001547	0.000282	0.011201	0.003871	0.110000
117	-3057.786548	-23.339113	0.001549	0.000283	0.011649	0.003834	0.110000
118	-3057.786572	-23.354052	0.001533	0.000278	0.011044	0.003840	0.110000
119	-3057.786595	-23.368481	0.001519	0.000279	0.011230	0.003846	0.110000
120	-3057.786617	-23.382428	0.001531	0.000282	0.011621	0.003815	0.110000
121	-3057.786640	-23.396961	0.001551	0.000280	0.011009	0.003835	0.110000
122	-3057.786663	-23.411219	0.001567	0.000283	0.011115	0.003852	0.110000
123	-3057.786685	-23.424833	0.001575	0.000285	0.011549	0.003818	0.110000
124	-3057.786707	-23.439148	0.001587	0.000284	0.011381	0.003848	0.110000
125	-3057.786730	-23.453251	0.001580	0.000283	0.011200	0.003852	0.110000
126	-3057.786751	-23.466731	0.001574	0.000284	0.011588	0.003816	0.110000
127	-3057.786774	-23.480842	0.001584	0.000283	0.011306	0.003849	0.110000
128	-3057.786796	-23.494762	0.001563	0.000281	0.011215	0.003858	0.110000
129	-3057.786817	-23.508113	0.001581	0.000285	0.011629	0.003842	0.110000
130	-3057.786840	-23.522145	0.001585	0.000283	0.011445	0.003866	0.110000
131	-3057.786861	-23.535821	0.001583	0.000283	0.011418	0.003883	0.110000
132	-3057.786883	-23.549306	0.001592	0.000286	0.011654	0.003859	0.110000
133	-3057.786905	-23.563244	0.001598	0.000285	0.011558	0.003880	0.110000
134	-3057.786927	-23.576677	0.001553	0.000280	0.011562	0.003840	0.110000
135	-3057.786948	-23.590299	0.001639	0.000291	0.011794	0.003914	0.110000
136	-3057.786955	-23.594718	0.001336	0.000247	0.014441	0.002440	0.110000
137	-3057.786977	-23.608375	0.001082	0.000225	0.011985	0.004223	0.110002
138	-3057.787005	-23.625834	0.000811	0.000141	0.013801	0.004547	0.110000
139	-3057.787030	-23.641641	0.000668	0.000129	0.015764	0.004621	0.110000
140	-3057.787050	-23.653996	0.000698	0.000174	0.013943	0.004619	0.110000
141	-3057.787077	-23.670943	0.000786	0.000216	0.014964	0.004469	0.110000
142	-3057.787101	-23.685894	0.000867	0.000236	0.014627	0.004376	0.110000
143	-3057.787124	-23.700391	0.001216	0.000268	0.014507	0.004365	0.110000
144	-3057.787140	-23.710391	0.001434	0.000295	0.013857	0.004259	0.110000
145	-3057.787169	-23.728877	0.001366	0.000287	0.013647	0.004113	0.110000
146	-3057.787193	-23.743722	0.001250	0.000260	0.012103	0.004063	0.110000
147	-3057.787215	-23.757546	0.001248	0.000253	0.012573	0.004189	0.110000
148	-3057.787237	-23.771733	0.001359	0.000265	0.012753	0.004205	0.110000
149	-3057.787259	-23.785114	0.001533	0.000286	0.012283	0.004201	0.110000
150	-3057.787280	-23.798574	0.001572	0.000293	0.012604	0.004175	0.110000
151	-3057.787307	-23.815279	0.001479	0.000281	0.012055	0.004144	0.110000
152	-3057.787330	-23.829606	0.001356	0.000266	0.012536	0.004224	0.110000
153	-3057.787352	-23.843390	0.001184	0.000247	0.012672	0.004223	0.110000
154	-3057.787370	-23.855064	0.001152	0.000248	0.013697	0.004325	0.110000
155	-3057.787398	-23.872224	0.001134	0.000253	0.013748	0.004320	0.110000
156	-3057.787420	-23.886321	0.001143	0.000254	0.012544	0.004239	0.110000
157	-3057.787442	-23.900053	0.001233	0.000261	0.012182	0.004236	0.110000
158	-3057.787464	-23.914096	0.001307	0.000267	0.012306	0.004229	0.110000
159	-3057.787483	-23.926161	0.001366	0.000270	0.012244	0.004206	0.110000
160	-3057.787501	-23.936890	0.001510	0.000283	0.012556	0.004227	0.110000
161	-3057.787531	-23.955823	0.001593	0.000291	0.013070	0.004215	0.110000
162	-3057.787553	-23.969949	0.001215	0.000228	0.012343	0.004020	0.110000
163	-3057.787578	-23.985345	0.001226	0.000211	0.012837	0.004389	0.110000
164	-3057.787601	-23.999972	0.001485	0.000237	0.013010	0.004480	0.110000
165	-3057.787623	-24.013856	0.001804	0.000286	0.012761	0.004402	0.110000
166	-3057.787636	-24.022134	0.001713	0.000283	0.012379	0.004254	0.110000
167	-3057.787667	-24.041084	0.001594	0.000274	0.014249	0.004288	0.110000
168	-3057.787689	-24.055229	0.001454	0.000271	0.012708	0.004308	0.110000
169	-3057.787710	-24.068488	0.001439	0.000275	0.010685	0.004185	0.110000
170	-3057.787733	-24.082613	0.001446	0.000276	0.012152	0.004199	0.110000
171	-3057.787751	-24.093873	0.001427	0.000270	0.010382	0.004118	0.110000

172	-3057.787775	-24.109017	0.001477	0.000274	0.012355	0.004217	0.110000
173	-3057.787800	-24.124487	0.001711	0.000294	0.011672	0.004215	0.110000
174	-3057.787820	-24.137235	0.001841	0.000299	0.010577	0.004128	0.110000
175	-3057.787843	-24.151855	0.001890	0.000296	0.010611	0.004152	0.110000
176	-3057.787866	-24.165981	0.001908	0.000293	0.010954	0.004171	0.110000
177	-3057.787888	-24.180146	0.001809	0.000279	0.010710	0.004200	0.110000
178	-3057.787911	-24.194528	0.001693	0.000268	0.011340	0.004230	0.110000
179	-3057.787932	-24.207794	0.001466	0.000253	0.011806	0.004251	0.110000
180	-3057.787954	-24.221687	0.001046	0.000229	0.012524	0.004269	0.110000
181	-3057.787979	-24.237214	0.000878	0.000217	0.013741	0.004306	0.110000
182	-3057.787998	-24.249014	0.001010	0.000221	0.012243	0.004250	0.110000
183	-3057.788020	-24.262865	0.000980	0.000220	0.011511	0.004199	0.110000
184	-3057.788042	-24.276663	0.000888	0.000227	0.010960	0.004206	0.110000
185	-3057.788064	-24.290514	0.000946	0.000234	0.010337	0.004174	0.110000
186	-3057.788086	-24.304137	0.000984	0.000231	0.010107	0.004168	0.110000
187	-3057.788108	-24.317951	0.000858	0.000221	0.010587	0.004206	0.110000
188	-3057.788131	-24.332384	0.000772	0.000210	0.010957	0.004237	0.110000
189	-3057.788152	-24.345373	0.000857	0.000209	0.012480	0.004290	0.110000
190	-3057.788174	-24.359748	0.000918	0.000216	0.011841	0.004271	0.110000
191	-3057.788194	-24.371872	0.000877	0.000220	0.011186	0.004215	0.110000
192	-3057.788219	-24.387701	0.000839	0.000218	0.012773	0.004275	0.110000
193	-3057.788237	-24.399186	0.000838	0.000227	0.011213	0.004260	0.110000
194	-3057.788259	-24.412878	0.000845	0.000222	0.012455	0.004252	0.110000
195	-3057.788281	-24.426487	0.000865	0.000218	0.011832	0.004266	0.110000
196	-3057.788302	-24.440042	0.000909	0.000217	0.011761	0.004251	0.110000
197	-3057.788324	-24.453831	0.000890	0.000216	0.011881	0.004252	0.110000
198	-3057.788346	-24.467640	0.000845	0.000216	0.011862	0.004260	0.110000
199	-3057.788368	-24.481384	0.000804	0.000216	0.011904	0.004265	0.110000
200	-3057.788390	-24.495187	0.000744	0.000217	0.011992	0.004277	0.110000
201	-3057.788412	-24.508782	0.000796	0.000212	0.011908	0.004278	0.110000
202	-3057.788434	-24.522747	0.000838	0.000211	0.012540	0.004304	0.110000
203	-3057.788456	-24.536602	0.000860	0.000213	0.012953	0.004327	0.110000
204	-3057.788478	-24.550456	0.000873	0.000217	0.013087	0.004333	0.110000
205	-3057.788500	-24.564261	0.000887	0.000218	0.013104	0.004338	0.110000
206	-3057.788522	-24.578020	0.000931	0.000219	0.012908	0.004339	0.110000
207	-3057.788545	-24.592328	0.000965	0.000220	0.012996	0.004337	0.110000
208	-3057.788567	-24.606049	0.001010	0.000221	0.012730	0.004332	0.110000
209	-3057.788589	-24.619858	0.001047	0.000222	0.012883	0.004340	0.110000
210	-3057.788611	-24.633589	0.001093	0.000225	0.012903	0.004344	0.110000
211	-3057.788633	-24.647409	0.001115	0.000226	0.013063	0.004348	0.110000
212	-3057.788678	-24.676017	0.001144	0.000230	0.012977	0.004346	0.110000
213	-3057.788660	-24.664274	0.000834	0.000208	0.011673	0.002084	0.110000
214	-3057.788681	-24.677670	0.000679	0.000167	0.012500	0.004329	0.110001
215	-3057.788706	-24.693564	0.000696	0.000213	0.011473	0.004800	0.110000
216	-3057.788725	-24.705373	0.001157	0.000273	0.009833	0.004402	0.110000
217	-3057.788749	-24.720116	0.001044	0.000256	0.011013	0.004190	0.110000
218	-3057.788770	-24.733611	0.000973	0.000250	0.009914	0.004203	0.110000
219	-3057.788792	-24.747240	0.000989	0.000244	0.009650	0.004195	0.110000
220	-3057.788814	-24.760815	0.000965	0.000241	0.009292	0.004203	0.110000
221	-3057.788835	-24.774308	0.000937	0.000240	0.009415	0.004219	0.110000
222	-3057.788857	-24.787798	0.000942	0.000239	0.009415	0.004229	0.110000
223	-3057.788878	-24.801184	0.000978	0.000238	0.009479	0.004236	0.110000
224	-3057.788899	-24.814429	0.000999	0.000240	0.010084	0.004245	0.110000
225	-3057.788920	-24.827513	0.000991	0.000244	0.010666	0.004265	0.110000
226	-3057.788940	-24.840455	0.000978	0.000246	0.010999	0.004282	0.110000
227	-3057.788961	-24.853207	0.000980	0.000246	0.011136	0.004286	0.110000
228	-3057.788981	-24.865744	0.000996	0.000245	0.011117	0.004276	0.110000
229	-3057.789000	-24.878077	0.001006	0.000245	0.010947	0.004254	0.110000
230	-3057.789020	-24.890207	0.001005	0.000244	0.010493	0.004236	0.110000
231	-3057.789039	-24.902249	0.001003	0.000244	0.009946	0.004225	0.110000
232	-3057.789058	-24.914198	0.001006	0.000244	0.009504	0.004217	0.110000
233	-3057.789077	-24.926031	0.001006	0.000243	0.009309	0.004212	0.110000
234	-3057.789095	-24.937612	0.001008	0.000244	0.009271	0.004210	0.110000

235	-3057.789114	-24.949120	0.001006	0.000244	0.009350	0.004202	0.110000
236	-3057.789132	-24.960454	0.000994	0.000239	0.009114	0.004188	0.110000
237	-3057.789149	-24.971418	0.001006	0.000245	0.009427	0.004193	0.110000
238	-3057.789167	-24.982518	0.000984	0.000235	0.009469	0.004170	0.110000
239	-3057.789184	-24.993023	0.001000	0.000248	0.008964	0.004166	0.110000
240	-3057.789201	-25.004194	0.000881	0.000204	0.009806	0.004144	0.110000

```
************************************************************
*  MAXIMUM NUMBER OF ITERATIONS REACHED - STOPPING IRC RUN  *
************************************************************


************************************************************
*                        BACKWARD IRC                       *
************************************************************
```

Iteration	E(Eh)	dE(kcal/mol)	max(|G|)	RMS(G)	max(|S|)	RMS(S)	MW-Displ.
Convergence thresholds		0.003138	0.000080	0.000010	0.004000	0.002000	
0	-3057.749405	-0.031642	0.002324	0.000551	0.034475	0.004020	0.046448
1	-3057.750078	-0.453681	0.008318	0.002127	0.085537	0.009822	0.110001
2	-3057.751687	-1.463319	0.014715	0.003721	0.087688	0.010008	0.110004
3	-3057.753892	-2.847236	0.019172	0.004728	0.085823	0.009882	0.110000
4	-3057.756078	-4.219249	0.022757	0.005182	0.077762	0.009303	0.110001
5	-3057.757895	-5.359306	0.024838	0.005265	0.057688	0.007941	0.110003
6	-3057.759398	-6.302204	0.024950	0.005125	0.031488	0.006376	0.110003
7	-3057.760736	-7.141614	0.024584	0.004902	0.025494	0.005544	0.110003
8	-3057.761957	-7.908065	0.024807	0.004672	0.021075	0.005070	0.110002
9	-3057.763088	-8.617763	0.024864	0.004462	0.019439	0.004751	0.110001
10	-3057.764148	-9.282740	0.024797	0.004273	0.019399	0.004536	0.110002
11	-3057.765146	-9.909434	0.024631	0.004103	0.019203	0.004405	0.110003
12	-3057.766097	-10.505927	0.024383	0.003950	0.019143	0.004337	0.110004
13	-3057.767004	-11.075432	0.024071	0.003810	0.019860	0.004311	0.110001
14	-3057.767875	-11.621808	0.023711	0.003681	0.020280	0.004311	0.110000
15	-3057.768714	-12.148227	0.023315	0.003561	0.020481	0.004326	0.110002
16	-3057.769520	-12.654112	0.022893	0.003446	0.020535	0.004348	0.110003
17	-3057.770299	-13.142906	0.022453	0.003337	0.020489	0.004372	0.110001
18	-3057.771052	-13.614990	0.022004	0.003232	0.020374	0.004398	0.110003
19	-3057.771777	-14.070233	0.021549	0.003132	0.020200	0.004422	0.110001
20	-3057.772477	-14.509760	0.021091	0.003036	0.019987	0.004444	0.110003
21	-3057.773154	-14.934160	0.020634	0.002944	0.019749	0.004464	0.110001
22	-3057.773807	-15.343781	0.020181	0.002858	0.019489	0.004481	0.110002
23	-3057.774432	-15.736152	0.019742	0.002782	0.019188	0.004495	0.110003
24	-3057.775027	-16.109344	0.019330	0.002729	0.018755	0.004487	0.110000
25	-3057.775590	-16.463114	0.018928	0.002684	0.018077	0.004390	0.110003
26	-3057.776128	-16.800606	0.018506	0.002606	0.017459	0.004292	0.110003
27	-3057.776647	-17.126122	0.018085	0.002534	0.017241	0.004293	0.110002
28	-3057.777145	-17.438959	0.017664	0.002466	0.017438	0.004277	0.110000
29	-3057.777624	-17.739554	0.017250	0.002400	0.017583	0.004254	0.110002
30	-3057.778085	-18.028422	0.016844	0.002334	0.017721	0.004236	0.110000
31	-3057.778527	-18.306120	0.016445	0.002269	0.017857	0.004220	0.110000
32	-3057.778953	-18.573076	0.016053	0.002208	0.017978	0.004204	0.110000
33	-3057.779362	-18.829908	0.015671	0.002148	0.018072	0.004184	0.110000
34	-3057.779756	-19.076825	0.015291	0.002091	0.018145	0.004166	0.110002
35	-3057.780134	-19.314328	0.014909	0.002034	0.018202	0.004146	0.110000
36	-3057.780498	-19.542504	0.014534	0.001981	0.018232	0.004127	0.110002
37	-3057.780848	-19.762443	0.014164	0.001923	0.018274	0.004106	0.110000
38	-3057.781184	-19.973403	0.013803	0.001876	0.018294	0.004092	0.110002
39	-3057.781509	-20.176996	0.013446	0.001822	0.018272	0.004066	0.110001
40	-3057.781820	-20.371983	0.013097	0.001779	0.018276	0.004050	0.110002
41	-3057.782121	-20.560912	0.012753	0.001723	0.018253	0.004025	0.110000
42	-3057.782408	-20.741074	0.012418	0.001688	0.018268	0.004016	0.110002
43	-3057.782688	-20.916823	0.012085	0.001629	0.018188	0.003984	0.110000
44	-3057.782952	-21.082686	0.011765	0.001609	0.018212	0.003980	0.110001

45	-3057.783215	-21.247644	0.011445	0.001531	0.018103	0.003943	0.110002
46	-3057.783456	-21.398885	0.011132	0.001547	0.018163	0.003958	0.110001
47	-3057.783708	-21.557012	0.010763	0.001413	0.018013	0.003918	0.110001
48	-3057.783938	-21.701097	0.010413	0.001430	0.018440	0.004035	0.110001
49	-3057.784166	-21.844664	0.010124	0.001359	0.018327	0.003945	0.110001
50	-3057.784368	-21.971126	0.009846	0.001401	0.018106	0.003922	0.110000
51	-3057.784592	-22.112004	0.009559	0.001250	0.017996	0.003881	0.110001
52	-3057.784798	-22.240697	0.009245	0.001223	0.018685	0.004054	0.110001
53	-3057.784980	-22.355232	0.008996	0.001314	0.018551	0.003982	0.110001
54	-3057.785187	-22.485286	0.008736	0.001131	0.017814	0.003863	0.110001
55	-3057.785391	-22.613242	0.008418	0.001034	0.019484	0.004234	0.110001
56	-3057.785558	-22.718095	0.008067	0.001257	0.020396	0.004625	0.110001
57	-3057.785745	-22.835566	0.007767	0.001033	0.015802	0.003642	0.110001
58	-3057.785933	-22.953254	0.007569	0.000921	0.019282	0.004226	0.110000
59	-3057.786077	-23.043608	0.007401	0.001170	0.020458	0.004424	0.110002
60	-3057.786249	-23.151726	0.007203	0.000910	0.017963	0.003934	0.110000
61	-3057.786409	-23.251791	0.006934	0.000980	0.020367	0.004534	0.110001
62	-3057.786545	-23.337331	0.006650	0.001050	0.017546	0.003935	0.110001
63	-3057.786684	-23.424522	0.006439	0.001012	0.017467	0.003797	0.110001
64	-3057.786820	-23.509532	0.006266	0.000978	0.017638	0.003844	0.110000
65	-3057.786949	-23.591016	0.006092	0.000962	0.017762	0.003862	0.110001
66	-3057.787074	-23.669108	0.005916	0.000955	0.017655	0.003834	0.110000
67	-3057.787194	-23.744483	0.005743	0.000943	0.017473	0.003795	0.110001
68	-3057.787310	-23.817421	0.005579	0.000928	0.017344	0.003771	0.110001
69	-3057.787422	-23.887838	0.005423	0.000914	0.017231	0.003754	0.110000
70	-3057.787531	-23.955829	0.005271	0.000902	0.017092	0.003736	0.110000
71	-3057.787635	-24.021505	0.005125	0.000891	0.016933	0.003715	0.110001
72	-3057.787737	-24.084981	0.004984	0.000880	0.016763	0.003693	0.110000
73	-3057.787834	-24.146338	0.004848	0.000870	0.016585	0.003673	0.110000
74	-3057.787929	-24.205684	0.004717	0.000860	0.016398	0.003653	0.110000
75	-3057.788020	-24.263068	0.004590	0.000851	0.016208	0.003633	0.110000
76	-3057.788109	-24.318502	0.004546	0.000843	0.016043	0.003618	0.110000
77	-3057.788194	-24.372129	0.004535	0.000833	0.015795	0.003592	0.110000
78	-3057.788277	-24.424085	0.004529	0.000824	0.015636	0.003577	0.110000
79	-3057.788357	-24.474344	0.004541	0.000818	0.015431	0.003562	0.110000
80	-3057.788435	-24.523231	0.004523	0.000809	0.015093	0.003533	0.110000
81	-3057.788511	-24.570647	0.004511	0.000801	0.014890	0.003522	0.110000
82	-3057.788584	-24.616663	0.004500	0.000793	0.014630	0.003506	0.110000
83	-3057.788655	-24.661339	0.004480	0.000785	0.014381	0.003490	0.110000
84	-3057.788724	-24.704757	0.004449	0.000776	0.014141	0.003476	0.110000
85	-3057.788792	-24.746983	0.004399	0.000766	0.013909	0.003463	0.110000
86	-3057.788857	-24.788117	0.004318	0.000752	0.013681	0.003451	0.110000
87	-3057.788921	-24.828255	0.004191	0.000735	0.013438	0.003442	0.110000
88	-3057.788984	-24.867476	0.004027	0.000713	0.013142	0.003439	0.110000
89	-3057.789044	-24.905609	0.003869	0.000693	0.012803	0.003446	0.110000
90	-3057.789103	-24.942405	0.003785	0.000680	0.012441	0.003455	0.110000
91	-3057.789159	-24.977632	0.003813	0.000679	0.012104	0.003448	0.110000
92	-3057.789213	-25.011381	0.003909	0.000683	0.011879	0.003413	0.110000
93	-3057.789265	-25.044253	0.003918	0.000677	0.011839	0.003352	0.110000
94	-3057.789317	-25.076543	0.003789	0.000657	0.011860	0.003321	0.110000
95	-3057.789367	-25.108226	0.003577	0.000632	0.011766	0.003347	0.110000
96	-3057.789418	-25.139828	0.003138	0.000583	0.011556	0.003374	0.110000
97	-3057.789466	-25.170393	0.002729	0.000543	0.011035	0.003487	0.110000
98	-3057.789513	-25.199396	0.002549	0.000525	0.011392	0.003584	0.110000
99	-3057.789556	-25.226570	0.002715	0.000538	0.012313	0.003621	0.110000
100	-3057.789595	-25.251386	0.003277	0.000590	0.012064	0.003575	0.110000
101	-3057.789609	-25.259816	0.003393	0.000568	0.011912	0.001898	0.110000
102	-3057.789647	-25.283712	0.002176	0.000468	0.017452	0.004510	0.110001
103	-3057.789698	-25.315753	0.002081	0.000349	0.016772	0.004077	0.110001
104	-3057.789736	-25.339373	0.002016	0.000342	0.017326	0.004281	0.110000
105	-3057.789772	-25.362170	0.001976	0.000405	0.015618	0.003919	0.110001
106	-3057.789811	-25.386681	0.001892	0.000379	0.012042	0.003562	0.110000
107	-3057.789847	-25.409263	0.001841	0.000373	0.014103	0.003715	0.110000

108	-3057.789881	-25.430439	0.001786	0.000382	0.012377	0.003633	0.110000
109	-3057.789913	-25.450581	0.001959	0.000361	0.010176	0.003539	0.110000
110	-3057.789948	-25.472863	0.002090	0.000357	0.010415	0.003635	0.110000
111	-3057.789980	-25.492931	0.002006	0.000356	0.011996	0.003753	0.110000
112	-3057.790011	-25.512322	0.001863	0.000348	0.013903	0.003825	0.110000
113	-3057.790041	-25.531001	0.001859	0.000342	0.014403	0.003840	0.110000
114	-3057.790068	-25.547696	0.002040	0.000343	0.013961	0.003801	0.110000
115	-3057.790093	-25.563833	0.002075	0.000342	0.012266	0.003697	0.110000
116	-3057.790123	-25.582178	0.001894	0.000342	0.011971	0.003603	0.110000
117	-3057.790150	-25.599700	0.001665	0.000341	0.011980	0.003575	0.110000
118	-3057.790175	-25.615370	0.001563	0.000334	0.011654	0.003622	0.110000
119	-3057.790199	-25.629882	0.001735	0.000333	0.011598	0.003670	0.110000
120	-3057.790223	-25.645150	0.002000	0.000334	0.011671	0.003657	0.110000
121	-3057.790243	-25.657597	0.002073	0.000325	0.011434	0.003667	0.110000
122	-3057.790268	-25.673722	0.002132	0.000327	0.011896	0.003708	0.110000
123	-3057.790290	-25.687282	0.002110	0.000323	0.011815	0.003738	0.110000
124	-3057.790308	-25.698714	0.002046	0.000315	0.011943	0.003708	0.110000
125	-3057.790328	-25.711075	0.002085	0.000323	0.012426	0.003715	0.110000
126	-3057.790348	-25.723676	0.001978	0.000313	0.012311	0.003641	0.110000
127	-3057.790367	-25.735418	0.002033	0.000319	0.012598	0.003667	0.110000
128	-3057.790385	-25.746784	0.002043	0.000317	0.012443	0.003651	0.110000
129	-3057.790402	-25.757685	0.002084	0.000316	0.012411	0.003670	0.110000
130	-3057.790419	-25.768253	0.002102	0.000315	0.012404	0.003690	0.110000
131	-3057.790436	-25.778795	0.002053	0.000307	0.012177	0.003696	0.110000
132	-3057.790452	-25.788951	0.002069	0.000308	0.012354	0.003714	0.110000
133	-3057.790467	-25.798611	0.002083	0.000309	0.012545	0.003706	0.110000
134	-3057.790483	-25.808148	0.002056	0.000305	0.012447	0.003686	0.110000
135	-3057.790497	-25.817272	0.002048	0.000304	0.012426	0.003679	0.110000
136	-3057.790511	-25.826007	0.002036	0.000301	0.012370	0.003674	0.110000
137	-3057.790524	-25.834366	0.002040	0.000299	0.012346	0.003676	0.110000
138	-3057.790537	-25.842458	0.002025	0.000296	0.012291	0.003672	0.110000
139	-3057.790550	-25.850264	0.002023	0.000295	0.012275	0.003674	0.110000
140	-3057.790562	-25.857894	0.001975	0.000288	0.012154	0.003655	0.110000
141	-3057.790573	-25.865071	0.001994	0.000290	0.012184	0.003670	0.110000
142	-3057.790585	-25.872337	0.001850	0.000270	0.011850	0.003611	0.110000
143	-3057.790595	-25.878435	0.002068	0.000299	0.012367	0.003736	0.110000
144	-3057.790596	-25.879381	0.002039	0.000305	0.014562	0.002294	0.110000
145	-3057.790610	-25.887897	0.002077	0.000290	0.011604	0.004176	0.110001
146	-3057.790616	-25.892118	0.001041	0.000168	0.010511	0.004153	0.110000
147	-3057.790629	-25.900168	0.000463	0.000096	0.013783	0.004515	0.110000
148	-3057.790638	-25.905469	0.000550	0.000141	0.014345	0.004464	0.110000
149	-3057.790648	-25.911858	0.001075	0.000221	0.012578	0.004333	0.110000
150	-3057.790657	-25.917838	0.000345	0.000072	0.011808	0.003684	0.110000
151	-3057.790665	-25.922348	0.001018	0.000196	0.013970	0.004764	0.110000
152	-3057.790668	-25.924684	0.001064	0.000244	0.010096	0.004187	0.110000
153	-3057.790652	-25.914222	0.002739	0.000536	0.013443	0.004567	0.110000
154	-3057.790643	-25.909033	0.003437	0.000668	0.021655	0.005366	0.110000
155	-3057.790668	-25.924585	0.001606	0.000285	0.012690	0.002643	0.058713
156	-3057.790674	-25.928227	0.001064	0.000207	0.012916	0.004123	0.099606
157	-3057.790685	-25.935364	0.000333	0.000058	0.013443	0.004438	0.110000
158	-3057.790695	-25.941165	0.000336	0.000057	0.014871	0.004772	0.110000
159	-3057.790704	-25.947264	0.000449	0.000084	0.014576	0.004666	0.110000
160	-3057.790713	-25.952454	0.000548	0.000095	0.014548	0.004574	0.110000
161	-3057.790721	-25.957432	0.000462	0.000090	0.014377	0.004511	0.110000
162	-3057.790728	-25.962075	0.000277	0.000080	0.014145	0.004473	0.110000
163	-3057.790735	-25.966228	0.000329	0.000078	0.013781	0.004408	0.110000
164	-3057.790741	-25.970182	0.000370	0.000081	0.013524	0.004326	0.110000
165	-3057.790747	-25.974102	0.000388	0.000081	0.013465	0.004272	0.110000
166	-3057.790753	-25.977881	0.000390	0.000079	0.013175	0.004150	0.107140
167	-3057.790759	-25.981373	0.000386	0.000078	0.012653	0.003970	0.101952
168	-3057.790764	-25.984534	0.000385	0.000080	0.012502	0.003908	0.099421
169	-3057.790768	-25.987268	0.000374	0.000081	0.012501	0.003861	0.097860
170	-3057.790772	-25.989698	0.000304	0.000073	0.012543	0.003837	0.098865

171	-3057.790776	-25.992169	0.000278	0.000073	0.012008	0.003852	0.100024
172	-3057.790779	-25.994200	0.000256	0.000068	0.010114	0.003308	0.087548
173	-3057.790782	-25.996114	0.000243	0.000063	0.009462	0.003087	0.081093
174	-3057.790785	-25.997951	0.000249	0.000062	0.009552	0.003116	0.081024
175	-3057.790788	-25.999701	0.000259	0.000063	0.009632	0.003168	0.082564
176	-3057.790790	-26.001316	0.000241	0.000059	0.009138	0.003072	0.081272
177	-3057.790793	-26.002740	0.000217	0.000055	0.008360	0.002886	0.076287
178	-3057.790795	-26.004072	0.000209	0.000054	0.007914	0.002813	0.074509
179	-3057.790797	-26.005324	0.000196	0.000052	0.007451	0.002724	0.072521
180	-3057.790799	-26.006495	0.000183	0.000049	0.006941	0.002638	0.070522
181	-3057.790800	-26.007599	0.000170	0.000047	0.006855	0.002619	0.069767
182	-3057.790802	-26.008668	0.000170	0.000047	0.006845	0.002637	0.070473
183	-3057.790804	-26.009651	0.000155	0.000044	0.006491	0.002557	0.068819
184	-3057.790805	-26.010574	0.000152	0.000043	0.006083	0.002443	0.066019
185	-3057.790805	-26.010513	0.000155	0.000036	0.002872	0.000647	0.064974
186	-3057.790805	-26.010334	0.000720	0.000187	0.011792	0.004363	0.110000
187	-3057.790806	-26.011305	0.000665	0.000149	0.009301	0.001935	0.044376
188	-3057.790808	-26.012019	0.000444	0.000095	0.005856	0.001314	0.027429
189	-3057.790806	-26.011268	0.001068	0.000234	0.012044	0.004582	0.110000
190	-3057.790809	-26.013101	0.000967	0.000186	0.010241	0.003726	0.097054
191	-3057.790810	-26.013443	0.000882	0.000164	0.006669	0.001797	0.042075
192	-3057.790809	-26.012757	0.000683	0.000198	0.006318	0.002649	0.063485
193	-3057.790810	-26.013383	0.000746	0.000175	0.008468	0.002938	0.068891
194	-3057.790808	-26.012337	0.001060	0.000238	0.013532	0.002930	0.061142
195	-3057.790814	-26.016034	0.000082	0.000021	0.008471	0.001335	0.029892
196	-3057.790811	-26.014440	0.000755	0.000158	0.015617	0.004687	0.110001
197	-3057.790815	-26.016676	0.000321	0.000055	0.002835	0.001323	0.041516
198	-3057.790814	-26.016218	0.000513	0.000114	0.011787	0.003779	0.099137
199	-3057.790815	-26.016530	0.000158	0.000046	0.003282	0.001019	0.085115
200	-3057.790814	-26.016385	0.000278	0.000069	0.012260	0.004297	0.110001
201	-3057.790816	-26.017494	0.000077	0.000026	0.002360	0.000737	0.069778
202	-3057.790814	-26.016107	0.000473	0.000135	0.013127	0.004522	0.110000
203	-3057.790820	-26.019570	0.000125	0.000038	0.010629	0.003687	0.100060
204	-3057.790822	-26.020876	0.000195	0.000054	0.013356	0.004448	0.110000
205	-3057.790822	-26.020956	0.000071	0.000024	0.006782	0.002579	0.066427
206	-3057.790824	-26.022594	0.000141	0.000030	0.009910	0.003421	0.081750
207	-3057.790828	-26.024708	0.000105	0.000026	0.005815	0.002402	0.059821
208	-3057.790826	-26.023330	0.000098	0.000027	0.007892	0.002447	0.064757
209	-3057.790828	-26.025144	0.000066	0.000020	0.007658	0.003276	0.082346
210	-3057.790831	-26.026696	0.000084	0.000020	0.006547	0.002647	0.065399
211	-3057.790832	-26.027144	0.000068	0.000021	0.008270	0.002707	0.069827
212	-3057.790833	-26.027752	0.000080	0.000025	0.007773	0.002606	0.067310
213	-3057.790834	-26.028451	0.000077	0.000024	0.006466	0.002166	0.057541
214	-3057.790834	-26.028519	0.000057	0.000016	0.002119	0.000632	0.053511
215	-3057.790832	-26.027607	0.000628	0.000160	0.013989	0.004548	0.110000
216	-3057.790834	-26.028862	0.000544	0.000135	0.009543	0.002195	0.047447
217	-3057.790831	-26.026853	0.000911	0.000226	0.010099	0.003336	0.074266
218	-3057.790829	-26.025238	0.000530	0.000110	0.007166	0.002136	0.055671
219	-3057.790836	-26.029936	0.000104	0.000022	0.003706	0.001277	0.038746
220	-3057.790837	-26.030726	0.000297	0.000065	0.012483	0.004098	0.110000
221	-3057.790837	-26.030452	0.000059	0.000011	0.004270	0.001642	0.041767
222	-3057.790841	-26.033031	0.000065	0.000016	0.011070	0.004388	0.110000
223	-3057.790840	-26.032691	0.000069	0.000015	0.008471	0.002487	0.067425
224	-3057.790842	-26.033485	0.000059	0.000014	0.008052	0.002853	0.071781
225	-3057.790843	-26.034313	0.000066	0.000017	0.008080	0.002638	0.068950
226	-3057.790844	-26.034620	0.000055	0.000014	0.007238	0.002150	0.056057
227	-3057.790844	-26.035217	0.000074	0.000018	0.007854	0.002449	0.065725
228	-3057.790845	-26.035675	0.000053	0.000014	0.007086	0.002269	0.058081
229	-3057.790846	-26.036428	0.000070	0.000016	0.006621	0.002182	0.058256
230	-3057.790847	-26.036719	0.000056	0.000013	0.006787	0.002095	0.054992
231	-3057.790848	-26.037288	0.000070	0.000016	0.007135	0.002101	0.057252
232	-3057.790849	-26.037769	0.000055	0.000012	0.006463	0.002058	0.053702
233	-3057.790849	-26.038269	0.000071	0.000017	0.006700	0.001986	0.054401

234	-3057.790850	-26.038756	0.000054	0.000012	0.006101	0.001881	0.049422
235	-3057.790851	-26.039189	0.000069	0.000017	0.006379	0.001925	0.052899
236	-3057.790852	-26.039652	0.000055	0.000013	0.005840	0.001733	0.046033
237	-3057.790852	-26.040051	0.000062	0.000016	0.006026	0.001873	0.051211
238	-3057.790853	-26.040487	0.000059	0.000014	0.005759	0.001632	0.044031
239	-3057.790853	-26.040873	0.000055	0.000015	0.005616	0.001751	0.047547
240	-3057.790854	-26.041249	0.000064	0.000016	0.005757	0.001586	0.043382

```
************************************************************
*  MAXIMUM NUMBER OF ITERATIONS REACHED - STOPPING IRC RUN  *
************************************************************
```

```
---------------------------------------------------------------
                      IRC PATH SUMMARY
---------------------------------------------------------------
```

All gradients are in Eh/Bohr.

Step	E(Eh)	dE(kcal/mol)	max(|G|)	RMS(G)	SMW-Displ.
1	-3057.790854	-26.041249	0.000064	0.000016	-23.535210
2	-3057.790853	-26.040873	0.000055	0.000015	-23.491828
3	-3057.790853	-26.040487	0.000059	0.000014	-23.444281
4	-3057.790852	-26.040051	0.000062	0.000016	-23.400250
5	-3057.790852	-26.039652	0.000055	0.000013	-23.349039
6	-3057.790851	-26.039189	0.000069	0.000017	-23.303006
7	-3057.790850	-26.038756	0.000054	0.000012	-23.250107
8	-3057.790849	-26.038269	0.000071	0.000017	-23.200685
9	-3057.790849	-26.037769	0.000055	0.000012	-23.146284
10	-3057.790848	-26.037288	0.000070	0.000016	-23.092582
11	-3057.790847	-26.036719	0.000056	0.000013	-23.035330
12	-3057.790846	-26.036428	0.000070	0.000016	-22.980338
13	-3057.790845	-26.035675	0.000053	0.000014	-22.922082
14	-3057.790844	-26.035217	0.000074	0.000018	-22.864001
15	-3057.790844	-26.034620	0.000055	0.000014	-22.798276
16	-3057.790843	-26.034313	0.000066	0.000017	-22.742219
17	-3057.790842	-26.033485	0.000059	0.000014	-22.673269
18	-3057.790840	-26.032691	0.000069	0.000015	-22.601488
19	-3057.790841	-26.033031	0.000065	0.000016	-22.534063
20	-3057.790837	-26.030452	0.000059	0.000011	-22.424063
21	-3057.790837	-26.030726	0.000297	0.000065	-22.382296
22	-3057.790836	-26.029936	0.000104	0.000022	-22.272296
23	-3057.790829	-26.025238	0.000530	0.000110	-22.233550
24	-3057.790831	-26.026853	0.000911	0.000226	-22.177879
25	-3057.790834	-26.028862	0.000544	0.000135	-22.103613
26	-3057.790832	-26.027607	0.000628	0.000160	-22.056166
27	-3057.790834	-26.028519	0.000057	0.000016	-21.946166
28	-3057.790834	-26.028451	0.000077	0.000024	-21.892655
29	-3057.790833	-26.027752	0.000080	0.000025	-21.835114
30	-3057.790832	-26.027144	0.000068	0.000021	-21.767804
31	-3057.790831	-26.026696	0.000084	0.000020	-21.697977
32	-3057.790828	-26.025144	0.000066	0.000020	-21.632578
33	-3057.790826	-26.023330	0.000098	0.000027	-21.550232
34	-3057.790828	-26.024708	0.000105	0.000026	-21.485475
35	-3057.790824	-26.022594	0.000141	0.000030	-21.425654
36	-3057.790822	-26.020956	0.000071	0.000024	-21.343904
37	-3057.790822	-26.020876	0.000195	0.000054	-21.277477
38	-3057.790820	-26.019570	0.000125	0.000038	-21.167477
39	-3057.790814	-26.016107	0.000473	0.000135	-21.067418
40	-3057.790816	-26.017494	0.000077	0.000026	-20.957418
41	-3057.790814	-26.016385	0.000278	0.000069	-20.887639
42	-3057.790815	-26.016530	0.000158	0.000046	-20.777639
43	-3057.790814	-26.016218	0.000513	0.000114	-20.692524
44	-3057.790815	-26.016676	0.000321	0.000055	-20.593387

45	-3057.790811	-26.014440	0.000755	0.000158	-20.551872
46	-3057.790814	-26.016034	0.000082	0.000021	-20.441870
47	-3057.790808	-26.012337	0.001060	0.000238	-20.411978
48	-3057.790810	-26.013383	0.000746	0.000175	-20.350836
49	-3057.790809	-26.012757	0.000683	0.000198	-20.281945
50	-3057.790810	-26.013443	0.000882	0.000164	-20.218460
51	-3057.790809	-26.013101	0.000967	0.000186	-20.176386
52	-3057.790806	-26.011268	0.001068	0.000234	-20.079331
53	-3057.790808	-26.012019	0.000444	0.000095	-19.969331
54	-3057.790806	-26.011305	0.000665	0.000149	-19.941902
55	-3057.790805	-26.010334	0.000720	0.000187	-19.897526
56	-3057.790805	-26.010513	0.000155	0.000036	-19.787526
57	-3057.790805	-26.010574	0.000152	0.000043	-19.722552
58	-3057.790804	-26.009651	0.000155	0.000044	-19.656533
59	-3057.790802	-26.008668	0.000170	0.000047	-19.587714
60	-3057.790800	-26.007599	0.000170	0.000047	-19.517241
61	-3057.790799	-26.006495	0.000183	0.000049	-19.447475
62	-3057.790797	-26.005324	0.000196	0.000052	-19.376953
63	-3057.790795	-26.004072	0.000209	0.000054	-19.304431
64	-3057.790793	-26.002740	0.000217	0.000055	-19.229922
65	-3057.790790	-26.001316	0.000241	0.000059	-19.153635
66	-3057.790788	-25.999701	0.000259	0.000063	-19.072364
67	-3057.790785	-25.997951	0.000249	0.000062	-18.989799
68	-3057.790782	-25.996114	0.000243	0.000063	-18.908775
69	-3057.790779	-25.994200	0.000256	0.000068	-18.827683
70	-3057.790776	-25.992169	0.000278	0.000073	-18.740135
71	-3057.790772	-25.989698	0.000304	0.000073	-18.640111
72	-3057.790768	-25.987268	0.000374	0.000081	-18.541246
73	-3057.790764	-25.984534	0.000385	0.000080	-18.443386
74	-3057.790759	-25.981373	0.000386	0.000078	-18.343965
75	-3057.790753	-25.977881	0.000390	0.000079	-18.242013
76	-3057.790747	-25.974102	0.000388	0.000081	-18.134873
77	-3057.790741	-25.970182	0.000370	0.000081	-18.024873
78	-3057.790735	-25.966228	0.000329	0.000078	-17.914873
79	-3057.790728	-25.962075	0.000277	0.000080	-17.804873
80	-3057.790721	-25.957432	0.000462	0.000090	-17.694873
81	-3057.790713	-25.952454	0.000548	0.000095	-17.584873
82	-3057.790704	-25.947264	0.000449	0.000084	-17.474873
83	-3057.790695	-25.941165	0.000336	0.000057	-17.364873
84	-3057.790685	-25.935364	0.000333	0.000058	-17.254873
85	-3057.790674	-25.928227	0.001064	0.000207	-17.144872
86	-3057.790668	-25.924585	0.001606	0.000285	-17.045267
87	-3057.790643	-25.909033	0.003437	0.000668	-16.986553
88	-3057.790652	-25.914222	0.002739	0.000536	-16.876553
89	-3057.790668	-25.924684	0.001064	0.000244	-16.766553
90	-3057.790665	-25.922348	0.001018	0.000196	-16.656553
91	-3057.790657	-25.917838	0.000345	0.000072	-16.546553
92	-3057.790648	-25.911858	0.001075	0.000221	-16.436553
93	-3057.790638	-25.905469	0.000550	0.000141	-16.326553
94	-3057.790629	-25.900168	0.000463	0.000096	-16.216552
95	-3057.790616	-25.892118	0.001041	0.000168	-16.106552
96	-3057.790610	-25.887897	0.002077	0.000290	-15.996552
97	-3057.790596	-25.879381	0.002039	0.000305	-15.886551
98	-3057.790595	-25.878435	0.002068	0.000299	-15.776551
99	-3057.790585	-25.872337	0.001850	0.000270	-15.666551
100	-3057.790573	-25.865071	0.001994	0.000290	-15.556551
101	-3057.790562	-25.857894	0.001975	0.000288	-15.446551
102	-3057.790550	-25.850264	0.002023	0.000295	-15.336551
103	-3057.790537	-25.842458	0.002025	0.000296	-15.226551
104	-3057.790524	-25.834366	0.002040	0.000299	-15.116551
105	-3057.790511	-25.826007	0.002036	0.000301	-15.006551
106	-3057.790497	-25.817272	0.002048	0.000304	-14.896551
107	-3057.790483	-25.808148	0.002056	0.000305	-14.786551

108	-3057.790467	-25.798611	0.002083	0.000309	-14.676551
109	-3057.790452	-25.788951	0.002069	0.000308	-14.566551
110	-3057.790436	-25.778795	0.002053	0.000307	-14.456551
111	-3057.790419	-25.768253	0.002102	0.000315	-14.346550
112	-3057.790402	-25.757685	0.002084	0.000316	-14.236550
113	-3057.790385	-25.746784	0.002043	0.000317	-14.126550
114	-3057.790367	-25.735418	0.002033	0.000319	-14.016550
115	-3057.790348	-25.723676	0.001978	0.000313	-13.906550
116	-3057.790328	-25.711075	0.002085	0.000323	-13.796550
117	-3057.790308	-25.698714	0.002046	0.000315	-13.686550
118	-3057.790290	-25.687282	0.002110	0.000323	-13.576550
119	-3057.790268	-25.673722	0.002132	0.000327	-13.466550
120	-3057.790243	-25.657597	0.002073	0.000325	-13.356550
121	-3057.790223	-25.645150	0.002000	0.000334	-13.246550
122	-3057.790199	-25.629882	0.001735	0.000333	-13.136550
123	-3057.790175	-25.615370	0.001563	0.000334	-13.026549
124	-3057.790150	-25.599700	0.001665	0.000341	-12.916549
125	-3057.790123	-25.582178	0.001894	0.000342	-12.806549
126	-3057.790093	-25.563833	0.002075	0.000342	-12.696549
127	-3057.790068	-25.547696	0.002040	0.000343	-12.586549
128	-3057.790041	-25.531001	0.001859	0.000342	-12.476549
129	-3057.790011	-25.512322	0.001863	0.000348	-12.366549
130	-3057.789980	-25.492931	0.002006	0.000356	-12.256549
131	-3057.789948	-25.472863	0.002090	0.000357	-12.146549
132	-3057.789913	-25.450581	0.001959	0.000361	-12.036549
133	-3057.789881	-25.430439	0.001786	0.000382	-11.926549
134	-3057.789847	-25.409263	0.001841	0.000373	-11.816549
135	-3057.789811	-25.386681	0.001892	0.000379	-11.706548
136	-3057.789772	-25.362170	0.001976	0.000405	-11.596548
137	-3057.789736	-25.339373	0.002016	0.000342	-11.486547
138	-3057.789698	-25.315753	0.002081	0.000349	-11.376547
139	-3057.789647	-25.283712	0.002176	0.000468	-11.266547
140	-3057.789609	-25.259816	0.003393	0.000568	-11.156545
141	-3057.789595	-25.251386	0.003277	0.000590	-11.046545
142	-3057.789556	-25.226570	0.002715	0.000538	-10.936545
143	-3057.789513	-25.199396	0.002549	0.000525	-10.826545
144	-3057.789466	-25.170393	0.002729	0.000543	-10.716545
145	-3057.789418	-25.139828	0.003138	0.000583	-10.606545
146	-3057.789367	-25.108226	0.003577	0.000632	-10.496545
147	-3057.789317	-25.076543	0.003789	0.000657	-10.386544
148	-3057.789265	-25.044253	0.003918	0.000677	-10.276544
149	-3057.789213	-25.011381	0.003909	0.000683	-10.166544
150	-3057.789159	-24.977632	0.003813	0.000679	-10.056544
151	-3057.789103	-24.942405	0.003785	0.000680	-9.946544
152	-3057.789044	-24.905609	0.003869	0.000693	-9.836544
153	-3057.788984	-24.867476	0.004027	0.000713	-9.726544
154	-3057.788921	-24.828255	0.004191	0.000735	-9.616543
155	-3057.788857	-24.788117	0.004318	0.000752	-9.506543
156	-3057.788792	-24.746983	0.004399	0.000766	-9.396543
157	-3057.788724	-24.704757	0.004449	0.000776	-9.286543
158	-3057.788655	-24.661339	0.004480	0.000785	-9.176542
159	-3057.788584	-24.616663	0.004500	0.000793	-9.066542
160	-3057.788511	-24.570647	0.004511	0.000801	-8.956542
161	-3057.788435	-24.523231	0.004523	0.000809	-8.846542
162	-3057.788357	-24.474344	0.004541	0.000818	-8.736542
163	-3057.788277	-24.424085	0.004529	0.000824	-8.626541
164	-3057.788194	-24.372129	0.004535	0.000833	-8.516541
165	-3057.788109	-24.318502	0.004546	0.000843	-8.406541
166	-3057.788020	-24.263068	0.004590	0.000851	-8.296540
167	-3057.787929	-24.205684	0.004717	0.000860	-8.186540
168	-3057.787834	-24.146338	0.004848	0.000870	-8.076540
169	-3057.787737	-24.084981	0.004984	0.000880	-7.966540
170	-3057.787635	-24.021505	0.005125	0.000891	-7.856539

171	-3057.787531	-23.955829	0.005271	0.000902	-7.746539
172	-3057.787422	-23.887838	0.005423	0.000914	-7.636539
173	-3057.787310	-23.817421	0.005579	0.000928	-7.526538
174	-3057.787194	-23.744483	0.005743	0.000943	-7.416538
175	-3057.787074	-23.669108	0.005916	0.000955	-7.306537
176	-3057.786949	-23.591016	0.006092	0.000962	-7.196537
177	-3057.786820	-23.509532	0.006266	0.000978	-7.086536
178	-3057.786684	-23.424522	0.006439	0.001012	-6.976536
179	-3057.786545	-23.337331	0.006650	0.001050	-6.866536
180	-3057.786409	-23.251791	0.006934	0.000980	-6.756535
181	-3057.786249	-23.151726	0.007203	0.000910	-6.646534
182	-3057.786077	-23.043608	0.007401	0.001170	-6.536534
183	-3057.785933	-22.953254	0.007569	0.000921	-6.426532
184	-3057.785745	-22.835566	0.007767	0.001033	-6.316532
185	-3057.785558	-22.718095	0.008067	0.001257	-6.206531
186	-3057.785391	-22.613242	0.008418	0.001034	-6.096530
187	-3057.785187	-22.485286	0.008736	0.001131	-5.986528
188	-3057.784980	-22.355232	0.008996	0.001314	-5.876527
189	-3057.784798	-22.240697	0.009245	0.001223	-5.766526
190	-3057.784592	-22.112004	0.009559	0.001250	-5.656525
191	-3057.784368	-21.971126	0.009846	0.001401	-5.546524
192	-3057.784166	-21.844664	0.010124	0.001359	-5.436524
193	-3057.783938	-21.701097	0.010413	0.001430	-5.326523
194	-3057.783708	-21.557012	0.010763	0.001413	-5.216522
195	-3057.783456	-21.398885	0.011132	0.001547	-5.106522
196	-3057.783215	-21.247644	0.011445	0.001531	-4.996521
197	-3057.782952	-21.082686	0.011765	0.001609	-4.886519
198	-3057.782688	-20.916823	0.012085	0.001629	-4.776517
199	-3057.782408	-20.741074	0.012418	0.001688	-4.666517
200	-3057.782121	-20.560912	0.012753	0.001723	-4.556516
201	-3057.781820	-20.371983	0.013097	0.001779	-4.446516
202	-3057.781509	-20.176996	0.013446	0.001822	-4.336514
203	-3057.781184	-19.973403	0.013803	0.001876	-4.226513
204	-3057.780848	-19.762443	0.014164	0.001923	-4.116511
205	-3057.780498	-19.542504	0.014534	0.001981	-4.006511
206	-3057.780134	-19.314328	0.014909	0.002034	-3.896509
207	-3057.779756	-19.076825	0.015291	0.002091	-3.786509
208	-3057.779362	-18.829908	0.015671	0.002148	-3.676507
209	-3057.778953	-18.573076	0.016053	0.002208	-3.566507
210	-3057.778527	-18.306120	0.016445	0.002269	-3.456507
211	-3057.778085	-18.028422	0.016844	0.002334	-3.346506
212	-3057.777624	-17.739554	0.017250	0.002400	-3.236506
213	-3057.777145	-17.438959	0.017664	0.002466	-3.126504
214	-3057.776647	-17.126122	0.018085	0.002534	-3.016504
215	-3057.776128	-16.800606	0.018506	0.002606	-2.906502
216	-3057.775590	-16.463114	0.018928	0.002684	-2.796499
217	-3057.775027	-16.109344	0.019330	0.002729	-2.686496
218	-3057.774432	-15.736152	0.019742	0.002782	-2.576496
219	-3057.773807	-15.343781	0.020181	0.002858	-2.466493
220	-3057.773154	-14.934160	0.020634	0.002944	-2.356491
221	-3057.772477	-14.509760	0.021091	0.003036	-2.246491
222	-3057.771777	-14.070233	0.021549	0.003132	-2.136488
223	-3057.771052	-13.614990	0.022004	0.003232	-2.026487
224	-3057.770299	-13.142906	0.022453	0.003337	-1.916483
225	-3057.769520	-12.654112	0.022893	0.003446	-1.806482
226	-3057.768714	-12.148227	0.023315	0.003561	-1.696479
227	-3057.767875	-11.621808	0.023711	0.003681	-1.586477
228	-3057.767004	-11.075432	0.024071	0.003810	-1.476477
229	-3057.766097	-10.505927	0.024383	0.003950	-1.366476
230	-3057.765146	-9.909434	0.024631	0.004103	-1.256472
231	-3057.764148	-9.282740	0.024797	0.004273	-1.146469
232	-3057.763088	-8.617763	0.024864	0.004462	-1.036467
233	-3057.761957	-7.908065	0.024807	0.004672	-0.926466

234	-3057.760736	-7.141614	0.024584	0.004902	-0.816464
235	-3057.759398	-6.302204	0.024950	0.005125	-0.706461
236	-3057.757895	-5.359306	0.024838	0.005265	-0.596458
237	-3057.756078	-4.219249	0.022757	0.005182	-0.486455
238	-3057.753892	-2.847236	0.019172	0.004728	-0.376454
239	-3057.751687	-1.463319	0.014715	0.003721	-0.266454
240	-3057.750078	-0.453681	0.008318	0.002127	-0.156449
241	-3057.749405	-0.031642	0.002324	0.000551	-0.046448
242	-3057.749355	0.000000	0.000027	0.000008	0.000000 <= TS
243	-3057.749399	-0.027604	0.002308	0.000510	0.046448
244	-3057.749795	-0.276493	0.007462	0.001481	0.156450
245	-3057.750405	-0.659232	0.011629	0.002160	0.266454
246	-3057.751090	-1.089100	0.014372	0.002583	0.376454
247	-3057.751807	-1.538621	0.016021	0.002848	0.486454
248	-3057.752543	-2.000746	0.017460	0.003027	0.596456
249	-3057.753300	-2.475519	0.018814	0.003158	0.706459
250	-3057.754068	-2.957628	0.019943	0.003262	0.816460
251	-3057.754848	-3.447422	0.020919	0.003350	0.926461
252	-3057.755641	-3.944972	0.021782	0.003427	1.036464
253	-3057.756443	-4.448063	0.022559	0.003496	1.146466
254	-3057.757255	-4.957290	0.023264	0.003559	1.256466
255	-3057.758075	-5.471992	0.023906	0.003616	1.366468
256	-3057.758901	-5.990680	0.024488	0.003668	1.476469
257	-3057.759737	-6.514734	0.025012	0.003714	1.586471
258	-3057.760580	-7.043880	0.025475	0.003753	1.696472
259	-3057.761426	-7.574980	0.025873	0.003786	1.806475
260	-3057.762276	-8.108550	0.026203	0.003811	1.916478
261	-3057.763129	-8.643628	0.026462	0.003828	2.026480
262	-3057.763983	-9.179284	0.026646	0.003837	2.136483
263	-3057.764836	-9.714456	0.026754	0.003837	2.246484
264	-3057.765686	-10.248135	0.026783	0.003827	2.356486
265	-3057.766525	-10.774657	0.026729	0.003808	2.466487
266	-3057.767373	-11.306416	0.026600	0.003778	2.576488
267	-3057.768191	-11.819931	0.026381	0.003737	2.686490
268	-3057.769006	-12.331700	0.026087	0.003686	2.796492
269	-3057.769806	-12.833416	0.025715	0.003626	2.906493
270	-3057.770584	-13.321675	0.025269	0.003559	3.016496
271	-3057.771337	-13.794155	0.024739	0.003483	3.126496
272	-3057.772068	-14.252724	0.024120	0.003394	3.236499
273	-3057.772778	-14.698114	0.023423	0.003296	3.346500
274	-3057.773463	-15.128469	0.022652	0.003190	3.456501
275	-3057.774123	-15.542458	0.021808	0.003075	3.566504
276	-3057.774755	-15.938823	0.020893	0.002953	3.676507
277	-3057.775357	-16.316907	0.019910	0.002823	3.786508
278	-3057.775930	-16.676550	0.018863	0.002687	3.896511
279	-3057.776473	-17.017202	0.017757	0.002544	4.006513
280	-3057.776984	-17.337686	0.016598	0.002396	4.116514
281	-3057.777463	-17.638172	0.015393	0.002243	4.226514
282	-3057.777910	-17.918692	0.014152	0.002089	4.336516
283	-3057.778326	-18.179626	0.012885	0.001930	4.446516
284	-3057.778708	-18.419377	0.011612	0.001780	4.556516
285	-3057.779064	-18.642946	0.010333	0.001614	4.666518
286	-3057.779380	-18.841139	0.009104	0.001499	4.776520
287	-3057.779691	-19.036341	0.007854	0.001294	4.886521
288	-3057.779951	-19.199354	0.006836	0.001372	4.996522
289	-3057.780227	-19.372495	0.006346	0.001033	5.106526
290	-3057.780458	-19.517758	0.005788	0.000987	5.216526
291	-3057.780681	-19.657858	0.005303	0.000829	5.326527
292	-3057.780896	-19.792761	0.004834	0.000715	5.436529
293	-3057.781096	-19.917963	0.004401	0.000662	5.546529
294	-3057.781275	-20.030414	0.004055	0.000687	5.656531
295	-3057.781445	-20.137220	0.003746	0.000699	5.766531
296	-3057.781608	-20.239523	0.003517	0.000736	5.876532

297	-3057.781767	-20.339291	0.004044	0.000742	5.986533
298	-3057.781924	-20.437707	0.004055	0.000703	6.096534
299	-3057.782077	-20.533629	0.003962	0.000672	6.206534
300	-3057.782228	-20.628544	0.003934	0.000661	6.316534
301	-3057.782376	-20.721248	0.003990	0.000674	6.426535
302	-3057.782518	-20.810140	0.003950	0.000691	6.536536
303	-3057.782660	-20.899100	0.003785	0.000678	6.646537
304	-3057.782800	-20.987109	0.003786	0.000671	6.756538
305	-3057.782937	-21.073427	0.003954	0.000679	6.866538
306	-3057.783072	-21.158227	0.004073	0.000679	6.976539
307	-3057.783205	-21.241205	0.004017	0.000668	7.086540
308	-3057.783334	-21.322369	0.003996	0.000667	7.196540
309	-3057.783461	-21.401783	0.003962	0.000665	7.306540
310	-3057.783585	-21.479711	0.003938	0.000661	7.416541
311	-3057.783706	-21.555970	0.003932	0.000657	7.526542
312	-3057.783825	-21.630612	0.003920	0.000654	7.636542
313	-3057.783942	-21.703739	0.003897	0.000649	7.746542
314	-3057.784056	-21.775582	0.003873	0.000645	7.856543
315	-3057.784168	-21.845581	0.003858	0.000642	7.966543
316	-3057.784277	-21.914020	0.003839	0.000639	8.076544
317	-3057.784384	-21.980904	0.003818	0.000635	8.186544
318	-3057.784487	-22.046153	0.003796	0.000631	8.296545
319	-3057.784589	-22.109744	0.003771	0.000626	8.406545
320	-3057.784687	-22.171632	0.003745	0.000622	8.516546
321	-3057.784783	-22.231742	0.003717	0.000618	8.626546
322	-3057.784876	-22.290053	0.003685	0.000613	8.736546
323	-3057.784966	-22.346459	0.003650	0.000607	8.846546
324	-3057.785053	-22.400924	0.003609	0.000601	8.956546
325	-3057.785136	-22.453317	0.003567	0.000595	9.066547
326	-3057.785216	-22.503580	0.003517	0.000588	9.176547
327	-3057.785293	-22.551674	0.003463	0.000581	9.286547
328	-3057.785366	-22.597497	0.003404	0.000573	9.396548
329	-3057.785436	-22.641087	0.003337	0.000564	9.506548
330	-3057.785501	-22.682364	0.003270	0.000555	9.616549
331	-3057.785564	-22.721399	0.003190	0.000545	9.726549
332	-3057.785622	-22.758083	0.003111	0.000534	9.836549
333	-3057.785677	-22.792636	0.003014	0.000520	9.946549
334	-3057.785728	-22.824855	0.002925	0.000507	10.056550
335	-3057.785777	-22.855427	0.002699	0.000480	10.166550
336	-3057.785824	-22.884993	0.002123	0.000430	10.276550
337	-3057.785870	-22.913579	0.001395	0.000368	10.386550
338	-3057.785907	-22.936919	0.001667	0.000388	10.496551
339	-3057.785951	-22.964240	0.001154	0.000326	10.606551
340	-3057.785963	-22.972015	0.003316	0.000587	10.716551
341	-3057.785973	-22.978303	0.003838	0.000649	10.826551
342	-3057.786021	-23.008703	0.001942	0.000446	10.936552
343	-3057.786074	-23.041472	0.000994	0.000181	11.046553
344	-3057.786097	-23.055941	0.001620	0.000368	11.156553
345	-3057.786140	-23.083121	0.000879	0.000229	11.266554
346	-3057.786178	-23.107220	0.000868	0.000171	11.376554
347	-3057.786206	-23.124766	0.001054	0.000321	11.486555
348	-3057.786239	-23.145223	0.000870	0.000285	11.596555
349	-3057.786268	-23.163336	0.001083	0.000269	11.706555
350	-3057.786295	-23.180611	0.001582	0.000298	11.816555
351	-3057.786324	-23.198559	0.001492	0.000282	11.926556
352	-3057.786350	-23.215096	0.001369	0.000284	12.036556
353	-3057.786377	-23.231629	0.001271	0.000282	12.146556
354	-3057.786403	-23.248090	0.001323	0.000280	12.256556
355	-3057.786428	-23.263677	0.001444	0.000278	12.366556
356	-3057.786452	-23.278628	0.001540	0.000285	12.476556
357	-3057.786477	-23.294401	0.001542	0.000284	12.586556
358	-3057.786501	-23.309525	0.001544	0.000285	12.696556
359	-3057.786525	-23.324627	0.001547	0.000282	12.806557

360	-3057.786548	-23.339113	0.001549	0.000283	12.916557
361	-3057.786572	-23.354052	0.001533	0.000278	13.026557
362	-3057.786595	-23.368481	0.001519	0.000279	13.136557
363	-3057.786617	-23.382428	0.001531	0.000282	13.246557
364	-3057.786640	-23.396961	0.001551	0.000280	13.356557
365	-3057.786663	-23.411219	0.001567	0.000283	13.466558
366	-3057.786685	-23.424833	0.001575	0.000285	13.576558
367	-3057.786707	-23.439148	0.001587	0.000284	13.686558
368	-3057.786730	-23.453251	0.001580	0.000283	13.796558
369	-3057.786751	-23.466731	0.001574	0.000284	13.906558
370	-3057.786774	-23.480842	0.001584	0.000283	14.016558
371	-3057.786796	-23.494762	0.001563	0.000281	14.126558
372	-3057.786817	-23.508113	0.001581	0.000285	14.236558
373	-3057.786840	-23.522145	0.001585	0.000283	14.346558
374	-3057.786861	-23.535821	0.001583	0.000283	14.456558
375	-3057.786883	-23.549306	0.001592	0.000286	14.566559
376	-3057.786905	-23.563244	0.001598	0.000285	14.676559
377	-3057.786927	-23.576677	0.001553	0.000280	14.786559
378	-3057.786948	-23.590299	0.001639	0.000291	14.896559
379	-3057.786955	-23.594718	0.001336	0.000247	15.006559
380	-3057.786977	-23.608375	0.001082	0.000225	15.116561
381	-3057.787005	-23.625834	0.000811	0.000141	15.226561
382	-3057.787030	-23.641641	0.000668	0.000129	15.336562
383	-3057.787050	-23.653996	0.000698	0.000174	15.446562
384	-3057.787077	-23.670943	0.000786	0.000216	15.556562
385	-3057.787101	-23.685894	0.000867	0.000236	15.666562
386	-3057.787124	-23.700391	0.001216	0.000268	15.776563
387	-3057.787140	-23.710391	0.001434	0.000295	15.886563
388	-3057.787169	-23.728877	0.001366	0.000287	15.996563
389	-3057.787193	-23.743722	0.001250	0.000260	16.106563
390	-3057.787215	-23.757546	0.001248	0.000253	16.216563
391	-3057.787237	-23.771733	0.001359	0.000265	16.326563
392	-3057.787259	-23.785114	0.001533	0.000286	16.436563
393	-3057.787280	-23.798574	0.001572	0.000293	16.546563
394	-3057.787307	-23.815279	0.001479	0.000281	16.656564
395	-3057.787330	-23.829606	0.001356	0.000266	16.766564
396	-3057.787352	-23.843390	0.001184	0.000247	16.876564
397	-3057.787370	-23.855064	0.001152	0.000248	16.986564
398	-3057.787398	-23.872224	0.001134	0.000253	17.096564
399	-3057.787420	-23.886321	0.001143	0.000254	17.206564
400	-3057.787442	-23.900053	0.001233	0.000261	17.316564
401	-3057.787464	-23.914096	0.001307	0.000267	17.426564
402	-3057.787483	-23.926161	0.001366	0.000270	17.536564
403	-3057.787501	-23.936890	0.001510	0.000283	17.646564
404	-3057.787531	-23.955823	0.001593	0.000291	17.756565
405	-3057.787553	-23.969949	0.001215	0.000228	17.866565
406	-3057.787578	-23.985345	0.001226	0.000211	17.976565
407	-3057.787601	-23.999972	0.001485	0.000237	18.086565
408	-3057.787623	-24.013856	0.001804	0.000286	18.196565
409	-3057.787636	-24.022134	0.001713	0.000283	18.306566
410	-3057.787667	-24.041084	0.001594	0.000274	18.416566
411	-3057.787689	-24.055229	0.001454	0.000271	18.526566
412	-3057.787710	-24.068488	0.001439	0.000275	18.636566
413	-3057.787733	-24.082613	0.001446	0.000276	18.746566
414	-3057.787751	-24.093873	0.001427	0.000270	18.856566
415	-3057.787775	-24.109017	0.001477	0.000274	18.966566
416	-3057.787800	-24.124487	0.001711	0.000294	19.076567
417	-3057.787820	-24.137235	0.001841	0.000299	19.186567
418	-3057.787843	-24.151855	0.001890	0.000296	19.296567
419	-3057.787866	-24.165981	0.001908	0.000293	19.406567
420	-3057.787888	-24.180146	0.001809	0.000279	19.516567
421	-3057.787911	-24.194528	0.001693	0.000268	19.626567
422	-3057.787932	-24.207794	0.001466	0.000253	19.736567

423	-3057.787954	-24.221687	0.001046	0.000229	19.846567
424	-3057.787979	-24.237214	0.000878	0.000217	19.956567
425	-3057.787998	-24.249014	0.001010	0.000221	20.066567
426	-3057.788020	-24.262865	0.000980	0.000220	20.176568
427	-3057.788042	-24.276663	0.000888	0.000227	20.286568
428	-3057.788064	-24.290514	0.000946	0.000234	20.396568
429	-3057.788086	-24.304137	0.000984	0.000231	20.506568
430	-3057.788108	-24.317951	0.000858	0.000221	20.616568
431	-3057.788131	-24.332384	0.000772	0.000210	20.726568
432	-3057.788152	-24.345373	0.000857	0.000209	20.836568
433	-3057.788174	-24.359748	0.000918	0.000216	20.946568
434	-3057.788194	-24.371872	0.000877	0.000220	21.056569
435	-3057.788219	-24.387701	0.000839	0.000218	21.166569
436	-3057.788237	-24.399186	0.000838	0.000227	21.276569
437	-3057.788259	-24.412878	0.000845	0.000222	21.386569
438	-3057.788281	-24.426487	0.000865	0.000218	21.496569
439	-3057.788302	-24.440042	0.000909	0.000217	21.606569
440	-3057.788324	-24.453831	0.000890	0.000216	21.716569
441	-3057.788346	-24.467640	0.000845	0.000216	21.826569
442	-3057.788368	-24.481384	0.000804	0.000216	21.936570
443	-3057.788390	-24.495187	0.000744	0.000217	22.046570
444	-3057.788412	-24.508782	0.000796	0.000212	22.156570
445	-3057.788434	-24.522747	0.000838	0.000211	22.266570
446	-3057.788456	-24.536602	0.000860	0.000213	22.376570
447	-3057.788478	-24.550456	0.000873	0.000217	22.486570
448	-3057.788500	-24.564261	0.000887	0.000218	22.596570
449	-3057.788522	-24.578020	0.000931	0.000219	22.706570
450	-3057.788545	-24.592328	0.000965	0.000220	22.816570
451	-3057.788567	-24.606049	0.001010	0.000221	22.926570
452	-3057.788589	-24.619858	0.001047	0.000222	23.036571
453	-3057.788611	-24.633589	0.001093	0.000225	23.146571
454	-3057.788633	-24.647409	0.001115	0.000226	23.256571
455	-3057.788678	-24.676017	0.001144	0.000230	23.366571
456	-3057.788660	-24.664274	0.000834	0.000208	23.476571
457	-3057.788681	-24.677670	0.000679	0.000167	23.586572
458	-3057.788706	-24.693564	0.000696	0.000213	23.696572
459	-3057.788725	-24.705373	0.001157	0.000273	23.806572
460	-3057.788749	-24.720116	0.001044	0.000256	23.916572
461	-3057.788770	-24.733611	0.000973	0.000250	24.026573
462	-3057.788792	-24.747240	0.000989	0.000244	24.136573
463	-3057.788814	-24.760815	0.000965	0.000241	24.246573
464	-3057.788835	-24.774308	0.000937	0.000240	24.356573
465	-3057.788857	-24.787798	0.000942	0.000239	24.466573
466	-3057.788878	-24.801184	0.000978	0.000238	24.576573
467	-3057.788899	-24.814429	0.000999	0.000240	24.686574
468	-3057.788920	-24.827513	0.000991	0.000244	24.796574
469	-3057.788940	-24.840455	0.000978	0.000246	24.906574
470	-3057.788961	-24.853207	0.000980	0.000246	25.016574
471	-3057.788981	-24.865744	0.000996	0.000245	25.126574
472	-3057.789000	-24.878077	0.001006	0.000245	25.236574
473	-3057.789020	-24.890207	0.001005	0.000244	25.346574
474	-3057.789039	-24.902249	0.001003	0.000244	25.456574
475	-3057.789058	-24.914198	0.001006	0.000244	25.566574
476	-3057.789077	-24.926031	0.001006	0.000243	25.676575
477	-3057.789095	-24.937612	0.001008	0.000244	25.786575
478	-3057.789114	-24.949120	0.001006	0.000244	25.896575
479	-3057.789132	-24.960454	0.000994	0.000239	26.006575
480	-3057.789149	-24.971418	0.001006	0.000245	26.116575
481	-3057.789167	-24.982518	0.000984	0.000235	26.226575
482	-3057.789184	-24.993023	0.001000	0.000248	26.336575
483	-3057.789201	-25.004194	0.000881	0.000204	26.446575

SUGGESTED CITATIONS FOR THIS RUN

Below you find a list of papers that are relevant to this ORCA run
We neither can nor want to force you to cite these papers, but we appreciate if you do
You receive ORCA, which is the product of decades of hard work by many enthusiastic individuals, for free
The only thing we kindly ask in return is that you cite our papers,
We deeply appreciate it, if you show your appreciation for ORCA by not just citing the generic ORCA reference.

Please note that relegating all ORCA citations to the supporting information does *not* help us.
SI sections are not indexed - citations you put there will not count into any citation statistics
But we need these citations in order to attract the funding resources that allow us to do what we are doing

Therefore, if you are a happy ORCA user, please consider citing a few of the papers listed below in the main body of your paper

In addition to the list printed below, the program has created the file H-IRC.bibtex that contains the list in bibtex format
You can import this file easily into all common literature databanks and citation aid programs

It goes without saying that in many instances, there are alternative algorithms to achieve similar
results as the ones you have gotten from ORCA. It is, of course, also the case that in some instances
ORCA just re-implements algorithms worked out by others. We are fully aware of that and we are also
fully appreciative of our colleagues work. Hence this citation list should not be read as indicating
that the listed papers, which are focused on our own work, are the only ones worth citing. It simply
meant to make it easier for users to cite ORCA specific papers. It is not a substitute for doing your
own literature research and citing the relevant literature in a scientifically appropriate manner.

List of essential papers. We consider these as the minimum necessary citations

1. Neese, F.
   Software update: the ORCA program system, version 6.0
   WIRES Comput. Molec. Sci. 2025 15(1), e70019
   doi.org/10.1002/wcms.70019

List of papers to cite with high priority. The work reported in these papers was absolutely necessary for this run to complete.
Our perspective: the developers of density functionals and basis sets usually get cited in chemistry papers
Good! But without the algorithms to do something with them, the functionals or basis sets would not do anything.
Hence, in our opinion, the algorithm design and method developments papers are equally worthy of getting cited

1. Page, M.; Doubleday, C.; McIver, J.
   Following steepest descent reaction paths. The use of higher energy derivatives with abinitio electronic structure methods
   J. Chem. Phys. 1990 93(8), 5634-5642
   doi.org/10.1063/1.459634
2. Neese, F.

An improvement of the resolution of the identity approximation for the formation of the Coulomb matrix
J. Comp. Chem. 2003 24(14), 1740-1747
doi.org/10.1002/jcc.10318
3. Hratchian, H.; Schlegel, B.
Using Hessian Updating To Increase the Efficiency of a Hessian Based Predictor-Corrector Reaction Path Following Method
J. Chem. Theory Comput. 2005 1(1), 61-69
doi.org/10.1021/ct0499783
4. Neese, F.; Wennmohs, F.; Hansen, A.; Becker, U.
Efficient, approximate and parallel Hartree-Fock and hybrid DFT calculations. A 'chain-of-spheres' algorithm for the Hartree-Fock exchange
Chem. Phys. 2009 356(1-3), 98-109
doi.org/10.1016/j.chemphys.2008.10.036
5. Grimme, S.; Antony, J.; Ehrlich, S.; Krieg, H.
A consistent and accurate ab initio parametrization of density functional dispersion correction (DFT-D) for the 94 elements H-Pu
J. Chem. Phys. 2010 132 , 154104
doi.org/10.1063/1.3382344
6. Grimme, S.; Ehrlich, S.; Goerigk, L.
Effect of the damping function in dispersion corrected density functional theory
J. Comput. Chem. 2011 32(7), 1456-1465
doi.org/10.1002/jcc.21759
7. Garcia-Rates, M.; Neese, F.
Effect of the Solute Cavity on the Solvation Energy and its Derivatives within the Framework of the Gaussian Charge Scheme
J. Comput. Chem. 2020 41 , 922-939
doi.org/10.1002/jcc.26139
8. Helmich-Paris, B.; de Souza, B.; Neese, F.; Izsák, R.
An improved chain of spheres for exchange algorithm
J. Chem. Phys. 2021 155(10), 104109
doi.org/10.1063/5.0058766
9. Neese, F.
The SHARK Integral Generation and Digestion System
J. Comp. Chem. 2022 44(3), 381
doi.org/10.1002/jcc.26942

List of suggested additional citations. These are papers that are important in the 'surrounding' of
of this run, or papers that preceded the highly important papers. If you like your results we are grateful for a citation.

1. Izsak, R.; Neese, F.
An overlap fitted chain of spheres exchange method
J. Chem. Phys. 2011 135 , 144105
doi.org/10.1063/1.3646921
2. Izsak, R.; Hansen, A.; Neese, F.
The resolution of identity and chain of spheres approximations for the LPNO-CCSD singles Fock term
Molec. Phys. 2012 110 , 2413-2417
doi.org/10.1080/00268976.2012.687466
3. Neese, F.
The ORCA program system
WIRES Comput. Molec. Sci. 2012 2(1), 73-78
doi.org/10.1002/wcms.81
4. Izsak, R.; Neese, F.; Klopper, W.
Robust fitting techniques in the chain of spheres approximation to the Fock exchange: The role of the complementary space
J. Chem. Phys. 2013 139 ,
doi.org/10.1063/1.4819264
5. Neese, F.
Software update: the ORCA program system, version 4.0
WIRES Comput. Molec. Sci. 2018 8(1), 1-6

```
     doi.org/10.1002/wcms.1327
  6. Neese, F.; Wennmohs, F.; Becker, U.; Riplinger, C.
     The ORCA quantum chemistry program package
     J. Chem. Phys. 2020 152(22), 224108
     doi.org/10.1063/5.0004608
  7. Neese, F.
     Software update: The ORCA program system—Version 5.0
     WIRES Comput. Molec. Sci. 2022 12(1), e1606
     doi.org/10.1002/wcms.1606

List of optional additional citations

  1. Neese, F.
     Approximate second-order SCF convergence for spin unrestricted wavefunctions
     Chem. Phys. Lett. 2000 325(1-3), 93-98
     doi.org/10.1016/s0009-2614(00)00662-x
```


```
Timings for individual modules:

Sum of individual times          ...   116774.209 sec (=1946.237 min)
Startup calculation              ...     3107.352 sec (=  51.789 min)   2.7 %
SCF iterations                   ...    69490.280 sec (=1158.171 min)  59.5 %
Property calculations            ...        0.388 sec (=   0.006 min)   0.0 %
SCF Gradient evaluation          ...    44176.190 sec (= 736.270 min)  37.8 %
                             ****ORCA TERMINATED NORMALLY****
TOTAL RUN TIME: 1 days 8 hours 44 minutes 55 seconds 54 msec
'H-IRC.bibtex' -> '/hsnfs/users/barasits/Tunneling_selenoxide/H/IRC/H-IRC.bibtex'
'H-IRC.cpcm' -> '/hsnfs/users/barasits/Tunneling_selenoxide/H/IRC/H-IRC.cpcm'
'H-IRC.cpcm_corr' -> '/hsnfs/users/barasits/Tunneling_selenoxide/H/IRC/H-IRC.cpcm_corr'
'H-IRC.densities' -> '/hsnfs/users/barasits/Tunneling_selenoxide/H/IRC/H-IRC.densities'
'H-IRC.densitiesinfo' -> '/hsnfs/users/barasits/Tunneling_selenoxide/H/IRC/H-IRC.densitiesinfo'
'H-IRC.engrad' -> '/hsnfs/users/barasits/Tunneling_selenoxide/H/IRC/H-IRC.engrad'
'H-IRC.err' -> '/hsnfs/users/barasits/Tunneling_selenoxide/H/IRC/H-IRC.err'
'H-IRC.gbw' -> '/hsnfs/users/barasits/Tunneling_selenoxide/H/IRC/H-IRC.gbw'
'H-IRC_IRC_B.cpcm' -> '/hsnfs/users/barasits/Tunneling_selenoxide/H/IRC/H-IRC_IRC_B.cpcm'
'H-IRC_IRC_B.cpcm_corr' -> '/hsnfs/users/barasits/Tunneling_selenoxide/H/IRC/H-
IRC_IRC_B.cpcm_corr'
'H-IRC_IRC_B.densities' -> '/hsnfs/users/barasits/Tunneling_selenoxide/H/IRC/H-
IRC_IRC_B.densities'
'H-IRC_IRC_B.densitiesinfo' -> '/hsnfs/users/barasits/Tunneling_selenoxide/H/IRC/H-
IRC_IRC_B.densitiesinfo'
'H-IRC_IRC_B.gbw' -> '/hsnfs/users/barasits/Tunneling_selenoxide/H/IRC/H-IRC_IRC_B.gbw'
'H-IRC_IRC_B.smd' -> '/hsnfs/users/barasits/Tunneling_selenoxide/H/IRC/H-IRC_IRC_B.smd'
'H-IRC_IRC_B.smd.grd' -> '/hsnfs/users/barasits/Tunneling_selenoxide/H/IRC/H-IRC_IRC_B.smd.grd'
'H-IRC_IRC_B_trj.xyz' -> '/hsnfs/users/barasits/Tunneling_selenoxide/H/IRC/H-IRC_IRC_B_trj.xyz'
'H-IRC_IRC_B.xyz' -> '/hsnfs/users/barasits/Tunneling_selenoxide/H/IRC/H-IRC_IRC_B.xyz'
'H-IRC_IRC_F.cpcm' -> '/hsnfs/users/barasits/Tunneling_selenoxide/H/IRC/H-IRC_IRC_F.cpcm'
'H-IRC_IRC_F.cpcm_corr' -> '/hsnfs/users/barasits/Tunneling_selenoxide/H/IRC/H-
IRC_IRC_F.cpcm_corr'
'H-IRC_IRC_F.densities' -> '/hsnfs/users/barasits/Tunneling_selenoxide/H/IRC/H-
IRC_IRC_F.densities'
'H-IRC_IRC_F.densitiesinfo' -> '/hsnfs/users/barasits/Tunneling_selenoxide/H/IRC/H-
IRC_IRC_F.densitiesinfo'
'H-IRC_IRC_F.gbw' -> '/hsnfs/users/barasits/Tunneling_selenoxide/H/IRC/H-IRC_IRC_F.gbw'
'H-IRC_IRC_F.smd' -> '/hsnfs/users/barasits/Tunneling_selenoxide/H/IRC/H-IRC_IRC_F.smd'
'H-IRC_IRC_F.smd.grd' -> '/hsnfs/users/barasits/Tunneling_selenoxide/H/IRC/H-IRC_IRC_F.smd.grd'
'H-IRC_IRC_F_trj.xyz' -> '/hsnfs/users/barasits/Tunneling_selenoxide/H/IRC/H-IRC_IRC_F_trj.xyz'
'H-IRC_IRC_Full_trj.xyz' -> '/hsnfs/users/barasits/Tunneling_selenoxide/H/IRC/H-
IRC_IRC_Full_trj.xyz'
'H-IRC_IRC_F.xyz' -> '/hsnfs/users/barasits/Tunneling_selenoxide/H/IRC/H-IRC_IRC_F.xyz'
'H-IRC.job' -> '/hsnfs/users/barasits/Tunneling_selenoxide/H/IRC/H-IRC.job'
'H-IRC.property.txt' -> '/hsnfs/users/barasits/Tunneling_selenoxide/H/IRC/H-IRC.property.txt'
'H-IRC.smd' -> '/hsnfs/users/barasits/Tunneling_selenoxide/H/IRC/H-IRC.smd'
```

```
'H-IRC.smd.grd' -> '/hsnfs/users/barasits/Tunneling_selenoxide/H/IRC/H-IRC.smd.grd'
'TS-H_Compound_3.hess' -> '/hsnfs/users/barasits/Tunneling_selenoxide/H/IRC/TS-
H_Compound_3.hess'
cp: cannot stat 'H-IRC.asa.inp': No such file or directory
cp: missing destination file operand after '/hsnfs/users/barasits/Tunneling_selenoxide/H/IRC/'
Try 'cp --help' for more information.
cp: cannot stat 'H-IRC.pc.tmp': No such file or directory
cp: cannot stat 'H-IRC.QM2.pc.tmp': No such file or directory
```

IRC output for R = Cl

```
! Job execution by suborca V 10.04 !
Job started from head2.mpi-muelheim.mpg.de, running
/hsnfs/software/orca/orca_current_directory/orca_current_20251201/orca on xe30th119.mpi-
muelheim.mpg.de in /usr/scratch/barasits/Cl-IRC__26954862 (/bin/bash)
Job execution start: Mon Dec  1 04:40:58 PM CET 2025
Shared library path: /hsnfs/software/openmpi-
4.1.0/lib:/hsnfs/software/orca/orca_current_directory/orca_current_20251201:/hsnfs/software/the
mis/llvm-14.0.1/lib:/hsnfs/software/orca/orca_stable:/usr/users/openmpi-
2.0.4/lib:/opt/intel/lib/intel64:/opt/intel/mkl/lib/intel64
SLURM Job ID is: 26954862
SLURM_JOB_NODELIST: xe30th119
Maxcore enforced by SLURM: 9600 MB per core
Expanded nodelist (1 nodes):
xe30th119

                                 *****************
                                 * O   R   C   A *
                                 *****************

                                            #,
                                            ###
                                            ####
                                            #####
                                            ######
                                           ########,
                                     ,,################,,,,,
                               ,,#################################,,
                          ,,##########################################,,
                       ,#########################################, ''#####,
                    ,#############################################,,   '####,
                  ,##################################################,,,,####,
                ,###########''''           ''''###############################
              ,#####''   ,,,,##########,,,,          '''####'''          '####
            ,##' ,,,,###########################,,,                        '##
           ' ,,###''''                  '''############,,,
         ,,##''                                '''############,,,,        ,,,,,,###''
      ,#''                                            '''#######################'''
     '                                                          ''''####''''
             ,#######,   #######,   ,#######,      ##
            ,#'     '#,  ##    ##  ,#'     '#,    #''#        ,####,    ,#,
            ##       ##  ##   ,#'  ##            #'  '#       #'       ,# #
            ##       ##  #######   ##           ,######,      #####,      #
            '#,     ,#'  ##    ##  '#,     ,#' ,#      #,     #,   #      #
             '#######'   ##     ##  '#######'  #'      '#     '####' #    #


                  #########################################################
                  #                        -***-                          #
                  #          Department of theory and spectroscopy        #
                  #                                                       #
                  #                      Frank Neese                      #
                  #                                                       #
                  #     Directorship, Architecture, Infrastructure        #
                  #                    SHARK, DRIVERS                     #
                  #        Core code/Algorithms in most modules           #
                  #                                                       #
                  #        Max Planck Institute fuer Kohlenforschung      #
                  #                Kaiser Wilhelm Platz 1                 #
                  #                 D-45470 Muelheim/Ruhr                 #
                  #                      Germany                          #
                  #                                                       #
                  #                  All rights reserved                  #
```

```
                  #                        -***-                        #
                  #######################################################


                           Program Version 6.1.x  -  CURRENT   -
                                  (GIT: $11b74ca1$)
                  ($2025-11-30 13:13:46 +0100 $)

                    !                    WARNING:                        !
                    !         This is a DEVELOPMENT version              !

                           (BUILD: $Mon 01 Dec 2025 12:05:39 AM CET$)


 With contributions from (in alphabetic order):
[Max-Planck-Institut fuer Kohlenforschung]
  Daniel Aravena         : Magnetic Suceptibility
  Michael Atanasov       : Ab Initio Ligand Field Theory (pilot matlab implementation)
  Alexander A. Auer      : GIAO ZORA, VPT2 properties, NMR spectrum
  Ute Becker             : All parallelization in ORCA, NUMFREQ, NUMCALC
  Giovanni Bistoni       : ED, misc. LED, open-shell LED, HFLD
  Dmytro Bykov           : pre 5.0 version of the SCF Hessian
  Marcos Casanova-Páez   : Triplet and SCS-CIS(D). UHF-(DLPNO)-IP/EA/STEOM-CCSD. UHF-CVS-
IP/STEOM-CCSD
  Vijay G. Chilkuri      : MRCI spin determinant printing, contributions to CSF-ICE
  Pauline Colinet        : FMM embedding
  Dipayan Datta          : RHF DLPNO-CCSD density
  Achintya Kumar Dutta   : EOM-CC, STEOM-CC
  Nicolas Foglia         : Exact transition moments, OPA infrastructure, MCD improvements
  Dmitry Ganyushin       : Spin-Orbit,Spin-Spin,Magnetic field MRCI
  Miquel Garcia-Rates    : C-PCM and meta-GGA Hessian, CCSD/C-PCM, Gaussian charge scheme
  Tiago L. C. Gouveia    : GS-ROHF, GS-ROCIS
  Yang Guo               : DLPNO-NEVPT2, F12-NEVPT2, CIM, IAO-localization
  Andreas Hansen         : Spin unrestricted coupled pair/coupled cluster methods
  Ingolf Harden          : AUTO-CI MPn and infrastructure
  Benjamin Helmich-Paris : MC-RPA, TRAH-(SCF,CASSCF), AVAS, COSX integrals, SCF dyn. polar.,
MC-PDFT, srDFT
  Lee Huntington         : MR-EOM, pCC
  Robert Izsak           : Overlap fitted RIJCOSX, COSX-SCS-MP3, EOM
  Riya Kayal             : Wick's Theorem for AUTO-CI, AUTO-CI UHF-CCSDT
  Emily Kempfer          : AUTO-CI RHF CISDT and CCSDT, approximate NEVPT4
  Christian Kollmar      : KDIIS, OOCD, Brueckner-CCSD(T), CCSD density, CASPT2, CASPT2-K,
improved NEVPT2
  Axel Koslowski         : Symmetry handling
  Simone Kossmann        : meta-GGA functionals, TD-DFT gradient, OOMP2, (MP2 Hessian;
deprecated post 5.0)
  Lucas Lang             : DCDCAS, Hyperfine gauge corrections, ICE-SOC+SSC
  Marvin Lechner         : AUTO-CI (C++ implementation), FIC-MRCC
  Spencer Leger          : CASSCF response
  Dagmar Lenk            : GEPOL surface, SMD, ORCA-2-JSON
  Dimitrios Liakos       : Extrapolation schemes; Compound Job, Property file
  Dimitrios Manganas     : Further ROCIS development; embedding schemes. LFT, Crystal Embedding
  Hagen Neugebauer       : wr2SCAN, Native XTB
  Dimitrios Pantazis     : SARC Basis sets
  Anastasios Papadopoulos: AUTO-CI, single reference methods and gradients
  Taras Petrenko         : pre 6.0 DFT Hessian and TD-DFT gradient,  ECA, NRVS
  Petra Pikulova         : Analytic Raman intensities
  Peter Pinski           : DLPNO-MP2, DLPNO-MP2 Gradient
  Shashank Vittal Rao    : ES-AILFT, MagRelax
  Christoph Reimann      : Effective Core Potentials
  Marius Retegan         : Local ZFS, SOC
  Christoph Riplinger    : Optimizer, TS searches, QM/MM, DLPNO-CCSD(T), (RO)-DLPNO pert.
Triples
```

```
  Michael Roemelt        : Original ROCIS implementation, recursive CI coupling coefficients
  Masaaki Saitow         : Open-shell DLPNO-CCSD energy and density
  Barbara Sandhoefer     : DKH picture change effects
  Yorick L. A. Schmerwitz: GMF and freeze-and-release deltaSCF, NEB S-IDPP initial path
  Kantharuban Sivalingam : CASSCF convergence/infrastructure, NEVPT2, NEVPT3, NEVPT4(SD), FIC-
MRCI and CEPA variants
  Bernardo de Souza      : ESD, SOC TD-DFT
  Georgi L. Stoychev     : AutoAux, RI-MP2 NMR, DLPNO-MP2 response, X2C
  Van Anh Tran           : RI-MP2 g-tensors
  Willem Van den Heuvel  : Paramagnetic NMR
  Zikuan Wang            : NOTCH, Electric field optimization
  Frank Wennmohs         : Technical directorship and infrastructure
  Hang Xu                : AUTO-CI-Response properties

[FACCTs GmbH]
  Markus Bursch, Nicolas Foglia, Miquel Garcia-Rates, Ingolf Harden, Hagen Neugebauer,
Anastasios Papadopoulos,
  Christoph Riplinger, Bernardo de Souza, Georgi L. Stoychev

  APM, various basis sets, CI-OPT, improved COSX, DLPNO-Multilevel,
  DOCKER, DRACO, updates on ESD, Fragmentator, GOAT, IRC, LR-CPCM, L-BFGS, MBIS, meta-GGA TD-
DFT gradient, ML-optimized integration grids,
  MM, NACMEs, nearIR, NEB, NEB-TS, NL-DFT gradient (VV10), 2- and 3-layer-ONIOM, interface
openCOSMO-RS, QMMM,
  Crystal-QMMM, RESP, rigid body optimization, SF, symmetry and pop. for TD-DFT, various
functionals, SOLVATOR

[Other institutions]
  V. Asgeirsson          : NEB
  Christoph Bannwarth    : sTDA-DFT, sTD-DFT, PBEh-3c, B97-3c, D3
  Giovanni Bistoni       : ETS/NOCV, ADLD/ADEX, COVALED
  Martin Brehm           : Molecular dynamics
  Ronald Cardenas        : ETS/NOCV
  Martina Colucci        : COVALED
  Sebastian Ehlert       : rSCAN, r2SCAN, r2SCAN-3c, D4, dhf basis sets
  Marvin Friede          : D4 for Fr, Ra, Ac-Lr
  Lars Goerigk           : TD-DFT with DH, B97 family of functionals
  Stefan Grimme          : VdW corrections, initial TS optimization, DFT functionals, gCP,
sTDA/sTD-DF
  Waldemar Hujo          : DFT-NL
  H. Jonsson             : NEB
  Holger Kruse           : gCP
  Marcel Mueller         : wB97X-3c, vDZP basis set
  Gianluca Regni         : ADLD/ADEX
  Tobias Risthaus        : pre 6.0 range-separated hybrid DFT and stability analysis
  Lukas Wittmann         : regularized MP2, r2SCAN double-hybrids, wr2SCAN

We gratefully acknowledge several colleagues who have allowed us to
interface, adapt or use parts of their codes:
  Ed Valeev, F. Pavosevic, A. Kumar             : LibInt (2-el integral package), F12 methods
  Garnet Chan, S. Sharma, J. Yang, R. Olivares  : DMRG
  Ulf Ekstrom                                   : XCFun DFT Library
  Mihaly Kallay                                 : mrcc  (arbitrary order and MRCC methods)
  Frank Weinhold                                : gennbo (NPA and NBO analysis)
  Simon Mueller                                 : openCOSMO-RS
  Christopher J. Cramer and Donald G. Truhlar   : smd solvation model
  S Lehtola, MJT Oliveira, MAL Marques          : LibXC Library
  Liviu Ungur et al                             : ANISO software


 Your calculation uses the libint2 library for the computation of 2-el integrals
 For citations please refer to: http://libint.valeyev.net
```

```
 Your ORCA version has been built with support for libXC version: 7.0.0
 For citations please refer to: https://libxc.gitlab.io

 This ORCA versions uses:
   CBLAS   interface :  Fast vector & matrix operations
   LAPACKE interface :  Fast linear algebra routines
   SCALAPACK package :  Parallel linear algebra routines
   Shared memory     :  Shared parallel matrices
   BLAS/LAPACK       :  OpenBLAS 0.3.26  USE64BITINT DYNAMIC_ARCH NO_AFFINITY Haswell
SINGLE_THREADED
        Core in use  :  Haswell
   Copyright (c) 2011-2014, The OpenBLAS Project


  ***********************************
  * Starting time: Mon Dec  1 16:40:58 2025
  * Host name:     xe30th119.mpi-muelheim.mpg.de
  * Process ID:    4052934
  * Working dir.:  /usr/scratch/barasits/Cl-IRC__26954862
  ***********************************

NOTE: MaxCore=8000 MB was set to SCF,MP2,MDCI,CIPSI,MRCI,RASCI and CIS
      => If you want to overwrite this, your respective input block should be placed after the
MaxCore statement
Your calculation utilizes the atom-pairwise dispersion correction
with the Becke-Johnson damping scheme (D3BJ)


Your calculation utilizes the SMD solvation module
Please cite in your paper:
Marenich, A. V.; Cramer, C. J.; Truhlar, D. G. J. Phys. Chem. B 2009, 113, 6378

================================================================================

----- Orbital basis set information -----
Your calculation utilizes the basis: def2-TZVP
   F. Weigend and R. Ahlrichs, Phys. Chem. Chem. Phys. 7, 3297 (2005).

----- AuxJ basis set information -----
Your calculation utilizes the auxiliary basis: def2/J
   H-Rn: F. Weigend, Phys. Chem. Chem. Phys. 8, 1057 (2006).
   Fr-Lr: K. Eichkorn, F. Weigend, O. Treutler, R. Ahlrichs; Theor. Chem. Acc. 97, 119 (1997).

================================================================================
                                        WARNINGS
                       Please study these warnings very carefully!
================================================================================

WARNING: SinglePoint found together with IRC
         Switching on EnGrad - energy plus gradient calculation

================================================================================
                                       INPUT FILE
================================================================================
NAME = Cl-IRC.inp
|  1> #
|  2> #IRC LQA
|  3> #
|  4> ! irc B3LYP D3BJ def2-TZVP def2/J verytightscf
|  5> %pal nprocs 12 end
|  6> %maxcore 8000
|  7> %irc
|  8> printlevel 1
```

```
|  9> method lqa
| 10> maxiter 241
| 11> tolrmsg 1.e-5 # Tighter convergence criteria
| 12> tolmaxg 8.e-5 # Tighter convergence criteria
| 13> follow_coordtype massweighted
| 14> inithess read
| 15> hess_filename "TS-Cl_Compound_2.hess"
| 16> lqahess lqacalc_bofillupdate
| 17> init_displ length
| 18> scale_init_displ 0.04
| 19> adapt_scale_displ false
| 20> scale_displ_sd 0.11
| 21> ENFORCESTRICTCONVERGENCE true
| 22> REPORTPROFILE true
| 23> BSCORRECTOR true
| 24> BSRICHTHRESH 1E-6
| 25> INITBSCORRECTOR false
| 26> end
| 27> %CPCM SMD true
| 28>      SMDSOLVENT "TETRAHYDROFURAN" END
| 29>
| 30> * xyz 0 1
| 31> C    -1.773872427369      0.370470891402     -6.064873419397
| 32> C    -1.257942913186     -0.293604436747     -4.951751723055
| 33> C    -0.972032827277      1.325095278614     -6.695917938730
| 34> C     0.018192987577     -0.010162043334     -4.477442588229
| 35> C     0.307693032359      1.606480456046     -6.231068079564
| 36> C     0.807339674561      0.938796186736     -5.117139330094
| 37> H    -1.863357316266     -1.039862806876     -4.451438413368
| 38> H    -1.359145135523      1.854828296799     -7.558778724324
| 39> H     0.399460443302     -0.537066693136     -3.612301688332
| 40> H     0.913557670565      2.349086779838     -6.736036741301
| 41> H     1.804617605258      1.152482504015     -4.753256346986
| 42> C    -3.155682211483      0.067925501241     -6.580488319370
| 43> H    -3.606307239862      0.999780465484     -6.936000980354
| 44> H    -3.778631676217     -0.282983967097     -5.754968118203
| 45> C    -3.229526222284     -0.958534228976     -7.723614994047
| 46> H    -3.147005653943     -2.141434869463     -7.163782419902
| 47> H    -4.232008713290     -1.034180772458     -8.146536575959
| 48> C    -2.193941526621     -1.093392494933     -8.666784114724
| 49> H    -2.418874444562     -1.365741112203     -9.688859016450
| 50> H    -1.227264633713     -0.632175247143     -8.514204513235
| 51> Se   -1.452734411582     -3.439624978748     -8.074595098410
| 52> O    -2.743735419766     -3.376935509137     -6.943571487493
| 53> C     0.072427916578     -3.006673281001     -6.965647524079
| 54> C     0.136388084590     -3.480849419524     -5.659904539563
| 55> C     1.124251872718     -2.270664668529     -7.500658730175
| 56> C     1.251884517340     -3.211234986242     -4.878963058278
| 57> C     2.245400875948     -1.998796949119     -6.726451662410
| 58> C     2.292841433877     -2.470608912941     -5.422459018026
| 59> H    -0.687035944434     -4.046998847171     -5.245591511702
| 60> H     1.072481646714     -1.892159990750     -8.513711571816
| 61> H     1.305715407360     -3.564396840620     -3.858393344796
| 62> H     3.059801791819     -1.411333207979     -7.127156857001
| 63> Cl    3.683482246812     -2.094094346046     -4.428888300628
| 64> *
| 65>
| 66>
| 67>                          ****END OF INPUT****
================================================================================

                    *******************************
                    * Energy+Gradient Calculation *
```

```
                    ******************************

--------------------------------
CARTESIAN COORDINATES (ANGSTROEM)
--------------------------------
  C     -1.773872    0.370471   -6.064873
  C     -1.257943   -0.293604   -4.951752
  C     -0.972033    1.325095   -6.695918
  C      0.018193   -0.010162   -4.477443
  C      0.307693    1.606480   -6.231068
  C      0.807340    0.938796   -5.117139
  H     -1.863357   -1.039863   -4.451438
  H     -1.359145    1.854828   -7.558779
  H      0.399460   -0.537067   -3.612302
  H      0.913558    2.349087   -6.736037
  H      1.804618    1.152483   -4.753256
  C     -3.155682    0.067926   -6.580488
  H     -3.606307    0.999780   -6.936001
  H     -3.778632   -0.282984   -5.754968
  C     -3.229526   -0.958534   -7.723615
  H     -3.147006   -2.141435   -7.163782
  H     -4.232009   -1.034181   -8.146537
  C     -2.193942   -1.093392   -8.666784
  H     -2.418874   -1.365741   -9.688859
  H     -1.227265   -0.632175   -8.514205
  Se    -1.452734   -3.439625   -8.074595
  O     -2.743735   -3.376936   -6.943571
  C      0.072428   -3.006673   -6.965648
  C      0.136388   -3.480849   -5.659905
  C      1.124252   -2.270665   -7.500659
  C      1.251885   -3.211235   -4.878963
  C      2.245401   -1.998797   -6.726452
  C      2.292841   -2.470609   -5.422459
  H     -0.687036   -4.046999   -5.245592
  H      1.072482   -1.892160   -8.513712
  H      1.305715   -3.564397   -3.858393
  H      3.059802   -1.411333   -7.127157
  Cl     3.683482   -2.094094   -4.428888

----------------------------
CARTESIAN COORDINATES (A.U.)
----------------------------
  NO LB      ZA    FRAG     MASS         X           Y           Z
   0 C     6.0000    0    12.011   -3.352133    0.700089  -11.460950
   1 C     6.0000    0    12.011   -2.377168   -0.554832   -9.357455
   2 C     6.0000    0    12.011   -1.836876    2.504067  -12.653451
   3 C     6.0000    0    12.011    0.034380   -0.019203   -8.461140
   4 C     6.0000    0    12.011    0.581456    3.035808  -11.775012
   5 C     6.0000    0    12.011    1.525651    1.774068   -9.669992
   6 H     1.0000    0     1.008   -3.521235   -1.965056   -8.412000
   7 H     1.0000    0     1.008   -2.568412    3.505118  -14.284022
   8 H     1.0000    0     1.008    0.754871   -1.014909   -6.826261
   9 H     1.0000    0     1.008    1.726374    4.439131  -12.729265
  10 H     1.0000    0     1.008    3.410233    2.177876   -8.982353
  11 C     6.0000    0    12.011   -5.963375    0.128361  -12.435321
  12 H     1.0000    0     1.008   -6.814933    1.889311  -13.107142
  13 H     1.0000    0     1.008   -7.140579   -0.534762  -10.875314
  14 C     6.0000    0    12.011   -6.102920   -1.811367  -14.595517
  15 H     1.0000    0     1.008   -5.946979   -4.046725  -13.537587
  16 H     1.0000    0     1.008   -7.997337   -1.954318  -15.394723
  17 C     6.0000    0    12.011   -4.145949   -2.066212  -16.377848
  18 H     1.0000    0     1.008   -4.571010   -2.580877  -18.309290
  19 H     1.0000    0     1.008   -2.319194   -1.194638  -16.089515
```

```
  20 Se   34.0000    0    78.960   -2.745270   -6.499949  -15.258773
  21 O     8.0000    0    15.999   -5.184909   -6.381483  -13.121449
  22 C     6.0000    0    12.011    0.136869   -5.681789  -13.163166
  23 C     6.0000    0    12.011    0.257736   -6.577852  -10.695670
  24 C     6.0000    0    12.011    2.124528   -4.290934  -14.174191
  25 C     6.0000    0    12.011    2.365719   -6.068355   -9.219904
  26 C     6.0000    0    12.011    4.243193   -3.777179  -12.711151
  27 C     6.0000    0    12.011    4.332842   -4.668774  -10.246963
  28 H     1.0000    0     1.008   -1.298310   -7.647719   -9.912731
  29 H     1.0000    0     1.008    2.026697   -3.575664  -16.088583
  30 H     1.0000    0     1.008    2.467445   -6.735734   -7.291307
  31 H     1.0000    0     1.008    5.782187   -2.667033  -13.468375
  32 Cl   17.0000    0    35.453    6.960773   -3.957265   -8.369386

--------------------------------
INTERNAL COORDINATES (ANGSTROEM)
--------------------------------
 C      0   0   0     0.000000000000     0.00000000     0.00000000
 C      1   0   0     1.395069609707     0.00000000     0.00000000
 C      1   2   0     1.397308716897   118.25056018     0.00000000
 C      2   1   3     1.390622732081   120.96386389     0.28469112
 C      3   1   2     1.390309780397   121.02927609     0.12149297
 C      4   2   1     1.390138891459   120.20692719   359.50026296
 H      2   1   3     1.083393539899   119.32056224   180.23294941
 H      3   1   2     1.083974974373   119.23736771   180.35177593
 H      4   2   1     1.082341055053   119.77967997   180.00843018
 H      5   3   1     1.083295648150   119.84664455   179.89357845
 H      6   4   2     1.082883160199   120.19022868   180.58643302
 C      1   2   3     1.505586502880   121.13250326   179.79868030
 H     12   1   2     1.094443165590   108.54434888   143.49634371
 H     12   1   2     1.092102144086   109.22113720    27.74361439
 C     12   1   2     1.538119341939   115.63814654   264.84218309
 H     15  12   1     1.311287947225   106.36113136    80.37871123
 H     15  12   1     1.090667781115   112.24690433   187.90722616
 C     15  12   1     1.407192383431   121.78622765   328.66331062
 H     18  15  12     1.081390628608   120.30125317   213.11100233
 H     18  15  12     1.081880815863   121.47833492    12.67373806
 Se    16  15  12     2.320653429073   112.52072114   233.91267083
 O     16  15  12     1.318173572471   159.40472447   228.65539471
 C     21  16  15     1.934769242684   103.02524260    90.38982040
 C     23  21  16     1.390646847711   119.87625562    72.32710903
 C     23  21  16     1.390783701009   119.61072200   249.56568460
 C     24  23  21     1.388126014202   119.93199011   177.69288140
 C     25  23  21     1.389346511162   119.96572113   182.42855886
 C     27  25  23     1.387535207607   118.99584508   359.94394471
 H     24  23  21     1.081761337382   120.20253359   356.78631115
 H     25  23  21     1.082692018701   120.58295895     3.38476122
 H     26  24  23     1.081287935836   120.50173178   179.19937383
 H     27  25  23     1.081169344690   120.50732514   181.18242032
 Cl    28  27  25     1.750093671823   119.17999735   181.64666796

---------------------------
INTERNAL COORDINATES (A.U.)
---------------------------
 C      0   0   0     0.000000000000     0.00000000     0.00000000
 C      1   0   0     2.636299500103     0.00000000     0.00000000
 C      1   2   0     2.640530799476   118.25056018     0.00000000
 C      2   1   3     2.627896119238   120.96386389     0.28469112
 C      3   1   2     2.627304726262   121.02927609     0.12149297
 C      4   2   1     2.626981792971   120.20692719   359.50026296
 H      2   1   3     2.047317085668   119.32056224   180.23294941
 H      3   1   2     2.048415837589   119.23736771   180.35177593
 H      4   2   1     2.045328177549   119.77967997   180.00843018
```

```
 H      5   3   1     2.047132097072   119.84664455   179.89357845
 H      6   4   2     2.046352607812   120.19022868   180.58643302
 C      1   2   3     2.845146161372   121.13250326   179.79868030
 H     12   1   2     2.068197852106   108.54434888   143.49634371
 H     12   1   2     2.063773962590   109.22113720    27.74361439
 C     12   1   2     2.906624317552   115.63814654   264.84218309
 H     15  12   1     2.477975102967   106.36113136    80.37871123
 H     15  12   1     2.061063409398   112.24690433   187.90722616
 C     15  12   1     2.659208222425   121.78622765   328.66331062
 H     18  15  12     2.043532131857   120.30125317   213.11100233
 H     18  15  12     2.044458451524   121.47833492    12.67373806
 Se    16  15  12     4.385399432693   112.52072114   233.91267083
 O     16  15  12     2.490987048942   159.40472447   228.65539471
 C     21  16  15     3.656184001007   103.02524260    90.38982040
 C     23  21  16     2.627941691175   119.87625562    72.32710903
 C     23  21  16     2.628200306428   119.61072200   249.56568460
 C     24  23  21     2.623178006213   119.93199011   177.69288140
 C     25  23  21     2.625484411215   119.96572113   182.42855886
 C     27  25  23     2.622061543551   118.99584508   359.94394471
 H     24  23  21     2.044232669916   120.20253359   356.78631115
 H     25  23  21     2.045991402726   120.58295895     3.38476122
 H     26  24  23     2.043338070643   120.50173178   179.19937383
 H     27  25  23     2.043113965856   120.50732514   181.18242032
 Cl    28  27  25     3.307197748454   119.17999735   181.64666796

---------------------
BASIS SET INFORMATION
---------------------
There are 5 groups of distinct atoms

 Group   1 Type C   : 11s6p2d1f contracted to 5s3p2d1f pattern {62111/411/11/1}
 Group   2 Type H   : 5s1p contracted to 3s1p pattern {311/1}
 Group   3 Type Se  : 17s13p8d1f contracted to 6s5p4d1f pattern {842111/64111/5111/1}
 Group   4 Type O   : 11s6p2d1f contracted to 5s3p2d1f pattern {62111/411/11/1}
 Group   5 Type Cl  : 14s9p3d1f contracted to 5s5p2d1f pattern {73211/51111/21/1}

Atom   0C    basis set group =>   1
Atom   1C    basis set group =>   1
Atom   2C    basis set group =>   1
Atom   3C    basis set group =>   1
Atom   4C    basis set group =>   1
Atom   5C    basis set group =>   1
Atom   6H    basis set group =>   2
Atom   7H    basis set group =>   2
Atom   8H    basis set group =>   2
Atom   9H    basis set group =>   2
Atom  10H    basis set group =>   2
Atom  11C    basis set group =>   1
Atom  12H    basis set group =>   2
Atom  13H    basis set group =>   2
Atom  14C    basis set group =>   1
Atom  15H    basis set group =>   2
Atom  16H    basis set group =>   2
Atom  17C    basis set group =>   1
Atom  18H    basis set group =>   2
Atom  19H    basis set group =>   2
Atom  20Se   basis set group =>   3
Atom  21O    basis set group =>   4
Atom  22C    basis set group =>   1
Atom  23C    basis set group =>   1
Atom  24C    basis set group =>   1
Atom  25C    basis set group =>   1
Atom  26C    basis set group =>   1
```

```
Atom  27C    basis set group =>   1
Atom  28H    basis set group =>   2
Atom  29H    basis set group =>   2
Atom  30H    basis set group =>   2
Atom  31H    basis set group =>   2
Atom  32Cl   basis set group =>   5
---------------------------------
AUXILIARY/J BASIS SET INFORMATION
---------------------------------
There are 5 groups of distinct atoms

 Group   1 Type C   : 12s5p4d2f1g contracted to 6s4p3d1f1g pattern {711111/2111/211/2/1}
 Group   2 Type H   : 5s2p1d contracted to 3s1p1d pattern {311/2/1}
 Group   3 Type Se  : 19s5p5d3f1g contracted to 8s4p3d2f1g pattern {121111111/2111/311/21/1}
 Group   4 Type O   : 12s5p4d2f1g contracted to 6s4p3d1f1g pattern {711111/2111/211/2/1}
 Group   5 Type Cl  : 14s5p5d2f1g contracted to 8s4p3d1f1g pattern {71111111/2111/311/2/1}

Atom   0C    basis set group =>   1
Atom   1C    basis set group =>   1
Atom   2C    basis set group =>   1
Atom   3C    basis set group =>   1
Atom   4C    basis set group =>   1
Atom   5C    basis set group =>   1
Atom   6H    basis set group =>   2
Atom   7H    basis set group =>   2
Atom   8H    basis set group =>   2
Atom   9H    basis set group =>   2
Atom  10H    basis set group =>   2
Atom  11C    basis set group =>   1
Atom  12H    basis set group =>   2
Atom  13H    basis set group =>   2
Atom  14C    basis set group =>   1
Atom  15H    basis set group =>   2
Atom  16H    basis set group =>   2
Atom  17C    basis set group =>   1
Atom  18H    basis set group =>   2
Atom  19H    basis set group =>   2
Atom  20Se   basis set group =>   3
Atom  21O    basis set group =>   4
Atom  22C    basis set group =>   1
Atom  23C    basis set group =>   1
Atom  24C    basis set group =>   1
Atom  25C    basis set group =>   1
Atom  26C    basis set group =>   1
Atom  27C    basis set group =>   1
Atom  28H    basis set group =>   2
Atom  29H    basis set group =>   2
Atom  30H    basis set group =>   2
Atom  31H    basis set group =>   2
Atom  32Cl   basis set group =>   5


           ************************************************************
           *        Program running with 12 parallel MPI-processes    *
           *              working on a common directory               *
           ************************************************************
------------------------------------------------------------------------------
                            ORCA STARTUP CALCULATIONS
                          -- RI-GTO INTEGRALS CHOSEN --
------------------------------------------------------------------------------
------------------------------------------------------------------------------

                   ___
                  /   \      - P O W E R E D   B Y -
```

```
                          /      \
                          |  |   |
                          |  |   |  _    _      __       _____    __    __
                          |  |   | | |  | |    /  \     |  _  \  |  |  /  |
                           \  \/   | |  | |   /    \    | | | |  |  | /  /
                          / \  \   | |__| |  /  /\  \   | |_| |  |  |/  /
                         |  |  |   |  __  | /  /__\  \  |    /   |      \
                         |  |  |   | |  | | |   __   |  |    \   |  |\   \
                         \     /   | |  | | |  |  |  |  | |\  \  |  |  \  \
                          \___/    |_|  |_| |__|  |__|  |_| \__\ |__|   \__/

                      - O R C A ' S   B I G   F R I E N D -
                                      &
                       - I N T E G R A L   F E E D E R -

 v1 FN, 2020, v2 2021, v3 2022-2024
------------------------------------------------------------------------------


----------------------
SHARK INTEGRAL PACKAGE
----------------------

Number of atoms                             ...     33
Number of basis functions                   ...    671
Number of shells                            ...    265
Maximum angular momentum                    ...      3
Integral batch strategy                     ... SHARK/LIBINT Hybrid
RI-J (if used) integral strategy            ... SPLIT-RIJ (Revised 2003 algorithm where
possible)
Printlevel                                  ...      1
Contraction scheme used                     ... SEGMENTED contraction
Prescreening option                         ... SCHWARTZ
   Thresh                                   ... 1.000e-12
   Tcut                                     ... 1.000e-14
   Tpresel                                  ... 1.000e-14
Coulomb Range Separation                    ... NOT USED
Exchange Range Separation                   ... NOT USED
Multipole approximations                    ... NOT USED
Finite Nucleus Model                        ... NOT USED
CABS basis                                  ... NOT available
Auxiliary Coulomb fitting basis             ... AVAILABLE
   # of basis functions in Aux-J            ...   1058
   # of shells in Aux-J                     ...    350
   Maximum angular momentum in Aux-J        ...      4
Auxiliary J/K fitting basis                 ... NOT available
Auxiliary Correlation fitting basis         ... NOT available
Auxiliary 'external' fitting basis          ... NOT available

Checking pre-screening integrals            ... done (  0.0 sec) Dimension = 265
   => SHARK Basis and OBASIS are compatible. Storing Pre-screening
Shell pair information
Shell pair cut-off parameter TPreSel        ...   1.0e-14
Total number of shell pairs                 ...     35245
Shell pairs after pre-screening             ...     29567
Total number of primitive shell pairs       ...    114025
Primitive shell pairs kept                  ...     64092
          la=0 lb=0:   7468 shell pairs
          la=1 lb=0:   8402 shell pairs
          la=1 lb=1:   2401 shell pairs
          la=2 lb=0:   4318 shell pairs
          la=2 lb=1:   2462 shell pairs
          la=2 lb=2:    661 shell pairs
          la=3 lb=0:   1953 shell pairs
```

```
          la=3 lb=1:   1149 shell pairs
          la=3 lb=2:    602 shell pairs
          la=3 lb=3:    151 shell pairs

Checking whether 4 symmetric matrices of dimension 671 fit in memory:
Max Core in MB      =   8000.00
 MB in use           =     40.08
 MB left             =   7959.92
 MB needed           =      6.88
 Data fit in memory  = YES
Calculating RI/J V-Matrix + Cholesky decomp.... done (  0.2 sec)
Calculating Nuclear repulsion               ... done (  0.0 sec) ENN=   1882.274438272222 Eh

Diagonalization of the overlap matrix:
Smallest eigenvalue                        ... 6.413e-06
Time for diagonalization                   ...    0.080 sec
Threshold for overlap eigenvalues          ... 1.000e-07
Number of eigenvalues below threshold      ... 0
Time for construction of square roots      ...    0.050 sec
Total time needed                          ...    0.135 sec

-------------------
DFT GRID GENERATION
-------------------

General Integration Accuracy     IntAcc      ... 4.388
Radial Grid Type                 RadialGrid  ... OptM3 with GC (2021)
Angular Grid (max. ang.)         AngularGrid ... 4 (Lebedev-302)
Angular grid pruning method      GridPruning ... 4 (adaptive)
Weight generation scheme         WeightScheme... mBecke (2022)
Basis function cutoff            BFCut       ... 1.0000e-12
Integration weight cutoff        WCut        ... 1.0000e-14
Partially contracted basis set               ... off
Rotationally invariant grid construction     ... off
Angular grids for H and He will be reduced by one unit
Core-polarized basis detected: some atoms will have their core
                               angular grid increased.

Total number of grid points                  ...   161478
Total number of batches                      ...     2538
Average number of points per batch           ...       63
Average number of grid points per atom       ...     4893

--------------------
COSX GRID GENERATION
--------------------

GRIDX 1
-------
General Integration Accuracy     IntAcc      ... 3.816
Radial Grid Type                 RadialGrid  ... OptM3 with GC (2021)
Angular Grid (max. ang.)         AngularGrid ... 1 (Lebedev-50)
Angular grid pruning method      GridPruning ... 4 (adaptive)
Weight generation scheme         WeightScheme... mBecke (2022)
Basis function cutoff            BFCut       ... 1.0000e-12
Integration weight cutoff        WCut        ... 1.0000e-14
Partially contracted basis set               ... on
Rotationally invariant grid construction     ... off
Angular grids for H and He will be reduced by one unit
Core-polarized basis detected: some atoms will have their core
                               angular grid increased.

Total number of grid points                  ...    26008
```

```
Total number of batches                      ...      218
Average number of points per batch           ...      119
Average number of grid points per atom       ...      788
UseSFitting                                  ... on

GRIDX 2
-------
General Integration Accuracy     IntAcc      ... 4.020
Radial Grid Type                 RadialGrid  ... OptM3 with GC (2021)
Angular Grid (max. ang.)         AngularGrid ... 2 (Lebedev-110)
Angular grid pruning method      GridPruning ... 4 (adaptive)
Weight generation scheme         WeightScheme... mBecke (2022)
Basis function cutoff            BFCut       ... 1.0000e-12
Integration weight cutoff        WCut        ... 1.0000e-14
Partially contracted basis set               ... on
Rotationally invariant grid construction     ... off
Angular grids for H and He will be reduced by one unit
Core-polarized basis detected: some atoms will have their core
                               angular grid increased.

Total number of grid points                  ...    46810
Total number of batches                      ...      383
Average number of points per batch           ...      122
Average number of grid points per atom       ...     1418
UseSFitting                                  ... on

GRIDX 3
-------
General Integration Accuracy     IntAcc      ... 4.338
Radial Grid Type                 RadialGrid  ... OptM3 with GC (2021)
Angular Grid (max. ang.)         AngularGrid ... 3 (Lebedev-194)
Angular grid pruning method      GridPruning ... 4 (adaptive)
Weight generation scheme         WeightScheme... mBecke (2022)
Basis function cutoff            BFCut       ... 1.0000e-12
Integration weight cutoff        WCut        ... 1.0000e-14
Partially contracted basis set               ... on
Rotationally invariant grid construction     ... off
Angular grids for H and He will be reduced by one unit
Core-polarized basis detected: some atoms will have their core
                               angular grid increased.

Total number of grid points                  ...    95762
Total number of batches                      ...      762
Average number of points per batch           ...      125
Average number of grid points per atom       ...     2902
UseSFitting                                  ... on
Grids setup in   2.5 sec
Initializing property integral containers    ... done (  0.0 sec)

SHARK setup successfully completed in   3.4 seconds

Maximum memory used throughout the entire STARTUP-calculation: 79.0 MB


           ************************************************************
           *        Program running with 12 parallel MPI-processes    *
           *              working on a common directory               *
           ************************************************************
------------------------------------------------------------------------------
                                 ORCA GUESS
                   Start orbitals & Density for SCF / CASSCF
------------------------------------------------------------------------------
```

```
------------
SCF SETTINGS
------------
Hamiltonian:
 Density Functional     Method          .... DFT(GTOs)
 Exchange Functional    Exchange        .... B88
   X-Alpha parameter    XAlpha          ....  0.666667
   Becke's b parameter  XBeta           ....  0.004200
 Correlation Functional Correlation     .... LYP
 LDA part of GGA corr.  LDAOpt          .... VWN-5
 Gradients option       PostSCFGGA      .... off
 Hybrid DFT is turned on
   Fraction HF Exchange ScalHFX         ....  0.200000
   Scaling of DF-GGA-X  ScalDFX         ....  0.720000
   Scaling of DF-GGA-C  ScalDFC         ....  0.810000
   Scaling of DF-LDA-C  ScalLDAC        ....  1.000000
   Perturbative correction              ....  0.000000
   NL short-range parameter             ....  4.800000
 RI-approximation to the Coulomb term is turned on
   Number of AuxJ basis functions       .... 1058
   RIJ-COSX (HFX calculated with COS-X)).... on


General Settings:
 Integral files         IntName         .... Cl-IRC
 Hartree-Fock type      HFTyp           .... RHF
 Total Charge           Charge          ....    0
 Multiplicity           Mult            ....    1
 Number of Electrons    NEL             ....  164
 Basis Dimension        Dim             ....  671
 Nuclear Repulsion      ENuc            ....   1882.2744382722 Eh

Convergence Acceleration:
 AO-DIIS                CNVDIIS         .... on
   Start iteration      DIISMaxIt       ....    12
   Startup error        DIISStart       ....  0.200000
   # of expansion vecs  DIISMaxEq       ....     5
   Bias factor          DIISBfac        ....   1.050
   Max. coefficient     DIISMaxC        ....  10.000
 MO-DIIS                CNVKDIIS        .... off
 Trust-Rad. Augm. Hess. CNVTRAH         .... auto
   Auto Start mean grad. ratio tolernc. ....  1.125000
   Auto Start start iteration           ....    50
   Auto Start num. interpolation iter.  ....    10
   Max. Number of Micro iterations      ....    24
   Max. Number of Macro iterations      .... Maxiter - #DIIS iter
   Number of Davidson start vectors     ....     2
   Converg. threshold    (grad. norm)   ....   2.000e-06
   Grad. Scal. Fac. for Micro threshold ....   0.100
   Minimum threshold for Micro iter.    ....   1.000e-02
   NR start threshold (gradient norm)   ....   1.000e-04
   Initial trust radius                 ....   0.400
   Minimum AH scaling param. (alpha)    ....   1.000
   Maximum AH scaling param. (alpha)    .... 1000.000
   Quad. conv. algorithm                .... NR
   White noise on init. David. guess    .... on
   Maximum white noise                  ....   0.010
   Pseudo random numbers                .... off
   Inactive MOs                         .... canonical
   Orbital update algorithm             .... Taylor
   Preconditioner                       .... Diag
   Full preconditioner red. dimension   ....   250
 SOSCF                 CNVSOSCF           .... on
```

```
   Start iteration     SOSCFMaxIt         ....   150
   Startup grad/error  SOSCFStart         ....  0.003300
   Hessian update      SOSCFHessUp        .... L-BFGS
   Autom. constraints  SOSCFAutoConstrain .... off
 Level Shifting         CNVShift       .... on
   Level shift para.    LevelShift      ....    0.2500
   Turn off err/grad.   ShiftErr        ....    0.0010
 Zerner damping         CNVZerner      .... off
 Static damping         CNVDamp        .... on
   Fraction old density DampFac         ....    0.7000
   Max. Damping (<1)    DampMax         ....    0.9800
   Min. Damping (>=0)   DampMin         ....    0.0000
   Turn off err/grad.   DampErr         ....    0.1000

SCF Procedure:
 Maximum # iterations   MaxIter         ....   125
 SCF integral mode      SCFMode         .... Direct
   Integral package                     .... SHARK and LIBINT hybrid scheme
 Reset frequency        DirectResetFreq ....    20
 Integral Threshold     Thresh          ....  1.000e-12 Eh
 Primitive CutOff       TCut            ....  1.000e-14 Eh

Convergence Tolerance:
 Convergence Check Mode ConvCheckMode   .... Total+1el-Energy
 Convergence forced     ConvForced      .... 0
 Energy Change          TolE            ....  1.000e-09 Eh
 1-El. energy change                    ....  1.000e-06 Eh
 Orbital Gradient       TolG            ....  2.000e-06
 Orbital Rotation angle TolX            ....  2.000e-06
 DIIS Error             TolErr          ....  1.000e-08

------------------------------
INITIAL GUESS: MODEL POTENTIAL
------------------------------
Loading Hartree-Fock densities                     ... done
Calculating cut-offs                               ... done
Initializing the effective Hamiltonian             ... done
Setting up the integral package (SHARK)            ... done
Starting the Coulomb interaction                   ... done (   0.4 sec)
Making the grid                                    ... done (   0.3 sec)
Mapping shells                                     ... done
Starting the XC term evaluation                    ... done (   0.2 sec)
  promolecular density results
     # of electrons  =    163.998532076
     EX              =   -202.919246946
     EC              =     -6.153872830
     EX+EC           =   -209.073119776
Transforming the Hamiltonian                       ... done (   0.1 sec)
Diagonalizing the Hamiltonian                      ... done (   0.1 sec)
Back transforming the eigenvectors                 ... done (   0.1 sec)
Now organizing SCF variables                       ... done
                      ------------------
                      INITIAL GUESS DONE (   1.1 sec)
                      ------------------
             **** ENERGY FILE WAS UPDATED (Cl-IRC.en.tmp) ****
Finished Guess after   1.5 sec
Maximum memory used throughout the entire GUESS-calculation: 46.0 MB


           ************************************************************
           *        Program running with 12 parallel MPI-processes    *
           *              working on a common directory               *
           ************************************************************
```

```
-------------------------------------------------------------------------------------------
                                     ORCA LEAN-SCF
                             memory conserving SCF solver
-------------------------------------------------------------------------------------------

--------------------
CPCM SOLVATION MODEL
--------------------
CPCM parameters:
  Epsilon                                         ...       7.4257
  Refrac                                          ...       1.4050
  Rsolv                                           ...       1.3000
  Surface type                                    ... GAUSSIAN VDW
  Discretization scheme                           ... Constant charge density
    Threshold for H atoms                         ...       5.0000 (charges/Ang^2)
    Threshold for non-H atoms                     ...       5.0000 (charges/Ang^2)
  Epsilon function type                           ...         CPCM
Solvent:                                          ... TETRAHYDROFURAN
SMD-CDS solvent descriptors:
  Soln                                            ...       1.4050
  Soln25                                          ...       1.4044
  Sola                                            ...       0.0000
  Solb                                            ...       0.4800
  Solg                                            ...      39.4400
  Solc                                            ...       0.0000
  Solh                                            ...       0.0000
Radii:
  Scheme                                          ... Element-dependent radii
  Radius for C  used is    3.4960 Bohr (=   1.8500 Ang.)
  Radius for H  used is    2.2677 Bohr (=   1.2000 Ang.)
  Radius for Se used is    3.5905 Bohr (=   1.9000 Ang.)
  Radius for O  used is    4.3350 Bohr (=   2.2940 Ang.)
  Radius for Cl used is    4.4975 Bohr (=   2.3800 Ang.)
Calculating surface                               ...        done! (  0.0s)
Cavity surface points                             ...         2681
Cavity Volume                                     ...    2112.5344
Cavity Surface-area                               ...    1138.0089
Calculating surface distance matrix               ...        done! (  0.1s)
Performing Cholesky decomposition & store         ...        done! (  0.3s)
Overall time for CPCM initialization              ...                 0.4s

----------------------------------------D-I-I-S--------------------------------------------
Iteration    Energy (Eh)           Delta-E    RMSDP     MaxDP     DIISErr   Damp  Time(sec)
-------------------------------------------------------------------------------------------
               ***  Starting incremental Fock matrix formation  ***
    1    -3516.6207253914521971     0.00e+00  5.82e-03  1.96e-01  3.12e-01  0.700   9.4
    2    -3516.9094097809952473    -2.89e-01  4.30e-03  1.56e-01  1.11e-01  0.700   6.0
                               ***Turning on AO-DIIS***
    3    -3517.0154573583299680    -1.06e-01  1.54e-03  3.18e-02  4.37e-02  0.700   5.7
    4    -3517.0799461802503174    -6.45e-02  3.09e-03  8.92e-02  2.04e-02  0.000   5.5
    5    -3517.2236101485918880    -1.44e-01  8.95e-04  2.66e-02  8.29e-03  0.000   5.7
                              *** Initializing SOSCF ***
---------------------------------------S-O-S-C-F--------------------------------------
Iteration    Energy (Eh)           Delta-E    RMSDP     MaxDP     MaxGrad    Time(sec)
--------------------------------------------------------------------------------------
    6    -3517.2249269544176968    -1.32e-03  3.85e-04  1.06e-02  2.98e-03     5.6
               *** Restarting incremental Fock matrix formation ***
    7    -3517.2250417379941609    -1.15e-04  4.16e-04  1.50e-02  5.19e-04     9.3
    8    -3517.2250320334546814     9.70e-06  1.36e-04  3.67e-03  6.17e-04     7.7
    9    -3517.2250597445349740    -2.77e-05  7.58e-05  1.02e-03  2.29e-04     7.3
   10    -3517.2250577847730710     1.96e-06  2.53e-05  3.60e-04  2.27e-04     7.6
   11    -3517.2250627755925052    -4.99e-06  1.36e-05  2.55e-04  3.80e-05     6.9
```

```
  12    -3517.2250626949203252     8.07e-08  6.74e-06  1.17e-04  4.49e-05     6.7
  13    -3517.2250628775359473    -1.83e-07  1.69e-06  2.87e-05  8.68e-06     7.7
  14    -3517.2250628742517620     3.28e-09  9.54e-07  1.61e-05  8.99e-06     5.7
  15    -3517.2250628797096397    -5.46e-09  3.92e-07  6.24e-06  2.05e-06     5.4
  16    -3517.2250628800152299    -3.06e-10  2.74e-07  5.11e-06  2.24e-06     5.0
               **** Energy Check signals convergence ****

             *****************************************************
             *                     SUCCESS                       *
             *           SCF CONVERGED AFTER  16 CYCLES          *
             *****************************************************

Recomputing exchange energy using gridx3       ... done (   13.408 sec)
Old exchange energy                            :    -40.859741697 Eh
New exchange energy                            :    -40.859744687 Eh
Exchange energy change after final integration :     -0.000002990 Eh
Total energy after final integration           :  -3517.225065870 Eh

SMD CDS free energy correction energy :                 -4.52381     Kcal/mol
Total Energy after SMD CDS correction =           -3517.232275018 Eh
             **** ENERGY FILE WAS UPDATED (Cl-IRC.en.tmp) ****

----------------
TOTAL SCF ENERGY
----------------

Total Energy       :      -3517.23227501756355 Eh          -95708.75594 eV

Components:
Nuclear Repulsion  :       1882.27443827222169 Eh           51219.29140 eV
Electronic Energy  :      -5399.48392638201040 Eh         -146927.42728 eV
One Electron Energy:      -8613.42268138378313 Eh         -234383.14697 eV
Two Electron Energy:       3213.93875500177228 Eh           87455.71969 eV
CPCM Dielectric    :         -0.01557477025257 Eh              -0.42381 eV
SMD CDS (Gcds)     :         -0.00720914744219 Eh              -0.19617 eV

Virial components:
Potential Energy   :      -7027.28957332598657 Eh         -191222.27084 eV
Kinetic Energy     :       3510.05729830842347 Eh           95513.51490 eV
Virial Ratio       :          2.00204411953976

DFT components:
N(Alpha)           :       81.999899878740 electrons
N(Beta)            :       81.999899878740 electrons
N(Total)           :      163.999799757481 electrons
E(X)               :     -162.489149379721 Eh
E(C)               :       -7.229056283105 Eh
E(XC)              :     -169.718205662826 Eh

CPCM Solvation Model Properties:
Surface-charge          :         -0.05086277640966
Corrected charge        :          0.00000000000000
Outlying charge corr.   :          0.00013055612309 Eh               0.00355 eV
Free-energy (cav+disp)  :         -0.00720914744219 Eh              -0.19617 eV

---------------
SCF CONVERGENCE
---------------

  Last Energy change         ...    3.0559e-10  Tolerance :   1.0000e-09
  Last MAX-Density change    ...    5.1088e-06  Tolerance :   1.0000e-08
  Last RMS-Density change    ...    2.7352e-07  Tolerance :   1.0000e-09
  Last DIIS Error            ...    2.9835e-03  Tolerance :   1.0000e-08
```

```
  Last Orbital Gradient      ...    2.2434e-06  Tolerance :   2.0000e-06
  Last Orbital Rotation      ...    1.8500e-06  Tolerance :   2.0000e-06


----------------
ORBITAL ENERGIES
----------------

  NO   OCC          E(Eh)            E(eV) 
   0   2.0000    -453.930259    -12352.0703 
   1   2.0000    -101.527310     -2762.6986 
   2   2.0000     -58.110602     -1581.2699 
   3   2.0000     -52.163048     -1419.4287 
   4   2.0000     -52.162286     -1419.4080 
   5   2.0000     -52.161213     -1419.3788 
   6   2.0000     -19.111488      -520.0500 
   7   2.0000     -10.236337      -278.5449 
   8   2.0000     -10.201504      -277.5970 
   9   2.0000     -10.186959      -277.2013 
  10   2.0000     -10.186436      -277.1870 
  11   2.0000     -10.185747      -277.1683 
  12   2.0000     -10.185097      -277.1506 
  13   2.0000     -10.184341      -277.1300 
  14   2.0000     -10.173870      -276.8451 
  15   2.0000     -10.166675      -276.6493 
  16   2.0000     -10.165798      -276.6254 
  17   2.0000     -10.165674      -276.6221 
  18   2.0000     -10.164678      -276.5949 
  19   2.0000     -10.164376      -276.5867 
  20   2.0000     -10.163639      -276.5667 
  21   2.0000     -10.155465      -276.3443 
  22   2.0000      -9.459432      -257.4042 
  23   2.0000      -7.848928      -213.5802 
  24   2.0000      -7.220715      -196.4857 
  25   2.0000      -7.212694      -196.2674 
  26   2.0000      -7.212267      -196.2558 
  27   2.0000      -5.792877      -157.6322 
  28   2.0000      -5.789042      -157.5279 
  29   2.0000      -5.785234      -157.4242 
  30   2.0000      -2.146403       -58.4066 
  31   2.0000      -2.145645       -58.3860 
  32   2.0000      -2.141883       -58.2836 
  33   2.0000      -2.138574       -58.1936 
  34   2.0000      -2.138205       -58.1835 
  35   2.0000      -0.963883       -26.2286 
  36   2.0000      -0.895359       -24.3640 
  37   2.0000      -0.855128       -23.2692 
  38   2.0000      -0.842995       -22.9391 
  39   2.0000      -0.794018       -21.6063 
  40   2.0000      -0.762281       -20.7427 
  41   2.0000      -0.748985       -20.3809 
  42   2.0000      -0.744001       -20.2453 
  43   2.0000      -0.739972       -20.1357 
  44   2.0000      -0.678988       -18.4762 
  45   2.0000      -0.656875       -17.8745 
  46   2.0000      -0.624016       -16.9803 
  47   2.0000      -0.600454       -16.3392 
  48   2.0000      -0.593997       -16.1635 
  49   2.0000      -0.584557       -15.9066 
  50   2.0000      -0.554441       -15.0871 
  51   2.0000      -0.511375       -13.9152 
  52   2.0000      -0.503051       -13.6887 
  53   2.0000      -0.479704       -13.0534 
```

```
  54   2.0000      -0.466617       -12.6973
  55   2.0000      -0.464632       -12.6433
  56   2.0000      -0.459055       -12.4915
  57   2.0000      -0.447282       -12.1712
  58   2.0000      -0.440420       -11.9844
  59   2.0000      -0.433475       -11.7955
  60   2.0000      -0.424075       -11.5397
  61   2.0000      -0.416114       -11.3230
  62   2.0000      -0.413582       -11.2541
  63   2.0000      -0.405185       -11.0257
  64   2.0000      -0.389444       -10.5973
  65   2.0000      -0.377772       -10.2797
  66   2.0000      -0.369995       -10.0681
  67   2.0000      -0.367024        -9.9872
  68   2.0000      -0.363324        -9.8865
  69   2.0000      -0.358434        -9.7535
  70   2.0000      -0.348976        -9.4961
  71   2.0000      -0.336843        -9.1660
  72   2.0000      -0.332728        -9.0540
  73   2.0000      -0.325601        -8.8600
  74   2.0000      -0.317116        -8.6292
  75   2.0000      -0.275358        -7.4929
  76   2.0000      -0.270841        -7.3700
  77   2.0000      -0.266844        -7.2612
  78   2.0000      -0.252110        -6.8603
  79   2.0000      -0.245409        -6.6779
  80   2.0000      -0.240854        -6.5540
  81   2.0000      -0.226847        -6.1728
  82   0.0000      -0.043256        -1.1771
  83   0.0000      -0.030294        -0.8243
  84   0.0000      -0.024025        -0.6538
  85   0.0000      -0.006304        -0.1715
  86   0.0000      -0.003938        -0.1072
  87   0.0000       0.011097         0.3020
  88   0.0000       0.021795         0.5931
  89   0.0000       0.040009         1.0887
  90   0.0000       0.054295         1.4774
  91   0.0000       0.064829         1.7641
  92   0.0000       0.071261         1.9391
*Only the first 10 virtual orbitals were printed (use %output print[p_orben] 2 end to see all).

                    ********************************
                    * MULLIKEN POPULATION ANALYSIS *
                    ********************************

-----------------------
MULLIKEN ATOMIC CHARGES
-----------------------
   0 C :    0.181341
   1 C :   -0.239075
   2 C :   -0.243362
   3 C :   -0.094479
   4 C :   -0.126445
   5 C :   -0.172802
   6 H :    0.125014
   7 H :    0.136442
   8 H :    0.138176
   9 H :    0.136714
  10 H :    0.135577
  11 C :   -0.178384
  12 H :    0.113368
  13 H :    0.116778
  14 C :   -0.267865
```

```
  15 H :    0.257058
  16 H :    0.127849
  17 C :   -0.300514
  18 H :    0.163359
  19 H :    0.164477
  20 Se:    0.423594
  21 O :   -0.571516
  22 C :    0.062112
  23 C :   -0.173628
  24 C :   -0.187093
  25 C :   -0.212312
  26 C :   -0.194210
  27 C :    0.221221
  28 H :    0.143503
  29 H :    0.157761
  30 H :    0.148139
  31 H :    0.148214
  32 Cl:   -0.139011
Sum of atomic charges:    0.0000000

-------------------------------
MULLIKEN REDUCED ORBITAL CHARGES
-------------------------------
  0 C s       :     3.161044  s :     3.161044
      pz      :     0.877612  p :     2.523511
      px      :     0.778940
      py      :     0.866959
      dz2     :     0.025089  d :     0.124285
      dxz     :     0.029140
      dyz     :     0.019755
      dx2y2   :     0.024062
      dxy     :     0.026239
      f0      :     0.001221  f :     0.009819
      f+1     :     0.001425
      f-1     :     0.001209
      f+2     :     0.001936
      f-2     :     0.001371
      f+3     :     0.001124
      f-3     :     0.001532

  1 C s       :     3.268829  s :     3.268829
      pz      :     0.969515  p :     2.880065
      px      :     0.974215
      py      :     0.936335
      dz2     :     0.017845  d :     0.082871
      dxz     :     0.021187
      dyz     :     0.011547
      dx2y2   :     0.012403
      dxy     :     0.019890
      f0      :     0.001154  f :     0.007310
      f+1     :     0.001127
      f-1     :     0.000612
      f+2     :     0.001266
      f-2     :     0.001322
      f+3     :     0.000958
      f-3     :     0.000869

  2 C s       :     3.272402  s :     3.272402
      pz      :     0.967946  p :     2.881528
      px      :     0.947769
      py      :     0.965812
      dz2     :     0.013446  d :     0.082155
      dxz     :     0.018748
```

```
    dyz     :     0.011156
    dx2y2   :     0.018865
    dxy     :     0.019939
    f0      :     0.000316  f :     0.007278
    f+1     :     0.001324
    f-1     :     0.001012
    f+2     :     0.001096
    f-2     :     0.001172
    f+3     :     0.001050
    f-3     :     0.001307

3 C s       :     3.188649  s :     3.188649
    pz      :     0.945884  p :     2.815519
    px      :     0.921074
    py      :     0.948560
    dz2     :     0.013337  d :     0.083026
    dxz     :     0.018796
    dyz     :     0.010566
    dx2y2   :     0.020120
    dxy     :     0.020206
    f0      :     0.000318  f :     0.007286
    f+1     :     0.001331
    f-1     :     0.001060
    f+2     :     0.001128
    f-2     :     0.001171
    f+3     :     0.001001
    f-3     :     0.001277

4 C s       :     3.211595  s :     3.211595
    pz      :     0.944228  p :     2.825183
    px      :     0.928178
    py      :     0.952777
    dz2     :     0.017492  d :     0.082406
    dxz     :     0.021427
    dyz     :     0.011254
    dx2y2   :     0.012417
    dxy     :     0.019816
    f0      :     0.001186  f :     0.007262
    f+1     :     0.001077
    f-1     :     0.000611
    f+2     :     0.001227
    f-2     :     0.001325
    f+3     :     0.000945
    f-3     :     0.000890

5 C s       :     3.237253  s :     3.237253
    pz      :     0.940744  p :     2.847313
    px      :     0.963218
    py      :     0.943351
    dz2     :     0.020316  d :     0.081007
    dxz     :     0.015591
    dyz     :     0.015427
    dx2y2   :     0.018988
    dxy     :     0.010685
    f0      :     0.001067  f :     0.007230
    f+1     :     0.000847
    f-1     :     0.001086
    f+2     :     0.001455
    f-2     :     0.001096
    f+3     :     0.000824
    f-3     :     0.000855

6 H s       :     0.852700  s :     0.852700
```

```
      pz      :     0.006276  p :     0.022286
      px      :     0.006966
      py      :     0.009043

  7 H s       :     0.841438  s :     0.841438
      pz      :     0.010443  p :     0.022120
      px      :     0.005012
      py      :     0.006665

  8 H s       :     0.839502  s :     0.839502
      pz      :     0.010702  p :     0.022321
      px      :     0.005018
      py      :     0.006601

  9 H s       :     0.841118  s :     0.841118
      pz      :     0.006213  p :     0.022168
      px      :     0.006954
      py      :     0.009001

 10 H s       :     0.842175  s :     0.842175
      pz      :     0.005182  p :     0.022249
      px      :     0.012464
      py      :     0.004602

 11 C s       :     3.232072  s :     3.232072
      pz      :     0.970435  p :     2.853985
      px      :     0.911356
      py      :     0.972194
      dz2     :     0.018714  d :     0.086587
      dxz     :     0.023456
      dyz     :     0.011334
      dx2y2   :     0.015257
      dxy     :     0.017826
      f0      :     0.000580  f :     0.005740
      f+1     :     0.000813
      f-1     :     0.000816
      f+2     :     0.001214
      f-2     :     0.000990
      f+3     :     0.000754
      f-3     :     0.000573

 12 H s       :     0.864932  s :     0.864932
      pz      :     0.005117  p :     0.021700
      px      :     0.006042
      py      :     0.010541

 13 H s       :     0.861417  s :     0.861417
      pz      :     0.009314  p :     0.021805
      px      :     0.007404
      py      :     0.005087

 14 C s       :     3.202805  s :     3.202805
      pz      :     0.983637  p :     2.963946
      px      :     0.946294
      py      :     1.034015
      dz2     :     0.026798  d :     0.094754
      dxz     :     0.014860
      dyz     :     0.017801
      dx2y2   :     0.022349
      dxy     :     0.012945
      f0      :     0.001096  f :     0.006361
      f+1     :     0.000753
      f-1     :     0.000686
```

```
     f+2     :     0.001686
     f-2     :     0.001046
     f+3     :     0.000393
     f-3     :     0.000701

15 H s       :     0.690794  s :     0.690794
     pz      :     0.008939  p :     0.052148
     px      :     0.007133
     py      :     0.036076

16 H s       :     0.849483  s :     0.849483
     pz      :     0.005755  p :     0.022668
     px      :     0.011673
     py      :     0.005240

17 C s       :     3.302661  s :     3.302661
     pz      :     1.011194  p :     2.922120
     px      :     1.000444
     py      :     0.910482
     dz2     :     0.023440  d :     0.070796
     dxz     :     0.006723
     dyz     :     0.010335
     dx2y2   :     0.014127
     dxy     :     0.016170
     f0      :     0.000717  f :     0.004936
     f+1     :     0.001169
     f-1     :     0.000568
     f+2     :     0.000398
     f-2     :     0.000920
     f+3     :     0.000371
     f-3     :     0.000794

18 H s       :     0.814300  s :     0.814300
     pz      :     0.012699  p :     0.022341
     px      :     0.004280
     py      :     0.005362

19 H s       :     0.812696  s :     0.812696
     pz      :     0.004189  p :     0.022827
     px      :     0.011975
     py      :     0.006663

20 Ses       :     7.841120  s :     7.841120
     pz      :     5.119086  p :    15.464202
     px      :     4.782579
     py      :     5.562537
     dz2     :     2.048282  d :    10.230539
     dxz     :     2.050952
     dyz     :     2.035273
     dx2y2   :     2.058365
     dxy     :     2.037666
     f0      :     0.005546  f :     0.040545
     f+1     :     0.010303
     f-1     :     0.001644
     f+2     :     0.007218
     f-2     :     0.007884
     f+3     :     0.003781
     f-3     :     0.004170

21 O s       :     3.866918  s :     3.866918
     pz      :     1.591164  p :     4.677723
     px      :     1.478796
     py      :     1.607764
```

```
     dz2     :     0.005290  d :     0.025906
     dxz     :     0.006946
     dyz     :     0.002964
     dx2y2   :     0.006172
     dxy     :     0.004533
     f0      :     0.000102  f :     0.000970
     f+1     :     0.000229
     f-1     :     0.000055
     f+2     :     0.000217
     f-2     :     0.000152
     f+3     :     0.000114
     f-3     :     0.000101

22 C s       :     3.161553  s :     3.161553
     pz      :     0.824235  p :     2.643325
     px      :     0.901878
     py      :     0.917212
     dz2     :     0.022284  d :     0.124720
     dxz     :     0.040453
     dyz     :     0.025334
     dx2y2   :     0.020158
     dxy     :     0.016491
     f0      :     0.001279  f :     0.008291
     f+1     :     0.001756
     f-1     :     0.001033
     f+2     :     0.001040
     f-2     :     0.001177
     f+3     :     0.001279
     f-3     :     0.000726

23 C s       :     3.271016  s :     3.271016
     pz      :     0.923559  p :     2.805500
     px      :     0.911327
     py      :     0.970615
     dz2     :     0.025024  d :     0.089564
     dxz     :     0.024205
     dyz     :     0.016977
     dx2y2   :     0.008218
     dxy     :     0.015140
     f0      :     0.001314  f :     0.007548
     f+1     :     0.001794
     f-1     :     0.000833
     f+2     :     0.000785
     f-2     :     0.001292
     f+3     :     0.000699
     f-3     :     0.000830

24 C s       :     3.258341  s :     3.258341
     pz      :     0.959752  p :     2.834312
     px      :     0.915926
     py      :     0.958634
     dz2     :     0.023191  d :     0.086961
     dxz     :     0.015522
     dyz     :     0.010123
     dx2y2   :     0.019642
     dxy     :     0.018484
     f0      :     0.000729  f :     0.007479
     f+1     :     0.001511
     f-1     :     0.000823
     f+2     :     0.000923
     f-2     :     0.001232
     f+3     :     0.001379
     f-3     :     0.000881
```

```
25 C s       :     3.294872  s :     3.294872
     pz      :     0.943484  p :     2.827112
     px      :     0.924881
     py      :     0.958747
     dz2     :     0.020874  d :     0.082839
     dxz     :     0.015879
     dyz     :     0.009101
     dx2y2   :     0.018568
     dxy     :     0.018416
     f0      :     0.000790  f :     0.007490
     f+1     :     0.001463
     f-1     :     0.000779
     f+2     :     0.000932
     f-2     :     0.001181
     f+3     :     0.001427
     f-3     :     0.000917

26 C s       :     3.270879  s :     3.270879
     pz      :     0.923772  p :     2.832659
     px      :     0.965980
     py      :     0.942907
     dz2     :     0.025565  d :     0.083158
     dxz     :     0.019014
     dyz     :     0.016265
     dx2y2   :     0.008444
     dxy     :     0.013870
     f0      :     0.001344  f :     0.007514
     f+1     :     0.001808
     f-1     :     0.000804
     f+2     :     0.000797
     f-2     :     0.001236
     f+3     :     0.000703
     f-3     :     0.000822

27 C s       :     3.070175  s :     3.070175
     pz      :     0.804448  p :     2.548971
     px      :     0.804414
     py      :     0.940109
     dz2     :     0.032529  d :     0.149132
     dxz     :     0.041715
     dyz     :     0.027328
     dx2y2   :     0.026380
     dxy     :     0.021180
     f0      :     0.001425  f :     0.010501
     f+1     :     0.002505
     f-1     :     0.001143
     f+2     :     0.001492
     f-2     :     0.001504
     f+3     :     0.001499
     f-3     :     0.000934

28 H s       :     0.833876  s :     0.833876
     pz      :     0.005143  p :     0.022621
     px      :     0.010278
     py      :     0.007200

29 H s       :     0.819860  s :     0.819860
     pz      :     0.013062  p :     0.022379
     px      :     0.003739
     py      :     0.005578

30 H s       :     0.829401  s :     0.829401
```

```
     pz      :     0.013285  p :     0.022460
     px      :     0.003686
     py      :     0.005490

 31 H s      :     0.829299  s :     0.829299
     pz      :     0.005095  p :     0.022487
     px      :     0.009910
     py      :     0.007481

 32 Cls      :     5.884277  s :     5.884277
     pz      :     3.749504  p :    11.188505
     px      :     3.532603
     py      :     3.906399
     dz2     :     0.007687  d :     0.060777
     dxz     :     0.020250
     dyz     :     0.007647
     dx2y2   :     0.013936
     dxy     :     0.011256
     f0      :     0.000609  f :     0.005452
     f+1     :     0.001041
     f-1     :     0.000139
     f+2     :     0.001377
     f-2     :     0.000975
     f+3     :     0.000696
     f-3     :     0.000616


                     *******************************
                     * LOEWDIN POPULATION ANALYSIS *
                     *******************************

----------------------
LOEWDIN ATOMIC CHARGES
----------------------
   0 C :   -0.148282
   1 C :   -0.115213
   2 C :   -0.123565
   3 C :   -0.141554
   4 C :   -0.152780
   5 C :   -0.154042
   6 H :    0.150855
   7 H :    0.151686
   8 H :    0.148795
   9 H :    0.147808
  10 H :    0.146877
  11 C :   -0.209772
  12 H :    0.148483
  13 H :    0.146162
  14 C :   -0.311595
  15 H :    0.187820
  16 H :    0.144813
  17 C :   -0.227609
  18 H :    0.125871
  19 H :    0.128739
  20 Se:    0.650790
  21 O :   -0.408441
  22 C :   -0.321534
  23 C :   -0.128615
  24 C :   -0.131341
  25 C :   -0.138621
  26 C :   -0.137945
  27 C :   -0.418419
```

```
  28 H :    0.154696
  29 H :    0.157318
  30 H :    0.155830
  31 H :    0.155961
  32 Cl:    0.366826

-------------------------------
LOEWDIN REDUCED ORBITAL CHARGES
-------------------------------
  0 C s       :     2.711027  s :     2.711027
      pz      :     0.963680  p :     2.860521
      px      :     0.963615
      py      :     0.933226
      dz2     :     0.101968  d :     0.519008
      dxz     :     0.118560
      dyz     :     0.086252
      dx2y2   :     0.110257
      dxy     :     0.101971
      f0      :     0.006460  f :     0.057726
      f+1     :     0.009933
      f-1     :     0.007446
      f+2     :     0.010431
      f-2     :     0.007208
      f+3     :     0.006483
      f-3     :     0.009765

  1 C s       :     2.759647  s :     2.759647
      pz      :     0.977137  p :     2.929128
      px      :     1.014401
      py      :     0.937590
      dz2     :     0.070844  d :     0.383675
      dxz     :     0.107330
      dyz     :     0.057420
      dx2y2   :     0.061012
      dxy     :     0.087070
      f0      :     0.006174  f :     0.042763
      f+1     :     0.007119
      f-1     :     0.004877
      f+2     :     0.007831
      f-2     :     0.005112
      f+3     :     0.005429
      f-3     :     0.006222

  2 C s       :     2.760250  s :     2.760250
      pz      :     0.953759  p :     2.936359
      px      :     1.027002
      py      :     0.955598
      dz2     :     0.059452  d :     0.384333
      dxz     :     0.088031
      dyz     :     0.056619
      dx2y2   :     0.086304
      dxy     :     0.093927
      f0      :     0.002687  f :     0.042624
      f+1     :     0.008154
      f-1     :     0.004856
      f+2     :     0.007293
      f-2     :     0.005369
      f+3     :     0.005280
      f-3     :     0.008985

  3 C s       :     2.765642  s :     2.765642
      pz      :     0.955059  p :     2.943769
      px      :     1.034431
```

```
    py      :     0.954279
    dz2     :     0.061461  d :     0.388663
    dxz     :     0.088402
    dyz     :     0.056143
    dx2y2   :     0.087751
    dxy     :     0.094906
    f0      :     0.002686  f :     0.043481
    f+1     :     0.008303
    f-1     :     0.005126
    f+2     :     0.007497
    f-2     :     0.005490
    f+3     :     0.005303
    f-3     :     0.009074

4 C s       :     2.766359  s :     2.766359
    pz      :     0.990486  p :     2.954094
    px      :     1.020927
    py      :     0.942681
    dz2     :     0.073805  d :     0.389045
    dxz     :     0.108517
    dyz     :     0.058281
    dx2y2   :     0.059925
    dxy     :     0.088517
    f0      :     0.006448  f :     0.043283
    f+1     :     0.007058
    f-1     :     0.005004
    f+2     :     0.007918
    f-2     :     0.005124
    f+3     :     0.005471
    f-3     :     0.006259

5 C s       :     2.766723  s :     2.766723
    pz      :     0.999758  p :     2.957232
    px      :     0.981081
    py      :     0.976393
    dz2     :     0.086188  d :     0.386646
    dxz     :     0.079822
    dyz     :     0.077534
    dx2y2   :     0.087415
    dxy     :     0.055687
    f0      :     0.005581  f :     0.043441
    f+1     :     0.006613
    f-1     :     0.006777
    f+2     :     0.007679
    f-2     :     0.006085
    f+3     :     0.004679
    f-3     :     0.006027

6 H s       :     0.789844  s :     0.789844
    pz      :     0.016901  p :     0.059301
    px      :     0.018599
    py      :     0.023800

7 H s       :     0.789143  s :     0.789143
    pz      :     0.027125  p :     0.059171
    px      :     0.014088
    py      :     0.017958

8 H s       :     0.791930  s :     0.791930
    pz      :     0.027136  p :     0.059275
    px      :     0.014198
    py      :     0.017941
```

```
 9 H s       :     0.793134  s :     0.793134
     pz      :     0.016952  p :     0.059059
     px      :     0.018653
     py      :     0.023455

10 H s       :     0.793741  s :     0.793741
     pz      :     0.014529  p :     0.059383
     px      :     0.031646
     py      :     0.013208

11 C s       :     2.766182  s :     2.766182
     pz      :     1.009957  p :     3.016962
     px      :     0.995637
     py      :     1.011368
     dz2     :     0.071124  d :     0.388561
     dxz     :     0.097209
     dyz     :     0.066865
     dx2y2   :     0.075308
     dxy     :     0.078055
     f0      :     0.005313  f :     0.038066
     f+1     :     0.006773
     f-1     :     0.004531
     f+2     :     0.005133
     f-2     :     0.005634
     f+3     :     0.005916
     f-3     :     0.004765

12 H s       :     0.793093  s :     0.793093
     pz      :     0.015264  p :     0.058424
     px      :     0.015305
     py      :     0.027854

13 H s       :     0.795675  s :     0.795675
     pz      :     0.024462  p :     0.058163
     px      :     0.018523
     py      :     0.015178

14 C s       :     2.787451  s :     2.787451
     pz      :     1.038654  p :     3.069988
     px      :     1.035934
     py      :     0.995401
     dz2     :     0.092148  d :     0.412422
     dxz     :     0.095601
     dyz     :     0.083163
     dx2y2   :     0.097527
     dxy     :     0.043985
     f0      :     0.008500  f :     0.041734
     f+1     :     0.006483
     f-1     :     0.004523
     f+2     :     0.007010
     f-2     :     0.006251
     f+3     :     0.004481
     f-3     :     0.004485

15 H s       :     0.653319  s :     0.653319
     pz      :     0.022899  p :     0.158861
     px      :     0.017646
     py      :     0.118316

16 H s       :     0.793200  s :     0.793200
     pz      :     0.017156  p :     0.061987
     px      :     0.029475
     py      :     0.015356
```

```
17 C s       :     2.871814  s :     2.871814
     pz      :     1.049832  p :     3.024165
     px      :     1.074634
     py      :     0.899699
     dz2     :     0.103512  d :     0.303097
     dxz     :     0.049887
     dyz     :     0.028483
     dx2y2   :     0.053312
     dxy     :     0.067903
     f0      :     0.003331  f :     0.028533
     f+1     :     0.006525
     f-1     :     0.002631
     f+2     :     0.004065
     f-2     :     0.005735
     f+3     :     0.002370
     f-3     :     0.003876

18 H s       :     0.809224  s :     0.809224
     pz      :     0.035291  p :     0.064905
     px      :     0.014219
     py      :     0.015395

19 H s       :     0.804515  s :     0.804515
     pz      :     0.013387  p :     0.066746
     px      :     0.034445
     py      :     0.018915

20 Ses       :     7.327600  s :     7.327600
     pz      :     5.141204  p :    15.370884
     px      :     4.786895
     py      :     5.442785
     dz2     :     2.098522  d :    10.543450
     dxz     :     2.157368
     dyz     :     2.073649
     dx2y2   :     2.134903
     dxy     :     2.079007
     f0      :     0.013757  f :     0.107276
     f+1     :     0.028578
     f-1     :     0.003608
     f+2     :     0.023554
     f-2     :     0.018532
     f+3     :     0.009459
     f-3     :     0.009790

21 O s       :     3.625958  s :     3.625958
     pz      :     1.636481  p :     4.718710
     px      :     1.525156
     py      :     1.557073
     dz2     :     0.010637  d :     0.060961
     dxz     :     0.020192
     dyz     :     0.005315
     dx2y2   :     0.012292
     dxy     :     0.012524
     f0      :     0.000232  f :     0.002813
     f+1     :     0.000608
     f-1     :     0.000209
     f+2     :     0.000635
     f-2     :     0.000411
     f+3     :     0.000356
     f-3     :     0.000361

22 C s       :     2.784489  s :     2.784489
```

```
     pz       :     1.049794  p :     3.000059
     px       :     1.002485
     py       :     0.947779
     dz2      :     0.098102  d :     0.487585
     dxz      :     0.156289
     dyz      :     0.091333
     dx2y2    :     0.072231
     dxy      :     0.069631
     f0       :     0.007841  f :     0.049402
     f+1      :     0.011907
     f-1      :     0.007281
     f+2      :     0.004810
     f-2      :     0.007233
     f+3      :     0.005107
     f-3      :     0.005223

 23 C s       :     2.764848  s :     2.764848
     pz       :     1.030233  p :     2.928262
     px       :     0.992124
     py       :     0.905905
     dz2      :     0.107071  d :     0.391200
     dxz      :     0.120201
     dyz      :     0.072099
     dx2y2    :     0.032361
     dxy      :     0.059468
     f0       :     0.007515  f :     0.044305
     f+1      :     0.012253
     f-1      :     0.005797
     f+2      :     0.005556
     f-2      :     0.005845
     f+3      :     0.002914
     f-3      :     0.004426

 24 C s       :     2.763890  s :     2.763890
     pz       :     0.983972  p :     2.932074
     px       :     1.029171
     py       :     0.918930
     dz2      :     0.099801  d :     0.391262
     dxz      :     0.084365
     dyz      :     0.046834
     dx2y2    :     0.082482
     dxy      :     0.077779
     f0       :     0.003752  f :     0.044115
     f+1      :     0.010848
     f-1      :     0.003899
     f+2      :     0.006608
     f-2      :     0.007478
     f+3      :     0.004996
     f-3      :     0.006534

 25 C s       :     2.767614  s :     2.767614
     pz       :     0.988085  p :     2.944739
     px       :     1.029152
     py       :     0.927502
     dz2      :     0.097325  d :     0.382301
     dxz      :     0.085229
     dyz      :     0.044084
     dx2y2    :     0.078294
     dxy      :     0.077369
     f0       :     0.003960  f :     0.043967
     f+1      :     0.010377
     f-1      :     0.003670
     f+2      :     0.006682
```

```
     f-2     :     0.007509
     f+3     :     0.005261
     f-3     :     0.006508

26 C s       :     2.767423  s :     2.767423
     pz      :     1.029514  p :     2.944584
     px      :     0.996860
     py      :     0.918210
     dz2     :     0.108922  d :     0.381980
     dxz     :     0.109852
     dyz     :     0.071548
     dx2y2   :     0.032852
     dxy     :     0.058807
     f0      :     0.007855  f :     0.043957
     f+1     :     0.011716
     f-1     :     0.005964
     f+2     :     0.005605
     f-2     :     0.005523
     f+3     :     0.002886
     f-3     :     0.004408

27 C s       :     2.781063  s :     2.781063
     pz      :     1.035809  p :     2.965665
     px      :     0.960597
     py      :     0.969259
     dz2     :     0.131572  d :     0.608265
     dxz     :     0.183579
     dyz     :     0.103533
     dx2y2   :     0.102640
     dxy     :     0.086941
     f0      :     0.008779  f :     0.063426
     f+1     :     0.016706
     f-1     :     0.007909
     f+2     :     0.007598
     f-2     :     0.009240
     f+3     :     0.006409
     f-3     :     0.006785

28 H s       :     0.784741  s :     0.784741
     pz      :     0.014397  p :     0.060563
     px      :     0.026827
     py      :     0.019340

29 H s       :     0.782797  s :     0.782797
     pz      :     0.033484  p :     0.059885
     px      :     0.010838
     py      :     0.015562

30 H s       :     0.783744  s :     0.783744
     pz      :     0.034119  p :     0.060426
     px      :     0.010908
     py      :     0.015399

31 H s       :     0.783561  s :     0.783561
     pz      :     0.014294  p :     0.060477
     px      :     0.025817
     py      :     0.020367

32 Cls       :     5.554629  s :     5.554629
     pz      :     3.665592  p :    10.969116
     px      :     3.480387
     py      :     3.823137
     dz2     :     0.014511  d :     0.098317
```

```
     dxz     :     0.031402
     dyz     :     0.011904
     dx2y2   :     0.021864
     dxy     :     0.018635
     f0      :     0.001136  f :     0.011112
     f+1     :     0.002464
     f-1     :     0.000289
     f+2     :     0.002533
     f-2     :     0.001998
     f+3     :     0.001402
     f-3     :     0.001290


                       ****************************
                       * MAYER POPULATION ANALYSIS *
                       ****************************

  NA   - Mulliken gross atomic population
  ZA   - Total nuclear charge
  QA   - Mulliken gross atomic charge
  VA   - Mayer's total valence
  BVA  - Mayer's bonded valence
  FA   - Mayer's free valence

  ATOM       NA         ZA         QA         VA         BVA        FA
  0 C      5.8187     6.0000     0.1813     3.5702     3.5702    -0.0000
  1 C      6.2391     6.0000    -0.2391     3.9266     3.9266    -0.0000
  2 C      6.2434     6.0000    -0.2434     3.8743     3.8743    -0.0000
  3 C      6.0945     6.0000    -0.0945     3.8243     3.8243     0.0000
  4 C      6.1264     6.0000    -0.1264     3.8275     3.8275     0.0000
  5 C      6.1728     6.0000    -0.1728     3.8422     3.8422     0.0000
  6 H      0.8750     1.0000     0.1250     0.9826     0.9826    -0.0000
  7 H      0.8636     1.0000     0.1364     0.9689     0.9689    -0.0000
  8 H      0.8618     1.0000     0.1382     0.9698     0.9698     0.0000
  9 H      0.8633     1.0000     0.1367     0.9648     0.9648     0.0000
 10 H      0.8644     1.0000     0.1356     0.9724     0.9724    -0.0000
 11 C      6.1784     6.0000    -0.1784     3.8438     3.8438     0.0000
 12 H      0.8866     1.0000     0.1134     0.9604     0.9604    -0.0000
 13 H      0.8832     1.0000     0.1168     0.9641     0.9641     0.0000
 14 C      6.2679     6.0000    -0.2679     3.7547     3.7547    -0.0000
 15 H      0.7429     1.0000     0.2571     1.0148     1.0148     0.0000
 16 H      0.8722     1.0000     0.1278     0.9630     0.9630    -0.0000
 17 C      6.3005     6.0000    -0.3005     3.8945     3.8945    -0.0000
 18 H      0.8366     1.0000     0.1634     0.9599     0.9599     0.0000
 19 H      0.8355     1.0000     0.1645     0.9621     0.9621    -0.0000
 20 Se    33.5764    34.0000     0.4236     2.9505     2.9505     0.0000
 21 O      8.5715     8.0000    -0.5715     1.8214     1.8214    -0.0000
 22 C      5.9379     6.0000     0.0621     3.6657     3.6657     0.0000
 23 C      6.1736     6.0000    -0.1736     3.8103     3.8103     0.0000
 24 C      6.1871     6.0000    -0.1871     3.8453     3.8453    -0.0000
 25 C      6.2123     6.0000    -0.2123     3.9327     3.9327     0.0000
 26 C      6.1942     6.0000    -0.1942     3.9019     3.9019    -0.0000
 27 C      5.7788     6.0000     0.2212     3.6333     3.6333     0.0000
 28 H      0.8565     1.0000     0.1435     0.9932     0.9932     0.0000
 29 H      0.8422     1.0000     0.1578     0.9633     0.9633    -0.0000
 30 H      0.8519     1.0000     0.1481     0.9773     0.9773     0.0000
 31 H      0.8518     1.0000     0.1482     0.9778     0.9778     0.0000
 32 Cl    17.1390    17.0000    -0.1390     1.1804     1.1804    -0.0000

  Mayer bond orders larger than 0.100000
B(  0-C ,  1-C ) :   1.3448 B(  0-C ,  2-C ) :   1.3251 B(  0-C , 11-C ) :   0.9092
B(  1-C ,  3-C ) :   1.4008 B(  1-C ,  6-H ) :   0.9795 B(  2-C ,  4-C ) :   1.3937
```

```
B(  2-C ,  7-H ) :   0.9926 B(  3-C ,  5-C ) :   1.3702 B(  3-C ,  8-H ) :   0.9698
B(  4-C ,  5-C ) :   1.3680 B(  4-C ,  9-H ) :   0.9834 B(  5-C , 10-H ) :   0.9795
B( 11-C , 12-H ) :   0.9503 B( 11-C , 13-H ) :   0.9794 B( 11-C , 14-C ) :   0.9554
B( 14-C , 15-H ) :   0.5447 B( 14-C , 16-H ) :   0.9421 B( 14-C , 17-C ) :   1.2983
B( 15-H , 21-O ) :   0.3945 B( 17-C , 18-H ) :   0.9566 B( 17-C , 19-H ) :   0.9523
B( 17-C , 20-Se) :   0.5017 B( 20-Se, 21-O ) :   1.2658 B( 20-Se, 22-C ) :   0.9620
B( 22-C , 23-C ) :   1.3007 B( 22-C , 24-C ) :   1.3312 B( 23-C , 25-C ) :   1.3750
B( 23-C , 28-H ) :   0.9731 B( 24-C , 26-C ) :   1.3726 B( 24-C , 29-H ) :   0.9724
B( 25-C , 27-C ) :   1.3161 B( 25-C , 30-H ) :   0.9869 B( 26-C , 27-C ) :   1.3259
B( 26-C , 31-H ) :   0.9811 B( 27-C , 32-Cl) :   0.9101

-------
TIMINGS
-------

Total SCF time: 0 days 0 hours 2 min 6 sec

Total time                  ....     126.594 sec
Sum of individual times     ....     122.035 sec  ( 96.4%)

SCF preparation             ....       0.776 sec  (  0.6%)
Fock matrix formation       ....     116.372 sec  ( 91.9%)
  Startup                   ....       0.052 sec  (  0.0% of F)
  Split-RI-J                ....       8.716 sec  (  7.5% of F)
  Chain of spheres X        ....      84.367 sec  ( 72.5% of F)
  XC integration            ....      12.746 sec  ( 11.0% of F)
    XC Preparation          ....       0.000 sec  (  0.0% of XC)
    Basis function eval.    ....       3.056 sec  ( 24.0% of XC)
    Density eval.           ....       4.119 sec  ( 32.3% of XC)
    XC-Functional eval.     ....       0.112 sec  (  0.9% of XC)
    XC-Potential eval.      ....       3.941 sec  ( 30.9% of XC)
  CPCM terms                ....      13.353 sec  ( 11.5% of F)
Diagonalization             ....       0.000 sec  (  0.0%)
Density matrix formation    ....       0.555 sec  (  0.4%)
Total Energy calculation    ....       0.236 sec  (  0.2%)
Population analysis         ....       0.159 sec  (  0.1%)
Orbital Transformation      ....       0.445 sec  (  0.4%)
Orbital Orthonormalization  ....       0.000 sec  (  0.0%)
DIIS solution               ....       1.594 sec  (  1.3%)
SOSCF solution              ....       1.898 sec  (  1.5%)
Finished LeanSCF after 126.9 sec

Maximum memory used throughout the entire LEANSCF-calculation: 97.8 MB


-------------------------------------------------------------------------------
                          DFT DISPERSION CORRECTION

                              DFTD3 V3.1  Rev 1
                          USING Becke-Johnson damping
-------------------------------------------------------------------------------
The B3LYP functional is recognized
Active option DFTDOPT                   ...         4

molecular C6(AA) [au] = 16148.189105


            DFT-D V3
 parameters
 s6 scaling factor         :     1.0000
 a1 scaling factor         :     0.3981
 s8 scaling factor         :     1.9889
 a2 scaling factor         :     4.4211
```

```
 ad hoc parameters k1-k3   :    16.0000     1.3333    -4.0000

 Edisp/kcal,au: -54.125738228731  -0.086254845054
 E6   /kcal   : -24.818091289
 E8   /kcal   : -29.307646940

-------------------------   ----------------
Dispersion correction           -0.086254845
-------------------------   ----------------


-------------------------   --------------------
FINAL SINGLE POINT ENERGY     -3517.318529862617
-------------------------   --------------------



           ************************************************************
           *        Program running with 12 parallel MPI-processes    *
           *              working on a common directory               *
           ************************************************************
------------------------------------------------------------------------------
                         ORCA SCF GRADIENT CALCULATION
------------------------------------------------------------------------------

Nuc. rep. gradient        (SHARK) ... done (  0.0 sec)
HCore & Overlap gradient  (SHARK) ... done (  0.4 sec)
Split-RIJ-J gradient      (SHARK) ... done (  2.1 sec)
COSX-gradient                     ... done ( 42.6 sec)
XC gradient                       ... done (  6.5 sec)
CPCM gradient                     ... done (  2.1 sec)
SMD gradient                      ... done (  0.0 sec)
Dispersion correction             ... done (  0.0 sec)

-------------------
DISPERSION GRADIENT
-------------------

   1   C   :   -0.000286461    0.000917066    0.000493272
   2   C   :   -0.000280431    0.000988927    0.000994097
   3   C   :    0.000159472    0.000879606    0.000228225
   4   C   :   -0.000316192    0.001026715    0.000821905
   5   C   :    0.000168817    0.000783239    0.000118951
   6   C   :   -0.000075443    0.000976723    0.000371472
   7   H   :   -0.000242345    0.000086892    0.000538223
   8   H   :    0.000033774    0.000432404   -0.000170048
   9   H   :   -0.000107074    0.000238745    0.000504573
  10   H   :    0.000162692    0.000289622   -0.000111938
  11   H   :    0.000185108    0.000361147    0.000171075
  12   C   :   -0.000690350    0.000408101    0.000020270
  13   H   :   -0.000362174    0.000243141    0.000011336
  14   H   :   -0.000407332    0.000113265    0.000189175
  15   C   :   -0.000762710   -0.000367534   -0.000178283
  16   H   :   -0.000063989   -0.000912446   -0.000152391
  17   H   :   -0.000358707    0.000077480   -0.000162789
  18   C   :   -0.000055234   -0.001655955   -0.000577122
  19   H   :   -0.000072011    0.000109604   -0.000298907
  20   H   :   -0.000187754    0.000107715   -0.000642250
  21   Se  :   -0.000960811   -0.000275598   -0.001620001
  22   O   :   -0.000914602    0.000390538   -0.000293729
  23   C   :    0.000700914   -0.000987389   -0.000182715
  24   C   :    0.000385236   -0.000836936   -0.000076967
  25   C   :    0.000629177   -0.000748827   -0.000373362
```

```
  26   C   :    0.000559481   -0.000616760    0.000166474
  27   C   :    0.000758645   -0.000510091   -0.000166996
  28   C   :    0.000503013   -0.000594362   -0.000098207
  29   H   :   -0.000039068   -0.000382434    0.000273709
  30   H   :    0.000352128   -0.000007033   -0.000346391
  31   H   :    0.000006820   -0.000286365    0.000284075
  32   H   :    0.000307207    0.000042241   -0.000265078
  33   Cl  :    0.001270205   -0.000291444    0.000530343

Difference to translation invariance:
           :    0.0000000000    0.0000000000   -0.0000000000

Difference to rotation invariance:
           :    0.0000000000   -0.0000000000    0.0000000000

Norm of the Dispersion gradient    ...    0.0053335285
RMS gradient                       ...    0.0005360398
MAX gradient                       ...    0.0016559551

------------------
CARTESIAN GRADIENT
------------------

   1   C   :   -0.000017828    0.000018025    0.000031701
   2   C   :    0.000012586   -0.000017417   -0.000003207
   3   C   :   -0.000005745    0.000008712    0.000008880
   4   C   :    0.000003869   -0.000007112   -0.000001600
   5   C   :    0.000000772   -0.000001491    0.000001365
   6   C   :    0.000003441   -0.000005721   -0.000000070
   7   H   :    0.000055788    0.000086743    0.000099360
   8   H   :   -0.000003789   -0.000000946    0.000005116
   9   H   :    0.000012244   -0.000030592    0.000002628
  10   H   :   -0.000001543    0.000000019    0.000002197
  11   H   :   -0.000000627    0.000003056    0.000001840
  12   C   :   -0.000002260   -0.000003166    0.000013168
  13   H   :    0.000014335   -0.000004916   -0.000021150
  14   H   :   -0.000009198    0.000025063    0.000009618
  15   C   :   -0.000003726   -0.000009613    0.000018154
  16   H   :   -0.000054315   -0.000010961   -0.000058202
  17   H   :   -0.000005620   -0.000003374    0.000012259
  18   C   :    0.000003315   -0.000003206    0.000004476
  19   H   :    0.000009011    0.000008597   -0.000002188
  20   H   :   -0.000005860    0.000008225    0.000006226
  21   Se  :   -0.000028735   -0.000012407   -0.000003881
  22   O   :    0.000016023   -0.000085473   -0.000067784
  23   C   :   -0.000000922    0.000010841   -0.000005987
  24   C   :    0.000009275   -0.000015382   -0.000007630
  25   C   :   -0.000006039    0.000009407   -0.000001622
  26   C   :    0.000000643   -0.000004263   -0.000004052
  27   C   :    0.000000688    0.000001505   -0.000002128
  28   C   :    0.000002931    0.000004843   -0.000009438
  29   H   :    0.000003108   -0.000000530   -0.000003528
  30   H   :   -0.000002862    0.000005649   -0.000002136
  31   H   :   -0.000000256    0.000023887   -0.000006032
  32   H   :   -0.000000186    0.000002366   -0.000005852
  33   Cl  :    0.000001482   -0.000000367   -0.000010501

Difference to translation invariance:
           :   -0.0000000003   -0.0000000012    0.0000000000

Difference to rotation invariance:
           :    0.0005062107    0.0006706219    0.0001184655
```

```
Norm of the Cartesian gradient     ...    0.0002190720
RMS gradient                       ...    0.0000220176
MAX gradient                       ...    0.0000993601

-------
TIMINGS
-------

Total SCF gradient time     ....       53.848 sec

Densities                   ....        0.007 sec  (  0.0%)
One electron gradient       ....        0.444 sec  (  0.8%)
RI-J Coulomb gradient       ....        2.114 sec  (  3.9%)
COSX gradient               ....       42.620 sec  ( 79.1%)
XC gradient                 ....        6.489 sec  ( 12.1%)
CPCM gradient               ....        2.127 sec  (  4.0%)
   A-Matrix (El+Nuc)        ....        0.022 sec  (  0.0%)
   Potential                ....        2.105 sec  (  3.9%)
SMD gradient                ....        0.004 sec  (  0.0%)

Maximum memory used throughout the entire SCFGRAD-calculation: 113.5 MB


           ************************************************************
           *        Program running with 12 parallel MPI-processes    *
           *              working on a common directory               *
           ************************************************************

------------------------------------------------------------------------------
                              ORCA PROPERTY CALCULATIONS
------------------------------------------------------------------------------

GBWName                                 ... Cl-IRC.gbw
Number of atoms                         ...     33
Number of basis functions               ...    671
Max core memory                         ...   8000 MB

Electric properties:
Dipole moment                           ... YES
Quadrupole moment                       ...  NO
General moments                         ...  NO (MaxL=10)
Static polarizability (Dipole/Dipole)   ...  NO
Static polarizability (Dipole/Quad.)    ...  NO
Static polarizability (Quad./Quad.)     ...  NO
Static polarizability (Velocity)        ...  NO
Static hyperpolarizability              ...  NO

Atomic electric properties:
Dipole moment                           ...  NO
Quadrupole moment                       ...  NO
Static polarizability                   ...  NO

Choice of electric origin               ... Center of mass
Position of electric origin             ...    -0.544556    -3.397335   -12.355635

General magnetic properties:
Magnetizability                         ...  NO

EPR properties:
g-Tensor (aka g-matrix)                 ...  NO
Zero-Field splitting spin-orbit         ...  NO
Zero-field splitting spin-spin          ...  NO
Hyperfine couplings                     ...  NO (   0 nuclei)
```

```
Quadrupole couplings                    ...  NO (   0 nuclei)
Contact density                         ...  NO (   0 nuclei)

NMR properties:
Chemical shifts                         ...  NO (   0 nuclei)
Spin-rotation constants                 ...  NO (   0 nuclei)
Spin-spin couplings                     ...  NO (   0 nuclei,    0 pairs)

Choice of magnetic origin               ... GIAO
Position of magnetic origin             ...     0.000000     0.000000     0.000000

Properties with geometric perturbations:
SCF Hessian                             ...  NO
IR spectrum                             ...  NO
Raman spectrum                          ...  NO
Raman option                            ... 0
VCD spectrum                            ...  NO
X-ray spectroscopy properties:
SCF XES/XAS/RIXS spectra                ...  NO

SCF SOC stabilization energy            ...  NO
Diagonal Born-Oppenheimer correction    ...  NO

-------------
DIPOLE MOMENT
-------------

Method             : SCF
Type of density    : Electron Density
Multiplicity       :   1
Irrep              :   0
Energy             : -3517.2322750175635520 Eh
Basis              : AO
                                X                 Y                 Z
Electronic contribution:      2.523085913     -31.073871279     -14.150595367
Nuclear contribution   :     -2.244539192      31.688696562      12.979878510
                        -----------------------------------------
Total Dipole Moment    :      0.278546722       0.614825283      -1.170716857
                        -----------------------------------------
Magnitude (a.u.)       :      1.351360930
Magnitude (Debye)      :      3.434886509



--------------------
Rotational spectrum
--------------------

Rotational constants in cm-1:            0.013899           0.008674           0.006197
Rotational constants in MHz :          416.671717         260.031715         185.774154

Dipole components along the rotational axes:
x,y,z [a.u.] :    -0.290268    -0.254486    -1.295051
x,y,z [Debye]:    -0.737803    -0.646852    -3.291759


Dipole moment calculation done in   0.0 sec

Maximum memory used throughout the entire PROP-calculation: 57.7 MB


--------------------------------------------------------------------------------
```

```
                   Intrinsic Reaction Coordinate Calculation
--------------------------------------------------------------------------------

IRC Settings:
  Nr. of atoms                         .... 33
  Algorithm                            .... LQA
  Coordinate system following type     .... Mass-Weighted
  Max. no of cycles (MaxIter)          .... 241
  Direction                            .... Forward and backward
  Steep Lenght                         .... 0.110 bohr Dalton^(1/2)

Initial displacement:
  Hessian type                         .... Read
  Hessian Filename                     .... TS-Cl_Compound_2.hess
  Following Hessian mode               .... 0
  Initial displacement type            .... Length
    Scaling normalized TS mode by      .... 0.040

Convergence Tolerances:
  Energy Change                        ....  5.0000e-06 Eh
  Max. Gradient                        ....  8.0000e-05 Eh/bohr
  RMS Gradient                         ....  1.0000e-05 Eh/bohr
  Max. Displacement                    ....  4.0000e-03 bohr
  RMS Displacement                     ....  2.0000e-03 bohr
  Strict Convergency criteria enforced

LQA Settings:
  Hessian update type                  .... Bofill
  Update steep lenght                  .... NO
  t Stepsize                           .... Gradient-based adjusted
  t MAX int. Steps                     .... 1000000
  Bulirsch-Stoer Correction            .... YES
    Euler Initial Steps                .... 100
    Richardson Max extrapolations      .... 20
    Richardson error threshould        ....  1.0000e-06
    Apply BS correction on TS disp.    .... NO
    Keep LQA direction                 .... NO
    LQA direction Theshould            ....  1.0000e-01
    Update Hessian and Gradient        .... YES

Storing full IRC trajectory in         .... Cl-IRC_IRC_Full_trj.xyz
Storing forward trajectory in          .... Cl-IRC_IRC_F_trj.xyz
Storing backward trajectory in         .... Cl-IRC_IRC_B_trj.xyz
Storing IRC profile in                 .... Cl-IRC.IRC.out


         *************************************************************
         *                          FORWARD IRC                      *
         *************************************************************

Iteration    E(Eh)      dE(kcal/mol)  max(|G|)   RMS(G)   max(|S|)   RMS(S)   MW-Displ.
Convergence thresholds    0.003138    0.000080  0.000010  0.004000  0.002000
    0     -3517.318571   -0.025918    0.002245  0.000500  0.035577  0.004020  0.046619
    1     -3517.318955   -0.266539    0.007377  0.001456  0.076165  0.008903  0.110004
    2     -3517.319544   -0.636202    0.011483  0.002121  0.062197  0.007993  0.110001
    3     -3517.320209   -1.053527    0.014195  0.002539  0.044172  0.006909  0.110000
    4     -3517.320906   -1.491049    0.015873  0.002808  0.030394  0.006110  0.110002
    5     -3517.321633   -1.947048    0.017135  0.002993  0.028963  0.005591  0.110003
    6     -3517.322373   -2.411849    0.018535  0.003130  0.027567  0.005253  0.110000
    7     -3517.323132   -2.887616    0.019708  0.003239  0.026342  0.005026  0.110002
    8     -3517.323904   -3.372357    0.020719  0.003330  0.025264  0.004864  0.110002
    9     -3517.324687   -3.863786    0.021609  0.003410  0.024321  0.004744  0.110003
   10     -3517.325482   -4.362374    0.022405  0.003479  0.023462  0.004651  0.110003
```

11	-3517.326284	-4.866084	0.023120	0.003540	0.022652	0.004575	0.110000
12	-3517.327094	-5.373984	0.023769	0.003589	0.021967	0.004519	0.110002
13	-3517.327910	-5.886176	0.024357	0.003639	0.021379	0.004477	0.110002
14	-3517.328731	-6.401100	0.024882	0.003683	0.020808	0.004439	0.110000
15	-3517.329559	-6.920802	0.025345	0.003721	0.020316	0.004410	0.110002
16	-3517.330394	-7.445015	0.025746	0.003753	0.019889	0.004389	0.110001
17	-3517.331227	-7.967706	0.026076	0.003778	0.019524	0.004374	0.110002
18	-3517.332068	-8.495019	0.026337	0.003794	0.019198	0.004363	0.110001
19	-3517.332906	-9.021117	0.026523	0.003803	0.018930	0.004357	0.110001
20	-3517.333745	-9.547673	0.026632	0.003804	0.018706	0.004354	0.110000
21	-3517.334590	-10.077703	0.026665	0.003795	0.018517	0.004354	0.110001
22	-3517.335424	-10.600985	0.026612	0.003776	0.018366	0.004356	0.110003
23	-3517.336239	-11.112912	0.026469	0.003745	0.018150	0.004357	0.110004
24	-3517.337055	-11.624668	0.026247	0.003704	0.017820	0.004353	0.110000
25	-3517.337857	-12.127988	0.025941	0.003653	0.017441	0.004344	0.110002
26	-3517.338641	-12.619767	0.025554	0.003593	0.016992	0.004325	0.110001
27	-3517.339401	-13.096727	0.025082	0.003524	0.016527	0.004283	0.110001
28	-3517.340138	-13.559321	0.024515	0.003443	0.016221	0.004236	0.110002
29	-3517.340854	-14.008822	0.023864	0.003350	0.016010	0.004217	0.110000
30	-3517.341548	-14.443938	0.023132	0.003248	0.015697	0.004205	0.110002
31	-3517.342216	-14.862946	0.022321	0.003137	0.015292	0.004182	0.110001
32	-3517.342855	-15.264263	0.021434	0.003018	0.014915	0.004152	0.110002
33	-3517.343466	-15.647340	0.020473	0.002890	0.014569	0.004123	0.110000
34	-3517.344046	-16.011438	0.019443	0.002755	0.014234	0.004091	0.110001
35	-3517.344595	-16.355812	0.018348	0.002612	0.014368	0.004057	0.110001
36	-3517.345111	-16.679992	0.017195	0.002464	0.014532	0.004020	0.110001
37	-3517.345594	-16.983217	0.015989	0.002311	0.014726	0.003982	0.110002
38	-3517.346046	-17.266328	0.014740	0.002154	0.014949	0.003944	0.110003
39	-3517.346463	-17.528303	0.013460	0.001995	0.015196	0.003906	0.110000
40	-3517.346848	-17.770078	0.012161	0.001834	0.015455	0.003870	0.110000
41	-3517.347201	-17.991482	0.010861	0.001678	0.015731	0.003842	0.110000
42	-3517.347526	-18.195014	0.009574	0.001518	0.015974	0.003821	0.110001
43	-3517.347816	-18.377489	0.008336	0.001386	0.016244	0.003814	0.110001
44	-3517.348092	-18.550618	0.007126	0.001209	0.016311	0.003846	0.110000
45	-3517.348320	-18.693737	0.006313	0.001179	0.016813	0.003967	0.110001
46	-3517.348565	-18.847261	0.005835	0.000943	0.016012	0.004137	0.110000
47	-3517.348759	-18.969263	0.005346	0.001074	0.017884	0.004803	0.110001
48	-3517.348973	-19.103123	0.004909	0.000773	0.015493	0.004188	0.110002
49	-3517.349145	-19.210972	0.004394	0.000822	0.019419	0.004923	0.110002
50	-3517.349333	-19.329254	0.003992	0.000605	0.018530	0.004742	0.110002
51	-3517.349500	-19.434013	0.003605	0.000723	0.024482	0.005662	0.110000
52	-3517.349660	-19.534133	0.003306	0.000702	0.020926	0.005321	0.110000
53	-3517.349820	-19.635035	0.003275	0.000667	0.022243	0.005536	0.110000
54	-3517.349981	-19.736127	0.003593	0.000661	0.023253	0.005759	0.110001
55	-3517.350131	-19.830281	0.003734	0.000655	0.023770	0.005823	0.110001
56	-3517.350287	-19.927760	0.003530	0.000618	0.024153	0.005835	0.110001
57	-3517.350444	-20.026260	0.003246	0.000577	0.024560	0.006015	0.110001
58	-3517.350601	-20.125049	0.002983	0.000543	0.024889	0.006234	0.110000
59	-3517.350762	-20.225724	0.002696	0.000513	0.025986	0.006423	0.110001
60	-3517.350924	-20.327346	0.002267	0.000483	0.026491	0.006557	0.110001
61	-3517.351084	-20.428269	0.001906	0.000470	0.026224	0.006600	0.110001
62	-3517.351238	-20.524319	0.002028	0.000517	0.025480	0.006503	0.110000
63	-3517.351391	-20.620413	0.002024	0.000520	0.025960	0.006392	0.110000
64	-3517.351545	-20.717382	0.001914	0.000512	0.026219	0.006400	0.110001
65	-3517.351700	-20.814322	0.001877	0.000510	0.025727	0.006386	0.110001
66	-3517.351853	-20.910437	0.001942	0.000515	0.025199	0.006329	0.110001
67	-3517.352004	-21.005556	0.001999	0.000519	0.024896	0.006265	0.110001
68	-3517.352155	-21.099761	0.002031	0.000521	0.024751	0.006223	0.110000
69	-3517.352303	-21.192861	0.002055	0.000522	0.024593	0.006192	0.110000
70	-3517.352449	-21.284758	0.002082	0.000521	0.024396	0.006154	0.110000
71	-3517.352593	-21.374931	0.002148	0.000526	0.024037	0.006097	0.110001
72	-3517.352735	-21.463989	0.002151	0.000522	0.023985	0.006058	0.110000
73	-3517.352874	-21.551299	0.002206	0.000525	0.023665	0.006016	0.110000

74	-3517.353011	-21.637421	0.002202	0.000519	0.023646	0.005994	0.110001
75	-3517.353146	-21.721989	0.002221	0.000518	0.023486	0.005972	0.110000
76	-3517.353278	-21.804981	0.002255	0.000517	0.023275	0.005944	0.110000
77	-3517.353408	-21.886427	0.002280	0.000516	0.023126	0.005915	0.110001
78	-3517.353536	-21.966360	0.002302	0.000514	0.022966	0.005888	0.110001
79	-3517.353660	-22.044746	0.002324	0.000513	0.022792	0.005863	0.110000
80	-3517.353783	-22.121529	0.002344	0.000511	0.022601	0.005836	0.110000
81	-3517.353902	-22.196261	0.002361	0.000509	0.022598	0.005806	0.110000
82	-3517.354019	-22.269764	0.002371	0.000506	0.022658	0.005777	0.110001
83	-3517.354133	-22.341523	0.002384	0.000504	0.022689	0.005742	0.110000
84	-3517.354245	-22.411533	0.002388	0.000502	0.022673	0.005703	0.110000
85	-3517.354353	-22.479600	0.002389	0.000499	0.022633	0.005661	0.110000
86	-3517.354459	-22.546042	0.002378	0.000496	0.022554	0.005615	0.110000
87	-3517.354562	-22.610384	0.002360	0.000493	0.022441	0.005562	0.110000
88	-3517.354661	-22.672789	0.002314	0.000489	0.022293	0.005506	0.110000
89	-3517.354757	-22.733112	0.002246	0.000485	0.022134	0.005447	0.110000
90	-3517.354853	-22.793128	0.002125	0.000479	0.021943	0.005381	0.110000
91	-3517.354943	-22.849525	0.001964	0.000479	0.021635	0.005312	0.110000
92	-3517.355032	-22.905223	0.001621	0.000457	0.021430	0.005231	0.110000
93	-3517.355115	-22.957551	0.001860	0.000474	0.021162	0.005184	0.110000
94	-3517.355210	-23.017119	0.001738	0.000436	0.020718	0.005000	0.110001
95	-3517.355260	-23.048629	0.002111	0.000510	0.020296	0.005167	0.110000
96	-3517.355342	-23.100070	0.001348	0.000358	0.019799	0.004694	0.110000
97	-3517.355413	-23.144719	0.001426	0.000384	0.022374	0.005386	0.110000
98	-3517.355493	-23.194784	0.001342	0.000295	0.025487	0.005826	0.110000
99	-3517.355544	-23.226699	0.003569	0.000607	0.025760	0.006481	0.110000
100	-3517.355613	-23.270140	0.002067	0.000419	0.017613	0.004375	0.110001
101	-3517.355668	-23.304513	0.001847	0.000442	0.016305	0.004881	0.110000
102	-3517.355716	-23.334593	0.002593	0.000497	0.018129	0.005112	0.110000
103	-3517.355763	-23.364083	0.002476	0.000477	0.015849	0.004576	0.110000
104	-3517.355804	-23.389527	0.002625	0.000508	0.015827	0.004393	0.110000
105	-3517.355811	-23.394357	0.002821	0.000514	0.011660	0.002248	0.110000
106	-3517.355867	-23.429572	0.001065	0.000273	0.017658	0.004279	0.110001
107	-3517.355918	-23.461164	0.001152	0.000256	0.020671	0.005155	0.110000
108	-3517.355960	-23.487672	0.001640	0.000308	0.019738	0.004770	0.110000
109	-3517.356002	-23.514316	0.001609	0.000303	0.017932	0.004482	0.110000
110	-3517.356043	-23.539578	0.001534	0.000299	0.017612	0.004429	0.110000
111	-3517.356081	-23.563704	0.001478	0.000299	0.016687	0.004340	0.110000
112	-3517.356118	-23.586887	0.001424	0.000303	0.016319	0.004301	0.110000
113	-3517.356154	-23.609204	0.001337	0.000310	0.015874	0.004270	0.110000
114	-3517.356188	-23.631027	0.001203	0.000320	0.015864	0.004280	0.110000
115	-3517.356220	-23.650628	0.001495	0.000337	0.015689	0.004288	0.110000
116	-3517.356255	-23.672665	0.001673	0.000350	0.015643	0.004308	0.110000
117	-3517.356289	-23.693931	0.001035	0.000263	0.011611	0.003888	0.110000
118	-3517.356319	-23.712849	0.001063	0.000292	0.016690	0.004588	0.110000
119	-3517.356349	-23.731899	0.001412	0.000299	0.014302	0.004281	0.110000
120	-3517.356379	-23.750871	0.001504	0.000315	0.014513	0.004253	0.110000
121	-3517.356407	-23.767985	0.001211	0.000324	0.012649	0.004017	0.110000
122	-3517.356435	-23.785744	0.001678	0.000344	0.012053	0.003921	0.110000
123	-3517.356467	-23.805932	0.001319	0.000272	0.011109	0.003632	0.110000
124	-3517.356492	-23.821804	0.001263	0.000299	0.013390	0.004150	0.110000
125	-3517.356522	-23.840160	0.001445	0.000268	0.011836	0.003874	0.110000
126	-3517.356548	-23.856441	0.001444	0.000294	0.014753	0.004084	0.110000
127	-3517.356572	-23.871670	0.001224	0.000314	0.014453	0.004066	0.110000
128	-3517.356602	-23.890350	0.001740	0.000343	0.015660	0.004120	0.110000
129	-3517.356634	-23.910599	0.001576	0.000321	0.013988	0.004131	0.110000
130	-3517.356637	-23.912344	0.001537	0.000294	0.013871	0.002248	0.110000
131	-3517.356664	-23.929202	0.000844	0.000215	0.011276	0.003806	0.110000
132	-3517.356686	-23.943240	0.001256	0.000326	0.013184	0.004228	0.110000
133	-3517.356712	-23.959737	0.001123	0.000241	0.014447	0.003757	0.110000
134	-3517.356741	-23.977692	0.000665	0.000136	0.016170	0.004245	0.110000
135	-3517.356761	-23.990053	0.001589	0.000342	0.014619	0.004436	0.110000
136	-3517.356790	-24.008826	0.000675	0.000135	0.009578	0.003394	0.110000

137	-3517.356813	-24.023228	0.001148	0.000294	0.015396	0.004711	0.110000
138	-3517.356834	-24.036288	0.000903	0.000217	0.009761	0.003443	0.110001
139	-3517.356855	-24.049459	0.000789	0.000221	0.008870	0.003614	0.110000
140	-3517.356883	-24.066667	0.000723	0.000210	0.013916	0.004324	0.110001
141	-3517.356908	-24.082605	0.000831	0.000249	0.014026	0.004223	0.110000
142	-3517.356929	-24.095600	0.000976	0.000289	0.014101	0.004040	0.110000
143	-3517.356959	-24.114725	0.001055	0.000291	0.014136	0.003985	0.110000
144	-3517.356983	-24.129739	0.001118	0.000292	0.013109	0.003873	0.110000
145	-3517.357008	-24.145234	0.001159	0.000294	0.013401	0.003889	0.110000
146	-3517.357033	-24.160829	0.001173	0.000297	0.012982	0.003860	0.110000
147	-3517.357058	-24.176459	0.001135	0.000293	0.012485	0.003897	0.110000
148	-3517.357084	-24.192990	0.001066	0.000288	0.012313	0.003855	0.110000
149	-3517.357109	-24.208759	0.000921	0.000278	0.012124	0.003887	0.110000
150	-3517.357133	-24.223602	0.001049	0.000268	0.012413	0.003888	0.110000
151	-3517.357159	-24.240214	0.001166	0.000263	0.013187	0.003995	0.110000
152	-3517.357182	-24.254281	0.001210	0.000267	0.012569	0.003947	0.110000
153	-3517.357206	-24.269550	0.001168	0.000268	0.012867	0.004003	0.110000
154	-3517.357230	-24.284605	0.001138	0.000275	0.012059	0.003931	0.110000
155	-3517.357253	-24.299341	0.001129	0.000275	0.013781	0.004072	0.110000
156	-3517.357278	-24.314646	0.001149	0.000267	0.012254	0.003958	0.110000
157	-3517.357302	-24.329557	0.001186	0.000264	0.012461	0.003915	0.110000
158	-3517.357327	-24.345702	0.001193	0.000266	0.012877	0.003933	0.110000
159	-3517.357351	-24.360331	0.001144	0.000270	0.012665	0.003872	0.110000
160	-3517.357374	-24.374752	0.001072	0.000269	0.013896	0.003993	0.110000
161	-3517.357398	-24.389768	0.001059	0.000265	0.012539	0.003895	0.110000
162	-3517.357421	-24.404332	0.001124	0.000267	0.012568	0.003819	0.110000
163	-3517.357442	-24.417644	0.001156	0.000264	0.012445	0.003841	0.110000
164	-3517.357467	-24.433250	0.001193	0.000266	0.012222	0.003862	0.110000
165	-3517.357487	-24.445748	0.001191	0.000268	0.013894	0.004013	0.110000
166	-3517.357510	-24.460287	0.001170	0.000262	0.012089	0.003941	0.110000
167	-3517.357534	-24.475669	0.001203	0.000267	0.012824	0.003919	0.110000
168	-3517.357557	-24.489730	0.001182	0.000265	0.011946	0.003870	0.110000
169	-3517.357577	-24.502313	0.001136	0.000261	0.012227	0.003899	0.110000
170	-3517.357601	-24.517350	0.001156	0.000266	0.012955	0.003952	0.110000
171	-3517.357623	-24.531175	0.001158	0.000262	0.012084	0.003884	0.110000
172	-3517.357645	-24.544945	0.001140	0.000260	0.012241	0.003911	0.110000
173	-3517.357664	-24.556844	0.001150	0.000256	0.011569	0.003875	0.110000
174	-3517.357688	-24.571764	0.001172	0.000264	0.013440	0.004015	0.110000
175	-3517.357709	-24.584985	0.001218	0.000266	0.011746	0.003892	0.110000
176	-3517.357727	-24.596708	0.001140	0.000254	0.012325	0.003945	0.110000
177	-3517.357749	-24.610427	0.001172	0.000265	0.013592	0.004144	0.110000
178	-3517.357770	-24.623596	0.001104	0.000242	0.012337	0.003973	0.110000
179	-3517.357786	-24.633841	0.001125	0.000258	0.012704	0.004055	0.110000
180	-3517.357809	-24.648241	0.001117	0.000254	0.013883	0.004075	0.110000
181	-3517.357828	-24.660131	0.001223	0.000265	0.012093	0.003978	0.110000
182	-3517.357849	-24.673342	0.001099	0.000244	0.012341	0.003976	0.110000
183	-3517.357868	-24.685074	0.001179	0.000263	0.011770	0.004013	0.110000
184	-3517.357887	-24.696806	0.001043	0.000236	0.013124	0.004017	0.110000
185	-3517.357902	-24.706170	0.001161	0.000266	0.013079	0.004122	0.110000
186	-3517.357922	-24.718999	0.001140	0.000256	0.013912	0.004109	0.110000
187	-3517.357941	-24.731069	0.001247	0.000275	0.012993	0.004099	0.110000
188	-3517.357961	-24.743129	0.001224	0.000258	0.011055	0.003936	0.110000
189	-3517.357980	-24.755179	0.001171	0.000269	0.012734	0.004090	0.110000
190	-3517.357999	-24.767379	0.001191	0.000263	0.013205	0.004059	0.110000
191	-3517.358018	-24.779202	0.001192	0.000270	0.013950	0.004123	0.110000
192	-3517.358037	-24.791249	0.001232	0.000269	0.013322	0.004079	0.110000
193	-3517.358056	-24.802989	0.001243	0.000273	0.013013	0.004080	0.110000
194	-3517.358075	-24.814902	0.001248	0.000270	0.012424	0.004044	0.110000
195	-3517.358094	-24.826535	0.001236	0.000272	0.012420	0.004053	0.110000
196	-3517.358112	-24.838331	0.001216	0.000268	0.012488	0.004050	0.110000
197	-3517.358131	-24.849887	0.001211	0.000271	0.012634	0.004068	0.110000
198	-3517.358149	-24.861509	0.001199	0.000266	0.012588	0.004060	0.110000
199	-3517.358167	-24.872795	0.001212	0.000271	0.012459	0.004068	0.110000

```
  200     -3517.358185   -24.884233    0.001192  0.000265  0.012231  0.004048  0.110000
  201     -3517.358203   -24.895205    0.001201  0.000272  0.012059  0.004058  0.110000
  202     -3517.358221   -24.906464    0.001152  0.000259  0.012079  0.004044  0.110000
  203     -3517.358238   -24.917100    0.001172  0.000272  0.012080  0.004067  0.110000
  204     -3517.358256   -24.928290    0.001111  0.000252  0.012199  0.004048  0.110000
  205     -3517.358272   -24.938529    0.001181  0.000276  0.011704  0.004074  0.110000
  206     -3517.358290   -24.949791    0.001077  0.000242  0.011836  0.004039  0.110000
  207     -3517.358305   -24.959436    0.001190  0.000273  0.010825  0.004067  0.110000
  208     -3517.358324   -24.971132    0.000997  0.000235  0.012267  0.004084  0.110000
  209     -3517.358341   -24.981644    0.001166  0.000260  0.011096  0.004146  0.110000
  210     -3517.358363   -24.995615    0.001139  0.000254  0.010867  0.004051  0.110000
  211     -3517.358365   -24.997180    0.001167  0.000242  0.012049  0.002213  0.110000
  212     -3517.358381   -25.006944    0.000904  0.000197  0.012601  0.004123  0.110000
  213     -3517.358399   -25.018517    0.000840  0.000175  0.014281  0.004630  0.110000
  214     -3517.358412   -25.026615    0.000881  0.000217  0.012241  0.004246  0.110000
  215     -3517.358426   -25.035399    0.001447  0.000277  0.012361  0.004212  0.110000
  216     -3517.358430   -25.037743    0.001499  0.000279  0.015490  0.002597  0.110000
  217     -3517.358449   -25.049547    0.000923  0.000169  0.010481  0.003465  0.110000
  218     -3517.358461   -25.057305    0.001446  0.000291  0.012708  0.004510  0.110000
  219     -3517.358472   -25.063779    0.000795  0.000212  0.011684  0.003905  0.110000
  220     -3517.358458   -25.055498    0.002214  0.000500  0.012386  0.004201  0.110000
  221     -3517.358461   -25.057144    0.002436  0.000522  0.017357  0.004489  0.110000
  222     -3517.358465   -25.059903    0.001868  0.000480  0.009960  0.004024  0.110000
  223     -3517.358486   -25.072805    0.000825  0.000205  0.011631  0.004267  0.110000
  224     -3517.358504   -25.083992    0.000545  0.000120  0.012165  0.004668  0.110000
  225     -3517.358520   -25.094253    0.000518  0.000128  0.012773  0.004679  0.110000
  226     -3517.358536   -25.104146    0.000529  0.000141  0.012663  0.004672  0.110000
  227     -3517.358551   -25.113667    0.000607  0.000154  0.012402  0.004643  0.110000
  228     -3517.358566   -25.122714    0.000718  0.000167  0.012138  0.004625  0.110000
  229     -3517.358580   -25.131976    0.000795  0.000173  0.011860  0.004604  0.110000
  230     -3517.358594   -25.140470    0.000801  0.000169  0.011947  0.004583  0.110000
  231     -3517.358609   -25.149983    0.000756  0.000160  0.011881  0.004585  0.110000
  232     -3517.358623   -25.158682    0.000695  0.000152  0.011876  0.004593  0.110000
  233     -3517.358636   -25.167167    0.000648  0.000151  0.011774  0.004592  0.110000
  234     -3517.358650   -25.175886    0.000622  0.000153  0.011577  0.004587  0.110000
  235     -3517.358663   -25.183986    0.000616  0.000155  0.011883  0.004566  0.110000
  236     -3517.358676   -25.191825    0.000639  0.000161  0.011718  0.004538  0.110000
  237     -3517.358688   -25.199466    0.000667  0.000167  0.011531  0.004516  0.110000
  238     -3517.358699   -25.206742    0.000690  0.000172  0.011577  0.004505  0.110000
  239     -3517.358711   -25.214155    0.000705  0.000174  0.011615  0.004498  0.110000
  240     -3517.358723   -25.221387    0.000702  0.000173  0.011651  0.004489  0.110000

         *************************************************************
         *  MAXIMUM NUMBER OF ITERATIONS REACHED - STOPPING IRC RUN  *
         *************************************************************


         *************************************************************
         *                        BACKWARD IRC                       *
         *************************************************************

Iteration     E(Eh)      dE(kcal/mol)  max(|G|)   RMS(G)   max(|S|)   RMS(S)   MW-Displ.
Convergence thresholds     0.003138    0.000080  0.000010  0.004000  0.002000
    0     -3517.318581   -0.032093    0.002382  0.000553  0.035577  0.004020  0.046619
    1     -3517.319239   -0.445276    0.008383  0.002103  0.087753  0.009792  0.110006
    2     -3517.320836   -1.447315    0.015427  0.003731  0.090700  0.010001  0.110006
    3     -3517.323100   -2.867870    0.020517  0.004804  0.089603  0.009924  0.110001
    4     -3517.325402   -4.312205    0.024444  0.005300  0.082890  0.009438  0.110004
    5     -3517.327344   -5.531192    0.026823  0.005408  0.065322  0.008243  0.110004
    6     -3517.328930   -6.526275    0.026996  0.005274  0.037787  0.006637  0.110004
    7     -3517.330325   -7.401577    0.025875  0.005041  0.025236  0.005711  0.110002
    8     -3517.331591   -8.195981    0.024462  0.004797  0.021172  0.005194  0.110002
    9     -3517.332758   -8.927962    0.024635  0.004572  0.020237  0.004846  0.110004
```

10	-3517.333844	-9.609728	0.024672	0.004369	0.020197	0.004614	0.110004
11	-3517.334868	-10.252170	0.024595	0.004188	0.019979	0.004467	0.110003
12	-3517.335833	-10.857836	0.024421	0.004024	0.019920	0.004384	0.110002
13	-3517.336757	-11.437372	0.024167	0.003876	0.020715	0.004343	0.110000
14	-3517.337638	-11.990595	0.023851	0.003739	0.021140	0.004329	0.110000
15	-3517.338484	-12.521248	0.023485	0.003612	0.021291	0.004331	0.110002
16	-3517.339297	-13.031516	0.023082	0.003491	0.021264	0.004339	0.110003
17	-3517.340076	-13.520527	0.022653	0.003376	0.021118	0.004353	0.110003
18	-3517.340829	-13.992742	0.022206	0.003266	0.020894	0.004369	0.110002
19	-3517.341556	-14.449223	0.021748	0.003160	0.020619	0.004387	0.110003
20	-3517.342254	-14.887386	0.021282	0.003060	0.020317	0.004404	0.110001
21	-3517.342928	-15.309717	0.020811	0.002963	0.020006	0.004420	0.110001
22	-3517.343576	-15.716807	0.020341	0.002872	0.019695	0.004436	0.110000
23	-3517.344200	-16.107917	0.019877	0.002787	0.019381	0.004451	0.110003
24	-3517.344797	-16.483092	0.019430	0.002715	0.019029	0.004461	0.110001
25	-3517.345360	-16.836334	0.019012	0.002672	0.018518	0.004435	0.110002
26	-3517.345897	-17.172968	0.018581	0.002615	0.017796	0.004313	0.110002
27	-3517.346409	-17.494395	0.018134	0.002538	0.017319	0.004256	0.110002
28	-3517.346903	-17.804062	0.017687	0.002466	0.017113	0.004254	0.110001
29	-3517.347377	-18.101581	0.017233	0.002397	0.017260	0.004243	0.110003
30	-3517.347832	-18.387284	0.016803	0.002331	0.017371	0.004223	0.110002
31	-3517.348270	-18.661882	0.016386	0.002267	0.017463	0.004205	0.110002
32	-3517.348690	-18.925822	0.015976	0.002202	0.017543	0.004187	0.110000
33	-3517.349095	-19.179660	0.015569	0.002142	0.017625	0.004173	0.110002
34	-3517.349483	-19.423439	0.015175	0.002082	0.017665	0.004161	0.110000
35	-3517.349857	-19.658240	0.014783	0.002024	0.017716	0.004136	0.110000
36	-3517.350217	-19.883676	0.014399	0.001970	0.017745	0.004116	0.110001
37	-3517.350562	-20.100577	0.014022	0.001914	0.017749	0.004092	0.110001
38	-3517.350894	-20.308837	0.013652	0.001863	0.017755	0.004071	0.110001
39	-3517.351214	-20.509333	0.013289	0.001811	0.017764	0.004047	0.110001
40	-3517.351520	-20.701696	0.012931	0.001763	0.017754	0.004025	0.110000
41	-3517.351816	-20.887109	0.012579	0.001712	0.017745	0.004000	0.110002
42	-3517.352099	-21.064767	0.012237	0.001670	0.017743	0.003983	0.110001
43	-3517.352372	-21.236442	0.011901	0.001619	0.017718	0.003957	0.110001
44	-3517.352633	-21.400218	0.011572	0.001585	0.017670	0.003940	0.110000
45	-3517.352888	-21.559719	0.011249	0.001528	0.017631	0.003911	0.110001
46	-3517.353127	-21.709967	0.010936	0.001511	0.017554	0.003898	0.110001
47	-3517.353366	-21.859679	0.010624	0.001433	0.017516	0.003865	0.110001
48	-3517.353583	-21.995820	0.010322	0.001456	0.017354	0.003865	0.110000
49	-3517.353812	-22.139817	0.010017	0.001321	0.017312	0.003829	0.110001
50	-3517.354017	-22.268724	0.009702	0.001332	0.017538	0.003939	0.110001
51	-3517.354226	-22.399652	0.009432	0.001292	0.017685	0.003860	0.110001
52	-3517.354415	-22.518149	0.009165	0.001301	0.017036	0.003782	0.110001
53	-3517.354609	-22.639702	0.008898	0.001206	0.017026	0.003744	0.110001
54	-3517.354778	-22.745988	0.008630	0.001259	0.016834	0.003751	0.110000
55	-3517.354965	-22.863041	0.008365	0.001106	0.016795	0.003723	0.110000
56	-3517.355132	-22.968316	0.008073	0.001096	0.017225	0.003858	0.110001
57	-3517.355287	-23.065626	0.007840	0.001168	0.017257	0.003813	0.110000
58	-3517.355454	-23.170375	0.007608	0.001021	0.016346	0.003678	0.110001
59	-3517.355600	-23.262124	0.007338	0.001030	0.016891	0.003783	0.110001
60	-3517.355744	-23.351953	0.007128	0.001064	0.017144	0.003726	0.110000
61	-3517.355886	-23.441136	0.006921	0.000991	0.015988	0.003602	0.110001
62	-3517.356011	-23.519650	0.006699	0.001041	0.016033	0.003588	0.110000
63	-3517.356148	-23.605772	0.006500	0.000919	0.015853	0.003566	0.110001
64	-3517.356266	-23.679526	0.006276	0.000980	0.015729	0.003600	0.110000
65	-3517.356392	-23.758749	0.006098	0.000892	0.015805	0.003570	0.110001
66	-3517.356500	-23.826352	0.005900	0.000961	0.015265	0.003542	0.110000
67	-3517.356620	-23.901987	0.005733	0.000842	0.015234	0.003519	0.110001
68	-3517.356723	-23.966210	0.005534	0.000905	0.015048	0.003543	0.110000
69	-3517.356832	-24.034973	0.005383	0.000834	0.015103	0.003514	0.110001
70	-3517.356927	-24.094283	0.005212	0.000894	0.014501	0.003475	0.110000
71	-3517.357031	-24.160074	0.005071	0.000792	0.014377	0.003453	0.110001
72	-3517.357120	-24.215731	0.004897	0.000858	0.014194	0.003465	0.110000

73	-3517.357153	-24.236162	0.004992	0.000917	0.019571	0.002470	0.110000
74	-3517.357257	-24.301562	0.004656	0.000566	0.008553	0.003050	0.110004
75	-3517.357358	-24.364747	0.004465	0.000549	0.015836	0.004023	0.110001
76	-3517.357454	-24.424994	0.004301	0.000548	0.016070	0.003929	0.110001
77	-3517.357545	-24.482457	0.004155	0.000539	0.015331	0.003858	0.110000
78	-3517.357634	-24.538333	0.004020	0.000523	0.015660	0.003871	0.110000
79	-3517.357720	-24.592224	0.003888	0.000508	0.015985	0.003885	0.110000
80	-3517.357803	-24.644332	0.003759	0.000495	0.016006	0.003883	0.110000
81	-3517.357883	-24.694390	0.003635	0.000485	0.015757	0.003861	0.110000
82	-3517.357960	-24.742706	0.003515	0.000474	0.015445	0.003835	0.110000
83	-3517.358034	-24.789202	0.003400	0.000463	0.015180	0.003817	0.110000
84	-3517.358106	-24.834037	0.003288	0.000453	0.014955	0.003801	0.110000
85	-3517.358174	-24.877241	0.003180	0.000443	0.014752	0.003787	0.110001
86	-3517.358241	-24.918926	0.003076	0.000434	0.014564	0.003775	0.110000
87	-3517.358305	-24.959058	0.002974	0.000425	0.014381	0.003766	0.110000
88	-3517.358366	-24.997681	0.002873	0.000416	0.014236	0.003754	0.110000
89	-3517.358425	-25.034653	0.002773	0.000408	0.014140	0.003743	0.110000
90	-3517.358482	-25.070515	0.002683	0.000400	0.013927	0.003726	0.110000
91	-3517.358537	-25.105083	0.002596	0.000393	0.013684	0.003715	0.110000
92	-3517.358591	-25.138461	0.002511	0.000389	0.013295	0.003709	0.110000
93	-3517.358642	-25.170781	0.002431	0.000380	0.013041	0.003688	0.110000
94	-3517.358692	-25.201820	0.002352	0.000375	0.012806	0.003686	0.110000
95	-3517.358740	-25.232075	0.002277	0.000367	0.012599	0.003674	0.110000
96	-3517.358786	-25.261062	0.002202	0.000362	0.012371	0.003673	0.110000
97	-3517.358831	-25.289296	0.002131	0.000354	0.012197	0.003663	0.110000
98	-3517.358874	-25.316378	0.002061	0.000348	0.012262	0.003659	0.110000
99	-3517.358918	-25.343827	0.001993	0.000346	0.011821	0.003671	0.110000
100	-3517.358956	-25.368013	0.001927	0.000335	0.011849	0.003647	0.110000
101	-3517.358996	-25.392653	0.001862	0.000333	0.011821	0.003667	0.110000
102	-3517.359033	-25.416199	0.001798	0.000326	0.011521	0.003652	0.110000
103	-3517.359070	-25.439548	0.001738	0.000325	0.011189	0.003678	0.110000
104	-3517.359107	-25.462468	0.001674	0.000314	0.011173	0.003638	0.110000
105	-3517.359141	-25.483839	0.001624	0.000316	0.010884	0.003710	0.110000
106	-3517.359175	-25.505315	0.001546	0.000305	0.010116	0.003611	0.110000
107	-3517.359206	-25.524611	0.001538	0.000290	0.010799	0.003755	0.110000
108	-3517.359237	-25.543824	0.001494	0.000302	0.011231	0.003605	0.110000
109	-3517.359268	-25.563209	0.001420	0.000279	0.011085	0.003765	0.110000
110	-3517.359291	-25.577662	0.001550	0.000288	0.011154	0.003718	0.110000
111	-3517.359324	-25.598824	0.001405	0.000268	0.011179	0.003652	0.110000
112	-3517.359352	-25.616437	0.001546	0.000280	0.011804	0.003808	0.110000
113	-3517.359381	-25.634165	0.001328	0.000252	0.011366	0.003668	0.110000
114	-3517.359406	-25.650129	0.001468	0.000268	0.012740	0.003904	0.110000
115	-3517.359432	-25.666326	0.001341	0.000250	0.011677	0.003725	0.110000
116	-3517.359456	-25.681574	0.001425	0.000257	0.012291	0.003883	0.110000
117	-3517.359480	-25.696630	0.001328	0.000243	0.011907	0.003748	0.110000
118	-3517.359503	-25.711031	0.001382	0.000247	0.011873	0.003871	0.110000
119	-3517.359526	-25.725660	0.001217	0.000223	0.011926	0.003751	0.110000
120	-3517.359547	-25.738868	0.001325	0.000238	0.012659	0.003991	0.110000
121	-3517.359568	-25.751983	0.001261	0.000228	0.012063	0.003758	0.110000
122	-3517.359589	-25.764952	0.001241	0.000227	0.011870	0.003807	0.110000
123	-3517.359610	-25.778144	0.000966	0.000195	0.011187	0.003622	0.110000
124	-3517.359628	-25.789194	0.000950	0.000224	0.013014	0.004076	0.110000
125	-3517.359645	-25.800174	0.001241	0.000238	0.011477	0.003724	0.110000
126	-3517.359652	-25.804290	0.001141	0.000208	0.008873	0.002184	0.110000
127	-3517.359669	-25.815253	0.000817	0.000156	0.015468	0.004350	0.110000
128	-3517.359686	-25.826119	0.001541	0.000232	0.016696	0.004404	0.110000
129	-3517.359701	-25.835099	0.001422	0.000266	0.012964	0.003812	0.110000
130	-3517.359716	-25.844415	0.000646	0.000118	0.009516	0.003402	0.110000
131	-3517.359729	-25.852975	0.001320	0.000250	0.014215	0.004312	0.110000
132	-3517.359748	-25.864954	0.000639	0.000091	0.014359	0.003620	0.110000
133	-3517.359761	-25.873073	0.001223	0.000214	0.017101	0.004379	0.110000
134	-3517.359760	-25.872042	0.001725	0.000313	0.011146	0.003517	0.110000
135	-3517.359760	-25.872481	0.002646	0.000416	0.016768	0.003940	0.110000

136	-3517.359769	-25.877682	0.002045	0.000359	0.014879	0.003898	0.110000
137	-3517.359785	-25.887853	0.001128	0.000197	0.011748	0.003433	0.110000
138	-3517.359798	-25.895861	0.000564	0.000112	0.012120	0.004032	0.110000
139	-3517.359814	-25.905829	0.000555	0.000098	0.014297	0.004320	0.110000
140	-3517.359825	-25.913078	0.000534	0.000085	0.013763	0.004248	0.110000
141	-3517.359838	-25.921309	0.000510	0.000074	0.013550	0.004231	0.110000
142	-3517.359856	-25.932651	0.000489	0.000068	0.013411	0.004255	0.110000
143	-3517.359860	-25.935004	0.000519	0.000099	0.013158	0.004031	0.110000
144	-3517.359872	-25.942418	0.000446	0.000097	0.012569	0.004179	0.110000
145	-3517.359881	-25.948213	0.000417	0.000083	0.012550	0.004199	0.110000
146	-3517.359891	-25.954142	0.000396	0.000069	0.013167	0.004258	0.110000
147	-3517.359900	-25.960117	0.000371	0.000058	0.013612	0.004310	0.110000
148	-3517.359913	-25.968480	0.000347	0.000050	0.013848	0.004339	0.110000
149	-3517.359920	-25.972391	0.000332	0.000047	0.014314	0.004351	0.110000
150	-3517.359928	-25.977647	0.000311	0.000047	0.014132	0.004315	0.110000
151	-3517.359936	-25.982588	0.000296	0.000050	0.014267	0.004291	0.110000
152	-3517.359943	-25.987152	0.000281	0.000058	0.014294	0.004253	0.110000
153	-3517.359950	-25.991206	0.000328	0.000069	0.014295	0.004209	0.110000
154	-3517.359956	-25.995260	0.000418	0.000079	0.013850	0.004120	0.110000
155	-3517.359962	-25.999087	0.000415	0.000082	0.013269	0.004039	0.110000
156	-3517.359968	-26.002704	0.000346	0.000079	0.012739	0.003986	0.110000
157	-3517.359973	-26.006124	0.000277	0.000074	0.012532	0.003972	0.110000
158	-3517.359979	-26.009390	0.000236	0.000069	0.012492	0.003955	0.109443
159	-3517.359984	-26.012509	0.000222	0.000066	0.012366	0.003866	0.106865
160	-3517.359988	-26.015491	0.000224	0.000065	0.012426	0.003810	0.105084
161	-3517.359993	-26.018317	0.000235	0.000065	0.012425	0.003752	0.103406
162	-3517.359997	-26.020968	0.000249	0.000066	0.012172	0.003670	0.101380
163	-3517.360001	-26.023441	0.000253	0.000067	0.011559	0.003546	0.098565
164	-3517.360005	-26.025750	0.000243	0.000065	0.010725	0.003380	0.094638
165	-3517.360008	-26.027856	0.000225	0.000062	0.010059	0.003218	0.090380
166	-3517.360011	-26.029849	0.000213	0.000058	0.009865	0.003142	0.087982
167	-3517.360014	-26.031782	0.000212	0.000057	0.010009	0.003152	0.087980
168	-3517.360017	-26.033660	0.000212	0.000057	0.010046	0.003163	0.088069
169	-3517.360020	-26.035466	0.000208	0.000058	0.009851	0.003099	0.086677
170	-3517.360023	-26.037155	0.000196	0.000057	0.009407	0.002978	0.083925
171	-3517.360025	-26.038780	0.000184	0.000055	0.008948	0.002866	0.081122
172	-3517.360028	-26.040484	0.000180	0.000053	0.008690	0.002782	0.078859
173	-3517.360030	-26.041896	0.000184	0.000052	0.008793	0.002750	0.078357
174	-3517.360032	-26.043240	0.000193	0.000053	0.008872	0.002813	0.079990
175	-3517.360035	-26.044614	0.000192	0.000053	0.008267	0.002717	0.077467
176	-3517.360037	-26.045896	0.000181	0.000050	0.007637	0.002525	0.072456
177	-3517.360039	-26.047096	0.000178	0.000048	0.007780	0.002508	0.071639
178	-3517.360041	-26.048272	0.000183	0.000048	0.008071	0.002574	0.073197
179	-3517.360043	-26.049533	0.000188	0.000049	0.008245	0.002595	0.073372
180	-3517.360044	-26.050725	0.000181	0.000050	0.008367	0.002601	0.073498
181	-3517.360046	-26.051907	0.000173	0.000050	0.008167	0.002558	0.072542
182	-3517.360048	-26.053233	0.000174	0.000048	0.008372	0.002591	0.071690
183	-3517.360051	-26.054558	0.000157	0.000047	0.009486	0.002794	0.076679
184	-3517.360052	-26.055758	0.000225	0.000052	0.009037	0.002786	0.077597
185	-3517.360054	-26.056858	0.000137	0.000038	0.007141	0.002280	0.069698
186	-3517.360055	-26.057414	0.000177	0.000040	0.010165	0.002913	0.078508
187	-3517.360057	-26.058360	0.000216	0.000048	0.010806	0.003156	0.080258
188	-3517.360059	-26.059761	0.000243	0.000054	0.010980	0.003324	0.082952
189	-3517.360063	-26.062116	0.000236	0.000064	0.009944	0.003281	0.082538
190	-3517.360065	-26.063352	0.000141	0.000044	0.008371	0.002532	0.073518
191	-3517.360081	-26.073852	0.000141	0.000047	0.010296	0.003050	0.078097
192	-3517.360067	-26.064932	0.000087	0.000027	0.001449	0.000360	0.079046
193	-3517.360069	-26.066075	0.000470	0.000100	0.013456	0.003863	0.110001
194	-3517.360070	-26.066710	0.000254	0.000080	0.008253	0.002379	0.074410
195	-3517.360073	-26.068725	0.000357	0.000116	0.015063	0.004533	0.110000
196	-3517.360077	-26.070985	0.000114	0.000032	0.006225	0.002395	0.082463
197	-3517.360079	-26.072336	0.000354	0.000075	0.014205	0.003936	0.110000
198	-3517.360080	-26.073265	0.000200	0.000053	0.010041	0.004060	0.110000

```
 199     -3517.360071   -26.067180    0.000162  0.000044  0.012145  0.003452  0.087413
 200     -3517.360084   -26.075480    0.000168  0.000044  0.009796  0.003144  0.078670
 201     -3517.360085   -26.076472    0.000126  0.000031  0.008882  0.002684  0.069123
 202     -3517.360088   -26.078284    0.000143  0.000038  0.010529  0.003152  0.079385
 203     -3517.360091   -26.079997    0.000268  0.000057  0.010046  0.003150  0.077544
 204     -3517.360085   -26.076188    0.000273  0.000056  0.005456  0.000899  0.058692
 205     -3517.360088   -26.077869    0.001142  0.000236  0.011950  0.003719  0.110000
 206     -3517.360086   -26.076925    0.000564  0.000109  0.010199  0.001571  0.031282
 207     -3517.360090   -26.079557    0.001044  0.000196  0.013027  0.004313  0.110000
 208     -3517.360093   -26.081138    0.000361  0.000082  0.006104  0.001829  0.057008
 209     -3517.360095   -26.082434    0.000784  0.000138  0.006608  0.002392  0.068837
 210     -3517.360091   -26.079680    0.000912  0.000168  0.007488  0.001950  0.051307
 211     -3517.360094   -26.081711    0.000395  0.000090  0.004782  0.000988  0.026172
 212     -3517.360091   -26.080061    0.000709  0.000204  0.008066  0.002860  0.088282
 213     -3517.360094   -26.081768    0.000393  0.000108  0.006720  0.002136  0.058871
 214     -3517.360094   -26.082125    0.000514  0.000139  0.006256  0.001568  0.042465
 215     -3517.360094   -26.082102    0.000385  0.000095  0.005539  0.001210  0.025630
 216     -3517.360091   -26.079954    0.000781  0.000214  0.014706  0.004155  0.110000
 217     -3517.360095   -26.082282    0.000364  0.000120  0.006707  0.002317  0.069905
 218     -3517.360095   -26.082505    0.000484  0.000124  0.005883  0.002273  0.072670
 219     -3517.360097   -26.083542    0.000505  0.000109  0.006068  0.001982  0.063909
 220     -3517.360100   -26.085287    0.000773  0.000184  0.009051  0.003064  0.091790
 221     -3517.360097   -26.083821    0.000699  0.000167  0.006260  0.002118  0.058579
 222     -3517.360098   -26.084501    0.000315  0.000073  0.003615  0.000937  0.022690
 223     -3517.360097   -26.083767    0.000893  0.000191  0.010259  0.003447  0.110000
 224     -3517.360100   -26.085911    0.000383  0.000091  0.005787  0.001417  0.039142
 225     -3517.360099   -26.085129    0.000435  0.000111  0.004425  0.001260  0.032260
 226     -3517.360099   -26.085271    0.000288  0.000097  0.004687  0.001356  0.039041
 227     -3517.360099   -26.084993    0.000465  0.000118  0.006122  0.002230  0.066192
 228     -3517.360104   -26.088121    0.000441  0.000097  0.005967  0.002358  0.072684
 229     -3517.360099   -26.085082    0.000722  0.000151  0.010245  0.002948  0.091317
 230     -3517.360103   -26.087472    0.000487  0.000116  0.009952  0.001704  0.039313
 231     -3517.360101   -26.086335    0.000374  0.000093  0.005259  0.001113  0.029288
 232     -3517.360105   -26.088592    0.000247  0.000066  0.003315  0.001262  0.039799
 233     -3517.360107   -26.090186    0.000455  0.000115  0.010748  0.003386  0.110000
 234     -3517.360104   -26.087816    0.000412  0.000119  0.006764  0.002111  0.060969
 235     -3517.360103   -26.087767    0.000287  0.000093  0.003812  0.001219  0.030127
 236     -3517.360100   -26.085774    0.000797  0.000252  0.009897  0.003583  0.110000
 237     -3517.360103   -26.087657    0.000337  0.000101  0.005290  0.001613  0.036831
 238     -3517.360106   -26.089642    0.000041  0.000013  0.001567  0.000384  0.008400
 239     -3517.360104   -26.088286    0.000605  0.000134  0.013888  0.004007  0.110002
 240     -3517.360105   -26.088886    0.000066  0.000019  0.004599  0.001772  0.053307

         **************************************************************
         *  MAXIMUM NUMBER OF ITERATIONS REACHED - STOPPING IRC RUN  *
         **************************************************************


----------------------------------------------------------------
                       IRC PATH SUMMARY
----------------------------------------------------------------
All gradients are in Eh/Bohr.

Step        E(Eh)      dE(kcal/mol)  max(|G|)   RMS(G)   SMW-Displ.
   1     -3517.360105   -26.088886    0.000066  0.000019  -23.529992
   2     -3517.360104   -26.088286    0.000605  0.000134  -23.476685
   3     -3517.360106   -26.089642    0.000041  0.000013  -23.366683
   4     -3517.360103   -26.087657    0.000337  0.000101  -23.358283
   5     -3517.360100   -26.085774    0.000797  0.000252  -23.321452
   6     -3517.360103   -26.087767    0.000287  0.000093  -23.211452
   7     -3517.360104   -26.087816    0.000412  0.000119  -23.181325
   8     -3517.360107   -26.090186    0.000455  0.000115  -23.120356
   9     -3517.360105   -26.088592    0.000247  0.000066  -23.010356
```

10	-3517.360101	-26.086335	0.000374	0.000093	-22.970557
11	-3517.360103	-26.087472	0.000487	0.000116	-22.941269
12	-3517.360099	-26.085082	0.000722	0.000151	-22.901956
13	-3517.360104	-26.088121	0.000441	0.000097	-22.810639
14	-3517.360099	-26.084993	0.000465	0.000118	-22.737955
15	-3517.360099	-26.085271	0.000288	0.000097	-22.671763
16	-3517.360099	-26.085129	0.000435	0.000111	-22.632722
17	-3517.360100	-26.085911	0.000383	0.000091	-22.600462
18	-3517.360097	-26.083767	0.000893	0.000191	-22.561320
19	-3517.360098	-26.084501	0.000315	0.000073	-22.451320
20	-3517.360097	-26.083821	0.000699	0.000167	-22.428630
21	-3517.360100	-26.085287	0.000773	0.000184	-22.370050
22	-3517.360097	-26.083542	0.000505	0.000109	-22.278261
23	-3517.360095	-26.082505	0.000484	0.000124	-22.214352
24	-3517.360095	-26.082282	0.000364	0.000120	-22.141681
25	-3517.360091	-26.079954	0.000781	0.000214	-22.071776
26	-3517.360094	-26.082102	0.000385	0.000095	-21.961776
27	-3517.360094	-26.082125	0.000514	0.000139	-21.936146
28	-3517.360094	-26.081768	0.000393	0.000108	-21.893681
29	-3517.360091	-26.080061	0.000709	0.000204	-21.834810
30	-3517.360094	-26.081711	0.000395	0.000090	-21.746527
31	-3517.360091	-26.079680	0.000912	0.000168	-21.720355
32	-3517.360095	-26.082434	0.000784	0.000138	-21.669048
33	-3517.360093	-26.081138	0.000361	0.000082	-21.600211
34	-3517.360090	-26.079557	0.001044	0.000196	-21.543203
35	-3517.360086	-26.076925	0.000564	0.000109	-21.433203
36	-3517.360088	-26.077869	0.001142	0.000236	-21.401921
37	-3517.360085	-26.076188	0.000273	0.000056	-21.291921
38	-3517.360091	-26.079997	0.000268	0.000057	-21.233229
39	-3517.360088	-26.078284	0.000143	0.000038	-21.155684
40	-3517.360085	-26.076472	0.000126	0.000031	-21.076299
41	-3517.360084	-26.075480	0.000168	0.000044	-21.007177
42	-3517.360071	-26.067180	0.000162	0.000044	-20.928506
43	-3517.360080	-26.073265	0.000200	0.000053	-20.841093
44	-3517.360079	-26.072336	0.000354	0.000075	-20.731093
45	-3517.360077	-26.070985	0.000114	0.000032	-20.621093
46	-3517.360073	-26.068725	0.000357	0.000116	-20.538630
47	-3517.360070	-26.066710	0.000254	0.000080	-20.428630
48	-3517.360069	-26.066075	0.000470	0.000100	-20.354220
49	-3517.360067	-26.064932	0.000087	0.000027	-20.244219
50	-3517.360081	-26.073852	0.000141	0.000047	-20.165173
51	-3517.360065	-26.063352	0.000141	0.000044	-20.087075
52	-3517.360063	-26.062116	0.000236	0.000064	-20.013557
53	-3517.360059	-26.059761	0.000243	0.000054	-19.931019
54	-3517.360057	-26.058360	0.000216	0.000048	-19.848068
55	-3517.360055	-26.057414	0.000177	0.000040	-19.767809
56	-3517.360054	-26.056858	0.000137	0.000038	-19.689301
57	-3517.360052	-26.055758	0.000225	0.000052	-19.619603
58	-3517.360051	-26.054558	0.000157	0.000047	-19.542006
59	-3517.360048	-26.053233	0.000174	0.000048	-19.465327
60	-3517.360046	-26.051907	0.000173	0.000050	-19.393637
61	-3517.360044	-26.050725	0.000181	0.000050	-19.321095
62	-3517.360043	-26.049533	0.000188	0.000049	-19.247597
63	-3517.360041	-26.048272	0.000183	0.000048	-19.174225
64	-3517.360039	-26.047096	0.000178	0.000048	-19.101028
65	-3517.360037	-26.045896	0.000181	0.000050	-19.029389
66	-3517.360035	-26.044614	0.000192	0.000053	-18.956933
67	-3517.360032	-26.043240	0.000193	0.000053	-18.879466
68	-3517.360030	-26.041896	0.000184	0.000052	-18.799475
69	-3517.360028	-26.040484	0.000180	0.000053	-18.721118
70	-3517.360025	-26.038780	0.000184	0.000055	-18.642259
71	-3517.360023	-26.037155	0.000196	0.000057	-18.561137
72	-3517.360020	-26.035466	0.000208	0.000058	-18.477212

73	-3517.360017	-26.033660	0.000212	0.000057	-18.390536
74	-3517.360014	-26.031782	0.000212	0.000057	-18.302466
75	-3517.360011	-26.029849	0.000213	0.000058	-18.214486
76	-3517.360008	-26.027856	0.000225	0.000062	-18.126505
77	-3517.360005	-26.025750	0.000243	0.000065	-18.036124
78	-3517.360001	-26.023441	0.000253	0.000067	-17.941486
79	-3517.359997	-26.020968	0.000249	0.000066	-17.842922
80	-3517.359993	-26.018317	0.000235	0.000065	-17.741542
81	-3517.359988	-26.015491	0.000224	0.000065	-17.638136
82	-3517.359984	-26.012509	0.000222	0.000066	-17.533052
83	-3517.359979	-26.009390	0.000236	0.000069	-17.426187
84	-3517.359973	-26.006124	0.000277	0.000074	-17.316744
85	-3517.359968	-26.002704	0.000346	0.000079	-17.206744
86	-3517.359962	-25.999087	0.000415	0.000082	-17.096744
87	-3517.359956	-25.995260	0.000418	0.000079	-16.986744
88	-3517.359950	-25.991206	0.000328	0.000069	-16.876744
89	-3517.359943	-25.987152	0.000281	0.000058	-16.766744
90	-3517.359936	-25.982588	0.000296	0.000050	-16.656744
91	-3517.359928	-25.977647	0.000311	0.000047	-16.546744
92	-3517.359920	-25.972391	0.000332	0.000047	-16.436744
93	-3517.359913	-25.968480	0.000347	0.000050	-16.326744
94	-3517.359900	-25.960117	0.000371	0.000058	-16.216743
95	-3517.359891	-25.954142	0.000396	0.000069	-16.106743
96	-3517.359881	-25.948213	0.000417	0.000083	-15.996743
97	-3517.359872	-25.942418	0.000446	0.000097	-15.886743
98	-3517.359860	-25.935004	0.000519	0.000099	-15.776743
99	-3517.359856	-25.932651	0.000489	0.000068	-15.666743
100	-3517.359838	-25.921309	0.000510	0.000074	-15.556743
101	-3517.359825	-25.913078	0.000534	0.000085	-15.446743
102	-3517.359814	-25.905829	0.000555	0.000098	-15.336742
103	-3517.359798	-25.895861	0.000564	0.000112	-15.226742
104	-3517.359785	-25.887853	0.001128	0.000197	-15.116742
105	-3517.359769	-25.877682	0.002045	0.000359	-15.006742
106	-3517.359760	-25.872481	0.002646	0.000416	-14.896742
107	-3517.359760	-25.872042	0.001725	0.000313	-14.786742
108	-3517.359761	-25.873073	0.001223	0.000214	-14.676742
109	-3517.359748	-25.864954	0.000639	0.000091	-14.566741
110	-3517.359729	-25.852975	0.001320	0.000250	-14.456741
111	-3517.359716	-25.844415	0.000646	0.000118	-14.346741
112	-3517.359701	-25.835099	0.001422	0.000266	-14.236741
113	-3517.359686	-25.826119	0.001541	0.000232	-14.126741
114	-3517.359669	-25.815253	0.000817	0.000156	-14.016741
115	-3517.359652	-25.804290	0.001141	0.000208	-13.906740
116	-3517.359645	-25.800174	0.001241	0.000238	-13.796740
117	-3517.359628	-25.789194	0.000950	0.000224	-13.686740
118	-3517.359610	-25.778144	0.000966	0.000195	-13.576740
119	-3517.359589	-25.764952	0.001241	0.000227	-13.466740
120	-3517.359568	-25.751983	0.001261	0.000228	-13.356740
121	-3517.359547	-25.738868	0.001325	0.000238	-13.246739
122	-3517.359526	-25.725660	0.001217	0.000223	-13.136739
123	-3517.359503	-25.711031	0.001382	0.000247	-13.026739
124	-3517.359480	-25.696630	0.001328	0.000243	-12.916739
125	-3517.359456	-25.681574	0.001425	0.000257	-12.806739
126	-3517.359432	-25.666326	0.001341	0.000250	-12.696739
127	-3517.359406	-25.650129	0.001468	0.000268	-12.586738
128	-3517.359381	-25.634165	0.001328	0.000252	-12.476738
129	-3517.359352	-25.616437	0.001546	0.000280	-12.366738
130	-3517.359324	-25.598824	0.001405	0.000268	-12.256738
131	-3517.359291	-25.577662	0.001550	0.000288	-12.146738
132	-3517.359268	-25.563209	0.001420	0.000279	-12.036737
133	-3517.359237	-25.543824	0.001494	0.000302	-11.926737
134	-3517.359206	-25.524611	0.001538	0.000290	-11.816737
135	-3517.359175	-25.505315	0.001546	0.000305	-11.706737

136	-3517.359141	-25.483839	0.001624	0.000316	-11.596736
137	-3517.359107	-25.462468	0.001674	0.000314	-11.486736
138	-3517.359070	-25.439548	0.001738	0.000325	-11.376736
139	-3517.359033	-25.416199	0.001798	0.000326	-11.266736
140	-3517.358996	-25.392653	0.001862	0.000333	-11.156736
141	-3517.358956	-25.368013	0.001927	0.000335	-11.046735
142	-3517.358918	-25.343827	0.001993	0.000346	-10.936735
143	-3517.358874	-25.316378	0.002061	0.000348	-10.826735
144	-3517.358831	-25.289296	0.002131	0.000354	-10.716735
145	-3517.358786	-25.261062	0.002202	0.000362	-10.606735
146	-3517.358740	-25.232075	0.002277	0.000367	-10.496735
147	-3517.358692	-25.201820	0.002352	0.000375	-10.386734
148	-3517.358642	-25.170781	0.002431	0.000380	-10.276734
149	-3517.358591	-25.138461	0.002511	0.000389	-10.166734
150	-3517.358537	-25.105083	0.002596	0.000393	-10.056734
151	-3517.358482	-25.070515	0.002683	0.000400	-9.946733
152	-3517.358425	-25.034653	0.002773	0.000408	-9.836733
153	-3517.358366	-24.997681	0.002873	0.000416	-9.726733
154	-3517.358305	-24.959058	0.002974	0.000425	-9.616732
155	-3517.358241	-24.918926	0.003076	0.000434	-9.506732
156	-3517.358174	-24.877241	0.003180	0.000443	-9.396732
157	-3517.358106	-24.834037	0.003288	0.000453	-9.286731
158	-3517.358034	-24.789202	0.003400	0.000463	-9.176731
159	-3517.357960	-24.742706	0.003515	0.000474	-9.066731
160	-3517.357883	-24.694390	0.003635	0.000485	-8.956730
161	-3517.357803	-24.644332	0.003759	0.000495	-8.846730
162	-3517.357720	-24.592224	0.003888	0.000508	-8.736730
163	-3517.357634	-24.538333	0.004020	0.000523	-8.626730
164	-3517.357545	-24.482457	0.004155	0.000539	-8.516730
165	-3517.357454	-24.424994	0.004301	0.000548	-8.406729
166	-3517.357358	-24.364747	0.004465	0.000549	-8.296729
167	-3517.357257	-24.301562	0.004656	0.000566	-8.186727
168	-3517.357153	-24.236162	0.004992	0.000917	-8.076723
169	-3517.357120	-24.215731	0.004897	0.000858	-7.966723
170	-3517.357031	-24.160074	0.005071	0.000792	-7.856723
171	-3517.356927	-24.094283	0.005212	0.000894	-7.746722
172	-3517.356832	-24.034973	0.005383	0.000834	-7.636722
173	-3517.356723	-23.966210	0.005534	0.000905	-7.526721
174	-3517.356620	-23.901987	0.005733	0.000842	-7.416721
175	-3517.356500	-23.826352	0.005900	0.000961	-7.306720
176	-3517.356392	-23.758749	0.006098	0.000892	-7.196720
177	-3517.356266	-23.679526	0.006276	0.000980	-7.086719
178	-3517.356148	-23.605772	0.006500	0.000919	-6.976719
179	-3517.356011	-23.519650	0.006699	0.001041	-6.866718
180	-3517.355886	-23.441136	0.006921	0.000991	-6.756718
181	-3517.355744	-23.351953	0.007128	0.001064	-6.646717
182	-3517.355600	-23.262124	0.007338	0.001030	-6.536717
183	-3517.355454	-23.170375	0.007608	0.001021	-6.426716
184	-3517.355287	-23.065626	0.007840	0.001168	-6.316715
185	-3517.355132	-22.968316	0.008073	0.001096	-6.206715
186	-3517.354965	-22.863041	0.008365	0.001106	-6.096714
187	-3517.354778	-22.745988	0.008630	0.001259	-5.986714
188	-3517.354609	-22.639702	0.008898	0.001206	-5.876714
189	-3517.354415	-22.518149	0.009165	0.001301	-5.766713
190	-3517.354226	-22.399652	0.009432	0.001292	-5.656711
191	-3517.354017	-22.268724	0.009702	0.001332	-5.546710
192	-3517.353812	-22.139817	0.010017	0.001321	-5.436710
193	-3517.353583	-21.995820	0.010322	0.001456	-5.326709
194	-3517.353366	-21.859679	0.010624	0.001433	-5.216709
195	-3517.353127	-21.709967	0.010936	0.001511	-5.106707
196	-3517.352888	-21.559719	0.011249	0.001528	-4.996707
197	-3517.352633	-21.400218	0.011572	0.001585	-4.886705
198	-3517.352372	-21.236442	0.011901	0.001619	-4.776705

199	-3517.352099	-21.064767	0.012237	0.001670	-4.666704
200	-3517.351816	-20.887109	0.012579	0.001712	-4.556703
201	-3517.351520	-20.701696	0.012931	0.001763	-4.446701
202	-3517.351214	-20.509333	0.013289	0.001811	-4.336701
203	-3517.350894	-20.308837	0.013652	0.001863	-4.226700
204	-3517.350562	-20.100577	0.014022	0.001914	-4.116699
205	-3517.350217	-19.883676	0.014399	0.001970	-4.006698
206	-3517.349857	-19.658240	0.014783	0.002024	-3.896697
207	-3517.349483	-19.423439	0.015175	0.002082	-3.786696
208	-3517.349095	-19.179660	0.015569	0.002142	-3.676696
209	-3517.348690	-18.925822	0.015976	0.002202	-3.566695
210	-3517.348270	-18.661882	0.016386	0.002267	-3.456694
211	-3517.347832	-18.387284	0.016803	0.002331	-3.346693
212	-3517.347377	-18.101581	0.017233	0.002397	-3.236691
213	-3517.346903	-17.804062	0.017687	0.002466	-3.126688
214	-3517.346409	-17.494395	0.018134	0.002538	-3.016688
215	-3517.345897	-17.172968	0.018581	0.002615	-2.906686
216	-3517.345360	-16.836334	0.019012	0.002672	-2.796684
217	-3517.344797	-16.483092	0.019430	0.002715	-2.686682
218	-3517.344200	-16.107917	0.019877	0.002787	-2.576680
219	-3517.343576	-15.716807	0.020341	0.002872	-2.466677
220	-3517.342928	-15.309717	0.020811	0.002963	-2.356677
221	-3517.342254	-14.887386	0.021282	0.003060	-2.246676
222	-3517.341556	-14.449223	0.021748	0.003160	-2.136675
223	-3517.340829	-13.992742	0.022206	0.003266	-2.026672
224	-3517.340076	-13.520527	0.022653	0.003376	-1.916669
225	-3517.339297	-13.031516	0.023082	0.003491	-1.806666
226	-3517.338484	-12.521248	0.023485	0.003612	-1.696663
227	-3517.337638	-11.990595	0.023851	0.003739	-1.586661
228	-3517.336757	-11.437372	0.024167	0.003876	-1.476661
229	-3517.335833	-10.857836	0.024421	0.004024	-1.366661
230	-3517.334868	-10.252170	0.024595	0.004188	-1.256659
231	-3517.333844	-9.609728	0.024672	0.004369	-1.146656
232	-3517.332758	-8.927962	0.024635	0.004572	-1.036652
233	-3517.331591	-8.195981	0.024462	0.004797	-0.926648
234	-3517.330325	-7.401577	0.025875	0.005041	-0.816646
235	-3517.328930	-6.526275	0.026996	0.005274	-0.706644
236	-3517.327344	-5.531192	0.026823	0.005408	-0.596641
237	-3517.325402	-4.312205	0.024444	0.005300	-0.486637
238	-3517.323100	-2.867870	0.020517	0.004804	-0.376633
239	-3517.320836	-1.447315	0.015427	0.003731	-0.266632
240	-3517.319239	-0.445276	0.008383	0.002103	-0.156625
241	-3517.318581	-0.032093	0.002382	0.000553	-0.046619
242	-3517.318530	0.000000	0.000099	0.000022	0.000000 <= TS
243	-3517.318571	-0.025918	0.002245	0.000500	0.046619
244	-3517.318955	-0.266539	0.007377	0.001456	0.156623
245	-3517.319544	-0.636202	0.011483	0.002121	0.266625
246	-3517.320209	-1.053527	0.014195	0.002539	0.376625
247	-3517.320906	-1.491049	0.015873	0.002808	0.486627
248	-3517.321633	-1.947048	0.017135	0.002993	0.596630
249	-3517.322373	-2.411849	0.018535	0.003130	0.706630
250	-3517.323132	-2.887616	0.019708	0.003239	0.816632
251	-3517.323904	-3.372357	0.020719	0.003330	0.926634
252	-3517.324687	-3.863786	0.021609	0.003410	1.036637
253	-3517.325482	-4.362374	0.022405	0.003479	1.146640
254	-3517.326284	-4.866084	0.023120	0.003540	1.256640
255	-3517.327094	-5.373984	0.023769	0.003589	1.366643
256	-3517.327910	-5.886176	0.024357	0.003639	1.476644
257	-3517.328731	-6.401100	0.024882	0.003683	1.586645
258	-3517.329559	-6.920802	0.025345	0.003721	1.696647
259	-3517.330394	-7.445015	0.025746	0.003753	1.806648
260	-3517.331227	-7.967706	0.026076	0.003778	1.916650
261	-3517.332068	-8.495019	0.026337	0.003794	2.026651

262	-3517.332906	-9.021117	0.026523	0.003803	2.136652
263	-3517.333745	-9.547673	0.026632	0.003804	2.246652
264	-3517.334590	-10.077703	0.026665	0.003795	2.356653
265	-3517.335424	-10.600985	0.026612	0.003776	2.466656
266	-3517.336239	-11.112912	0.026469	0.003745	2.576660
267	-3517.337055	-11.624668	0.026247	0.003704	2.686660
268	-3517.337857	-12.127988	0.025941	0.003653	2.796663
269	-3517.338641	-12.619767	0.025554	0.003593	2.906663
270	-3517.339401	-13.096727	0.025082	0.003524	3.016664
271	-3517.340138	-13.559321	0.024515	0.003443	3.126667
272	-3517.340854	-14.008822	0.023864	0.003350	3.236667
273	-3517.341548	-14.443938	0.023132	0.003248	3.346668
274	-3517.342216	-14.862946	0.022321	0.003137	3.456669
275	-3517.342855	-15.264263	0.021434	0.003018	3.566672
276	-3517.343466	-15.647340	0.020473	0.002890	3.676672
277	-3517.344046	-16.011438	0.019443	0.002755	3.786673
278	-3517.344595	-16.355812	0.018348	0.002612	3.896674
279	-3517.345111	-16.679992	0.017195	0.002464	4.006676
280	-3517.345594	-16.983217	0.015989	0.002311	4.116678
281	-3517.346046	-17.266328	0.014740	0.002154	4.226680
282	-3517.346463	-17.528303	0.013460	0.001995	4.336681
283	-3517.346848	-17.770078	0.012161	0.001834	4.446681
284	-3517.347201	-17.991482	0.010861	0.001678	4.556681
285	-3517.347526	-18.195014	0.009574	0.001518	4.666682
286	-3517.347816	-18.377489	0.008336	0.001386	4.776684
287	-3517.348092	-18.550618	0.007126	0.001209	4.886684
288	-3517.348320	-18.693737	0.006313	0.001179	4.996684
289	-3517.348565	-18.847261	0.005835	0.000943	5.106685
290	-3517.348759	-18.969263	0.005346	0.001074	5.216685
291	-3517.348973	-19.103123	0.004909	0.000773	5.326687
292	-3517.349145	-19.210972	0.004394	0.000822	5.436689
293	-3517.349333	-19.329254	0.003992	0.000605	5.546691
294	-3517.349500	-19.434013	0.003605	0.000723	5.656691
295	-3517.349660	-19.534133	0.003306	0.000702	5.766691
296	-3517.349820	-19.635035	0.003275	0.000667	5.876691
297	-3517.349981	-19.736127	0.003593	0.000661	5.986692
298	-3517.350131	-19.830281	0.003734	0.000655	6.096694
299	-3517.350287	-19.927760	0.003530	0.000618	6.206695
300	-3517.350444	-20.026260	0.003246	0.000577	6.316696
301	-3517.350601	-20.125049	0.002983	0.000543	6.426696
302	-3517.350762	-20.225724	0.002696	0.000513	6.536697
303	-3517.350924	-20.327346	0.002267	0.000483	6.646697
304	-3517.351084	-20.428269	0.001906	0.000470	6.756698
305	-3517.351238	-20.524319	0.002028	0.000517	6.866698
306	-3517.351391	-20.620413	0.002024	0.000520	6.976698
307	-3517.351545	-20.717382	0.001914	0.000512	7.086700
308	-3517.351700	-20.814322	0.001877	0.000510	7.196701
309	-3517.351853	-20.910437	0.001942	0.000515	7.306702
310	-3517.352004	-21.005556	0.001999	0.000519	7.416703
311	-3517.352155	-21.099761	0.002031	0.000521	7.526703
312	-3517.352303	-21.192861	0.002055	0.000522	7.636703
313	-3517.352449	-21.284758	0.002082	0.000521	7.746704
314	-3517.352593	-21.374931	0.002148	0.000526	7.856704
315	-3517.352735	-21.463989	0.002151	0.000522	7.966705
316	-3517.352874	-21.551299	0.002206	0.000525	8.076705
317	-3517.353011	-21.637421	0.002202	0.000519	8.186706
318	-3517.353146	-21.721989	0.002221	0.000518	8.296706
319	-3517.353278	-21.804981	0.002255	0.000517	8.406706
320	-3517.353408	-21.886427	0.002280	0.000516	8.516707
321	-3517.353536	-21.966360	0.002302	0.000514	8.626707
322	-3517.353660	-22.044746	0.002324	0.000513	8.736708
323	-3517.353783	-22.121529	0.002344	0.000511	8.846708
324	-3517.353902	-22.196261	0.002361	0.000509	8.956708

325	-3517.354019	-22.269764	0.002371	0.000506	9.066709
326	-3517.354133	-22.341523	0.002384	0.000504	9.176710
327	-3517.354245	-22.411533	0.002388	0.000502	9.286710
328	-3517.354353	-22.479600	0.002389	0.000499	9.396710
329	-3517.354459	-22.546042	0.002378	0.000496	9.506710
330	-3517.354562	-22.610384	0.002360	0.000493	9.616711
331	-3517.354661	-22.672789	0.002314	0.000489	9.726711
332	-3517.354757	-22.733112	0.002246	0.000485	9.836711
333	-3517.354853	-22.793128	0.002125	0.000479	9.946711
334	-3517.354943	-22.849525	0.001964	0.000479	10.056712
335	-3517.355032	-22.905223	0.001621	0.000457	10.166712
336	-3517.355115	-22.957551	0.001860	0.000474	10.276712
337	-3517.355210	-23.017119	0.001738	0.000436	10.386713
338	-3517.355260	-23.048629	0.002111	0.000510	10.496713
339	-3517.355342	-23.100070	0.001348	0.000358	10.606713
340	-3517.355413	-23.144719	0.001426	0.000384	10.716713
341	-3517.355493	-23.194784	0.001342	0.000295	10.826714
342	-3517.355544	-23.226699	0.003569	0.000607	10.936714
343	-3517.355613	-23.270140	0.002067	0.000419	11.046715
344	-3517.355668	-23.304513	0.001847	0.000442	11.156715
345	-3517.355716	-23.334593	0.002593	0.000497	11.266715
346	-3517.355763	-23.364083	0.002476	0.000477	11.376716
347	-3517.355804	-23.389527	0.002625	0.000508	11.486716
348	-3517.355811	-23.394357	0.002821	0.000514	11.596716
349	-3517.355867	-23.429572	0.001065	0.000273	11.706717
350	-3517.355918	-23.461164	0.001152	0.000256	11.816718
351	-3517.355960	-23.487672	0.001640	0.000308	11.926718
352	-3517.356002	-23.514316	0.001609	0.000303	12.036718
353	-3517.356043	-23.539578	0.001534	0.000299	12.146718
354	-3517.356081	-23.563704	0.001478	0.000299	12.256718
355	-3517.356118	-23.586887	0.001424	0.000303	12.366718
356	-3517.356154	-23.609204	0.001337	0.000310	12.476719
357	-3517.356188	-23.631027	0.001203	0.000320	12.586719
358	-3517.356220	-23.650628	0.001495	0.000337	12.696719
359	-3517.356255	-23.672665	0.001673	0.000350	12.806719
360	-3517.356289	-23.693931	0.001035	0.000263	12.916719
361	-3517.356319	-23.712849	0.001063	0.000292	13.026720
362	-3517.356349	-23.731899	0.001412	0.000299	13.136720
363	-3517.356379	-23.750871	0.001504	0.000315	13.246720
364	-3517.356407	-23.767985	0.001211	0.000324	13.356720
365	-3517.356435	-23.785744	0.001678	0.000344	13.466721
366	-3517.356467	-23.805932	0.001319	0.000272	13.576721
367	-3517.356492	-23.821804	0.001263	0.000299	13.686721
368	-3517.356522	-23.840160	0.001445	0.000268	13.796721
369	-3517.356548	-23.856441	0.001444	0.000294	13.906721
370	-3517.356572	-23.871670	0.001224	0.000314	14.016721
371	-3517.356602	-23.890350	0.001740	0.000343	14.126721
372	-3517.356634	-23.910599	0.001576	0.000321	14.236722
373	-3517.356637	-23.912344	0.001537	0.000294	14.346722
374	-3517.356664	-23.929202	0.000844	0.000215	14.456722
375	-3517.356686	-23.943240	0.001256	0.000326	14.566722
376	-3517.356712	-23.959737	0.001123	0.000241	14.676722
377	-3517.356741	-23.977692	0.000665	0.000136	14.786723
378	-3517.356761	-23.990053	0.001589	0.000342	14.896723
379	-3517.356790	-24.008826	0.000675	0.000135	15.006723
380	-3517.356813	-24.023228	0.001148	0.000294	15.116724
381	-3517.356834	-24.036288	0.000903	0.000217	15.226724
382	-3517.356855	-24.049459	0.000789	0.000221	15.336724
383	-3517.356883	-24.066667	0.000723	0.000210	15.446725
384	-3517.356908	-24.082605	0.000831	0.000249	15.556725
385	-3517.356929	-24.095600	0.000976	0.000289	15.666725
386	-3517.356959	-24.114725	0.001055	0.000291	15.776725
387	-3517.356983	-24.129739	0.001118	0.000292	15.886725

388	-3517.357008	-24.145234	0.001159	0.000294	15.996726
389	-3517.357033	-24.160829	0.001173	0.000297	16.106726
390	-3517.357058	-24.176459	0.001135	0.000293	16.216726
391	-3517.357084	-24.192990	0.001066	0.000288	16.326726
392	-3517.357109	-24.208759	0.000921	0.000278	16.436726
393	-3517.357133	-24.223602	0.001049	0.000268	16.546726
394	-3517.357159	-24.240214	0.001166	0.000263	16.656726
395	-3517.357182	-24.254281	0.001210	0.000267	16.766727
396	-3517.357206	-24.269550	0.001168	0.000268	16.876727
397	-3517.357230	-24.284605	0.001138	0.000275	16.986727
398	-3517.357253	-24.299341	0.001129	0.000275	17.096727
399	-3517.357278	-24.314646	0.001149	0.000267	17.206727
400	-3517.357302	-24.329557	0.001186	0.000264	17.316727
401	-3517.357327	-24.345702	0.001193	0.000266	17.426728
402	-3517.357351	-24.360331	0.001144	0.000270	17.536728
403	-3517.357374	-24.374752	0.001072	0.000269	17.646728
404	-3517.357398	-24.389768	0.001059	0.000265	17.756728
405	-3517.357421	-24.404332	0.001124	0.000267	17.866728
406	-3517.357442	-24.417644	0.001156	0.000264	17.976728
407	-3517.357467	-24.433250	0.001193	0.000266	18.086729
408	-3517.357487	-24.445748	0.001191	0.000268	18.196729
409	-3517.357510	-24.460287	0.001170	0.000262	18.306729
410	-3517.357534	-24.475669	0.001203	0.000267	18.416729
411	-3517.357557	-24.489730	0.001182	0.000265	18.526729
412	-3517.357577	-24.502313	0.001136	0.000261	18.636729
413	-3517.357601	-24.517350	0.001156	0.000266	18.746729
414	-3517.357623	-24.531175	0.001158	0.000262	18.856729
415	-3517.357645	-24.544945	0.001140	0.000260	18.966730
416	-3517.357664	-24.556844	0.001150	0.000256	19.076730
417	-3517.357688	-24.571764	0.001172	0.000264	19.186730
418	-3517.357709	-24.584985	0.001218	0.000266	19.296730
419	-3517.357727	-24.596708	0.001140	0.000254	19.406730
420	-3517.357749	-24.610427	0.001172	0.000265	19.516730
421	-3517.357770	-24.623596	0.001104	0.000242	19.626730
422	-3517.357786	-24.633841	0.001125	0.000258	19.736730
423	-3517.357809	-24.648241	0.001117	0.000254	19.846731
424	-3517.357828	-24.660131	0.001223	0.000265	19.956731
425	-3517.357849	-24.673342	0.001099	0.000244	20.066731
426	-3517.357868	-24.685074	0.001179	0.000263	20.176731
427	-3517.357887	-24.696806	0.001043	0.000236	20.286731
428	-3517.357902	-24.706170	0.001161	0.000266	20.396732
429	-3517.357922	-24.718999	0.001140	0.000256	20.506732
430	-3517.357941	-24.731069	0.001247	0.000275	20.616732
431	-3517.357961	-24.743129	0.001224	0.000258	20.726732
432	-3517.357980	-24.755179	0.001171	0.000269	20.836732
433	-3517.357999	-24.767379	0.001191	0.000263	20.946732
434	-3517.358018	-24.779202	0.001192	0.000270	21.056733
435	-3517.358037	-24.791249	0.001232	0.000269	21.166733
436	-3517.358056	-24.802989	0.001243	0.000273	21.276733
437	-3517.358075	-24.814902	0.001248	0.000270	21.386733
438	-3517.358094	-24.826535	0.001236	0.000272	21.496733
439	-3517.358112	-24.838331	0.001216	0.000268	21.606733
440	-3517.358131	-24.849887	0.001211	0.000271	21.716733
441	-3517.358149	-24.861509	0.001199	0.000266	21.826733
442	-3517.358167	-24.872795	0.001212	0.000271	21.936733
443	-3517.358185	-24.884233	0.001192	0.000265	22.046734
444	-3517.358203	-24.895205	0.001201	0.000272	22.156734
445	-3517.358221	-24.906464	0.001152	0.000259	22.266734
446	-3517.358238	-24.917100	0.001172	0.000272	22.376734
447	-3517.358256	-24.928290	0.001111	0.000252	22.486734
448	-3517.358272	-24.938529	0.001181	0.000276	22.596734
449	-3517.358290	-24.949791	0.001077	0.000242	22.706734
450	-3517.358305	-24.959436	0.001190	0.000273	22.816734

```
 451     -3517.358324   -24.971132    0.000997  0.000235  22.926734
 452     -3517.358341   -24.981644    0.001166  0.000260  23.036734
 453     -3517.358363   -24.995615    0.001139  0.000254  23.146735
 454     -3517.358365   -24.997180    0.001167  0.000242  23.256735
 455     -3517.358381   -25.006944    0.000904  0.000197  23.366735
 456     -3517.358399   -25.018517    0.000840  0.000175  23.476735
 457     -3517.358412   -25.026615    0.000881  0.000217  23.586735
 458     -3517.358426   -25.035399    0.001447  0.000277  23.696736
 459     -3517.358430   -25.037743    0.001499  0.000279  23.806736
 460     -3517.358449   -25.049547    0.000923  0.000169  23.916736
 461     -3517.358461   -25.057305    0.001446  0.000291  24.026736
 462     -3517.358472   -25.063779    0.000795  0.000212  24.136736
 463     -3517.358458   -25.055498    0.002214  0.000500  24.246737
 464     -3517.358461   -25.057144    0.002436  0.000522  24.356737
 465     -3517.358465   -25.059903    0.001868  0.000480  24.466737
 466     -3517.358486   -25.072805    0.000825  0.000205  24.576737
 467     -3517.358504   -25.083992    0.000545  0.000120  24.686737
 468     -3517.358520   -25.094253    0.000518  0.000128  24.796737
 469     -3517.358536   -25.104146    0.000529  0.000141  24.906738
 470     -3517.358551   -25.113667    0.000607  0.000154  25.016738
 471     -3517.358566   -25.122714    0.000718  0.000167  25.126738
 472     -3517.358580   -25.131976    0.000795  0.000173  25.236738
 473     -3517.358594   -25.140470    0.000801  0.000169  25.346738
 474     -3517.358609   -25.149983    0.000756  0.000160  25.456738
 475     -3517.358623   -25.158682    0.000695  0.000152  25.566738
 476     -3517.358636   -25.167167    0.000648  0.000151  25.676738
 477     -3517.358650   -25.175886    0.000622  0.000153  25.786738
 478     -3517.358663   -25.183986    0.000616  0.000155  25.896738
 479     -3517.358676   -25.191825    0.000639  0.000161  26.006738
 480     -3517.358688   -25.199466    0.000667  0.000167  26.116738
 481     -3517.358699   -25.206742    0.000690  0.000172  26.226738
 482     -3517.358711   -25.214155    0.000705  0.000174  26.336738
 483     -3517.358723   -25.221387    0.000702  0.000173  26.446738

-------------------------------
SUGGESTED CITATIONS FOR THIS RUN
-------------------------------

Below you find a list of papers that are relevant to this ORCA run
We neither can nor want to force you to cite these papers, but we appreciate if you do
You receive ORCA, which is the product of decades of hard work by many enthusiastic
individuals, for free
The only thing we kindly ask in return is that you cite our papers,
We deeply appreciate it, if you show your appreciation for ORCA by not just citing the generic
ORCA reference.

Please note that relegating all ORCA citations to the supporting information does *not* help
us.
SI sections are not indexed - citations you put there will not count into any citation
statistics
But we need these citations in order to attract the funding resources that allow us to do what
we are doing

Therefore, if you are a happy ORCA user, please consider citing a few of the papers listed
below in the main body of your paper

In addition to the list printed below, the program has created the file Cl-IRC.bibtex that
contains the list in bibtex format
You can import this file easily into all common literature databanks and citation aid programs

It goes without saying that in many instances, there are alternative algorithms to achieve
similar
```

results as the ones you have gotten from ORCA. It is, of course, also the case that in some instances
ORCA just re-implements algorithms worked out by others. We are fully aware of that and we are also
fully appreciative of our colleagues work. Hence this citation list should not be read as indicating
that the listed papers, which are focused on our own work, are the only ones worth citing. It simply
meant to make it easier for users to cite ORCA specific papers. It is not a substitute for doing your
own literature research and citing the relevant literature in a scientifically appropriate manner.

List of essential papers. We consider these as the minimum necessary citations

1. Neese, F.
   Software update: the ORCA program system, version 6.0
   WIRES Comput. Molec. Sci. 2025 15(1), e70019
   doi.org/10.1002/wcms.70019

List of papers to cite with high priority. The work reported in these papers was absolutely necessary for this run to complete.
Our perspective: the developers of density functionals and basis sets usually get cited in chemistry papers
Good! But without the algorithms to do something with them, the functionals or basis sets would not do anything.
Hence, in our opinion, the algorithm design and method developments papers are equally worthy of getting cited

1. Page, M.; Doubleday, C.; McIver, J.
   Following steepest descent reaction paths. The use of higher energy derivatives with abinitio electronic structure methods
   J. Chem. Phys. 1990 93(8), 5634-5642
   doi.org/10.1063/1.459634
2. Neese, F.
   An improvement of the resolution of the identity approximation for the formation of the Coulomb matrix
   J. Comp. Chem. 2003 24(14), 1740-1747
   doi.org/10.1002/jcc.10318
3. Hratchian, H.; Schlegel, B.
   Using Hessian Updating To Increase the Efficiency of a Hessian Based Predictor-Corrector Reaction Path Following Method
   J. Chem. Theory Comput. 2005 1(1), 61-69
   doi.org/10.1021/ct0499783
4. Neese, F.; Wennmohs, F.; Hansen, A.; Becker, U.
   Efficient, approximate and parallel Hartree-Fock and hybrid DFT calculations. A 'chain-of-spheres' algorithm for the Hartree-Fock exchange
   Chem. Phys. 2009 356(1-3), 98-109
   doi.org/10.1016/j.chemphys.2008.10.036
5. Grimme, S.; Antony, J.; Ehrlich, S.; Krieg, H.
   A consistent and accurate ab initio parametrization of density functional dispersion correction (DFT-D) for the 94 elements H-Pu
   J. Chem. Phys. 2010 132 , 154104
   doi.org/10.1063/1.3382344
6. Grimme, S.; Ehrlich, S.; Goerigk, L.
   Effect of the damping function in dispersion corrected density functional theory
   J. Comput. Chem. 2011 32(7), 1456-1465
   doi.org/10.1002/jcc.21759
7. Garcia-Rates, M.; Neese, F.
   Effect of the Solute Cavity on the Solvation Energy and its Derivatives within the Framework of the Gaussian Charge Scheme
   J. Comput. Chem. 2020 41 , 922-939
   doi.org/10.1002/jcc.26139

```
  8. Helmich-Paris, B.; de Souza, B.; Neese, F.; Izsák, R.
     An improved chain of spheres for exchange algorithm
     J. Chem. Phys. 2021 155(10), 104109
     doi.org/10.1063/5.0058766
  9. Neese, F.
     The SHARK Integral Generation and Digestion System
     J. Comp. Chem. 2022 44(3), 381
     doi.org/10.1002/jcc.26942

List of suggested additional citations. These are papers that are important in the
'surrounding' of
of this run, or papers that preceded the highly important papers. If you like your results we
are grateful for a citation.

  1. Izsak, R.; Neese, F.
     An overlap fitted chain of spheres exchange method
     J. Chem. Phys. 2011 135 , 144105
     doi.org/10.1063/1.3646921
  2. Izsak, R.; Hansen, A.; Neese, F.
     The resolution of identity and chain of spheres approximations for the LPNO-CCSD singles
Fock term
     Molec. Phys. 2012 110 , 2413-2417
     doi.org/10.1080/00268976.2012.687466
  3. Neese, F.
     The ORCA program system
     WIRES Comput. Molec. Sci. 2012 2(1), 73-78
     doi.org/10.1002/wcms.81
  4. Izsak, R.; Neese, F.; Klopper, W.
     Robust fitting techniques in the chain of spheres approximation to the Fock exchange: The
role of the complementary space
     J. Chem. Phys. 2013 139 ,
     doi.org/10.1063/1.4819264
  5. Neese, F.
     Software update: the ORCA program system, version 4.0
     WIRES Comput. Molec. Sci. 2018 8(1), 1-6
     doi.org/10.1002/wcms.1327
  6. Neese, F.; Wennmohs, F.; Becker, U.; Riplinger, C.
     The ORCA quantum chemistry program package
     J. Chem. Phys. 2020 152(22), 224108
     doi.org/10.1063/5.0004608
  7. Neese, F.
     Software update: The ORCA program system—Version 5.0
     WIRES Comput. Molec. Sci. 2022 12(1), e1606
     doi.org/10.1002/wcms.1606

List of optional additional citations

  1. Neese, F.
     Approximate second-order SCF convergence for spin unrestricted wavefunctions
     Chem. Phys. Lett. 2000 325(1-3), 93-98
     doi.org/10.1016/s0009-2614(00)00662-x

Timings for individual modules:

Sum of individual times          ...   126533.131 sec (=2108.886 min)
Startup calculation              ...     3418.949 sec (=  56.982 min)   2.7 %
SCF iterations                   ...    74664.628 sec (=1244.410 min)  59.0 %
Property calculations            ...        0.413 sec (=   0.007 min)   0.0 %
SCF Gradient evaluation          ...    48449.142 sec (= 807.486 min)  38.3 %
                             ****ORCA TERMINATED NORMALLY****
TOTAL RUN TIME: 1 days 11 hours 26 minutes 19 seconds 708 msec
'Cl-IRC.bibtex' -> '/hsnfs/users/barasits/Tunneling_selenoxide/Cl/IRC/Cl-IRC.bibtex'
'Cl-IRC.cpcm' -> '/hsnfs/users/barasits/Tunneling_selenoxide/Cl/IRC/Cl-IRC.cpcm'
```

```
'Cl-IRC.cpcm_corr' -> '/hsnfs/users/barasits/Tunneling_selenoxide/Cl/IRC/Cl-IRC.cpcm_corr'
'Cl-IRC.densities' -> '/hsnfs/users/barasits/Tunneling_selenoxide/Cl/IRC/Cl-IRC.densities'
'Cl-IRC.densitiesinfo' -> '/hsnfs/users/barasits/Tunneling_selenoxide/Cl/IRC/Cl-
IRC.densitiesinfo'
'Cl-IRC.engrad' -> '/hsnfs/users/barasits/Tunneling_selenoxide/Cl/IRC/Cl-IRC.engrad'
'Cl-IRC.err' -> '/hsnfs/users/barasits/Tunneling_selenoxide/Cl/IRC/Cl-IRC.err'
'Cl-IRC.gbw' -> '/hsnfs/users/barasits/Tunneling_selenoxide/Cl/IRC/Cl-IRC.gbw'
'Cl-IRC_IRC_B.cpcm' -> '/hsnfs/users/barasits/Tunneling_selenoxide/Cl/IRC/Cl-IRC_IRC_B.cpcm'
'Cl-IRC_IRC_B.cpcm_corr' -> '/hsnfs/users/barasits/Tunneling_selenoxide/Cl/IRC/Cl-
IRC_IRC_B.cpcm_corr'
'Cl-IRC_IRC_B.densities' -> '/hsnfs/users/barasits/Tunneling_selenoxide/Cl/IRC/Cl-
IRC_IRC_B.densities'
'Cl-IRC_IRC_B.densitiesinfo' -> '/hsnfs/users/barasits/Tunneling_selenoxide/Cl/IRC/Cl-
IRC_IRC_B.densitiesinfo'
'Cl-IRC_IRC_B.gbw' -> '/hsnfs/users/barasits/Tunneling_selenoxide/Cl/IRC/Cl-IRC_IRC_B.gbw'
'Cl-IRC_IRC_B.smd' -> '/hsnfs/users/barasits/Tunneling_selenoxide/Cl/IRC/Cl-IRC_IRC_B.smd'
'Cl-IRC_IRC_B.smd.grd' -> '/hsnfs/users/barasits/Tunneling_selenoxide/Cl/IRC/Cl-
IRC_IRC_B.smd.grd'
'Cl-IRC_IRC_B_trj.xyz' -> '/hsnfs/users/barasits/Tunneling_selenoxide/Cl/IRC/Cl-
IRC_IRC_B_trj.xyz'
'Cl-IRC_IRC_B.xyz' -> '/hsnfs/users/barasits/Tunneling_selenoxide/Cl/IRC/Cl-IRC_IRC_B.xyz'
'Cl-IRC_IRC_F.cpcm' -> '/hsnfs/users/barasits/Tunneling_selenoxide/Cl/IRC/Cl-IRC_IRC_F.cpcm'
'Cl-IRC_IRC_F.cpcm_corr' -> '/hsnfs/users/barasits/Tunneling_selenoxide/Cl/IRC/Cl-
IRC_IRC_F.cpcm_corr'
'Cl-IRC_IRC_F.densities' -> '/hsnfs/users/barasits/Tunneling_selenoxide/Cl/IRC/Cl-
IRC_IRC_F.densities'
'Cl-IRC_IRC_F.densitiesinfo' -> '/hsnfs/users/barasits/Tunneling_selenoxide/Cl/IRC/Cl-
IRC_IRC_F.densitiesinfo'
'Cl-IRC_IRC_F.gbw' -> '/hsnfs/users/barasits/Tunneling_selenoxide/Cl/IRC/Cl-IRC_IRC_F.gbw'
'Cl-IRC_IRC_F.smd' -> '/hsnfs/users/barasits/Tunneling_selenoxide/Cl/IRC/Cl-IRC_IRC_F.smd'
'Cl-IRC_IRC_F.smd.grd' -> '/hsnfs/users/barasits/Tunneling_selenoxide/Cl/IRC/Cl-
IRC_IRC_F.smd.grd'
'Cl-IRC_IRC_F_trj.xyz' -> '/hsnfs/users/barasits/Tunneling_selenoxide/Cl/IRC/Cl-
IRC_IRC_F_trj.xyz'
'Cl-IRC_IRC_Full_trj.xyz' -> '/hsnfs/users/barasits/Tunneling_selenoxide/Cl/IRC/Cl-
IRC_IRC_Full_trj.xyz'
'Cl-IRC_IRC_F.xyz' -> '/hsnfs/users/barasits/Tunneling_selenoxide/Cl/IRC/Cl-IRC_IRC_F.xyz'
'Cl-IRC.job' -> '/hsnfs/users/barasits/Tunneling_selenoxide/Cl/IRC/Cl-IRC.job'
'Cl-IRC.property.txt' -> '/hsnfs/users/barasits/Tunneling_selenoxide/Cl/IRC/Cl-
IRC.property.txt'
'Cl-IRC.smd' -> '/hsnfs/users/barasits/Tunneling_selenoxide/Cl/IRC/Cl-IRC.smd'
'Cl-IRC.smd.grd' -> '/hsnfs/users/barasits/Tunneling_selenoxide/Cl/IRC/Cl-IRC.smd.grd'
'TS-Cl_Compound_2.hess' -> '/hsnfs/users/barasits/Tunneling_selenoxide/Cl/IRC/TS-
Cl_Compound_2.hess'
cp: cannot stat 'Cl-IRC.asa.inp': No such file or directory
cp: missing destination file operand after '/hsnfs/users/barasits/Tunneling_selenoxide/Cl/IRC/'
Try 'cp --help' for more information.
cp: cannot stat 'Cl-IRC.pc.tmp': No such file or directory
cp: cannot stat 'Cl-IRC.QM2.pc.tmp': No such file or directory
```

IRC output for R = CF_3

```
! Job execution by suborca V 10.06 !
Job started from head2.mpi-muelheim.mpg.de, running
/hsnfs/software/orca/orca_current_directory/orca_current_20251216/orca on xe30th97.mpi-
muelheim.mpg.de in /usr/scratch/barasits/CF3-IRC__26969142 (/bin/bash)
Job execution start: Tue Dec 16 12:14:18 PM CET 2025
Shared library path: /hsnfs/software/openmpi-
4.1.0/lib:/hsnfs/software/orca/orca_current_directory/orca_current_20251216:/hsnfs/software/the
mis/llvm-14.0.1/lib:/hsnfs/software/orca/orca_stable:/usr/users/openmpi-
2.0.4/lib:/opt/intel/lib/intel64:/opt/intel/mkl/lib/intel64
SLURM Job ID is: 26969142
SLURM_JOB_NODELIST: xe30th97
Maxcore enforced by SLURM: 9600 MB per core
Expanded nodelist (1 nodes):
xe30th97

                                 *****************
                                 * O   R   C   A *
                                 *****************

                                            #,
                                            ###
                                            ####
                                            #####
                                            ######
                                           ########,
                                     ,,################,,,,,
                               ,,#################################,,
                          ,,##########################################,,
                       ,#########################################, ''#####,
                    ,#############################################,,   '####,
                  ,##################################################,,,,####,
                ,###########''''           ''''###############################
              ,#####''   ,,,,##########,,,,          '''####'''          '####
            ,##' ,,,,###########################,,,                        '##
           ' ,,###''''                  '''############,,,
         ,,##''                                '''############,,,,        ,,,,,,###''
      ,#''                                            '''#######################'''
     '                                                          ''''####''''
             ,#######,   #######,   ,#######,      ##
            ,#'     '#,  ##    ##  ,#'     '#,    #''#        ,####,    ,#,
            ##       ##  ##   ,#'  ##            #'  '#       #'       ,# #
            ##       ##  #######   ##           ,######,      #####,      #
            '#,     ,#'  ##    ##  '#,     ,#' ,#      #,     #,   #      #
             '#######'   ##     ##  '#######'  #'      '#     '####' #    #



                #####################################################
                #                        -***-                      #
                #          Department of theory and spectroscopy    #
                #                                                   #
                #                      Frank Neese                  #
                #                                                   #
                #     Directorship, Architecture, Infrastructure    #
                #                    SHARK, DRIVERS                 #
                #        Core code/Algorithms in most modules       #
                #                                                   #
                #        Max Planck Institute fuer Kohlenforschung  #
                #                Kaiser Wilhelm Platz 1             #
                #                 D-45470 Muelheim/Ruhr             #
                #                      Germany                      #
                #                                                   #
                #                  All rights reserved              #
```

```
                #                         -***-                            #
                ##########################################################


                         Program Version 6.1.x  -  CURRENT   -
                                (GIT: $2a5ddc4e$)
                ($2025-12-15 18:08:28 +0100 $)

                  !                      WARNING:                          !
                  !         This is a DEVELOPMENT version                  !

                         (BUILD: $Tue 16 Dec 2025 12:05:34 AM CET$)


 With contributions from (in alphabetic order):
[Max-Planck-Institut fuer Kohlenforschung]
  Daniel Aravena         : Magnetic Suceptibility
  Michael Atanasov       : Ab Initio Ligand Field Theory (pilot matlab implementation)
  Alexander A. Auer      : GIAO ZORA, VPT2 properties, NMR spectrum
  Ute Becker             : All parallelization in ORCA, NUMFREQ, NUMCALC
  Giovanni Bistoni       : ED, misc. LED, open-shell LED, HFLD
  Dmytro Bykov           : pre 5.0 version of the SCF Hessian
  Marcos Casanova-Páez   : Triplet and SCS-CIS(D). UHF-(DLPNO)-IP/EA/STEOM-CCSD. UHF-CVS-
IP/STEOM-CCSD
  Vijay G. Chilkuri      : MRCI spin determinant printing, contributions to CSF-ICE
  Pauline Colinet        : FMM embedding
  Dipayan Datta          : RHF DLPNO-CCSD density
  Achintya Kumar Dutta   : EOM-CC, STEOM-CC
  Nicolas Foglia         : Exact transition moments, OPA infrastructure, MCD improvements
  Dmitry Ganyushin       : Spin-Orbit,Spin-Spin,Magnetic field MRCI
  Miquel Garcia-Rates    : C-PCM and meta-GGA Hessian, CCSD/C-PCM, Gaussian charge scheme
  Tiago L. C. Gouveia    : GS-ROHF, GS-ROCIS
  Yang Guo               : DLPNO-NEVPT2, F12-NEVPT2, CIM, IAO-localization
  Andreas Hansen         : Spin unrestricted coupled pair/coupled cluster methods
  Ingolf Harden          : AUTO-CI MPn and infrastructure
  Benjamin Helmich-Paris : MC-RPA, TRAH-(SCF,CASSCF), AVAS, COSX integrals, SCF dyn. polar.,
MC-PDFT, srDFT
  Lee Huntington         : MR-EOM, pCC
  Robert Izsak           : Overlap fitted RIJCOSX, COSX-SCS-MP3, EOM
  Riya Kayal             : Wick's Theorem for AUTO-CI, AUTO-CI UHF-CCSDT
  Emily Kempfer          : AUTO-CI RHF CISDT and CCSDT, approximate NEVPT4
  Christian Kollmar      : KDIIS, OOCD, Brueckner-CCSD(T), CCSD density, CASPT2, CASPT2-K,
improved NEVPT2
  Axel Koslowski         : Symmetry handling
  Simone Kossmann        : meta-GGA functionals, TD-DFT gradient, OOMP2, (MP2 Hessian;
deprecated post 5.0)
  Lucas Lang             : DCDCAS, Hyperfine gauge corrections, ICE-SOC+SSC
  Marvin Lechner         : AUTO-CI (C++ implementation), FIC-MRCC
  Spencer Leger          : CASSCF response
  Dagmar Lenk            : GEPOL surface, SMD, ORCA-2-JSON
  Dimitrios Liakos       : Extrapolation schemes; Compound Job, Property file
  Dimitrios Manganas     : Further ROCIS development; embedding schemes. LFT, Crystal Embedding
  Hagen Neugebauer       : wr2SCAN, Native XTB
  Dimitrios Pantazis     : SARC Basis sets
  Anastasios Papadopoulos: AUTO-CI, single reference methods and gradients
  Taras Petrenko         : pre 6.0 DFT Hessian and TD-DFT gradient,  ECA, NRVS
  Petra Pikulova         : Analytic Raman intensities
  Peter Pinski           : DLPNO-MP2, DLPNO-MP2 Gradient
  Shashank Vittal Rao    : ES-AILFT, MagRelax
  Christoph Reimann      : Effective Core Potentials
  Marius Retegan         : Local ZFS, SOC
  Christoph Riplinger    : Optimizer, TS searches, QM/MM, DLPNO-CCSD(T), (RO)-DLPNO pert.
Triples
```

```
  Michael Roemelt        : Original ROCIS implementation, recursive CI coupling coefficients
  Masaaki Saitow         : Open-shell DLPNO-CCSD energy and density
  Barbara Sandhoefer     : DKH picture change effects
  Yorick L. A. Schmerwitz: GMF and freeze-and-release deltaSCF, NEB S-IDPP initial path
  Kantharuban Sivalingam : CASSCF convergence/infrastructure, NEVPT2, NEVPT3, NEVPT4(SD), FIC-
MRCI and CEPA variants
  Bernardo de Souza      : ESD, SOC TD-DFT
  Georgi L. Stoychev     : AutoAux, RI-MP2 NMR, DLPNO-MP2 response, X2C
  Van Anh Tran           : RI-MP2 g-tensors
  Willem Van den Heuvel  : Paramagnetic NMR
  Zikuan Wang            : NOTCH, Electric field optimization
  Frank Wennmohs         : Technical directorship and infrastructure
  Hang Xu                : AUTO-CI-Response properties

[FACCTs GmbH]
  Markus Bursch, Nicolas Foglia, Miquel Garcia-Rates, Ingolf Harden, Hagen Neugebauer,
Anastasios Papadopoulos,
  Christoph Riplinger, Bernardo de Souza, Georgi L. Stoychev

  APM, various basis sets, CI-OPT, improved COSX, DLPNO-Multilevel,
  DOCKER, DRACO, updates on ESD, Fragmentator, GOAT, IRC, LR-CPCM, L-BFGS, MBIS, meta-GGA TD-
DFT gradient, ML-optimized integration grids,
  MM, NACMEs, nearIR, NEB, NEB-TS, NL-DFT gradient (VV10), 2- and 3-layer-ONIOM, interface
openCOSMO-RS, QMMM,
  Crystal-QMMM, RESP, rigid body optimization, SF, symmetry and pop. for TD-DFT, various
functionals, SOLVATOR

[Other institutions]
  V. Asgeirsson          : NEB
  Christoph Bannwarth    : sTDA-DFT, sTD-DFT, PBEh-3c, B97-3c, D3
  Giovanni Bistoni       : ETS/NOCV, ADLD/ADEX, COVALED
  Martin Brehm           : Molecular dynamics
  Ronald Cardenas        : ETS/NOCV
  Martina Colucci        : COVALED
  Sebastian Ehlert       : rSCAN, r2SCAN, r2SCAN-3c, D4, dhf basis sets
  Marvin Friede          : D4 for Fr, Ra, Ac-Lr
  Lars Goerigk           : TD-DFT with DH, B97 family of functionals
  Stefan Grimme          : VdW corrections, initial TS optimization, DFT functionals, gCP,
sTDA/sTD-DF
  Waldemar Hujo          : DFT-NL
  H. Jonsson             : NEB
  Holger Kruse           : gCP
  Marcel Mueller         : wB97X-3c, vDZP basis set
  Gianluca Regni         : ADLD/ADEX
  Tobias Risthaus        : pre 6.0 range-separated hybrid DFT and stability analysis
  Lukas Wittmann         : regularized MP2, r2SCAN double-hybrids, wr2SCAN

We gratefully acknowledge several colleagues who have allowed us to
interface, adapt or use parts of their codes:
  Ed Valeev, F. Pavosevic, A. Kumar             : LibInt (2-el integral package), F12 methods
  Garnet Chan, S. Sharma, J. Yang, R. Olivares  : DMRG
  Ulf Ekstrom                                   : XCFun DFT Library
  Mihaly Kallay                                 : mrcc  (arbitrary order and MRCC methods)
  Frank Weinhold                                : gennbo (NPA and NBO analysis)
  Simon Mueller                                 : openCOSMO-RS
  Christopher J. Cramer and Donald G. Truhlar   : smd solvation model
  S Lehtola, MJT Oliveira, MAL Marques          : LibXC Library
  Liviu Ungur et al                             : ANISO software


 Your calculation uses the libint2 library for the computation of 2-el integrals
 For citations please refer to: http://libint.valeyev.net
```

```
 Your ORCA version has been built with support for libXC version: 7.0.0
 For citations please refer to: https://libxc.gitlab.io

 This ORCA versions uses:
   CBLAS   interface :  Fast vector & matrix operations
   LAPACKE interface :  Fast linear algebra routines
   SCALAPACK package :  Parallel linear algebra routines
   Shared memory     :  Shared parallel matrices
   BLAS/LAPACK       :  OpenBLAS 0.3.26  USE64BITINT DYNAMIC_ARCH NO_AFFINITY Haswell
SINGLE_THREADED
        Core in use  :  Haswell
   Copyright (c) 2011-2014, The OpenBLAS Project


  ***********************************
  * Starting time: Tue Dec 16 12:14:18 2025
  * Host name:     xe30th97.mpi-muelheim.mpg.de
  * Process ID:    328672
  * Working dir.:  /usr/scratch/barasits/CF3-IRC__26969142
  ***********************************

NOTE: MaxCore=8000 MB was set to SCF,MP2,MDCI,CIPSI,MRCI,RASCI and CIS
      => If you want to overwrite this, your respective input block should be placed after the
MaxCore statement
Your calculation utilizes the atom-pairwise dispersion correction
with the Becke-Johnson damping scheme (D3BJ)


Your calculation utilizes the SMD solvation module
Please cite in your paper:
Marenich, A. V.; Cramer, C. J.; Truhlar, D. G. J. Phys. Chem. B 2009, 113, 6378

================================================================================

----- Orbital basis set information -----
Your calculation utilizes the basis: def2-TZVP
   F. Weigend and R. Ahlrichs, Phys. Chem. Chem. Phys. 7, 3297 (2005).

----- AuxJ basis set information -----
Your calculation utilizes the auxiliary basis: def2/J
   H-Rn: F. Weigend, Phys. Chem. Chem. Phys. 8, 1057 (2006).
   Fr-Lr: K. Eichkorn, F. Weigend, O. Treutler, R. Ahlrichs; Theor. Chem. Acc. 97, 119 (1997).

================================================================================
                                        WARNINGS
                       Please study these warnings very carefully!
================================================================================

WARNING: SinglePoint found together with IRC
         Switching on EnGrad - energy plus gradient calculation

================================================================================
                                       INPUT FILE
================================================================================
NAME = CF3-IRC.inp
|  1> #
|  2> #IRC LQA
|  3> #
|  4> ! irc B3LYP D3BJ def2-TZVP def2/J verytightscf
|  5> %pal nprocs 12 end
|  6> %maxcore 8000
|  7> %irc
|  8> printlevel 1
```

```
|  9> method lqa
| 10> maxiter 241
| 11> tolrmsg 1.e-5 # Tighter convergence criteria
| 12> tolmaxg 8.e-5 # Tighter convergence criteria
| 13> follow_coordtype massweighted
| 14> inithess read
| 15> hess_filename "TS-CF3_Compound_3.hess"
| 16> lqahess lqacalc_bofillupdate
| 17> init_displ length
| 18> scale_init_displ 0.04
| 19> adapt_scale_displ false
| 20> scale_displ_sd 0.11
| 21> ENFORCESTRICTCONVERGENCE true
| 22> REPORTPROFILE true
| 23> BSCORRECTOR true
| 24> BSRICHTHRESH 1E-6
| 25> INITBSCORRECTOR false
| 26> end
| 27> %CPCM SMD true
| 28>      SMDSOLVENT "TETRAHYDROFURAN" END
| 29>
| 30> * xyz 0 1
| 31>   C          -1.77440141462015      0.38880370329309     -6.06583304245525
| 32>   C          -1.20810943770561     -0.28366186751805     -4.98309124407938
| 33>   C          -1.01539420751738      1.37312924022124     -6.70426338060164
| 34>   C           0.07639753979557      0.01996259293240     -4.54503705739509
| 35>   C           0.27224717059325      1.67472178581823     -6.27615819646588
| 36>   C           0.82297314896890      0.99796272366582     -5.19201240131108
| 37>   H          -1.77979858432027     -1.05396371748399     -4.47947594317359
| 38>   H          -1.44211230494851      1.90956759046781     -7.54403280134238
| 39>   H           0.49694700418455     -0.51358933152937     -3.70281273515429
| 40>   H           0.84456963561391      2.44040289599028     -6.78575299408253
| 41>   H           1.82574438315274      1.22940359492299     -4.85599303707047
| 42>   C          -3.16259896440199      0.06055601548115     -6.54662712499949
| 43>   H          -3.64707156286316      0.98591764897404     -6.87315367941293
| 44>   H          -3.75323518669903     -0.32122423512253     -5.71114795888799
| 45>   C          -3.23707265582239     -0.94688403857286     -7.70617187520571
| 46>   H          -3.13012415200923     -2.13634554935976     -7.17030207070386
| 47>   H          -4.24301102645347     -1.03211063592001     -8.11887759845052
| 48>   C          -2.21066380945580     -1.04769863232636     -8.66348697647831
| 49>   H          -2.44273379781999     -1.31502534701977     -9.68536500351479
| 50>   H          -1.25138149266883     -0.56913208142032     -8.51822201110182
| 51>   Se         -1.42010039467490     -3.38740527945077     -8.11690912202046
| 52>   O          -2.70517407172786     -3.37423207068231     -6.97934902588030
| 53>   C           0.10453386127675     -2.97872298820515     -6.99677347959807
| 54>   C           0.13984013346247     -3.43842210951100     -5.68490850605044
| 55>   C           1.18636634504793     -2.29182702756934     -7.53776376543200
| 56>   C           1.26925720044759     -3.21767322539947     -4.91101476948073
| 57>   C           2.31616813253630     -2.07090358061916     -6.76269000799637
| 58>   C           2.35922380251404     -2.54270696028363     -5.45400114790860
| 59>   H          -0.71144294186167     -3.96088358867847     -5.27036838986168
| 60>   H           1.15148483159383     -1.92364586419707     -8.55517482609896
| 61>   H           1.30009145051062     -3.57312645001833     -3.89009986150516
| 62>   H           3.15579143563586     -1.52937485108016     -7.17670543498727
| 63>   C           3.56087353922682     -2.25960695204582     -4.60377410489179
| 64>   F           3.46399848238418     -1.06497084191313     -3.96196264363380
| 65>   F           3.74675899247295     -3.18829399210950     -3.64154029027094
| 66>   F           4.70354185615198     -2.20668080373060     -5.32265587249577
| 67> *
| 68>
| 69>
| 70>                          ****END OF INPUT****
================================================================================
```

```
                    ******************************
                    * Energy+Gradient Calculation *
                    ******************************

---------------------------------
CARTESIAN COORDINATES (ANGSTROEM)
---------------------------------
  C     -1.774401    0.388804   -6.065833
  C     -1.208109   -0.283662   -4.983091
  C     -1.015394    1.373129   -6.704263
  C      0.076398    0.019963   -4.545037
  C      0.272247    1.674722   -6.276158
  C      0.822973    0.997963   -5.192012
  H     -1.779799   -1.053964   -4.479476
  H     -1.442112    1.909568   -7.544033
  H      0.496947   -0.513589   -3.702813
  H      0.844570    2.440403   -6.785753
  H      1.825744    1.229404   -4.855993
  C     -3.162599    0.060556   -6.546627
  H     -3.647072    0.985918   -6.873154
  H     -3.753235   -0.321224   -5.711148
  C     -3.237073   -0.946884   -7.706172
  H     -3.130124   -2.136346   -7.170302
  H     -4.243011   -1.032111   -8.118878
  C     -2.210664   -1.047699   -8.663487
  H     -2.442734   -1.315025   -9.685365
  H     -1.251381   -0.569132   -8.518222
  Se    -1.420100   -3.387405   -8.116909
  O     -2.705174   -3.374232   -6.979349
  C      0.104534   -2.978723   -6.996773
  C      0.139840   -3.438422   -5.684909
  C      1.186366   -2.291827   -7.537764
  C      1.269257   -3.217673   -4.911015
  C      2.316168   -2.070904   -6.762690
  C      2.359224   -2.542707   -5.454001
  H     -0.711443   -3.960884   -5.270368
  H      1.151485   -1.923646   -8.555175
  H      1.300091   -3.573126   -3.890100
  H      3.155791   -1.529375   -7.176705
  C      3.560874   -2.259607   -4.603774
  F      3.463998   -1.064971   -3.961963
  F      3.746759   -3.188294   -3.641540
  F      4.703542   -2.206681   -5.322656

----------------------------
CARTESIAN COORDINATES (A.U.)
----------------------------
  NO LB      ZA    FRAG     MASS         X           Y           Z
   0 C     6.0000    0    12.011   -3.353133    0.734733  -11.462763
   1 C     6.0000    0    12.011   -2.282996   -0.536043   -9.416678
   2 C     6.0000    0    12.011   -1.918817    2.594838  -12.669222
   3 C     6.0000    0    12.011    0.144370    0.037724   -8.588875
   4 C     6.0000    0    12.011    0.514473    3.164766  -11.860220
   5 C     6.0000    0    12.011    1.555194    1.885876   -9.811482
   6 H     1.0000    0     1.008   -3.363332   -1.991703   -8.464983
   7 H     1.0000    0     1.008   -2.725197    3.608560  -14.256156
   8 H     1.0000    0     1.008    0.939094   -0.970543   -6.997302
   9 H     1.0000    0     1.008    1.596005    4.611693  -12.823215
  10 H     1.0000    0     1.008    3.450157    2.323236   -9.176497
  11 C     6.0000    0    12.011   -5.976446    0.114434  -12.371332
  12 H     1.0000    0     1.008   -6.891966    1.863114  -12.988378
  13 H     1.0000    0     1.008   -7.092587   -0.607026  -10.792506
```

```
  14 C      6.0000    0    12.011   -6.117181   -1.789352  -14.562554
  15 H      1.0000    0     1.008   -5.915077   -4.037108  -13.549907
  16 H      1.0000    0     1.008   -8.018129   -1.950406  -15.342455
  17 C      6.0000    0    12.011   -4.177549   -1.979863  -16.371618
  18 H      1.0000    0     1.008   -4.616098   -2.485038  -18.302687
  19 H      1.0000    0     1.008   -2.364768   -1.075504  -16.097107
  20 Se    34.0000    0    78.960   -2.683601   -6.401268  -15.338735
  21 O      8.0000    0    15.999   -5.112038   -6.376375  -13.189058
  22 C      6.0000    0    12.011    0.197540   -5.628971  -13.221986
  23 C      6.0000    0    12.011    0.264260   -6.497676  -10.742920
  24 C      6.0000    0    12.011    2.241907   -4.330925  -14.244309
  25 C      6.0000    0    12.011    2.398549   -6.080521   -9.280473
  26 C      6.0000    0    12.011    4.376923   -3.913441  -12.779632
  27 C      6.0000    0    12.011    4.458287   -4.805020  -10.306569
  28 H      1.0000    0     1.008   -1.344432   -7.484985   -9.959553
  29 H      1.0000    0     1.008    2.175991   -3.635164  -16.166937
  30 H      1.0000    0     1.008    2.456817   -6.752230   -7.351223
  31 H      1.0000    0     1.008    5.963582   -2.890100  -13.562008
  32 C      6.0000    0    12.011    6.729076   -4.270038   -8.699872
  33 F      9.0000    0    18.998    6.546008   -2.012503   -7.487024
  34 F      9.0000    0    18.998    7.080348   -6.025002   -6.881514
  35 F      9.0000    0    18.998    8.888406   -4.170022  -10.058362
```

```
--------------------------------
INTERNAL COORDINATES (ANGSTROEM)
--------------------------------
 C      0   0   0     0.000000000000     0.00000000     0.00000000
 C      1   0   0     1.394713715783     0.00000000     0.00000000
 C      1   2   0     1.397348202757   118.28029213     0.00000000
 C      2   1   3     1.390696752964   120.94998993     0.26688934
 C      3   1   2     1.390054830165   121.01630854     0.15379199
 C      4   2   1     1.390121034861   120.20203715   359.48782892
 H      2   1   3     1.083430566176   119.30217462   180.35024587
 H      3   1   2     1.084005128356   119.25657253   180.34072348
 H      4   2   1     1.082072694909   119.76821193   179.98261487
 H      5   3   1     1.083285476779   119.85935267   179.91313937
 H      6   4   2     1.082600590310   120.22554026   180.46201752
 C      1   2   3     1.505324526999   121.14799939   179.52532975
 H     12   1   2     1.094361659662   108.61262683   143.73801771
 H     12   1   2     1.092077260933   109.30746151    27.88814470
 C     12   1   2     1.537864045017   115.36159757   264.98552597
 H     15  12   1     1.308974069830   106.41842951    81.43966301
 H     15  12   1     1.090642742791   112.40639913   188.96225667
 C     15  12   1     1.407171242365   121.63881709   329.73053983
 H     18  15  12     1.081459548031   120.26605751   212.46724041
 H     18  15  12     1.081827350880   121.58377127    12.26295255
 Se    16  15  12     2.320645730935   112.52551484   234.32302397
 O     16  15  12     1.322652137083   159.36015620   228.46820820
 C     21  16  15     1.935519229309   103.32939720    91.34430467
 C     23  21  16     1.390524909452   119.74789082    69.03378673
 C     23  21  16     1.390991758799   119.46066318   245.44112717
 C     24  23  21     1.386803697910   119.63866870   175.91328341
 C     25  23  21     1.387803508386   119.64040592   184.05846071
 C     27  25  23     1.391804135753   119.75939683     0.26032397
 H     24  23  21     1.081430709513   120.09075892   355.62074349
 H     25  23  21     1.082543003954   120.55831672     4.42814326
 H     26  24  23     1.081464282903   119.84788945   179.95749634
 H     27  25  23     1.081494072943   119.86349809   180.55894549
 C     28  27  25     1.498997574742   119.61063171   182.19206100
 F     33  28  27     1.359581613613   112.12210440    86.99223446
 F     33  28  27     1.350150707925   112.62485048   206.61299345
 F     33  28  27     1.351030368080   112.55668227   327.47624779
```

```
---------------------------
INTERNAL COORDINATES (A.U.)
---------------------------
 C      0   0   0     0.000000000000     0.00000000     0.00000000
 C      1   0   0     2.635626958054     0.00000000     0.00000000
 C      1   2   0     2.640605416938   118.28029213     0.00000000
 C      2   1   3     2.628035998436   120.94998993     0.26688934
 C      3   1   2     2.626822940147   121.01630854     0.15379199
 C      4   2   1     2.626948048890   120.20203715   359.48782892
 H      2   1   3     2.047387055192   119.30217462   180.35024587
 H      3   1   2     2.048472820359   119.25657253   180.34072348
 H      4   2   1     2.044821050373   119.76821193   179.98261487
 H      5   3   1     2.047112875966   119.85935267   179.91313937
 H      6   4   2     2.045818628107   120.22554026   180.46201752
 C      1   2   3     2.844651098702   121.14799939   179.52532975
 H     12   1   2     2.068043828224   108.61262683   143.73801771
 H     12   1   2     2.063726940246   109.30746151    27.88814470
 C     12   1   2     2.906141876287   115.36159757   264.98552597
 H     15  12   1     2.473602508384   106.41842951    81.43966301
 H     15  12   1     2.061016093823   112.40639913   188.96225667
 C     15  12   1     2.659168271599   121.63881709   329.73053983
 H     18  15  12     2.043662370693   120.26605751   212.46724041
 H     18  15  12     2.044357417349   121.58377127    12.26295255
 Se    16  15  12     4.385384885320   112.52551484   234.32302397
 O     16  15  12     2.499450309533   159.36015620   228.46820820
 C     21  16  15     3.657601270332   103.32939720    91.34430467
 C     23  21  16     2.627711261260   119.74789082    69.03378673
 C     23  21  16     2.628593478672   119.46066318   245.44112717
 C     24  23  21     2.620679190558   119.63866870   175.91328341
 C     25  23  21     2.622568558545   119.64040592   184.05846071
 C     27  25  23     2.630128648631   119.75939683     0.26032397
 H     24  23  21     2.043607873791   120.09075892   355.62074349
 H     25  23  21     2.045709805666   120.55831672     4.42814326
 H     26  24  23     2.043671318305   119.84788945   179.95749634
 H     27  25  23     2.043727613321   119.86349809   180.55894549
 C     28  27  25     2.832694891675   119.61063171   182.19206100
 F     33  28  27     2.569236906442   112.12210440    86.99223446
 F     33  28  27     2.551415077498   112.62485048   206.61299345
 F     33  28  27     2.553077394281   112.55668227   327.47624779

---------------------
BASIS SET INFORMATION
---------------------
There are 5 groups of distinct atoms

 Group   1 Type C   : 11s6p2d1f contracted to 5s3p2d1f pattern {62111/411/11/1}
 Group   2 Type H   : 5s1p contracted to 3s1p pattern {311/1}
 Group   3 Type Se  : 17s13p8d1f contracted to 6s5p4d1f pattern {842111/64111/5111/1}
 Group   4 Type O   : 11s6p2d1f contracted to 5s3p2d1f pattern {62111/411/11/1}
 Group   5 Type F   : 11s6p2d1f contracted to 5s3p2d1f pattern {62111/411/11/1}

Atom   0C    basis set group =>   1
Atom   1C    basis set group =>   1
Atom   2C    basis set group =>   1
Atom   3C    basis set group =>   1
Atom   4C    basis set group =>   1
Atom   5C    basis set group =>   1
Atom   6H    basis set group =>   2
Atom   7H    basis set group =>   2
Atom   8H    basis set group =>   2
Atom   9H    basis set group =>   2
Atom  10H    basis set group =>   2
Atom  11C    basis set group =>   1
```

```
Atom  12H    basis set group =>   2
Atom  13H    basis set group =>   2
Atom  14C    basis set group =>   1
Atom  15H    basis set group =>   2
Atom  16H    basis set group =>   2
Atom  17C    basis set group =>   1
Atom  18H    basis set group =>   2
Atom  19H    basis set group =>   2
Atom  20Se   basis set group =>   3
Atom  21O    basis set group =>   4
Atom  22C    basis set group =>   1
Atom  23C    basis set group =>   1
Atom  24C    basis set group =>   1
Atom  25C    basis set group =>   1
Atom  26C    basis set group =>   1
Atom  27C    basis set group =>   1
Atom  28H    basis set group =>   2
Atom  29H    basis set group =>   2
Atom  30H    basis set group =>   2
Atom  31H    basis set group =>   2
Atom  32C    basis set group =>   1
Atom  33F    basis set group =>   5
Atom  34F    basis set group =>   5
Atom  35F    basis set group =>   5
---------------------------------
AUXILIARY/J BASIS SET INFORMATION
---------------------------------
There are 5 groups of distinct atoms

 Group   1 Type C   : 12s5p4d2f1g contracted to 6s4p3d1f1g pattern {711111/2111/211/2/1}
 Group   2 Type H   : 5s2p1d contracted to 3s1p1d pattern {311/2/1}
 Group   3 Type Se  : 19s5p5d3f1g contracted to 8s4p3d2f1g pattern {121111111/2111/311/21/1}
 Group   4 Type O   : 12s5p4d2f1g contracted to 6s4p3d1f1g pattern {711111/2111/211/2/1}
 Group   5 Type F   : 12s5p4d2f1g contracted to 6s4p3d1f1g pattern {711111/2111/211/2/1}

Atom   0C    basis set group =>   1
Atom   1C    basis set group =>   1
Atom   2C    basis set group =>   1
Atom   3C    basis set group =>   1
Atom   4C    basis set group =>   1
Atom   5C    basis set group =>   1
Atom   6H    basis set group =>   2
Atom   7H    basis set group =>   2
Atom   8H    basis set group =>   2
Atom   9H    basis set group =>   2
Atom  10H    basis set group =>   2
Atom  11C    basis set group =>   1
Atom  12H    basis set group =>   2
Atom  13H    basis set group =>   2
Atom  14C    basis set group =>   1
Atom  15H    basis set group =>   2
Atom  16H    basis set group =>   2
Atom  17C    basis set group =>   1
Atom  18H    basis set group =>   2
Atom  19H    basis set group =>   2
Atom  20Se   basis set group =>   3
Atom  21O    basis set group =>   4
Atom  22C    basis set group =>   1
Atom  23C    basis set group =>   1
Atom  24C    basis set group =>   1
Atom  25C    basis set group =>   1
Atom  26C    basis set group =>   1
Atom  27C    basis set group =>   1
```

```
Atom  28H    basis set group =>   2
Atom  29H    basis set group =>   2
Atom  30H    basis set group =>   2
Atom  31H    basis set group =>   2
Atom  32C    basis set group =>   1
Atom  33F    basis set group =>   5
Atom  34F    basis set group =>   5
Atom  35F    basis set group =>   5

           ************************************************************
           *        Program running with 12 parallel MPI-processes    *
           *              working on a common directory               *
           ************************************************************
------------------------------------------------------------------------------
                            ORCA STARTUP CALCULATIONS
                          -- RI-GTO INTEGRALS CHOSEN --
------------------------------------------------------------------------------
------------------------------------------------------------------------------
                   ___
                  /   \      - P O W E R E D   B Y -
                 /     \
                 |  |  |   _    _      __       _____    __    __
                 |  |  |  | |  | |    /  \     |  _  \  |  |  /  |
                  \  \/   | |  | |   /    \    | | | |  |  | /  /
                 / \  \   | |__| |  /  /\  \   | |_| |  |  |/  /
                |  |  |   |  __  | /  /__\  \  |    /   |      \
                |  |  |   | |  | | |   __   |  |    \   |  |\   \
                 \     /  | |  | | |  |  |  |  | |\  \  |  | \   \
                  \___/   |_|  |_| |__|  |__|  |_| \__\ |__|  \__/

                      - O R C A' S   B I G   F R I E N D -
                                       &
                       - I N T E G R A L   F E E D E R -

 v1 FN, 2020, v2 2021, v3 2022-2024
------------------------------------------------------------------------------


----------------------
SHARK INTEGRAL PACKAGE
----------------------

Number of atoms                             ...     36
Number of basis functions                   ...    758
Number of shells                            ...    296
Maximum angular momentum                    ...      3
Integral batch strategy                     ... SHARK/LIBINT Hybrid
RI-J (if used) integral strategy            ... SPLIT-RIJ (Revised 2003 algorithm where
possible)
Printlevel                                  ...      1
Contraction scheme used                     ... SEGMENTED contraction
Prescreening option                         ... SCHWARTZ
   Thresh                                   ... 1.000e-12
   Tcut                                     ... 1.000e-14
   Tpresel                                  ... 1.000e-14
Coulomb Range Separation                    ... NOT USED
Exchange Range Separation                   ... NOT USED
Multipole approximations                    ... NOT USED
Finite Nucleus Model                        ... NOT USED
CABS basis                                  ... NOT available
Auxiliary Coulomb fitting basis             ... AVAILABLE
   # of basis functions in Aux-J            ...   1203
```

```
   # of shells in Aux-J                     ...    393
   Maximum angular momentum in Aux-J        ...      4
Auxiliary J/K fitting basis                 ... NOT available
Auxiliary Correlation fitting basis         ... NOT available
Auxiliary 'external' fitting basis          ... NOT available

Checking pre-screening integrals            ... done (  0.0 sec) Dimension = 296
   => SHARK Basis and OBASIS are compatible. Storing Pre-screening
Shell pair information
Shell pair cut-off parameter TPreSel        ...   1.0e-14
Total number of shell pairs                 ...     43956
Shell pairs after pre-screening             ...     34727
Total number of primitive shell pairs       ...    140736
Primitive shell pairs kept                  ...     73854
          la=0 lb=0:   8699 shell pairs
          la=1 lb=0:   9808 shell pairs
          la=1 lb=1:   2812 shell pairs
          la=2 lb=0:   5107 shell pairs
          la=2 lb=1:   2944 shell pairs
          la=2 lb=2:    790 shell pairs
          la=3 lb=0:   2311 shell pairs
          la=3 lb=1:   1371 shell pairs
          la=3 lb=2:    710 shell pairs
          la=3 lb=3:    175 shell pairs

Checking whether 4 symmetric matrices of dimension 758 fit in memory:
Max Core in MB      =   8000.00
 MB in use           =     46.25
 MB left             =   7953.75
 MB needed           =      8.78
 Data fit in memory  = YES
Calculating RI/J V-Matrix + Cholesky decomp.... done (  0.1 sec)
Calculating Nuclear repulsion               ... done (  0.0 sec) ENN=   2226.977101228486 Eh

Diagonalization of the overlap matrix:
Smallest eigenvalue                        ... 5.837e-06
Time for diagonalization                   ...    0.104 sec
Threshold for overlap eigenvalues          ... 1.000e-07
Number of eigenvalues below threshold      ... 0
Time for construction of square roots      ...    0.066 sec
Total time needed                          ...    0.176 sec

-------------------
DFT GRID GENERATION
-------------------

General Integration Accuracy     IntAcc      ... 4.388
Radial Grid Type                 RadialGrid  ... OptM3 with GC (2021)
Angular Grid (max. ang.)         AngularGrid ... 4 (Lebedev-302)
Angular grid pruning method      GridPruning ... 4 (adaptive)
Weight generation scheme         WeightScheme... mBecke (2022)
Basis function cutoff            BFCut       ... 1.0000e-12
Integration weight cutoff        WCut        ... 1.0000e-14
Partially contracted basis set               ... off
Rotationally invariant grid construction     ... off
Angular grids for H and He will be reduced by one unit
Core-polarized basis detected: some atoms will have their core
                               angular grid increased.

Total number of grid points                  ...   178247
Total number of batches                      ...     2808
Average number of points per batch           ...       63
Average number of grid points per atom       ...     4951
```

```
-------------------
COSX GRID GENERATION
-------------------

GRIDX 1
-------
General Integration Accuracy     IntAcc      ... 3.816
Radial Grid Type                 RadialGrid  ... OptM3 with GC (2021)
Angular Grid (max. ang.)         AngularGrid ... 1 (Lebedev-50)
Angular grid pruning method      GridPruning ... 4 (adaptive)
Weight generation scheme         WeightScheme... mBecke (2022)
Basis function cutoff            BFCut       ... 1.0000e-12
Integration weight cutoff        WCut        ... 1.0000e-14
Partially contracted basis set               ... on
Rotationally invariant grid construction     ... off
Angular grids for H and He will be reduced by one unit
Core-polarized basis detected: some atoms will have their core
                               angular grid increased.

Total number of grid points                  ...    27065
Total number of batches                      ...      229
Average number of points per batch           ...      118
Average number of grid points per atom       ...      752
UseSFitting                                  ... on

GRIDX 2
-------
General Integration Accuracy     IntAcc      ... 4.020
Radial Grid Type                 RadialGrid  ... OptM3 with GC (2021)
Angular Grid (max. ang.)         AngularGrid ... 2 (Lebedev-110)
Angular grid pruning method      GridPruning ... 4 (adaptive)
Weight generation scheme         WeightScheme... mBecke (2022)
Basis function cutoff            BFCut       ... 1.0000e-12
Integration weight cutoff        WCut        ... 1.0000e-14
Partially contracted basis set               ... on
Rotationally invariant grid construction     ... off
Angular grids for H and He will be reduced by one unit
Core-polarized basis detected: some atoms will have their core
                               angular grid increased.

Total number of grid points                  ...    51964
Total number of batches                      ...      427
Average number of points per batch           ...      121
Average number of grid points per atom       ...     1443
UseSFitting                                  ... on

GRIDX 3
-------
General Integration Accuracy     IntAcc      ... 4.338
Radial Grid Type                 RadialGrid  ... OptM3 with GC (2021)
Angular Grid (max. ang.)         AngularGrid ... 3 (Lebedev-194)
Angular grid pruning method      GridPruning ... 4 (adaptive)
Weight generation scheme         WeightScheme... mBecke (2022)
Basis function cutoff            BFCut       ... 1.0000e-12
Integration weight cutoff        WCut        ... 1.0000e-14
Partially contracted basis set               ... on
Rotationally invariant grid construction     ... off
Angular grids for H and He will be reduced by one unit
Core-polarized basis detected: some atoms will have their core
                               angular grid increased.

Total number of grid points                  ...   106565
```

```
Total number of batches                      ...      850
Average number of points per batch           ...      125
Average number of grid points per atom       ...     2960
UseSFitting                                  ... on
Grids setup in   2.6 sec
Initializing property integral containers    ... done (  0.0 sec)

SHARK setup successfully completed in   3.4 seconds

Maximum memory used throughout the entire STARTUP-calculation: 89.2 MB


           ************************************************************
           *        Program running with 12 parallel MPI-processes    *
           *              working on a common directory               *
           ************************************************************
--------------------------------------------------------------------------------
                                 ORCA GUESS
                   Start orbitals & Density for SCF / CASSCF
--------------------------------------------------------------------------------


------------
SCF SETTINGS
------------
Hamiltonian:
 Density Functional     Method          .... DFT(GTOs)
 Exchange Functional    Exchange        .... B88
   X-Alpha parameter    XAlpha          ....  0.666667
   Becke's b parameter  XBeta           ....  0.004200
 Correlation Functional Correlation     .... LYP
 LDA part of GGA corr.  LDAOpt          .... VWN-5
 Gradients option       PostSCFGGA      .... off
 Hybrid DFT is turned on
   Fraction HF Exchange ScalHFX         ....  0.200000
   Scaling of DF-GGA-X  ScalDFX         ....  0.720000
   Scaling of DF-GGA-C  ScalDFC         ....  0.810000
   Scaling of DF-LDA-C  ScalLDAC        ....  1.000000
   Perturbative correction              ....  0.000000
   NL short-range parameter             ....  4.800000
 RI-approximation to the Coulomb term is turned on
   Number of AuxJ basis functions       .... 1203
   RIJ-COSX (HFX calculated with COS-X)).... on


General Settings:
 Integral files         IntName         .... CF3-IRC
 Hartree-Fock type      HFTyp           .... RHF
 Total Charge           Charge          ....    0
 Multiplicity           Mult            ....    1
 Number of Electrons    NEL             ....  180
 Basis Dimension        Dim             ....  758
 Nuclear Repulsion      ENuc            ....   2226.9771012285 Eh

Convergence Acceleration:
 AO-DIIS                CNVDIIS         .... on
   Start iteration      DIISMaxIt       ....    12
   Startup error        DIISStart       ....  0.200000
   # of expansion vecs  DIISMaxEq       ....     5
   Bias factor          DIISBfac        ....   1.050
   Max. coefficient     DIISMaxC        ....  10.000
 MO-DIIS                CNVKDIIS        .... off
 Trust-Rad. Augm. Hess. CNVTRAH         .... auto
   Auto Start mean grad. ratio tolernc. ....  1.125000
```

```
   Auto Start start iteration           ....    50
   Auto Start num. interpolation iter.  ....    10
   Max. Number of Micro iterations      ....    24
   Max. Number of Macro iterations      .... Maxiter - #DIIS iter
   Number of Davidson start vectors     ....     2
   Converg. threshold    (grad. norm)   ....   2.000e-06
   Grad. Scal. Fac. for Micro threshold ....   0.100
   Minimum threshold for Micro iter.    ....   1.000e-02
   NR start threshold (gradient norm)   ....   1.000e-04
   Initial trust radius                 ....   0.400
   Minimum AH scaling param. (alpha)    ....   1.000
   Maximum AH scaling param. (alpha)    .... 1000.000
   Quad. conv. algorithm                .... NR
   White noise on init. David. guess    .... on
   Maximum white noise                  ....   0.010
   Pseudo random numbers                .... off
   Inactive MOs                         .... canonical
   Orbital update algorithm             .... Taylor
   Preconditioner                       .... Diag
   Full preconditioner red. dimension   ....   250
 SOSCF                 CNVSOSCF           .... on
   Start iteration     SOSCFMaxIt         ....   150
   Startup grad/error  SOSCFStart         ....  0.003300
   Hessian update      SOSCFHessUp        .... L-BFGS
   Autom. constraints  SOSCFAutoConstrain .... off
 Level Shifting         CNVShift        .... on
   Level shift para.    LevelShift      ....    0.2500
   Turn off err/grad.   ShiftErr        ....    0.0010
 Zerner damping         CNVZerner       .... off
 Static damping         CNVDamp         .... on
   Fraction old density DampFac         ....    0.7000
   Max. Damping (<1)    DampMax         ....    0.9800
   Min. Damping (>=0)   DampMin         ....    0.0000
   Turn off err/grad.   DampErr         ....    0.1000

SCF Procedure:
 Maximum # iterations   MaxIter         ....   125
 SCF integral mode      SCFMode         .... Direct
   Integral package                     .... SHARK and LIBINT hybrid scheme
 Reset frequency        DirectResetFreq ....    20
 Integral Threshold     Thresh          ....  1.000e-12 Eh
 Primitive CutOff       TCut            ....  1.000e-14 Eh

Convergence Tolerance:
 Convergence Check Mode ConvCheckMode   .... Total+1el-Energy
 Convergence forced     ConvForced      .... 0
 Energy Change          TolE            ....  1.000e-09 Eh
 1-El. energy change                    ....  1.000e-06 Eh
 Orbital Gradient       TolG            ....  2.000e-06
 Orbital Rotation angle TolX            ....  2.000e-06
 DIIS Error             TolErr          ....  1.000e-08

------------------------------
INITIAL GUESS: MODEL POTENTIAL
------------------------------
Loading Hartree-Fock densities                    ... done
Calculating cut-offs                              ... done
Initializing the effective Hamiltonian            ... done
Setting up the integral package (SHARK)           ... done
Starting the Coulomb interaction                  ... done (   0.5 sec)
Making the grid                                   ... done (   0.3 sec)
Mapping shells                                    ... done
Starting the XC term evaluation                   ... done (   0.2 sec)
```

```
  promolecular density results
     # of electrons  =    180.000116687
     EX              =   -210.712657219
     EC              =     -6.698278431
     EX+EC           =   -217.410935650
Transforming the Hamiltonian                       ... done (   0.1 sec)
Diagonalizing the Hamiltonian                      ... done (   0.1 sec)
Back transforming the eigenvectors                 ... done (   0.0 sec)
Now organizing SCF variables                       ... done
                      ------------------
                      INITIAL GUESS DONE (   1.3 sec)
                      ------------------
             **** ENERGY FILE WAS UPDATED (CF3-IRC.en.tmp) ****
Finished Guess after   1.6 sec
Maximum memory used throughout the entire GUESS-calculation: 53.3 MB


           ************************************************************
           *        Program running with 12 parallel MPI-processes    *
           *              working on a common directory               *
           ************************************************************

------------------------------------------------------------------------------------------
                                      ORCA LEAN-SCF
                              memory conserving SCF solver
------------------------------------------------------------------------------------------

--------------------
CPCM SOLVATION MODEL
--------------------
CPCM parameters:
  Epsilon                                         ...       7.4257
  Refrac                                          ...       1.4050
  Rsolv                                           ...       1.3000
  Surface type                                    ... GAUSSIAN VDW
  Discretization scheme                           ... Constant charge density
    Threshold for H atoms                         ...       5.0000 (charges/Ang^2)
    Threshold for non-H atoms                     ...       5.0000 (charges/Ang^2)
  Epsilon function type                           ...         CPCM
Solvent:                                          ... TETRAHYDROFURAN
SMD-CDS solvent descriptors:
  Soln                                            ...       1.4050
  Soln25                                          ...       1.4044
  Sola                                            ...       0.0000
  Solb                                            ...       0.4800
  Solg                                            ...      39.4400
  Solc                                            ...       0.0000
  Solh                                            ...       0.0000
Radii:
  Scheme                                          ... Element-dependent radii
  Radius for C  used is    3.4960 Bohr (=   1.8500 Ang.)
  Radius for H  used is    2.2677 Bohr (=   1.2000 Ang.)
  Radius for Se used is    3.5905 Bohr (=   1.9000 Ang.)
  Radius for O  used is    4.3350 Bohr (=   2.2940 Ang.)
  Radius for F  used is    3.2692 Bohr (=   1.7300 Ang.)
Calculating surface                               ...        done! (  0.0s)
Cavity surface points                             ...         2885
Cavity Volume                                     ...    2161.6353
Cavity Surface-area                               ...    1188.1225
Calculating surface distance matrix               ...        done! (  0.1s)
Performing Cholesky decomposition & store         ...        done! (  0.2s)
Overall time for CPCM initialization              ...                 0.4s
```

```
                                      --------------------------------------D-I-I-S--------------------------------------------
Iteration    Energy (Eh)           Delta-E    RMSDP     MaxDP     DIISErr   Damp  Time(sec)
-------------------------------------------------------------------------------------------
               ***  Starting incremental Fock matrix formation  ***
    1    -3394.0601964796260290     0.00e+00  5.27e-03  2.05e-01  3.12e-01  0.700  11.4
    2    -3394.3829257291145041    -3.23e-01  3.80e-03  1.58e-01  1.11e-01  0.700   7.1
                               ***Turning on AO-DIIS***
    3    -3394.5000715337237125    -1.17e-01  1.27e-03  3.12e-02  4.39e-02  0.700   6.7
    4    -3394.5722846732014659    -7.22e-02  2.44e-03  9.06e-02  2.04e-02  0.000   6.5
    5    -3394.7337810627432191    -1.61e-01  7.88e-04  2.56e-02  7.93e-03  0.000   6.8
                              *** Initializing SOSCF ***
-------------------------------------S-O-S-C-F--------------------------------------
Iteration    Energy (Eh)           Delta-E    RMSDP     MaxDP     MaxGrad    Time(sec)
--------------------------------------------------------------------------------------
    6    -3394.7350261060782941    -1.25e-03  3.26e-04  9.48e-03  2.52e-03     6.6
               *** Restarting incremental Fock matrix formation ***
    7    -3394.7351197150446751    -9.36e-05  3.61e-04  1.46e-02  5.67e-04    11.3
    8    -3394.7351082065433729     1.15e-05  1.20e-04  4.08e-03  7.02e-04     9.0
    9    -3394.7351372395642102    -2.90e-05  6.16e-05  7.94e-04  1.72e-04     8.4
   10    -3394.7351348931470056     2.35e-06  2.31e-05  3.64e-04  2.02e-04     8.4
   11    -3394.7351399067274542    -5.01e-06  1.02e-05  2.52e-04  3.59e-05     8.0
   12    -3394.7351398420632904     6.47e-08  5.08e-06  1.16e-04  4.56e-05     7.4
   13    -3394.7351399892404515    -1.47e-07  1.19e-06  2.37e-05  7.62e-06     7.0
   14    -3394.7351399869121451     2.33e-09  6.10e-07  9.52e-06  7.37e-06     6.2
   15    -3394.7351399909566680    -4.04e-09  3.59e-07  9.75e-06  1.66e-06     5.8
   16    -3394.7351399906119696     3.45e-10  1.57e-07  3.00e-06  1.78e-06     5.0
                 **** Energy Check signals convergence ****

               *****************************************************
               *                     SUCCESS                       *
               *           SCF CONVERGED AFTER  16 CYCLES          *
               *****************************************************

Recomputing exchange energy using gridx3       ... done (   16.142 sec)
Old exchange energy                            :    -42.427485068 Eh
New exchange energy                            :    -42.427486555 Eh
Exchange energy change after final integration :     -0.000001487 Eh
Total energy after final integration           :  -3394.735141478 Eh

SMD CDS free energy correction energy :                 -2.85471     Kcal/mol
Total Energy after SMD CDS correction =             -3394.739690753 Eh
             **** ENERGY FILE WAS UPDATED (CF3-IRC.en.tmp) ****

----------------
TOTAL SCF ENERGY
----------------

Total Energy       :      -3394.73969075286095 Eh          -92375.56327 eV

Components:
Nuclear Repulsion  :       2226.97710122848639 Eh           60599.12772 eV
Electronic Energy  :      -5621.69521054439792 Eh         -152974.10373 eV
One Electron Energy:      -9134.31002839571011 Eh         -248557.21228 eV
Two Electron Energy:       3512.61481785131218 Eh           95583.10855 eV
CPCM Dielectric    :         -0.01703067534898 Eh              -0.46343 eV
SMD CDS (Gcds)     :         -0.00454927460310 Eh              -0.12379 eV

Virial components:
Potential Energy   :      -6781.78438575354812 Eh         -184541.73506 eV
Kinetic Energy     :       3387.04469500068672 Eh           92166.17179 eV
Virial Ratio       :          2.00227189082079

DFT components:
```

```
N(Alpha)           :       89.999951169373 electrons
N(Beta)            :       89.999951169373 electrons
N(Total)           :      179.999902338746 electrons
E(X)               :     -168.823340643090 Eh
E(C)               :       -7.864089238022 Eh
E(XC)              :     -176.687429881111 Eh

CPCM Solvation Model Properties:
Surface-charge          :         -0.05101721431769
Corrected charge        :          0.00000000000000
Outlying charge corr.   :          0.00012964367681 Eh               0.00353 eV
Free-energy (cav+disp)  :         -0.00454927460310 Eh              -0.12379 eV

---------------
SCF CONVERGENCE
---------------

  Last Energy change         ...   -3.4470e-10  Tolerance :   1.0000e-09
  Last MAX-Density change    ...    2.9965e-06  Tolerance :   1.0000e-08
  Last RMS-Density change    ...    1.5740e-07  Tolerance :   1.0000e-09
  Last DIIS Error            ...    2.5237e-03  Tolerance :   1.0000e-08
  Last Orbital Gradient      ...    1.7848e-06  Tolerance :   2.0000e-06
  Last Orbital Rotation      ...    1.9193e-06  Tolerance :   2.0000e-06


----------------
ORBITAL ENERGIES
----------------

  NO   OCC          E(Eh)            E(eV) 
   0   2.0000    -453.932501    -12352.1313 
   1   2.0000     -58.112839     -1581.3307 
   2   2.0000     -52.165282     -1419.4895 
   3   2.0000     -52.164533     -1419.4691 
   4   2.0000     -52.163443     -1419.4395 
   5   2.0000     -24.716320      -672.5653 
   6   2.0000     -24.716011      -672.5569 
   7   2.0000     -24.712279      -672.4553 
   8   2.0000     -19.113058      -520.0927 
   9   2.0000     -10.426115      -283.7090 
  10   2.0000     -10.207267      -277.7538 
  11   2.0000     -10.202247      -277.6172 
  12   2.0000     -10.190856      -277.3073 
  13   2.0000     -10.189807      -277.2787 
  14   2.0000     -10.188260      -277.2366 
  15   2.0000     -10.187819      -277.2246 
  16   2.0000     -10.185065      -277.1497 
  17   2.0000     -10.174207      -276.8542 
  18   2.0000     -10.167026      -276.6588 
  19   2.0000     -10.166321      -276.6397 
  20   2.0000     -10.165859      -276.6271 
  21   2.0000     -10.164899      -276.6010 
  22   2.0000     -10.164759      -276.5972 
  23   2.0000     -10.163997      -276.5764 
  24   2.0000     -10.156617      -276.3756 
  25   2.0000      -7.851160      -213.6409 
  26   2.0000      -5.795097      -157.6926 
  27   2.0000      -5.791288      -157.5890 
  28   2.0000      -5.787459      -157.4848 
  29   2.0000      -2.148627       -58.4671 
  30   2.0000      -2.147892       -58.4471 
  31   2.0000      -2.144099       -58.3439 
  32   2.0000      -2.140815       -58.2545 
```

33	2.0000	-2.140444	-58.2445
34	2.0000	-1.315059	-35.7846
35	2.0000	-1.229435	-33.4546
36	2.0000	-1.224592	-33.3228
37	2.0000	-0.965749	-26.2794
38	2.0000	-0.880633	-23.9632
39	2.0000	-0.855587	-23.2817
40	2.0000	-0.796937	-21.6858
41	2.0000	-0.795114	-21.6361
42	2.0000	-0.764005	-20.7896
43	2.0000	-0.744885	-20.2693
44	2.0000	-0.740381	-20.1468
45	2.0000	-0.717463	-19.5231
46	2.0000	-0.679616	-18.4933
47	2.0000	-0.663800	-18.0629
48	2.0000	-0.630521	-17.1573
49	2.0000	-0.611327	-16.6350
50	2.0000	-0.600709	-16.3461
51	2.0000	-0.595897	-16.2152
52	2.0000	-0.590620	-16.0716
53	2.0000	-0.590374	-16.0649
54	2.0000	-0.570031	-15.5113
55	2.0000	-0.555394	-15.1130
56	2.0000	-0.503988	-13.7142
57	2.0000	-0.484259	-13.1773
58	2.0000	-0.482460	-13.1284
59	2.0000	-0.476234	-12.9590
60	2.0000	-0.470319	-12.7980
61	2.0000	-0.466059	-12.6821
62	2.0000	-0.464995	-12.6531
63	2.0000	-0.457593	-12.4517
64	2.0000	-0.447356	-12.1732
65	2.0000	-0.442628	-12.0445
66	2.0000	-0.440442	-11.9850
67	2.0000	-0.438592	-11.9347
68	2.0000	-0.425421	-11.5763
69	2.0000	-0.417654	-11.3649
70	2.0000	-0.416793	-11.3415
71	2.0000	-0.413723	-11.2580
72	2.0000	-0.404978	-11.0200
73	2.0000	-0.393771	-10.7151
74	2.0000	-0.389755	-10.6058
75	2.0000	-0.376881	-10.2555
76	2.0000	-0.367712	-10.0060
77	2.0000	-0.365511	-9.9461
78	2.0000	-0.359981	-9.7956
79	2.0000	-0.353330	-9.6146
80	2.0000	-0.349320	-9.5055
81	2.0000	-0.333098	-9.0641
82	2.0000	-0.326221	-8.8769
83	2.0000	-0.289414	-7.8754
84	2.0000	-0.274679	-7.4744
85	2.0000	-0.271282	-7.3820
86	2.0000	-0.253107	-6.8874
87	2.0000	-0.246700	-6.7130
88	2.0000	-0.242762	-6.6059
89	2.0000	-0.231753	-6.3063
90	0.0000	-0.050839	-1.3834
91	0.0000	-0.033332	-0.9070
92	0.0000	-0.025540	-0.6950
93	0.0000	-0.006805	-0.1852
94	0.0000	-0.005108	-0.1390
95	0.0000	0.007272	0.1979

```
  96   0.0000       0.032708         0.8900
  97   0.0000       0.051631         1.4049
  98   0.0000       0.064848         1.7646
  99   0.0000       0.070152         1.9089
 100   0.0000       0.073995         2.0135
*Only the first 10 virtual orbitals were printed (use %output print[p_orben] 2 end to see all).

                    ********************************
                    * MULLIKEN POPULATION ANALYSIS *
                    ********************************

-----------------------
MULLIKEN ATOMIC CHARGES
-----------------------
   0 C :    0.175102
   1 C :   -0.229306
   2 C :   -0.241998
   3 C :   -0.106359
   4 C :   -0.125477
   5 C :   -0.171918
   6 H :    0.125795
   7 H :    0.136251
   8 H :    0.138533
   9 H :    0.137248
  10 H :    0.136175
  11 C :   -0.179405
  12 H :    0.114226
  13 H :    0.117437
  14 C :   -0.267355
  15 H :    0.257340
  16 H :    0.129121
  17 C :   -0.298113
  18 H :    0.164702
  19 H :    0.167739
  20 Se:    0.431210
  21 O :   -0.571565
  22 C :    0.062826
  23 C :   -0.164952
  24 C :   -0.184323
  25 C :   -0.220529
  26 C :   -0.205434
  27 C :    0.113815
  28 H :    0.144818
  29 H :    0.155949
  30 H :    0.153034
  31 H :    0.154817
  32 C :    0.527962
  33 F :   -0.193068
  34 F :   -0.191888
  35 F :   -0.192409
Sum of atomic charges:    0.0000000

--------------------------------
MULLIKEN REDUCED ORBITAL CHARGES
--------------------------------
  0 C s       :     3.165345  s :     3.165345
      pz      :     0.882483  p :     2.525383
      px      :     0.778439
      py      :     0.864461
      dz2     :     0.024766  d :     0.124345
      dxz     :     0.028639
      dyz     :     0.019516
      dx2y2   :     0.024352
```

```
    dxy     :     0.027072
    f0      :     0.001163  f :     0.009824
    f+1     :     0.001347
    f-1     :     0.001274
    f+2     :     0.001962
    f-2     :     0.001344
    f+3     :     0.001121
    f-3     :     0.001614

1 C s       :     3.258071  s :     3.258071
    pz      :     0.974570  p :     2.880925
    px      :     0.971928
    py      :     0.934427
    dz2     :     0.017108  d :     0.082998
    dxz     :     0.021091
    dyz     :     0.011200
    dx2y2   :     0.013374
    dxy     :     0.020225
    f0      :     0.001113  f :     0.007311
    f+1     :     0.001072
    f-1     :     0.000634
    f+2     :     0.001319
    f-2     :     0.001281
    f+3     :     0.001028
    f-3     :     0.000864

2 C s       :     3.273289  s :     3.273289
    pz      :     0.965882  p :     2.879386
    px      :     0.950952
    py      :     0.962552
    dz2     :     0.012674  d :     0.082046
    dxz     :     0.019139
    dyz     :     0.011162
    dx2y2   :     0.018836
    dxy     :     0.020235
    f0      :     0.000333  f :     0.007277
    f+1     :     0.001243
    f-1     :     0.001038
    f+2     :     0.001102
    f-2     :     0.001182
    f+3     :     0.001040
    f-3     :     0.001339

3 C s       :     3.193218  s :     3.193218
    pz      :     0.947720  p :     2.822567
    px      :     0.926801
    py      :     0.948047
    dz2     :     0.012667  d :     0.083294
    dxz     :     0.019173
    dyz     :     0.010657
    dx2y2   :     0.020168
    dxy     :     0.020629
    f0      :     0.000335  f :     0.007280
    f+1     :     0.001248
    f-1     :     0.001091
    f+2     :     0.001131
    f-2     :     0.001177
    f+3     :     0.000993
    f-3     :     0.001306

4 C s       :     3.211446  s :     3.211446
    pz      :     0.945883  p :     2.824407
    px      :     0.924317
```

```
      py       :      0.954207
      dz2      :      0.016945  d :      0.082367
      dxz      :      0.021115
      dyz      :      0.010890
      dx2y2    :      0.013355
      dxy      :      0.020062
      f0       :      0.001146  f :      0.007257
      f+1      :      0.001017
      f-1      :      0.000636
      f+2      :      0.001283
      f-2      :      0.001282
      f+3      :      0.001010
      f-3      :      0.000884

  5 C s        :      3.237192  s :      3.237192
      pz       :      0.941482  p :      2.846391
      px       :      0.966438
      py       :      0.938471
      dz2      :      0.020484  d :      0.081111
      dxz      :      0.014519
      dyz      :      0.015456
      dx2y2    :      0.019376
      dxy      :      0.011275
      f0       :      0.000984  f :      0.007224
      f+1      :      0.000833
      f-1      :      0.001141
      f+2      :      0.001462
      f-2      :      0.001090
      f+3      :      0.000789
      f-3      :      0.000926

  6 H s        :      0.851921  s :      0.851921
      pz       :      0.006349  p :      0.022284
      px       :      0.006607
      py       :      0.009327

  7 H s        :      0.841629  s :      0.841629
      pz       :      0.010136  p :      0.022120
      px       :      0.005287
      py       :      0.006697

  8 H s        :      0.839100  s :      0.839100
      pz       :      0.010408  p :      0.022367
      px       :      0.005323
      py       :      0.006636

  9 H s        :      0.840589  s :      0.840589
      pz       :      0.006291  p :      0.022163
      px       :      0.006603
      py       :      0.009270

 10 H s        :      0.841561  s :      0.841561
      pz       :      0.005061  p :      0.022264
      px       :      0.012567
      py       :      0.004636

 11 C s        :      3.232700  s :      3.232700
      pz       :      0.970243  p :      2.854609
      px       :      0.910957
      py       :      0.973409
      dz2      :      0.018775  d :      0.086355
      dxz      :      0.023348
      dyz      :      0.011359
```

```
      dx2y2   :     0.014728
      dxy     :     0.018145
      f0      :     0.000613  f :     0.005741
      f+1     :     0.000790
      f-1     :     0.000870
      f+2     :     0.001110
      f-2     :     0.000983
      f+3     :     0.000808
      f-3     :     0.000567

 12 H s       :     0.864077  s :     0.864077
      pz      :     0.004979  p :     0.021697
      px      :     0.006286
      py      :     0.010433

 13 H s       :     0.860758  s :     0.860758
      pz      :     0.009455  p :     0.021805
      px      :     0.007110
      py      :     0.005240

 14 C s       :     3.203724  s :     3.203724
      pz      :     0.978718  p :     2.962799
      px      :     0.946102
      py      :     1.037979
      dz2     :     0.027452  d :     0.094486
      dxz     :     0.014837
      dyz     :     0.017356
      dx2y2   :     0.021957
      dxy     :     0.012883
      f0      :     0.001145  f :     0.006347
      f+1     :     0.000753
      f-1     :     0.000691
      f+2     :     0.001625
      f-2     :     0.001064
      f+3     :     0.000390
      f-3     :     0.000678

 15 H s       :     0.690780  s :     0.690780
      pz      :     0.008502  p :     0.051880
      px      :     0.007305
      py      :     0.036072

 16 H s       :     0.848201  s :     0.848201
      pz      :     0.005668  p :     0.022678
      px      :     0.011732
      py      :     0.005278

 17 C s       :     3.302777  s :     3.302777
      pz      :     1.012077  p :     2.919470
      px      :     1.000378
      py      :     0.907016
      dz2     :     0.023639  d :     0.070926
      dxz     :     0.006359
      dyz     :     0.010683
      dx2y2   :     0.013371
      dxy     :     0.016873
      f0      :     0.000727  f :     0.004939
      f+1     :     0.001167
      f-1     :     0.000591
      f+2     :     0.000367
      f-2     :     0.000942
      f+3     :     0.000376
      f-3     :     0.000769
```

```
18 H s       :     0.812975  s :     0.812975
     pz      :     0.012715  p :     0.022323
     px      :     0.004318
     py      :     0.005290

19 H s       :     0.809400  s :     0.809400
     pz      :     0.004184  p :     0.022861
     px      :     0.011879
     py      :     0.006798

20 Ses       :     7.841983  s :     7.841983
     pz      :     5.112833  p :    15.455792
     px      :     4.782962
     py      :     5.559996
     dz2     :     2.048743  d :    10.230410
     dxz     :     2.051322
     dyz     :     2.035000
     dx2y2   :     2.057629
     dxy     :     2.037716
     f0      :     0.005661  f :     0.040604
     f+1     :     0.010422
     f-1     :     0.001688
     f+2     :     0.007176
     f-2     :     0.007849
     f+3     :     0.003758
     f-3     :     0.004050

21 O s       :     3.868139  s :     3.868139
     pz      :     1.586524  p :     4.676504
     px      :     1.477648
     py      :     1.612331
     dz2     :     0.005331  d :     0.025952
     dxz     :     0.006911
     dyz     :     0.002935
     dx2y2   :     0.006059
     dxy     :     0.004717
     f0      :     0.000100  f :     0.000971
     f+1     :     0.000232
     f-1     :     0.000060
     f+2     :     0.000213
     f-2     :     0.000153
     f+3     :     0.000117
     f-3     :     0.000095

22 C s       :     3.169877  s :     3.169877
     pz      :     0.827130  p :     2.632777
     px      :     0.896873
     py      :     0.908774
     dz2     :     0.022413  d :     0.126223
     dxz     :     0.042005
     dyz     :     0.025260
     dx2y2   :     0.019638
     dxy     :     0.016907
     f0      :     0.001263  f :     0.008297
     f+1     :     0.001835
     f-1     :     0.001029
     f+2     :     0.001044
     f-2     :     0.001129
     f+3     :     0.001302
     f-3     :     0.000695

23 C s       :     3.265491  s :     3.265491
```

```
       pz      :     0.919050  p :     2.803524
       px      :     0.910810
       py      :     0.973664
       dz2     :     0.025226  d :     0.088416
       dxz     :     0.024030
       dyz     :     0.015986
       dx2y2   :     0.008423
       dxy     :     0.014750
       f0      :     0.001290  f :     0.007522
       f+1     :     0.001852
       f-1     :     0.000842
       f+2     :     0.000779
       f-2     :     0.001236
       f+3     :     0.000627
       f-3     :     0.000896

  24 C s       :     3.260902  s :     3.260902
       pz      :     0.954579  p :     2.830896
       px      :     0.917041
       py      :     0.959276
       dz2     :     0.023859  d :     0.085076
       dxz     :     0.015423
       dyz     :     0.009333
       dx2y2   :     0.017996
       dxy     :     0.018466
       f0      :     0.000753  f :     0.007449
       f+1     :     0.001581
       f-1     :     0.000757
       f+2     :     0.000904
       f-2     :     0.001209
       f+3     :     0.001356
       f-3     :     0.000889

  25 C s       :     3.314726  s :     3.314726
       pz      :     0.958464  p :     2.815903
       px      :     0.914639
       py      :     0.942800
       dz2     :     0.021384  d :     0.082404
       dxz     :     0.016059
       dyz     :     0.008967
       dx2y2   :     0.016811
       dxy     :     0.019183
       f0      :     0.000771  f :     0.007496
       f+1     :     0.001578
       f-1     :     0.000730
       f+2     :     0.000916
       f-2     :     0.001184
       f+3     :     0.001416
       f-3     :     0.000900

  26 C s       :     3.284029  s :     3.284029
       pz      :     0.923346  p :     2.831490
       px      :     0.972435
       py      :     0.935710
       dz2     :     0.024536  d :     0.082402
       dxz     :     0.018225
       dyz     :     0.016667
       dx2y2   :     0.008509
       dxy     :     0.014465
       f0      :     0.001341  f :     0.007513
       f+1     :     0.001825
       f-1     :     0.000795
       f+2     :     0.000777
```

```
     f-2     :     0.001266
     f+3     :     0.000641
     f-3     :     0.000867

27 C s       :     3.174442  s :     3.174442
     pz      :     0.822257  p :     2.598532
     px      :     0.825478
     py      :     0.950798
     dz2     :     0.017456  d :     0.103399
     dxz     :     0.034541
     dyz     :     0.022841
     dx2y2   :     0.019224
     dxy     :     0.009336
     f0      :     0.001430  f :     0.009812
     f+1     :     0.002282
     f-1     :     0.001040
     f+2     :     0.001504
     f-2     :     0.001269
     f+3     :     0.001456
     f-3     :     0.000830

28 H s       :     0.832496  s :     0.832496
     pz      :     0.005125  p :     0.022687
     px      :     0.010757
     py      :     0.006804

29 H s       :     0.821661  s :     0.821661
     pz      :     0.013147  p :     0.022390
     px      :     0.003686
     py      :     0.005557

30 H s       :     0.824433  s :     0.824433
     pz      :     0.013340  p :     0.022534
     px      :     0.003728
     py      :     0.005465

31 H s       :     0.822656  s :     0.822656
     pz      :     0.005267  p :     0.022527
     px      :     0.010345
     py      :     0.006915

32 C s       :     2.997276  s :     2.997276
     pz      :     0.701625  p :     2.112361
     px      :     0.777506
     py      :     0.633230
     dz2     :     0.072901  d :     0.323481
     dxz     :     0.053355
     dyz     :     0.065413
     dx2y2   :     0.058544
     dxy     :     0.073269
     f0      :     0.003669  f :     0.038919
     f+1     :     0.005109
     f-1     :     0.006970
     f+2     :     0.005584
     f-2     :     0.007482
     f+3     :     0.005179
     f-3     :     0.004926

33 F s       :     3.913512  s :     3.913512
     pz      :     1.801631  p :     5.263854
     px      :     1.905541
     py      :     1.556683
     dz2     :     0.001740  d :     0.015015
```

```
     dxz     :     0.000967
     dyz     :     0.005382
     dx2y2   :     0.004429
     dxy     :     0.002496
     f0      :     0.000077  f :     0.000687
     f+1     :     0.000004
     f-1     :     0.000114
     f+2     :     0.000197
     f-2     :     0.000077
     f+3     :     0.000086
     f-3     :     0.000133

 34 F s       :     3.913878  s :     3.913878
     pz      :     1.671318  p :     5.261698
     px      :     1.894958
     py      :     1.695422
     dz2     :     0.002908  d :     0.015600
     dxz     :     0.002067
     dyz     :     0.006428
     dx2y2   :     0.002485
     dxy     :     0.001712
     f0      :     0.000073  f :     0.000712
     f+1     :     0.000039
     f-1     :     0.000218
     f+2     :     0.000185
     f-2     :     0.000110
     f+3     :     0.000038
     f-3     :     0.000050

 35 F s       :     3.913767  s :     3.913767
     pz      :     1.782088  p :     5.262381
     px      :     1.583697
     py      :     1.896596
     dz2     :     0.002073  d :     0.015550
     dxz     :     0.006089
     dyz     :     0.001059
     dx2y2   :     0.004114
     dxy     :     0.002215
     f0      :     0.000076  f :     0.000710
     f+1     :     0.000148
     f-1     :     0.000008
     f+2     :     0.000216
     f-2     :     0.000083
     f+3     :     0.000110
     f-3     :     0.000069


                       *******************************
                       * LOEWDIN POPULATION ANALYSIS *
                       *******************************

----------------------
LOEWDIN ATOMIC CHARGES
----------------------
   0 C :   -0.148685
   1 C :   -0.114820
   2 C :   -0.123220
   3 C :   -0.142648
   4 C :   -0.152464
   5 C :   -0.155563
   6 H :    0.150983
   7 H :    0.151841
```

```
   8 H :    0.148027
   9 H :    0.147931
  10 H :    0.146158
  11 C :   -0.209375
  12 H :    0.148681
  13 H :    0.146169
  14 C :   -0.310516
  15 H :    0.189117
  16 H :    0.145433
  17 C :   -0.224776
  18 H :    0.126576
  19 H :    0.129457
  20 Se:    0.657883
  21 O :   -0.407893
  22 C :   -0.308334
  23 C :   -0.124661
  24 C :   -0.127818
  25 C :   -0.085972
  26 C :   -0.085377
  27 C :   -0.134484
  28 H :    0.154631
  29 H :    0.157470
  30 H :    0.161011
  31 H :    0.161292
  32 C :   -0.603843
  33 F :    0.169973
  34 F :    0.184776
  35 F :    0.183041

-------------------------------
LOEWDIN REDUCED ORBITAL CHARGES
-------------------------------
  0 C s       :     2.710848  s :     2.710848
      pz      :     0.958289  p :     2.860716
      px      :     0.963247
      py      :     0.939180
      dz2     :     0.099712  d :     0.519341
      dxz     :     0.115036
      dyz     :     0.086208
      dx2y2   :     0.113167
      dxy     :     0.105218
      f0      :     0.006340  f :     0.057780
      f+1     :     0.009272
      f-1     :     0.007442
      f+2     :     0.010879
      f-2     :     0.007025
      f+3     :     0.006702
      f-3     :     0.010121

  1 C s       :     2.759524  s :     2.759524
      pz      :     0.971728  p :     2.928562
      px      :     1.016632
      py      :     0.940201
      dz2     :     0.067437  d :     0.383944
      dxz     :     0.104798
      dyz     :     0.056616
      dx2y2   :     0.066095
      dxy     :     0.088998
      f0      :     0.006134  f :     0.042791
      f+1     :     0.006568
      f-1     :     0.004744
      f+2     :     0.008077
      f-2     :     0.005053
```

```
    f+3     :     0.005865
    f-3     :     0.006350

2 C s       :     2.760251  s :     2.760251
    pz      :     0.950606  p :     2.935941
    px      :     1.026103
    py      :     0.959232
    dz2     :     0.055509  d :     0.384388
    dxz     :     0.088617
    dyz     :     0.056789
    dx2y2   :     0.086617
    dxy     :     0.096856
    f0      :     0.002835  f :     0.042639
    f+1     :     0.007551
    f-1     :     0.004965
    f+2     :     0.007433
    f-2     :     0.005188
    f+3     :     0.005488
    f-3     :     0.009179

3 C s       :     2.765359  s :     2.765359
    pz      :     0.952036  p :     2.945332
    px      :     1.033977
    py      :     0.959319
    dz2     :     0.057373  d :     0.388483
    dxz     :     0.089049
    dyz     :     0.056499
    dx2y2   :     0.087955
    dxy     :     0.097608
    f0      :     0.002832  f :     0.043475
    f+1     :     0.007691
    f-1     :     0.005246
    f+2     :     0.007627
    f-2     :     0.005316
    f+3     :     0.005492
    f-3     :     0.009270

4 C s       :     2.766303  s :     2.766303
    pz      :     0.984399  p :     2.953811
    px      :     1.024105
    py      :     0.945307
    dz2     :     0.070248  d :     0.389063
    dxz     :     0.106223
    dyz     :     0.057155
    dx2y2   :     0.065112
    dxy     :     0.090325
    f0      :     0.006400  f :     0.043286
    f+1     :     0.006492
    f-1     :     0.004879
    f+2     :     0.008176
    f-2     :     0.005048
    f+3     :     0.005898
    f-3     :     0.006394

5 C s       :     2.766518  s :     2.766518
    pz      :     0.995751  p :     2.959114
    px      :     0.981976
    py      :     0.981387
    dz2     :     0.085532  d :     0.386505
    dxz     :     0.074319
    dyz     :     0.077841
    dx2y2   :     0.089922
    dxy     :     0.058891
```

```
     f0      :     0.005310  f :     0.043426
     f+1     :     0.006326
     f-1     :     0.006690
     f+2     :     0.008019
     f-2     :     0.006035
     f+3     :     0.004699
     f-3     :     0.006348

  6 H s      :     0.789714  s :     0.789714
     pz      :     0.017071  p :     0.059304
     px      :     0.017755
     py      :     0.024477

  7 H s      :     0.788990  s :     0.788990
     pz      :     0.026409  p :     0.059169
     px      :     0.014739
     py      :     0.018022

  8 H s      :     0.792541  s :     0.792541
     pz      :     0.026483  p :     0.059432
     px      :     0.014893
     py      :     0.018055

  9 H s      :     0.793013  s :     0.793013
     pz      :     0.017129  p :     0.059057
     px      :     0.017836
     py      :     0.024091

 10 H s      :     0.794351  s :     0.794351
     pz      :     0.014251  p :     0.059491
     px      :     0.031970
     py      :     0.013270

 11 C s      :     2.766002  s :     2.766002
     pz      :     1.009828  p :     3.016895
     px      :     0.995516
     py      :     1.011552
     dz2     :     0.073267  d :     0.388359
     dxz     :     0.094748
     dyz     :     0.066734
     dx2y2   :     0.072013
     dxy     :     0.081596
     f0      :     0.005248  f :     0.038120
     f+1     :     0.006754
     f-1     :     0.004626
     f+2     :     0.005148
     f-2     :     0.005605
     f+3     :     0.006106
     f-3     :     0.004633

 12 H s      :     0.792886  s :     0.792886
     pz      :     0.014923  p :     0.058433
     px      :     0.015827
     py      :     0.027684

 13 H s      :     0.795649  s :     0.795649
     pz      :     0.024740  p :     0.058182
     px      :     0.017847
     py      :     0.015595

 14 C s      :     2.787088  s :     2.787088
     pz      :     1.039151  p :     3.069600
     px      :     1.034899
```

```
     py      :     0.995551
     dz2     :     0.094946  d :     0.412098
     dxz     :     0.095744
     dyz     :     0.082058
     dx2y2   :     0.096207
     dxy     :     0.043144
     f0      :     0.008733  f :     0.041729
     f+1     :     0.006546
     f-1     :     0.004488
     f+2     :     0.006839
     f-2     :     0.006351
     f+3     :     0.004378
     f-3     :     0.004395

 15 H s      :     0.653220  s :     0.653220
     pz      :     0.021409  p :     0.157663
     px      :     0.018349
     py      :     0.117906

 16 H s      :     0.792555  s :     0.792555
     pz      :     0.016890  p :     0.062012
     px      :     0.029692
     py      :     0.015431

 17 C s      :     2.872237  s :     2.872237
     pz      :     1.052044  p :     3.020684
     px      :     1.071946
     py      :     0.896694
     dz2     :     0.104401  d :     0.303320
     dxz     :     0.049767
     dyz     :     0.029103
     dx2y2   :     0.050199
     dxy     :     0.069851
     f0      :     0.003438  f :     0.028535
     f+1     :     0.006462
     f-1     :     0.002739
     f+2     :     0.003998
     f-2     :     0.005764
     f+3     :     0.002366
     f-3     :     0.003768

 18 H s      :     0.808532  s :     0.808532
     pz      :     0.035352  p :     0.064892
     px      :     0.014304
     py      :     0.015237

 19 H s      :     0.803719  s :     0.803719
     pz      :     0.013333  p :     0.066823
     px      :     0.034222
     py      :     0.019268

 20 Ses      :     7.327265  s :     7.327265
     pz      :     5.138893  p :    15.365076
     px      :     4.786536
     py      :     5.439647
     dz2     :     2.099393  d :    10.542346
     dxz     :     2.158717
     dyz     :     2.072116
     dx2y2   :     2.133226
     dxy     :     2.078894
     f0      :     0.013937  f :     0.107429
     f+1     :     0.029067
     f-1     :     0.003748
```

```
     f+2     :     0.023405
     f-2     :     0.018332
     f+3     :     0.009515
     f-3     :     0.009424

21 O s       :     3.625882  s :     3.625882
     pz      :     1.633892  p :     4.718217
     px      :     1.522896
     py      :     1.561429
     dz2     :     0.010714  d :     0.060980
     dxz     :     0.020249
     dyz     :     0.005300
     dx2y2   :     0.011888
     dxy     :     0.012829
     f0      :     0.000220  f :     0.002815
     f+1     :     0.000624
     f-1     :     0.000226
     f+2     :     0.000626
     f-2     :     0.000401
     f+3     :     0.000381
     f-3     :     0.000336

22 C s       :     2.785741  s :     2.785741
     pz      :     1.052535  p :     2.983726
     px      :     1.003808
     py      :     0.927383
     dz2     :     0.098473  d :     0.489512
     dxz     :     0.161149
     dyz     :     0.089642
     dx2y2   :     0.071431
     dxy     :     0.068817
     f0      :     0.007852  f :     0.049354
     f+1     :     0.012348
     f-1     :     0.007095
     f+2     :     0.004775
     f-2     :     0.007134
     f+3     :     0.005129
     f-3     :     0.005022

23 C s       :     2.763528  s :     2.763528
     pz      :     1.030256  p :     2.930888
     px      :     0.998308
     py      :     0.902323
     dz2     :     0.107188  d :     0.386085
     dxz     :     0.122351
     dyz     :     0.067473
     dx2y2   :     0.033641
     dxy     :     0.055432
     f0      :     0.007549  f :     0.044161
     f+1     :     0.012584
     f-1     :     0.005719
     f+2     :     0.005383
     f-2     :     0.005732
     f+3     :     0.002768
     f-3     :     0.004426

24 C s       :     2.762557  s :     2.762557
     pz      :     0.985848  p :     2.935336
     px      :     1.034257
     py      :     0.915231
     dz2     :     0.101425  d :     0.385983
     dxz     :     0.087092
     dyz     :     0.042647
```

```
     dx2y2   :     0.079231
     dxy     :     0.075588
     f0      :     0.003812  f :     0.043942
     f+1     :     0.011304
     f-1     :     0.003444
     f+2     :     0.006395
     f-2     :     0.007508
     f+3     :     0.005199
     f-3     :     0.006279

 25 C s      :     2.757569  s :     2.757569
     pz      :     0.985464  p :     2.901693
     px      :     1.017727
     py      :     0.898502
     dz2     :     0.098852  d :     0.382733
     dxz     :     0.087782
     dyz     :     0.041260
     dx2y2   :     0.077958
     dxy     :     0.076882
     f0      :     0.003902  f :     0.043977
     f+1     :     0.011253
     f-1     :     0.003290
     f+2     :     0.006575
     f-2     :     0.007578
     f+3     :     0.005340
     f-3     :     0.006038

 26 C s      :     2.757217  s :     2.757217
     pz      :     1.013108  p :     2.901836
     px      :     0.993640
     py      :     0.895088
     dz2     :     0.105558  d :     0.382368
     dxz     :     0.114506
     dyz     :     0.070159
     dx2y2   :     0.034433
     dxy     :     0.057712
     f0      :     0.007514  f :     0.043956
     f+1     :     0.012322
     f-1     :     0.005628
     f+2     :     0.005530
     f-2     :     0.005616
     f+3     :     0.002847
     f-3     :     0.004500

 27 C s      :     2.711180  s :     2.711180
     pz      :     1.004006  p :     2.882847
     px      :     0.956864
     py      :     0.921977
     dz2     :     0.104236  d :     0.484495
     dxz     :     0.158395
     dyz     :     0.085403
     dx2y2   :     0.079325
     dxy     :     0.057137
     f0      :     0.008231  f :     0.055962
     f+1     :     0.014344
     f-1     :     0.007195
     f+2     :     0.006433
     f-2     :     0.008059
     f+3     :     0.006176
     f-3     :     0.005525

 28 H s      :     0.784498  s :     0.784498
     pz      :     0.014376  p :     0.060871
```

```
     px      :     0.028017
     py      :     0.018478

29 H s       :     0.782594  s :     0.782594
     pz      :     0.033708  p :     0.059936
     px      :     0.010712
     py      :     0.015516

30 H s       :     0.778392  s :     0.778392
     pz      :     0.034358  p :     0.060597
     px      :     0.010932
     py      :     0.015307

31 H s       :     0.778130  s :     0.778130
     pz      :     0.014719  p :     0.060578
     px      :     0.026920
     py      :     0.018938

32 C s       :     2.749811  s :     2.749811
     pz      :     0.825814  p :     2.492250
     px      :     0.895450
     py      :     0.770987
     dz2     :     0.177844  d :     1.113373
     dxz     :     0.239505
     dyz     :     0.267246
     dx2y2   :     0.250292
     dxy     :     0.178487
     f0      :     0.029481  f :     0.248409
     f+1     :     0.030221
     f-1     :     0.043118
     f+2     :     0.039333
     f-2     :     0.039904
     f+3     :     0.033695
     f-3     :     0.032657

33 F s       :     3.612646  s :     3.612646
     pz      :     1.758809  p :     5.187756
     px      :     1.830608
     py      :     1.598339
     dz2     :     0.002779  d :     0.028173
     dxz     :     0.001762
     dyz     :     0.010540
     dx2y2   :     0.008461
     dxy     :     0.004632
     f0      :     0.000150  f :     0.001453
     f+1     :     0.000007
     f-1     :     0.000253
     f+2     :     0.000389
     f-2     :     0.000187
     f+3     :     0.000198
     f-3     :     0.000269

34 F s       :     3.602816  s :     3.602816
     pz      :     1.673734  p :     5.181950
     px      :     1.816585
     py      :     1.691632
     dz2     :     0.005099  d :     0.028942
     dxz     :     0.003938
     dyz     :     0.012320
     dx2y2   :     0.004546
     dxy     :     0.003039
     f0      :     0.000146  f :     0.001515
     f+1     :     0.000100
```

```
     f-1     :     0.000449
     f+2     :     0.000377
     f-2     :     0.000251
     f+3     :     0.000080
     f-3     :     0.000112

35 F s       :     3.603595  s :     3.603595
     pz      :     1.749724  p :     5.182993
     px      :     1.608827
     py      :     1.824442
     dz2     :     0.004060  d :     0.028862
     dxz     :     0.011469
     dyz     :     0.001809
     dx2y2   :     0.008039
     dxy     :     0.003485
     f0      :     0.000150  f :     0.001508
     f+1     :     0.000358
     f-1     :     0.000015
     f+2     :     0.000422
     f-2     :     0.000185
     f+3     :     0.000235
     f-3     :     0.000143


                     *****************************
                     * MAYER POPULATION ANALYSIS *
                     *****************************

  NA   - Mulliken gross atomic population
  ZA   - Total nuclear charge
  QA   - Mulliken gross atomic charge
  VA   - Mayer's total valence
  BVA  - Mayer's bonded valence
  FA   - Mayer's free valence
```

ATOM	NA	ZA	QA	VA	BVA	FA
0 C	5.8249	6.0000	0.1751	3.5748	3.5748	-0.0000
1 C	6.2293	6.0000	-0.2293	3.9181	3.9181	0.0000
2 C	6.2420	6.0000	-0.2420	3.8720	3.8720	0.0000
3 C	6.1064	6.0000	-0.1064	3.8349	3.8349	-0.0000
4 C	6.1255	6.0000	-0.1255	3.8285	3.8285	0.0000
5 C	6.1719	6.0000	-0.1719	3.8411	3.8411	0.0000
6 H	0.8742	1.0000	0.1258	0.9823	0.9823	0.0000
7 H	0.8637	1.0000	0.1363	0.9687	0.9687	0.0000
8 H	0.8615	1.0000	0.1385	0.9741	0.9741	0.0000
9 H	0.8628	1.0000	0.1372	0.9646	0.9646	0.0000
10 H	0.8638	1.0000	0.1362	0.9768	0.9768	0.0000
11 C	6.1794	6.0000	-0.1794	3.8441	3.8441	0.0000
12 H	0.8858	1.0000	0.1142	0.9603	0.9603	0.0000
13 H	0.8826	1.0000	0.1174	0.9641	0.9641	0.0000
14 C	6.2674	6.0000	-0.2674	3.7564	3.7564	0.0000
15 H	0.7427	1.0000	0.2573	1.0134	1.0134	-0.0000
16 H	0.8709	1.0000	0.1291	0.9631	0.9631	0.0000
17 C	6.2981	6.0000	-0.2981	3.8945	3.8945	0.0000
18 H	0.8353	1.0000	0.1647	0.9596	0.9596	0.0000
19 H	0.8323	1.0000	0.1677	0.9594	0.9594	0.0000
20 Se	33.5688	34.0000	0.4312	2.9541	2.9541	-0.0000
21 O	8.5716	8.0000	-0.5716	1.8244	1.8244	-0.0000
22 C	5.9372	6.0000	0.0628	3.6817	3.6817	-0.0000
23 C	6.1650	6.0000	-0.1650	3.7976	3.7976	-0.0000
24 C	6.1843	6.0000	-0.1843	3.8471	3.8471	0.0000
25 C	6.2205	6.0000	-0.2205	3.8995	3.8995	0.0000

```
 26 C      6.2054     6.0000    -0.2054     3.8869     3.8869    -0.0000
 27 C      5.8862     6.0000     0.1138     3.5011     3.5011    -0.0000
 28 H      0.8552     1.0000     0.1448     0.9966     0.9966     0.0000
 29 H      0.8441     1.0000     0.1559     0.9657     0.9657     0.0000
 30 H      0.8470     1.0000     0.1530     0.9789     0.9789     0.0000
 31 H      0.8452     1.0000     0.1548     0.9779     0.9779    -0.0000
 32 C      5.4720     6.0000     0.5280     4.4368     4.4368    -0.0000
 33 F      9.1931     9.0000    -0.1931     1.0865     1.0865    -0.0000
 34 F      9.1919     9.0000    -0.1919     1.1079     1.1079     0.0000
 35 F      9.1924     9.0000    -0.1924     1.1059     1.1059    -0.0000

  Mayer bond orders larger than 0.100000
B(  0-C ,  1-C ) :   1.3464 B(  0-C ,  2-C ) :   1.3241 B(  0-C , 11-C ) :   0.9086 
B(  1-C ,  3-C ) :   1.4007 B(  1-C ,  6-H ) :   0.9773 B(  2-C ,  4-C ) :   1.3942 
B(  2-C ,  7-H ) :   0.9939 B(  3-C ,  5-C ) :   1.3757 B(  3-C ,  8-H ) :   0.9706 
B(  4-C ,  5-C ) :   1.3666 B(  4-C ,  9-H ) :   0.9836 B(  5-C , 10-H ) :   0.9776 
B( 11-C , 12-H ) :   0.9500 B( 11-C , 13-H ) :   0.9796 B( 11-C , 14-C ) :   0.9554 
B( 14-C , 15-H ) :   0.5478 B( 14-C , 16-H ) :   0.9417 B( 14-C , 17-C ) :   1.2981 
B( 15-H , 21-O ) :   0.3898 B( 17-C , 18-H ) :   0.9561 B( 17-C , 19-H ) :   0.9512 
B( 17-C , 20-Se) :   0.4992 B( 17-C , 21-O ) :   0.1001 B( 20-Se, 21-O ) :   1.2708 
B( 20-Se, 22-C ) :   0.9670 B( 22-C , 23-C ) :   1.2957 B( 22-C , 24-C ) :   1.3272 
B( 23-C , 25-C ) :   1.3823 B( 23-C , 28-H ) :   0.9766 B( 24-C , 26-C ) :   1.3905 
B( 24-C , 29-H ) :   0.9794 B( 25-C , 27-C ) :   1.2947 B( 25-C , 30-H ) :   0.9876 
B( 26-C , 27-C ) :   1.3136 B( 26-C , 31-H ) :   0.9761 B( 27-C , 32-C ) :   1.0096 
B( 32-C , 33-F ) :   1.1230 B( 32-C , 34-F ) :   1.1254 B( 32-C , 35-F ) :   1.1250 


-------
TIMINGS
-------

Total SCF time: 0 days 0 hours 2 min 23 sec

Total time                  ....     143.518 sec
Sum of individual times     ....     139.177 sec  ( 97.0%)

SCF preparation             ....       0.777 sec  (  0.5%)
Fock matrix formation       ....     132.863 sec  ( 92.6%)
  Startup                   ....       0.063 sec  (  0.0% of F)
  Split-RI-J                ....      10.280 sec  (  7.7% of F)
  Chain of spheres X        ....      99.557 sec  ( 74.9% of F)
  XC integration            ....      12.686 sec  (  9.5% of F)
    XC Preparation          ....       0.000 sec  (  0.0% of XC)
    Basis function eval.    ....       3.132 sec  ( 24.7% of XC)
    Density eval.           ....       4.148 sec  ( 32.7% of XC)
    XC-Functional eval.     ....       0.117 sec  (  0.9% of XC)
    XC-Potential eval.      ....       4.290 sec  ( 33.8% of XC)
  CPCM terms                ....      13.953 sec  ( 10.5% of F)
Diagonalization             ....       0.000 sec  (  0.0%)
Density matrix formation    ....       0.517 sec  (  0.4%)
Total Energy calculation    ....       0.236 sec  (  0.2%)
Population analysis         ....       0.183 sec  (  0.1%)
Orbital Transformation      ....       0.509 sec  (  0.4%)
Orbital Orthonormalization  ....       0.000 sec  (  0.0%)
DIIS solution               ....       2.112 sec  (  1.5%)
SOSCF solution              ....       1.980 sec  (  1.4%)
Finished LeanSCF after 143.9 sec

Maximum memory used throughout the entire LEANSCF-calculation: 113.9 MB


-------------------------------------------------------------------------------
                          DFT DISPERSION CORRECTION
```

```
                              DFTD3 V3.1  Rev 1
                          USING Becke-Johnson damping
-------------------------------------------------------------------------------
The B3LYP functional is recognized
Active option DFTDOPT                  ...         4

molecular C6(AA) [au] = 16726.511109


            DFT-D V3
 parameters
 s6 scaling factor         :     1.0000
 a1 scaling factor         :     0.3981
 s8 scaling factor         :     1.9889
 a2 scaling factor         :     4.4211
 ad hoc parameters k1-k3   :    16.0000     1.3333    -4.0000

 Edisp/kcal,au: -55.799521586464  -0.088922188334
 E6   /kcal   : -25.880176159
 E8   /kcal   : -29.919345427

-------------------------   ----------------
Dispersion correction           -0.088922188
-------------------------   ----------------



-------------------------   --------------------
FINAL SINGLE POINT ENERGY     -3394.828612941195
-------------------------   --------------------



           ************************************************************
           *        Program running with 12 parallel MPI-processes    *
           *              working on a common directory               *
           ************************************************************
------------------------------------------------------------------------------
                         ORCA SCF GRADIENT CALCULATION
------------------------------------------------------------------------------

Nuc. rep. gradient        (SHARK) ... done (  0.0 sec)
HCore & Overlap gradient  (SHARK) ... done (  0.5 sec)
Split-RIJ-J gradient      (SHARK) ... done (  2.7 sec)
COSX-gradient                     ... done ( 44.2 sec)
XC gradient                       ... done (  6.4 sec)
CPCM gradient                     ... done (  2.5 sec)
SMD gradient                      ... done (  0.0 sec)
Dispersion correction             ... done (  0.0 sec)

-------------------
DISPERSION GRADIENT
-------------------

   1   C   :   -0.000269369    0.000895540    0.000490364
   2   C   :   -0.000254730    0.000996461    0.000990823
   3   C   :    0.000171401    0.000847715    0.000221606
   4   C   :   -0.000307466    0.001037528    0.000790187
   5   C   :    0.000187658    0.000732164    0.000103331
   6   C   :   -0.000062660    0.000932165    0.000323015
   7   H   :   -0.000235429    0.000093755    0.000547239
   8   H   :    0.000021044    0.000431950   -0.000164341
   9   H   :   -0.000101886    0.000249904    0.000494642
```

```
  10   H   :    0.000157209    0.000282874   -0.000114546
  11   H   :    0.000190026    0.000337461    0.000142053
  12   C   :   -0.000692623    0.000394547    0.000039713
  13   H   :   -0.000367442    0.000233903    0.000024595
  14   H   :   -0.000406735    0.000103592    0.000202583
  15   C   :   -0.000753934   -0.000358235   -0.000183206
  16   H   :   -0.000074073   -0.000861139   -0.000174462
  17   H   :   -0.000363662    0.000073145   -0.000157534
  18   C   :   -0.000013261   -0.001651396   -0.000595800
  19   H   :   -0.000071450    0.000111128   -0.000294102
  20   H   :   -0.000195470    0.000115435   -0.000637346
  21   Se  :   -0.000991694   -0.000253612   -0.001636835
  22   O   :   -0.000895334    0.000317589   -0.000264198
  23   C   :    0.000672505   -0.000983967   -0.000216918
  24   C   :    0.000421929   -0.000839995   -0.000062812
  25   C   :    0.000617786   -0.000695726   -0.000340179
  26   C   :    0.000281413   -0.000754993    0.000167281
  27   C   :    0.000591652   -0.000431305   -0.000431913
  28   C   :    0.000470104   -0.000532517   -0.000076017
  29   H   :   -0.000050395   -0.000389273    0.000275875
  30   H   :    0.000338403    0.000001027   -0.000342751
  31   H   :   -0.000022741   -0.000298138    0.000289838
  32   H   :    0.000286132    0.000053611   -0.000282179
  33   C   :    0.000609524   -0.000139107    0.000257586
  34   F   :    0.000504864    0.000050058    0.000328816
  35   F   :    0.000258682   -0.000097863    0.000224378
  36   F   :    0.000350019   -0.000004287    0.000061213

Difference to translation invariance:
           :    0.0000000000    0.0000000000   -0.0000000000

Difference to rotation invariance:
           :   -0.0000000000    0.0000000000    0.0000000000

Norm of the Dispersion gradient    ...    0.0051642896
RMS gradient                       ...    0.0004969340
MAX gradient                       ...    0.0016513956

------------------
CARTESIAN GRADIENT
------------------

   1   C   :    0.000009379   -0.000013694   -0.000012338
   2   C   :    0.000000733   -0.000002319    0.000003480
   3   C   :   -0.000003453    0.000003770    0.000009041
   4   C   :   -0.000000571    0.000001115    0.000005180
   5   C   :   -0.000001660    0.000002337    0.000009017
   6   C   :   -0.000000559   -0.000001773    0.000003932
   7   H   :    0.000004128   -0.000009762   -0.000003793
   8   H   :    0.000000223    0.000002412    0.000005812
   9   H   :   -0.000024673    0.000005259    0.000007607
  10   H   :    0.000000693   -0.000001304    0.000005679
  11   H   :   -0.000000927   -0.000003406    0.000005033
  12   C   :   -0.000002928    0.000011387    0.000009490
  13   H   :   -0.000001404   -0.000000138    0.000001829
  14   H   :    0.000000317   -0.000000607    0.000002747
  15   C   :   -0.000022306    0.000147407   -0.000092803
  16   H   :    0.000037990   -0.000194369    0.000103897
  17   H   :    0.000001399    0.000029431   -0.000009910
  18   C   :   -0.000011443    0.000034493    0.000003685
  19   H   :    0.000005158   -0.000009375    0.000001504
  20   H   :   -0.000004487    0.000015283   -0.000000822
  21   Se  :    0.000027010   -0.000028723   -0.000009868
```

```
  22   O   :   -0.000027333    0.000016756   -0.000013502
  23   C   :   -0.000004700    0.000007094   -0.000004901
  24   C   :    0.000007128   -0.000009386   -0.000005747
  25   C   :   -0.000011067    0.000022306    0.000004756
  26   C   :   -0.000006938    0.000010187    0.000000378
  27   C   :    0.000008911   -0.000016101   -0.000008599
  28   C   :    0.000000385   -0.000008656   -0.000002317
  29   H   :   -0.000000689    0.000001243   -0.000004665
  30   H   :    0.000000125    0.000006413   -0.000001032
  31   H   :   -0.000001049   -0.000002759   -0.000004293
  32   H   :   -0.000000706    0.000003898    0.000000291
  33   C   :    0.000003583    0.000003663   -0.000009981
  34   F   :    0.000015075   -0.000016285    0.000013272
  35   F   :    0.000005390   -0.000010142   -0.000011801
  36   F   :   -0.000000736    0.000004345   -0.000000259

Difference to translation invariance:
           :   -0.0000000003   -0.0000000008   -0.0000000018

Difference to rotation invariance:
           :    0.0007460509    0.0000155343   -0.0001362934

Norm of the Cartesian gradient     ...    0.0003016701
RMS gradient                       ...    0.0000290282
MAX gradient                       ...    0.0001943686

-------
TIMINGS
-------

Total SCF gradient time     ....       56.267 sec

Densities                   ....        0.008 sec  (  0.0%)
One electron gradient       ....        0.544 sec  (  1.0%)
RI-J Coulomb gradient       ....        2.658 sec  (  4.7%)
COSX gradient               ....       44.154 sec  ( 78.5%)
XC gradient                 ....        6.369 sec  ( 11.3%)
CPCM gradient               ....        2.487 sec  (  4.4%)
   A-Matrix (El+Nuc)        ....        0.025 sec  (  0.0%)
   Potential                ....        2.462 sec  (  4.4%)
SMD gradient                ....        0.004 sec  (  0.0%)

Maximum memory used throughout the entire SCFGRAD-calculation: 131.2 MB


           ************************************************************
           *        Program running with 12 parallel MPI-processes    *
           *              working on a common directory               *
           ************************************************************

------------------------------------------------------------------------------
                              ORCA PROPERTY CALCULATIONS
------------------------------------------------------------------------------

GBWName                                    ... CF3-IRC.gbw
Number of atoms                            ...     36
Number of basis functions                  ...    758
Max core memory                            ...   8000 MB

Electric properties:
Dipole moment                              ... YES
Quadrupole moment                          ...  NO
General moments                            ...  NO (MaxL=10)
```

```
Static polarizability (Dipole/Dipole)   ...  NO
Static polarizability (Dipole/Quad.)    ...  NO
Static polarizability (Quad./Quad.)     ...  NO
Static polarizability (Velocity)        ...  NO
Static hyperpolarizability              ...  NO

Atomic electric properties:
Dipole moment                           ...  NO
Quadrupole moment                       ...  NO
Static polarizability                   ...  NO

Choice of electric origin               ... Center of mass
Position of electric origin             ...     0.269278    -3.443187   -12.003581

General magnetic properties:
Magnetizability                         ...  NO

EPR properties:
g-Tensor (aka g-matrix)                 ...  NO
Zero-Field splitting spin-orbit         ...  NO
Zero-field splitting spin-spin          ...  NO
Hyperfine couplings                     ...  NO (   0 nuclei)
Quadrupole couplings                    ...  NO (   0 nuclei)
Contact density                         ...  NO (   0 nuclei)

NMR properties:
Chemical shifts                         ...  NO (   0 nuclei)
Spin-rotation constants                 ...  NO (   0 nuclei)
Spin-spin couplings                     ...  NO (   0 nuclei,    0 pairs)

Choice of magnetic origin               ... GIAO
Position of magnetic origin             ...     0.000000     0.000000     0.000000

Properties with geometric perturbations:
SCF Hessian                             ...  NO
IR spectrum                             ...  NO
Raman spectrum                          ...  NO
Raman option                            ... 0
VCD spectrum                            ...  NO
X-ray spectroscopy properties:
SCF XES/XAS/RIXS spectra                ...  NO

SCF SOC stabilization energy            ...  NO
Diagonal Born-Oppenheimer correction    ...  NO

-------------
DIPOLE MOMENT
-------------

Method              : SCF
Type of density     : Electron Density
Multiplicity        :   1
Irrep               :   0
Energy              : -3394.7396907528609518 Eh
Basis               : AO
                                X                 Y                 Z
Electronic contribution:      9.152401925     -31.424453668     -11.109369683
Nuclear contribution   :     -9.399174797      31.981673335       9.599057057
                        -----------------------------------------
Total Dipole Moment    :     -0.246772872       0.557219667      -1.510312626
                        -----------------------------------------
Magnitude (a.u.)       :      1.628629741
Magnitude (Debye)      :      4.139647820
```

```
--------------------
Rotational spectrum
--------------------

Rotational constants in cm-1:          0.012725          0.006182          0.004781
Rotational constants in MHz :        381.495603        185.345157        143.343295

Dipole components along the rotational axes:
x,y,z [a.u.] :    -0.947666    -0.134158    -1.317712
x,y,z [Debye]:    -2.408775    -0.341002    -3.349359



Dipole moment calculation done in   0.0 sec

Maximum memory used throughout the entire PROP-calculation: 68.3 MB


-------------------------------------------------------------------------------
                   Intrinsic Reaction Coordinate Calculation
-------------------------------------------------------------------------------

IRC Settings:
  Nr. of atoms                        .... 36
  Algorithm                           .... LQA
  Coordinate system following type    .... Mass-Weighted
  Max. no of cycles (MaxIter)         .... 241
  Direction                           .... Forward and backward
  Steep Lenght                        .... 0.110 bohr Dalton^(1/2)

Initial displacement:
  Hessian type                        .... Read
  Hessian Filename                    .... TS-CF3_Compound_3.hess
  Following Hessian mode              .... 0
  Initial displacement type           .... Length
    Scaling normalized TS mode by     .... 0.040

Convergence Tolerances:
  Energy Change                       ....  5.0000e-06 Eh
  Max. Gradient                       ....  8.0000e-05 Eh/bohr
  RMS Gradient                        ....  1.0000e-05 Eh/bohr
  Max. Displacement                   ....  4.0000e-03 bohr
  RMS Displacement                    ....  2.0000e-03 bohr
  Strict Convergency criteria enforced

LQA Settings:
  Hessian update type                 .... Bofill
  Update steep lenght                 .... NO
  t Stepsize                          .... Gradient-based adjusted
  t MAX int. Steps                    .... 1000000
  Bulirsch-Stoer Correction           .... YES
    Euler Initial Steps               .... 100
    Richardson Max extrapolations     .... 20
    Richardson error threshould       ....  1.0000e-06
    Apply BS correction on TS disp.   .... NO
    Keep LQA direction                .... NO
    LQA direction Theshould           ....  1.0000e-01
    Update Hessian and Gradient       .... YES

Storing full IRC trajectory in        .... CF3-IRC_IRC_Full_trj.xyz
```

```
Storing forward trajectory in        .... CF3-IRC_IRC_F_trj.xyz
Storing backward trajectory in       .... CF3-IRC_IRC_B_trj.xyz
Storing IRC profile in               .... CF3-IRC.IRC.out


         *************************************************************
         *                          FORWARD IRC                      *
         *************************************************************

Iteration    E(Eh)      dE(kcal/mol)  max(|G|)   RMS(G)   max(|S|)   RMS(S)   MW-Displ.
Convergence thresholds    0.003138    0.000080  0.000010  0.004000  0.002000
    0     -3394.828667   -0.034022    0.002318  0.000496  0.035537  0.003849  0.046693
    1     -3394.829061   -0.281125    0.007436  0.001409  0.076258  0.008563  0.110002
    2     -3394.829653   -0.652738    0.011450  0.002031  0.061141  0.007627  0.110005
    3     -3394.830315   -1.068029    0.014084  0.002420  0.042770  0.006588  0.110001
    4     -3394.831011   -1.504492    0.015718  0.002671  0.030861  0.005843  0.110004
    5     -3394.831731   -1.956306    0.016731  0.002846  0.029389  0.005362  0.110003
    6     -3394.832471   -2.421271    0.017977  0.002976  0.027981  0.005049  0.110004
    7     -3394.833227   -2.895211    0.019136  0.003080  0.026763  0.004839  0.110003
    8     -3394.833996   -3.377661    0.020132  0.003165  0.025686  0.004689  0.110002
    9     -3394.834777   -3.867864    0.021007  0.003238  0.024688  0.004576  0.110002
   10     -3394.835565   -4.362160    0.021786  0.003296  0.023813  0.004489  0.110001
   11     -3394.836360   -4.861223    0.022486  0.003352  0.023055  0.004424  0.110000
   12     -3394.837164   -5.365783    0.023115  0.003403  0.022319  0.004366  0.110003
   13     -3394.837975   -5.874626    0.023680  0.003449  0.021677  0.004320  0.110001
   14     -3394.838794   -6.388457    0.024184  0.003488  0.021110  0.004284  0.110001
   15     -3394.839615   -6.903625    0.024623  0.003523  0.020614  0.004257  0.110001
   16     -3394.840443   -7.423401    0.025000  0.003551  0.020175  0.004236  0.110004
   17     -3394.841271   -7.943228    0.025308  0.003572  0.019806  0.004221  0.110003
   18     -3394.842102   -8.464600    0.025546  0.003587  0.019479  0.004212  0.110001
   19     -3394.842935   -8.987421    0.025712  0.003594  0.019228  0.004207  0.110002
   20     -3394.843773   -9.513372    0.025808  0.003594  0.019046  0.004207  0.110001
   21     -3394.844605   -10.035301    0.025823  0.003585  0.018896  0.004210  0.110003
   22     -3394.845433   -10.554844    0.025758  0.003566  0.018725  0.004213  0.110003
   23     -3394.846244   -11.063407    0.025605  0.003537  0.018505  0.004214  0.110003
   24     -3394.847053   -11.571462    0.025375  0.003497  0.018177  0.004212  0.110002
   25     -3394.847850   -12.071221    0.025065  0.003448  0.017818  0.004206  0.110002
   26     -3394.848628   -12.559561    0.024678  0.003391  0.017392  0.004190  0.110001
   27     -3394.849383   -13.033343    0.024209  0.003326  0.016928  0.004151  0.110002
   28     -3394.850115   -13.492545    0.023647  0.003248  0.016609  0.004105  0.110003
   29     -3394.850826   -13.938660    0.023000  0.003160  0.016400  0.004087  0.110000
   30     -3394.851514   -14.370299    0.022276  0.003062  0.016096  0.004076  0.110003
   31     -3394.852176   -14.785990    0.021476  0.002957  0.015694  0.004052  0.110001
   32     -3394.852809   -15.183425    0.020602  0.002843  0.015279  0.004023  0.110001
   33     -3394.853413   -15.562399    0.019655  0.002720  0.014850  0.003991  0.110003
   34     -3394.853986   -15.922026    0.018638  0.002591  0.014381  0.003958  0.110000
   35     -3394.854527   -16.261558    0.017556  0.002454  0.014269  0.003921  0.110002
   36     -3394.855036   -16.580472    0.016414  0.002311  0.014457  0.003882  0.110000
   37     -3394.855510   -16.878329    0.015218  0.002164  0.014676  0.003841  0.110001
   38     -3394.855951   -17.154571    0.013979  0.002012  0.014923  0.003799  0.110001
   39     -3394.856358   -17.410144    0.012706  0.001858  0.015195  0.003757  0.110002
   40     -3394.856731   -17.644509    0.011416  0.001705  0.015492  0.003717  0.110002
   41     -3394.857073   -17.859222    0.010121  0.001549  0.015780  0.003680  0.110002
   42     -3394.857382   -18.052806    0.008851  0.001408  0.016091  0.003655  0.110002
   43     -3394.857668   -18.232019    0.007603  0.001245  0.016290  0.003642  0.110000
   44     -3394.857908   -18.382806    0.006568  0.001179  0.016628  0.003670  0.110000
   45     -3394.858157   -18.539434    0.006054  0.000949  0.016269  0.003801  0.110000
   46     -3394.858344   -18.656508    0.005530  0.001114  0.018239  0.004568  0.110001
   47     -3394.858566   -18.795980    0.005051  0.000735  0.015601  0.003888  0.110001
   48     -3394.858744   -18.907727    0.004478  0.000733  0.017987  0.004444  0.110002
   49     -3394.858917   -19.015766    0.004001  0.000610  0.017720  0.004335  0.110001
   50     -3394.859079   -19.117847    0.003575  0.000542  0.019898  0.004747  0.110001
   51     -3394.859230   -19.212186    0.003202  0.000523  0.021317  0.005086  0.110001
```

52	-3394.859377	-19.304793	0.002897	0.000494	0.022412	0.005430	0.110000
53	-3394.859524	-19.396759	0.002646	0.000476	0.023983	0.005649	0.110001
54	-3394.859669	-19.488221	0.002444	0.000466	0.024407	0.005777	0.110000
55	-3394.859816	-19.579977	0.002281	0.000457	0.024805	0.005873	0.110001
56	-3394.859961	-19.671241	0.002152	0.000447	0.025095	0.005961	0.110000
57	-3394.860108	-19.763144	0.002046	0.000436	0.025263	0.006048	0.110001
58	-3394.860254	-19.854874	0.001959	0.000436	0.024904	0.006111	0.110000
59	-3394.860396	-19.944082	0.001953	0.000478	0.024526	0.006135	0.110001
60	-3394.860535	-20.031242	0.002294	0.000495	0.023851	0.006000	0.110001
61	-3394.860675	-20.119141	0.002197	0.000488	0.024533	0.005847	0.110000
62	-3394.860817	-20.208319	0.002104	0.000482	0.024652	0.005826	0.110001
63	-3394.860959	-20.297385	0.002121	0.000483	0.024773	0.005862	0.110000
64	-3394.861100	-20.386088	0.002147	0.000488	0.024930	0.005910	0.110001
65	-3394.861242	-20.474822	0.002134	0.000487	0.024952	0.005950	0.110000
66	-3394.861383	-20.563420	0.002150	0.000489	0.024749	0.005951	0.110001
67	-3394.861524	-20.652021	0.002131	0.000488	0.024567	0.005925	0.110001
68	-3394.861664	-20.739992	0.002164	0.000493	0.024322	0.005889	0.110000
69	-3394.861804	-20.827606	0.002170	0.000495	0.024156	0.005868	0.110001
70	-3394.861943	-20.914693	0.002165	0.000495	0.024065	0.005857	0.110001
71	-3394.862080	-21.001096	0.002165	0.000495	0.023920	0.005840	0.110001
72	-3394.862217	-21.086666	0.002169	0.000496	0.023765	0.005815	0.110000
73	-3394.862352	-21.171346	0.002190	0.000497	0.023620	0.005789	0.110000
74	-3394.862485	-21.255063	0.002217	0.000497	0.023476	0.005765	0.110001
75	-3394.862617	-21.337689	0.002249	0.000498	0.023317	0.005738	0.110001
76	-3394.862747	-21.419160	0.002292	0.000501	0.023122	0.005703	0.110000
77	-3394.862875	-21.499776	0.002283	0.000496	0.023138	0.005688	0.110000
78	-3394.863001	-21.579082	0.002316	0.000497	0.022957	0.005669	0.110000
79	-3394.863126	-21.656987	0.002329	0.000495	0.022845	0.005642	0.110000
80	-3394.863249	-21.734390	0.002348	0.000494	0.022693	0.005621	0.110000
81	-3394.863370	-21.810172	0.002367	0.000494	0.022508	0.005593	0.110000
82	-3394.863488	-21.884666	0.002380	0.000492	0.022336	0.005565	0.110001
83	-3394.863605	-21.957752	0.002394	0.000490	0.022144	0.005534	0.110000
84	-3394.863719	-22.029437	0.002402	0.000488	0.022181	0.005499	0.110000
85	-3394.863831	-22.099575	0.002411	0.000487	0.022197	0.005460	0.110001
86	-3394.863940	-22.168135	0.002413	0.000484	0.022167	0.005417	0.110001
87	-3394.864047	-22.235013	0.002417	0.000482	0.022113	0.005370	0.110000
88	-3394.864151	-22.300184	0.002411	0.000479	0.022014	0.005320	0.110000
89	-3394.864251	-22.363477	0.002410	0.000477	0.021891	0.005267	0.110000
90	-3394.864349	-22.424958	0.002391	0.000473	0.021717	0.005211	0.110000
91	-3394.864444	-22.484349	0.002382	0.000471	0.021534	0.005151	0.110000
92	-3394.864536	-22.541897	0.002335	0.000465	0.021293	0.005089	0.110000
93	-3394.864624	-22.597092	0.002310	0.000464	0.021078	0.005025	0.110000
94	-3394.864709	-22.650706	0.002193	0.000453	0.020792	0.004958	0.110000
95	-3394.864790	-22.701553	0.002140	0.000456	0.020595	0.004893	0.110001
96	-3394.864870	-22.751568	0.001880	0.000432	0.020244	0.004810	0.110000
97	-3394.864948	-22.800615	0.001842	0.000454	0.020046	0.004754	0.110001
98	-3394.865025	-22.848855	0.001394	0.000398	0.019487	0.004625	0.110001
99	-3394.865083	-22.885130	0.001933	0.000476	0.019225	0.004726	0.110000
100	-3394.865158	-22.932556	0.001303	0.000318	0.018572	0.004407	0.110001
101	-3394.865220	-22.971443	0.001671	0.000405	0.020719	0.005031	0.110000
102	-3394.865299	-23.020684	0.001132	0.000250	0.024572	0.005332	0.110001
103	-3394.865350	-23.052627	0.002717	0.000519	0.025499	0.006108	0.110001
104	-3394.865408	-23.089274	0.002624	0.000479	0.016675	0.004223	0.110000
105	-3394.865459	-23.121454	0.002738	0.000488	0.018029	0.004508	0.110000
106	-3394.865466	-23.125538	0.002962	0.000518	0.011478	0.002050	0.110000
107	-3394.865529	-23.164907	0.001032	0.000266	0.019198	0.004408	0.110000
108	-3394.865584	-23.199555	0.000960	0.000257	0.020019	0.005096	0.110001
109	-3394.865633	-23.230308	0.000931	0.000265	0.020536	0.004943	0.110000
110	-3394.865679	-23.259341	0.001516	0.000298	0.019484	0.004706	0.110000
111	-3394.865726	-23.289013	0.001642	0.000292	0.017306	0.004325	0.110001
112	-3394.865771	-23.316729	0.001631	0.000293	0.017251	0.004366	0.110000
113	-3394.865812	-23.342811	0.001656	0.000294	0.016334	0.004299	0.110000
114	-3394.865854	-23.368833	0.001653	0.000292	0.015854	0.004249	0.110000

115	-3394.865893	-23.393356	0.001640	0.000293	0.015579	0.004212	0.110000
116	-3394.865930	-23.416873	0.001611	0.000290	0.015013	0.004141	0.110000
117	-3394.865967	-23.440107	0.001571	0.000285	0.014963	0.004106	0.110000
118	-3394.866001	-23.461436	0.001581	0.000285	0.014898	0.004098	0.110000
119	-3394.866035	-23.482528	0.001601	0.000282	0.015103	0.004006	0.110000
120	-3394.866067	-23.502823	0.001613	0.000281	0.015155	0.004009	0.110000
121	-3394.866100	-23.523265	0.001621	0.000280	0.015054	0.004025	0.110000
122	-3394.866131	-23.542732	0.001626	0.000281	0.014885	0.004009	0.110000
123	-3394.866161	-23.561418	0.001628	0.000281	0.014668	0.003991	0.110000
124	-3394.866191	-23.580622	0.001616	0.000280	0.014302	0.003949	0.110000
125	-3394.866220	-23.598529	0.001627	0.000281	0.014279	0.003969	0.110000
126	-3394.866248	-23.616582	0.001611	0.000278	0.013785	0.003898	0.110000
127	-3394.866277	-23.634414	0.001600	0.000274	0.014020	0.003948	0.110000
128	-3394.866305	-23.652003	0.001557	0.000272	0.013450	0.003839	0.110000
129	-3394.866332	-23.668704	0.001529	0.000265	0.013971	0.003908	0.110000
130	-3394.866357	-23.685004	0.001453	0.000260	0.013219	0.003748	0.110000
131	-3394.866383	-23.701148	0.001615	0.000278	0.014077	0.003918	0.110000
132	-3394.866410	-23.717926	0.001543	0.000267	0.012918	0.003714	0.110000
133	-3394.866435	-23.733581	0.001653	0.000282	0.013841	0.003887	0.110000
134	-3394.866461	-23.749807	0.001606	0.000273	0.013087	0.003746	0.110000
135	-3394.866485	-23.765229	0.001655	0.000281	0.013762	0.003864	0.110000
136	-3394.866510	-23.780950	0.001635	0.000277	0.013345	0.003768	0.110000
137	-3394.866535	-23.796253	0.001628	0.000276	0.013620	0.003832	0.110000
138	-3394.866559	-23.811296	0.001661	0.000281	0.013586	0.003780	0.110000
139	-3394.866583	-23.826614	0.001591	0.000268	0.013415	0.003797	0.110000
140	-3394.866606	-23.841054	0.001673	0.000284	0.013795	0.003796	0.110000
141	-3394.866615	-23.846454	0.001283	0.000251	0.013698	0.002339	0.110000
142	-3394.866640	-23.862407	0.000854	0.000170	0.015121	0.004168	0.110001
143	-3394.866666	-23.878665	0.000667	0.000172	0.016920	0.004704	0.110000
144	-3394.866689	-23.893145	0.000942	0.000207	0.012732	0.003826	0.110000
145	-3394.866714	-23.908733	0.000883	0.000199	0.013926	0.004131	0.110000
146	-3394.866737	-23.923008	0.000951	0.000212	0.013019	0.003864	0.110000
147	-3394.866762	-23.938671	0.000880	0.000227	0.012830	0.003973	0.110000
148	-3394.866785	-23.953366	0.000988	0.000241	0.012926	0.003966	0.110000
149	-3394.866808	-23.968022	0.001506	0.000271	0.013384	0.004038	0.110000
150	-3394.866831	-23.982030	0.002288	0.000332	0.013747	0.004076	0.110000
151	-3394.866839	-23.987258	0.001879	0.000300	0.009459	0.002093	0.110000
152	-3394.866864	-24.002746	0.000717	0.000181	0.014559	0.003947	0.110001
153	-3394.866884	-24.015272	0.000970	0.000246	0.014822	0.004276	0.110000
154	-3394.866907	-24.029765	0.000787	0.000180	0.010542	0.003849	0.110000
155	-3394.866933	-24.045896	0.000748	0.000169	0.013172	0.004323	0.110000
156	-3394.866955	-24.060117	0.001081	0.000228	0.016538	0.004420	0.110000
157	-3394.866980	-24.075493	0.000946	0.000230	0.015692	0.004307	0.110000
158	-3394.867004	-24.090597	0.000905	0.000229	0.014536	0.004222	0.110000
159	-3394.867027	-24.105272	0.000877	0.000228	0.014121	0.004197	0.110000
160	-3394.867050	-24.119824	0.000975	0.000234	0.013904	0.004183	0.110000
161	-3394.867074	-24.134373	0.001019	0.000238	0.013888	0.004161	0.110000
162	-3394.867097	-24.148984	0.001012	0.000238	0.013976	0.004156	0.110000
163	-3394.867120	-24.163604	0.000988	0.000234	0.014060	0.004169	0.110000
164	-3394.867143	-24.178077	0.000966	0.000233	0.013999	0.004180	0.110000
165	-3394.867166	-24.192346	0.000932	0.000235	0.013693	0.004165	0.110000
166	-3394.867186	-24.205022	0.000921	0.000236	0.013206	0.004115	0.110000
167	-3394.867211	-24.220729	0.000939	0.000230	0.013047	0.004099	0.110000
168	-3394.867232	-24.233909	0.000920	0.000222	0.013107	0.004133	0.110000
169	-3394.867256	-24.248805	0.000887	0.000214	0.013645	0.004183	0.110000
170	-3394.867277	-24.261889	0.000845	0.000205	0.013750	0.004227	0.110000
171	-3394.867299	-24.275723	0.000811	0.000198	0.014420	0.004270	0.110000
172	-3394.867321	-24.289538	0.000790	0.000195	0.014827	0.004310	0.110000
173	-3394.867343	-24.303167	0.000801	0.000196	0.014975	0.004328	0.110000
174	-3394.867362	-24.315478	0.000846	0.000203	0.014993	0.004329	0.110000
175	-3394.867387	-24.330772	0.000912	0.000214	0.015215	0.004320	0.110000
176	-3394.867405	-24.342379	0.000943	0.000224	0.014302	0.004279	0.110000
177	-3394.867427	-24.356391	0.000976	0.000233	0.014196	0.004237	0.110000

178	-3394.867447	-24.368839	0.000956	0.000234	0.013537	0.004202	0.110000
179	-3394.867468	-24.381551	0.000893	0.000235	0.013122	0.004190	0.110000
180	-3394.867488	-24.394492	0.000859	0.000230	0.012214	0.004169	0.110000
181	-3394.867509	-24.407640	0.001039	0.000221	0.011432	0.004163	0.110000
182	-3394.867530	-24.420530	0.001150	0.000211	0.011110	0.004173	0.110000
183	-3394.867550	-24.433230	0.001185	0.000202	0.011077	0.004172	0.110000
184	-3394.867570	-24.445903	0.001148	0.000195	0.011118	0.004158	0.110000
185	-3394.867590	-24.458441	0.001052	0.000189	0.011291	0.004146	0.110000
186	-3394.867610	-24.470820	0.000940	0.000185	0.011965	0.004144	0.110000
187	-3394.867629	-24.483147	0.000859	0.000181	0.012707	0.004163	0.110000
188	-3394.867649	-24.495531	0.000869	0.000179	0.013402	0.004215	0.110000
189	-3394.867669	-24.507890	0.001002	0.000182	0.013725	0.004269	0.110000
190	-3394.867688	-24.519892	0.001191	0.000196	0.013222	0.004286	0.110000
191	-3394.867706	-24.531445	0.001305	0.000218	0.011979	0.004242	0.110000
192	-3394.867723	-24.541981	0.001267	0.000235	0.011354	0.004161	0.110000
193	-3394.867742	-24.553817	0.001107	0.000246	0.011436	0.004122	0.110000
194	-3394.867759	-24.564780	0.000923	0.000247	0.013068	0.004083	0.110000
195	-3394.867777	-24.575833	0.000990	0.000245	0.014089	0.004138	0.110000
196	-3394.867794	-24.586741	0.001015	0.000240	0.014709	0.004166	0.110000
197	-3394.867812	-24.597461	0.001003	0.000234	0.014857	0.004198	0.110000
198	-3394.867828	-24.607767	0.001028	0.000235	0.014782	0.004227	0.110000
199	-3394.867845	-24.618549	0.000844	0.000199	0.013410	0.004119	0.110000
200	-3394.867860	-24.628096	0.000840	0.000200	0.012751	0.004220	0.110000
201	-3394.867876	-24.637860	0.000961	0.000184	0.013622	0.004215	0.110000
202	-3394.867889	-24.645846	0.001540	0.000247	0.016670	0.004299	0.110000
203	-3394.867893	-24.648581	0.001349	0.000237	0.015980	0.002644	0.110000
204	-3394.867908	-24.657826	0.001155	0.000188	0.013203	0.004018	0.110000
205	-3394.867924	-24.667989	0.000536	0.000127	0.018844	0.005119	0.110000
206	-3394.867934	-24.674571	0.000595	0.000165	0.009592	0.003610	0.110000
207	-3394.867948	-24.682850	0.001120	0.000226	0.012784	0.004542	0.110000
208	-3394.867959	-24.690060	0.001138	0.000234	0.015986	0.002386	0.110000
209	-3394.867967	-24.694811	0.000903	0.000212	0.012361	0.003789	0.110001
210	-3394.867980	-24.702957	0.000741	0.000143	0.014011	0.004277	0.110000
211	-3394.867995	-24.712796	0.000340	0.000085	0.011296	0.004184	0.110000
212	-3394.868011	-24.722523	0.000494	0.000092	0.013227	0.004517	0.110000
213	-3394.868024	-24.730500	0.000426	0.000092	0.012319	0.004546	0.110000
214	-3394.868039	-24.740158	0.000333	0.000083	0.012945	0.004624	0.110000
215	-3394.868054	-24.749894	0.000264	0.000073	0.014244	0.004729	0.110000
216	-3394.868070	-24.759367	0.000239	0.000068	0.014944	0.004802	0.110000
217	-3394.868084	-24.768433	0.000280	0.000069	0.015086	0.004821	0.110000
218	-3394.868108	-24.783457	0.000344	0.000076	0.014857	0.004795	0.110000
219	-3394.868104	-24.780956	0.000377	0.000080	0.006683	0.001727	0.110000
220	-3394.868115	-24.787652	0.001126	0.000219	0.013833	0.004849	0.110000
221	-3394.868129	-24.796348	0.000357	0.000122	0.011377	0.003896	0.110000
222	-3394.868146	-24.807077	0.000405	0.000092	0.012511	0.004613	0.110000
223	-3394.868153	-24.811856	0.000337	0.000082	0.011701	0.004071	0.110000
224	-3394.868165	-24.819445	0.000415	0.000107	0.012248	0.004602	0.110000
225	-3394.868177	-24.826477	0.000496	0.000134	0.012223	0.004487	0.110000
226	-3394.868187	-24.832740	0.000470	0.000139	0.012269	0.004422	0.110000
227	-3394.868198	-24.839652	0.000421	0.000136	0.012923	0.004381	0.110000
228	-3394.868207	-24.845891	0.000434	0.000131	0.012638	0.004409	0.110000
229	-3394.868218	-24.852367	0.000427	0.000130	0.012531	0.004413	0.110000
230	-3394.868229	-24.859390	0.000443	0.000132	0.012212	0.004428	0.110000
231	-3394.868240	-24.866116	0.000502	0.000135	0.011673	0.004425	0.110000
232	-3394.868250	-24.872536	0.000509	0.000128	0.011478	0.004384	0.110000
233	-3394.868260	-24.878632	0.000481	0.000118	0.011417	0.004349	0.110000
234	-3394.868269	-24.884280	0.000472	0.000117	0.011246	0.004347	0.110000
235	-3394.868278	-24.889870	0.000496	0.000125	0.011366	0.004361	0.110000
236	-3394.868286	-24.895385	0.000517	0.000133	0.010956	0.004364	0.110000
237	-3394.868295	-24.900818	0.000530	0.000134	0.011109	0.004327	0.110000
238	-3394.868304	-24.906371	0.000528	0.000130	0.010829	0.004315	0.110000
239	-3394.868313	-24.911831	0.000499	0.000118	0.010798	0.004281	0.110000
240	-3394.868321	-24.916923	0.000498	0.000114	0.010621	0.004272	0.110000

```
************************************************************
*  MAXIMUM NUMBER OF ITERATIONS REACHED - STOPPING IRC RUN  *
************************************************************


************************************************************
*                        BACKWARD IRC                      *
************************************************************
```

Iteration	E(Eh)	dE(kcal/mol)	max(|G|)	RMS(G)	max(|S|)	RMS(S)	MW-Displ.
Convergence thresholds		0.003138	0.000080	0.000010	0.004000	0.002000	
0	-3394.828654	-0.025565	0.002243	0.000500	0.035537	0.003849	0.046693
1	-3394.829276	-0.415822	0.008043	0.001955	0.087437	0.009316	0.110005
2	-3394.830840	-1.397198	0.015375	0.003532	0.090847	0.009567	0.110004
3	-3394.833115	-2.824807	0.020704	0.004607	0.090242	0.009528	0.110008
4	-3394.835479	-4.308759	0.024789	0.005120	0.084353	0.009116	0.110000
5	-3394.837501	-5.577392	0.027362	0.005239	0.068507	0.008071	0.110002
6	-3394.839139	-6.605464	0.027640	0.005115	0.041265	0.006524	0.110005
7	-3394.840569	-7.502422	0.026484	0.004883	0.026372	0.005569	0.110001
8	-3394.841861	-8.313498	0.024811	0.004637	0.022491	0.005046	0.110001
9	-3394.843048	-9.057912	0.023639	0.004409	0.020293	0.004692	0.110001
10	-3394.844150	-9.749440	0.023695	0.004204	0.020259	0.004449	0.110002
11	-3394.845186	-10.399742	0.023634	0.004021	0.020027	0.004295	0.110000
12	-3394.846160	-11.010937	0.023476	0.003856	0.019795	0.004209	0.110002
13	-3394.847090	-11.594619	0.023233	0.003707	0.020668	0.004167	0.110002
14	-3394.847971	-12.147603	0.022925	0.003569	0.021159	0.004155	0.110001
15	-3394.848829	-12.685456	0.022563	0.003442	0.021370	0.004160	0.110001
16	-3394.849643	-13.196843	0.022162	0.003322	0.021398	0.004174	0.110002
17	-3394.850427	-13.688489	0.021735	0.003207	0.021302	0.004193	0.110002
18	-3394.851181	-14.161799	0.021287	0.003098	0.021123	0.004212	0.110003
19	-3394.851908	-14.617874	0.020827	0.002994	0.020890	0.004233	0.110000
20	-3394.852607	-15.056693	0.020357	0.002894	0.020622	0.004253	0.110002
21	-3394.853281	-15.479325	0.019882	0.002799	0.020337	0.004271	0.110000
22	-3394.853930	-15.886429	0.019405	0.002708	0.020045	0.004288	0.110001
23	-3394.854553	-16.277368	0.018934	0.002623	0.019743	0.004303	0.110003
24	-3394.855151	-16.652619	0.018474	0.002550	0.019403	0.004314	0.110003
25	-3394.855716	-17.007212	0.018040	0.002502	0.018921	0.004299	0.110000
26	-3394.856249	-17.342042	0.017613	0.002454	0.018201	0.004195	0.110001
27	-3394.856759	-17.661683	0.017171	0.002378	0.017611	0.004113	0.110002
28	-3394.857250	-17.969801	0.016726	0.002306	0.017315	0.004114	0.110001
29	-3394.857721	-18.265365	0.016288	0.002240	0.016977	0.004103	0.110001
30	-3394.858173	-18.549011	0.015855	0.002176	0.017082	0.004082	0.110001
31	-3394.858606	-18.820644	0.015427	0.002112	0.017171	0.004062	0.110001
32	-3394.859021	-19.081527	0.015003	0.002051	0.017266	0.004042	0.110001
33	-3394.859421	-19.332167	0.014575	0.001989	0.017359	0.004025	0.110000
34	-3394.859803	-19.572251	0.014166	0.001931	0.017425	0.004009	0.110001
35	-3394.860171	-19.802703	0.013776	0.001875	0.017476	0.003989	0.110001
36	-3394.860523	-20.023779	0.013395	0.001822	0.017510	0.003971	0.110002
37	-3394.860861	-20.235989	0.013021	0.001769	0.017530	0.003950	0.110000
38	-3394.861185	-20.439458	0.012654	0.001719	0.017535	0.003929	0.110000
39	-3394.861497	-20.634781	0.012293	0.001669	0.017523	0.003906	0.110000
40	-3394.861795	-20.822088	0.011942	0.001622	0.017531	0.003888	0.110002
41	-3394.862082	-21.001971	0.011599	0.001575	0.017522	0.003868	0.110002
42	-3394.862357	-21.174405	0.011265	0.001532	0.017487	0.003849	0.110001
43	-3394.862621	-21.340163	0.010939	0.001488	0.017446	0.003827	0.110001
44	-3394.862874	-21.498876	0.010620	0.001449	0.017390	0.003808	0.110001
45	-3394.863117	-21.651806	0.010309	0.001405	0.017330	0.003784	0.110000
46	-3394.863350	-21.797690	0.010006	0.001374	0.017251	0.003766	0.110000
47	-3394.863575	-21.939190	0.009709	0.001326	0.017177	0.003739	0.110000
48	-3394.863788	-22.072720	0.009421	0.001308	0.017069	0.003724	0.110000
49	-3394.863999	-22.204855	0.009138	0.001243	0.016991	0.003693	0.110001
50	-3394.864191	-22.325554	0.008862	0.001261	0.016838	0.003691	0.110001

51	-3394.864393	-22.452030	0.008590	0.001143	0.016714	0.003651	0.110001
52	-3394.864569	-22.562615	0.008310	0.001200	0.016659	0.003717	0.110000
53	-3394.864760	-22.682509	0.008054	0.001061	0.016651	0.003648	0.110001
54	-3394.864930	-22.788991	0.007774	0.001077	0.016736	0.003763	0.110001
55	-3394.865095	-22.892998	0.007547	0.001080	0.016950	0.003682	0.110001
56	-3394.865254	-22.992391	0.007321	0.001043	0.016127	0.003576	0.110001
57	-3394.865402	-23.085127	0.007091	0.001045	0.016130	0.003548	0.110001
58	-3394.865551	-23.178752	0.006870	0.000980	0.015827	0.003508	0.110000
59	-3394.865683	-23.261994	0.006647	0.001023	0.015611	0.003499	0.110001
60	-3394.865829	-23.353405	0.006442	0.000893	0.015508	0.003478	0.110001
61	-3394.865953	-23.430959	0.006208	0.000962	0.015386	0.003540	0.110000
62	-3394.866088	-23.515715	0.006024	0.000856	0.015598	0.003497	0.110000
63	-3394.866200	-23.586386	0.005813	0.000940	0.014997	0.003496	0.110001
64	-3394.866329	-23.667024	0.005640	0.000804	0.015071	0.003452	0.110001
65	-3394.866435	-23.733756	0.005426	0.000860	0.014835	0.003509	0.110001
66	-3394.866550	-23.805859	0.005268	0.000823	0.015217	0.003465	0.110000
67	-3394.866647	-23.866504	0.005098	0.000866	0.014123	0.003383	0.110000
68	-3394.866758	-23.936157	0.004942	0.000770	0.014325	0.003365	0.110000
69	-3394.866848	-23.993062	0.004758	0.000842	0.013886	0.003373	0.110000
70	-3394.866878	-24.011700	0.005233	0.000913	0.020412	0.002396	0.110000
71	-3394.866984	-24.078021	0.004556	0.000547	0.007726	0.002932	0.110001
72	-3394.867092	-24.145824	0.004335	0.000524	0.015983	0.003937	0.110001
73	-3394.867193	-24.209116	0.004147	0.000515	0.016179	0.003873	0.110000
74	-3394.867287	-24.268276	0.003981	0.000504	0.015369	0.003796	0.110001
75	-3394.867379	-24.326060	0.003825	0.000486	0.015144	0.003792	0.110000
76	-3394.867468	-24.381743	0.003672	0.000468	0.015244	0.003806	0.110000
77	-3394.867553	-24.435421	0.003522	0.000452	0.015241	0.003813	0.110000
78	-3394.867635	-24.486684	0.003377	0.000440	0.015080	0.003800	0.110001
79	-3394.867713	-24.535730	0.003238	0.000430	0.014790	0.003772	0.110000
80	-3394.867788	-24.582728	0.003103	0.000418	0.014498	0.003746	0.110000
81	-3394.867860	-24.627823	0.002971	0.000407	0.014314	0.003732	0.110000
82	-3394.867929	-24.671040	0.002843	0.000396	0.014191	0.003725	0.110000
83	-3394.867995	-24.712343	0.002717	0.000385	0.014088	0.003717	0.110000
84	-3394.868058	-24.752193	0.002601	0.000376	0.013863	0.003703	0.110001
85	-3394.868119	-24.790503	0.002491	0.000367	0.013598	0.003694	0.110000
86	-3394.868178	-24.827421	0.002385	0.000358	0.013328	0.003686	0.110000
87	-3394.868235	-24.863010	0.002284	0.000350	0.013050	0.003680	0.110000
88	-3394.868289	-24.897306	0.002187	0.000343	0.012772	0.003674	0.110000
89	-3394.868342	-24.930422	0.002094	0.000336	0.012494	0.003670	0.110000
90	-3394.868393	-24.962347	0.002005	0.000329	0.012213	0.003666	0.110000
91	-3394.868442	-24.993165	0.001919	0.000322	0.011936	0.003663	0.110000
92	-3394.868491	-25.023881	0.001838	0.000316	0.011662	0.003662	0.110000
93	-3394.868538	-25.053191	0.001758	0.000312	0.011334	0.003662	0.110000
94	-3394.868579	-25.079260	0.001682	0.000306	0.011015	0.003657	0.110000
95	-3394.868623	-25.106372	0.001610	0.000298	0.010905	0.003649	0.110000
96	-3394.868664	-25.132322	0.001539	0.000294	0.010594	0.003666	0.110000
97	-3394.868707	-25.159288	0.001529	0.000289	0.010348	0.003660	0.110000
98	-3394.868746	-25.183859	0.001546	0.000287	0.009910	0.003675	0.110000
99	-3394.868784	-25.207563	0.001527	0.000281	0.009741	0.003657	0.110000
100	-3394.868820	-25.230513	0.001524	0.000277	0.009880	0.003677	0.110000
101	-3394.868856	-25.252644	0.001525	0.000274	0.009989	0.003661	0.110000
102	-3394.868889	-25.273368	0.001428	0.000261	0.010115	0.003715	0.110000
103	-3394.868921	-25.293547	0.001463	0.000268	0.011528	0.003607	0.110000
104	-3394.868955	-25.314828	0.001170	0.000236	0.010287	0.003749	0.110000
105	-3394.868984	-25.333131	0.001219	0.000224	0.014424	0.003773	0.110000
106	-3394.869014	-25.352126	0.001447	0.000251	0.012249	0.003838	0.110000
107	-3394.869044	-25.370631	0.001362	0.000235	0.010849	0.003644	0.110000
108	-3394.869069	-25.386724	0.001439	0.000244	0.011177	0.003708	0.110000
109	-3394.869098	-25.404428	0.001386	0.000234	0.011191	0.003642	0.110000
110	-3394.869123	-25.420193	0.001457	0.000242	0.011635	0.003729	0.110000
111	-3394.869148	-25.436274	0.001338	0.000226	0.011323	0.003660	0.110000
112	-3394.869173	-25.451676	0.001407	0.000233	0.012521	0.003793	0.110000
113	-3394.869198	-25.467148	0.001300	0.000218	0.011539	0.003699	0.110000

114	-3394.869220	-25.481448	0.001364	0.000225	0.012623	0.003806	0.110000
115	-3394.869243	-25.495831	0.001275	0.000211	0.011780	0.003712	0.110000
116	-3394.869265	-25.509269	0.001335	0.000218	0.012269	0.003804	0.110000
117	-3394.869286	-25.522842	0.001261	0.000207	0.011968	0.003725	0.110000
118	-3394.869306	-25.535376	0.001304	0.000213	0.012524	0.003818	0.110000
119	-3394.869327	-25.548412	0.001207	0.000198	0.012087	0.003734	0.110000
120	-3394.869346	-25.560158	0.001276	0.000208	0.013091	0.003874	0.110000
121	-3394.869365	-25.572554	0.001109	0.000184	0.012058	0.003721	0.110000
122	-3394.869382	-25.583215	0.001225	0.000199	0.013678	0.003968	0.110000
123	-3394.869402	-25.595492	0.001084	0.000178	0.012265	0.003756	0.110000
124	-3394.869418	-25.605790	0.001165	0.000191	0.013204	0.003963	0.110000
125	-3394.869436	-25.616629	0.001136	0.000184	0.012696	0.003806	0.110000
126	-3394.869452	-25.626652	0.001097	0.000184	0.012870	0.003870	0.110000
127	-3394.869467	-25.636240	0.001082	0.000179	0.012221	0.003772	0.110000
128	-3394.869481	-25.645111	0.000956	0.000165	0.012085	0.003721	0.110000
129	-3394.869495	-25.654032	0.001034	0.000175	0.012604	0.003851	0.110000
130	-3394.869509	-25.662340	0.000891	0.000157	0.012251	0.003704	0.110000
131	-3394.869511	-25.664030	0.000580	0.000124	0.007748	0.001803	0.110000
132	-3394.869521	-25.670002	0.000705	0.000151	0.016978	0.004511	0.110001
133	-3394.869536	-25.679664	0.000530	0.000132	0.016801	0.004101	0.110000
134	-3394.869545	-25.685163	0.000915	0.000152	0.020587	0.004484	0.110000
135	-3394.869558	-25.693043	0.001465	0.000216	0.013675	0.003996	0.110000
136	-3394.869566	-25.698477	0.001703	0.000231	0.011176	0.003738	0.110000
137	-3394.869577	-25.705433	0.001516	0.000210	0.011847	0.003751	0.110000
138	-3394.869585	-25.710561	0.001571	0.000222	0.010346	0.003568	0.110000
139	-3394.869595	-25.716502	0.001372	0.000195	0.010557	0.003564	0.110000
140	-3394.869596	-25.717493	0.001348	0.000187	0.009957	0.001920	0.110000
141	-3394.869606	-25.723603	0.000729	0.000144	0.011869	0.003623	0.110000
142	-3394.869619	-25.731438	0.001676	0.000229	0.009647	0.003646	0.110000
143	-3394.869614	-25.728279	0.001061	0.000146	0.011292	0.001947	0.110000
144	-3394.869617	-25.730424	0.000920	0.000141	0.010586	0.003861	0.110000
145	-3394.869626	-25.735819	0.000180	0.000051	0.011174	0.004229	0.110000
146	-3394.869633	-25.740456	0.000298	0.000069	0.011831	0.004392	0.110000
147	-3394.869640	-25.744842	0.000327	0.000079	0.012718	0.004152	0.110000
148	-3394.869646	-25.748419	0.000391	0.000077	0.011714	0.004061	0.110000
149	-3394.869652	-25.752170	0.000562	0.000079	0.011427	0.004207	0.110000
150	-3394.869654	-25.753857	0.000482	0.000082	0.011533	0.003916	0.107392
151	-3394.869657	-25.755466	0.000261	0.000067	0.009185	0.003100	0.086979
152	-3394.869660	-25.757069	0.000228	0.000056	0.008661	0.002866	0.078936
153	-3394.869662	-25.758639	0.000224	0.000057	0.009618	0.003157	0.085286
154	-3394.869669	-25.762762	0.000287	0.000059	0.010584	0.003435	0.092744
155	-3394.869669	-25.762962	0.000317	0.000072	0.011084	0.004208	0.110000
156	-3394.869674	-25.766459	0.000193	0.000049	0.009628	0.003163	0.081377
157	-3394.869677	-25.768165	0.000138	0.000040	0.010009	0.003727	0.110000
158	-3394.869680	-25.770134	0.000404	0.000064	0.012815	0.004150	0.110000
159	-3394.869683	-25.771615	0.000400	0.000070	0.010224	0.003585	0.099327
160	-3394.869685	-25.773044	0.000304	0.000060	0.007440	0.002724	0.073578
161	-3394.869690	-25.775982	0.000188	0.000045	0.007159	0.002544	0.067234
162	-3394.869685	-25.773348	0.000214	0.000046	0.003360	0.000742	0.084188
163	-3394.869685	-25.773136	0.000770	0.000166	0.014204	0.003632	0.110000
164	-3394.869688	-25.774624	0.000260	0.000063	0.006559	0.001356	0.035567
165	-3394.869689	-25.775523	0.000298	0.000085	0.003938	0.001582	0.047574
166	-3394.869691	-25.776743	0.000436	0.000110	0.010286	0.003487	0.110000
167	-3394.869693	-25.777837	0.000417	0.000098	0.006728	0.002231	0.068433
168	-3394.869695	-25.779079	0.000462	0.000123	0.006420	0.002256	0.070199
169	-3394.869694	-25.778667	0.000497	0.000127	0.005107	0.002224	0.070373
170	-3394.869695	-25.779131	0.000536	0.000126	0.007104	0.001850	0.055872
171	-3394.869694	-25.778846	0.000967	0.000220	0.008454	0.003209	0.098240
172	-3394.869695	-25.779473	0.000822	0.000201	0.012453	0.002821	0.078222
173	-3394.869698	-25.781231	0.000516	0.000159	0.009501	0.001882	0.047802
174	-3394.869699	-25.781812	0.000287	0.000071	0.004312	0.001223	0.031949
175	-3394.869699	-25.781821	0.000139	0.000027	0.006846	0.002194	0.051633
176	-3394.869701	-25.783179	0.000119	0.000030	0.007570	0.002413	0.058916

177	-3394.869704	-25.784723	0.000217	0.000060	0.011721	0.004043	0.110000
178	-3394.869707	-25.786921	0.000304	0.000057	0.009832	0.003054	0.075150
179	-3394.869706	-25.786454	0.000178	0.000041	0.004670	0.001795	0.064496
180	-3394.869706	-25.786323	0.000584	0.000094	0.010074	0.003532	0.105146
181	-3394.869716	-25.792301	0.000724	0.000113	0.013023	0.004224	0.110000
182	-3394.869711	-25.789089	0.000480	0.000081	0.004138	0.000909	0.059797
183	-3394.869711	-25.789418	0.000362	0.000093	0.009444	0.003474	0.110000
184	-3394.869710	-25.788950	0.000343	0.000079	0.006922	0.001845	0.061269
185	-3394.869713	-25.790556	0.000366	0.000073	0.002853	0.000928	0.028151
186	-3394.869714	-25.791366	0.000602	0.000113	0.004069	0.001737	0.055658
187	-3394.869712	-25.789882	0.000500	0.000096	0.007131	0.002402	0.078923
188	-3394.869715	-25.791666	0.000149	0.000030	0.007207	0.001804	0.050290
189	-3394.869717	-25.793321	0.000432	0.000106	0.014714	0.004761	0.110000
190	-3394.869716	-25.792195	0.000339	0.000083	0.005763	0.001087	0.062123
191	-3394.869717	-25.792923	0.000462	0.000125	0.010867	0.003848	0.110000
192	-3394.869721	-25.795666	0.000117	0.000024	0.003845	0.000861	0.023002
193	-3394.869719	-25.794451	0.000508	0.000120	0.009287	0.003691	0.110000
194	-3394.869719	-25.794596	0.000098	0.000026	0.004259	0.001349	0.034851
195	-3394.869724	-25.797262	0.000239	0.000063	0.013923	0.004258	0.110000
196	-3394.869722	-25.796334	0.000278	0.000088	0.009958	0.003492	0.107995
197	-3394.869724	-25.797234	0.000168	0.000052	0.003258	0.001158	0.038073
198	-3394.869720	-25.795243	0.000455	0.000107	0.006511	0.001998	0.058468
199	-3394.869724	-25.797380	0.000227	0.000048	0.006789	0.001526	0.040365
200	-3394.869724	-25.797476	0.000410	0.000084	0.011784	0.003416	0.110000
201	-3394.869724	-25.797797	0.000111	0.000025	0.002616	0.000354	0.021871
202	-3394.869725	-25.798390	0.000205	0.000057	0.004636	0.001362	0.041721
203	-3394.869725	-25.798421	0.000225	0.000063	0.005249	0.002050	0.066196
204	-3394.869726	-25.798938	0.000080	0.000027	0.002555	0.000779	0.063624
205	-3394.869726	-25.798975	0.000244	0.000065	0.009343	0.003546	0.110000
206	-3394.869730	-25.801082	0.000085	0.000023	0.003253	0.000855	0.023701
207	-3394.869728	-25.800109	0.000134	0.000034	0.002874	0.001043	0.110000
208	-3394.869729	-25.800477	0.000210	0.000055	0.007860	0.002810	0.090323
209	-3394.869728	-25.800017	0.000143	0.000030	0.001863	0.000654	0.054541
210	-3394.869730	-25.801094	0.000148	0.000033	0.012397	0.004175	0.110000
211	-3394.869731	-25.802058	0.000118	0.000023	0.005588	0.001780	0.048348
212	-3394.869732	-25.802752	0.000121	0.000029	0.008892	0.002874	0.083760
213	-3394.869732	-25.802470	0.000197	0.000038	0.006509	0.002283	0.077185
214	-3394.869735	-25.804576	0.000118	0.000025	0.006940	0.002491	0.060278
215	-3394.869734	-25.803965	0.000154	0.000035	0.010820	0.003776	0.110000
216	-3394.869737	-25.805522	0.000128	0.000031	0.006969	0.002481	0.057762
217	-3394.869737	-25.805927	0.000122	0.000024	0.008291	0.002772	0.080926
218	-3394.869739	-25.807052	0.000116	0.000027	0.011315	0.003600	0.086999
219	-3394.869740	-25.807737	0.000109	0.000025	0.009929	0.003368	0.094377
220	-3394.869742	-25.808850	0.000103	0.000023	0.010641	0.003279	0.079206
221	-3394.869744	-25.810197	0.000117	0.000030	0.009685	0.003594	0.110000
222	-3394.869744	-25.810274	0.000155	0.000029	0.009812	0.003132	0.075674
223	-3394.869741	-25.808455	0.000205	0.000042	0.008886	0.003270	0.099639
224	-3394.869747	-25.811699	0.000076	0.000020	0.007185	0.002291	0.058826
225	-3394.869748	-25.812285	0.000106	0.000022	0.014170	0.004446	0.110000
226	-3394.869750	-25.813644	0.000099	0.000022	0.008724	0.002908	0.074628
227	-3394.869751	-25.814690	0.000095	0.000021	0.007476	0.002639	0.071186
228	-3394.869754	-25.816118	0.000115	0.000022	0.007525	0.002728	0.082649
229	-3394.869755	-25.816676	0.000124	0.000028	0.009074	0.003320	0.103121
230	-3394.869756	-25.817301	0.000154	0.000026	0.007019	0.002589	0.080105
231	-3394.869759	-25.819702	0.000067	0.000018	0.009779	0.003723	0.101544
232	-3394.869761	-25.820497	0.000131	0.000026	0.002162	0.000840	0.098031
233	-3394.869757	-25.818470	0.000285	0.000048	0.008424	0.003382	0.110000
234	-3394.869761	-25.820617	0.000078	0.000018	0.005277	0.001101	0.031400
235	-3394.869766	-25.823894	0.000107	0.000026	0.012474	0.004406	0.110000
236	-3394.869765	-25.822996	0.000058	0.000018	0.007007	0.002888	0.092108
237	-3394.869767	-25.824455	0.000171	0.000033	0.012453	0.004356	0.110000
238	-3394.869769	-25.825809	0.000148	0.000027	0.006186	0.001938	0.107874
239	-3394.869771	-25.826776	0.000141	0.000031	0.008737	0.003708	0.110000

240 -3394.869773 -25.828210 0.000090 0.000024 0.008205 0.002733 0.070674

```
************************************************************
*  MAXIMUM NUMBER OF ITERATIONS REACHED - STOPPING IRC RUN  *
************************************************************
```

```
---------------------------------------------------------------
                      IRC PATH SUMMARY
---------------------------------------------------------------
```

All gradients are in Eh/Bohr.

Step	E(Eh)	dE(kcal/mol)	max(|G|)	RMS(G)	SMW-Displ.
1	-3394.869773	-25.828210	0.000090	0.000024	-23.660653
2	-3394.869771	-25.826776	0.000141	0.000031	-23.589979
3	-3394.869769	-25.825809	0.000148	0.000027	-23.479979
4	-3394.869767	-25.824455	0.000171	0.000033	-23.372105
5	-3394.869765	-25.822996	0.000058	0.000018	-23.262105
6	-3394.869766	-25.823894	0.000107	0.000026	-23.169997
7	-3394.869761	-25.820617	0.000078	0.000018	-23.059996
8	-3394.869757	-25.818470	0.000285	0.000048	-23.028596
9	-3394.869761	-25.820497	0.000131	0.000026	-22.918596
10	-3394.869759	-25.819702	0.000067	0.000018	-22.820565
11	-3394.869756	-25.817301	0.000154	0.000026	-22.719021
12	-3394.869755	-25.816676	0.000124	0.000028	-22.638916
13	-3394.869754	-25.816118	0.000115	0.000022	-22.535795
14	-3394.869751	-25.814690	0.000095	0.000021	-22.453146
15	-3394.869750	-25.813644	0.000099	0.000022	-22.381961
16	-3394.869748	-25.812285	0.000106	0.000022	-22.307332
17	-3394.869747	-25.811699	0.000076	0.000020	-22.197332
18	-3394.869741	-25.808455	0.000205	0.000042	-22.138507
19	-3394.869744	-25.810274	0.000155	0.000029	-22.038867
20	-3394.869744	-25.810197	0.000117	0.000030	-21.963194
21	-3394.869742	-25.808850	0.000103	0.000023	-21.853193
22	-3394.869740	-25.807737	0.000109	0.000025	-21.773988
23	-3394.869739	-25.807052	0.000116	0.000027	-21.679610
24	-3394.869737	-25.805927	0.000122	0.000024	-21.592612
25	-3394.869737	-25.805522	0.000128	0.000031	-21.511685
26	-3394.869734	-25.803965	0.000154	0.000035	-21.453924
27	-3394.869735	-25.804576	0.000118	0.000025	-21.343924
28	-3394.869732	-25.802470	0.000197	0.000038	-21.283646
29	-3394.869732	-25.802752	0.000121	0.000029	-21.206460
30	-3394.869731	-25.802058	0.000118	0.000023	-21.122700
31	-3394.869730	-25.801094	0.000148	0.000033	-21.074352
32	-3394.869728	-25.800017	0.000143	0.000030	-20.964352
33	-3394.869729	-25.800477	0.000210	0.000055	-20.909811
34	-3394.869728	-25.800109	0.000134	0.000034	-20.819488
35	-3394.869730	-25.801082	0.000085	0.000023	-20.709488
36	-3394.869726	-25.798975	0.000244	0.000065	-20.685787
37	-3394.869726	-25.798938	0.000080	0.000027	-20.575787
38	-3394.869725	-25.798421	0.000225	0.000063	-20.512162
39	-3394.869725	-25.798390	0.000205	0.000057	-20.445967
40	-3394.869724	-25.797797	0.000111	0.000025	-20.404246
41	-3394.869724	-25.797476	0.000410	0.000084	-20.382375
42	-3394.869724	-25.797380	0.000227	0.000048	-20.272375
43	-3394.869720	-25.795243	0.000455	0.000107	-20.232010
44	-3394.869724	-25.797234	0.000168	0.000052	-20.173543
45	-3394.869722	-25.796334	0.000278	0.000088	-20.135469
46	-3394.869724	-25.797262	0.000239	0.000063	-20.027475
47	-3394.869719	-25.794596	0.000098	0.000026	-19.917474
48	-3394.869719	-25.794451	0.000508	0.000120	-19.882624
49	-3394.869721	-25.795666	0.000117	0.000024	-19.772624
50	-3394.869717	-25.792923	0.000462	0.000125	-19.749622

51	-3394.869716	-25.792195	0.000339	0.000083	-19.639622
52	-3394.869717	-25.793321	0.000432	0.000106	-19.577498
53	-3394.869715	-25.791666	0.000149	0.000030	-19.467498
54	-3394.869712	-25.789882	0.000500	0.000096	-19.417208
55	-3394.869714	-25.791366	0.000602	0.000113	-19.338285
56	-3394.869713	-25.790556	0.000366	0.000073	-19.282628
57	-3394.869710	-25.788950	0.000343	0.000079	-19.254476
58	-3394.869711	-25.789418	0.000362	0.000093	-19.193207
59	-3394.869711	-25.789089	0.000480	0.000081	-19.083207
60	-3394.869716	-25.792301	0.000724	0.000113	-19.023410
61	-3394.869706	-25.786323	0.000584	0.000094	-18.913410
62	-3394.869706	-25.786454	0.000178	0.000041	-18.808263
63	-3394.869707	-25.786921	0.000304	0.000057	-18.743767
64	-3394.869704	-25.784723	0.000217	0.000060	-18.668617
65	-3394.869701	-25.783179	0.000119	0.000030	-18.558617
66	-3394.869699	-25.781821	0.000139	0.000027	-18.499701
67	-3394.869699	-25.781812	0.000287	0.000071	-18.448069
68	-3394.869698	-25.781231	0.000516	0.000159	-18.416120
69	-3394.869695	-25.779473	0.000822	0.000201	-18.368318
70	-3394.869694	-25.778846	0.000967	0.000220	-18.290096
71	-3394.869695	-25.779131	0.000536	0.000126	-18.191856
72	-3394.869694	-25.778667	0.000497	0.000127	-18.135984
73	-3394.869695	-25.779079	0.000462	0.000123	-18.065612
74	-3394.869693	-25.777837	0.000417	0.000098	-17.995413
75	-3394.869691	-25.776743	0.000436	0.000110	-17.926980
76	-3394.869689	-25.775523	0.000298	0.000085	-17.816980
77	-3394.869688	-25.774624	0.000260	0.000063	-17.769406
78	-3394.869685	-25.773136	0.000770	0.000166	-17.733839
79	-3394.869685	-25.773348	0.000214	0.000046	-17.623839
80	-3394.869690	-25.775982	0.000188	0.000045	-17.539651
81	-3394.869685	-25.773044	0.000304	0.000060	-17.472417
82	-3394.869683	-25.771615	0.000400	0.000070	-17.398839
83	-3394.869680	-25.770134	0.000404	0.000064	-17.299511
84	-3394.869677	-25.768165	0.000138	0.000040	-17.189511
85	-3394.869674	-25.766459	0.000193	0.000049	-17.079511
86	-3394.869669	-25.762962	0.000317	0.000072	-16.998135
87	-3394.869669	-25.762762	0.000287	0.000059	-16.888135
88	-3394.869662	-25.758639	0.000224	0.000057	-16.795390
89	-3394.869660	-25.757069	0.000228	0.000056	-16.710104
90	-3394.869657	-25.755466	0.000261	0.000067	-16.631168
91	-3394.869654	-25.753857	0.000482	0.000082	-16.544189
92	-3394.869652	-25.752170	0.000562	0.000079	-16.436797
93	-3394.869646	-25.748419	0.000391	0.000077	-16.326797
94	-3394.869640	-25.744842	0.000327	0.000079	-16.216797
95	-3394.869633	-25.740456	0.000298	0.000069	-16.106797
96	-3394.869626	-25.735819	0.000180	0.000051	-15.996797
97	-3394.869617	-25.730424	0.000920	0.000141	-15.886797
98	-3394.869614	-25.728279	0.001061	0.000146	-15.776796
99	-3394.869619	-25.731438	0.001676	0.000229	-15.666796
100	-3394.869606	-25.723603	0.000729	0.000144	-15.556796
101	-3394.869596	-25.717493	0.001348	0.000187	-15.446796
102	-3394.869595	-25.716502	0.001372	0.000195	-15.336796
103	-3394.869585	-25.710561	0.001571	0.000222	-15.226796
104	-3394.869577	-25.705433	0.001516	0.000210	-15.116796
105	-3394.869566	-25.698477	0.001703	0.000231	-15.006795
106	-3394.869558	-25.693043	0.001465	0.000216	-14.896795
107	-3394.869545	-25.685163	0.000915	0.000152	-14.786795
108	-3394.869536	-25.679664	0.000530	0.000132	-14.676795
109	-3394.869521	-25.670002	0.000705	0.000151	-14.566795
110	-3394.869511	-25.664030	0.000580	0.000124	-14.456794
111	-3394.869509	-25.662340	0.000891	0.000157	-14.346794
112	-3394.869495	-25.654032	0.001034	0.000175	-14.236794
113	-3394.869481	-25.645111	0.000956	0.000165	-14.126794

114	-3394.869467	-25.636240	0.001082	0.000179	-14.016794
115	-3394.869452	-25.626652	0.001097	0.000184	-13.906794
116	-3394.869436	-25.616629	0.001136	0.000184	-13.796794
117	-3394.869418	-25.605790	0.001165	0.000191	-13.686793
118	-3394.869402	-25.595492	0.001084	0.000178	-13.576793
119	-3394.869382	-25.583215	0.001225	0.000199	-13.466793
120	-3394.869365	-25.572554	0.001109	0.000184	-13.356793
121	-3394.869346	-25.560158	0.001276	0.000208	-13.246793
122	-3394.869327	-25.548412	0.001207	0.000198	-13.136793
123	-3394.869306	-25.535376	0.001304	0.000213	-13.026793
124	-3394.869286	-25.522842	0.001261	0.000207	-12.916793
125	-3394.869265	-25.509269	0.001335	0.000218	-12.806793
126	-3394.869243	-25.495831	0.001275	0.000211	-12.696792
127	-3394.869220	-25.481448	0.001364	0.000225	-12.586792
128	-3394.869198	-25.467148	0.001300	0.000218	-12.476792
129	-3394.869173	-25.451676	0.001407	0.000233	-12.366792
130	-3394.869148	-25.436274	0.001338	0.000226	-12.256792
131	-3394.869123	-25.420193	0.001457	0.000242	-12.146792
132	-3394.869098	-25.404428	0.001386	0.000234	-12.036791
133	-3394.869069	-25.386724	0.001439	0.000244	-11.926791
134	-3394.869044	-25.370631	0.001362	0.000235	-11.816791
135	-3394.869014	-25.352126	0.001447	0.000251	-11.706791
136	-3394.868984	-25.333131	0.001219	0.000224	-11.596791
137	-3394.868955	-25.314828	0.001170	0.000236	-11.486791
138	-3394.868921	-25.293547	0.001463	0.000268	-11.376790
139	-3394.868889	-25.273368	0.001428	0.000261	-11.266790
140	-3394.868856	-25.252644	0.001525	0.000274	-11.156790
141	-3394.868820	-25.230513	0.001524	0.000277	-11.046789
142	-3394.868784	-25.207563	0.001527	0.000281	-10.936789
143	-3394.868746	-25.183859	0.001546	0.000287	-10.826789
144	-3394.868707	-25.159288	0.001529	0.000289	-10.716789
145	-3394.868664	-25.132322	0.001539	0.000294	-10.606789
146	-3394.868623	-25.106372	0.001610	0.000298	-10.496789
147	-3394.868579	-25.079260	0.001682	0.000306	-10.386788
148	-3394.868538	-25.053191	0.001758	0.000312	-10.276788
149	-3394.868491	-25.023881	0.001838	0.000316	-10.166788
150	-3394.868442	-24.993165	0.001919	0.000322	-10.056788
151	-3394.868393	-24.962347	0.002005	0.000329	-9.946788
152	-3394.868342	-24.930422	0.002094	0.000336	-9.836788
153	-3394.868289	-24.897306	0.002187	0.000343	-9.726787
154	-3394.868235	-24.863010	0.002284	0.000350	-9.616787
155	-3394.868178	-24.827421	0.002385	0.000358	-9.506787
156	-3394.868119	-24.790503	0.002491	0.000367	-9.396787
157	-3394.868058	-24.752193	0.002601	0.000376	-9.286786
158	-3394.867995	-24.712343	0.002717	0.000385	-9.176785
159	-3394.867929	-24.671040	0.002843	0.000396	-9.066785
160	-3394.867860	-24.627823	0.002971	0.000407	-8.956785
161	-3394.867788	-24.582728	0.003103	0.000418	-8.846785
162	-3394.867713	-24.535730	0.003238	0.000430	-8.736784
163	-3394.867635	-24.486684	0.003377	0.000440	-8.626784
164	-3394.867553	-24.435421	0.003522	0.000452	-8.516784
165	-3394.867468	-24.381743	0.003672	0.000468	-8.406784
166	-3394.867379	-24.326060	0.003825	0.000486	-8.296783
167	-3394.867287	-24.268276	0.003981	0.000504	-8.186783
168	-3394.867193	-24.209116	0.004147	0.000515	-8.076782
169	-3394.867092	-24.145824	0.004335	0.000524	-7.966782
170	-3394.866984	-24.078021	0.004556	0.000547	-7.856781
171	-3394.866878	-24.011700	0.005233	0.000913	-7.746780
172	-3394.866848	-23.993062	0.004758	0.000842	-7.636779
173	-3394.866758	-23.936157	0.004942	0.000770	-7.526779
174	-3394.866647	-23.866504	0.005098	0.000866	-7.416779
175	-3394.866550	-23.805859	0.005268	0.000823	-7.306778
176	-3394.866435	-23.733756	0.005426	0.000860	-7.196778

177	-3394.866329	-23.667024	0.005640	0.000804	-7.086778
178	-3394.866200	-23.586386	0.005813	0.000940	-6.976777
179	-3394.866088	-23.515715	0.006024	0.000856	-6.866776
180	-3394.865953	-23.430959	0.006208	0.000962	-6.756776
181	-3394.865829	-23.353405	0.006442	0.000893	-6.646776
182	-3394.865683	-23.261994	0.006647	0.001023	-6.536775
183	-3394.865551	-23.178752	0.006870	0.000980	-6.426774
184	-3394.865402	-23.085127	0.007091	0.001045	-6.316774
185	-3394.865254	-22.992391	0.007321	0.001043	-6.206773
186	-3394.865095	-22.892998	0.007547	0.001080	-6.096772
187	-3394.864930	-22.788991	0.007774	0.001077	-5.986771
188	-3394.864760	-22.682509	0.008054	0.001061	-5.876771
189	-3394.864569	-22.562615	0.008310	0.001200	-5.766769
190	-3394.864393	-22.452030	0.008590	0.001143	-5.656769
191	-3394.864191	-22.325554	0.008862	0.001261	-5.546768
192	-3394.863999	-22.204855	0.009138	0.001243	-5.436767
193	-3394.863788	-22.072720	0.009421	0.001308	-5.326766
194	-3394.863575	-21.939190	0.009709	0.001326	-5.216766
195	-3394.863350	-21.797690	0.010006	0.001374	-5.106765
196	-3394.863117	-21.651806	0.010309	0.001405	-4.996765
197	-3394.862874	-21.498876	0.010620	0.001449	-4.886765
198	-3394.862621	-21.340163	0.010939	0.001488	-4.776764
199	-3394.862357	-21.174405	0.011265	0.001532	-4.666763
200	-3394.862082	-21.001971	0.011599	0.001575	-4.556762
201	-3394.861795	-20.822088	0.011942	0.001622	-4.446760
202	-3394.861497	-20.634781	0.012293	0.001669	-4.336759
203	-3394.861185	-20.439458	0.012654	0.001719	-4.226758
204	-3394.860861	-20.235989	0.013021	0.001769	-4.116758
205	-3394.860523	-20.023779	0.013395	0.001822	-4.006758
206	-3394.860171	-19.802703	0.013776	0.001875	-3.896756
207	-3394.859803	-19.572251	0.014166	0.001931	-3.786755
208	-3394.859421	-19.332167	0.014575	0.001989	-3.676754
209	-3394.859021	-19.081527	0.015003	0.002051	-3.566754
210	-3394.858606	-18.820644	0.015427	0.002112	-3.456753
211	-3394.858173	-18.549011	0.015855	0.002176	-3.346752
212	-3394.857721	-18.265365	0.016288	0.002240	-3.236750
213	-3394.857250	-17.969801	0.016726	0.002306	-3.126750
214	-3394.856759	-17.661683	0.017171	0.002378	-3.016748
215	-3394.856249	-17.342042	0.017613	0.002454	-2.906746
216	-3394.855716	-17.007212	0.018040	0.002502	-2.796745
217	-3394.855151	-16.652619	0.018474	0.002550	-2.686745
218	-3394.854553	-16.277368	0.018934	0.002623	-2.576742
219	-3394.853930	-15.886429	0.019405	0.002708	-2.466739
220	-3394.853281	-15.479325	0.019882	0.002799	-2.356738
221	-3394.852607	-15.056693	0.020357	0.002894	-2.246738
222	-3394.851908	-14.617874	0.020827	0.002994	-2.136736
223	-3394.851181	-14.161799	0.021287	0.003098	-2.026736
224	-3394.850427	-13.688489	0.021735	0.003207	-1.916732
225	-3394.849643	-13.196843	0.022162	0.003322	-1.806730
226	-3394.848829	-12.685456	0.022563	0.003442	-1.696728
227	-3394.847971	-12.147603	0.022925	0.003569	-1.586726
228	-3394.847090	-11.594619	0.023233	0.003707	-1.476726
229	-3394.846160	-11.010937	0.023476	0.003856	-1.366724
230	-3394.845186	-10.399742	0.023634	0.004021	-1.256722
231	-3394.844150	-9.749440	0.023695	0.004204	-1.146721
232	-3394.843048	-9.057912	0.023639	0.004409	-1.036719
233	-3394.841861	-8.313498	0.024811	0.004637	-0.926718
234	-3394.840569	-7.502422	0.026484	0.004883	-0.816717
235	-3394.839139	-6.605464	0.027640	0.005115	-0.706717
236	-3394.837501	-5.577392	0.027362	0.005239	-0.596712
237	-3394.835479	-4.308759	0.024789	0.005120	-0.486710
238	-3394.833115	-2.824807	0.020704	0.004607	-0.376710
239	-3394.830840	-1.397198	0.015375	0.003532	-0.266702

240	-3394.829276	-0.415822	0.008043	0.001955	-0.156698	
241	-3394.828654	-0.025565	0.002243	0.000500	-0.046693	
242	-3394.828613	0.000000	0.000194	0.000029	0.000000	<= TS
243	-3394.828667	-0.034022	0.002318	0.000496	0.046693	
244	-3394.829061	-0.281125	0.007436	0.001409	0.156695	
245	-3394.829653	-0.652738	0.011450	0.002031	0.266700	
246	-3394.830315	-1.068029	0.014084	0.002420	0.376701	
247	-3394.831011	-1.504492	0.015718	0.002671	0.486704	
248	-3394.831731	-1.956306	0.016731	0.002846	0.596707	
249	-3394.832471	-2.421271	0.017977	0.002976	0.706710	
250	-3394.833227	-2.895211	0.019136	0.003080	0.816714	
251	-3394.833996	-3.377661	0.020132	0.003165	0.926716	
252	-3394.834777	-3.867864	0.021007	0.003238	1.036718	
253	-3394.835565	-4.362160	0.021786	0.003296	1.146719	
254	-3394.836360	-4.861223	0.022486	0.003352	1.256719	
255	-3394.837164	-5.365783	0.023115	0.003403	1.366723	
256	-3394.837975	-5.874626	0.023680	0.003449	1.476723	
257	-3394.838794	-6.388457	0.024184	0.003488	1.586725	
258	-3394.839615	-6.903625	0.024623	0.003523	1.696725	
259	-3394.840443	-7.423401	0.025000	0.003551	1.806729	
260	-3394.841271	-7.943228	0.025308	0.003572	1.916732	
261	-3394.842102	-8.464600	0.025546	0.003587	2.026732	
262	-3394.842935	-8.987421	0.025712	0.003594	2.136735	
263	-3394.843773	-9.513372	0.025808	0.003594	2.246736	
264	-3394.844605	-10.035301	0.025823	0.003585	2.356739	
265	-3394.845433	-10.554844	0.025758	0.003566	2.466742	
266	-3394.846244	-11.063407	0.025605	0.003537	2.576745	
267	-3394.847053	-11.571462	0.025375	0.003497	2.686747	
268	-3394.847850	-12.071221	0.025065	0.003448	2.796749	
269	-3394.848628	-12.559561	0.024678	0.003391	2.906750	
270	-3394.849383	-13.033343	0.024209	0.003326	3.016752	
271	-3394.850115	-13.492545	0.023647	0.003248	3.126755	
272	-3394.850826	-13.938660	0.023000	0.003160	3.236755	
273	-3394.851514	-14.370299	0.022276	0.003062	3.346758	
274	-3394.852176	-14.785990	0.021476	0.002957	3.456759	
275	-3394.852809	-15.183425	0.020602	0.002843	3.566760	
276	-3394.853413	-15.562399	0.019655	0.002720	3.676763	
277	-3394.853986	-15.922026	0.018638	0.002591	3.786763	
278	-3394.854527	-16.261558	0.017556	0.002454	3.896765	
279	-3394.855036	-16.580472	0.016414	0.002311	4.006766	
280	-3394.855510	-16.878329	0.015218	0.002164	4.116766	
281	-3394.855951	-17.154571	0.013979	0.002012	4.226767	
282	-3394.856358	-17.410144	0.012706	0.001858	4.336769	
283	-3394.856731	-17.644509	0.011416	0.001705	4.446772	
284	-3394.857073	-17.859222	0.010121	0.001549	4.556774	
285	-3394.857382	-18.052806	0.008851	0.001408	4.666775	
286	-3394.857668	-18.232019	0.007603	0.001245	4.776776	
287	-3394.857908	-18.382806	0.006568	0.001179	4.886776	
288	-3394.858157	-18.539434	0.006054	0.000949	4.996776	
289	-3394.858344	-18.656508	0.005530	0.001114	5.106777	
290	-3394.858566	-18.795980	0.005051	0.000735	5.216778	
291	-3394.858744	-18.907727	0.004478	0.000733	5.326780	
292	-3394.858917	-19.015766	0.004001	0.000610	5.436780	
293	-3394.859079	-19.117847	0.003575	0.000542	5.546781	
294	-3394.859230	-19.212186	0.003202	0.000523	5.656782	
295	-3394.859377	-19.304793	0.002897	0.000494	5.766782	
296	-3394.859524	-19.396759	0.002646	0.000476	5.876783	
297	-3394.859669	-19.488221	0.002444	0.000466	5.986783	
298	-3394.859816	-19.579977	0.002281	0.000457	6.096784	
299	-3394.859961	-19.671241	0.002152	0.000447	6.206784	
300	-3394.860108	-19.763144	0.002046	0.000436	6.316785	
301	-3394.860254	-19.854874	0.001959	0.000436	6.426785	
302	-3394.860396	-19.944082	0.001953	0.000478	6.536787	

303	-3394.860535	-20.031242	0.002294	0.000495	6.646788
304	-3394.860675	-20.119141	0.002197	0.000488	6.756788
305	-3394.860817	-20.208319	0.002104	0.000482	6.866789
306	-3394.860959	-20.297385	0.002121	0.000483	6.976789
307	-3394.861100	-20.386088	0.002147	0.000488	7.086790
308	-3394.861242	-20.474822	0.002134	0.000487	7.196790
309	-3394.861383	-20.563420	0.002150	0.000489	7.306791
310	-3394.861524	-20.652021	0.002131	0.000488	7.416792
311	-3394.861664	-20.739992	0.002164	0.000493	7.526793
312	-3394.861804	-20.827606	0.002170	0.000495	7.636793
313	-3394.861943	-20.914693	0.002165	0.000495	7.746794
314	-3394.862080	-21.001096	0.002165	0.000495	7.856795
315	-3394.862217	-21.086666	0.002169	0.000496	7.966795
316	-3394.862352	-21.171346	0.002190	0.000497	8.076796
317	-3394.862485	-21.255063	0.002217	0.000497	8.186797
318	-3394.862617	-21.337689	0.002249	0.000498	8.296797
319	-3394.862747	-21.419160	0.002292	0.000501	8.406798
320	-3394.862875	-21.499776	0.002283	0.000496	8.516798
321	-3394.863001	-21.579082	0.002316	0.000497	8.626798
322	-3394.863126	-21.656987	0.002329	0.000495	8.736798
323	-3394.863249	-21.734390	0.002348	0.000494	8.846799
324	-3394.863370	-21.810172	0.002367	0.000494	8.956799
325	-3394.863488	-21.884666	0.002380	0.000492	9.066800
326	-3394.863605	-21.957752	0.002394	0.000490	9.176800
327	-3394.863719	-22.029437	0.002402	0.000488	9.286800
328	-3394.863831	-22.099575	0.002411	0.000487	9.396800
329	-3394.863940	-22.168135	0.002413	0.000484	9.506801
330	-3394.864047	-22.235013	0.002417	0.000482	9.616801
331	-3394.864151	-22.300184	0.002411	0.000479	9.726801
332	-3394.864251	-22.363477	0.002410	0.000477	9.836801
333	-3394.864349	-22.424958	0.002391	0.000473	9.946802
334	-3394.864444	-22.484349	0.002382	0.000471	10.056802
335	-3394.864536	-22.541897	0.002335	0.000465	10.166802
336	-3394.864624	-22.597092	0.002310	0.000464	10.276802
337	-3394.864709	-22.650706	0.002193	0.000453	10.386802
338	-3394.864790	-22.701553	0.002140	0.000456	10.496803
339	-3394.864870	-22.751568	0.001880	0.000432	10.606803
340	-3394.864948	-22.800615	0.001842	0.000454	10.716804
341	-3394.865025	-22.848855	0.001394	0.000398	10.826804
342	-3394.865083	-22.885130	0.001933	0.000476	10.936804
343	-3394.865158	-22.932556	0.001303	0.000318	11.046805
344	-3394.865220	-22.971443	0.001671	0.000405	11.156805
345	-3394.865299	-23.020684	0.001132	0.000250	11.266806
346	-3394.865350	-23.052627	0.002717	0.000519	11.376807
347	-3394.865408	-23.089274	0.002624	0.000479	11.486808
348	-3394.865459	-23.121454	0.002738	0.000488	11.596808
349	-3394.865466	-23.125538	0.002962	0.000518	11.706808
350	-3394.865529	-23.164907	0.001032	0.000266	11.816808
351	-3394.865584	-23.199555	0.000960	0.000257	11.926809
352	-3394.865633	-23.230308	0.000931	0.000265	12.036809
353	-3394.865679	-23.259341	0.001516	0.000298	12.146809
354	-3394.865726	-23.289013	0.001642	0.000292	12.256810
355	-3394.865771	-23.316729	0.001631	0.000293	12.366810
356	-3394.865812	-23.342811	0.001656	0.000294	12.476810
357	-3394.865854	-23.368833	0.001653	0.000292	12.586811
358	-3394.865893	-23.393356	0.001640	0.000293	12.696811
359	-3394.865930	-23.416873	0.001611	0.000290	12.806811
360	-3394.865967	-23.440107	0.001571	0.000285	12.916811
361	-3394.866001	-23.461436	0.001581	0.000285	13.026811
362	-3394.866035	-23.482528	0.001601	0.000282	13.136811
363	-3394.866067	-23.502823	0.001613	0.000281	13.246812
364	-3394.866100	-23.523265	0.001621	0.000280	13.356812
365	-3394.866131	-23.542732	0.001626	0.000281	13.466812

366	-3394.866161	-23.561418	0.001628	0.000281	13.576812
367	-3394.866191	-23.580622	0.001616	0.000280	13.686812
368	-3394.866220	-23.598529	0.001627	0.000281	13.796813
369	-3394.866248	-23.616582	0.001611	0.000278	13.906813
370	-3394.866277	-23.634414	0.001600	0.000274	14.016813
371	-3394.866305	-23.652003	0.001557	0.000272	14.126813
372	-3394.866332	-23.668704	0.001529	0.000265	14.236813
373	-3394.866357	-23.685004	0.001453	0.000260	14.346813
374	-3394.866383	-23.701148	0.001615	0.000278	14.456813
375	-3394.866410	-23.717926	0.001543	0.000267	14.566814
376	-3394.866435	-23.733581	0.001653	0.000282	14.676814
377	-3394.866461	-23.749807	0.001606	0.000273	14.786814
378	-3394.866485	-23.765229	0.001655	0.000281	14.896814
379	-3394.866510	-23.780950	0.001635	0.000277	15.006814
380	-3394.866535	-23.796253	0.001628	0.000276	15.116814
381	-3394.866559	-23.811296	0.001661	0.000281	15.226814
382	-3394.866583	-23.826614	0.001591	0.000268	15.336814
383	-3394.866606	-23.841054	0.001673	0.000284	15.446814
384	-3394.866615	-23.846454	0.001283	0.000251	15.556815
385	-3394.866640	-23.862407	0.000854	0.000170	15.666816
386	-3394.866666	-23.878665	0.000667	0.000172	15.776816
387	-3394.866689	-23.893145	0.000942	0.000207	15.886817
388	-3394.866714	-23.908733	0.000883	0.000199	15.996817
389	-3394.866737	-23.923008	0.000951	0.000212	16.106817
390	-3394.866762	-23.938671	0.000880	0.000227	16.216817
391	-3394.866785	-23.953366	0.000988	0.000241	16.326817
392	-3394.866808	-23.968022	0.001506	0.000271	16.436818
393	-3394.866831	-23.982030	0.002288	0.000332	16.546818
394	-3394.866839	-23.987258	0.001879	0.000300	16.656818
395	-3394.866864	-24.002746	0.000717	0.000181	16.766819
396	-3394.866884	-24.015272	0.000970	0.000246	16.876820
397	-3394.866907	-24.029765	0.000787	0.000180	16.986820
398	-3394.866933	-24.045896	0.000748	0.000169	17.096820
399	-3394.866955	-24.060117	0.001081	0.000228	17.206821
400	-3394.866980	-24.075493	0.000946	0.000230	17.316821
401	-3394.867004	-24.090597	0.000905	0.000229	17.426821
402	-3394.867027	-24.105272	0.000877	0.000228	17.536821
403	-3394.867050	-24.119824	0.000975	0.000234	17.646821
404	-3394.867074	-24.134373	0.001019	0.000238	17.756821
405	-3394.867097	-24.148984	0.001012	0.000238	17.866821
406	-3394.867120	-24.163604	0.000988	0.000234	17.976821
407	-3394.867143	-24.178077	0.000966	0.000233	18.086821
408	-3394.867166	-24.192346	0.000932	0.000235	18.196822
409	-3394.867186	-24.205022	0.000921	0.000236	18.306822
410	-3394.867211	-24.220729	0.000939	0.000230	18.416822
411	-3394.867232	-24.233909	0.000920	0.000222	18.526822
412	-3394.867256	-24.248805	0.000887	0.000214	18.636822
413	-3394.867277	-24.261889	0.000845	0.000205	18.746822
414	-3394.867299	-24.275723	0.000811	0.000198	18.856822
415	-3394.867321	-24.289538	0.000790	0.000195	18.966823
416	-3394.867343	-24.303167	0.000801	0.000196	19.076823
417	-3394.867362	-24.315478	0.000846	0.000203	19.186823
418	-3394.867387	-24.330772	0.000912	0.000214	19.296823
419	-3394.867405	-24.342379	0.000943	0.000224	19.406823
420	-3394.867427	-24.356391	0.000976	0.000233	19.516823
421	-3394.867447	-24.368839	0.000956	0.000234	19.626823
422	-3394.867468	-24.381551	0.000893	0.000235	19.736824
423	-3394.867488	-24.394492	0.000859	0.000230	19.846824
424	-3394.867509	-24.407640	0.001039	0.000221	19.956824
425	-3394.867530	-24.420530	0.001150	0.000211	20.066824
426	-3394.867550	-24.433230	0.001185	0.000202	20.176824
427	-3394.867570	-24.445903	0.001148	0.000195	20.286824
428	-3394.867590	-24.458441	0.001052	0.000189	20.396824

429	-3394.867610	-24.470820	0.000940	0.000185	20.506824
430	-3394.867629	-24.483147	0.000859	0.000181	20.616825
431	-3394.867649	-24.495531	0.000869	0.000179	20.726825
432	-3394.867669	-24.507890	0.001002	0.000182	20.836825
433	-3394.867688	-24.519892	0.001191	0.000196	20.946825
434	-3394.867706	-24.531445	0.001305	0.000218	21.056825
435	-3394.867723	-24.541981	0.001267	0.000235	21.166825
436	-3394.867742	-24.553817	0.001107	0.000246	21.276825
437	-3394.867759	-24.564780	0.000923	0.000247	21.386825
438	-3394.867777	-24.575833	0.000990	0.000245	21.496825
439	-3394.867794	-24.586741	0.001015	0.000240	21.606825
440	-3394.867812	-24.597461	0.001003	0.000234	21.716825
441	-3394.867828	-24.607767	0.001028	0.000235	21.826825
442	-3394.867845	-24.618549	0.000844	0.000199	21.936826
443	-3394.867860	-24.628096	0.000840	0.000200	22.046826
444	-3394.867876	-24.637860	0.000961	0.000184	22.156826
445	-3394.867889	-24.645846	0.001540	0.000247	22.266826
446	-3394.867893	-24.648581	0.001349	0.000237	22.376826
447	-3394.867908	-24.657826	0.001155	0.000188	22.486826
448	-3394.867924	-24.667989	0.000536	0.000127	22.596826
449	-3394.867934	-24.674571	0.000595	0.000165	22.706826
450	-3394.867948	-24.682850	0.001120	0.000226	22.816827
451	-3394.867959	-24.690060	0.001138	0.000234	22.926827
452	-3394.867967	-24.694811	0.000903	0.000212	23.036828
453	-3394.867980	-24.702957	0.000741	0.000143	23.146828
454	-3394.867995	-24.712796	0.000340	0.000085	23.256828
455	-3394.868011	-24.722523	0.000494	0.000092	23.366828
456	-3394.868024	-24.730500	0.000426	0.000092	23.476828
457	-3394.868039	-24.740158	0.000333	0.000083	23.586828
458	-3394.868054	-24.749894	0.000264	0.000073	23.696828
459	-3394.868070	-24.759367	0.000239	0.000068	23.806829
460	-3394.868084	-24.768433	0.000280	0.000069	23.916829
461	-3394.868108	-24.783457	0.000344	0.000076	24.026829
462	-3394.868104	-24.780956	0.000377	0.000080	24.136829
463	-3394.868115	-24.787652	0.001126	0.000219	24.246829
464	-3394.868129	-24.796348	0.000357	0.000122	24.356830
465	-3394.868146	-24.807077	0.000405	0.000092	24.466830
466	-3394.868153	-24.811856	0.000337	0.000082	24.576830
467	-3394.868165	-24.819445	0.000415	0.000107	24.686830
468	-3394.868177	-24.826477	0.000496	0.000134	24.796830
469	-3394.868187	-24.832740	0.000470	0.000139	24.906830
470	-3394.868198	-24.839652	0.000421	0.000136	25.016830
471	-3394.868207	-24.845891	0.000434	0.000131	25.126830
472	-3394.868218	-24.852367	0.000427	0.000130	25.236830
473	-3394.868229	-24.859390	0.000443	0.000132	25.346830
474	-3394.868240	-24.866116	0.000502	0.000135	25.456831
475	-3394.868250	-24.872536	0.000509	0.000128	25.566831
476	-3394.868260	-24.878632	0.000481	0.000118	25.676831
477	-3394.868269	-24.884280	0.000472	0.000117	25.786831
478	-3394.868278	-24.889870	0.000496	0.000125	25.896831
479	-3394.868286	-24.895385	0.000517	0.000133	26.006831
480	-3394.868295	-24.900818	0.000530	0.000134	26.116831
481	-3394.868304	-24.906371	0.000528	0.000130	26.226831
482	-3394.868313	-24.911831	0.000499	0.000118	26.336831
483	-3394.868321	-24.916923	0.000498	0.000114	26.446831

SUGGESTED CITATIONS FOR THIS RUN

Below you find a list of papers that are relevant to this ORCA run
We neither can nor want to force you to cite these papers, but we appreciate if you do

You receive ORCA, which is the product of decades of hard work by many enthusiastic individuals, for free
The only thing we kindly ask in return is that you cite our papers,
We deeply appreciate it, if you show your appreciation for ORCA by not just citing the generic ORCA reference.

Please note that relegating all ORCA citations to the supporting information does *not* help us.
SI sections are not indexed - citations you put there will not count into any citation statistics
But we need these citations in order to attract the funding resources that allow us to do what we are doing

Therefore, if you are a happy ORCA user, please consider citing a few of the papers listed below in the main body of your paper

In addition to the list printed below, the program has created the file CF3-IRC.bibtex that contains the list in bibtex format
You can import this file easily into all common literature databanks and citation aid programs

It goes without saying that in many instances, there are alternative algorithms to achieve similar
results as the ones you have gotten from ORCA. It is, of course, also the case that in some instances
ORCA just re-implements algorithms worked out by others. We are fully aware of that and we are also
fully appreciative of our colleagues work. Hence this citation list should not be read as indicating
that the listed papers, which are focused on our own work, are the only ones worth citing. It simply
meant to make it easier for users to cite ORCA specific papers. It is not a substitute for doing your
own literature research and citing the relevant literature in a scientifically appropriate manner.

List of essential papers. We consider these as the minimum necessary citations

1. Neese, F.
   Software update: the ORCA program system, version 6.0
   WIRES Comput. Molec. Sci. 2025 15(1), e70019
   doi.org/10.1002/wcms.70019

List of papers to cite with high priority. The work reported in these papers was absolutely necessary for this run to complete.
Our perspective: the developers of density functionals and basis sets usually get cited in chemistry papers
Good! But without the algorithms to do something with them, the functionals or basis sets would not do anything.
Hence, in our opinion, the algorithm design and method developments papers are equally worthy of getting cited

1. Page, M.; Doubleday, C.; McIver, J.
   Following steepest descent reaction paths. The use of higher energy derivatives with abinitio electronic structure methods
   J. Chem. Phys. 1990 93(8), 5634-5642
   doi.org/10.1063/1.459634
2. Neese, F.
   An improvement of the resolution of the identity approximation for the formation of the Coulomb matrix
   J. Comp. Chem. 2003 24(14), 1740-1747
   doi.org/10.1002/jcc.10318
3. Hratchian, H.; Schlegel, B.

Using Hessian Updating To Increase the Efficiency of a Hessian Based Predictor-Corrector Reaction Path Following Method
J. Chem. Theory Comput. 2005 1(1), 61-69
doi.org/10.1021/ct0499783
4. Neese, F.; Wennmohs, F.; Hansen, A.; Becker, U.
Efficient, approximate and parallel Hartree-Fock and hybrid DFT calculations. A 'chain-of-spheres' algorithm for the Hartree-Fock exchange
Chem. Phys. 2009 356(1-3), 98-109
doi.org/10.1016/j.chemphys.2008.10.036
5. Grimme, S.; Antony, J.; Ehrlich, S.; Krieg, H.
A consistent and accurate ab initio parametrization of density functional dispersion correction (DFT-D) for the 94 elements H-Pu
J. Chem. Phys. 2010 132 , 154104
doi.org/10.1063/1.3382344
6. Grimme, S.; Ehrlich, S.; Goerigk, L.
Effect of the damping function in dispersion corrected density functional theory
J. Comput. Chem. 2011 32(7), 1456-1465
doi.org/10.1002/jcc.21759
7. Garcia-Rates, M.; Neese, F.
Effect of the Solute Cavity on the Solvation Energy and its Derivatives within the Framework of the Gaussian Charge Scheme
J. Comput. Chem. 2020 41 , 922-939
doi.org/10.1002/jcc.26139
8. Helmich-Paris, B.; de Souza, B.; Neese, F.; Izsák, R.
An improved chain of spheres for exchange algorithm
J. Chem. Phys. 2021 155(10), 104109
doi.org/10.1063/5.0058766
9. Neese, F.
The SHARK Integral Generation and Digestion System
J. Comp. Chem. 2022 44(3), 381
doi.org/10.1002/jcc.26942

List of suggested additional citations. These are papers that are important in the 'surrounding' of
of this run, or papers that preceded the highly important papers. If you like your results we are grateful for a citation.

1. Izsak, R.; Neese, F.
An overlap fitted chain of spheres exchange method
J. Chem. Phys. 2011 135 , 144105
doi.org/10.1063/1.3646921
2. Izsak, R.; Hansen, A.; Neese, F.
The resolution of identity and chain of spheres approximations for the LPNO-CCSD singles Fock term
Molec. Phys. 2012 110 , 2413-2417
doi.org/10.1080/00268976.2012.687466
3. Neese, F.
The ORCA program system
WIRES Comput. Molec. Sci. 2012 2(1), 73-78
doi.org/10.1002/wcms.81
4. Izsak, R.; Neese, F.; Klopper, W.
Robust fitting techniques in the chain of spheres approximation to the Fock exchange: The role of the complementary space
J. Chem. Phys. 2013 139 ,
doi.org/10.1063/1.4819264
5. Neese, F.
Software update: the ORCA program system, version 4.0
WIRES Comput. Molec. Sci. 2018 8(1), 1-6
doi.org/10.1002/wcms.1327
6. Neese, F.; Wennmohs, F.; Becker, U.; Riplinger, C.
The ORCA quantum chemistry program package
J. Chem. Phys. 2020 152(22), 224108
doi.org/10.1063/5.0004608

```
  7. Neese, F.
     Software update: The ORCA program system—Version 5.0
     WIRES Comput. Molec. Sci. 2022 12(1), e1606
     doi.org/10.1002/wcms.1606

List of optional additional citations

  1. Neese, F.
     Approximate second-order SCF convergence for spin unrestricted wavefunctions
     Chem. Phys. Lett. 2000 325(1-3), 93-98
     doi.org/10.1016/s0009-2614(00)00662-x

Timings for individual modules:

Sum of individual times          ...   146347.439 sec (=2439.124 min)
Startup calculation              ...     3692.462 sec (=  61.541 min)   2.5 %
SCF iterations                   ...    88079.381 sec (=1467.990 min)  60.2 %
Property calculations            ...        0.455 sec (=   0.008 min)   0.0 %
SCF Gradient evaluation          ...    54575.141 sec (= 909.586 min)  37.3 %
                             ****ORCA TERMINATED NORMALLY****
TOTAL RUN TIME: 1 days 17 hours 0 minutes 7 seconds 109 msec
'CF3-IRC.bibtex' -> '/hsnfs/users/barasits/Tunneling_selenoxide/CF3/IRC/CF3-IRC.bibtex'
'CF3-IRC.cpcm' -> '/hsnfs/users/barasits/Tunneling_selenoxide/CF3/IRC/CF3-IRC.cpcm'
'CF3-IRC.cpcm_corr' -> '/hsnfs/users/barasits/Tunneling_selenoxide/CF3/IRC/CF3-IRC.cpcm_corr'
'CF3-IRC.densities' -> '/hsnfs/users/barasits/Tunneling_selenoxide/CF3/IRC/CF3-IRC.densities'
'CF3-IRC.densitiesinfo' -> '/hsnfs/users/barasits/Tunneling_selenoxide/CF3/IRC/CF3-
IRC.densitiesinfo'
'CF3-IRC.engrad' -> '/hsnfs/users/barasits/Tunneling_selenoxide/CF3/IRC/CF3-IRC.engrad'
'CF3-IRC.err' -> '/hsnfs/users/barasits/Tunneling_selenoxide/CF3/IRC/CF3-IRC.err'
'CF3-IRC.gbw' -> '/hsnfs/users/barasits/Tunneling_selenoxide/CF3/IRC/CF3-IRC.gbw'
'CF3-IRC_IRC_B.cpcm' -> '/hsnfs/users/barasits/Tunneling_selenoxide/CF3/IRC/CF3-IRC_IRC_B.cpcm'
'CF3-IRC_IRC_B.cpcm_corr' -> '/hsnfs/users/barasits/Tunneling_selenoxide/CF3/IRC/CF3-
IRC_IRC_B.cpcm_corr'
'CF3-IRC_IRC_B.densities' -> '/hsnfs/users/barasits/Tunneling_selenoxide/CF3/IRC/CF3-
IRC_IRC_B.densities'
'CF3-IRC_IRC_B.densitiesinfo' -> '/hsnfs/users/barasits/Tunneling_selenoxide/CF3/IRC/CF3-
IRC_IRC_B.densitiesinfo'
'CF3-IRC_IRC_B.gbw' -> '/hsnfs/users/barasits/Tunneling_selenoxide/CF3/IRC/CF3-IRC_IRC_B.gbw'
'CF3-IRC_IRC_B.smd' -> '/hsnfs/users/barasits/Tunneling_selenoxide/CF3/IRC/CF3-IRC_IRC_B.smd'
'CF3-IRC_IRC_B.smd.grd' -> '/hsnfs/users/barasits/Tunneling_selenoxide/CF3/IRC/CF3-
IRC_IRC_B.smd.grd'
'CF3-IRC_IRC_B_trj.xyz' -> '/hsnfs/users/barasits/Tunneling_selenoxide/CF3/IRC/CF3-
IRC_IRC_B_trj.xyz'
'CF3-IRC_IRC_B.xyz' -> '/hsnfs/users/barasits/Tunneling_selenoxide/CF3/IRC/CF3-IRC_IRC_B.xyz'
'CF3-IRC_IRC_F.cpcm' -> '/hsnfs/users/barasits/Tunneling_selenoxide/CF3/IRC/CF3-IRC_IRC_F.cpcm'
'CF3-IRC_IRC_F.cpcm_corr' -> '/hsnfs/users/barasits/Tunneling_selenoxide/CF3/IRC/CF3-
IRC_IRC_F.cpcm_corr'
'CF3-IRC_IRC_F.densities' -> '/hsnfs/users/barasits/Tunneling_selenoxide/CF3/IRC/CF3-
IRC_IRC_F.densities'
'CF3-IRC_IRC_F.densitiesinfo' -> '/hsnfs/users/barasits/Tunneling_selenoxide/CF3/IRC/CF3-
IRC_IRC_F.densitiesinfo'
'CF3-IRC_IRC_F.gbw' -> '/hsnfs/users/barasits/Tunneling_selenoxide/CF3/IRC/CF3-IRC_IRC_F.gbw'
'CF3-IRC_IRC_F.smd' -> '/hsnfs/users/barasits/Tunneling_selenoxide/CF3/IRC/CF3-IRC_IRC_F.smd'
'CF3-IRC_IRC_F.smd.grd' -> '/hsnfs/users/barasits/Tunneling_selenoxide/CF3/IRC/CF3-
IRC_IRC_F.smd.grd'
'CF3-IRC_IRC_F_trj.xyz' -> '/hsnfs/users/barasits/Tunneling_selenoxide/CF3/IRC/CF3-
IRC_IRC_F_trj.xyz'
'CF3-IRC_IRC_Full_trj.xyz' -> '/hsnfs/users/barasits/Tunneling_selenoxide/CF3/IRC/CF3-
IRC_IRC_Full_trj.xyz'
'CF3-IRC_IRC_F.xyz' -> '/hsnfs/users/barasits/Tunneling_selenoxide/CF3/IRC/CF3-IRC_IRC_F.xyz'
'CF3-IRC.job' -> '/hsnfs/users/barasits/Tunneling_selenoxide/CF3/IRC/CF3-IRC.job'
'CF3-IRC.property.txt' -> '/hsnfs/users/barasits/Tunneling_selenoxide/CF3/IRC/CF3-
IRC.property.txt'
```

```
'CF3-IRC.smd' -> '/hsnfs/users/barasits/Tunneling_selenoxide/CF3/IRC/CF3-IRC.smd'
'CF3-IRC.smd.grd' -> '/hsnfs/users/barasits/Tunneling_selenoxide/CF3/IRC/CF3-IRC.smd.grd'
'TS-CF3_Compound_3.hess' -> '/hsnfs/users/barasits/Tunneling_selenoxide/CF3/IRC/TS-
CF3_Compound_3.hess'
cp: cannot stat 'CF3-IRC.asa.inp': No such file or directory
cp: missing destination file operand after
'/hsnfs/users/barasits/Tunneling_selenoxide/CF3/IRC/'
Try 'cp --help' for more information.
cp: cannot stat 'CF3-IRC.pc.tmp': No such file or directory
cp: cannot stat 'CF3-IRC.QM2.pc.tmp': No such file or directory
```

IRC output for R = CN

```
! Job execution by suborca V 10.06 !
Job started from head2.mpi-muelheim.mpg.de, running
/hsnfs/software/orca/orca_current_directory/orca_current_20251216/orca on xe30th116.mpi-
muelheim.mpg.de in /usr/scratch/barasits/CN-IRC__26969135 (/bin/bash)
Job execution start: Tue Dec 16 12:05:49 PM CET 2025
Shared library path: /hsnfs/software/openmpi-
4.1.0/lib:/hsnfs/software/orca/orca_current_directory/orca_current_20251216:/hsnfs/software/the
mis/llvm-14.0.1/lib:/hsnfs/software/orca/orca_stable:/usr/users/openmpi-
2.0.4/lib:/opt/intel/lib/intel64:/opt/intel/mkl/lib/intel64
SLURM Job ID is: 26969135
SLURM_JOB_NODELIST: xe30th116
Maxcore enforced by SLURM: 9600 MB per core
Expanded nodelist (1 nodes):
xe30th116

                                 *****************
                                 * O   R   C   A *
                                 *****************

                                            #,
                                            ###
                                            ####
                                            #####
                                            ######
                                           ########,
                                     ,,################,,,,,
                               ,,#################################,,
                          ,,##########################################,,
                       ,#########################################, ''#####,
                    ,#############################################,,   '####,
                  ,##################################################,,,,####,
                ,###########''''           ''''###############################
              ,#####''   ,,,,##########,,,,          '''####'''          '####
            ,##' ,,,,###########################,,,                        '##
           ' ,,###''''                  '''############,,,
         ,,##''                                '''############,,,,        ,,,,,,###''
      ,#''                                            '''#######################'''
     '                                                          ''''####''''
             ,#######,   #######,   ,#######,      ##
            ,#'     '#,  ##    ##  ,#'     '#,    #''#        ,####,    ,#,
            ##       ##  ##   ,#'  ##            #'  '#       #'       ,# #
            ##       ##  #######   ##           ,######,      #####,      #
            '#,     ,#'  ##    ##  '#,     ,#' ,#      #,     #,   #      #
             '#######'   ##     ##  '#######'  #'      '#     '####' #    #


               #######################################################
               #                        -***-                        #
               #          Department of theory and spectroscopy      #
               #                                                     #
               #                      Frank Neese                    #
               #                                                     #
               #     Directorship, Architecture, Infrastructure      #
               #                    SHARK, DRIVERS                   #
               #        Core code/Algorithms in most modules         #
               #                                                     #
               #        Max Planck Institute fuer Kohlenforschung    #
               #                Kaiser Wilhelm Platz 1               #
               #                 D-45470 Muelheim/Ruhr               #
               #                      Germany                        #
               #                                                     #
               #                  All rights reserved                #
```

```
                #                          -***-                         #
                #########################################################


                         Program Version 6.1.x  -  CURRENT   -
                                (GIT: $2a5ddc4e$)
                ($2025-12-15 18:08:28 +0100 $)

                  !                     WARNING:                        !
                  !         This is a DEVELOPMENT version               !

                         (BUILD: $Tue 16 Dec 2025 12:05:34 AM CET$)


 With contributions from (in alphabetic order):
[Max-Planck-Institut fuer Kohlenforschung]
  Daniel Aravena         : Magnetic Suceptibility
  Michael Atanasov       : Ab Initio Ligand Field Theory (pilot matlab implementation)
  Alexander A. Auer      : GIAO ZORA, VPT2 properties, NMR spectrum
  Ute Becker             : All parallelization in ORCA, NUMFREQ, NUMCALC
  Giovanni Bistoni       : ED, misc. LED, open-shell LED, HFLD
  Dmytro Bykov           : pre 5.0 version of the SCF Hessian
  Marcos Casanova-Páez   : Triplet and SCS-CIS(D). UHF-(DLPNO)-IP/EA/STEOM-CCSD. UHF-CVS-
IP/STEOM-CCSD
  Vijay G. Chilkuri      : MRCI spin determinant printing, contributions to CSF-ICE
  Pauline Colinet        : FMM embedding
  Dipayan Datta          : RHF DLPNO-CCSD density
  Achintya Kumar Dutta   : EOM-CC, STEOM-CC
  Nicolas Foglia         : Exact transition moments, OPA infrastructure, MCD improvements
  Dmitry Ganyushin       : Spin-Orbit,Spin-Spin,Magnetic field MRCI
  Miquel Garcia-Rates    : C-PCM and meta-GGA Hessian, CCSD/C-PCM, Gaussian charge scheme
  Tiago L. C. Gouveia    : GS-ROHF, GS-ROCIS
  Yang Guo               : DLPNO-NEVPT2, F12-NEVPT2, CIM, IAO-localization
  Andreas Hansen         : Spin unrestricted coupled pair/coupled cluster methods
  Ingolf Harden          : AUTO-CI MPn and infrastructure
  Benjamin Helmich-Paris : MC-RPA, TRAH-(SCF,CASSCF), AVAS, COSX integrals, SCF dyn. polar.,
MC-PDFT, srDFT
  Lee Huntington         : MR-EOM, pCC
  Robert Izsak           : Overlap fitted RIJCOSX, COSX-SCS-MP3, EOM
  Riya Kayal             : Wick's Theorem for AUTO-CI, AUTO-CI UHF-CCSDT
  Emily Kempfer          : AUTO-CI RHF CISDT and CCSDT, approximate NEVPT4
  Christian Kollmar      : KDIIS, OOCD, Brueckner-CCSD(T), CCSD density, CASPT2, CASPT2-K,
improved NEVPT2
  Axel Koslowski         : Symmetry handling
  Simone Kossmann        : meta-GGA functionals, TD-DFT gradient, OOMP2, (MP2 Hessian;
deprecated post 5.0)
  Lucas Lang             : DCDCAS, Hyperfine gauge corrections, ICE-SOC+SSC
  Marvin Lechner         : AUTO-CI (C++ implementation), FIC-MRCC
  Spencer Leger          : CASSCF response
  Dagmar Lenk            : GEPOL surface, SMD, ORCA-2-JSON
  Dimitrios Liakos       : Extrapolation schemes; Compound Job, Property file
  Dimitrios Manganas     : Further ROCIS development; embedding schemes. LFT, Crystal Embedding
  Hagen Neugebauer       : wr2SCAN, Native XTB
  Dimitrios Pantazis     : SARC Basis sets
  Anastasios Papadopoulos: AUTO-CI, single reference methods and gradients
  Taras Petrenko         : pre 6.0 DFT Hessian and TD-DFT gradient,  ECA, NRVS
  Petra Pikulova         : Analytic Raman intensities
  Peter Pinski           : DLPNO-MP2, DLPNO-MP2 Gradient
  Shashank Vittal Rao    : ES-AILFT, MagRelax
  Christoph Reimann      : Effective Core Potentials
  Marius Retegan         : Local ZFS, SOC
  Christoph Riplinger    : Optimizer, TS searches, QM/MM, DLPNO-CCSD(T), (RO)-DLPNO pert.
Triples
```

```
  Michael Roemelt        : Original ROCIS implementation, recursive CI coupling coefficients
  Masaaki Saitow         : Open-shell DLPNO-CCSD energy and density
  Barbara Sandhoefer     : DKH picture change effects
  Yorick L. A. Schmerwitz: GMF and freeze-and-release deltaSCF, NEB S-IDPP initial path
  Kantharuban Sivalingam : CASSCF convergence/infrastructure, NEVPT2, NEVPT3, NEVPT4(SD), FIC-
MRCI and CEPA variants
  Bernardo de Souza      : ESD, SOC TD-DFT
  Georgi L. Stoychev     : AutoAux, RI-MP2 NMR, DLPNO-MP2 response, X2C
  Van Anh Tran           : RI-MP2 g-tensors
  Willem Van den Heuvel  : Paramagnetic NMR
  Zikuan Wang            : NOTCH, Electric field optimization
  Frank Wennmohs         : Technical directorship and infrastructure
  Hang Xu                : AUTO-CI-Response properties

[FACCTs GmbH]
  Markus Bursch, Nicolas Foglia, Miquel Garcia-Rates, Ingolf Harden, Hagen Neugebauer,
Anastasios Papadopoulos,
  Christoph Riplinger, Bernardo de Souza, Georgi L. Stoychev

  APM, various basis sets, CI-OPT, improved COSX, DLPNO-Multilevel,
  DOCKER, DRACO, updates on ESD, Fragmentator, GOAT, IRC, LR-CPCM, L-BFGS, MBIS, meta-GGA TD-
DFT gradient, ML-optimized integration grids,
  MM, NACMEs, nearIR, NEB, NEB-TS, NL-DFT gradient (VV10), 2- and 3-layer-ONIOM, interface
openCOSMO-RS, QMMM,
  Crystal-QMMM, RESP, rigid body optimization, SF, symmetry and pop. for TD-DFT, various
functionals, SOLVATOR

[Other institutions]
  V. Asgeirsson          : NEB
  Christoph Bannwarth    : sTDA-DFT, sTD-DFT, PBEh-3c, B97-3c, D3
  Giovanni Bistoni       : ETS/NOCV, ADLD/ADEX, COVALED
  Martin Brehm           : Molecular dynamics
  Ronald Cardenas        : ETS/NOCV
  Martina Colucci        : COVALED
  Sebastian Ehlert       : rSCAN, r2SCAN, r2SCAN-3c, D4, dhf basis sets
  Marvin Friede          : D4 for Fr, Ra, Ac-Lr
  Lars Goerigk           : TD-DFT with DH, B97 family of functionals
  Stefan Grimme          : VdW corrections, initial TS optimization, DFT functionals, gCP,
sTDA/sTD-DF
  Waldemar Hujo          : DFT-NL
  H. Jonsson             : NEB
  Holger Kruse           : gCP
  Marcel Mueller         : wB97X-3c, vDZP basis set
  Gianluca Regni         : ADLD/ADEX
  Tobias Risthaus        : pre 6.0 range-separated hybrid DFT and stability analysis
  Lukas Wittmann         : regularized MP2, r2SCAN double-hybrids, wr2SCAN

We gratefully acknowledge several colleagues who have allowed us to
interface, adapt or use parts of their codes:
  Ed Valeev, F. Pavosevic, A. Kumar             : LibInt (2-el integral package), F12 methods
  Garnet Chan, S. Sharma, J. Yang, R. Olivares  : DMRG
  Ulf Ekstrom                                   : XCFun DFT Library
  Mihaly Kallay                                 : mrcc  (arbitrary order and MRCC methods)
  Frank Weinhold                                : gennbo (NPA and NBO analysis)
  Simon Mueller                                 : openCOSMO-RS
  Christopher J. Cramer and Donald G. Truhlar   : smd solvation model
  S Lehtola, MJT Oliveira, MAL Marques          : LibXC Library
  Liviu Ungur et al                             : ANISO software


 Your calculation uses the libint2 library for the computation of 2-el integrals
 For citations please refer to: http://libint.valeyev.net
```

```
 Your ORCA version has been built with support for libXC version: 7.0.0
 For citations please refer to: https://libxc.gitlab.io

 This ORCA versions uses:
   CBLAS   interface :  Fast vector & matrix operations
   LAPACKE interface :  Fast linear algebra routines
   SCALAPACK package :  Parallel linear algebra routines
   Shared memory     :  Shared parallel matrices
   BLAS/LAPACK       :  OpenBLAS 0.3.26  USE64BITINT DYNAMIC_ARCH NO_AFFINITY Haswell
SINGLE_THREADED
        Core in use  :  Haswell
   Copyright (c) 2011-2014, The OpenBLAS Project


  ***********************************
  * Starting time: Tue Dec 16 12:05:49 2025
  * Host name:     xe30th116.mpi-muelheim.mpg.de
  * Process ID:    2005241
  * Working dir.:  /usr/scratch/barasits/CN-IRC__26969135
  ***********************************

NOTE: MaxCore=8000 MB was set to SCF,MP2,MDCI,CIPSI,MRCI,RASCI and CIS
      => If you want to overwrite this, your respective input block should be placed after the
MaxCore statement
Your calculation utilizes the atom-pairwise dispersion correction
with the Becke-Johnson damping scheme (D3BJ)


Your calculation utilizes the SMD solvation module
Please cite in your paper:
Marenich, A. V.; Cramer, C. J.; Truhlar, D. G. J. Phys. Chem. B 2009, 113, 6378

================================================================================

----- Orbital basis set information -----
Your calculation utilizes the basis: def2-TZVP
   F. Weigend and R. Ahlrichs, Phys. Chem. Chem. Phys. 7, 3297 (2005).

----- AuxJ basis set information -----
Your calculation utilizes the auxiliary basis: def2/J
   H-Rn: F. Weigend, Phys. Chem. Chem. Phys. 8, 1057 (2006).
   Fr-Lr: K. Eichkorn, F. Weigend, O. Treutler, R. Ahlrichs; Theor. Chem. Acc. 97, 119 (1997).

================================================================================
                                        WARNINGS
                       Please study these warnings very carefully!
================================================================================

WARNING: SinglePoint found together with IRC
         Switching on EnGrad - energy plus gradient calculation

================================================================================
                                       INPUT FILE
================================================================================
NAME = CN-IRC.inp
|  1> #
|  2> #IRC LQA
|  3> #
|  4> ! irc B3LYP D3BJ def2-TZVP def2/J verytightscf
|  5> %pal nprocs 12 end
|  6> %maxcore 8000
|  7> %irc
|  8> printlevel 1
```

```
|  9> method lqa
| 10> maxiter 241
| 11> tolrmsg 1.e-5 # Tighter convergence criteria
| 12> tolmaxg 8.e-5 # Tighter convergence criteria
| 13> follow_coordtype massweighted
| 14> inithess read
| 15> hess_filename "TS-CN_Compound_2.hess"
| 16> lqahess lqacalc_bofillupdate
| 17> init_displ length
| 18> scale_init_displ 0.04
| 19> adapt_scale_displ false
| 20> scale_displ_sd 0.11
| 21> ENFORCESTRICTCONVERGENCE true
| 22> REPORTPROFILE true
| 23> BSCORRECTOR true
| 24> BSRICHTHRESH 1E-6
| 25> INITBSCORRECTOR false
| 26> end
| 27> %CPCM SMD true
| 28>      SMDSOLVENT "TETRAHYDROFURAN" END
| 29> 
| 30> * xyz 0 1
| 31>   C         -1.77242691535023      0.35764577579332     -6.04284326329639
| 32>   C         -1.24513308044884     -0.29185151429486     -4.92697042059703
| 33>   C         -0.98025429092625      1.31005911485483     -6.68990496860998
| 34>   C          0.03350147714416      0.00225503051936     -4.46573887185988
| 35>   C          0.30238057931614      1.60027379808288     -6.23957668980280
| 36>   C          0.81420425150147      0.94603405023154     -5.12287161720221
| 37>   H         -1.84369132009485     -1.03557458482369     -4.41453517977957
| 38>   H         -1.37705443382930      1.83115411546006     -7.55358139265468
| 39>   H          0.42262920593828     -0.51205942194717     -3.59655668466148
| 40>   H          0.90099552496254      2.34006807634732     -6.75710357728518
| 41>   H          1.81327672420764      1.16820048697840     -4.76912812321233
| 42>   C         -3.15711021350337      0.04283319592225     -6.54286179313954
| 43>   H         -3.62786892958744      0.97279891376756     -6.87654949576556
| 44>   H         -3.76215566517727     -0.33162765008727     -5.71459806457575
| 45>   C         -3.23171370816286     -0.96506985558072     -7.70203265496649
| 46>   H         -3.12349358911175     -2.15390202634392     -7.16798213190321
| 47>   H         -4.23857931371867     -1.05142895283341     -8.11233934771858
| 48>   C         -2.20855155101092     -1.06497696223903     -8.66200525161729
| 49>   H         -2.44294820747503     -1.33503196679574     -9.68261640027021
| 50>   H         -1.24763702639749     -0.58917241449077     -8.51902986893319
| 51>   Se        -1.41546583943925     -3.40793771537516     -8.11551418574199
| 52>   O         -2.69818006766329     -3.39403145314802     -6.97598715289002
| 53>   C          0.10451329895762     -2.98407392230421     -6.99684300467531
| 54>   C          0.12985384439283     -3.41946140691141     -5.67535225383118
| 55>   C          1.18625041112994     -2.29972435844452     -7.54442997672785
| 56>   C          1.24518266940499     -3.17367442192366     -4.89364921532387
| 57>   C          2.30366737411746     -2.04661820102484     -6.76623261291995
| 58>   C          2.33590643260710     -2.48721714471770     -5.43887879396682
| 59>   H         -0.72269713455429     -3.93910586917340     -5.26005742318268
| 60>   H          1.15547424841149     -1.95287620438934     -8.56938019149265
| 61>   H          1.27553613574436     -3.50251819165961     -3.86384330502029
| 62>   H          3.14364894819371     -1.50140407410422     -7.17439351677692
| 63>   C          3.47437667053850     -2.20499475682017     -4.62783736727402
| 64>   N          4.39188096988287     -1.96901557852466     -3.97039661232491
| 65> *
| 66> 
| 67> 
| 68>                          ****END OF INPUT****
================================================================================

                     ******************************
```

```
                    * Energy+Gradient Calculation *
                    *******************************

---------------------------------
CARTESIAN COORDINATES (ANGSTROEM)
---------------------------------
  C     -1.772427    0.357646   -6.042843
  C     -1.245133   -0.291852   -4.926970
  C     -0.980254    1.310059   -6.689905
  C      0.033501    0.002255   -4.465739
  C      0.302381    1.600274   -6.239577
  C      0.814204    0.946034   -5.122872
  H     -1.843691   -1.035575   -4.414535
  H     -1.377054    1.831154   -7.553581
  H      0.422629   -0.512059   -3.596557
  H      0.900996    2.340068   -6.757104
  H      1.813277    1.168200   -4.769128
  C     -3.157110    0.042833   -6.542862
  H     -3.627869    0.972799   -6.876549
  H     -3.762156   -0.331628   -5.714598
  C     -3.231714   -0.965070   -7.702033
  H     -3.123494   -2.153902   -7.167982
  H     -4.238579   -1.051429   -8.112339
  C     -2.208552   -1.064977   -8.662005
  H     -2.442948   -1.335032   -9.682616
  H     -1.247637   -0.589172   -8.519030
  Se    -1.415466   -3.407938   -8.115514
  O     -2.698180   -3.394031   -6.975987
  C      0.104513   -2.984074   -6.996843
  C      0.129854   -3.419461   -5.675352
  C      1.186250   -2.299724   -7.544430
  C      1.245183   -3.173674   -4.893649
  C      2.303667   -2.046618   -6.766233
  C      2.335906   -2.487217   -5.438879
  H     -0.722697   -3.939106   -5.260057
  H      1.155474   -1.952876   -8.569380
  H      1.275536   -3.502518   -3.863843
  H      3.143649   -1.501404   -7.174394
  C      3.474377   -2.204995   -4.627837
  N      4.391881   -1.969016   -3.970397

----------------------------
CARTESIAN COORDINATES (A.U.)
----------------------------
  NO LB      ZA    FRAG     MASS         X           Y           Z
   0 C     6.0000    0    12.011   -3.349401    0.675853  -11.419319
   1 C     6.0000    0    12.011   -2.352961   -0.551519   -9.310625
   2 C     6.0000    0    12.011   -1.852412    2.475653  -12.642088
   3 C     6.0000    0    12.011    0.063309    0.004261   -8.439023
   4 C     6.0000    0    12.011    0.571416    3.024079  -11.791091
   5 C     6.0000    0    12.011    1.538623    1.787745   -9.680824
   6 H     1.0000    0     1.008   -3.484072   -1.956952   -8.342262
   7 H     1.0000    0     1.008   -2.602256    3.460380  -14.274200
   8 H     1.0000    0     1.008    0.798653   -0.967652   -6.796507
   9 H     1.0000    0     1.008    1.702635    4.422088  -12.769075
  10 H     1.0000    0     1.008    3.426596    2.207579   -9.012346
  11 C     6.0000    0    12.011   -5.966074    0.080943  -12.364217
  12 H     1.0000    0     1.008   -6.855679    1.838324  -12.994795
  13 H     1.0000    0     1.008   -7.109444   -0.626685  -10.799025
  14 C     6.0000    0    12.011   -6.107054   -1.823718  -14.554732
  15 H     1.0000    0     1.008   -5.902547   -4.070285  -13.545523
  16 H     1.0000    0     1.008   -8.009754   -1.986913  -15.330100
  17 C     6.0000    0    12.011   -4.173558   -2.012515  -16.368818
```

```
  18 H      1.0000    0     1.008   -4.616503   -2.522845  -18.297493
  19 H      1.0000    0     1.008   -2.357692   -1.113375  -16.098633
  20 Se    34.0000    0    78.960   -2.674843   -6.440069  -15.336099
  21 O      8.0000    0    15.999   -5.098821   -6.413790  -13.182705
  22 C      6.0000    0    12.011    0.197502   -5.639082  -13.222117
  23 C      6.0000    0    12.011    0.245388   -6.461846  -10.724861
  24 C      6.0000    0    12.011    2.241688   -4.345849  -14.256906
  25 C      6.0000    0    12.011    2.353054   -5.997375   -9.247657
  26 C      6.0000    0    12.011    4.353300   -3.867548  -12.786327
  27 C      6.0000    0    12.011    4.414223   -4.700159  -10.277991
  28 H      1.0000    0     1.008   -1.365700   -7.443831   -9.940068
  29 H      1.0000    0     1.008    2.183530   -3.690401  -16.193782
  30 H      1.0000    0     1.008    2.410414   -6.618800   -7.301606
  31 H      1.0000    0     1.008    5.940636   -2.837243  -13.557639
  32 C      6.0000    0    12.011    6.565620   -4.166836   -8.745345
  33 N      7.0000    0    14.007    8.299452   -3.720900   -7.502962

--------------------------------
INTERNAL COORDINATES (ANGSTROEM)
--------------------------------
 C      0   0   0     0.000000000000     0.00000000     0.00000000
 C      1   0   0     1.394653261291     0.00000000     0.00000000
 C      1   2   0     1.397611350051   118.26114494     0.00000000
 C      2   1   3     1.390733451501   120.96234987     0.21647202
 C      3   1   2     1.390026018252   121.01753552     0.29943262
 C      4   2   1     1.389978095129   120.21140126   359.40689763
 H      2   1   3     1.083506274978   119.28822587   180.19767530
 H      3   1   2     1.083940643446   119.27556465   180.53849685
 H      4   2   1     1.082320386889   119.76177564   179.86766742
 H      5   3   1     1.083267975420   119.84902480   179.83563747
 H      6   4   2     1.082884200123   120.20264271   180.57383972
 C      1   2   3     1.505481094805   121.07120450   179.88607674
 H     12   1   2     1.094439348717   108.61471652   141.76090602
 H     12   1   2     1.091934855126   109.23375123    25.95808585
 C     12   1   2     1.537891845857   115.59494455   263.06727677
 H     15  12   1     1.307762778792   106.49664723    80.39258718
 H     15  12   1     1.090682274288   112.34769151   187.84764428
 C     15  12   1     1.406552386550   121.71483149   328.56897506
 H     18  15  12     1.081442839360   120.27702711   212.37177497
 H     18  15  12     1.081752583230   121.64936579    12.56303387
 Se    16  15  12     2.321159472347   112.66365032   234.27307497
 O     16  15  12     1.325018739007   159.48047844   228.51000734
 C     21  16  15     1.934275654499   102.93023716    91.00525162
 C     23  21  16     1.391596999696   119.66676880    68.09314100
 C     23  21  16     1.392243153105   119.39326223   245.14672640
 C     24  23  21     1.383990343284   119.70108920   176.82935417
 C     25  23  21     1.385017881882   119.71539041   182.94871824
 C     27  25  23     1.398940651624   119.61389327     0.29594056
 H     24  23  21     1.081361796637   120.04577598   356.25422860
 H     25  23  21     1.082484991540   120.51857570     3.74512336
 H     26  24  23     1.081461867432   120.42812765   179.70137899
 H     27  25  23     1.081398544672   120.44710028   181.04381576
 C     28  27  25     1.426026701958   119.71625309   181.22503924
 N     33  28  27     1.153138611864   179.57697803    82.37697667

---------------------------
INTERNAL COORDINATES (A.U.)
---------------------------
 C      0   0   0     0.000000000000     0.00000000     0.00000000
 C      1   0   0     2.635512715621     0.00000000     0.00000000
 C      1   2   0     2.641102693257   118.26114494     0.00000000
 C      2   1   3     2.628105348620   120.96234987     0.21647202
 C      3   1   2     2.626768493520   121.01753552     0.29943262
```

```
 C      4   2   1     2.626677931944   120.21140126   359.40689763
 H      2   1   3     2.047530124094   119.28822587   180.19767530
 H      3   1   2     2.048350961539   119.27556465   180.53849685
 H      4   2   1     2.045289120380   119.76177564   179.86766742
 H      5   3   1     2.047079803192   119.84902480   179.83563747
 H      6   4   2     2.046354572983   120.20264271   180.57383972
 C      1   2   3     2.844946968978   121.07120450   179.88607674
 H     12   1   2     2.068190639263   108.61471652   141.76090602
 H     12   1   2     2.063457832272   109.23375123    25.95808585
 C     12   1   2     2.906194412260   115.59494455   263.06727677
 H     15  12   1     2.471313500052   106.49664723    80.39258718
 H     15  12   1     2.061090797527   112.34769151   187.84764428
 C     15  12   1     2.657998803593   121.71483149   328.56897506
 H     18  15  12     2.043630795880   120.27702711   212.37177497
 H     18  15  12     2.044216126966   121.64936579    12.56303387
 Se    16  15  12     4.386355715893   112.66365032   234.27307497
 O     16  15  12     2.503922539036   159.48047844   228.51000734
 C     21  16  15     3.655251254514   102.93023716    91.00525162
 C     23  21  16     2.629737218211   119.66676880    68.09314100
 C     23  21  16     2.630958271195   119.39326223   245.14672640
 C     24  23  21     2.615362720799   119.70108920   176.82935417
 C     25  23  21     2.617304487341   119.71539041   182.94871824
 C     27  25  23     2.643614709178   119.61389327     0.29594056
 H     24  23  21     2.043477647330   120.04577598   356.25422860
 H     25  23  21     2.045600178091   120.51857570     3.74512336
 H     26  24  23     2.043666753725   120.42812765   179.70137899
 H     27  25  23     2.043547091051   120.44710028   181.04381576
 C     28  27  25     2.694799926360   119.71625309   181.22503924
 N     33  28  27     2.179116170874   179.57697803    82.37697667

---------------------
BASIS SET INFORMATION
---------------------
There are 5 groups of distinct atoms

 Group   1 Type C    : 11s6p2d1f contracted to 5s3p2d1f pattern {62111/411/11/1}
 Group   2 Type H    : 5s1p contracted to 3s1p pattern {311/1}
 Group   3 Type Se   : 17s13p8d1f contracted to 6s5p4d1f pattern {842111/64111/5111/1}
 Group   4 Type O    : 11s6p2d1f contracted to 5s3p2d1f pattern {62111/411/11/1}
 Group   5 Type N    : 11s6p2d1f contracted to 5s3p2d1f pattern {62111/411/11/1}

Atom   0C    basis set group =>   1
Atom   1C    basis set group =>   1
Atom   2C    basis set group =>   1
Atom   3C    basis set group =>   1
Atom   4C    basis set group =>   1
Atom   5C    basis set group =>   1
Atom   6H    basis set group =>   2
Atom   7H    basis set group =>   2
Atom   8H    basis set group =>   2
Atom   9H    basis set group =>   2
Atom  10H    basis set group =>   2
Atom  11C    basis set group =>   1
Atom  12H    basis set group =>   2
Atom  13H    basis set group =>   2
Atom  14C    basis set group =>   1
Atom  15H    basis set group =>   2
Atom  16H    basis set group =>   2
Atom  17C    basis set group =>   1
Atom  18H    basis set group =>   2
Atom  19H    basis set group =>   2
Atom  20Se   basis set group =>   3
Atom  21O    basis set group =>   4
```

```
Atom  22C    basis set group =>   1
Atom  23C    basis set group =>   1
Atom  24C    basis set group =>   1
Atom  25C    basis set group =>   1
Atom  26C    basis set group =>   1
Atom  27C    basis set group =>   1
Atom  28H    basis set group =>   2
Atom  29H    basis set group =>   2
Atom  30H    basis set group =>   2
Atom  31H    basis set group =>   2
Atom  32C    basis set group =>   1
Atom  33N    basis set group =>   5
---------------------------------
AUXILIARY/J BASIS SET INFORMATION
---------------------------------
There are 5 groups of distinct atoms

 Group   1 Type C   : 12s5p4d2f1g contracted to 6s4p3d1f1g pattern {711111/2111/211/2/1}
 Group   2 Type H   : 5s2p1d contracted to 3s1p1d pattern {311/2/1}
 Group   3 Type Se  : 19s5p5d3f1g contracted to 8s4p3d2f1g pattern {121111111/2111/311/21/1}
 Group   4 Type O   : 12s5p4d2f1g contracted to 6s4p3d1f1g pattern {711111/2111/211/2/1}
 Group   5 Type N   : 12s5p4d2f1g contracted to 6s4p3d1f1g pattern {711111/2111/211/2/1}

Atom   0C    basis set group =>   1
Atom   1C    basis set group =>   1
Atom   2C    basis set group =>   1
Atom   3C    basis set group =>   1
Atom   4C    basis set group =>   1
Atom   5C    basis set group =>   1
Atom   6H    basis set group =>   2
Atom   7H    basis set group =>   2
Atom   8H    basis set group =>   2
Atom   9H    basis set group =>   2
Atom  10H    basis set group =>   2
Atom  11C    basis set group =>   1
Atom  12H    basis set group =>   2
Atom  13H    basis set group =>   2
Atom  14C    basis set group =>   1
Atom  15H    basis set group =>   2
Atom  16H    basis set group =>   2
Atom  17C    basis set group =>   1
Atom  18H    basis set group =>   2
Atom  19H    basis set group =>   2
Atom  20Se   basis set group =>   3
Atom  21O    basis set group =>   4
Atom  22C    basis set group =>   1
Atom  23C    basis set group =>   1
Atom  24C    basis set group =>   1
Atom  25C    basis set group =>   1
Atom  26C    basis set group =>   1
Atom  27C    basis set group =>   1
Atom  28H    basis set group =>   2
Atom  29H    basis set group =>   2
Atom  30H    basis set group =>   2
Atom  31H    basis set group =>   2
Atom  32C    basis set group =>   1
Atom  33N    basis set group =>   5


           ************************************************************
           *        Program running with 12 parallel MPI-processes    *
           *              working on a common directory               *
           ************************************************************
```

```
------------------------------------------------------------------------------
                             ORCA STARTUP CALCULATIONS
                           -- RI-GTO INTEGRALS CHOSEN --
------------------------------------------------------------------------------
------------------------------------------------------------------------------
                   ___
                  /   \      - P O W E R E D   B Y -
                 /     \
                 |  |  |   _    _      __       _____    __    __
                 |  |  |  | |  | |    /  \     |  _  \  |  |  /  |
                  \  \/   | |  | |   /    \    | | | |  |  | /  /
                 / \  \   | |__| |  /  /\  \   | |_| |  |  |/  /
                |  |  |   |  __  | /  /__\  \  |    /   |      \
                |  |  |   | |  | | |   __   |  |    \   |  |\   \
                 \    /   | |  | | |  |  |  |  | |\  \  |  | \   \
                  \___/   |_|  |_| |__|  |__|  |_| \__\ |__|  \__/

                      - O R C A' S   B I G   F R I E N D -
                                      &
                       - I N T E G R A L   F E E D E R -

 v1 FN, 2020, v2 2021, v3 2022-2024
------------------------------------------------------------------------------


----------------------
SHARK INTEGRAL PACKAGE
----------------------

Number of atoms                             ...     34
Number of basis functions                   ...    696
Number of shells                            ...    274
Maximum angular momentum                    ...      3
Integral batch strategy                     ... SHARK/LIBINT Hybrid
RI-J (if used) integral strategy            ... SPLIT-RIJ (Revised 2003 algorithm where
possible)
Printlevel                                  ...      1
Contraction scheme used                     ... SEGMENTED contraction
Prescreening option                         ... SCHWARTZ
   Thresh                                   ... 1.000e-12
   Tcut                                     ... 1.000e-14
   Tpresel                                  ... 1.000e-14
Coulomb Range Separation                    ... NOT USED
Exchange Range Separation                   ... NOT USED
Multipole approximations                    ... NOT USED
Finite Nucleus Model                        ... NOT USED
CABS basis                                  ... NOT available
Auxiliary Coulomb fitting basis             ... AVAILABLE
   # of basis functions in Aux-J            ...   1105
   # of shells in Aux-J                     ...    363
   Maximum angular momentum in Aux-J        ...      4
Auxiliary J/K fitting basis                 ... NOT available
Auxiliary Correlation fitting basis         ... NOT available
Auxiliary 'external' fitting basis          ... NOT available

Checking pre-screening integrals            ... done (  0.0 sec) Dimension = 274
   => SHARK Basis and OBASIS are compatible. Storing Pre-screening
Shell pair information
Shell pair cut-off parameter TPreSel        ...   1.0e-14
Total number of shell pairs                 ...     37675
Shell pairs after pre-screening             ...     31268
Total number of primitive shell pairs       ...    120312
Primitive shell pairs kept                  ...     67029
```

```
          la=0 lb=0:   7942 shell pairs
          la=1 lb=0:   8823 shell pairs
          la=1 lb=1:   2493 shell pairs
          la=2 lb=0:   4594 shell pairs
          la=2 lb=1:   2606 shell pairs
          la=2 lb=2:    705 shell pairs
          la=3 lb=0:   2087 shell pairs
          la=3 lb=1:   1219 shell pairs
          la=3 lb=2:    638 shell pairs
          la=3 lb=3:    161 shell pairs

Checking whether 4 symmetric matrices of dimension 696 fit in memory:
Max Core in MB      =   8000.00
 MB in use           =     42.05
 MB left             =   7957.95
 MB needed           =      7.40
 Data fit in memory  = YES
Calculating RI/J V-Matrix + Cholesky decomp.... done (  0.1 sec)
Calculating Nuclear repulsion               ... done (  0.0 sec) ENN=   1824.527754159542 Eh

Diagonalization of the overlap matrix:
Smallest eigenvalue                        ... 6.087e-06
Time for diagonalization                   ...    0.089 sec
Threshold for overlap eigenvalues          ... 1.000e-07
Number of eigenvalues below threshold      ... 0
Time for construction of square roots      ...    0.054 sec
Total time needed                          ...    0.147 sec

-------------------
DFT GRID GENERATION
-------------------

General Integration Accuracy     IntAcc      ... 4.388
Radial Grid Type                 RadialGrid  ... OptM3 with GC (2021)
Angular Grid (max. ang.)         AngularGrid ... 4 (Lebedev-302)
Angular grid pruning method      GridPruning ... 4 (adaptive)
Weight generation scheme         WeightScheme... mBecke (2022)
Basis function cutoff            BFCut       ... 1.0000e-12
Integration weight cutoff        WCut        ... 1.0000e-14
Partially contracted basis set               ... off
Rotationally invariant grid construction     ... off
Angular grids for H and He will be reduced by one unit
Core-polarized basis detected: some atoms will have their core
                               angular grid increased.

Total number of grid points                  ...   166230
Total number of batches                      ...     2617
Average number of points per batch           ...       63
Average number of grid points per atom       ...     4889

--------------------
COSX GRID GENERATION
--------------------

GRIDX 1
-------
General Integration Accuracy     IntAcc      ... 3.816
Radial Grid Type                 RadialGrid  ... OptM3 with GC (2021)
Angular Grid (max. ang.)         AngularGrid ... 1 (Lebedev-50)
Angular grid pruning method      GridPruning ... 4 (adaptive)
Weight generation scheme         WeightScheme... mBecke (2022)
Basis function cutoff            BFCut       ... 1.0000e-12
Integration weight cutoff        WCut        ... 1.0000e-14
```

```
Partially contracted basis set               ... on
Rotationally invariant grid construction     ... off
Angular grids for H and He will be reduced by one unit
Core-polarized basis detected: some atoms will have their core
                               angular grid increased.

Total number of grid points                  ...    25567
Total number of batches                      ...      215
Average number of points per batch           ...      118
Average number of grid points per atom       ...      752
UseSFitting                                  ... on

GRIDX 2
-------
General Integration Accuracy     IntAcc      ... 4.020
Radial Grid Type                 RadialGrid  ... OptM3 with GC (2021)
Angular Grid (max. ang.)         AngularGrid ... 2 (Lebedev-110)
Angular grid pruning method      GridPruning ... 4 (adaptive)
Weight generation scheme         WeightScheme... mBecke (2022)
Basis function cutoff            BFCut       ... 1.0000e-12
Integration weight cutoff        WCut        ... 1.0000e-14
Partially contracted basis set               ... on
Rotationally invariant grid construction     ... off
Angular grids for H and He will be reduced by one unit
Core-polarized basis detected: some atoms will have their core
                               angular grid increased.

Total number of grid points                  ...    48445
Total number of batches                      ...      397
Average number of points per batch           ...      122
Average number of grid points per atom       ...     1425
UseSFitting                                  ... on

GRIDX 3
-------
General Integration Accuracy     IntAcc      ... 4.338
Radial Grid Type                 RadialGrid  ... OptM3 with GC (2021)
Angular Grid (max. ang.)         AngularGrid ... 3 (Lebedev-194)
Angular grid pruning method      GridPruning ... 4 (adaptive)
Weight generation scheme         WeightScheme... mBecke (2022)
Basis function cutoff            BFCut       ... 1.0000e-12
Integration weight cutoff        WCut        ... 1.0000e-14
Partially contracted basis set               ... on
Rotationally invariant grid construction     ... off
Angular grids for H and He will be reduced by one unit
Core-polarized basis detected: some atoms will have their core
                               angular grid increased.

Total number of grid points                  ...    98769
Total number of batches                      ...      789
Average number of points per batch           ...      125
Average number of grid points per atom       ...     2905
UseSFitting                                  ... on
Grids setup in   2.4 sec
Initializing property integral containers    ... done (  0.0 sec)

SHARK setup successfully completed in   3.1 seconds

Maximum memory used throughout the entire STARTUP-calculation: 81.6 MB


           ************************************************************
           *        Program running with 12 parallel MPI-processes    *
```

```
           *              working on a common directory              *
           ***********************************************************
--------------------------------------------------------------------------------
                              ORCA GUESS
                  Start orbitals & Density for SCF / CASSCF
--------------------------------------------------------------------------------


------------
SCF SETTINGS
------------
Hamiltonian:
 Density Functional     Method          .... DFT(GTOs)
 Exchange Functional    Exchange        .... B88
   X-Alpha parameter    XAlpha          ....  0.666667
   Becke's b parameter  XBeta           ....  0.004200
 Correlation Functional Correlation     .... LYP
 LDA part of GGA corr.  LDAOpt          .... VWN-5
 Gradients option       PostSCFGGA      .... off
 Hybrid DFT is turned on
   Fraction HF Exchange ScalHFX         ....  0.200000
   Scaling of DF-GGA-X  ScalDFX         ....  0.720000
   Scaling of DF-GGA-C  ScalDFC         ....  0.810000
   Scaling of DF-LDA-C  ScalLDAC        ....  1.000000
   Perturbative correction              ....  0.000000
   NL short-range parameter             ....  4.800000
 RI-approximation to the Coulomb term is turned on
   Number of AuxJ basis functions       .... 1105
   RIJ-COSX (HFX calculated with COS-X)).... on


General Settings:
 Integral files         IntName         .... CN-IRC
 Hartree-Fock type      HFTyp           .... RHF
 Total Charge           Charge          ....    0
 Multiplicity           Mult            ....    1
 Number of Electrons    NEL             ....  160
 Basis Dimension        Dim             ....  696
 Nuclear Repulsion      ENuc            ....   1824.5277541595 Eh

Convergence Acceleration:
 AO-DIIS                CNVDIIS         .... on
   Start iteration      DIISMaxIt       ....    12
   Startup error        DIISStart       ....  0.200000
   # of expansion vecs  DIISMaxEq       ....     5
   Bias factor          DIISBfac        ....   1.050
   Max. coefficient     DIISMaxC        ....  10.000
 MO-DIIS                CNVKDIIS        .... off
 Trust-Rad. Augm. Hess. CNVTRAH         .... auto
   Auto Start mean grad. ratio tolernc. ....  1.125000
   Auto Start start iteration           ....    50
   Auto Start num. interpolation iter.  ....    10
   Max. Number of Micro iterations      ....    24
   Max. Number of Macro iterations      .... Maxiter - #DIIS iter
   Number of Davidson start vectors     ....     2
   Converg. threshold    (grad. norm)   ....   2.000e-06
   Grad. Scal. Fac. for Micro threshold ....   0.100
   Minimum threshold for Micro iter.    ....   1.000e-02
   NR start threshold (gradient norm)   ....   1.000e-04
   Initial trust radius                 ....   0.400
   Minimum AH scaling param. (alpha)    ....   1.000
   Maximum AH scaling param. (alpha)    .... 1000.000
   Quad. conv. algorithm                .... NR
   White noise on init. David. guess    .... on
```

```
   Maximum white noise                  ....   0.010
   Pseudo random numbers                .... off
   Inactive MOs                         .... canonical
   Orbital update algorithm             .... Taylor
   Preconditioner                       .... Diag
   Full preconditioner red. dimension   ....   250
 SOSCF                 CNVSOSCF           .... on
   Start iteration     SOSCFMaxIt         ....   150
   Startup grad/error  SOSCFStart         ....  0.003300
   Hessian update      SOSCFHessUp        .... L-BFGS
   Autom. constraints  SOSCFAutoConstrain .... off
 Level Shifting         CNVShift        .... on
   Level shift para.    LevelShift      ....    0.2500
   Turn off err/grad.   ShiftErr        ....    0.0010
 Zerner damping         CNVZerner       .... off
 Static damping         CNVDamp         .... on
   Fraction old density DampFac         ....    0.7000
   Max. Damping (<1)    DampMax         ....    0.9800
   Min. Damping (>=0)   DampMin         ....    0.0000
   Turn off err/grad.   DampErr         ....    0.1000

SCF Procedure:
 Maximum # iterations   MaxIter         ....   125
 SCF integral mode      SCFMode         .... Direct
   Integral package                     .... SHARK and LIBINT hybrid scheme
 Reset frequency        DirectResetFreq ....    20
 Integral Threshold     Thresh          ....  1.000e-12 Eh
 Primitive CutOff       TCut            ....  1.000e-14 Eh

Convergence Tolerance:
 Convergence Check Mode ConvCheckMode   .... Total+1el-Energy
 Convergence forced     ConvForced      .... 0
 Energy Change          TolE            ....  1.000e-09 Eh
 1-El. energy change                    ....  1.000e-06 Eh
 Orbital Gradient       TolG            ....  2.000e-06
 Orbital Rotation angle TolX            ....  2.000e-06
 DIIS Error             TolErr          ....  1.000e-08

------------------------------
INITIAL GUESS: MODEL POTENTIAL
------------------------------
Loading Hartree-Fock densities                     ... done
Calculating cut-offs                               ... done
Initializing the effective Hamiltonian             ... done
Setting up the integral package (SHARK)            ... done
Starting the Coulomb interaction                   ... done (   0.4 sec)
Making the grid                                    ... done (   0.3 sec)
Mapping shells                                     ... done
Starting the XC term evaluation                    ... done (   0.3 sec)
  promolecular density results
     # of electrons  =    159.999629354
     EX              =   -187.042573546
     EC              =     -5.890097961
     EX+EC           =   -192.932671507
Transforming the Hamiltonian                       ... done (   0.1 sec)
Diagonalizing the Hamiltonian                      ... done (   0.1 sec)
Back transforming the eigenvectors                 ... done (   0.0 sec)
Now organizing SCF variables                       ... done
                      ------------------
                      INITIAL GUESS DONE (   1.2 sec)
                      ------------------
             **** ENERGY FILE WAS UPDATED (CN-IRC.en.tmp) ****
Finished Guess after   1.5 sec
```

```
Maximum memory used throughout the entire GUESS-calculation: 48.3 MB


           ************************************************************
           *        Program running with 12 parallel MPI-processes    *
           *              working on a common directory               *
           ************************************************************

------------------------------------------------------------------------------------------
                                     ORCA LEAN-SCF
                             memory conserving SCF solver
------------------------------------------------------------------------------------------

--------------------
CPCM SOLVATION MODEL
--------------------
CPCM parameters:
  Epsilon                                         ...       7.4257
  Refrac                                          ...       1.4050
  Rsolv                                           ...       1.3000
  Surface type                                    ... GAUSSIAN VDW
  Discretization scheme                           ... Constant charge density
    Threshold for H atoms                         ...       5.0000 (charges/Ang^2)
    Threshold for non-H atoms                     ...       5.0000 (charges/Ang^2)
  Epsilon function type                           ...         CPCM
Solvent:                                          ... TETRAHYDROFURAN
SMD-CDS solvent descriptors:
  Soln                                            ...       1.4050
  Soln25                                          ...       1.4044
  Sola                                            ...       0.0000
  Solb                                            ...       0.4800
  Solg                                            ...      39.4400
  Solc                                            ...       0.0000
  Solh                                            ...       0.0000
Radii:
  Scheme                                          ... Element-dependent radii
  Radius for C  used is    3.4960 Bohr (=   1.8500 Ang.)
  Radius for H  used is    2.2677 Bohr (=   1.2000 Ang.)
  Radius for Se used is    3.5905 Bohr (=   1.9000 Ang.)
  Radius for O  used is    4.3350 Bohr (=   2.2940 Ang.)
  Radius for N  used is    3.5716 Bohr (=   1.8900 Ang.)
Calculating surface                               ...        done! (  0.0s)
Cavity surface points                             ...         2729
Cavity Volume                                     ...    2036.9493
Cavity Surface-area                               ...    1125.1561
Calculating surface distance matrix               ...        done! (  0.1s)
Performing Cholesky decomposition & store         ...        done! (  0.2s)
Overall time for CPCM initialization              ...                 0.3s

----------------------------------------D-I-I-S-------------------------------------------
Iteration    Energy (Eh)           Delta-E    RMSDP     MaxDP     DIISErr   Damp  Time(sec)
------------------------------------------------------------------------------------------
               ***  Starting incremental Fock matrix formation  ***
    1    -3149.2476203518131115     0.00e+00  5.89e-03  1.93e-01  3.12e-01  0.700  10.0
    2    -3149.5596260885854463    -3.12e-01  4.38e-03  1.45e-01  1.10e-01  0.700   6.3
                               ***Turning on AO-DIIS***
    3    -3149.6724831915003051    -1.13e-01  1.54e-03  3.05e-02  4.38e-02  0.700   6.0
    4    -3149.7425524980531009    -7.01e-02  2.83e-03  9.15e-02  2.03e-02  0.000   5.8
    5    -3149.8980855226641324    -1.56e-01  9.58e-04  2.39e-02  8.12e-03  0.000   6.0
    6    -3149.8994196934422689    -1.33e-03  3.91e-04  8.88e-03  4.08e-03  0.000   5.6
    7    -3149.8995218272598322    -1.02e-04  2.36e-04  8.84e-03  4.22e-03  0.000   5.3
                              *** Initializing SOSCF ***
--------------------------------------S-O-S-C-F-----------------------------------
```

```
Iteration    Energy (Eh)           Delta-E    RMSDP     MaxDP     MaxGrad    Time(sec)
-----------------------------------------------------------------------------------------
    8    -3149.8995519216446155    -3.01e-05  7.14e-05  1.18e-03  2.04e-03     5.3
               *** Restarting incremental Fock matrix formation ***
    9    -3149.8995765990862310    -2.47e-05  7.86e-05  9.72e-04  2.20e-04     9.8
   10    -3149.8995772728517295    -6.74e-07  4.38e-05  8.60e-04  2.66e-04     7.5
   11    -3149.8995780254458623    -7.53e-07  3.02e-05  2.85e-04  1.27e-04     7.2
   12    -3149.8995781501912461    -1.25e-07  1.10e-05  2.62e-04  5.35e-05     7.1
   13    -3149.8995782501356189    -9.99e-08  1.12e-05  1.47e-04  1.65e-05     6.7
   14    -3149.8995782732349653    -2.31e-08  1.80e-06  3.80e-05  1.19e-05     6.8
   15    -3149.8995782775341468    -4.30e-09  2.25e-06  6.26e-05  3.58e-06     5.8
   16    -3149.8995782785791562    -1.05e-09  6.98e-07  1.51e-05  2.72e-06     5.8
   17    -3149.8995782784763833     1.03e-10  6.91e-07  7.91e-06  1.91e-06     5.1
                 **** Energy Check signals convergence ****

               *****************************************************
               *                     SUCCESS                       *
               *           SCF CONVERGED AFTER  17 CYCLES          *
               *****************************************************

Recomputing exchange energy using gridx3       ... done (   14.081 sec)
Old exchange energy                            :    -37.699504310 Eh
New exchange energy                            :    -37.699506943 Eh
Exchange energy change after final integration :     -0.000002633 Eh
Total energy after final integration           :  -3149.899580912 Eh

SMD CDS free energy correction energy :               -2.64080     Kcal/mol
Total Energy after SMD CDS correction =            -3149.903789296 Eh
             **** ENERGY FILE WAS UPDATED (CN-IRC.en.tmp) ****

----------------
TOTAL SCF ENERGY
----------------

Total Energy       :      -3149.90378929618055 Eh          -85713.23968 eV

Components:
Nuclear Repulsion  :       1824.52775415954193 Eh           49647.92424 eV
Electronic Energy  :      -4974.40422521227902 Eh         -135360.42056 eV
One Electron Energy:      -7985.56344010091470 Eh         -217298.22843 eV
Two Electron Energy:       3011.15921488863569 Eh           81937.80787 eV
CPCM Dielectric    :         -0.02310722582782 Eh              -0.62878 eV
SMD CDS (Gcds)     :         -0.00420838467187 Eh              -0.11452 eV

Virial components:
Potential Energy   :      -6292.84361538325538 Eh         -171236.98029 eV
Kinetic Energy     :       3142.93982608707529 Eh           85523.74061 eV
Virial Ratio       :          2.00221574818306

DFT components:
N(Alpha)           :       79.999937615341 electrons
N(Beta)            :       79.999937615341 electrons
N(Total)           :      159.999875230682 electrons
E(X)               :     -149.983905956950 Eh
E(C)               :       -6.928113053129 Eh
E(XC)              :     -156.912019010079 Eh

CPCM Solvation Model Properties:
Surface-charge          :         -0.05584171583370
Corrected charge        :         -0.00000000000000
Outlying charge corr.   :          0.00015799733768 Eh               0.00430 eV
Free-energy (cav+disp)  :         -0.00420838467187 Eh              -0.11452 eV
```

```
---------------
SCF CONVERGENCE
---------------

  Last Energy change         ...   -1.0277e-10  Tolerance :   1.0000e-09
  Last MAX-Density change    ...    7.9083e-06  Tolerance :   1.0000e-08
  Last RMS-Density change    ...    6.9095e-07  Tolerance :   1.0000e-09
  Last DIIS Error            ...    2.0371e-03  Tolerance :   1.0000e-08
  Last Orbital Gradient      ...    1.9060e-06  Tolerance :   2.0000e-06
  Last Orbital Rotation      ...    4.7313e-06  Tolerance :   2.0000e-06


----------------
ORBITAL ENERGIES
----------------

  NO   OCC          E(Eh)            E(eV) 
   0   2.0000    -453.934529    -12352.1865 
   1   2.0000     -58.114845     -1581.3853 
   2   2.0000     -52.167289     -1419.5441 
   3   2.0000     -52.166541     -1419.5238 
   4   2.0000     -52.165441     -1419.4938 
   5   2.0000     -19.114641      -520.1358 
   6   2.0000     -14.320330      -389.6760 
   7   2.0000     -10.220517      -278.1144 
   8   2.0000     -10.218946      -278.0716 
   9   2.0000     -10.212017      -277.8831 
  10   2.0000     -10.196834      -277.4699 
  11   2.0000     -10.195672      -277.4383 
  12   2.0000     -10.191155      -277.3154 
  13   2.0000     -10.189772      -277.2778 
  14   2.0000     -10.188240      -277.2361 
  15   2.0000     -10.175050      -276.8772 
  16   2.0000     -10.167694      -276.6770 
  17   2.0000     -10.167225      -276.6643 
  18   2.0000     -10.166777      -276.6521 
  19   2.0000     -10.165744      -276.6239 
  20   2.0000     -10.165462      -276.6163 
  21   2.0000     -10.164759      -276.5972 
  22   2.0000     -10.158017      -276.4137 
  23   2.0000      -7.853173      -213.6957 
  24   2.0000      -5.797100      -157.7471 
  25   2.0000      -5.793312      -157.6440 
  26   2.0000      -5.789465      -157.5393 
  27   2.0000      -2.150633       -58.5217 
  28   2.0000      -2.149916       -58.5022 
  29   2.0000      -2.146096       -58.3982 
  30   2.0000      -2.142831       -58.3094 
  31   2.0000      -2.142460       -58.2993 
  32   2.0000      -0.967444       -26.3255 
  33   2.0000      -0.920974       -25.0610 
  34   2.0000      -0.880716       -23.9655 
  35   2.0000      -0.856300       -23.3011 
  36   2.0000      -0.796201       -21.6657 
  37   2.0000      -0.790887       -21.5211 
  38   2.0000      -0.768313       -20.9069 
  39   2.0000      -0.746059       -20.3013 
  40   2.0000      -0.741085       -20.1660 
  41   2.0000      -0.691900       -18.8276 
  42   2.0000      -0.675303       -18.3759 
  43   2.0000      -0.628705       -17.1079 
  44   2.0000      -0.617494       -16.8029 
  45   2.0000      -0.601374       -16.3642 
```

```
  46   2.0000      -0.591383       -16.0923
  47   2.0000      -0.562594       -15.3090
  48   2.0000      -0.556615       -15.1463
  49   2.0000      -0.504678       -13.7330
  50   2.0000      -0.485636       -13.2148
  51   2.0000      -0.474542       -12.9129
  52   2.0000      -0.466841       -12.7034
  53   2.0000      -0.465390       -12.6639
  54   2.0000      -0.451910       -12.2971
  55   2.0000      -0.446247       -12.1430
  56   2.0000      -0.442988       -12.0543
  57   2.0000      -0.437781       -11.9126
  58   2.0000      -0.419423       -11.4131
  59   2.0000      -0.415857       -11.3160
  60   2.0000      -0.411144       -11.1878
  61   2.0000      -0.391387       -10.6502
  62   2.0000      -0.388386       -10.5685
  63   2.0000      -0.379271       -10.3205
  64   2.0000      -0.374953       -10.2030
  65   2.0000      -0.371865       -10.1190
  66   2.0000      -0.368030       -10.0146
  67   2.0000      -0.365103        -9.9350
  68   2.0000      -0.351894        -9.5755
  69   2.0000      -0.348456        -9.4820
  70   2.0000      -0.344444        -9.3728
  71   2.0000      -0.333794        -9.0830
  72   2.0000      -0.327048        -8.8994
  73   2.0000      -0.285825        -7.7777
  74   2.0000      -0.278473        -7.5776
  75   2.0000      -0.273636        -7.4460
  76   2.0000      -0.253873        -6.9082
  77   2.0000      -0.247844        -6.7442
  78   2.0000      -0.243239        -6.6189
  79   2.0000      -0.233052        -6.3417
  80   0.0000      -0.064773        -1.7626
  81   0.0000      -0.037748        -1.0272
  82   0.0000      -0.029480        -0.8022
  83   0.0000      -0.007337        -0.1996
  84   0.0000      -0.005936        -0.1615
  85   0.0000       0.001777         0.0483
  86   0.0000       0.025948         0.7061
  87   0.0000       0.028321         0.7706
  88   0.0000       0.043841         1.1930
  89   0.0000       0.058789         1.5997
  90   0.0000       0.062041         1.6882
*Only the first 10 virtual orbitals were printed (use %output print[p_orben] 2 end to see all).

                    ********************************
                    * MULLIKEN POPULATION ANALYSIS *
                    ********************************

-----------------------
MULLIKEN ATOMIC CHARGES
-----------------------
   0 C :    0.177120
   1 C :   -0.233850
   2 C :   -0.244771
   3 C :   -0.100162
   4 C :   -0.129075
   5 C :   -0.172414
   6 H :    0.126664
   7 H :    0.139005
   8 H :    0.139268
```

```
   9 H :    0.137473
  10 H :    0.141217
  11 C :   -0.177856
  12 H :    0.114709
  13 H :    0.117944
  14 C :   -0.266660
  15 H :    0.257482
  16 H :    0.130672
  17 C :   -0.295685
  18 H :    0.165398
  19 H :    0.168060
  20 Se:    0.435461
  21 O :   -0.571080
  22 C :    0.066202
  23 C :   -0.148434
  24 C :   -0.175853
  25 C :   -0.238872
  26 C :   -0.219603
  27 C :    0.277404
  28 H :    0.146801
  29 H :    0.157688
  30 H :    0.156224
  31 H :    0.157538
  32 C :   -0.177270
  33 N :   -0.060744
Sum of atomic charges:    0.0000000

--------------------------------
MULLIKEN REDUCED ORBITAL CHARGES
--------------------------------
  0 C s       :     3.164824  s :     3.164824
      pz      :     0.877349  p :     2.524070
      px      :     0.777995
      py      :     0.868726
      dz2     :     0.025147  d :     0.124170
      dxz     :     0.029255
      dyz     :     0.019602
      dx2y2   :     0.023802
      dxy     :     0.026363
      f0      :     0.001237  f :     0.009817
      f+1     :     0.001420
      f-1     :     0.001216
      f+2     :     0.001950
      f-2     :     0.001347
      f+3     :     0.001117
      f-3     :     0.001529

  1 C s       :     3.259599  s :     3.259599
      pz      :     0.969665  p :     2.883788
      px      :     0.975726
      py      :     0.938398
      dz2     :     0.017647  d :     0.083150
      dxz     :     0.021471
      dyz     :     0.011647
      dx2y2   :     0.012570
      dxy     :     0.019814
      f0      :     0.001172  f :     0.007313
      f+1     :     0.001109
      f-1     :     0.000611
      f+2     :     0.001271
      f-2     :     0.001316
      f+3     :     0.000973
      f-3     :     0.000861
```

```
2 C s       :     3.272695  s :     3.272695
    pz      :     0.969480  p :     2.882582
    px      :     0.947844
    py      :     0.965258
    dz2     :     0.013511  d :     0.082215
    dxz     :     0.019019
    dyz     :     0.010999
    dx2y2   :     0.018745
    dxy     :     0.019940
    f0      :     0.000327  f :     0.007279
    f+1     :     0.001311
    f-1     :     0.001020
    f+2     :     0.001114
    f-2     :     0.001157
    f+3     :     0.001053
    f-3     :     0.001298

3 C s       :     3.190479  s :     3.190479
    pz      :     0.949938  p :     2.819399
    px      :     0.921438
    py      :     0.948024
    dz2     :     0.013425  d :     0.083010
    dxz     :     0.019064
    dyz     :     0.010302
    dx2y2   :     0.020057
    dxy     :     0.020162
    f0      :     0.000333  f :     0.007274
    f+1     :     0.001318
    f-1     :     0.001061
    f+2     :     0.001146
    f-2     :     0.001147
    f+3     :     0.001009
    f-3     :     0.001261

4 C s       :     3.214129  s :     3.214129
    pz      :     0.943868  p :     2.825141
    px      :     0.926574
    py      :     0.954700
    dz2     :     0.017303  d :     0.082540
    dxz     :     0.021659
    dyz     :     0.011372
    dx2y2   :     0.012498
    dxy     :     0.019707
    f0      :     0.001201  f :     0.007265
    f+1     :     0.001061
    f-1     :     0.000612
    f+2     :     0.001238
    f-2     :     0.001314
    f+3     :     0.000956
    f-3     :     0.000883

5 C s       :     3.234457  s :     3.234457
    pz      :     0.938293  p :     2.849868
    px      :     0.969772
    py      :     0.941804
    dz2     :     0.020662  d :     0.080869
    dxz     :     0.015495
    dyz     :     0.015453
    dx2y2   :     0.018707
    dxy     :     0.010553
    f0      :     0.001064  f :     0.007220
    f+1     :     0.000862
```

```
      f-1     :     0.001085
      f+2     :     0.001468
      f-2     :     0.001082
      f+3     :     0.000810
      f-3     :     0.000850

  6 H s       :     0.851056  s :     0.851056
      pz      :     0.006380  p :     0.022280
      px      :     0.006895
      py      :     0.009005

  7 H s       :     0.838904  s :     0.838904
      pz      :     0.010445  p :     0.022091
      px      :     0.005071
      py      :     0.006576

  8 H s       :     0.838403  s :     0.838403
      pz      :     0.010785  p :     0.022329
      px      :     0.005065
      py      :     0.006479

  9 H s       :     0.840356  s :     0.840356
      pz      :     0.006320  p :     0.022171
      px      :     0.006877
      py      :     0.008975

 10 H s       :     0.836526  s :     0.836526
      pz      :     0.005111  p :     0.022257
      px      :     0.012506
      py      :     0.004640

 11 C s       :     3.231163  s :     3.231163
      pz      :     0.969863  p :     2.854516
      px      :     0.911425
      py      :     0.973228
      dz2     :     0.018752  d :     0.086440
      dxz     :     0.023362
      dyz     :     0.011428
      dx2y2   :     0.014815
      dxy     :     0.018082
      f0      :     0.000608  f :     0.005738
      f+1     :     0.000807
      f-1     :     0.000845
      f+2     :     0.001147
      f-2     :     0.000988
      f+3     :     0.000780
      f-3     :     0.000563

 12 H s       :     0.863595  s :     0.863595
      pz      :     0.005008  p :     0.021696
      px      :     0.006181
      py      :     0.010507

 13 H s       :     0.860225  s :     0.860225
      pz      :     0.009373  p :     0.021831
      px      :     0.007255
      py      :     0.005203

 14 C s       :     3.202606  s :     3.202606
      pz      :     0.978763  p :     2.962956
      px      :     0.948290
      py      :     1.035903
      dz2     :     0.027543  d :     0.094757
```

```
     dxz     :    0.014979
     dyz     :    0.017332
     dx2y2   :    0.022107
     dxy     :    0.012796
     f0      :    0.001145  f :     0.006340
     f+1     :    0.000754
     f-1     :    0.000695
     f+2     :    0.001622
     f-2     :    0.001064
     f+3     :    0.000386
     f-3     :    0.000674

15 H s       :    0.690786  s :     0.690786
     pz      :    0.008479  p :     0.051732
     px      :    0.007287
     py      :    0.035966

16 H s       :    0.846654  s :     0.846654
     pz      :    0.005648  p :     0.022674
     px      :    0.011760
     py      :    0.005266

17 C s       :    3.303644  s :     3.303644
     pz      :    1.010858  p :     2.916188
     px      :    1.000800
     py      :    0.904530
     dz2     :    0.023689  d :     0.070911
     dxz     :    0.006310
     dyz     :    0.010783
     dx2y2   :    0.013437
     dxy     :    0.016693
     f0      :    0.000725  f :     0.004942
     f+1     :    0.001168
     f-1     :    0.000596
     f+2     :    0.000367
     f-2     :    0.000943
     f+3     :    0.000376
     f-3     :    0.000766

18 H s       :    0.812280  s :     0.812280
     pz      :    0.012714  p :     0.022322
     px      :    0.004324
     py      :    0.005284

19 H s       :    0.809087  s :     0.809087
     pz      :    0.004177  p :     0.022853
     px      :    0.011914
     py      :    0.006762

20 Ses       :    7.842699  s :     7.842699
     pz      :    5.110841  p :    15.450708
     px      :    4.782896
     py      :    5.556970
     dz2     :    2.048760  d :    10.230451
     dxz     :    2.051377
     dyz     :    2.034960
     dx2y2   :    2.057410
     dxy     :    2.037944
     f0      :    0.005716  f :     0.040682
     f+1     :    0.010394
     f-1     :    0.001707
     f+2     :    0.007161
     f-2     :    0.007914
```

```
     f+3     :      0.003747
     f-3     :      0.004043

21 O s       :      3.868706  s :     3.868706
     pz      :      1.584391  p :     4.675440
     px      :      1.477910
     py      :      1.613139
     dz2     :      0.005351  d :     0.025964
     dxz     :      0.006931
     dyz     :      0.002941
     dx2y2   :      0.006044
     dxy     :      0.004697
     f0      :      0.000101  f :     0.000970
     f+1     :      0.000233
     f-1     :      0.000060
     f+2     :      0.000213
     f-2     :      0.000153
     f+3     :      0.000116
     f-3     :      0.000095

22 C s       :      3.168737  s :     3.168737
     pz      :      0.829280  p :     2.630161
     px      :      0.900638
     py      :      0.900243
     dz2     :      0.022578  d :     0.126617
     dxz     :      0.042242
     dyz     :      0.025596
     dx2y2   :      0.019341
     dxy     :      0.016859
     f0      :      0.001238  f :     0.008282
     f+1     :      0.001871
     f-1     :      0.001079
     f+2     :      0.001010
     f-2     :      0.001091
     f+3     :      0.001300
     f-3     :      0.000693

23 C s       :      3.249543  s :     3.249543
     pz      :      0.920587  p :     2.802573
     px      :      0.911943
     py      :      0.970043
     dz2     :      0.025255  d :     0.088806
     dxz     :      0.024365
     dyz     :      0.016413
     dx2y2   :      0.008505
     dxy     :      0.014267
     f0      :      0.001280  f :     0.007513
     f+1     :      0.001866
     f-1     :      0.000898
     f+2     :      0.000741
     f-2     :      0.001203
     f+3     :      0.000654
     f-3     :      0.000870

24 C s       :      3.254908  s :     3.254908
     pz      :      0.956097  p :     2.828000
     px      :      0.913859
     py      :      0.958044
     dz2     :      0.024394  d :     0.085507
     dxz     :      0.015752
     dyz     :      0.009338
     dx2y2   :      0.018025
     dxy     :      0.017996
```

```
     f0      :     0.000792  f :     0.007439
     f+1     :     0.001570
     f-1     :     0.000743
     f+2     :     0.000883
     f-2     :     0.001192
     f+3     :     0.001387
     f-3     :     0.000872

25 C s       :     3.340054  s :     3.340054
     pz      :     0.969490  p :     2.809335
     px      :     0.898935
     py      :     0.940911
     dz2     :     0.020401  d :     0.082075
     dxz     :     0.016917
     dyz     :     0.009033
     dx2y2   :     0.017720
     dxy     :     0.018004
     f0      :     0.000845  f :     0.007409
     f+1     :     0.001518
     f-1     :     0.000694
     f+2     :     0.000877
     f-2     :     0.001169
     f+3     :     0.001426
     f-3     :     0.000879

26 C s       :     3.310433  s :     3.310433
     pz      :     0.915294  p :     2.819617
     px      :     0.971943
     py      :     0.932379
     dz2     :     0.023837  d :     0.082131
     dxz     :     0.018427
     dyz     :     0.017729
     dx2y2   :     0.008503
     dxy     :     0.013634
     f0      :     0.001305  f :     0.007423
     f+1     :     0.001800
     f-1     :     0.000885
     f+2     :     0.000722
     f-2     :     0.001180
     f+3     :     0.000678
     f-3     :     0.000852

27 C s       :     3.116852  s :     3.116852
     pz      :     0.792593  p :     2.496007
     px      :     0.742534
     py      :     0.960880
     dz2     :     0.023391  d :     0.100846
     dxz     :     0.024820
     dyz     :     0.019272
     dx2y2   :     0.017826
     dxy     :     0.015537
     f0      :     0.001404  f :     0.008891
     f+1     :     0.001982
     f-1     :     0.001117
     f+2     :     0.001184
     f-2     :     0.001136
     f+3     :     0.001371
     f-3     :     0.000697

28 H s       :     0.830461  s :     0.830461
     pz      :     0.005120  p :     0.022738
     px      :     0.010843
     py      :     0.006774
```

```
 29 H s       :     0.819900  s :     0.819900
      pz      :     0.013331  p :     0.022411
      px      :     0.003676
      py      :     0.005404

 30 H s       :     0.821364  s :     0.821364
      pz      :     0.013557  p :     0.022412
      px      :     0.003610
      py      :     0.005245

 31 H s       :     0.820033  s :     0.820033
      pz      :     0.005168  p :     0.022429
      px      :     0.010289
      py      :     0.006971

 32 C s       :     3.193900  s :     3.193900
      pz      :     0.962164  p :     2.879651
      px      :     1.011147
      py      :     0.906340
      dz2     :     0.023041  d :     0.086939
      dxz     :     0.010275
      dyz     :     0.014954
      dx2y2   :     0.015128
      dxy     :     0.023541
      f0      :     0.001233  f :     0.016780
      f+1     :     0.005081
      f-1     :     0.000503
      f+2     :     0.002249
      f-2     :     0.003242
      f+3     :     0.002213
      f-3     :     0.002259

 33 N s       :     3.585937  s :     3.585937
      pz      :     1.135720  p :     3.438039
      px      :     1.191634
      py      :     1.110685
      dz2     :     0.009842  d :     0.034976
      dxz     :     0.005354
      dyz     :     0.004758
      dx2y2   :     0.006216
      dxy     :     0.008806
      f0      :     0.000093  f :     0.001792
      f+1     :     0.000651
      f-1     :     0.000061
      f+2     :     0.000112
      f-2     :     0.000368
      f+3     :     0.000245
      f-3     :     0.000263


                     *******************************
                     * LOEWDIN POPULATION ANALYSIS *
                     *******************************

----------------------
LOEWDIN ATOMIC CHARGES
----------------------
   0 C :   -0.148287
   1 C :   -0.115183
   2 C :   -0.123243
   3 C :   -0.141203
```

```
  4 C :   -0.152094
  5 C :   -0.153018
  6 H :    0.151082
  7 H :    0.152006
  8 H :    0.149260
  9 H :    0.148107
 10 H :    0.148251
 11 C :   -0.208697
 12 H :    0.149175
 13 H :    0.146549
 14 C :   -0.309381
 15 H :    0.190080
 16 H :    0.146125
 17 C :   -0.221376
 18 H :    0.127089
 19 H :    0.129683
 20 Se:    0.661039
 21 O :   -0.407221
 22 C :   -0.302364
 23 C :   -0.122016
 24 C :   -0.124906
 25 C :   -0.066968
 26 C :   -0.066572
 27 C :   -0.148119
 28 H :    0.156189
 29 H :    0.159135
 30 H :    0.164063
 31 H :    0.164261
 32 C :   -0.141972
 33 N :    0.010527

-------------------------------
LOEWDIN REDUCED ORBITAL CHARGES
-------------------------------
  0 C s       :     2.710884  s :     2.710884
      pz      :     0.965423  p :     2.860668
      px      :     0.963411
      py      :     0.931834
      dz2     :     0.102803  d :     0.518991
      dxz     :     0.118682
      dyz     :     0.086220
      dx2y2   :     0.109517
      dxy     :     0.101769
      f0      :     0.006521  f :     0.057744
      f+1     :     0.009873
      f-1     :     0.007576
      f+2     :     0.010544
      f-2     :     0.007090
      f+3     :     0.006461
      f-3     :     0.009680

  1 C s       :     2.759593  s :     2.759593
      pz      :     0.977282  p :     2.928829
      px      :     1.014896
      py      :     0.936652
      dz2     :     0.070328  d :     0.383976
      dxz     :     0.108202
      dyz     :     0.057800
      dx2y2   :     0.061376
      dxy     :     0.086270
      f0      :     0.006279  f :     0.042786
      f+1     :     0.007064
      f-1     :     0.004896
```

```
     f+2     :      0.007830
     f-2     :      0.005045
     f+3     :      0.005497
     f-3     :      0.006174

 2 C s       :      2.760235  s :      2.760235
     pz      :      0.954092  p :      2.936054
     px      :      1.027019
     py      :      0.954943
     dz2     :      0.059831  d :      0.384330
     dxz     :      0.089082
     dyz     :      0.055833
     dx2y2   :      0.085716
     dxy     :      0.093868
     f0      :      0.002752  f :      0.042623
     f+1     :      0.008048
     f-1     :      0.004934
     f+2     :      0.007368
     f-2     :      0.005307
     f+3     :      0.005287
     f-3     :      0.008926

 3 C s       :      2.765591  s :      2.765591
     pz      :      0.955869  p :      2.943508
     px      :      1.033978
     py      :      0.953661
     dz2     :      0.062256  d :      0.388635
     dxz     :      0.089532
     dyz     :      0.055162
     dx2y2   :      0.087092
     dxy     :      0.094594
     f0      :      0.002751  f :      0.043469
     f+1     :      0.008225
     f-1     :      0.005217
     f+2     :      0.007568
     f-2     :      0.005433
     f+3     :      0.005295
     f-3     :      0.008980

 4 C s       :      2.766365  s :      2.766365
     pz      :      0.990171  p :      2.953475
     px      :      1.021896
     py      :      0.941409
     dz2     :      0.073040  d :      0.388971
     dxz     :      0.109396
     dyz     :      0.058466
     dx2y2   :      0.060312
     dxy     :      0.087758
     f0      :      0.006548  f :      0.043283
     f+1     :      0.006995
     f-1     :      0.005018
     f+2     :      0.007922
     f-2     :      0.005043
     f+3     :      0.005537
     f-3     :      0.006220

 5 C s       :      2.766677  s :      2.766677
     pz      :      1.000925  p :      2.956523
     px      :      0.981124
     py      :      0.974474
     dz2     :      0.087732  d :      0.386392
     dxz     :      0.079536
     dyz     :      0.077628
```

```
      dx2y2   :     0.086280
      dxy     :     0.055216
      f0      :     0.005549  f :     0.043425
      f+1     :     0.006676
      f-1     :     0.006856
      f+2     :     0.007767
      f-2     :     0.006048
      f+3     :     0.004587
      f-3     :     0.005943

  6 H s       :     0.789641  s :     0.789641
      pz      :     0.017136  p :     0.059278
      px      :     0.018425
      py      :     0.023717

  7 H s       :     0.788798  s :     0.788798
      pz      :     0.027164  p :     0.059196
      px      :     0.014244
      py      :     0.017788

  8 H s       :     0.791471  s :     0.791471
      pz      :     0.027290  p :     0.059269
      px      :     0.014315
      py      :     0.017664

  9 H s       :     0.792818  s :     0.792818
      pz      :     0.017203  p :     0.059075
      px      :     0.018472
      py      :     0.023399

 10 H s       :     0.792359  s :     0.792359
      pz      :     0.014360  p :     0.059390
      px      :     0.031751
      py      :     0.013280

 11 C s       :     2.765941  s :     2.765941
      pz      :     1.009362  p :     3.016657
      px      :     0.995698
      py      :     1.011597
      dz2     :     0.072351  d :     0.388027
      dxz     :     0.095870
      dyz     :     0.066872
      dx2y2   :     0.072894
      dxy     :     0.080040
      f0      :     0.005317  f :     0.038072
      f+1     :     0.006797
      f-1     :     0.004560
      f+2     :     0.005138
      f-2     :     0.005639
      f+3     :     0.005979
      f-3     :     0.004641

 12 H s       :     0.792352  s :     0.792352
      pz      :     0.015001  p :     0.058473
      px      :     0.015641
      py      :     0.027831

 13 H s       :     0.795265  s :     0.795265
      pz      :     0.024544  p :     0.058186
      px      :     0.018147
      py      :     0.015495

 14 C s       :     2.786943  s :     2.786943
```

```
     pz      :     1.039226  p :     3.068634
     px      :     1.034954
     py      :     0.994454
     dz2     :     0.095297  d :     0.412103
     dxz     :     0.095722
     dyz     :     0.081895
     dx2y2   :     0.096335
     dxy     :     0.042854
     f0      :     0.008735  f :     0.041700
     f+1     :     0.006555
     f-1     :     0.004508
     f+2     :     0.006842
     f-2     :     0.006336
     f+3     :     0.004354
     f-3     :     0.004370

 15 H s      :     0.652942  s :     0.652942
     pz      :     0.021328  p :     0.156978
     px      :     0.018275
     py      :     0.117375

 16 H s      :     0.791890  s :     0.791890
     pz      :     0.016837  p :     0.061985
     px      :     0.029770
     py      :     0.015377

 17 C s      :     2.872406  s :     2.872406
     pz      :     1.052050  p :     3.017855
     px      :     1.071733
     py      :     0.894072
     dz2     :     0.104219  d :     0.302600
     dxz     :     0.049753
     dyz     :     0.029344
     dx2y2   :     0.050196
     dxy     :     0.069089
     f0      :     0.003451  f :     0.028515
     f+1     :     0.006461
     f-1     :     0.002763
     f+2     :     0.003997
     f-2     :     0.005748
     f+3     :     0.002357
     f-3     :     0.003738

 18 H s      :     0.808000  s :     0.808000
     pz      :     0.035354  p :     0.064911
     px      :     0.014328
     py      :     0.015230

 19 H s      :     0.803459  s :     0.803459
     pz      :     0.013315  p :     0.066858
     px      :     0.034382
     py      :     0.019162

 20 Ses      :     7.327545  s :     7.327545
     pz      :     5.137936  p :    15.362012
     px      :     4.786224
     py      :     5.437852
     dz2     :     2.099499  d :    10.541781
     dxz     :     2.158655
     dyz     :     2.071884
     dx2y2   :     2.132477
     dxy     :     2.079266
     f0      :     0.014051  f :     0.107623
```

```
      f+1     :     0.029060
      f-1     :     0.003788
      f+2     :     0.023378
      f-2     :     0.018493
      f+3     :     0.009470
      f-3     :     0.009385

 21 O s       :     3.626106  s :     3.626106
      pz      :     1.632361  p :     4.717359
      px      :     1.522794
      py      :     1.562205
      dz2     :     0.010754  d :     0.060943
      dxz     :     0.020291
      dyz     :     0.005315
      dx2y2   :     0.011826
      dxy     :     0.012756
      f0      :     0.000220  f :     0.002813
      f+1     :     0.000629
      f-1     :     0.000225
      f+2     :     0.000626
      f-2     :     0.000401
      f+3     :     0.000378
      f-3     :     0.000334

 22 C s       :     2.786616  s :     2.786616
      pz      :     1.054412  p :     2.977178
      px      :     1.003913
      py      :     0.918853
      dz2     :     0.099036  d :     0.489376
      dxz     :     0.161680
      dyz     :     0.090265
      dx2y2   :     0.069991
      dxy     :     0.068405
      f0      :     0.007855  f :     0.049194
      f+1     :     0.012508
      f-1     :     0.007196
      f+2     :     0.004690
      f-2     :     0.006965
      f+3     :     0.005027
      f-3     :     0.004954

 23 C s       :     2.763311  s :     2.763311
      pz      :     1.031757  p :     2.929897
      px      :     0.997928
      py      :     0.900213
      dz2     :     0.108279  d :     0.384651
      dxz     :     0.122584
      dyz     :     0.067604
      dx2y2   :     0.032784
      dxy     :     0.053400
      f0      :     0.007661  f :     0.044156
      f+1     :     0.012702
      f-1     :     0.005801
      f+2     :     0.005306
      f-2     :     0.005659
      f+3     :     0.002703
      f-3     :     0.004323

 24 C s       :     2.762376  s :     2.762376
      pz      :     0.987863  p :     2.934227
      px      :     1.032684
      py      :     0.913681
      dz2     :     0.103622  d :     0.384395
```

```
      dxz     :     0.086811
      dyz     :     0.041704
      dx2y2   :     0.077751
      dxy     :     0.074507
      f0      :     0.003941  f :     0.043907
      f+1     :     0.011287
      f-1     :     0.003430
      f+2     :     0.006466
      f-2     :     0.007464
      f+3     :     0.005086
      f-3     :     0.006233

 25 C s       :     2.758669  s :     2.758669
      pz      :     0.986127  p :     2.884567
      px      :     1.007811
      py      :     0.890629
      dz2     :     0.100453  d :     0.380333
      dxz     :     0.087024
      dyz     :     0.040175
      dx2y2   :     0.077123
      dxy     :     0.075558
      f0      :     0.004110  f :     0.043399
      f+1     :     0.010937
      f-1     :     0.003196
      f+2     :     0.006519
      f-2     :     0.007547
      f+3     :     0.005221
      f-3     :     0.005869

 26 C s       :     2.758321  s :     2.758321
      pz      :     1.007535  p :     2.884937
      px      :     0.988836
      py      :     0.888567
      dz2     :     0.106121  d :     0.379951
      dxz     :     0.114256
      dyz     :     0.070779
      dx2y2   :     0.033099
      dxy     :     0.055695
      f0      :     0.007464  f :     0.043363
      f+1     :     0.012194
      f-1     :     0.005748
      f+2     :     0.005383
      f-2     :     0.005381
      f+3     :     0.002780
      f-3     :     0.004413

 27 C s       :     2.697664  s :     2.697664
      pz      :     1.001946  p :     2.881289
      px      :     0.958953
      py      :     0.920390
      dz2     :     0.117497  d :     0.511801
      dxz     :     0.158578
      dyz     :     0.088666
      dx2y2   :     0.079456
      dxy     :     0.067604
      f0      :     0.008368  f :     0.057365
      f+1     :     0.015364
      f-1     :     0.007468
      f+2     :     0.006185
      f-2     :     0.008097
      f+3     :     0.006045
      f-3     :     0.005839
```

```
28 H s       :     0.782842  s :     0.782842
     pz      :     0.014350  p :     0.060969
     px      :     0.028226
     py      :     0.018394

29 H s       :     0.780896  s :     0.780896
     pz      :     0.034138  p :     0.059969
     px      :     0.010689
     py      :     0.015142

30 H s       :     0.775642  s :     0.775642
     pz      :     0.034867  p :     0.060295
     px      :     0.010691
     py      :     0.014737

31 H s       :     0.775419  s :     0.775419
     pz      :     0.014384  p :     0.060320
     px      :     0.026896
     py      :     0.019040

32 C s       :     2.812709  s :     2.812709
     pz      :     0.934973  p :     2.831693
     px      :     1.045915
     py      :     0.850805
     dz2     :     0.089306  d :     0.436819
     dxz     :     0.112938
     dyz     :     0.052999
     dx2y2   :     0.087898
     dxy     :     0.093676
     f0      :     0.005818  f :     0.060751
     f+1     :     0.014966
     f-1     :     0.001419
     f+2     :     0.013143
     f-2     :     0.010619
     f+3     :     0.007420
     f-3     :     0.007365

33 N s       :     3.297837  s :     3.297837
     pz      :     1.181538  p :     3.573971
     px      :     1.325434
     py      :     1.066999
     dz2     :     0.018407  d :     0.101692
     dxz     :     0.031820
     dyz     :     0.010711
     dx2y2   :     0.021090
     dxy     :     0.019663
     f0      :     0.001540  f :     0.015972
     f+1     :     0.003829
     f-1     :     0.000350
     f+2     :     0.003622
     f-2     :     0.002764
     f+3     :     0.001930
     f-3     :     0.001936


                      *****************************
                      * MAYER POPULATION ANALYSIS *
                      *****************************

  NA   - Mulliken gross atomic population
  ZA   - Total nuclear charge
  QA   - Mulliken gross atomic charge
```

```
  VA   - Mayer's total valence
  BVA  - Mayer's bonded valence
  FA   - Mayer's free valence

  ATOM       NA         ZA         QA         VA         BVA        FA
  0 C      5.8229     6.0000     0.1771     3.5775     3.5775     0.0000
  1 C      6.2339     6.0000    -0.2339     3.9205     3.9205     0.0000
  2 C      6.2448     6.0000    -0.2448     3.8716     3.8716    -0.0000
  3 C      6.1002     6.0000    -0.1002     3.8392     3.8392     0.0000
  4 C      6.1291     6.0000    -0.1291     3.8276     3.8276    -0.0000
  5 C      6.1724     6.0000    -0.1724     3.8439     3.8439    -0.0000
  6 H      0.8733     1.0000     0.1267     0.9804     0.9804     0.0000
  7 H      0.8610     1.0000     0.1390     0.9677     0.9677    -0.0000
  8 H      0.8607     1.0000     0.1393     0.9682     0.9682     0.0000
  9 H      0.8625     1.0000     0.1375     0.9649     0.9649    -0.0000
 10 H      0.8588     1.0000     0.1412     0.9670     0.9670    -0.0000
 11 C      6.1779     6.0000    -0.1779     3.8464     3.8464     0.0000
 12 H      0.8853     1.0000     0.1147     0.9600     0.9600     0.0000
 13 H      0.8821     1.0000     0.1179     0.9645     0.9645     0.0000
 14 C      6.2667     6.0000    -0.2667     3.7590     3.7590     0.0000
 15 H      0.7425     1.0000     0.2575     1.0130     1.0130     0.0000
 16 H      0.8693     1.0000     0.1307     0.9627     0.9627     0.0000
 17 C      6.2957     6.0000    -0.2957     3.8919     3.8919     0.0000
 18 H      0.8346     1.0000     0.1654     0.9597     0.9597     0.0000
 19 H      0.8319     1.0000     0.1681     0.9603     0.9603     0.0000
 20 Se    33.5645    34.0000     0.4355     2.9551     2.9551     0.0000
 21 O      8.5711     8.0000    -0.5711     1.8258     1.8258    -0.0000
 22 C      5.9338     6.0000     0.0662     3.6938     3.6938    -0.0000
 23 C      6.1484     6.0000    -0.1484     3.7796     3.7796    -0.0000
 24 C      6.1759     6.0000    -0.1759     3.8332     3.8332    -0.0000
 25 C      6.2389     6.0000    -0.2389     3.9294     3.9294     0.0000
 26 C      6.2196     6.0000    -0.2196     3.9043     3.9043     0.0000
 27 C      5.7226     6.0000     0.2774     3.5328     3.5328     0.0000
 28 H      0.8532     1.0000     0.1468     0.9974     0.9974     0.0000
 29 H      0.8423     1.0000     0.1577     0.9665     0.9665    -0.0000
 30 H      0.8438     1.0000     0.1562     0.9723     0.9723     0.0000
 31 H      0.8425     1.0000     0.1575     0.9715     0.9715     0.0000
 32 C      6.1773     6.0000    -0.1773     4.0595     4.0595     0.0000
 33 N      7.0607     7.0000    -0.0607     3.1812     3.1812     0.0000

  Mayer bond orders larger than 0.100000
B(  0-C ,  1-C ) :   1.3481 B(  0-C ,  2-C ) :   1.3222 B(  0-C , 11-C ) :   0.9097
B(  1-C ,  3-C ) :   1.4010 B(  1-C ,  6-H ) :   0.9770 B(  2-C ,  4-C ) :   1.3956
B(  2-C ,  7-H ) :   0.9925 B(  3-C ,  5-C ) :   1.3742 B(  3-C ,  8-H ) :   0.9698
B(  4-C ,  5-C ) :   1.3653 B(  4-C ,  9-H ) :   0.9836 B(  5-C , 10-H ) :   0.9784
B( 11-C , 12-H ) :   0.9487 B( 11-C , 13-H ) :   0.9800 B( 11-C , 14-C ) :   0.9566
B( 14-C , 15-H ) :   0.5483 B( 14-C , 16-H ) :   0.9408 B( 14-C , 17-C ) :   1.2997
B( 15-H , 21-O ) :   0.3880 B( 17-C , 18-H ) :   0.9561 B( 17-C , 19-H ) :   0.9505
B( 17-C , 20-Se) :   0.4938 B( 17-C , 21-O ) :   0.1005 B( 20-Se, 21-O ) :   1.2723
B( 20-Se, 22-C ) :   0.9699 B( 22-C , 23-C ) :   1.2905 B( 22-C , 24-C ) :   1.3157
B( 23-C , 25-C ) :   1.4015 B( 23-C , 28-H ) :   0.9744 B( 24-C , 26-C ) :   1.4073
B( 24-C , 29-H ) :   0.9803 B( 25-C , 27-C ) :   1.2747 B( 25-C , 30-H ) :   0.9958
B( 26-C , 27-C ) :   1.2914 B( 26-C , 31-H ) :   0.9859 B( 27-C , 32-C ) :   0.9653
B( 32-C , 33-N ) :   3.0595

-------
TIMINGS
-------

Total SCF time: 0 days 0 hours 2 min 12 sec

Total time                  ....     132.452 sec
Sum of individual times     ....     127.297 sec  ( 96.1%)
```

```
SCF preparation             ....       0.715 sec  (  0.5%)
Fock matrix formation       ....     121.462 sec  ( 91.7%)
  Startup                   ....       0.056 sec  (  0.0% of F)
  Split-RI-J                ....       9.695 sec  (  8.0% of F)
  Chain of spheres X        ....      90.106 sec  ( 74.2% of F)
  XC integration            ....      13.221 sec  ( 10.9% of F)
    XC Preparation          ....       0.000 sec  (  0.0% of XC)
    Basis function eval.    ....       3.238 sec  ( 24.5% of XC)
    Density eval.           ....       4.388 sec  ( 33.2% of XC)
    XC-Functional eval.     ....       0.120 sec  (  0.9% of XC)
    XC-Potential eval.      ....       4.343 sec  ( 32.8% of XC)
  CPCM terms                ....      12.848 sec  ( 10.6% of F)
Diagonalization             ....       0.000 sec  (  0.0%)
Density matrix formation    ....       0.499 sec  (  0.4%)
Total Energy calculation    ....       0.215 sec  (  0.2%)
Population analysis         ....       0.156 sec  (  0.1%)
Orbital Transformation      ....       0.419 sec  (  0.3%)
Orbital Orthonormalization  ....       0.000 sec  (  0.0%)
DIIS solution               ....       2.294 sec  (  1.7%)
SOSCF solution              ....       1.538 sec  (  1.2%)
Finished LeanSCF after 132.7 sec

Maximum memory used throughout the entire LEANSCF-calculation: 102.8 MB


-------------------------------------------------------------------------------
                          DFT DISPERSION CORRECTION

                              DFTD3 V3.1  Rev 1
                          USING Becke-Johnson damping
-------------------------------------------------------------------------------
The B3LYP functional is recognized
Active option DFTDOPT                   ...         4

molecular C6(AA) [au] = 16315.202855


            DFT-D V3
 parameters
 s6 scaling factor         :     1.0000
 a1 scaling factor         :     0.3981
 s8 scaling factor         :     1.9889
 a2 scaling factor         :     4.4211
 ad hoc parameters k1-k3   :    16.0000     1.3333    -4.0000

 Edisp/kcal,au: -54.440693646669  -0.086756758407
 E6   /kcal   : -25.133023738
 E8   /kcal   : -29.307669909

-------------------------   ----------------
Dispersion correction           -0.086756758
-------------------------   ----------------


-------------------------   --------------------
FINAL SINGLE POINT ENERGY     -3149.990546054587
-------------------------   --------------------


           ************************************************************
           *        Program running with 12 parallel MPI-processes    *
```

```
          *              working on a common directory              *
          ***********************************************************
------------------------------------------------------------------------------
                         ORCA SCF GRADIENT CALCULATION
------------------------------------------------------------------------------

Nuc. rep. gradient        (SHARK) ... done (  0.0 sec)
HCore & Overlap gradient  (SHARK) ... done (  0.5 sec)
Split-RIJ-J gradient      (SHARK) ... done (  2.4 sec)
COSX-gradient                     ... done ( 41.7 sec)
XC gradient                       ... done (  6.3 sec)
CPCM gradient                     ... done (  2.2 sec)
SMD gradient                      ... done (  0.0 sec)
Dispersion correction             ... done (  0.0 sec)

-------------------
DISPERSION GRADIENT
-------------------

   1   C   :   -0.000281473    0.000912178    0.000498617
   2   C   :   -0.000265002    0.000974465    0.000978847
   3   C   :    0.000168439    0.000874023    0.000226755
   4   C   :   -0.000273811    0.001007078    0.000806078
   5   C   :    0.000184276    0.000767251    0.000116290
   6   C   :   -0.000042280    0.000944937    0.000362848
   7   H   :   -0.000239632    0.000083192    0.000530857
   8   H   :    0.000034105    0.000431971   -0.000170248
   9   H   :   -0.000082800    0.000232222    0.000494873
  10   H   :    0.000163224    0.000285827   -0.000114623
  11   H   :    0.000194581    0.000340183    0.000162901
  12   C   :   -0.000695620    0.000401668    0.000034202
  13   H   :   -0.000367102    0.000238329    0.000020248
  14   H   :   -0.000406492    0.000105485    0.000196266
  15   C   :   -0.000758353   -0.000346844   -0.000190755
  16   H   :   -0.000087308   -0.000832859   -0.000178996
  17   H   :   -0.000363715    0.000074051   -0.000157244
  18   C   :   -0.000025668   -0.001618637   -0.000604403
  19   H   :   -0.000073320    0.000111652   -0.000295579
  20   H   :   -0.000195631    0.000123612   -0.000632505
  21   Se  :   -0.000974083   -0.000285897   -0.001616829
  22   O   :   -0.000878453    0.000278268   -0.000257702
  23   C   :    0.000674627   -0.000983178   -0.000212270
  24   C   :    0.000385052   -0.000885598   -0.000049885
  25   C   :    0.000609615   -0.000675768   -0.000386053
  26   C   :    0.000400494   -0.000729472    0.000188816
  27   C   :    0.000662062   -0.000437783   -0.000335826
  28   C   :    0.000450684   -0.000584982   -0.000121892
  29   H   :   -0.000047165   -0.000393821    0.000277070
  30   H   :    0.000339474   -0.000002268   -0.000341241
  31   H   :   -0.000012504   -0.000298945    0.000296652
  32   H   :    0.000292483    0.000054677   -0.000275597
  33   C   :    0.000811459   -0.000205941    0.000327979
  34   N   :    0.000699837    0.000040925    0.000422347

Difference to translation invariance:
           :    0.0000000000   -0.0000000000    0.0000000000

Difference to rotation invariance:
           :   -0.0000000000   -0.0000000000    0.0000000000

Norm of the Dispersion gradient    ...    0.0051995605
RMS gradient                       ...    0.0005148332
MAX gradient                       ...    0.0016186373
```

```
------------------
CARTESIAN GRADIENT
------------------

   1   C   :    0.000001216   -0.000001377    0.000005881
   2   C   :   -0.000001496    0.000009042    0.000006818
   3   C   :    0.000001034   -0.000003677    0.000000032
   4   C   :    0.000001333    0.000002936    0.000002075
   5   C   :    0.000000177   -0.000000166    0.000000240
   6   C   :    0.000000547    0.000002542   -0.000002215
   7   H   :    0.000001114   -0.000000848    0.000000909
   8   H   :    0.000000101    0.000001197    0.000000405
   9   H   :   -0.000002068    0.000007245   -0.000003927
  10   H   :    0.000000479   -0.000000353   -0.000001571
  11   H   :    0.000002637   -0.000001641   -0.000001962
  12   C   :    0.000009522    0.000004606   -0.000001148
  13   H   :   -0.000000151    0.000001869    0.000007763
  14   H   :    0.000002093   -0.000004688    0.000001384
  15   C   :    0.000001886    0.000011474    0.000004710
  16   H   :   -0.000024323   -0.000014315   -0.000018166
  17   H   :   -0.000002357    0.000000847    0.000005557
  18   C   :    0.000002871   -0.000001689   -0.000000820
  19   H   :   -0.000005471   -0.000002798    0.000003277
  20   H   :    0.000000070   -0.000002381    0.000001204
  21   Se  :    0.000001642    0.000000180    0.000002579
  22   O   :    0.000010567    0.000002266    0.000009148
  23   C   :   -0.000001588    0.000001726   -0.000000596
  24   C   :   -0.000000159    0.000002734    0.000000288
  25   C   :   -0.000003315    0.000000325   -0.000003222
  26   C   :    0.000004789    0.000002317   -0.000002358
  27   C   :    0.000002376   -0.000007267   -0.000006761
  28   C   :   -0.000001106   -0.000015345    0.000020348
  29   H   :   -0.000000023   -0.000000069    0.000001088
  30   H   :   -0.000003175    0.000000187   -0.000002882
  31   H   :    0.000004635   -0.000006376    0.000000844
  32   H   :   -0.000001441   -0.000000029   -0.000002678
  33   C   :   -0.000010965   -0.000000746   -0.000009705
  34   N   :    0.000008551    0.000012270   -0.000016541

Difference to translation invariance:
           :   -0.0000000002   -0.0000000007   -0.0000000025

Difference to rotation invariance:
           :   -0.0000496840    0.0004259423   -0.0000362274

Norm of the Cartesian gradient     ...    0.0000611036
RMS gradient                       ...    0.0000060502
MAX gradient                       ...    0.0000243232

-------
TIMINGS
-------

Total SCF gradient time     ....       53.074 sec

Densities                   ....       0.007 sec  (  0.0%)
One electron gradient       ....       0.484 sec  (  0.9%)
RI-J Coulomb gradient       ....       2.358 sec  (  4.4%)
COSX gradient               ....      41.651 sec  ( 78.5%)
XC gradient                 ....       6.321 sec  ( 11.9%)
CPCM gradient               ....       2.199 sec  (  4.1%)
   A-Matrix (El+Nuc)        ....       0.023 sec  (  0.0%)
```

```
   Potential                ....       2.177 sec  (  4.1%)
SMD gradient                ....       0.004 sec  (  0.0%)

Maximum memory used throughout the entire SCFGRAD-calculation: 119.0 MB


           ************************************************************
           *        Program running with 12 parallel MPI-processes    *
           *              working on a common directory               *
           ************************************************************

------------------------------------------------------------------------------
                              ORCA PROPERTY CALCULATIONS
------------------------------------------------------------------------------

GBWName                                 ... CN-IRC.gbw
Number of atoms                         ...     34
Number of basis functions               ...    696
Max core memory                         ...   8000 MB

Electric properties:
Dipole moment                           ... YES
Quadrupole moment                       ...  NO
General moments                         ...  NO (MaxL=10)
Static polarizability (Dipole/Dipole)   ...  NO
Static polarizability (Dipole/Quad.)    ...  NO
Static polarizability (Quad./Quad.)     ...  NO
Static polarizability (Velocity)        ...  NO
Static hyperpolarizability              ...  NO

Atomic electric properties:
Dipole moment                           ...  NO
Quadrupole moment                       ...  NO
Static polarizability                   ...  NO

Choice of electric origin               ... Center of mass
Position of electric origin             ...     -0.688293    -3.363933   -12.476064

General magnetic properties:
Magnetizability                         ...  NO

EPR properties:
g-Tensor (aka g-matrix)                 ...  NO
Zero-Field splitting spin-orbit         ...  NO
Zero-field splitting spin-spin          ...  NO
Hyperfine couplings                     ...  NO (   0 nuclei)
Quadrupole couplings                    ...  NO (   0 nuclei)
Contact density                         ...  NO (   0 nuclei)

NMR properties:
Chemical shifts                         ...  NO (   0 nuclei)
Spin-rotation constants                 ...  NO (   0 nuclei)
Spin-spin couplings                     ...  NO (   0 nuclei,    0 pairs)

Choice of magnetic origin               ... GIAO
Position of magnetic origin             ...      0.000000     0.000000     0.000000

Properties with geometric perturbations:
SCF Hessian                             ...  NO
IR spectrum                             ...  NO
Raman spectrum                          ...  NO
Raman option                            ... 0
VCD spectrum                            ...  NO
```

```
X-ray spectroscopy properties:
SCF XES/XAS/RIXS spectra                ...  NO

SCF SOC stabilization energy            ...  NO
Diagonal Born-Oppenheimer correction    ...  NO

-------------
DIPOLE MOMENT
-------------

Method             : SCF
Type of density    : Electron Density
Multiplicity       :   1
Irrep              :   0
Energy             : -3149.9037892961805483 Eh
Basis              : AO
                                X                 Y                 Z
Electronic contribution:     -4.202823571     -30.482795938     -18.609496435
Nuclear contribution   :      3.102649632      30.896254341      16.515817186
                        -----------------------------------------
Total Dipole Moment    :     -1.100173939       0.413458403      -2.093679249
                        -----------------------------------------
Magnitude (a.u.)       :      2.401004654
Magnitude (Debye)      :      6.102868826



--------------------
Rotational spectrum
--------------------

Rotational constants in cm-1:             0.013957             0.009149             0.006468
Rotational constants in MHz :           418.414702           274.280673           193.907019

Dipole components along the rotational axes:
x,y,z [a.u.] :    -1.953808    -0.253749    -1.372249
x,y,z [Debye]:    -4.966186    -0.644978    -3.487980



Dipole moment calculation done in   0.0 sec

Maximum memory used throughout the entire PROP-calculation: 60.9 MB


--------------------------------------------------------------------------------
                   Intrinsic Reaction Coordinate Calculation
--------------------------------------------------------------------------------

IRC Settings:
  Nr. of atoms                        .... 34
  Algorithm                           .... LQA
  Coordinate system following type    .... Mass-Weighted
  Max. no of cycles (MaxIter)         .... 241
  Direction                           .... Forward and backward
  Steep Lenght                        .... 0.110 bohr Dalton^(1/2)

Initial displacement:
  Hessian type                        .... Read
  Hessian Filename                    .... TS-CN_Compound_2.hess
  Following Hessian mode              .... 0
  Initial displacement type           .... Length
    Scaling normalized TS mode by     .... 0.040
```

```
Convergence Tolerances:
  Energy Change                      ....  5.0000e-06 Eh
  Max. Gradient                      ....  8.0000e-05 Eh/bohr
  RMS Gradient                       ....  1.0000e-05 Eh/bohr
  Max. Displacement                  ....  4.0000e-03 bohr
  RMS Displacement                   ....  2.0000e-03 bohr
  Strict Convergency criteria enforced

LQA Settings:
  Hessian update type                .... Bofill
  Update steep lenght                .... NO
  t Stepsize                         .... Gradient-based adjusted
  t MAX int. Steps                   .... 1000000
  Bulirsch-Stoer Correction          .... YES
    Euler Initial Steps              .... 100
    Richardson Max extrapolations    .... 20
    Richardson error threshould      ....  1.0000e-06
    Apply BS correction on TS disp.  .... NO
    Keep LQA direction               .... NO
    LQA direction Theshould          ....  1.0000e-01
    Update Hessian and Gradient      .... YES

Storing full IRC trajectory in       .... CN-IRC_IRC_Full_trj.xyz
Storing forward trajectory in        .... CN-IRC_IRC_F_trj.xyz
Storing backward trajectory in       .... CN-IRC_IRC_B_trj.xyz
Storing IRC profile in               .... CN-IRC.IRC.out


         *************************************************************
         *                         FORWARD IRC                       *
         *************************************************************

Iteration    E(Eh)      dE(kcal/mol)  max(|G|)  RMS(G)   max(|S|)  RMS(S)   MW-Displ.
Convergence thresholds    0.003138    0.000080  0.000010  0.004000  0.002000
    0      -3149.990592   -0.028848   0.002311  0.000492  0.035537  0.003961  0.046716
    1      -3149.990966   -0.263785   0.007352  0.001416  0.074915  0.008713  0.110002
    2      -3149.991542   -0.625224   0.011322  0.002052  0.060537  0.007800  0.110001
    3      -3149.992192   -1.032739   0.013938  0.002453  0.042553  0.006752  0.110002
    4      -3149.992876   -1.461762   0.015582  0.002714  0.030723  0.005998  0.110000
    5      -3149.993589   -1.909773   0.016613  0.002896  0.029284  0.005508  0.110001
    6      -3149.994321   -2.369039   0.017736  0.003023  0.027890  0.005192  0.110004
    7      -3149.995067   -2.836776   0.018892  0.003130  0.026668  0.004981  0.110001
    8      -3149.995825   -3.312782   0.019885  0.003218  0.025535  0.004823  0.110002
    9      -3149.996597   -3.796704   0.020757  0.003294  0.024542  0.004705  0.110001
   10      -3149.997377   -4.286192   0.021533  0.003360  0.023652  0.004614  0.110003
   11      -3149.998165   -4.780788   0.022229  0.003419  0.022849  0.004541  0.110000
   12      -3149.998962   -5.280837   0.022859  0.003472  0.022128  0.004483  0.110003
   13      -3149.999766   -5.785290   0.023426  0.003520  0.021483  0.004436  0.110001
   14      -3150.000579   -6.295473   0.023936  0.003562  0.020910  0.004399  0.110000
   15      -3150.001392   -6.805972   0.024383  0.003598  0.020407  0.004370  0.110003
   16      -3150.002211   -7.320040   0.024770  0.003629  0.019957  0.004349  0.110000
   17      -3150.003037   -7.838440   0.025093  0.003653  0.019577  0.004333  0.110003
   18      -3150.003863   -8.356505   0.025347  0.003670  0.019255  0.004323  0.110000
   19      -3150.004691   -8.876341   0.025531  0.003680  0.019006  0.004318  0.110001
   20      -3150.005520   -9.396070   0.025641  0.003682  0.018812  0.004317  0.110003
   21      -3150.006354   -9.919820   0.025680  0.003675  0.018637  0.004319  0.110001
   22      -3150.007169   -10.430887   0.025631  0.003658  0.018472  0.004321  0.110003
   23      -3150.007985   -10.942967   0.025507  0.003631  0.018200  0.004321  0.110001
   24      -3150.008792   -11.449176   0.025304  0.003593  0.017873  0.004317  0.110003
   25      -3150.009585   -11.946783   0.025023  0.003546  0.017470  0.004306  0.110002
   26      -3150.010358   -12.432303   0.024665  0.003492  0.017017  0.004280  0.110001
   27      -3150.011110   -12.904124   0.024219  0.003427  0.016618  0.004234  0.110000
```

28	-3150.011842	-13.363397	0.023682	0.003349	0.016366	0.004202	0.110001
29	-3150.012555	-13.810507	0.023065	0.003261	0.016129	0.004192	0.110002
30	-3150.013244	-14.243141	0.022373	0.003165	0.015782	0.004179	0.110002
31	-3150.013908	-14.660113	0.021605	0.003059	0.015369	0.004153	0.110002
32	-3150.014545	-15.059484	0.020762	0.002945	0.015047	0.004123	0.110001
33	-3150.015153	-15.440942	0.019846	0.002823	0.014707	0.004094	0.110001
34	-3150.015731	-15.803584	0.018860	0.002693	0.014319	0.004061	0.110000
35	-3150.016277	-16.146677	0.017808	0.002555	0.014177	0.004026	0.110001
36	-3150.016792	-16.469674	0.016695	0.002412	0.014353	0.003989	0.110002
37	-3150.017274	-16.772145	0.015527	0.002262	0.014558	0.003951	0.110002
38	-3150.017723	-17.053621	0.014313	0.002109	0.014791	0.003912	0.110002
39	-3150.018139	-17.314689	0.013063	0.001953	0.015046	0.003874	0.110000
40	-3150.018522	-17.555001	0.011796	0.001797	0.015326	0.003840	0.110001
41	-3150.018874	-17.775927	0.010519	0.001639	0.015597	0.003811	0.110002
42	-3150.019193	-17.976272	0.009260	0.001492	0.015887	0.003792	0.110002
43	-3150.019489	-18.161761	0.008019	0.001329	0.016080	0.003791	0.110002
44	-3150.019742	-18.320924	0.006871	0.001245	0.016372	0.003818	0.110002
45	-3150.020001	-18.483344	0.006213	0.001024	0.016095	0.003952	0.110001
46	-3150.020190	-18.602129	0.005698	0.001235	0.017910	0.004873	0.110002
47	-3150.020430	-18.752724	0.005228	0.000801	0.016559	0.004095	0.110004
48	-3150.020625	-18.874800	0.004676	0.000758	0.018444	0.004628	0.110002
49	-3150.020807	-18.989193	0.004199	0.000675	0.018725	0.004639	0.110001
50	-3150.020981	-19.098176	0.003772	0.000603	0.020134	0.004931	0.110000
51	-3150.021144	-19.200594	0.003399	0.000562	0.021284	0.005299	0.110000
52	-3150.021305	-19.301175	0.003088	0.000537	0.022705	0.005622	0.110001
53	-3150.021462	-19.399827	0.002828	0.000517	0.023546	0.005811	0.110001
54	-3150.021618	-19.498192	0.002613	0.000497	0.024276	0.005936	0.110001
55	-3150.021775	-19.596612	0.002436	0.000478	0.024884	0.006041	0.110001
56	-3150.021931	-19.694123	0.002291	0.000464	0.025123	0.006134	0.110000
57	-3150.022085	-19.790860	0.002170	0.000463	0.025019	0.006219	0.110001
58	-3150.022238	-19.887065	0.002070	0.000473	0.024775	0.006273	0.110001
59	-3150.022389	-19.981913	0.001988	0.000484	0.024603	0.006269	0.110000
60	-3150.022539	-20.076028	0.001921	0.000490	0.024469	0.006230	0.110001
61	-3150.022690	-20.170826	0.001867	0.000487	0.024708	0.006186	0.110000
62	-3150.022843	-20.266640	0.001823	0.000480	0.025024	0.006191	0.110001
63	-3150.022996	-20.362727	0.001786	0.000476	0.025219	0.006227	0.110000
64	-3150.023148	-20.458275	0.001752	0.000479	0.025239	0.006260	0.110001
65	-3150.023301	-20.553837	0.001786	0.000486	0.025104	0.006274	0.110000
66	-3150.023451	-20.648192	0.001835	0.000490	0.024898	0.006263	0.110001
67	-3150.023601	-20.742092	0.001863	0.000492	0.024657	0.006231	0.110000
68	-3150.023749	-20.835262	0.001886	0.000493	0.024443	0.006186	0.110000
69	-3150.023896	-20.927514	0.001921	0.000494	0.024262	0.006148	0.110001
70	-3150.024042	-21.018751	0.001955	0.000495	0.024100	0.006121	0.110000
71	-3150.024185	-21.108792	0.001991	0.000497	0.023938	0.006095	0.110000
72	-3150.024326	-21.197398	0.002023	0.000496	0.023824	0.006054	0.110000
73	-3150.024465	-21.284398	0.002038	0.000494	0.023809	0.006027	0.110000
74	-3150.024601	-21.369570	0.002094	0.000496	0.023547	0.005990	0.110000
75	-3150.024734	-21.453248	0.002135	0.000497	0.023377	0.005955	0.110000
76	-3150.024865	-21.535524	0.002161	0.000496	0.023232	0.005920	0.110001
77	-3150.024994	-21.616230	0.002193	0.000496	0.023026	0.005889	0.110001
78	-3150.025120	-21.695546	0.002219	0.000496	0.022853	0.005858	0.110001
79	-3150.025244	-21.773440	0.002241	0.000495	0.022673	0.005828	0.110001
80	-3150.025366	-21.849861	0.002261	0.000495	0.022458	0.005798	0.110000
81	-3150.025486	-21.924805	0.002275	0.000494	0.022260	0.005762	0.110000
82	-3150.025603	-21.998249	0.002283	0.000493	0.022137	0.005726	0.110001
83	-3150.025717	-22.070129	0.002282	0.000492	0.022165	0.005688	0.110001
84	-3150.025829	-22.140401	0.002267	0.000490	0.022161	0.005646	0.110000
85	-3150.025938	-22.209032	0.002233	0.000488	0.022127	0.005602	0.110000
86	-3150.026045	-22.276022	0.002168	0.000485	0.022060	0.005554	0.110000
87	-3150.026149	-22.341374	0.002060	0.000482	0.021957	0.005501	0.110001
88	-3150.026251	-22.404941	0.001907	0.000480	0.021777	0.005443	0.110001
89	-3150.026350	-22.467333	0.001746	0.000472	0.021581	0.005379	0.110001
90	-3150.026446	-22.527570	0.001874	0.000470	0.021402	0.005316	0.110000

91	-3150.026532	-22.581159	0.001939	0.000467	0.021086	0.005228	0.110000
92	-3150.026620	-22.636724	0.001959	0.000461	0.020809	0.005139	0.110001
93	-3150.026704	-22.689614	0.002002	0.000468	0.020471	0.005085	0.110001
94	-3150.026786	-22.740878	0.001919	0.000452	0.020183	0.004975	0.110001
95	-3150.026863	-22.788876	0.001997	0.000466	0.019995	0.004988	0.110001
96	-3150.026940	-22.837211	0.001753	0.000422	0.019907	0.004843	0.110001
97	-3150.027006	-22.878663	0.001930	0.000474	0.019960	0.004976	0.110000
98	-3150.027087	-22.929807	0.001274	0.000356	0.019926	0.004818	0.110000
99	-3150.027145	-22.966310	0.002231	0.000415	0.020974	0.004989	0.110000
100	-3150.027219	-23.012318	0.001449	0.000309	0.019986	0.005520	0.110000
101	-3150.027277	-23.048769	0.001668	0.000396	0.020605	0.005474	0.110000
102	-3150.027340	-23.088440	0.001241	0.000317	0.024135	0.005794	0.110001
103	-3150.027370	-23.107234	0.003828	0.000661	0.022952	0.005892	0.110000
104	-3150.027396	-23.123813	0.002663	0.000481	0.013090	0.002357	0.110001
105	-3150.027439	-23.150759	0.002337	0.000512	0.025016	0.005716	0.110001
106	-3150.027507	-23.193173	0.000962	0.000210	0.015575	0.004299	0.110001
107	-3150.027552	-23.221777	0.001468	0.000394	0.020104	0.005194	0.110001
108	-3150.027603	-23.253788	0.000999	0.000290	0.016261	0.004465	0.110001
109	-3150.027649	-23.282164	0.000873	0.000244	0.018170	0.004722	0.110000
110	-3150.027687	-23.306295	0.001506	0.000325	0.018080	0.004659	0.110000
111	-3150.027728	-23.331668	0.001482	0.000291	0.015940	0.004211	0.110000
112	-3150.027765	-23.355088	0.001463	0.000279	0.016223	0.004287	0.110000
113	-3150.027800	-23.377372	0.001550	0.000292	0.015012	0.004173	0.110000
114	-3150.027835	-23.398894	0.001562	0.000300	0.014451	0.004123	0.110000
115	-3150.027868	-23.419951	0.001515	0.000301	0.014322	0.004131	0.110000
116	-3150.027901	-23.440408	0.001481	0.000298	0.014392	0.004143	0.110000
117	-3150.027933	-23.460470	0.001509	0.000293	0.014342	0.004127	0.110000
118	-3150.027964	-23.479925	0.001556	0.000288	0.014122	0.004083	0.110000
119	-3150.027994	-23.498878	0.001572	0.000286	0.013863	0.004042	0.110000
120	-3150.028024	-23.517722	0.001559	0.000291	0.013665	0.004025	0.110000
121	-3150.028053	-23.536139	0.001525	0.000297	0.013404	0.004027	0.110000
122	-3150.028082	-23.554155	0.001493	0.000303	0.013184	0.004027	0.110000
123	-3150.028111	-23.572232	0.001478	0.000302	0.013085	0.003979	0.110000
124	-3150.028129	-23.583707	0.001485	0.000297	0.013137	0.003892	0.110000
125	-3150.028167	-23.607298	0.001484	0.000282	0.013061	0.003923	0.110000
126	-3150.028194	-23.624212	0.001519	0.000283	0.013523	0.003886	0.110000
127	-3150.028210	-23.634128	0.001577	0.000283	0.012816	0.003847	0.110000
128	-3150.028246	-23.656943	0.001563	0.000276	0.013024	0.003783	0.110000
129	-3150.028271	-23.672416	0.001625	0.000295	0.013234	0.003875	0.110000
130	-3150.028297	-23.688878	0.001561	0.000282	0.012301	0.003774	0.110000
131	-3150.028313	-23.698955	0.001610	0.000290	0.012813	0.003850	0.110000
132	-3150.028348	-23.720823	0.001577	0.000275	0.012411	0.003800	0.110000
133	-3150.028371	-23.735732	0.001628	0.000297	0.012867	0.003909	0.110000
134	-3150.028397	-23.751846	0.001540	0.000285	0.011874	0.003766	0.110000
135	-3150.028421	-23.766804	0.001564	0.000303	0.012714	0.003849	0.110000
136	-3150.028446	-23.782726	0.001528	0.000294	0.011885	0.003738	0.110000
137	-3150.028470	-23.797824	0.001602	0.000301	0.012407	0.003788	0.110000
138	-3150.028495	-23.813312	0.001605	0.000289	0.011751	0.003713	0.110000
139	-3150.028518	-23.827973	0.001688	0.000296	0.012295	0.003771	0.110000
140	-3150.028543	-23.843369	0.001645	0.000288	0.011786	0.003727	0.110000
141	-3150.028566	-23.858064	0.001676	0.000296	0.012194	0.003778	0.110000
142	-3150.028576	-23.864001	0.001226	0.000255	0.013248	0.002327	0.110000
143	-3150.028601	-23.880082	0.000473	0.000143	0.015636	0.004079	0.110001
144	-3150.028629	-23.897126	0.000689	0.000163	0.014154	0.004486	0.110000
145	-3150.028651	-23.910887	0.001060	0.000222	0.009972	0.003994	0.110000
146	-3150.028676	-23.926548	0.000999	0.000227	0.011019	0.003860	0.110000
147	-3150.028701	-23.942287	0.000914	0.000230	0.011204	0.003919	0.110000
148	-3150.028725	-23.957423	0.000944	0.000256	0.012306	0.004002	0.110000
149	-3150.028748	-23.972336	0.001373	0.000289	0.011942	0.003981	0.110000
150	-3150.028772	-23.986985	0.001973	0.000324	0.012961	0.004049	0.110000
151	-3150.028796	-24.001844	0.002139	0.000323	0.010614	0.003850	0.110000
152	-3150.028821	-24.017774	0.001399	0.000228	0.013219	0.004026	0.110000
153	-3150.028842	-24.031008	0.000896	0.000282	0.013339	0.004349	0.110000

154	-3150.028867	-24.046933	0.001009	0.000261	0.012390	0.003952	0.110000
155	-3150.028891	-24.061917	0.000963	0.000272	0.013028	0.004020	0.110000
156	-3150.028912	-24.075086	0.001716	0.000315	0.013447	0.003979	0.110000
157	-3150.028936	-24.089934	0.001426	0.000256	0.012751	0.003793	0.110000
158	-3150.028958	-24.103676	0.001672	0.000291	0.013203	0.004088	0.110000
159	-3150.028966	-24.109025	0.001681	0.000303	0.017300	0.002521	0.110000
160	-3150.028988	-24.122909	0.000916	0.000220	0.011664	0.003911	0.110002
161	-3150.029008	-24.135372	0.001821	0.000275	0.019133	0.004733	0.110000
162	-3150.029030	-24.148772	0.000969	0.000240	0.009085	0.003768	0.110000
163	-3150.029053	-24.163713	0.001152	0.000252	0.011230	0.004075	0.110000
164	-3150.029076	-24.177910	0.000998	0.000245	0.010400	0.003884	0.110000
165	-3150.029098	-24.191875	0.000961	0.000234	0.009913	0.003848	0.110000
166	-3150.029121	-24.206197	0.000947	0.000230	0.010171	0.003863	0.110000
167	-3150.029143	-24.220148	0.000947	0.000230	0.010877	0.003869	0.110000
168	-3150.029166	-24.234277	0.000944	0.000228	0.011085	0.003862	0.110000
169	-3150.029188	-24.248109	0.000966	0.000229	0.010983	0.003879	0.110000
170	-3150.029210	-24.261741	0.000992	0.000236	0.011208	0.003896	0.110000
171	-3150.029230	-24.274318	0.000986	0.000242	0.011620	0.003935	0.110000
172	-3150.029251	-24.287581	0.000992	0.000246	0.011983	0.003960	0.110000
173	-3150.029272	-24.300891	0.000998	0.000245	0.012139	0.003983	0.110000
174	-3150.029293	-24.314135	0.000965	0.000241	0.012047	0.003993	0.110000
175	-3150.029314	-24.327390	0.000962	0.000235	0.011703	0.003980	0.110000
176	-3150.029336	-24.341228	0.000951	0.000230	0.010990	0.003956	0.110000
177	-3150.029357	-24.353892	0.000946	0.000229	0.011414	0.003947	0.110000
178	-3150.029378	-24.367429	0.000911	0.000222	0.011230	0.003911	0.110000
179	-3150.029397	-24.379462	0.000901	0.000222	0.011233	0.003943	0.110000
180	-3150.029419	-24.393158	0.000908	0.000224	0.010891	0.003922	0.110000
181	-3150.029438	-24.404836	0.000943	0.000229	0.010925	0.003929	0.110000
182	-3150.029459	-24.418242	0.000961	0.000233	0.010819	0.003925	0.110000
183	-3150.029479	-24.430826	0.000985	0.000237	0.011616	0.003945	0.110000
184	-3150.029498	-24.442639	0.000967	0.000233	0.011838	0.003954	0.110000
185	-3150.029519	-24.456126	0.000945	0.000232	0.011543	0.003984	0.110000
186	-3150.029538	-24.467925	0.000929	0.000225	0.012300	0.003966	0.110000
187	-3150.029559	-24.480888	0.000853	0.000220	0.011139	0.003985	0.110000
188	-3150.029578	-24.493170	0.000874	0.000226	0.011202	0.004000	0.110000
189	-3150.029598	-24.505632	0.000863	0.000219	0.010850	0.003915	0.110000
190	-3150.029624	-24.521584	0.000878	0.000224	0.011163	0.003977	0.110000
191	-3150.029637	-24.529880	0.000752	0.000189	0.012495	0.003779	0.110000
192	-3150.029657	-24.542678	0.000799	0.000207	0.010171	0.004218	0.110000
193	-3150.029676	-24.554307	0.000877	0.000237	0.011791	0.004101	0.110000
194	-3150.029696	-24.566846	0.000763	0.000201	0.009401	0.003779	0.110000
195	-3150.029715	-24.579133	0.000863	0.000214	0.011721	0.004170	0.110000
196	-3150.029733	-24.590431	0.000968	0.000239	0.012134	0.004047	0.110000
197	-3150.029753	-24.602766	0.000888	0.000221	0.011396	0.003905	0.110000
198	-3150.029770	-24.613463	0.000888	0.000226	0.011608	0.004040	0.110000
199	-3150.029790	-24.625778	0.000909	0.000217	0.009837	0.003940	0.110000
200	-3150.029808	-24.636917	0.000965	0.000223	0.010561	0.003985	0.110000
201	-3150.029826	-24.648370	0.000895	0.000211	0.009676	0.003875	0.110000
202	-3150.029843	-24.659360	0.000945	0.000230	0.010663	0.004017	0.110000
203	-3150.029858	-24.668626	0.000841	0.000201	0.009909	0.003772	0.110000
204	-3150.029877	-24.680724	0.000980	0.000229	0.010167	0.004140	0.110000
205	-3150.029895	-24.691859	0.000929	0.000206	0.010111	0.003804	0.110000
206	-3150.029911	-24.702030	0.000983	0.000202	0.011158	0.003858	0.110000
207	-3150.029927	-24.712171	0.001087	0.000231	0.010131	0.004075	0.110000
208	-3150.029945	-24.722904	0.000976	0.000205	0.009418	0.003758	0.110000
209	-3150.029959	-24.732238	0.001006	0.000220	0.009610	0.003932	0.110000
210	-3150.029976	-24.742522	0.001005	0.000215	0.009805	0.003842	0.110000
211	-3150.029991	-24.751718	0.001017	0.000219	0.009579	0.003888	0.110000
212	-3150.030006	-24.761458	0.001051	0.000216	0.009877	0.003824	0.110000
213	-3150.030021	-24.770730	0.001029	0.000214	0.009465	0.003845	0.110000
214	-3150.030035	-24.779867	0.001124	0.000226	0.010267	0.003891	0.110000
215	-3150.030052	-24.789994	0.000917	0.000187	0.009022	0.003659	0.110000
216	-3150.030065	-24.798513	0.001114	0.000225	0.012400	0.004187	0.110000

217	-3150.030081	-24.808528	0.000905	0.000184	0.009081	0.003696	0.110000
218	-3150.030094	-24.816515	0.001130	0.000224	0.012860	0.004207	0.110000
219	-3150.030110	-24.826392	0.000934	0.000188	0.009329	0.003753	0.110000
220	-3150.030122	-24.834380	0.001132	0.000222	0.012368	0.004211	0.110000
221	-3150.030137	-24.843621	0.000992	0.000194	0.009379	0.003775	0.110000
222	-3150.030149	-24.850933	0.001211	0.000228	0.011491	0.004102	0.110000
223	-3150.030152	-24.852867	0.000995	0.000203	0.009236	0.001996	0.110000
224	-3150.030167	-24.862591	0.000736	0.000139	0.012507	0.003556	0.110002
225	-3150.030179	-24.870137	0.000630	0.000161	0.008991	0.003483	0.110000
226	-3150.030193	-24.879017	0.000390	0.000097	0.015383	0.004890	0.110000
227	-3150.030208	-24.887981	0.000520	0.000121	0.012032	0.004473	0.110000
228	-3150.030217	-24.893683	0.001134	0.000285	0.013227	0.004488	0.110000
229	-3150.030217	-24.893684	0.001251	0.000303	0.014795	0.002582	0.110000
230	-3150.030234	-24.904599	0.000681	0.000158	0.011581	0.003713	0.110001
231	-3150.030249	-24.913790	0.000245	0.000070	0.014532	0.004690	0.110000
232	-3150.030262	-24.922176	0.000388	0.000104	0.011906	0.004886	0.110000
233	-3150.030274	-24.929879	0.000662	0.000161	0.011921	0.004620	0.110000
234	-3150.030285	-24.936256	0.000900	0.000231	0.011353	0.004415	0.110000
235	-3150.030301	-24.946377	0.000316	0.000088	0.009797	0.003199	0.110000
236	-3150.030307	-24.950070	0.000536	0.000144	0.009466	0.003693	0.110000
237	-3150.030315	-24.955647	0.001013	0.000237	0.011365	0.004435	0.110000
238	-3150.030328	-24.963730	0.000262	0.000065	0.010530	0.003414	0.110000
239	-3150.030339	-24.970269	0.000814	0.000167	0.012973	0.004584	0.110000
240	-3150.030345	-24.974167	0.000986	0.000220	0.010414	0.003390	0.110000

```
**************************************************************
*  MAXIMUM NUMBER OF ITERATIONS REACHED - STOPPING IRC RUN   *
**************************************************************


**************************************************************
*                          BACKWARD IRC                      *
**************************************************************
```

Iteration	E(Eh)	dE(kcal/mol)	max(|G|)	RMS(G)	max(|S|)	RMS(S)	MW-Displ.
Convergence thresholds		0.003138	0.000080	0.000010	0.004000	0.002000	
0	-3149.990596	-0.031354	0.002260	0.000530	0.035537	0.003961	0.046716
1	-3149.991235	-0.432037	0.008168	0.002030	0.087974	0.009635	0.110005
2	-3149.992817	-1.424814	0.015594	0.003660	0.090984	0.009853	0.110001
3	-3149.995125	-2.873499	0.020828	0.004779	0.090507	0.009821	0.110002
4	-3149.997539	-4.388051	0.024950	0.005314	0.084980	0.009422	0.110005
5	-3149.999610	-5.687528	0.027606	0.005436	0.069885	0.008392	0.110002
6	-3150.001278	-6.734289	0.027944	0.005305	0.042743	0.006791	0.110003
7	-3150.002726	-7.642913	0.026782	0.005058	0.026518	0.005772	0.110003
8	-3150.004030	-8.461524	0.025074	0.004795	0.022688	0.005220	0.110004
9	-3150.005226	-9.211971	0.023363	0.004552	0.020341	0.004850	0.110004
10	-3150.006333	-9.906733	0.023405	0.004333	0.020319	0.004597	0.110000
11	-3150.007373	-10.559175	0.023336	0.004139	0.020098	0.004436	0.110000
12	-3150.008353	-11.174280	0.023172	0.003964	0.019862	0.004345	0.110000
13	-3150.009284	-11.757925	0.022928	0.003806	0.020752	0.004301	0.110003
14	-3150.010171	-12.314885	0.022619	0.003663	0.021243	0.004288	0.110004
15	-3150.011021	-12.848198	0.022260	0.003529	0.021445	0.004292	0.110003
16	-3150.011836	-13.359834	0.021861	0.003403	0.021455	0.004305	0.110003
17	-3150.012617	-13.849702	0.021437	0.003283	0.021339	0.004322	0.110002
18	-3150.013369	-14.321887	0.020993	0.003170	0.021138	0.004342	0.110003
19	-3150.014095	-14.777316	0.020537	0.003062	0.020880	0.004362	0.110000
20	-3150.014792	-15.214375	0.020071	0.002958	0.020594	0.004382	0.110003
21	-3150.015462	-15.634772	0.019600	0.002859	0.020296	0.004400	0.110001
22	-3150.016107	-16.039552	0.019126	0.002764	0.019994	0.004417	0.110000
23	-3150.016728	-16.429215	0.018655	0.002675	0.019692	0.004433	0.110001
24	-3150.017323	-16.802489	0.018193	0.002594	0.019376	0.004446	0.110002
25	-3150.017890	-17.158237	0.017750	0.002532	0.018984	0.004449	0.110002
26	-3150.018419	-17.490629	0.017330	0.002497	0.018369	0.004390	0.110002

27	-3150.018929	-17.810359	0.016899	0.002424	0.017636	0.004256	0.110001
28	-3150.019415	-18.115433	0.016471	0.002352	0.017295	0.004243	0.110001
29	-3150.019881	-18.408216	0.016042	0.002284	0.017312	0.004230	0.110002
30	-3150.020329	-18.688866	0.015617	0.002218	0.017427	0.004210	0.110001
31	-3150.020758	-18.957973	0.015195	0.002153	0.017528	0.004190	0.110001
32	-3150.021169	-19.216076	0.014779	0.002090	0.017619	0.004172	0.110002
33	-3150.021564	-19.463916	0.014364	0.002029	0.017683	0.004153	0.110000
34	-3150.021942	-19.701350	0.013941	0.001967	0.017755	0.004133	0.110001
35	-3150.022305	-19.928960	0.013557	0.001910	0.017817	0.004116	0.110000
36	-3150.022653	-20.147288	0.013180	0.001855	0.017862	0.004094	0.110001
37	-3150.022987	-20.356648	0.012811	0.001802	0.017899	0.004074	0.110001
38	-3150.023307	-20.557483	0.012450	0.001750	0.017925	0.004053	0.110000
39	-3150.023614	-20.750094	0.012098	0.001701	0.017938	0.004033	0.110001
40	-3150.023908	-20.934878	0.011753	0.001652	0.017940	0.004011	0.110000
41	-3150.024190	-21.112090	0.011416	0.001606	0.017932	0.003990	0.110002
42	-3150.024461	-21.282107	0.011086	0.001561	0.017911	0.003968	0.110001
43	-3150.024721	-21.445113	0.010765	0.001518	0.017876	0.003945	0.110001
44	-3150.024970	-21.601594	0.010450	0.001476	0.017830	0.003922	0.110001
45	-3150.025209	-21.751574	0.010144	0.001436	0.017768	0.003899	0.110001
46	-3150.025439	-21.895688	0.009844	0.001396	0.017697	0.003874	0.110000
47	-3150.025659	-22.033629	0.009552	0.001361	0.017611	0.003852	0.110000
48	-3150.025871	-22.166515	0.009267	0.001321	0.017519	0.003825	0.110001
49	-3150.026073	-22.293225	0.008989	0.001293	0.017410	0.003803	0.110001
50	-3150.026269	-22.416242	0.008718	0.001248	0.017300	0.003774	0.110001
51	-3150.026453	-22.531996	0.008454	0.001236	0.017168	0.003755	0.110001
52	-3150.026637	-22.647243	0.008197	0.001169	0.017047	0.003722	0.110000
53	-3150.026802	-22.751190	0.007944	0.001200	0.016890	0.003718	0.110000
54	-3150.026980	-22.862688	0.007696	0.001068	0.016634	0.003672	0.110001
55	-3150.027133	-22.958320	0.007433	0.001137	0.016733	0.003757	0.110001
56	-3150.027300	-23.063408	0.007207	0.001001	0.016753	0.003686	0.110001
57	-3150.027441	-23.151745	0.006954	0.001054	0.016586	0.003762	0.110001
58	-3150.027594	-23.247950	0.006750	0.000986	0.016902	0.003688	0.110001
59	-3150.027722	-23.328284	0.006533	0.001057	0.015938	0.003624	0.110000
60	-3150.027868	-23.419837	0.006331	0.000899	0.015963	0.003578	0.110000
61	-3150.027993	-23.498503	0.006089	0.000918	0.016058	0.003699	0.110000
62	-3150.028116	-23.575500	0.005909	0.000963	0.016413	0.003647	0.110000
63	-3150.028240	-23.653020	0.005735	0.000885	0.015174	0.003514	0.110000
64	-3150.028346	-23.719752	0.005538	0.000948	0.015299	0.003516	0.110000
65	-3150.028466	-23.795105	0.005372	0.000821	0.015047	0.003476	0.110000
66	-3150.028566	-23.857771	0.005172	0.000894	0.014914	0.003532	0.110000
67	-3150.028676	-23.926804	0.005022	0.000806	0.015035	0.003489	0.110001
68	-3150.028768	-23.984296	0.004846	0.000883	0.014386	0.003473	0.110001
69	-3150.028797	-24.002510	0.005246	0.000946	0.020217	0.002453	0.110000
70	-3150.028905	-24.070391	0.004592	0.000571	0.008297	0.003085	0.110001
71	-3150.029014	-24.139078	0.004401	0.000548	0.016411	0.004047	0.110001
72	-3150.029125	-24.208840	0.004220	0.000534	0.016842	0.003991	0.110001
73	-3150.029213	-24.263583	0.004053	0.000529	0.016084	0.003902	0.110000
74	-3150.029307	-24.322664	0.003897	0.000511	0.015395	0.003862	0.110000
75	-3150.029397	-24.379562	0.003746	0.000489	0.015554	0.003888	0.110000
76	-3150.029481	-24.432136	0.003598	0.000469	0.015847	0.003929	0.110000
77	-3150.029566	-24.485212	0.003450	0.000454	0.015516	0.003931	0.110001
78	-3150.029648	-24.536718	0.003310	0.000442	0.015146	0.003888	0.110000
79	-3150.029724	-24.584640	0.003173	0.000433	0.014788	0.003864	0.110000
80	-3150.029798	-24.630623	0.003040	0.000421	0.014591	0.003804	0.110000
81	-3150.029867	-24.674361	0.002910	0.000410	0.014453	0.003803	0.110000
82	-3150.029935	-24.716653	0.002792	0.000400	0.014278	0.003793	0.110000
83	-3150.029998	-24.756179	0.002666	0.000391	0.014030	0.003781	0.110000
84	-3150.030061	-24.796082	0.002552	0.000379	0.013792	0.003771	0.110000
85	-3150.030120	-24.833084	0.002440	0.000370	0.013533	0.003778	0.110000
86	-3150.030177	-24.868979	0.002334	0.000362	0.013253	0.003762	0.110000
87	-3150.030232	-24.903438	0.002227	0.000353	0.012987	0.003752	0.110001
88	-3150.030284	-24.935936	0.002121	0.000344	0.012860	0.003743	0.110000
89	-3150.030334	-24.967185	0.002028	0.000337	0.012505	0.003732	0.110000

90	-3150.030383	-24.998060	0.001939	0.000330	0.012141	0.003721	0.110000
91	-3150.030430	-25.027450	0.001854	0.000324	0.011800	0.003715	0.110000
92	-3150.030473	-25.054713	0.001772	0.000318	0.011492	0.003711	0.110000
93	-3150.030519	-25.083048	0.001695	0.000312	0.011153	0.003718	0.110000
94	-3150.030560	-25.109387	0.001621	0.000307	0.010774	0.003715	0.110000
95	-3150.030601	-25.134785	0.001550	0.000302	0.010472	0.003708	0.110000
96	-3150.030640	-25.159399	0.001529	0.000297	0.010151	0.003710	0.110000
97	-3150.030678	-25.183141	0.001523	0.000292	0.009871	0.003709	0.110000
98	-3150.030715	-25.206056	0.001519	0.000287	0.009992	0.003711	0.110000
99	-3150.030750	-25.228157	0.001511	0.000283	0.010118	0.003711	0.110000
100	-3150.030784	-25.249501	0.001504	0.000278	0.010234	0.003714	0.110000
101	-3150.030816	-25.269623	0.001494	0.000274	0.010356	0.003716	0.110000
102	-3150.030848	-25.289594	0.001484	0.000270	0.010477	0.003720	0.110000
103	-3150.030878	-25.308378	0.001472	0.000265	0.010595	0.003724	0.110000
104	-3150.030907	-25.326985	0.001460	0.000261	0.010757	0.003725	0.110000
105	-3150.030937	-25.345347	0.001459	0.000257	0.010890	0.003714	0.110000
106	-3150.030963	-25.361777	0.001421	0.000251	0.011097	0.003706	0.110000
107	-3150.030989	-25.378417	0.001392	0.000246	0.011187	0.003782	0.110000
108	-3150.031015	-25.394340	0.001373	0.000242	0.011350	0.003787	0.110000
109	-3150.031039	-25.409874	0.001367	0.000239	0.011492	0.003794	0.110000
110	-3150.031063	-25.424886	0.001348	0.000235	0.011609	0.003789	0.110000
111	-3150.031088	-25.440368	0.001298	0.000228	0.011698	0.003797	0.110000
112	-3150.031110	-25.454010	0.001138	0.000211	0.011680	0.003820	0.110000
113	-3150.031133	-25.468463	0.001124	0.000204	0.012607	0.004014	0.110000
114	-3150.031154	-25.481805	0.001416	0.000239	0.013047	0.004015	0.110000
115	-3150.031176	-25.495795	0.001032	0.000192	0.011560	0.003712	0.110000
116	-3150.031196	-25.508158	0.000977	0.000173	0.013617	0.004058	0.110000
117	-3150.031216	-25.520852	0.001304	0.000218	0.014131	0.004163	0.110000
118	-3150.031236	-25.533417	0.001126	0.000193	0.012417	0.003764	0.110000
119	-3150.031253	-25.544071	0.001115	0.000195	0.012955	0.003963	0.110000
120	-3150.031271	-25.555016	0.001211	0.000207	0.013370	0.003980	0.110000
121	-3150.031289	-25.566821	0.001214	0.000206	0.012994	0.003885	0.110000
122	-3150.031306	-25.577502	0.001133	0.000196	0.012550	0.003818	0.110000
123	-3150.031321	-25.586708	0.001052	0.000184	0.012694	0.003890	0.110000
124	-3150.031337	-25.596407	0.001147	0.000197	0.013098	0.004035	0.110000
125	-3150.031352	-25.605926	0.001135	0.000194	0.012917	0.003916	0.110000
126	-3150.031368	-25.616276	0.001145	0.000195	0.012632	0.003914	0.110000
127	-3150.031384	-25.625954	0.000977	0.000175	0.012088	0.003796	0.110000
128	-3150.031398	-25.635041	0.000950	0.000170	0.012493	0.004012	0.110000
129	-3150.031412	-25.643412	0.001133	0.000196	0.013031	0.004043	0.110000
130	-3150.031427	-25.653037	0.000881	0.000158	0.012018	0.003736	0.110000
131	-3150.031439	-25.660614	0.000763	0.000153	0.012073	0.003987	0.110000
132	-3150.031452	-25.668828	0.000913	0.000162	0.013117	0.004222	0.110000
133	-3150.031462	-25.675109	0.000968	0.000168	0.013939	0.004095	0.110000
134	-3150.031476	-25.683902	0.000744	0.000134	0.011995	0.003885	0.110000
135	-3150.031488	-25.691246	0.000559	0.000113	0.012303	0.004048	0.110000
136	-3150.031496	-25.696659	0.000729	0.000144	0.012813	0.004244	0.110000
137	-3150.031506	-25.702768	0.000922	0.000185	0.013511	0.004179	0.110000
138	-3150.031518	-25.709934	0.000913	0.000168	0.011557	0.003930	0.110000
139	-3150.031530	-25.717515	0.000783	0.000135	0.010578	0.003899	0.110000
140	-3150.031530	-25.717757	0.000683	0.000125	0.010110	0.003163	0.110000
141	-3150.031538	-25.722518	0.000498	0.000115	0.014223	0.004087	0.110000
142	-3150.031545	-25.727264	0.000811	0.000154	0.014920	0.004405	0.110000
143	-3150.031555	-25.733576	0.000503	0.000100	0.009704	0.003637	0.110000
144	-3150.031561	-25.737482	0.000512	0.000084	0.010787	0.004130	0.110000
145	-3150.031571	-25.743407	0.001185	0.000202	0.011445	0.004451	0.110000
146	-3150.031569	-25.742241	0.001518	0.000237	0.011999	0.002301	0.110000
147	-3150.031575	-25.745685	0.000967	0.000169	0.013910	0.003953	0.110001
148	-3150.031580	-25.748840	0.001097	0.000169	0.009889	0.003826	0.104757
149	-3150.031588	-25.753849	0.000675	0.000144	0.011539	0.004069	0.110000
150	-3150.031593	-25.757378	0.000712	0.000142	0.012842	0.003896	0.110000
151	-3150.031595	-25.758494	0.000757	0.000137	0.008244	0.002109	0.110000
152	-3150.031600	-25.761858	0.000546	0.000127	0.009671	0.003706	0.110001

153	-3150.031601	-25.762040	0.000737	0.000152	0.008779	0.002581	0.073691
154	-3150.031605	-25.764706	0.000636	0.000140	0.009142	0.003560	0.110000
155	-3150.031606	-25.765588	0.000723	0.000157	0.008493	0.003129	0.087367
156	-3150.031607	-25.766060	0.000870	0.000209	0.009589	0.003498	0.110000
157	-3150.031610	-25.767793	0.001043	0.000205	0.014491	0.003833	0.105742
158	-3150.031613	-25.769528	0.001332	0.000219	0.008344	0.003140	0.100392
159	-3150.031615	-25.771394	0.001250	0.000207	0.012764	0.003342	0.092635
160	-3150.031618	-25.773155	0.001426	0.000223	0.008704	0.003524	0.110000
161	-3150.031621	-25.774839	0.001094	0.000197	0.010202	0.003564	0.104182
162	-3150.031622	-25.775699	0.001061	0.000208	0.014108	0.003721	0.106713
163	-3150.031624	-25.776533	0.000765	0.000207	0.012724	0.003797	0.110000
164	-3150.031627	-25.778666	0.000728	0.000182	0.014778	0.003195	0.084542
165	-3150.031628	-25.779314	0.000921	0.000233	0.013398	0.003504	0.104005
166	-3150.031631	-25.780891	0.000839	0.000228	0.011197	0.003277	0.092480
167	-3150.031631	-25.781139	0.000908	0.000253	0.014683	0.003322	0.091222
168	-3150.031633	-25.782387	0.000718	0.000214	0.009854	0.003318	0.091704
169	-3150.031635	-25.783724	0.000489	0.000141	0.010333	0.002658	0.073993
170	-3150.031640	-25.786722	0.000898	0.000189	0.011798	0.003759	0.110000
171	-3150.031643	-25.788539	0.001005	0.000187	0.008114	0.003258	0.103766
172	-3150.031644	-25.789331	0.001153	0.000237	0.011923	0.003544	0.105199
173	-3150.031646	-25.790856	0.000927	0.000205	0.007665	0.002992	0.096659
174	-3150.031650	-25.793024	0.000665	0.000172	0.011703	0.003291	0.096902
175	-3150.031653	-25.794866	0.000585	0.000137	0.008572	0.003102	0.100553
176	-3150.031655	-25.796507	0.000689	0.000145	0.012298	0.003426	0.099768
177	-3150.031656	-25.797059	0.000765	0.000186	0.009496	0.003531	0.110000
178	-3150.031657	-25.797676	0.000829	0.000211	0.013433	0.003229	0.091186
179	-3150.031659	-25.798893	0.000704	0.000200	0.008758	0.002899	0.081183
180	-3150.031661	-25.800254	0.000521	0.000142	0.009879	0.002657	0.077304
181	-3150.031667	-25.803661	0.000250	0.000076	0.008643	0.002820	0.085050
182	-3150.031672	-25.806703	0.000101	0.000033	0.013040	0.003984	0.110000
183	-3150.031676	-25.809297	0.000114	0.000027	0.014329	0.004147	0.110000
184	-3150.031680	-25.811954	0.000092	0.000025	0.014468	0.004219	0.110000
185	-3150.031685	-25.814814	0.000101	0.000025	0.014809	0.004598	0.110000
186	-3150.031688	-25.816834	0.000108	0.000030	0.013986	0.004016	0.110000
187	-3150.031693	-25.819750	0.000077	0.000023	0.014673	0.004549	0.110000
188	-3150.031696	-25.822168	0.000082	0.000026	0.014269	0.004230	0.110000
189	-3150.031701	-25.825181	0.000074	0.000021	0.014314	0.004768	0.110000
190	-3150.031705	-25.827624	0.000069	0.000021	0.014627	0.004637	0.110000
191	-3150.031709	-25.830297	0.000071	0.000023	0.013986	0.004399	0.110000
192	-3150.031707	-25.828581	0.000076	0.000021	0.014143	0.004821	0.110000
193	-3150.031715	-25.834117	0.000073	0.000017	0.013644	0.004913	0.110000
194	-3150.031723	-25.838741	0.000071	0.000017	0.014057	0.004886	0.110000
195	-3150.031728	-25.842076	0.000068	0.000019	0.014057	0.004821	0.110000
196	-3150.031729	-25.842333	0.000068	0.000019	0.014497	0.004917	0.110000
197	-3150.031738	-25.848316	0.000072	0.000018	0.014552	0.004982	0.110000
198	-3150.031742	-25.850842	0.000070	0.000019	0.013935	0.004844	0.110000
199	-3150.031744	-25.852176	0.000070	0.000019	0.014417	0.004956	0.110000
200	-3150.031751	-25.856518	0.000073	0.000018	0.014523	0.004983	0.110000
201	-3150.031759	-25.861232	0.000072	0.000018	0.014678	0.004980	0.110000
202	-3150.031764	-25.864298	0.000072	0.000018	0.014666	0.004969	0.110000
203	-3150.031768	-25.866878	0.000072	0.000019	0.014619	0.004979	0.110000
204	-3150.031775	-25.871216	0.000073	0.000019	0.014439	0.005008	0.110000
205	-3150.031781	-25.875154	0.000073	0.000019	0.013689	0.004959	0.110000
206	-3150.031782	-25.875996	0.000071	0.000021	0.013580	0.004985	0.110000
207	-3150.031786	-25.878450	0.000075	0.000020	0.013801	0.005021	0.110000
208	-3150.031796	-25.884416	0.000076	0.000018	0.013850	0.005011	0.110000
209	-3150.031802	-25.888244	0.000075	0.000019	0.013703	0.004925	0.110000
210	-3150.031807	-25.891518	0.000080	0.000025	0.013461	0.004879	0.110000
211	-3150.031813	-25.895495	0.000125	0.000035	0.013116	0.004799	0.110000
212	-3150.031819	-25.898990	0.000134	0.000041	0.012899	0.004414	0.110000
213	-3150.031819	-25.898930	0.000087	0.000027	0.006874	0.001764	0.110000
214	-3150.031824	-25.902231	0.000193	0.000046	0.012991	0.004776	0.110000
215	-3150.031831	-25.906526	0.000185	0.000040	0.013034	0.004729	0.110000

216	-3150.031831	-25.906456	0.000193	0.000035	0.013549	0.004828	0.110000
217	-3150.031835	-25.909069	0.000159	0.000032	0.013733	0.004960	0.110000
218	-3150.031844	-25.914805	0.000088	0.000023	0.013355	0.005014	0.110000
219	-3150.031852	-25.920123	0.000077	0.000022	0.013447	0.004988	0.110000
220	-3150.031857	-25.923160	0.000140	0.000038	0.013995	0.004721	0.110000
221	-3150.031863	-25.926491	0.000267	0.000050	0.013379	0.004782	0.110000
222	-3150.031867	-25.929364	0.000322	0.000054	0.013570	0.004684	0.110000
223	-3150.031874	-25.933348	0.000294	0.000046	0.013651	0.004712	0.110000
224	-3150.031881	-25.937908	0.000180	0.000033	0.013003	0.004770	0.110000
225	-3150.031885	-25.940791	0.000170	0.000037	0.012881	0.004816	0.110000
226	-3150.031893	-25.945234	0.000261	0.000057	0.013242	0.004918	0.110000
227	-3150.031898	-25.948613	0.000349	0.000065	0.012502	0.004785	0.110000
228	-3150.031904	-25.952371	0.000358	0.000063	0.012688	0.004751	0.110000
229	-3150.031909	-25.955413	0.000314	0.000061	0.011909	0.004652	0.110000
230	-3150.031915	-25.959055	0.000303	0.000059	0.011780	0.004671	0.110000
231	-3150.031920	-25.962577	0.000312	0.000061	0.011726	0.004680	0.110000
232	-3150.031926	-25.966225	0.000327	0.000064	0.011739	0.004662	0.110000
233	-3150.031932	-25.969775	0.000331	0.000065	0.011627	0.004635	0.110000
234	-3150.031937	-25.973357	0.000332	0.000065	0.011614	0.004630	0.110000
235	-3150.031943	-25.976963	0.000337	0.000066	0.011696	0.004636	0.110000
236	-3150.031949	-25.980602	0.000346	0.000067	0.011772	0.004641	0.110000
237	-3150.031955	-25.984282	0.000356	0.000068	0.011809	0.004642	0.110000
238	-3150.031961	-25.988008	0.000365	0.000070	0.011813	0.004640	0.110000
239	-3150.031967	-25.991760	0.000374	0.000071	0.011805	0.004638	0.110000
240	-3150.031973	-25.995554	0.000381	0.000072	0.011794	0.004636	0.110000

```
************************************************************
*  MAXIMUM NUMBER OF ITERATIONS REACHED - STOPPING IRC RUN  *
************************************************************
```

```
---------------------------------------------------------------
                       IRC PATH SUMMARY
---------------------------------------------------------------
```

All gradients are in Eh/Bohr.

Step	E(Eh)	dE(kcal/mol)	max(|G|)	RMS(G)	SMW-Displ.
1	-3150.031973	-25.995554	0.000381	0.000072	-26.057823
2	-3150.031967	-25.991760	0.000374	0.000071	-25.947823
3	-3150.031961	-25.988008	0.000365	0.000070	-25.837823
4	-3150.031955	-25.984282	0.000356	0.000068	-25.727823
5	-3150.031949	-25.980602	0.000346	0.000067	-25.617823
6	-3150.031943	-25.976963	0.000337	0.000066	-25.507823
7	-3150.031937	-25.973357	0.000332	0.000065	-25.397823
8	-3150.031932	-25.969775	0.000331	0.000065	-25.287823
9	-3150.031926	-25.966225	0.000327	0.000064	-25.177823
10	-3150.031920	-25.962577	0.000312	0.000061	-25.067823
11	-3150.031915	-25.959055	0.000303	0.000059	-24.957823
12	-3150.031909	-25.955413	0.000314	0.000061	-24.847823
13	-3150.031904	-25.952371	0.000358	0.000063	-24.737823
14	-3150.031898	-25.948613	0.000349	0.000065	-24.627823
15	-3150.031893	-25.945234	0.000261	0.000057	-24.517823
16	-3150.031885	-25.940791	0.000170	0.000037	-24.407822
17	-3150.031881	-25.937908	0.000180	0.000033	-24.297822
18	-3150.031874	-25.933348	0.000294	0.000046	-24.187822
19	-3150.031867	-25.929364	0.000322	0.000054	-24.077822
20	-3150.031863	-25.926491	0.000267	0.000050	-23.967822
21	-3150.031857	-25.923160	0.000140	0.000038	-23.857822
22	-3150.031852	-25.920123	0.000077	0.000022	-23.747822
23	-3150.031844	-25.914805	0.000088	0.000023	-23.637822
24	-3150.031835	-25.909069	0.000159	0.000032	-23.527822
25	-3150.031831	-25.906456	0.000193	0.000035	-23.417822
26	-3150.031831	-25.906526	0.000185	0.000040	-23.307821

27	-3150.031824	-25.902231	0.000193	0.000046	-23.197821
28	-3150.031819	-25.898930	0.000087	0.000027	-23.087821
29	-3150.031819	-25.898990	0.000134	0.000041	-22.977821
30	-3150.031813	-25.895495	0.000125	0.000035	-22.867821
31	-3150.031807	-25.891518	0.000080	0.000025	-22.757821
32	-3150.031802	-25.888244	0.000075	0.000019	-22.647821
33	-3150.031796	-25.884416	0.000076	0.000018	-22.537821
34	-3150.031786	-25.878450	0.000075	0.000020	-22.427821
35	-3150.031782	-25.875996	0.000071	0.000021	-22.317821
36	-3150.031781	-25.875154	0.000073	0.000019	-22.207821
37	-3150.031775	-25.871216	0.000073	0.000019	-22.097821
38	-3150.031768	-25.866878	0.000072	0.000019	-21.987821
39	-3150.031764	-25.864298	0.000072	0.000018	-21.877821
40	-3150.031759	-25.861232	0.000072	0.000018	-21.767821
41	-3150.031751	-25.856518	0.000073	0.000018	-21.657820
42	-3150.031744	-25.852176	0.000070	0.000019	-21.547820
43	-3150.031742	-25.850842	0.000070	0.000019	-21.437820
44	-3150.031738	-25.848316	0.000072	0.000018	-21.327820
45	-3150.031729	-25.842333	0.000068	0.000019	-21.217820
46	-3150.031728	-25.842076	0.000068	0.000019	-21.107820
47	-3150.031723	-25.838741	0.000071	0.000017	-20.997820
48	-3150.031715	-25.834117	0.000073	0.000017	-20.887819
49	-3150.031707	-25.828581	0.000076	0.000021	-20.777819
50	-3150.031709	-25.830297	0.000071	0.000023	-20.667819
51	-3150.031705	-25.827624	0.000069	0.000021	-20.557819
52	-3150.031701	-25.825181	0.000074	0.000021	-20.447819
53	-3150.031696	-25.822168	0.000082	0.000026	-20.337819
54	-3150.031693	-25.819750	0.000077	0.000023	-20.227819
55	-3150.031688	-25.816834	0.000108	0.000030	-20.117819
56	-3150.031685	-25.814814	0.000101	0.000025	-20.007819
57	-3150.031680	-25.811954	0.000092	0.000025	-19.897819
58	-3150.031676	-25.809297	0.000114	0.000027	-19.787818
59	-3150.031672	-25.806703	0.000101	0.000033	-19.677818
60	-3150.031667	-25.803661	0.000250	0.000076	-19.567818
61	-3150.031661	-25.800254	0.000521	0.000142	-19.482768
62	-3150.031659	-25.798893	0.000704	0.000200	-19.405465
63	-3150.031657	-25.797676	0.000829	0.000211	-19.324282
64	-3150.031656	-25.797059	0.000765	0.000186	-19.233096
65	-3150.031655	-25.796507	0.000689	0.000145	-19.123096
66	-3150.031653	-25.794866	0.000585	0.000137	-19.023327
67	-3150.031650	-25.793024	0.000665	0.000172	-18.922774
68	-3150.031646	-25.790856	0.000927	0.000205	-18.825872
69	-3150.031644	-25.789331	0.001153	0.000237	-18.729213
70	-3150.031643	-25.788539	0.001005	0.000187	-18.624014
71	-3150.031640	-25.786722	0.000898	0.000189	-18.520248
72	-3150.031635	-25.783724	0.000489	0.000141	-18.410248
73	-3150.031633	-25.782387	0.000718	0.000214	-18.336255
74	-3150.031631	-25.781139	0.000908	0.000253	-18.244552
75	-3150.031631	-25.780891	0.000839	0.000228	-18.153330
76	-3150.031628	-25.779314	0.000921	0.000233	-18.060850
77	-3150.031627	-25.778666	0.000728	0.000182	-17.956845
78	-3150.031624	-25.776533	0.000765	0.000207	-17.872303
79	-3150.031622	-25.775699	0.001061	0.000208	-17.762303
80	-3150.031621	-25.774839	0.001094	0.000197	-17.655590
81	-3150.031618	-25.773155	0.001426	0.000223	-17.551408
82	-3150.031615	-25.771394	0.001250	0.000207	-17.441408
83	-3150.031613	-25.769528	0.001332	0.000219	-17.348772
84	-3150.031610	-25.767793	0.001043	0.000205	-17.248380
85	-3150.031607	-25.766060	0.000870	0.000209	-17.142638
86	-3150.031606	-25.765588	0.000723	0.000157	-17.032638
87	-3150.031605	-25.764706	0.000636	0.000140	-16.945271
88	-3150.031601	-25.762040	0.000737	0.000152	-16.835271
89	-3150.031600	-25.761858	0.000546	0.000127	-16.761580

90	-3150.031595	-25.758494	0.000757	0.000137	-16.651579
91	-3150.031593	-25.757378	0.000712	0.000142	-16.541579
92	-3150.031588	-25.753849	0.000675	0.000144	-16.431579
93	-3150.031580	-25.748840	0.001097	0.000169	-16.321579
94	-3150.031575	-25.745685	0.000967	0.000169	-16.216823
95	-3150.031569	-25.742241	0.001518	0.000237	-16.106822
96	-3150.031571	-25.743407	0.001185	0.000202	-15.996821
97	-3150.031561	-25.737482	0.000512	0.000084	-15.886821
98	-3150.031555	-25.733576	0.000503	0.000100	-15.776821
99	-3150.031545	-25.727264	0.000811	0.000154	-15.666821
100	-3150.031538	-25.722518	0.000498	0.000115	-15.556821
101	-3150.031530	-25.717757	0.000683	0.000125	-15.446821
102	-3150.031530	-25.717515	0.000783	0.000135	-15.336821
103	-3150.031518	-25.709934	0.000913	0.000168	-15.226821
104	-3150.031506	-25.702768	0.000922	0.000185	-15.116821
105	-3150.031496	-25.696659	0.000729	0.000144	-15.006821
106	-3150.031488	-25.691246	0.000559	0.000113	-14.896820
107	-3150.031476	-25.683902	0.000744	0.000134	-14.786820
108	-3150.031462	-25.675109	0.000968	0.000168	-14.676820
109	-3150.031452	-25.668828	0.000913	0.000162	-14.566820
110	-3150.031439	-25.660614	0.000763	0.000153	-14.456820
111	-3150.031427	-25.653037	0.000881	0.000158	-14.346820
112	-3150.031412	-25.643412	0.001133	0.000196	-14.236819
113	-3150.031398	-25.635041	0.000950	0.000170	-14.126819
114	-3150.031384	-25.625954	0.000977	0.000175	-14.016819
115	-3150.031368	-25.616276	0.001145	0.000195	-13.906819
116	-3150.031352	-25.605926	0.001135	0.000194	-13.796819
117	-3150.031337	-25.596407	0.001147	0.000197	-13.686819
118	-3150.031321	-25.586708	0.001052	0.000184	-13.576819
119	-3150.031306	-25.577502	0.001133	0.000196	-13.466819
120	-3150.031289	-25.566821	0.001214	0.000206	-13.356819
121	-3150.031271	-25.555016	0.001211	0.000207	-13.246819
122	-3150.031253	-25.544071	0.001115	0.000195	-13.136819
123	-3150.031236	-25.533417	0.001126	0.000193	-13.026819
124	-3150.031216	-25.520852	0.001304	0.000218	-12.916818
125	-3150.031196	-25.508158	0.000977	0.000173	-12.806818
126	-3150.031176	-25.495795	0.001032	0.000192	-12.696818
127	-3150.031154	-25.481805	0.001416	0.000239	-12.586818
128	-3150.031133	-25.468463	0.001124	0.000204	-12.476818
129	-3150.031110	-25.454010	0.001138	0.000211	-12.366817
130	-3150.031088	-25.440368	0.001298	0.000228	-12.256817
131	-3150.031063	-25.424886	0.001348	0.000235	-12.146817
132	-3150.031039	-25.409874	0.001367	0.000239	-12.036817
133	-3150.031015	-25.394340	0.001373	0.000242	-11.926817
134	-3150.030989	-25.378417	0.001392	0.000246	-11.816816
135	-3150.030963	-25.361777	0.001421	0.000251	-11.706816
136	-3150.030937	-25.345347	0.001459	0.000257	-11.596816
137	-3150.030907	-25.326985	0.001460	0.000261	-11.486816
138	-3150.030878	-25.308378	0.001472	0.000265	-11.376816
139	-3150.030848	-25.289594	0.001484	0.000270	-11.266815
140	-3150.030816	-25.269623	0.001494	0.000274	-11.156815
141	-3150.030784	-25.249501	0.001504	0.000278	-11.046815
142	-3150.030750	-25.228157	0.001511	0.000283	-10.936815
143	-3150.030715	-25.206056	0.001519	0.000287	-10.826814
144	-3150.030678	-25.183141	0.001523	0.000292	-10.716814
145	-3150.030640	-25.159399	0.001529	0.000297	-10.606814
146	-3150.030601	-25.134785	0.001550	0.000302	-10.496814
147	-3150.030560	-25.109387	0.001621	0.000307	-10.386814
148	-3150.030519	-25.083048	0.001695	0.000312	-10.276814
149	-3150.030473	-25.054713	0.001772	0.000318	-10.166814
150	-3150.030430	-25.027450	0.001854	0.000324	-10.056813
151	-3150.030383	-24.998060	0.001939	0.000330	-9.946813
152	-3150.030334	-24.967185	0.002028	0.000337	-9.836813

153	-3150.030284	-24.935936	0.002121	0.000344	-9.726813
154	-3150.030232	-24.903438	0.002227	0.000353	-9.616813
155	-3150.030177	-24.868979	0.002334	0.000362	-9.506812
156	-3150.030120	-24.833084	0.002440	0.000370	-9.396812
157	-3150.030061	-24.796082	0.002552	0.000379	-9.286812
158	-3150.029998	-24.756179	0.002666	0.000391	-9.176811
159	-3150.029935	-24.716653	0.002792	0.000400	-9.066811
160	-3150.029867	-24.674361	0.002910	0.000410	-8.956811
161	-3150.029798	-24.630623	0.003040	0.000421	-8.846811
162	-3150.029724	-24.584640	0.003173	0.000433	-8.736810
163	-3150.029648	-24.536718	0.003310	0.000442	-8.626810
164	-3150.029566	-24.485212	0.003450	0.000454	-8.516810
165	-3150.029481	-24.432136	0.003598	0.000469	-8.406809
166	-3150.029397	-24.379562	0.003746	0.000489	-8.296809
167	-3150.029307	-24.322664	0.003897	0.000511	-8.186809
168	-3150.029213	-24.263583	0.004053	0.000529	-8.076808
169	-3150.029125	-24.208840	0.004220	0.000534	-7.966808
170	-3150.029014	-24.139078	0.004401	0.000548	-7.856807
171	-3150.028905	-24.070391	0.004592	0.000571	-7.746806
172	-3150.028797	-24.002510	0.005246	0.000946	-7.636805
173	-3150.028768	-23.984296	0.004846	0.000883	-7.526805
174	-3150.028676	-23.926804	0.005022	0.000806	-7.416804
175	-3150.028566	-23.857771	0.005172	0.000894	-7.306804
176	-3150.028466	-23.795105	0.005372	0.000821	-7.196803
177	-3150.028346	-23.719752	0.005538	0.000948	-7.086803
178	-3150.028240	-23.653020	0.005735	0.000885	-6.976803
179	-3150.028116	-23.575500	0.005909	0.000963	-6.866803
180	-3150.027993	-23.498503	0.006089	0.000918	-6.756803
181	-3150.027868	-23.419837	0.006331	0.000899	-6.646803
182	-3150.027722	-23.328284	0.006533	0.001057	-6.536803
183	-3150.027594	-23.247950	0.006750	0.000986	-6.426802
184	-3150.027441	-23.151745	0.006954	0.001054	-6.316802
185	-3150.027300	-23.063408	0.007207	0.001001	-6.206801
186	-3150.027133	-22.958320	0.007433	0.001137	-6.096800
187	-3150.026980	-22.862688	0.007696	0.001068	-5.986799
188	-3150.026802	-22.751190	0.007944	0.001200	-5.876799
189	-3150.026637	-22.647243	0.008197	0.001169	-5.766798
190	-3150.026453	-22.531996	0.008454	0.001236	-5.656798
191	-3150.026269	-22.416242	0.008718	0.001248	-5.546797
192	-3150.026073	-22.293225	0.008989	0.001293	-5.436796
193	-3150.025871	-22.166515	0.009267	0.001321	-5.326796
194	-3150.025659	-22.033629	0.009552	0.001361	-5.216795
195	-3150.025439	-21.895688	0.009844	0.001396	-5.106795
196	-3150.025209	-21.751574	0.010144	0.001436	-4.996794
197	-3150.024970	-21.601594	0.010450	0.001476	-4.886793
198	-3150.024721	-21.445113	0.010765	0.001518	-4.776792
199	-3150.024461	-21.282107	0.011086	0.001561	-4.666791
200	-3150.024190	-21.112090	0.011416	0.001606	-4.556789
201	-3150.023908	-20.934878	0.011753	0.001652	-4.446788
202	-3150.023614	-20.750094	0.012098	0.001701	-4.336787
203	-3150.023307	-20.557483	0.012450	0.001750	-4.226786
204	-3150.022987	-20.356648	0.012811	0.001802	-4.116786
205	-3150.022653	-20.147288	0.013180	0.001855	-4.006785
206	-3150.022305	-19.928960	0.013557	0.001910	-3.896784
207	-3150.021942	-19.701350	0.013941	0.001967	-3.786784
208	-3150.021564	-19.463916	0.014364	0.002029	-3.676783
209	-3150.021169	-19.216076	0.014779	0.002090	-3.566782
210	-3150.020758	-18.957973	0.015195	0.002153	-3.456781
211	-3150.020329	-18.688866	0.015617	0.002218	-3.346779
212	-3150.019881	-18.408216	0.016042	0.002284	-3.236779
213	-3150.019415	-18.115433	0.016471	0.002352	-3.126777
214	-3150.018929	-17.810359	0.016899	0.002424	-3.016776
215	-3150.018419	-17.490629	0.017330	0.002497	-2.906775

216	-3150.017890	-17.158237	0.017750	0.002532	-2.796772
217	-3150.017323	-16.802489	0.018193	0.002594	-2.686771
218	-3150.016728	-16.429215	0.018655	0.002675	-2.576768
219	-3150.016107	-16.039552	0.019126	0.002764	-2.466767
220	-3150.015462	-15.634772	0.019600	0.002859	-2.356767
221	-3150.014792	-15.214375	0.020071	0.002958	-2.246766
222	-3150.014095	-14.777316	0.020537	0.003062	-2.136763
223	-3150.013369	-14.321887	0.020993	0.003170	-2.026763
224	-3150.012617	-13.849702	0.021437	0.003283	-1.916760
225	-3150.011836	-13.359834	0.021861	0.003403	-1.806758
226	-3150.011021	-12.848198	0.022260	0.003529	-1.696755
227	-3150.010171	-12.314885	0.022619	0.003663	-1.586752
228	-3150.009284	-11.757925	0.022928	0.003806	-1.476749
229	-3150.008353	-11.174280	0.023172	0.003964	-1.366746
230	-3150.007373	-10.559175	0.023336	0.004139	-1.256746
231	-3150.006333	-9.906733	0.023405	0.004333	-1.146745
232	-3150.005226	-9.211971	0.023363	0.004552	-1.036745
233	-3150.004030	-8.461524	0.025074	0.004795	-0.926741
234	-3150.002726	-7.642913	0.026782	0.005058	-0.816737
235	-3150.001278	-6.734289	0.027944	0.005305	-0.706734
236	-3149.999610	-5.687528	0.027606	0.005436	-0.596731
237	-3149.997539	-4.388051	0.024950	0.005314	-0.486728
238	-3149.995125	-2.873499	0.020828	0.004779	-0.376724
239	-3149.992817	-1.424814	0.015594	0.003660	-0.266722
240	-3149.991235	-0.432037	0.008168	0.002030	-0.156721
241	-3149.990596	-0.031354	0.002260	0.000530	-0.046716
242	-3149.990546	0.000000	0.000024	0.000006	0.000000 <= TS
243	-3149.990592	-0.028848	0.002311	0.000492	0.046716
244	-3149.990966	-0.263785	0.007352	0.001416	0.156717
245	-3149.991542	-0.625224	0.011322	0.002052	0.266719
246	-3149.992192	-1.032739	0.013938	0.002453	0.376720
247	-3149.992876	-1.461762	0.015582	0.002714	0.486720
248	-3149.993589	-1.909773	0.016613	0.002896	0.596722
249	-3149.994321	-2.369039	0.017736	0.003023	0.706725
250	-3149.995067	-2.836776	0.018892	0.003130	0.816726
251	-3149.995825	-3.312782	0.019885	0.003218	0.926728
252	-3149.996597	-3.796704	0.020757	0.003294	1.036730
253	-3149.997377	-4.286192	0.021533	0.003360	1.146733
254	-3149.998165	-4.780788	0.022229	0.003419	1.256733
255	-3149.998962	-5.280837	0.022859	0.003472	1.366737
256	-3149.999766	-5.785290	0.023426	0.003520	1.476737
257	-3150.000579	-6.295473	0.023936	0.003562	1.586738
258	-3150.001392	-6.805972	0.024383	0.003598	1.696740
259	-3150.002211	-7.320040	0.024770	0.003629	1.806741
260	-3150.003037	-7.838440	0.025093	0.003653	1.916743
261	-3150.003863	-8.356505	0.025347	0.003670	2.026744
262	-3150.004691	-8.876341	0.025531	0.003680	2.136745
263	-3150.005520	-9.396070	0.025641	0.003682	2.246748
264	-3150.006354	-9.919820	0.025680	0.003675	2.356749
265	-3150.007169	-10.430887	0.025631	0.003658	2.466752
266	-3150.007985	-10.942967	0.025507	0.003631	2.576752
267	-3150.008792	-11.449176	0.025304	0.003593	2.686755
268	-3150.009585	-11.946783	0.025023	0.003546	2.796757
269	-3150.010358	-12.432303	0.024665	0.003492	2.906758
270	-3150.011110	-12.904124	0.024219	0.003427	3.016758
271	-3150.011842	-13.363397	0.023682	0.003349	3.126760
272	-3150.012555	-13.810507	0.023065	0.003261	3.236762
273	-3150.013244	-14.243141	0.022373	0.003165	3.346764
274	-3150.013908	-14.660113	0.021605	0.003059	3.456766
275	-3150.014545	-15.059484	0.020762	0.002945	3.566767
276	-3150.015153	-15.440942	0.019846	0.002823	3.676768
277	-3150.015731	-15.803584	0.018860	0.002693	3.786768
278	-3150.016277	-16.146677	0.017808	0.002555	3.896769

279	-3150.016792	-16.469674	0.016695	0.002412	4.006771
280	-3150.017274	-16.772145	0.015527	0.002262	4.116773
281	-3150.017723	-17.053621	0.014313	0.002109	4.226774
282	-3150.018139	-17.314689	0.013063	0.001953	4.336775
283	-3150.018522	-17.555001	0.011796	0.001797	4.446775
284	-3150.018874	-17.775927	0.010519	0.001639	4.556777
285	-3150.019193	-17.976272	0.009260	0.001492	4.666779
286	-3150.019489	-18.161761	0.008019	0.001329	4.776781
287	-3150.019742	-18.320924	0.006871	0.001245	4.886782
288	-3150.020001	-18.483344	0.006213	0.001024	4.996784
289	-3150.020190	-18.602129	0.005698	0.001235	5.106786
290	-3150.020430	-18.752724	0.005228	0.000801	5.216789
291	-3150.020625	-18.874800	0.004676	0.000758	5.326792
292	-3150.020807	-18.989193	0.004199	0.000675	5.436792
293	-3150.020981	-19.098176	0.003772	0.000603	5.546793
294	-3150.021144	-19.200594	0.003399	0.000562	5.656793
295	-3150.021305	-19.301175	0.003088	0.000537	5.766794
296	-3150.021462	-19.399827	0.002828	0.000517	5.876794
297	-3150.021618	-19.498192	0.002613	0.000497	5.986795
298	-3150.021775	-19.596612	0.002436	0.000478	6.096796
299	-3150.021931	-19.694123	0.002291	0.000464	6.206796
300	-3150.022085	-19.790860	0.002170	0.000463	6.316797
301	-3150.022238	-19.887065	0.002070	0.000473	6.426798
302	-3150.022389	-19.981913	0.001988	0.000484	6.536799
303	-3150.022539	-20.076028	0.001921	0.000490	6.646800
304	-3150.022690	-20.170826	0.001867	0.000487	6.756800
305	-3150.022843	-20.266640	0.001823	0.000480	6.866801
306	-3150.022996	-20.362727	0.001786	0.000476	6.976801
307	-3150.023148	-20.458275	0.001752	0.000479	7.086801
308	-3150.023301	-20.553837	0.001786	0.000486	7.196802
309	-3150.023451	-20.648192	0.001835	0.000490	7.306802
310	-3150.023601	-20.742092	0.001863	0.000492	7.416802
311	-3150.023749	-20.835262	0.001886	0.000493	7.526803
312	-3150.023896	-20.927514	0.001921	0.000494	7.636804
313	-3150.024042	-21.018751	0.001955	0.000495	7.746804
314	-3150.024185	-21.108792	0.001991	0.000497	7.856804
315	-3150.024326	-21.197398	0.002023	0.000496	7.966804
316	-3150.024465	-21.284398	0.002038	0.000494	8.076805
317	-3150.024601	-21.369570	0.002094	0.000496	8.186805
318	-3150.024734	-21.453248	0.002135	0.000497	8.296805
319	-3150.024865	-21.535524	0.002161	0.000496	8.406806
320	-3150.024994	-21.616230	0.002193	0.000496	8.516807
321	-3150.025120	-21.695546	0.002219	0.000496	8.626807
322	-3150.025244	-21.773440	0.002241	0.000495	8.736808
323	-3150.025366	-21.849861	0.002261	0.000495	8.846808
324	-3150.025486	-21.924805	0.002275	0.000494	8.956808
325	-3150.025603	-21.998249	0.002283	0.000493	9.066809
326	-3150.025717	-22.070129	0.002282	0.000492	9.176810
327	-3150.025829	-22.140401	0.002267	0.000490	9.286810
328	-3150.025938	-22.209032	0.002233	0.000488	9.396810
329	-3150.026045	-22.276022	0.002168	0.000485	9.506811
330	-3150.026149	-22.341374	0.002060	0.000482	9.616811
331	-3150.026251	-22.404941	0.001907	0.000480	9.726812
332	-3150.026350	-22.467333	0.001746	0.000472	9.836813
333	-3150.026446	-22.527570	0.001874	0.000470	9.946813
334	-3150.026532	-22.581159	0.001939	0.000467	10.056814
335	-3150.026620	-22.636724	0.001959	0.000461	10.166814
336	-3150.026704	-22.689614	0.002002	0.000468	10.276815
337	-3150.026786	-22.740878	0.001919	0.000452	10.386815
338	-3150.026863	-22.788876	0.001997	0.000466	10.496816
339	-3150.026940	-22.837211	0.001753	0.000422	10.606816
340	-3150.027006	-22.878663	0.001930	0.000474	10.716817
341	-3150.027087	-22.929807	0.001274	0.000356	10.826817

342	-3150.027145	-22.966310	0.002231	0.000415	10.936817
343	-3150.027219	-23.012318	0.001449	0.000309	11.046818
344	-3150.027277	-23.048769	0.001668	0.000396	11.156818
345	-3150.027340	-23.088440	0.001241	0.000317	11.266818
346	-3150.027370	-23.107234	0.003828	0.000661	11.376819
347	-3150.027396	-23.123813	0.002663	0.000481	11.486820
348	-3150.027439	-23.150759	0.002337	0.000512	11.596821
349	-3150.027507	-23.193173	0.000962	0.000210	11.706822
350	-3150.027552	-23.221777	0.001468	0.000394	11.816822
351	-3150.027603	-23.253788	0.000999	0.000290	11.926823
352	-3150.027649	-23.282164	0.000873	0.000244	12.036824
353	-3150.027687	-23.306295	0.001506	0.000325	12.146824
354	-3150.027728	-23.331668	0.001482	0.000291	12.256824
355	-3150.027765	-23.355088	0.001463	0.000279	12.366824
356	-3150.027800	-23.377372	0.001550	0.000292	12.476825
357	-3150.027835	-23.398894	0.001562	0.000300	12.586825
358	-3150.027868	-23.419951	0.001515	0.000301	12.696825
359	-3150.027901	-23.440408	0.001481	0.000298	12.806826
360	-3150.027933	-23.460470	0.001509	0.000293	12.916826
361	-3150.027964	-23.479925	0.001556	0.000288	13.026826
362	-3150.027994	-23.498878	0.001572	0.000286	13.136826
363	-3150.028024	-23.517722	0.001559	0.000291	13.246826
364	-3150.028053	-23.536139	0.001525	0.000297	13.356826
365	-3150.028082	-23.554155	0.001493	0.000303	13.466826
366	-3150.028111	-23.572232	0.001478	0.000302	13.576826
367	-3150.028129	-23.583707	0.001485	0.000297	13.686827
368	-3150.028167	-23.607298	0.001484	0.000282	13.796827
369	-3150.028194	-23.624212	0.001519	0.000283	13.906827
370	-3150.028210	-23.634128	0.001577	0.000283	14.016827
371	-3150.028246	-23.656943	0.001563	0.000276	14.126827
372	-3150.028271	-23.672416	0.001625	0.000295	14.236827
373	-3150.028297	-23.688878	0.001561	0.000282	14.346827
374	-3150.028313	-23.698955	0.001610	0.000290	14.456827
375	-3150.028348	-23.720823	0.001577	0.000275	14.566827
376	-3150.028371	-23.735732	0.001628	0.000297	14.676827
377	-3150.028397	-23.751846	0.001540	0.000285	14.786827
378	-3150.028421	-23.766804	0.001564	0.000303	14.896827
379	-3150.028446	-23.782726	0.001528	0.000294	15.006828
380	-3150.028470	-23.797824	0.001602	0.000301	15.116828
381	-3150.028495	-23.813312	0.001605	0.000289	15.226828
382	-3150.028518	-23.827973	0.001688	0.000296	15.336828
383	-3150.028543	-23.843369	0.001645	0.000288	15.446828
384	-3150.028566	-23.858064	0.001676	0.000296	15.556828
385	-3150.028576	-23.864001	0.001226	0.000255	15.666828
386	-3150.028601	-23.880082	0.000473	0.000143	15.776829
387	-3150.028629	-23.897126	0.000689	0.000163	15.886829
388	-3150.028651	-23.910887	0.001060	0.000222	15.996830
389	-3150.028676	-23.926548	0.000999	0.000227	16.106830
390	-3150.028701	-23.942287	0.000914	0.000230	16.216830
391	-3150.028725	-23.957423	0.000944	0.000256	16.326830
392	-3150.028748	-23.972336	0.001373	0.000289	16.436830
393	-3150.028772	-23.986985	0.001973	0.000324	16.546830
394	-3150.028796	-24.001844	0.002139	0.000323	16.656831
395	-3150.028821	-24.017774	0.001399	0.000228	16.766831
396	-3150.028842	-24.031008	0.000896	0.000282	16.876831
397	-3150.028867	-24.046933	0.001009	0.000261	16.986831
398	-3150.028891	-24.061917	0.000963	0.000272	17.096831
399	-3150.028912	-24.075086	0.001716	0.000315	17.206831
400	-3150.028936	-24.089934	0.001426	0.000256	17.316831
401	-3150.028958	-24.103676	0.001672	0.000291	17.426831
402	-3150.028966	-24.109025	0.001681	0.000303	17.536832
403	-3150.028988	-24.122909	0.000916	0.000220	17.646833
404	-3150.029008	-24.135372	0.001821	0.000275	17.756833

405	-3150.029030	-24.148772	0.000969	0.000240	17.866833
406	-3150.029053	-24.163713	0.001152	0.000252	17.976834
407	-3150.029076	-24.177910	0.000998	0.000245	18.086834
408	-3150.029098	-24.191875	0.000961	0.000234	18.196834
409	-3150.029121	-24.206197	0.000947	0.000230	18.306834
410	-3150.029143	-24.220148	0.000947	0.000230	18.416834
411	-3150.029166	-24.234277	0.000944	0.000228	18.526834
412	-3150.029188	-24.248109	0.000966	0.000229	18.636834
413	-3150.029210	-24.261741	0.000992	0.000236	18.746835
414	-3150.029230	-24.274318	0.000986	0.000242	18.856835
415	-3150.029251	-24.287581	0.000992	0.000246	18.966835
416	-3150.029272	-24.300891	0.000998	0.000245	19.076835
417	-3150.029293	-24.314135	0.000965	0.000241	19.186835
418	-3150.029314	-24.327390	0.000962	0.000235	19.296835
419	-3150.029336	-24.341228	0.000951	0.000230	19.406835
420	-3150.029357	-24.353892	0.000946	0.000229	19.516836
421	-3150.029378	-24.367429	0.000911	0.000222	19.626836
422	-3150.029397	-24.379462	0.000901	0.000222	19.736836
423	-3150.029419	-24.393158	0.000908	0.000224	19.846836
424	-3150.029438	-24.404836	0.000943	0.000229	19.956836
425	-3150.029459	-24.418242	0.000961	0.000233	20.066836
426	-3150.029479	-24.430826	0.000985	0.000237	20.176836
427	-3150.029498	-24.442639	0.000967	0.000233	20.286837
428	-3150.029519	-24.456126	0.000945	0.000232	20.396837
429	-3150.029538	-24.467925	0.000929	0.000225	20.506837
430	-3150.029559	-24.480888	0.000853	0.000220	20.616837
431	-3150.029578	-24.493170	0.000874	0.000226	20.726837
432	-3150.029598	-24.505632	0.000863	0.000219	20.836837
433	-3150.029624	-24.521584	0.000878	0.000224	20.946837
434	-3150.029637	-24.529880	0.000752	0.000189	21.056837
435	-3150.029657	-24.542678	0.000799	0.000207	21.166837
436	-3150.029676	-24.554307	0.000877	0.000237	21.276837
437	-3150.029696	-24.566846	0.000763	0.000201	21.386838
438	-3150.029715	-24.579133	0.000863	0.000214	21.496838
439	-3150.029733	-24.590431	0.000968	0.000239	21.606838
440	-3150.029753	-24.602766	0.000888	0.000221	21.716838
441	-3150.029770	-24.613463	0.000888	0.000226	21.826838
442	-3150.029790	-24.625778	0.000909	0.000217	21.936838
443	-3150.029808	-24.636917	0.000965	0.000223	22.046838
444	-3150.029826	-24.648370	0.000895	0.000211	22.156838
445	-3150.029843	-24.659360	0.000945	0.000230	22.266838
446	-3150.029858	-24.668626	0.000841	0.000201	22.376838
447	-3150.029877	-24.680724	0.000980	0.000229	22.486839
448	-3150.029895	-24.691859	0.000929	0.000206	22.596839
449	-3150.029911	-24.702030	0.000983	0.000202	22.706839
450	-3150.029927	-24.712171	0.001087	0.000231	22.816839
451	-3150.029945	-24.722904	0.000976	0.000205	22.926839
452	-3150.029959	-24.732238	0.001006	0.000220	23.036839
453	-3150.029976	-24.742522	0.001005	0.000215	23.146839
454	-3150.029991	-24.751718	0.001017	0.000219	23.256839
455	-3150.030006	-24.761458	0.001051	0.000216	23.366840
456	-3150.030021	-24.770730	0.001029	0.000214	23.476840
457	-3150.030035	-24.779867	0.001124	0.000226	23.586840
458	-3150.030052	-24.789994	0.000917	0.000187	23.696840
459	-3150.030065	-24.798513	0.001114	0.000225	23.806840
460	-3150.030081	-24.808528	0.000905	0.000184	23.916840
461	-3150.030094	-24.816515	0.001130	0.000224	24.026840
462	-3150.030110	-24.826392	0.000934	0.000188	24.136840
463	-3150.030122	-24.834380	0.001132	0.000222	24.246840
464	-3150.030137	-24.843621	0.000992	0.000194	24.356841
465	-3150.030149	-24.850933	0.001211	0.000228	24.466841
466	-3150.030152	-24.852867	0.000995	0.000203	24.576841
467	-3150.030167	-24.862591	0.000736	0.000139	24.686843

468	-3150.030179	-24.870137	0.000630	0.000161	24.796843
469	-3150.030193	-24.879017	0.000390	0.000097	24.906843
470	-3150.030208	-24.887981	0.000520	0.000121	25.016843
471	-3150.030217	-24.893683	0.001134	0.000285	25.126844
472	-3150.030217	-24.893684	0.001251	0.000303	25.236844
473	-3150.030234	-24.904599	0.000681	0.000158	25.346845
474	-3150.030249	-24.913790	0.000245	0.000070	25.456846
475	-3150.030262	-24.922176	0.000388	0.000104	25.566846
476	-3150.030274	-24.929879	0.000662	0.000161	25.676846
477	-3150.030285	-24.936256	0.000900	0.000231	25.786846
478	-3150.030301	-24.946377	0.000316	0.000088	25.896846
479	-3150.030307	-24.950070	0.000536	0.000144	26.006846
480	-3150.030315	-24.955647	0.001013	0.000237	26.116846
481	-3150.030328	-24.963730	0.000262	0.000065	26.226846
482	-3150.030339	-24.970269	0.000814	0.000167	26.336847
483	-3150.030345	-24.974167	0.000986	0.000220	26.446847

SUGGESTED CITATIONS FOR THIS RUN

Below you find a list of papers that are relevant to this ORCA run
We neither can nor want to force you to cite these papers, but we appreciate if you do
You receive ORCA, which is the product of decades of hard work by many enthusiastic individuals, for free
The only thing we kindly ask in return is that you cite our papers,
We deeply appreciate it, if you show your appreciation for ORCA by not just citing the generic ORCA reference.

Please note that relegating all ORCA citations to the supporting information does *not* help us.
SI sections are not indexed - citations you put there will not count into any citation statistics
But we need these citations in order to attract the funding resources that allow us to do what we are doing

Therefore, if you are a happy ORCA user, please consider citing a few of the papers listed below in the main body of your paper

In addition to the list printed below, the program has created the file CN-IRC.bibtex that contains the list in bibtex format
You can import this file easily into all common literature databanks and citation aid programs

It goes without saying that in many instances, there are alternative algorithms to achieve similar
results as the ones you have gotten from ORCA. It is, of course, also the case that in some instances
ORCA just re-implements algorithms worked out by others. We are fully aware of that and we are also
fully appreciative of our colleagues work. Hence this citation list should not be read as indicating
that the listed papers, which are focused on our own work, are the only ones worth citing. It simply
meant to make it easier for users to cite ORCA specific papers. It is not a substitute for doing your
own literature research and citing the relevant literature in a scientifically appropriate manner.

List of essential papers. We consider these as the minimum necessary citations

1. Neese, F.
   Software update: the ORCA program system, version 6.0
   WIRES Comput. Molec. Sci. 2025 15(1), e70019

doi.org/10.1002/wcms.70019

List of papers to cite with high priority. The work reported in these papers was absolutely necessary for this run to complete.
Our perspective: the developers of density functionals and basis sets usually get cited in chemistry papers
Good! But without the algorithms to do something with them, the functionals or basis sets would not do anything.
Hence, in our opinion, the algorithm design and method developments papers are equally worthy of getting cited

1. Page, M.; Doubleday, C.; McIver, J.
   Following steepest descent reaction paths. The use of higher energy derivatives with abinitio electronic structure methods
   J. Chem. Phys. 1990 93(8), 5634-5642
   doi.org/10.1063/1.459634
2. Neese, F.
   An improvement of the resolution of the identity approximation for the formation of the Coulomb matrix
   J. Comp. Chem. 2003 24(14), 1740-1747
   doi.org/10.1002/jcc.10318
3. Hratchian, H.; Schlegel, B.
   Using Hessian Updating To Increase the Efficiency of a Hessian Based Predictor-Corrector Reaction Path Following Method
   J. Chem. Theory Comput. 2005 1(1), 61-69
   doi.org/10.1021/ct0499783
4. Neese, F.; Wennmohs, F.; Hansen, A.; Becker, U.
   Efficient, approximate and parallel Hartree-Fock and hybrid DFT calculations. A 'chain-of-spheres' algorithm for the Hartree-Fock exchange
   Chem. Phys. 2009 356(1-3), 98-109
   doi.org/10.1016/j.chemphys.2008.10.036
5. Grimme, S.; Antony, J.; Ehrlich, S.; Krieg, H.
   A consistent and accurate ab initio parametrization of density functional dispersion correction (DFT-D) for the 94 elements H-Pu
   J. Chem. Phys. 2010 132 , 154104
   doi.org/10.1063/1.3382344
6. Grimme, S.; Ehrlich, S.; Goerigk, L.
   Effect of the damping function in dispersion corrected density functional theory
   J. Comput. Chem. 2011 32(7), 1456-1465
   doi.org/10.1002/jcc.21759
7. Garcia-Rates, M.; Neese, F.
   Effect of the Solute Cavity on the Solvation Energy and its Derivatives within the Framework of the Gaussian Charge Scheme
   J. Comput. Chem. 2020 41 , 922-939
   doi.org/10.1002/jcc.26139
8. Helmich-Paris, B.; de Souza, B.; Neese, F.; Izsák, R.
   An improved chain of spheres for exchange algorithm
   J. Chem. Phys. 2021 155(10), 104109
   doi.org/10.1063/5.0058766
9. Neese, F.
   The SHARK Integral Generation and Digestion System
   J. Comp. Chem. 2022 44(3), 381
   doi.org/10.1002/jcc.26942

List of suggested additional citations. These are papers that are important in the 'surrounding' of
of this run, or papers that preceded the highly important papers. If you like your results we are grateful for a citation.

1. Izsak, R.; Neese, F.
   An overlap fitted chain of spheres exchange method
   J. Chem. Phys. 2011 135 , 144105
   doi.org/10.1063/1.3646921

2. Izsak, R.; Hansen, A.; Neese, F.
The resolution of identity and chain of spheres approximations for the LPNO-CCSD singles Fock term
Molec. Phys. 2012 110 , 2413-2417
doi.org/10.1080/00268976.2012.687466
3. Neese, F.
The ORCA program system
WIRES Comput. Molec. Sci. 2012 2(1), 73-78
doi.org/10.1002/wcms.81
4. Izsak, R.; Neese, F.; Klopper, W.
Robust fitting techniques in the chain of spheres approximation to the Fock exchange: The role of the complementary space
J. Chem. Phys. 2013 139 ,
doi.org/10.1063/1.4819264
5. Neese, F.
Software update: the ORCA program system, version 4.0
WIRES Comput. Molec. Sci. 2018 8(1), 1-6
doi.org/10.1002/wcms.1327
6. Neese, F.; Wennmohs, F.; Becker, U.; Riplinger, C.
The ORCA quantum chemistry program package
J. Chem. Phys. 2020 152(22), 224108
doi.org/10.1063/5.0004608
7. Neese, F.
Software update: The ORCA program system—Version 5.0
WIRES Comput. Molec. Sci. 2022 12(1), e1606
doi.org/10.1002/wcms.1606

List of optional additional citations

1. Neese, F.
Approximate second-order SCF convergence for spin unrestricted wavefunctions
Chem. Phys. Lett. 2000 325(1-3), 93-98
doi.org/10.1016/s0009-2614(00)00662-x

```
Timings for individual modules:

Sum of individual times          ...   131035.581 sec (=2183.926 min)
Startup calculation              ...     3249.154 sec (=  54.153 min)   2.5 %
SCF iterations                   ...    78536.072 sec (=1308.935 min)  59.9 %
Property calculations            ...        0.430 sec (=   0.007 min)   0.0 %
SCF Gradient evaluation          ...    49249.924 sec (= 820.832 min)  37.6 %
                             ****ORCA TERMINATED NORMALLY****
TOTAL RUN TIME: 1 days 12 hours 41 minutes 1 seconds 858 msec
'CN-IRC.bibtex' -> '/hsnfs/users/barasits/Tunneling_selenoxide/CN/IRC/CN-IRC.bibtex'
'CN-IRC.cpcm' -> '/hsnfs/users/barasits/Tunneling_selenoxide/CN/IRC/CN-IRC.cpcm'
'CN-IRC.cpcm_corr' -> '/hsnfs/users/barasits/Tunneling_selenoxide/CN/IRC/CN-IRC.cpcm_corr'
'CN-IRC.densities' -> '/hsnfs/users/barasits/Tunneling_selenoxide/CN/IRC/CN-IRC.densities'
'CN-IRC.densitiesinfo' -> '/hsnfs/users/barasits/Tunneling_selenoxide/CN/IRC/CN-IRC.densitiesinfo'
'CN-IRC.engrad' -> '/hsnfs/users/barasits/Tunneling_selenoxide/CN/IRC/CN-IRC.engrad'
'CN-IRC.err' -> '/hsnfs/users/barasits/Tunneling_selenoxide/CN/IRC/CN-IRC.err'
'CN-IRC.gbw' -> '/hsnfs/users/barasits/Tunneling_selenoxide/CN/IRC/CN-IRC.gbw'
'CN-IRC_IRC_B.cpcm' -> '/hsnfs/users/barasits/Tunneling_selenoxide/CN/IRC/CN-IRC_IRC_B.cpcm'
'CN-IRC_IRC_B.cpcm_corr' -> '/hsnfs/users/barasits/Tunneling_selenoxide/CN/IRC/CN-IRC_IRC_B.cpcm_corr'
'CN-IRC_IRC_B.densities' -> '/hsnfs/users/barasits/Tunneling_selenoxide/CN/IRC/CN-IRC_IRC_B.densities'
'CN-IRC_IRC_B.densitiesinfo' -> '/hsnfs/users/barasits/Tunneling_selenoxide/CN/IRC/CN-IRC_IRC_B.densitiesinfo'
'CN-IRC_IRC_B.gbw' -> '/hsnfs/users/barasits/Tunneling_selenoxide/CN/IRC/CN-IRC_IRC_B.gbw'
'CN-IRC_IRC_B.smd' -> '/hsnfs/users/barasits/Tunneling_selenoxide/CN/IRC/CN-IRC_IRC_B.smd'
'CN-IRC_IRC_B.smd.grd' -> '/hsnfs/users/barasits/Tunneling_selenoxide/CN/IRC/CN-IRC_IRC_B.smd.grd'
```

```
'CN-IRC_IRC_B_trj.xyz' -> '/hsnfs/users/barasits/Tunneling_selenoxide/CN/IRC/CN-
IRC_IRC_B_trj.xyz'
'CN-IRC_IRC_B.xyz' -> '/hsnfs/users/barasits/Tunneling_selenoxide/CN/IRC/CN-IRC_IRC_B.xyz'
'CN-IRC_IRC_F.cpcm' -> '/hsnfs/users/barasits/Tunneling_selenoxide/CN/IRC/CN-IRC_IRC_F.cpcm'
'CN-IRC_IRC_F.cpcm_corr' -> '/hsnfs/users/barasits/Tunneling_selenoxide/CN/IRC/CN-
IRC_IRC_F.cpcm_corr'
'CN-IRC_IRC_F.densities' -> '/hsnfs/users/barasits/Tunneling_selenoxide/CN/IRC/CN-
IRC_IRC_F.densities'
'CN-IRC_IRC_F.densitiesinfo' -> '/hsnfs/users/barasits/Tunneling_selenoxide/CN/IRC/CN-
IRC_IRC_F.densitiesinfo'
'CN-IRC_IRC_F.gbw' -> '/hsnfs/users/barasits/Tunneling_selenoxide/CN/IRC/CN-IRC_IRC_F.gbw'
'CN-IRC_IRC_F.smd' -> '/hsnfs/users/barasits/Tunneling_selenoxide/CN/IRC/CN-IRC_IRC_F.smd'
'CN-IRC_IRC_F.smd.grd' -> '/hsnfs/users/barasits/Tunneling_selenoxide/CN/IRC/CN-
IRC_IRC_F.smd.grd'
'CN-IRC_IRC_F_trj.xyz' -> '/hsnfs/users/barasits/Tunneling_selenoxide/CN/IRC/CN-
IRC_IRC_F_trj.xyz'
'CN-IRC_IRC_Full_trj.xyz' -> '/hsnfs/users/barasits/Tunneling_selenoxide/CN/IRC/CN-
IRC_IRC_Full_trj.xyz'
'CN-IRC_IRC_F.xyz' -> '/hsnfs/users/barasits/Tunneling_selenoxide/CN/IRC/CN-IRC_IRC_F.xyz'
'CN-IRC.job' -> '/hsnfs/users/barasits/Tunneling_selenoxide/CN/IRC/CN-IRC.job'
'CN-IRC.property.txt' -> '/hsnfs/users/barasits/Tunneling_selenoxide/CN/IRC/CN-
IRC.property.txt'
'CN-IRC.smd' -> '/hsnfs/users/barasits/Tunneling_selenoxide/CN/IRC/CN-IRC.smd'
'CN-IRC.smd.grd' -> '/hsnfs/users/barasits/Tunneling_selenoxide/CN/IRC/CN-IRC.smd.grd'
'TS-CN_Compound_2.hess' -> '/hsnfs/users/barasits/Tunneling_selenoxide/CN/IRC/TS-
CN_Compound_2.hess'
cp: cannot stat 'CN-IRC.asa.inp': No such file or directory
cp: missing destination file operand after '/hsnfs/users/barasits/Tunneling_selenoxide/CN/IRC/'
Try 'cp --help' for more information.
cp: cannot stat 'CN-IRC.pc.tmp': No such file or directory
cp: cannot stat 'CN-IRC.QM2.pc.tmp': No such file or directory
```
