# Supplementary material for: Identifying Quantum Tunneling at Ambient Temperature through Rate–Driving Force Responsiveness
Source: J Am Chem Soc. 2026 Aug 7;148(32):34038–44. doi: 10.1021/jacs.6c08355 (PMC13495758; doi:10.1021/jacs.6c08355)
Supplement: Supplementary file 2 [file ja6c08355_si_002.pdf]

Identifying Quantum Tunneling at Ambient Temperature through Rate–Driving Force Responsiveness

Flóra Barasits[1], Guanqi Qiu[1]*

[1]*Max-Planck-Institut für Kohlenforschung, Kaiser-Wilhelm-Platz 1, 45470, Mülheim an der Ruhr, Germany*

*Corresponding author. Email: gqiu@kofo.mpg.de

Supporting Information

Table of Contents

General Information 3
Synthetic procedures 3
Synthesis of 3-phenylpropyl 4-methylbenzenesulfonate [3] 3
General procedure for synthesis of selenides [3,5] 4
Measurement of selenoxide elimination reaction rates 7
Responsiveness analysis 10
Construction of the Experimental Brønsted plot: 10
Construction of the predicted thermal Brønsted plot: 10
Formal derivation of amplified observed responsiveness as a result of tunneling contribution: 11
Computational methods 13
TS (transition state) calculation full input: 13
IRC (intrinsic reaction coordinate) calculation full input: 16
Determination of tP (tunneling product) and IRC point-geometry correlation check 18
Computational results for constructing the Brønsted plot 19
Conceptual summary of responsiveness analysis 21
Evaluation of other non-classical effects in responsiveness analysis 24
NMR of selenides 29
(4-methoxyphenyl)(3-phenylpropyl)selane (**1a**) 29
(3-phenylpropyl)(p-tolyl)selane (**1b**) 30
phenyl(3-phenylpropyl)selane (**1c**) 31
(4-chlorophenyl)(3-phenylpropyl)selane (**1d**) 32
(3-phenylpropyl)(4-(trifluoromethyl)phenyl)selane (**1e**) 33
4-((3-phenylpropyl)selanyl)benzonitrile (**1f**) 35
Computational coordinates 36
IRC point 483 (selenoxides) 36
IRC point 242 (TS) 39
IRC point 226 (tunneling product) 42
References: 45

General Information

Reactions were carried out under air at 21 °C, unless otherwise specified. Materials purchased from commercial suppliers were used without further purification. DMSO was dried on an activated alumina column, degassed and stored over 4Å molecular sieves. Reactions were monitored by thin layer chromatography (TLC) on 0.2 mm silica gel 60 coated polyester sheets, which were visualized under 254 nm UV light. Purification via column chromatography was performed on 40-63 µm silica gel using a mixture of pentane and ethyl acetate as eluents.

^1H, ^{13}C and ^{19}F NMR spectra were recorded on a Bruker 300 MHz NMR spectrometer in $CDCl_3$, THF-d_8 or $(CD_3)_2SO$. ^1H and ^{13}C chemical shifts were determined relative to the solvent residual signals ($CDCl_3$: 7.26 ppm (^1H); $(CD_3)_2SO$: 2.50 ppm (^1H), 39.5 ppm (^{13}C); THF-d_8: 3.58 ppm (^1H)). ^{19}F chemical shift was determined without a reference. Multiplet patterns are abbreviated as s: singlet, d: doublet, t: triplet, q: quartet, m: multiplet and their combinations. High-resolution mass spectra (HRMS) were recorded on Q Exactive GC Orbitrap mass spectrometer applying electron ionization on MS90 ZB-1 HT column.

Transition state and single point calculations were performed in ORCA 6.1. IRC (intrinsic reaction coordinate) calculations were performed in ORCA 6.1 current version (2025 December)[1,2]. Energies are calculated for 298 K at 1 atm. SMD solvation in tetrahydrofuran was used in all cases.

Synthetic procedures

Synthesis of 3-phenylpropyl 4-methylbenzenesulfonate [3]

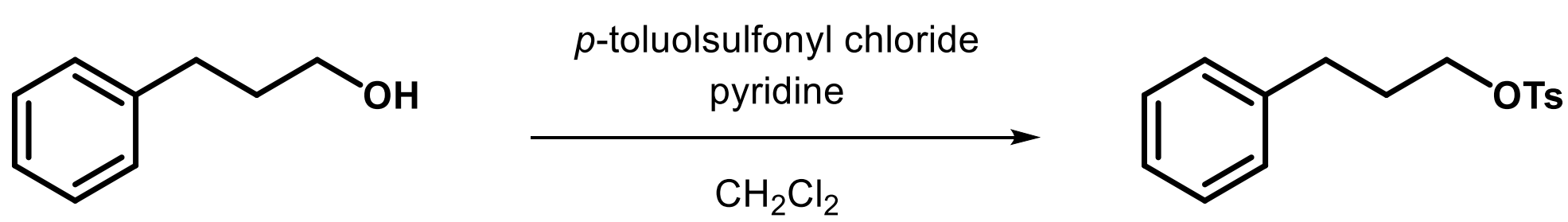

To a solution of 3-phenyl-1-propanol (1.0 eq, 1.00 g, 7.34 mmol) in CH_2Cl_2 (6.1 ml, 1.2 M), toluol-4-sulfonyl chloride (1.3 eq, 1.82 g, 9.55 mmol) and pyridine (1.3 eq, 755.0 mg, 9.55 mmol) were added. The reaction mixture is homogenous at first, but as the reaction proceeds a precipitate forms in the solution. After being stirred at room temperature for 20 h, the reaction mixture was diluted with 40 mL of ether and extracted sequentially two times with 10 mL of H_2O and two times with 10 mL of 10% HCl. The organic solution was stirred vigorously with 20 mL of concentrated NH_4OH for 1 h, followed by TLC until excess toluol-4-sulfonyl chloride was consumed. The layers were separated, the organic layer was extracted with 2×20 ml 2 N NaOH, then 2×20 ml H_2O, and 2×20 ml brine, and the solvent was evaporated to give 2.06 g of pale yellow oil (97%). The crude 3-phenylpropyl 4-methylbenzenesulfonate was used in the next step without further purification. ^1H NMR spectrum is in agreement with literature[4].

^1H NMR (300 MHz, $CDCl_3$) δ 7.85 – 7.78 (m, 2H), 7.39 – 7.33 (m, 2H), 7.30 – 7.15 (m, 3H), 7.12 – 7.05 (m, 2H), 4.05 (t, J = 6.2 Hz, 2H), 2.66 (dd, J = 8.5, 6.6 Hz, 2H), 2.47 (s, 3H), 2.05 – 1.91 (m, 2H). HRMS: theoretical $[C_{16}H_{18}O_3S_1 + Na]^+$ 313.086887, found (m/z): 313.086790.

General procedure for synthesis of selenides [3,5]

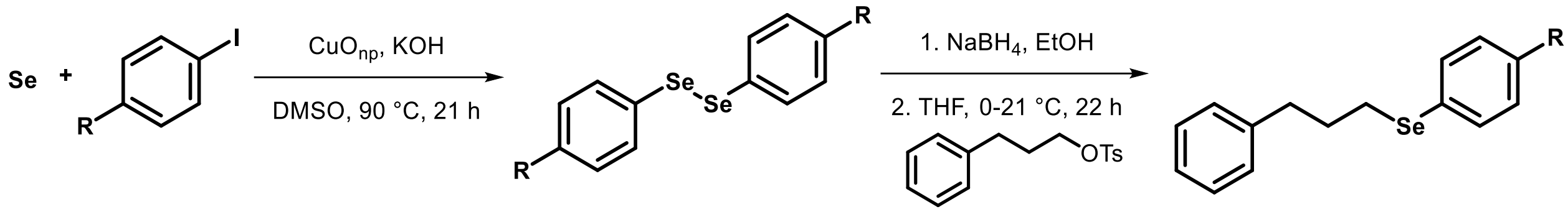

Se^0 metal (4.0 eq, 1.28 g, 16.2 mmol), CuO nanoparticles (0.1 eq, 32 mg, 0.41 mmol) and the corresponding aryl-iodide (1.0 eq, 4.05 mmol) were added to an oven-dried flask and purged with Ar. Dry DMSO (8.6 ml, 0.50 M) was added to the flask followed by KOH (2.0 eq, 455 mg, 8.10 mmol) under argon atmosphere. The reaction was stirred for 21 h at 90 °C and monitored by TLC.

After the reaction was complete, the reaction mixture was allowed to cool, and crude was mixed with celite followed by filtering on silica pad and washing with pentane.

After evaporation, the crude diphenyl diselenide (1.0 eq, 1.00 mmol) was dissolved in ethanol (5.6 ml, 0.18 M). $NaBH_4$ (2.0 eq, 76 mg, 2.00 mmol) was slowly added to the solution, which turned from yellow to colourless. The mixture was cooled to 0 °C and 3-phenylpropyl 4-methylbenzenesulfonate (3.0 eq, 871 mg, 3.00 mmol) in THF (4.2 ml, 0.72 M) was slowly added. The reaction mixture was let to warm to room temperature and stirred for 22 h before being poured into 30 ml 5% aqueous K_2CO_3 solution. Extraction with 3×20 ml ether, washing with 2×20 ml brine and drying on $MgSO_4$, followed by filtration and evaporation resulted in crude. Purification via column chromatography afforded selenides **1a-1f**. Isolated yields reported over two steps.

(4-methoxyphenyl)(3-phenylpropyl)selane (**1a**)

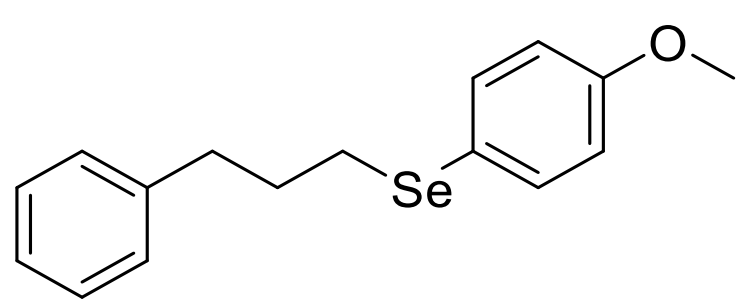

General procedure was followed starting from 1-iodo-4-methoxybenzene (1 eq, 947.8 mg, 4.05 mmol). Of the intermediate crude diphenyl diselenide (1.74 g dark brown solid-oil mixture) 340.2 mg was transferred to the next step. The product was purified via column chromatography (2% EtOAc in pentane) to yield **1a** as 213.8 mg light yellow oil (46 %). The product contains 20% inseparable impurity (bis(3-phenylpropyl)selane), which does not contribute to formation of **3** in following reactions.

^{1}H NMR (300 MHz, DMSO) δ 7.45 – 7.38 (m, 2H), 7.31 – 7.21 (m, 2H), 7.21 – 7.11 (m, 3H), 6.91 – 6.84 (m, 2H), 3.74 (s, 3H), 2.82 (t, J = 7.3 Hz, 2H), 2.66 (t, J = 7.6 Hz, 2H), 1.85 (p, J = 7.4 Hz, 2H). ^{13}C NMR (75 MHz, DMSO) δ 158.8, 141.2, 134.7, 129.5, 128.3, 125.8, 119.2, 115.0, 55.1, 34.9, 31.4, 27.4. HRMS: theoretical $[C_{16}H_{18}OSe]^+$ 306.051727, found (m/z): 306.052060.

(3-phenylpropyl)(p-tolyl)selane (**1b**)

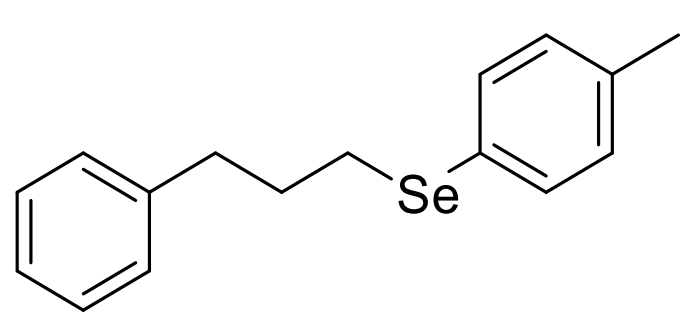

General procedure was followed starting from 1-iodo-4-methylbenzene (1 eq, 883.0 mg, 4.05 mmol). Of the intermediate crude diphenyl diselenide (679.2 mg orange solid-oil mixture) 372.2 mg was transferred to

the next step. The product was purified via column chromatography (1% EtOAc in pentane) to yield **1b** as 514.7 mg colourless oil (38 %).

^{1}H NMR (300 MHz, DMSO) *δ* 7.37 – 7.31 (m, 2H), 7.25 (dddd, *J* = 7.5, 6.5, 2.6, 1.3 Hz, 2H), 7.21 – 7.06 (m, 5H), 2.87 (dd, *J* = 7.8, 6.7 Hz, 2H), 2.66 (dd, *J* = 8.5, 6.6 Hz, 2H), 2.25 (s, 3H), 1.88 (p, *J* = 7.4 Hz, 2H). ^{13}C NMR (75 MHz, DMSO) *δ* 141.2, 136.1, 132.1, 129.9, 129.5, 128.3, 126.0, 125.8, 34.9, 31.4, 26.5, 20.6. HRMS: theoretical [$C_{16}H_{18}Se$]+ 290.056812, found (m/z): 290.056630.

phenyl(3-phenylpropyl)selane (**1c**)

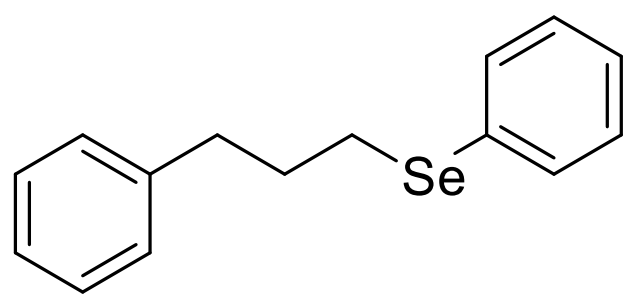

General procedure was followed only from second step starting from commercially available diphenyl diselenide (1 eq, 312.1 mg, 1 mmol). The product was purified via column chromatography (2% EtOAc in pentane) to yield **1c** as 282.2 mg pale yellow oil (51 %).

^{1}H NMR (300 MHz, DMSO) *δ* 7.44 (dd, *J* = 8.0, 1.6 Hz, 2H), 7.31 – 7.12 (m, 8H), 2.97 – 2.86 (m, 2H), 2.72 – 2.63 (m, 2H), 1.91 (p, *J* = 7.5 Hz, 2H). ^{13}C NMR (75 MHz, DMSO) *δ* 141.1, 131.4, 130.0, 129.5, 129.2, 128.3, 126.5, 125.8, 35.0, 31.4, 26.1. HRMS: theoretical [$C_{15}H_{16}Se$]+ 276.041162, found (m/z): 276.041350.

(4-chlorophenyl)(3-phenylpropyl)selane (**1d**)

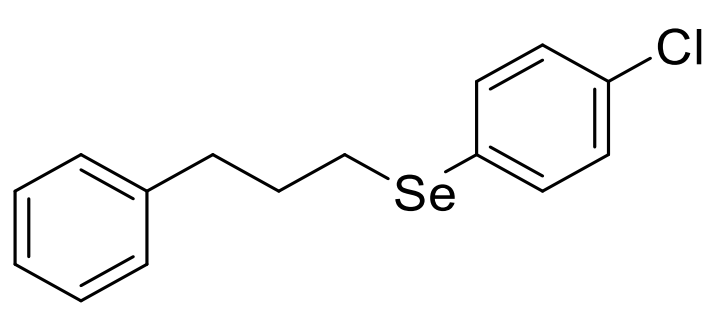

General procedure was followed starting from 1-chloro-4-iodobenzene (1 eq, 965.7 mg, 4.05 mmol). Of the intermediate crude diphenyl diselenide (727.1 mg orange solid) 381.0 mg was transferred to the next step. The product was purified via column chromatography (1% EtOAc in pentane) to yield **1d** as 532.0 mg yellow oil (41 %).

^{1}H NMR (300 MHz, DMSO) *δ* 7.46 – 7.39 (m, 2H), 7.35 – 7.21 (m, 4H), 7.19 – 7.10 (m, 3H), 2.92 (dd, *J* = 7.9, 6.9 Hz, 2H), 2.66 (dd, *J* = 8.5, 6.6 Hz, 2H), 1.90 (p, *J* = 7.5 Hz, 2H). ^{13}C NMR (75 MHz, DMSO) *δ* 141.9, 133.9, 132.2, 130.3, 129.9, 129.7, 129.1, 126.6, 35.8, 32.1, 27.2. HRMS: theoretical [$C_{15}H_{15}ClSe$]+ 310.002189, found (m/z): 310.002290.

(3-phenylpropyl)(4-(trifluoromethyl)phenyl)selane (**1e**)

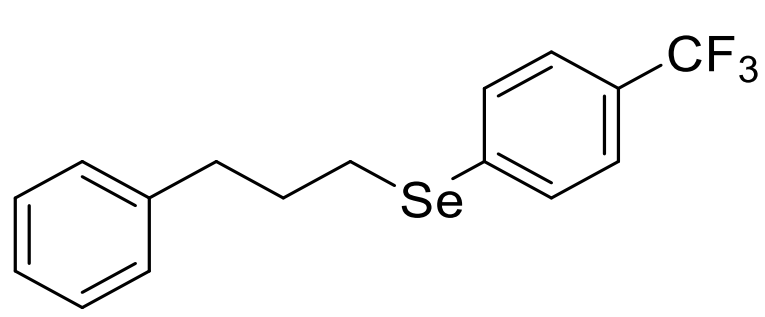

General procedure was followed starting from 1-iodo-4-(trifluoromethyl)benzene (1 eq, 1.10 g, 4.05 mmol). Of the intermediate crude diphenyl diselenide (419.1 mg yellow oil) 224.1 mg was transferred to the next step. The product was purified via column chromatography (1→5% EtOAc in pentane) to yield **1e** as 145.0 mg pale yellow solid (10 %).

^{1}H NMR (300 MHz, DMSO) δ 7.63 – 7.52 (m, 4H), 7.31 – 7.12 (m, 5H), 3.03 (dd, J = 7.9, 6.7 Hz, 2H), 2.69 (dd, J = 8.5, 6.6 Hz, 2H), 1.95 (p, J = 7.5 Hz, 2H). ^{13}C NMR (75 MHz, DMSO) δ 141.0, 137.0 (q, J = 1.6 Hz), 130.5, 129.5, 128.3, 126.5 (q, J = 32.2 Hz), 125.9, 125.6 (q, J = 3.7 Hz), 124.3 (q, J = 271.8 Hz), 35.0, 31.1, 25.5. ^{19}F NMR (282 MHz, DMSO) δ -61.1. HRMS: theoretical [$C_{16}H_{15}F_3Se$]+ 344.028547, found (m/z): 344.028480.

4-((3-phenylpropyl)selanyl)benzonitrile (**1f**)

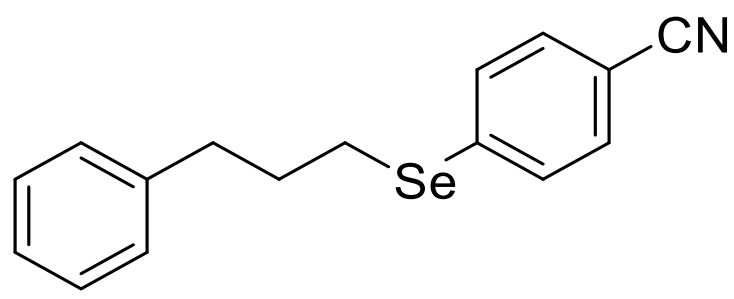

General procedure was followed starting from 4-iodobenzonitrile (1 eq, 927.5 mg, 4.05 mmol). Of the intermediate crude diphenyl diselenide (1.59 g yellow solid-oil mixture) 362.2 mg was transferred to the next step. The product was purified via column chromatography (**1% EtOAc in pentane**) to yield **1f** as 112.4 mg pale yellow solid (20 %).

^{1}H NMR (300 MHz, DMSO) δ 7.68 – 7.61 (m, 2H), 7.56 – 7.49 (m, 2H), 7.26 (ddd, J = 9.0, 6.3, 1.0 Hz, 2H), 7.19 – 7.12 (m, 3H), 3.02 (dd, J = 8.0, 6.8 Hz, 2H), 2.68 (dd, J = 8.5, 6.6 Hz, 2H), 1.94 (p, J = 7.6 Hz, 2H). ^{13}C NMR (75 MHz, DMSO) δ 140.9, 139.2, 132.4, 130.1, 129.5, 128.3, 125.9, 118.8, 108.1, 35.0, 31.1, 25.4. HRMS: theoretical [$C_{16}H_{15}NSe$]+ 301.036410, found (m/z): 301.036560.

Measurement of selenoxide elimination reaction rates

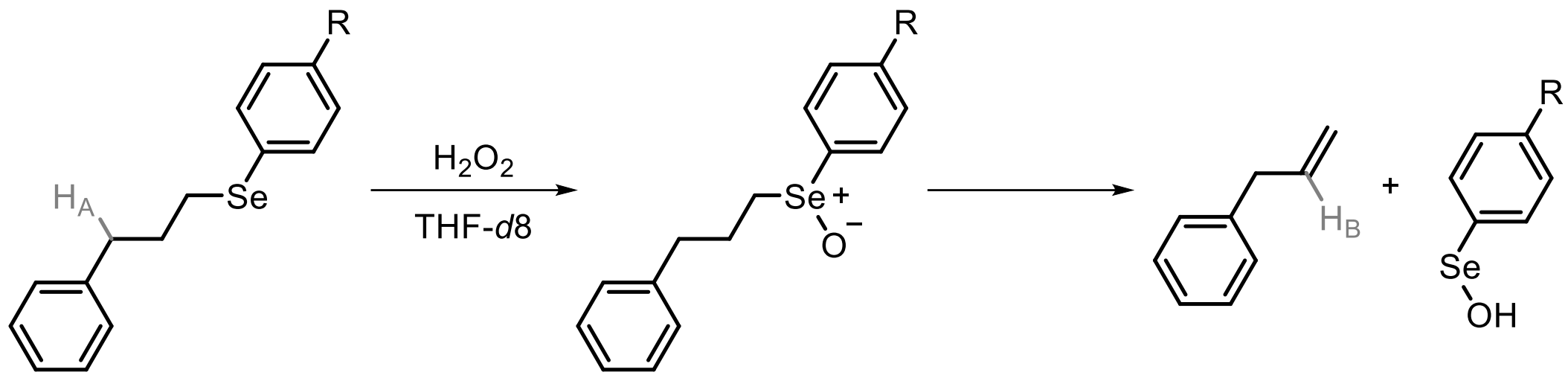

1a-f **2a-f** **3** **4a-f**

Stock solution with internal standard (IS) was prepared by dissolving mesitylene (0.083 mmol, 10 mg) in THF-d_8 (5 ml, 0.017 M). Selenide compounds (1 eq, 0.023 mmol) were weighed in separate NMR tubes and 0.5 ml of stock solution (0.33 eq IS, solution is 0.046 M for selenide) was added to each. The sealed tubes were shaken before every measurement to ensure homogeneity.

A 0 min reference spectrum was measured before oxidation, integral of IS (6.76 ppm, 3H) corresponding to int(2 H_A) = 200 was determined. For kinetics, the reaction time associated with each spectrum measurement is the timepoint when the fid file is created referenced to the moment of H_2O_2 addition. After addition of H_2O_2 (35%, 10 eq, 20.0 µl, 0.21 mmol), ^{1}H NMR measurement was performed once every hour for approximately 6 h (**Figure S1**). Integration of well-separated H_B vinilic proton multiplet at 5.96 ppm of allylbenzene[6] **3** was used to calculate concentration of product formed for all substrates. The reactions were reproduced three times for each substrate, reaction rates measured for each run separately (**Table S1**). Reaction rates were determined by linear fitting on selenoxide concentration logarithm plotted against timepoints (**Figure S2**). For each substrate the average of three reaction rates was used as k_{obs} in responsiveness analysis.

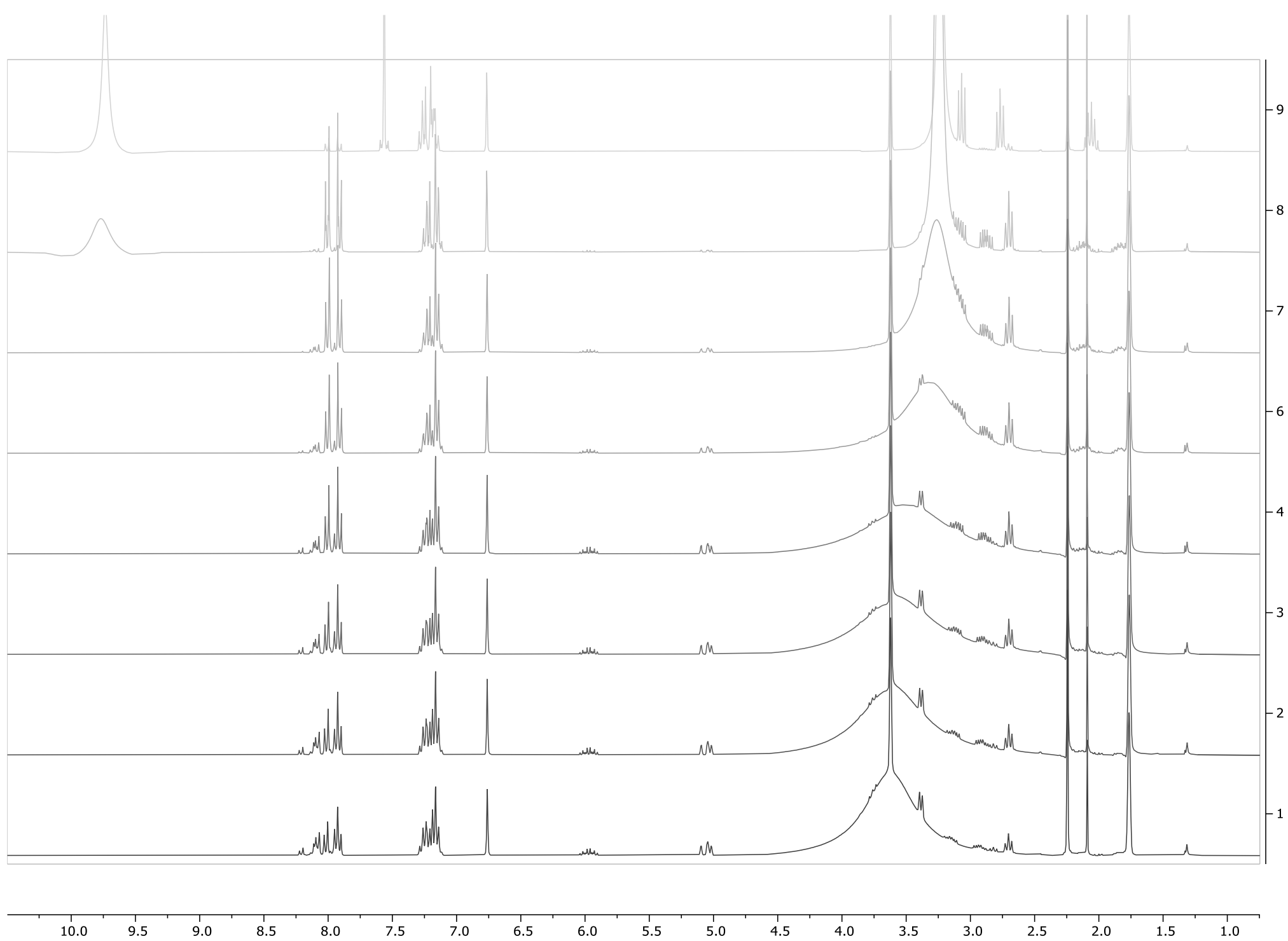

Figure S1. Representative stack of ^{1}H NMR (300 MHz, THF-d_8) spectra following selenoxide elimination after oxidation starting from **1f** as the starting material (Table S1 entry 6, 2nd run) . Timepoints taken at 8, 48, 103, 141, 217, 275, 317 and 377 minutes

(from top to bottom). **1f** is fully consumed at 48 min (peak at 7.56 ppm disappeared), selenoxide elimination rate measurement from this timepoint via multiplet at 5.96 ppm.

Table S1. Measurement times, integration and calculated concentration of **3** used for rate determination.

#	R	1st run			2nd run			3rd run		
		***t* (min)**	**Int[a]**	**c_3 (M)**	***t* (min)**	**Int[a]**	**c_3 (M)**	***t* (min)**	**Int[a]**	**c_3 (M)**
1	**OMe** (2a)	124	4.79	0.00281	102	3.21	0.00148	56	1.87	0.00086
		185	6.94	0.00407	141	4.78	0.0022	104	3.67	0.00169
		230	9.28	0.00545	180	6.32	0.00291	153	5.7	0.00262
		281	11.23	0.00659	218	7.89	0.00363	203	7.55	0.00347
		378	14.13	0.00829	277	9.7	0.00446	253	9.09	0.00418
					318	11.22	0.00516	307	10.98	0.00505
					379	13.41	0.00617	369	13.32	0.00613
2	**Me** (2b)	146	4.59	0.00269	148	4.46	0.00205	155	2.87	0.00132
		206	6.95	0.00408	213	7.32	0.00337	205	5.31	0.00244
		250	9.21	0.00541	276	9.95	0.00458	258	7.88	0.00362
		299	11.5	0.00675	406	16.42	0.00755	309	10.12	0.00466
		413	16.29	0.00956	560	22.33	0.01027	381	13.16	0.00605
3	**H** (2c)	140	2.4	0.00141	145	3.74	0.00172	141	2.81	0.00129
		200	5.6	0.00329	210	6.97	0.00321	179	4.86	0.00224
		244	8.89	0.00522	273	10.78	0.00496	218	6.99	0.00322
		295	11.65	0.00684	403	17.35	0.00798	277	10.34	0.00476
		407	17.94	0.01053	556	25.05	0.01152	319	12.56	0.00578
								380	15.37	0.00707
4	**Cl** (2d)	133	5.58	0.00328	148	6.56	0.00302	107	2.15	0.00099
		193	9.83	0.00577	214	11.35	0.00522	156	5.82	0.00268
		237	13.65	0.00801	277	16.13	0.00742	206	9.16	0.00421
		288	17.62	0.01034	406	23.83	0.01096	259	12.52	0.00576
		399	25	0.01468	560	31.67	0.01457	310	15.43	0.00710
								383	19.64	0.00903
5	**CF_3** (2e)	178	11.64	0.00683	215	9.93	0.00457	107	4.63	0.00213
		222	18.52	0.01087	261	15.61	0.00718	157	10.57	0.00486
		274	24.26	0.01424	304	20.78	0.00956	206	16.02	0.00737
		357	33.72	0.01979	346	25.26	0.01162	260	21.28	0.00979
								311	25.85	0.01189
								384	32.07	0.01475
6	**CN** (2f)	102	4.58	0.00211	48	5.36	0.00247	58	2.93	0.00135
		163	14.92	0.00686	103	13.35	0.00614	107	10.56	0.00486
		217	22.61	0.0104	141	18.6	0.00856	158	17.75	0.00817
		263	29.2	0.01343	217	28.23	0.01299	206	23.83	0.01096
		306	34.34	0.0158	275	34.7	0.01596	260	29.95	0.01378
		348	38.93	0.01791	317	38.65	0.01778	320	35.53	0.01634
					377	43.66	0.02008	385	41.96	0.0193

a) integral of characteristic multiplet at 5.96 ppm (1 ^{1}H), internal standard used as a reference (Int 100 = 100% of 1 ^{1}H)

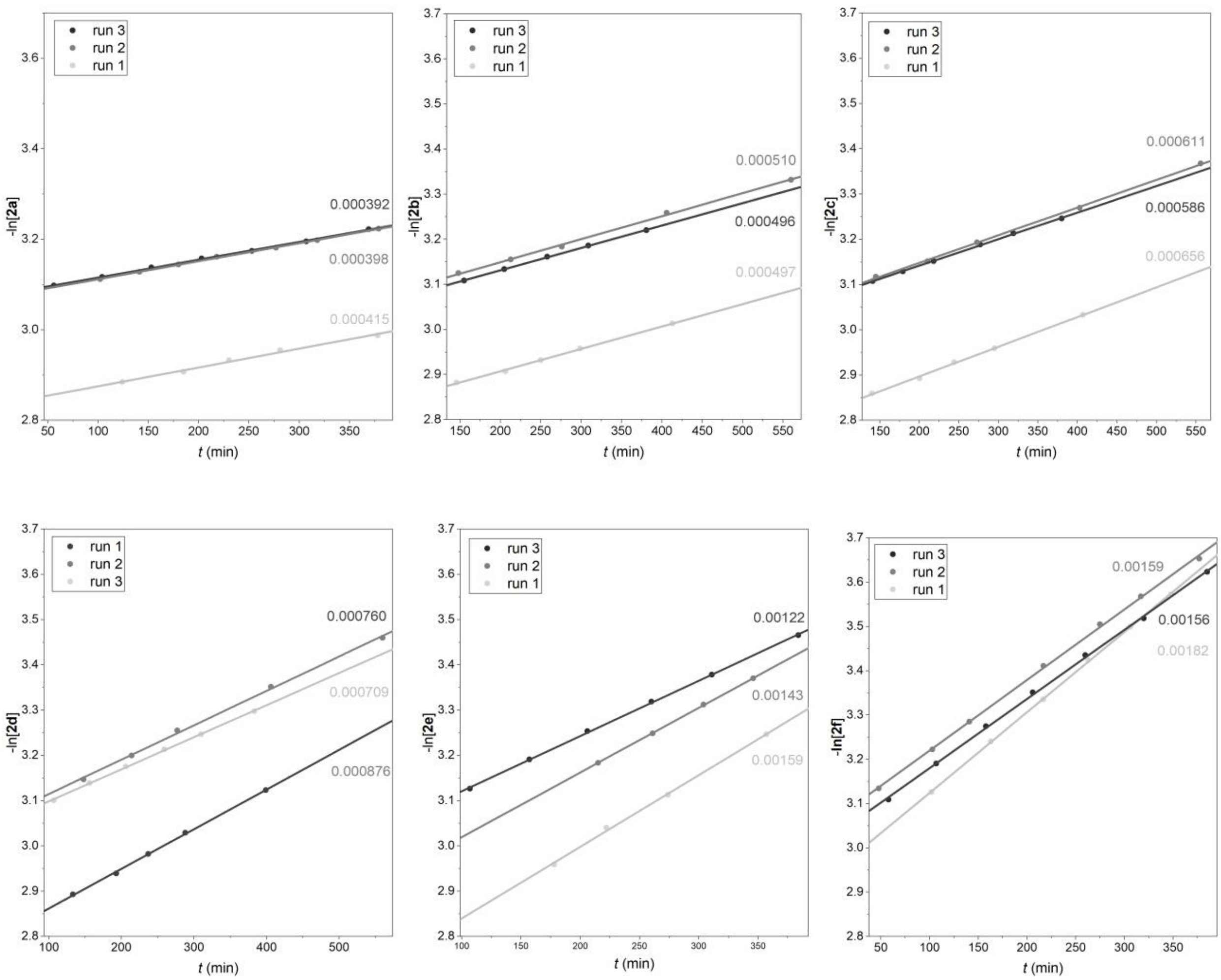

Figure S2. Kinetic graph for all substrates for each run - consumption of selenoxide calculated from concentration of product allylbenzene **3**. Rate (min^{-1}) determined by slope of linear fitting and displayed next to each line.

Table S2. Summary of rate constants.

**R**	**OMe** (2a)	**Me** (2b)	**H** (2c)	**Cl** (2d)	**CF$_3$** (2e)	**CN** (2f)
1st run	0.000415	0.000497	0.000656	0.000876	0.00159	0.00182
2nd run	0.000398	0.000510	0.000611	0.000760	0.00143	0.00159
3rd run	0.000392	0.000496	0.000586	0.000709	0.00122	0.00156
**average**[a]	0.000402 min^{-1}	0.000501 min^{-1}	0.000618 min^{-1}	0.000782 min^{-1}	0.00141 min^{-1}	0.00166 min^{-1}
	**0.0000067 s^{-1}**	**0.0000084 s^{-1}**	**0.0000103 s^{-1}**	**0.0000130 s^{-1}**	**0.0000235 s^{-1}**	**0.0000277 s^{-1}**

a) average in s^{-1} units is taken as k_{obs} for experimental Brønsted plot of the responsiveness analysis

Responsiveness analysis

Responsiveness analysis is the term used in this work for our new tool that enables diagnosis of tunneling in organic reactions at any temperature. The diagnostic is based on intrinsic reactivity analysis, which in practice is quantified via the Brønsted plot: reactivity plotted against the driving force of a family of reactions. In the linear approximation, intrinsic reactivity parameters can be measured through linear fitting of the datapoints on a Brønsted plot: the slope α (Brønsted slope/responsiveness) and intercept $\Delta G^{‡}_{0}$ (intrinsic barrier). Each datapoint belongs to a different condition, in the present case different substrates (**2a-f**). When these conditions belong to the same family of reactions, the datapoints give a good correlation and intrinsic reactivity parameters can be determined by fitting. In responsiveness analysis we focus on the responsiveness or Brønsted slope (α) of a reaction family.

According to our conceptual derivation, in case of tunneling, the experimentally observed Brønsted slope α_{obs} will be significantly higher than a predicted thermal Brønsted slope α_{TST}.

Construction of the Experimental Brønsted plot:

x axis: **driving force** ($-\Delta G$)

calculated from computational energies of starting material and product, here: $-\Delta G = G_{SM} - G_{tP}$
where G_{SM} is the energy of starting material ground state, G_{tP} is the energy of tunneling product (see: Computational methods section)

y axis: **rate** ($RT\ln(k)$)

calculated from the experimentally measured rate constants k_{obs}, T = 294 K and R = 0.001987 kcal/K

Required data for experimental Brønsted plot:

- energy difference of starting states and product states (here: computational)
- rate and temperature of reactions (here: experimental)

Construction of the predicted thermal Brønsted plot:

x axis: **driving force** ($-\Delta G$)

same as for the x axis of experimental Brønsted plot

y axis: inverse **thermal barrier height** ($-\Delta G^{‡}$)

calculated from computational energies of starting material and transition state, here: $-\Delta G = G_{SM} - G_{TS}$
where G_{SM} is the energy of starting material ground state, G_{TS} is the energy of transition state

Required data for thermal Brønsted plot:

- energy difference of starting states and product states (here: computational)
- energy difference of starting states and transition states (here: computational)

Formal derivation of amplified observed responsiveness as a result of tunneling contribution:

Thermal pathway of a family of reactions follow the Leffler equation:

$$\Delta G^{\ddagger} = \Delta G_0^{\ddagger} + \alpha \Delta G$$

where $\Delta G^{\ddagger}$: barrier height, $\Delta G^{\ddagger}_0$: intrinsic barrier, α: Brønsted slope, $-\Delta G$: driving force

Rate of thermal reactions (k_{TST}) can be expressed by the Eyring equation:

$$k_{\mathrm{TST}} = \frac{bT}{h} \times e^{\frac{-\Delta G^{\ddagger}}{RT}} \qquad \text{rearranged:} \qquad -\Delta G^{\ddagger} = c \times RT\ln(k_{\mathrm{TST}})$$

where b: Boltzmann constant, h: Planck constant, R: gas constant, T: temperature, c: $\ln(R \times h/b)$

Tunneling contributes as an additional product generating pathway. The rate of tunneling k_{QMT} is commonly expressed as:

$$k_{QMT} = k_{\mathrm{TST}}\kappa = \nu \times \frac{1}{1 + e^{\frac{-2\sqrt{2}}{h}\int \sqrt{(V_0 - E_0)m}\,dx}}$$

where κ: tunneling correction factor, ν: frequency of productive mode, m: mass of tunneling particle

The $(V_0 - E_0)$ term corresponds to barrier height, while dx term corresponds to barrier width. Because the exponential function is strictly monotonically increasing while the reciprocal function is strictly monotonically decreasing, the higher these terms are the lower the tunneling rate is.

On the Brønsted plot two systems can be depicted as A and B (belonging to the same family of reactions), where B has higher driving force, consequentially lower barrier height and smaller barrier width than A.

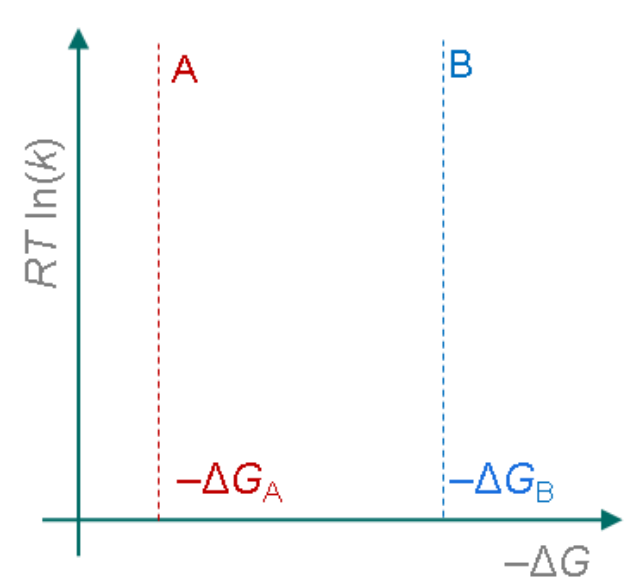

The thermal Brønsted slope α_{TST} for this plot can be calculated as:

$$\alpha_{\mathrm{TST}} = \frac{\partial \Delta G^{\ddagger}}{\partial \Delta G} = \frac{\partial(-\Delta G^{\ddagger})}{\partial(-\Delta G)} = \frac{\Delta G_{\mathrm{A}}^{\ddagger} - \Delta G_{\mathrm{B}}^{\ddagger}}{\Delta G_{\mathrm{A}} - \Delta G_{\mathrm{B}}} = \frac{RT\ln(k_{\mathrm{B}}) - RT\,ln(k_{\mathrm{A}})}{\Delta G_{\mathrm{A}} - \Delta G_{\mathrm{B}}}$$

Here $\Delta G_{\mathrm{A}} > \Delta G_{\mathrm{B}}$ and $\Delta G^{\ddagger}_{\mathrm{A}} > \Delta G^{\ddagger}_{\mathrm{B}}$, so the thermal slope will be positive.

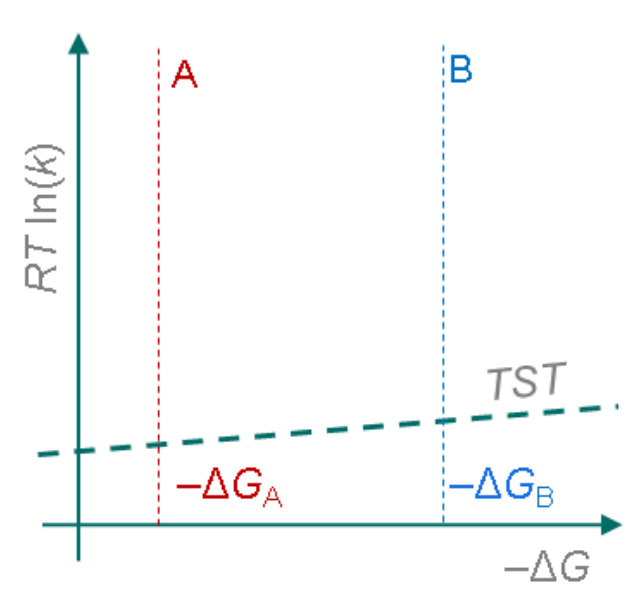

The coexisting tunneling Brønsted slope can be calculated as:

$$\alpha_{\mathrm{QMT}} = \frac{RT\ln(k_{\mathrm{B}}\kappa_{\mathrm{B}}) - RT\,ln(k_{\mathrm{A}}\kappa_{\mathrm{A}})}{\Delta G_{\mathrm{A}} - \Delta G_{\mathrm{B}}}$$

The addition of two linear relationships could only decrease the combined slope if one of them has a negative sign. As B has lower barrier height and smaller barrier width than A: $k_{\mathrm{QMT,\,B}}$ will be larger than $k_{\mathrm{QMT,\,A}}$. While the driving force axis is the same for both tunneling and thermal Brønsted slopes, and the rate of B is higher in both cases, both slopes will be positive.

Thus the tunneling contribution cause an amplification to the observed Brønsted slope.

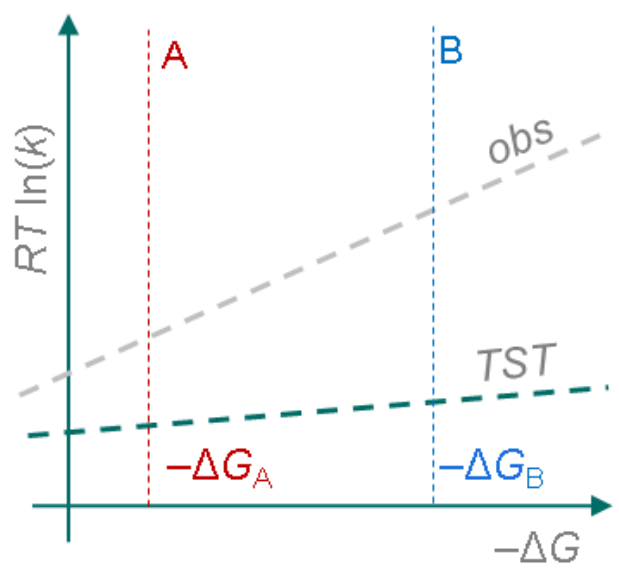

Computational methods

Transition state calculation was aided by Compound, a scripting language embedded in ORCA[2]. The original script (form ORCA 6.1 manual[7]) was modified by the author. StartGeom.xyz was created separately for each substrate, containing an initial guess for TS geometry.

IRC calculations were carried out to inspect the shape of the potential and determine the tunneling product and starting state connecting with the transition state. Table 3 summarizes the meaning of keywords used and their setting applied here. Hessian used from previous TS calculation denoted as "[filename.hess]" in the input.

TS (transition state) calculation full input:

```
%maxcore 10000
%pal nprocs 8
end
%Compound
# Step 1. Run a single point calculation (we need it for the first property file)
#
# Step 1. Loop and perform calculations with (optimization and frequencies)
#
# Step 2. Check the frequencies. If there are negative ones use the hessian
#         of the appropriate normal mode to adjust the geometry
#
# ------ Variables to adjust (e.g. using 'with') -------------------
Variable method          = "B3LYP D3BJ def2-TZVP verytightscf"; #"functional and basis set";
Variable MaxNTries       = 5;   # Number of maximum tries
Variable CutOff          = -10.0; # CutOff for a negative frequency
Variable scaling         = 0.6;   # Scaling factor for normal mode
Variable NNegativeTarget = 1;     # Number of negative frequencies we allow
Variable myFilename      = "StartGeom.xyz";
Variable charge          = 0;
Variable multiplicity    = 1;
# ---------------------------------------------------------------
# ------           Rest of variables           -------------------
Geometry myGeom;
Variable freqs, modes;
Variable res = -1;
Variable NNegative =   0;
Variable OptDone;
```

```
# -----------------------------------------------------------
# Perform a single point calculation. We need it for
# the initial geometry from the property file
# -----------------------------------------------------------
New_Step
  !&{method}
  *xyzfile &{charge} &{multiplicity} &{myFilename}
Step_End
myGeom.Read();
myGeom.WriteXYZFile(filename=myFilename);

# -----------------------------------------------------------
# Start a for loop over number of tries
# ----------------------------------------------------------
For itry From 1 To maxNTries Do
  # ---------------------------------------------
  # Perform a geometry optimization/Frequency calculation
  # ---------------------------------------------
  New_Step
    ! &{method} freq OptTS
    %geom
     Calc_Hess true
     end
    %CPCM SMD true
     SMDSOLVENT "TETRAHYDROFURAN" END
    *xyzfile &{charge} &{multiplicity} &{myFilename}
  Step_End
  res = freqs.readProperty(propertyName = "THERMO_FREQS");
  res = modes.readProperty(propertyName = "HESSIAN_MODES");
  myGeom.Read();

  # ----------------------------------------------
  #  check for sufficiently negative frequencies
  # ----------------------------------------------
  NNegative = 0;
  For ifreq From 0 to freqs.GetSize()-1 Do
    if ( freqs[ifreq] < CutOff )  then
       myGeom.FollowNormalMode(vibrationSN=ifreq, scalingFactor=scaling);
```

```
      NNegative = NNegative + 1;
    endif
  endfor
  myGeom.WriteXYZFile(filename=myFilename);
  If ( NNegative <= NNegativeTarget ) then
    goto OptDone;
  endif
endfor

# ------------------------------------------------------------------
# Either found correct geometry or reached maximum number of tries.
# ------------------------------------------------------------------
OptDone :
if (NNegative > NNegativeTarget) then
  print("ERROR The program did not find a structure with the desired\n number of imaginary frequencies.\n There are %9.3lf negative frequencies after %3d steps", NNegative,itry);
else
  print("\nSUCCESS optimized structure with (%d) negative\n frequencies found after %3d steps", NNegative, itry);
endif

End
```

IRC (intrinsic reaction coordinate) calculation full input:

```
! irc B3LYP D3BJ def2-TZVP def2/J verytightscf
%pal nprocs 12 end
%maxcore 8000
%irc
printlevel 1
method lqa
maxiter 241
tolrmsg 1.e-5
tolmaxg 8.e-5
follow_coordtype massweighted
inithess read
hess_filename "[filename.hess]"
lqahess lqacalc_bofillupdate
init_displ length
scale_init_displ 0.04
adapt_scale_displ false
scale_displ_sd 0.11
enforcestrictconvergence true
reportprofile true
bscorrector true
bsrichthresh 1E-6
initbscorrector false
end
%CPCM SMD true
    smdsolvent "tetrahydrofuran" end
```

Table S3. Keywords for IRC calculation with ORCA

Feature	keyword	setting
Use the local quadratic approximation (LQA) algorithm	method	lqa
The followed coordinates are mass-weighted (not cartesian)	follow_coordtype	massweighted
Use previously computed Hessian (defined by Hess_Filename)	inithess<br>hess_filename	read<br>"[filename.hess]"
LQA Hessian updated with the Bofill method for convergence	lqahess	lqacalc_bofillupdate
Adjust convergence thresholds: RMS gradient (a.u.), maximum element of gradient (a.u.)	tolrmsg<br>tolmaxg	1.e-5<br>8.e-5
Enforce strict convergence	enforcestrictconvergence	true
Initial displacement by step size (not energy difference)	init_displ	length
Step size for initial displacement from TS (a.u.)	scale_init_displ	0.04
Scaling factor for scaling the first steepest descent (SD) step	scale_displ_sd	0.11
Do not modify the scaling factor	adapt_scale_displ	false
Use Bulirsch-Stoer extrapolation method, but not on initial displacement	bscorrector<br>initbscorrector	true<br>false
Set Bulirsch-Stoer correction threshold	bsrichthresh	1E-6
Report energy profile	reportprofile	true
Control the amount of output.	printlevel	1

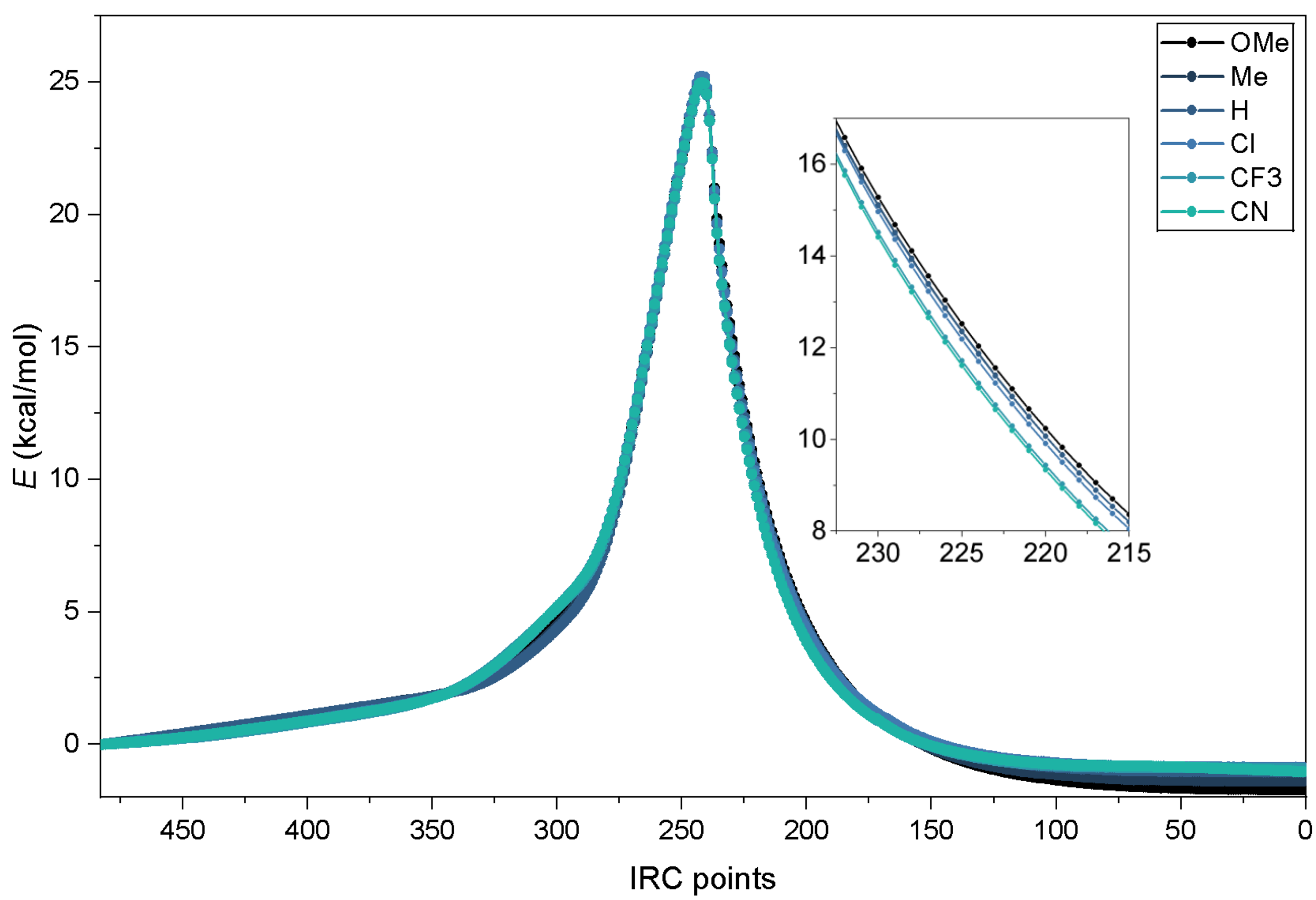

Figure S3. Overlay of IRC plots with expansion of the tunneling product region. R substituents indicated in the top right corner.

Determination of tP (tunneling product) and IRC point-geometry correlation check

IRC calculation results in the minimal energy pathway connecting the TS (transition state, IRC point 242) with SM (starting material ground state, IRC point 483) and P (product ground state, IRC point 1), representing the geometry and energy changes of the thermal channel. An overlay of IRC plots calculated for each substrate can be seen on **Figure S3**, referenced to SM (left end of the graph), each substrate depicted with a different colour. The reaction proceeds from left to right. The shift in barrier width is visible on the inserted expansion, providing a real-life counterpart to **Figure 2** of the main text.

To ensure comparability IRCs of different substrates with each other, **Figure S4** was created to check the geometrical agreement for a range of IRC point around the TS. IRC points correspond to mass-weighted distance of atomic movements, in this case 0.110 $a_0 \times u^{0.5}$ (a_0: bohr–distance; u: dalton–mass) between each point.

On **Figure S4** the C−H bond breaking (circles) and the O−H bond formation (squares) is monitored through the respective distances (atoms involved highlighted with red). It is visible, that the geometrical movement of substrates is aligned throughout the set (different colours/sizes), only starting to diverge further from the TS. To ensure comparability between different substrates, the energy of the IRC point 226 (marked with a black circle) was taken as the tP (tunneling product), where the O−H bond is fully formed, but the geometries have not started diverging for different substrates. These energies were used for the construction of the final Brønsted plot, ensuring that the driving force differences are the result of purely energetic rather than geometrical mismatch corresponding to the tunneling process.

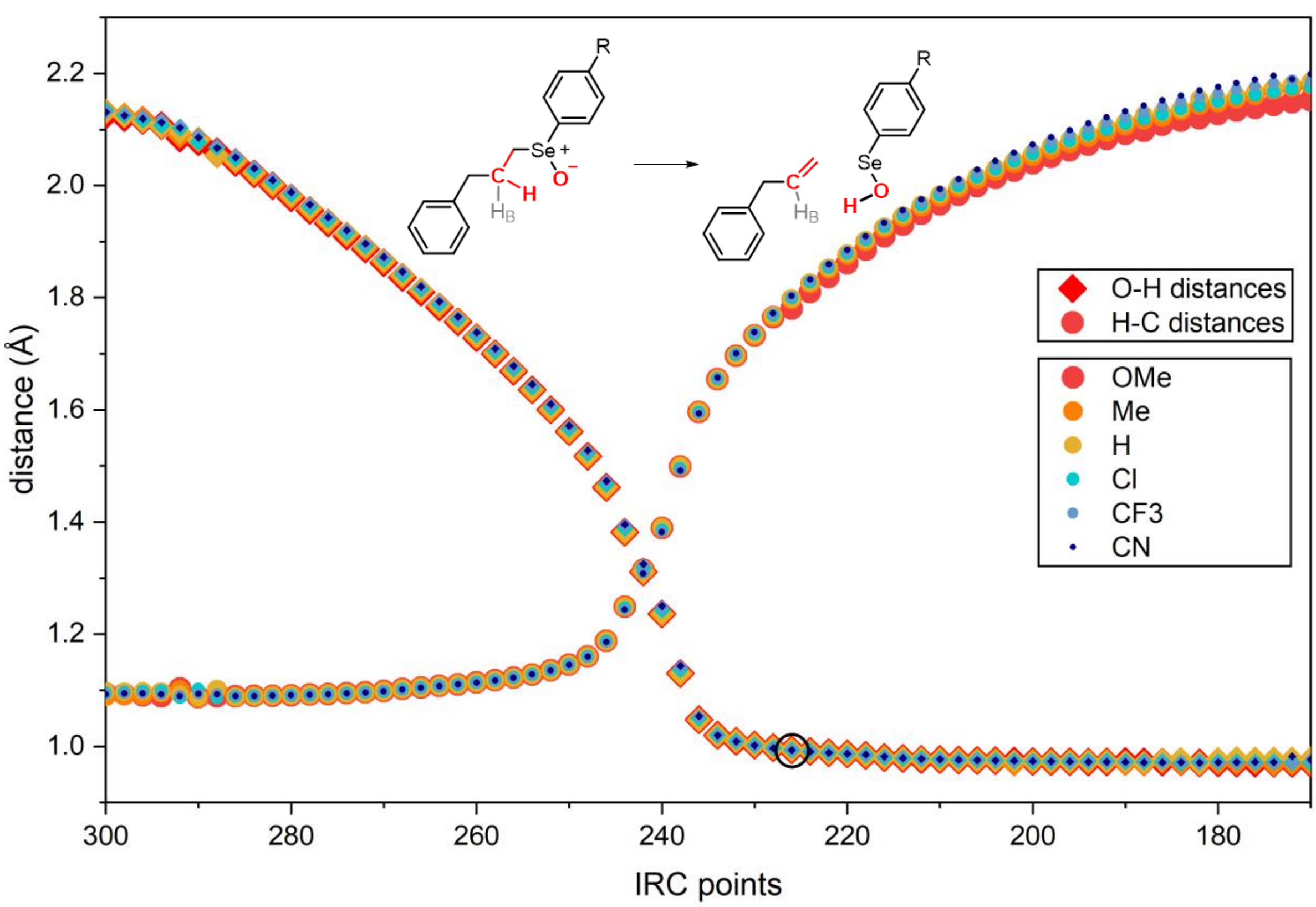

Figure S4. Formation of O−H bond (squares) and breaking of C−H bond (circles) through IRC for all substrates overlayed. Black circle indicates IRC point taken as tunneling product (IRC point 226). Direction of reaction is from left to right.

Computational results for constructing the Brønsted plot

Energies of transition state (**E_{TS}**), starting material ground state (**E_{SM}**) and tunneling product (**E_{tP}**) were obtained with different levels of theory (Table S4.). Geometries were derived from IRC calculations at B3LYP-D3(BJ)/def2-TZVP level of theory. **E_{SM}-E_{tP}** is taken as driving force ($-\Delta G$) for Brønsted plots. **E_{SM}** − **E_{TS}** is taken as inverse thermal barrier height ($-\Delta G^{\ddagger}$) for Brønsted plots. Units were converted to kcal/mol for responsiveness analysis ([Eh]×627.51=[kcal/mol]).

Energies obtained directly from IRC calculations were used for Fig. 3B in main text (Method: A*), results from single point calculation with other levels of theory displayed on Figure S4 to show that our tunneling analysis does not depend significantly on the applied computational method.

Table S4. Computational data used for Brønsted plots.

#	R	Method[a]	**OMe** (2a)	**Me** (2b)	**H** (2c)	**Cl** (2d)	**CF_3** (2e)	**CN** (2f)
1	**E_{SM}** (Eh)	A*	-3172.30966	-3097.09817	-3057.78920	-3517.35872	-3394.86832	-3150.03035
2		A	-3172.32069	-3097.10853	-3057.79873	-3517.36861	-3394.87906	-3150.03973
3		B	-3171.66039	-3096.49458	-3057.20785	-3516.67953	-3394.09202	-3149.38486
4		C	-3172.19325	-3096.98180	-3057.67951	-3517.28012	-3394.76329	-3149.9055
5		D	-3169.76116	-3094.6437	-3055.39794	-3514.53134	-3392.08364	-3147.49889
6	**E_{TS}** (Eh)	A*	-3172.26948	-3097.05830	-3057.74935	-3517.31853	-3394.82861	-3149.99055
7		A	-3172.28002	-3097.0681	-3057.75834	-3517.32793	-3394.83879	-3149.99943
8		B	-3171.61657	-3096.45110	-3057.16434	-3516.63585	-3394.04871	-3149.34166
9		C	-3172.14280	-3096.93172	-3057.62990	-3517.22968	-3394.71305	-3149.85519
10		D	-3169.71087	-3094.5937	-3055.34797	-3514.48085	-3392.03340	-3147.44850
11	**E_{SM}-E_{TS}** (kcal/mol)	A*	**-25.21**	**-25.02**	**-25.00**	**-25.22**	**-24.92**	**-24.97**
12		A	**-25.52**	**-25.38**	**-25.35**	**-25.52**	**-25.27**	**-25.28**
13		B	**-27.49**	**-27.28**	**-27.30**	**-27.41**	**-27.18**	**-27.11**
14		C	**-31.66**	**-31.43**	**-31.13**	**-31.65**	**-31.53**	**-31.57**
15		D	**-31.56**	**-31.37**	**-31.36**	**-31.68**	**-31.53**	**-31.62**
16	**E_{tP}** (Eh)	A*	-3172.28888	-3097.07765	-3057.76871	-3517.33848	-3394.84883	-3150.01102
17		A	-3172.30007	-3097.08799	-3057.77843	-3517.34855	-3394.85965	-3150.02055
18		B	-3171.63519	-3096.46976	-3057.18301	-3516.65511	-3394.06823	-3149.36148
19		C	-3172.16614	-3096.95510	-3057.65331	-3517.25375	-3394.73736	-3149.87980
20		D	-3169.73428	-3094.61715	-3055.37147	-3514.50520	-3392.05795	-3147.47322
21	**E_{SM}-E_{tP}** (kcal/mol)	A*	**-13.04**	**-12.87**	**-12.86**	**-12.70**	**-12.23**	**-12.13**
22		A	**-12.94**	**-12.88**	**-12.74**	**-12.59**	**-12.18**	**-12.03**
23		B	**-15.81**	**-15.57**	**-15.59**	**-15.32**	**-14.93**	**-14.68**
24		C	**-17.02**	**-16.76**	**-16.44**	**-16.55**	**-16.27**	**-16.12**
25		D	**-16.87**	**-16.66**	**-16.61**	**-16.40**	**-16.12**	**-16.11**

a) Methods: all with SMD(tetrahydrofuran) solvation with different levels of theory. A: B3LYP-D3(BJ)/def2-TZVPPD DefGrid3; A*: B3LYP-D3(BJ)/def2-TZVP, energies directly form IRC calculation; B: PBE0-D4/def2-TZVPPD DefGrid3; C: M06-D4/def2-TZVPPD DefGrid3; D: DLPNO-CCSD(T)/def2-TZVPPD def2-TZVPPD/C def2/J RIJCOSX TightPNO.

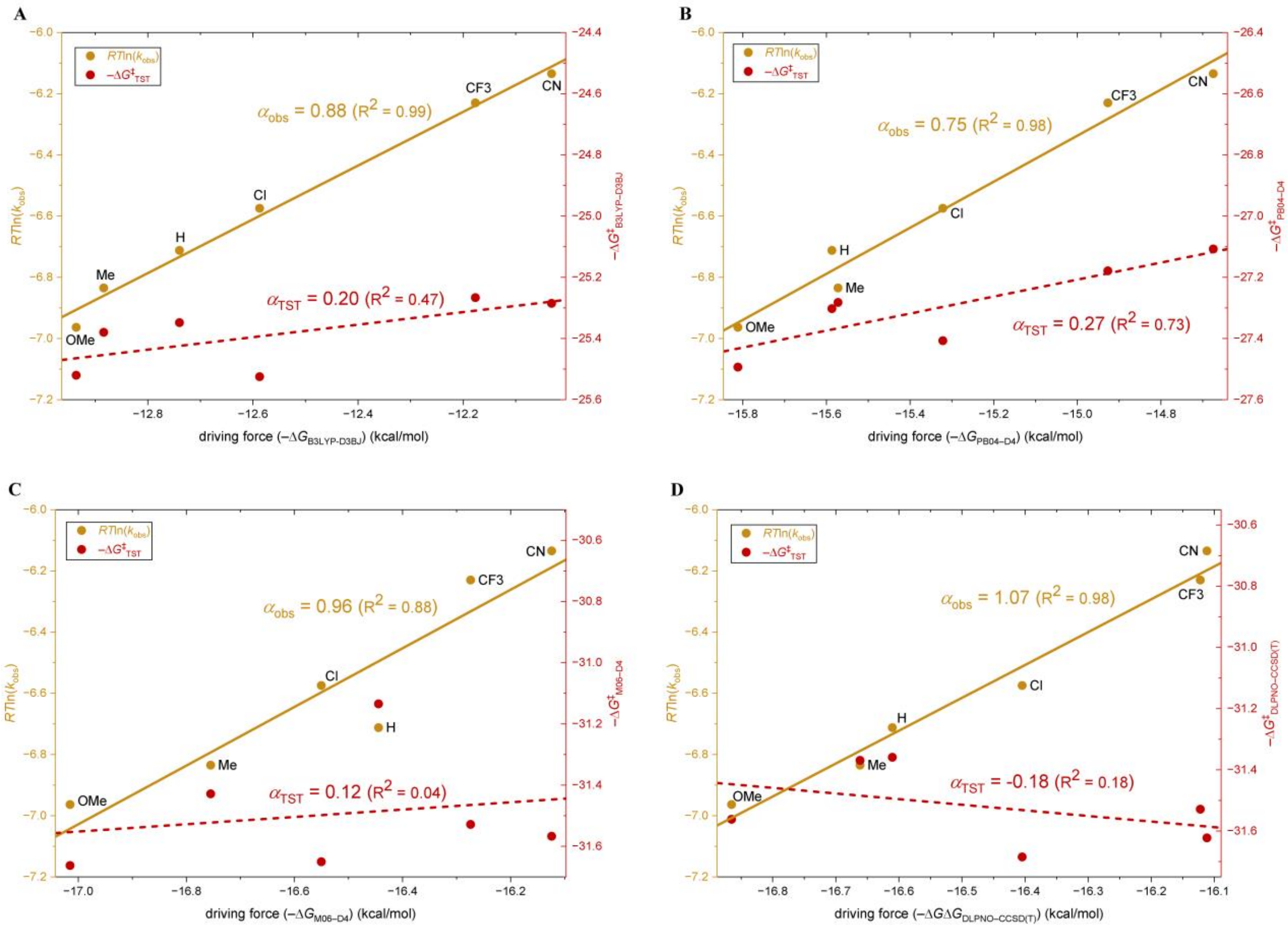

Figure S5. Brønsted plots with different levels of theory. A: B3LYP-D3(BJ)/def2-TZVPPD DefGrid3; B: PBE0-D4/def2-TZVPPD DefGrid3; C: M06-D4/def2-TZVPPD DefGrid3; D: DLPNO-CCSD(T)/def2-TZVPPD def2-TZVPPD/C def2/J RIJCOSX TightPNO.

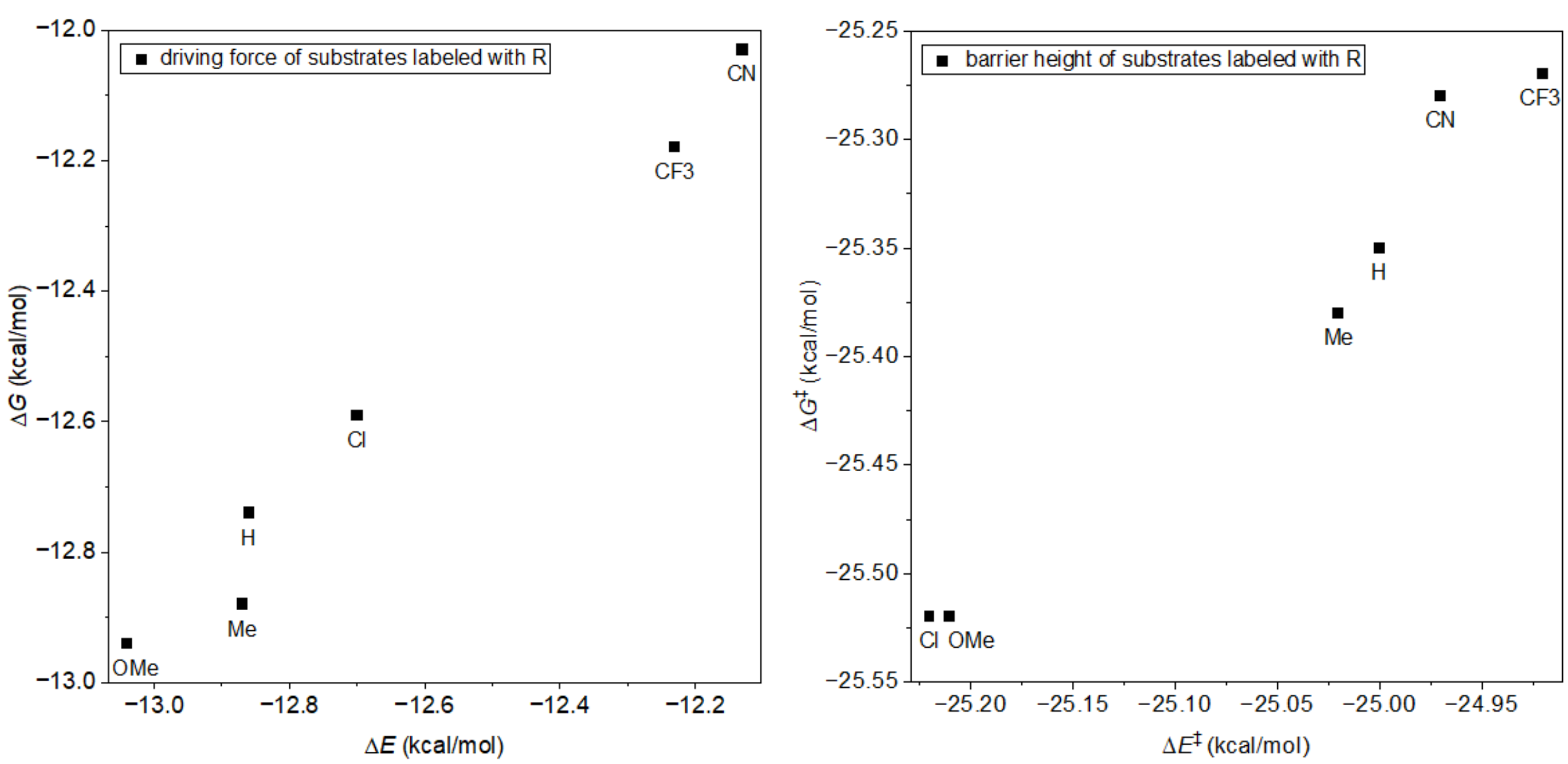

Figure S6. Correlation check between potential and free energy surface measures at B3LYP-D3(BJ)/def2-TZVP level of theory.

Conceptual summary of responsiveness analysis

The responsiveness analysis is based on a differential comparison of reactivity changes within a homologous reaction family under a controlled thermodynamic perturbation. While the absolute reaction rate can be influenced by many factors, including barrier height, barrier topology, vibrational properties, anharmonicity, and dynamical effects, the diagnostic examines whether these factors exhibit a coherent response to the same thermodynamic driving force. Contributions that are preserved throughout the reaction family become part of the intrinsic reactivity baseline, whereas non-systematic variations are expected to manifest as deviations from a coherent Brønsted relationship. Therefore, the central mechanistic information arises not from the absolute value of the rate, but from the rate–driving force responsiveness that survives the differential comparison.

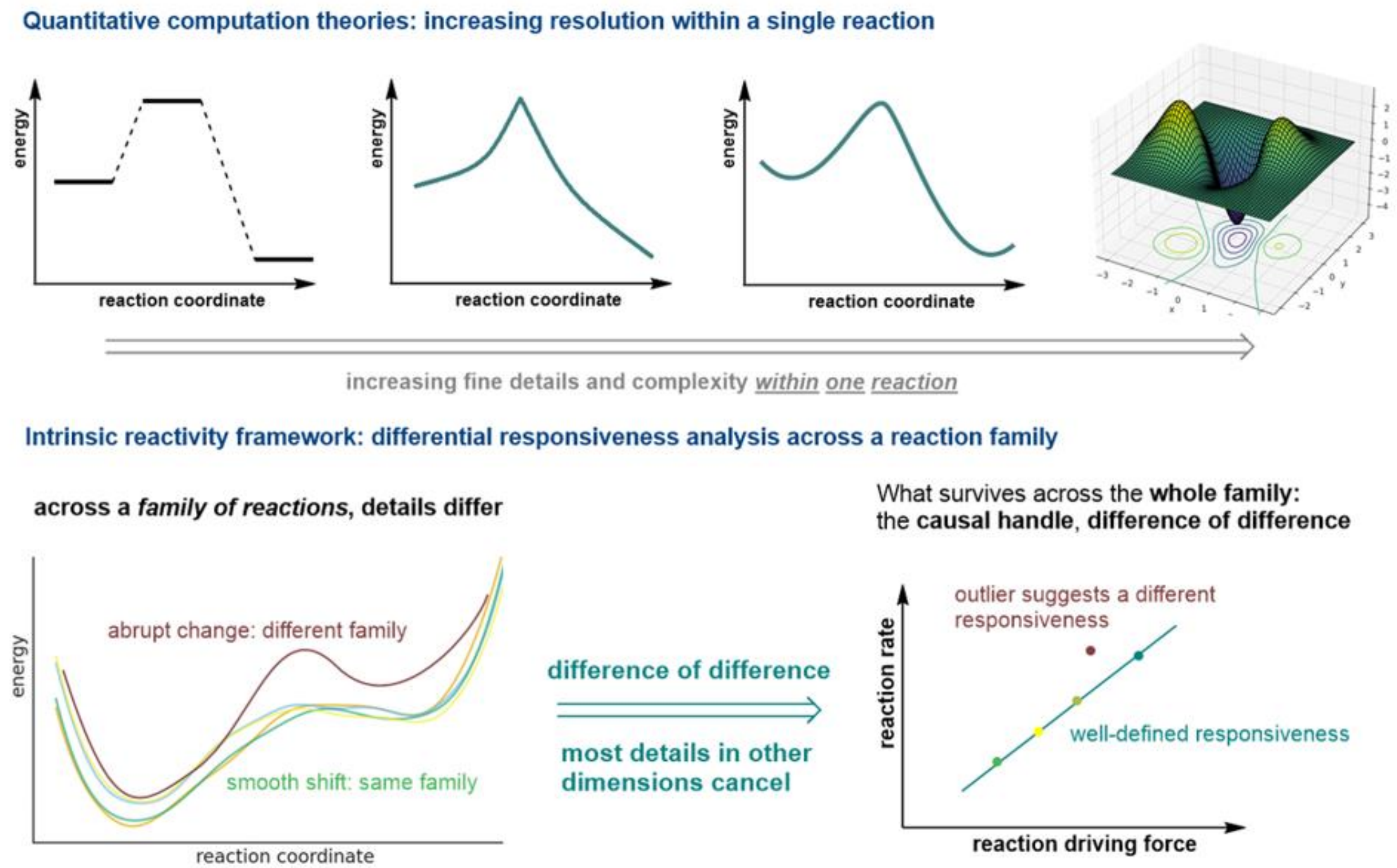

Figure S7. Conceptual comparison between quantitative reaction-rate theories and differential responsiveness analysis.

Quantitative reaction-rate theories generally aim to achieve increasingly accurate descriptions of a single reaction by improving the representation of the potential energy surface, increasing dimensionality, and incorporating more detailed dynamical effects (**Figure S7**, top). Such approaches are essential for quantitative prediction of absolute rate constants. In contrast, the present responsiveness analysis examines a family of intrinsically similar reactions under controlled thermodynamic perturbations (**Figure S7**, bottom). *Rather than resolving every contribution to the absolute reaction rate, it identifies the components of reactivity that change systematically with the driving force* and therefore survive the differential comparison. Within an intrinsic reaction family, contributions that remain approximately constant across the series become part of the intrinsic reactivity baseline, whereas irregular perturbations are expected to manifest as deviations from the Brønsted relationship. Consequently, the rate–driving force responsiveness

represents a “difference of differences” that isolates the systematic response of the reaction family to thermodynamic perturbation.

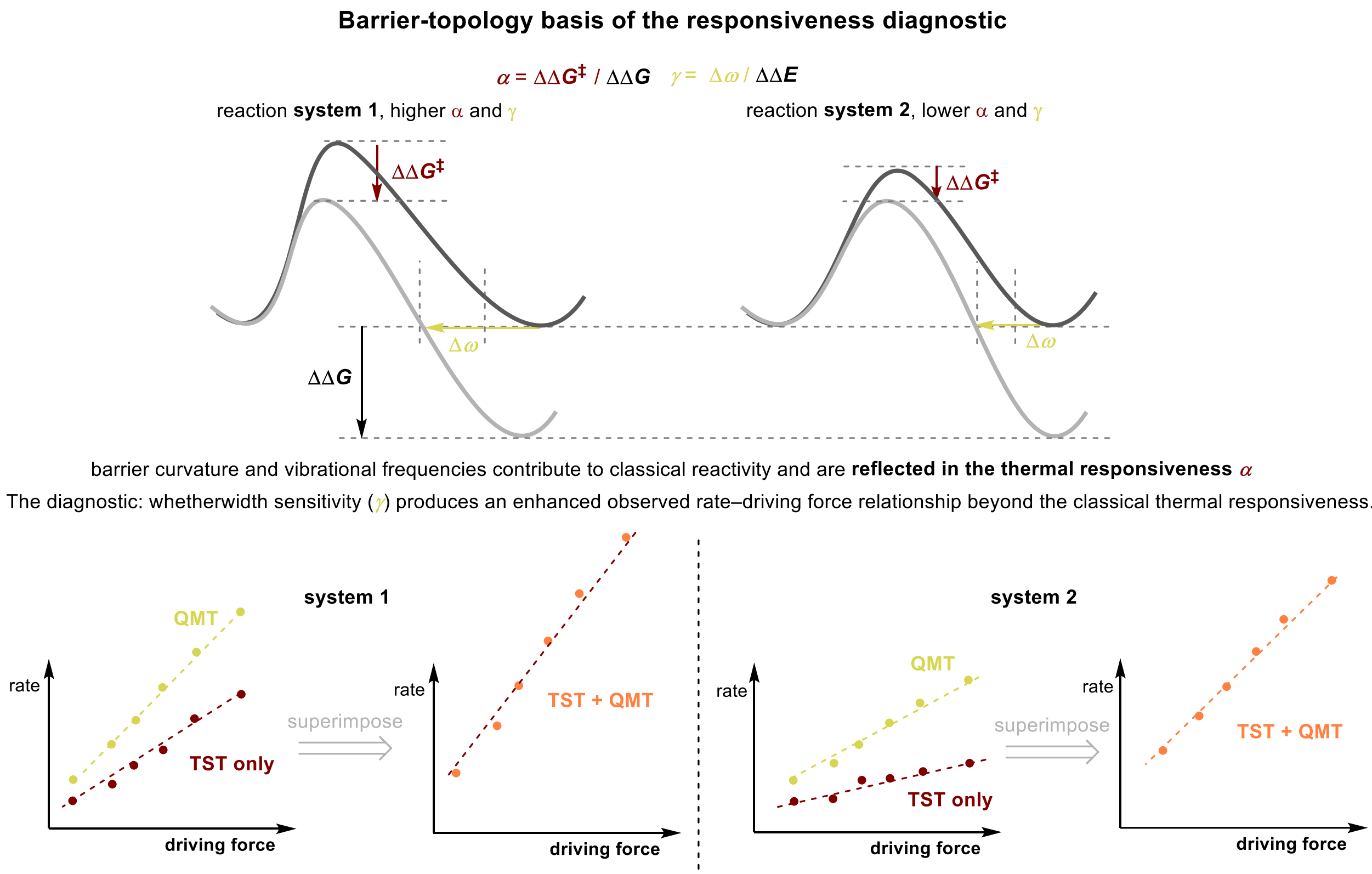

Figure S8. Barrier-topology origin of classical and tunneling rate–driving force responsiveness.

Different reaction families can possess distinct intrinsic barrier topologies, resulting in different classical thermal responsiveness α_{TST} to the same thermodynamic driving-force perturbation (**Figure S8**, system 1 vs system 2). Such differences may arise from variations in barrier curvature, vibrational frequencies, and other features affecting over-the-barrier reactivity, and are therefore inherently included in the corresponding classical reference. In addition to this thermal contribution, through-the-barrier reactivity exhibits sensitivity to the response of barrier width to the same thermodynamic perturbation, represented by the width responsiveness $\gamma=\Delta\omega/\Delta\Delta E$. The barrier-width sensitivity is an intrinsic feature of barrier topology under thermodynamic perturbation. The diagnostic tests whether that width sensitivity is translated into observable rate–driving force responsiveness, which occurs when through-the-barrier reactivity contributes significantly to the measured rate.

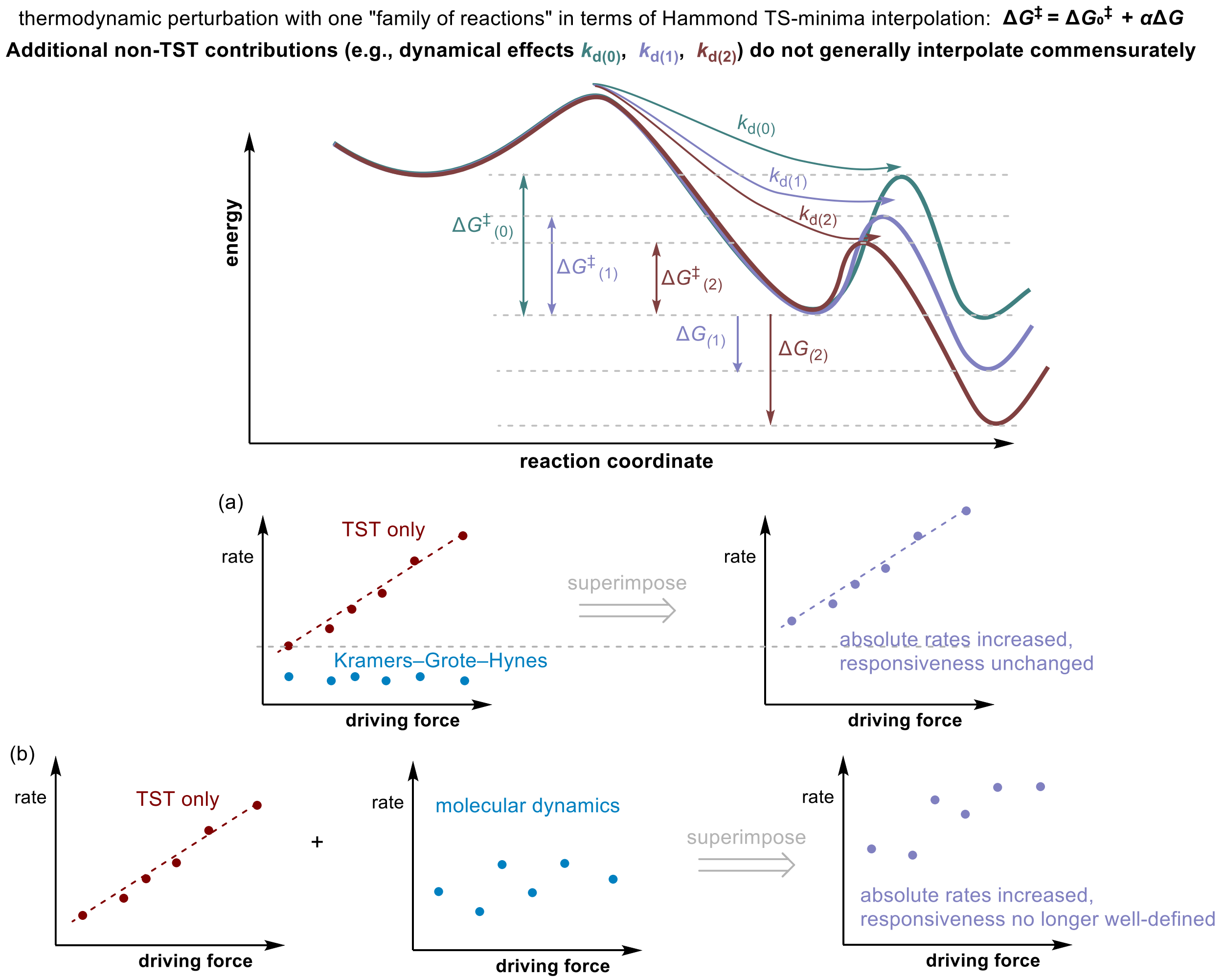

Figure S9. Influence of additional non-TST contributions on the rate–driving force responsiveness.

Additional contributions to the reaction rate beyond the classical transition-state description can modify the absolute reaction rate. However, within a homologous reaction family, these contributions influence the differential responsiveness only if they exhibit a systematic dependence on the same thermodynamic perturbation (**Figure S9**). Family-invariant additional contributions result in a constant shift of the absolute rate but preserve the original rate–driving force responsiveness (**Figure S9a**). Additional contributions that vary non-systematically with the thermodynamic perturbation lead to increased scatter or deterioration of the Brønsted relationship (**Figure S9b**). The trajectory sketches are schematic illustrations of possible non-TST dynamical effects, which are not generally expected to follow the same Hammond-type interpolation as the IRC-related barrier response. Therefore, unlike the barrier-height and barrier-width responses encoded in the reaction coordinate, additional non-TST contributions are unlikely to introduce a coherent additional responsiveness.

Evaluation of other non-classical effects in responsiveness analysis

As explained in the previous section, responsiveness analysis identifies the systematic changes of rate with respect to driving force. For an alternative non-classical effect to distort the conclusions about tunneling contribution, it must display a large enough, monotonic, same-sign enhancement of rate–driving-force responsiveness across a preserved reaction family. Below we examine common potential disrupters to determine the presence and magnitude of their effect over the mapped driving force range.

Necessary conditions for an alternative mechanism

> Is the effect systematically modulated by the remote driving-force perturbation?
>
> • Δ*G*-coupled? • Same-sign? • Large enough?

a) b)

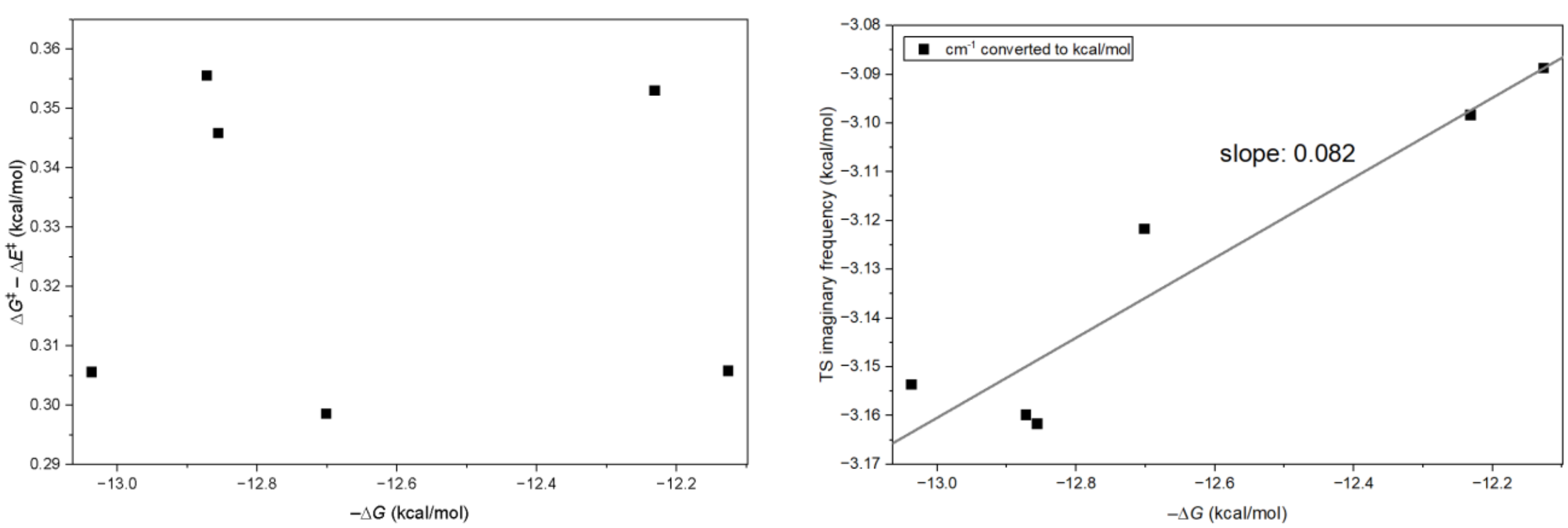

Figure S10. Tests for alternative driving-force-coupled contributions to responsiveness. (a) Aggregate thermochemical correction, $\Delta G^{\ddagger}-\Delta E^{\ddagger}$, across the substituent series. (b) Transition-state imaginary frequency, converted to an hcν energy scale, as a local barrier-curvature descriptor. Both quantities are evaluated against the same driving-force perturbation used in the responsiveness analysis. Level of theory: B3LYP-D3(BJ)/def2-TZVP.

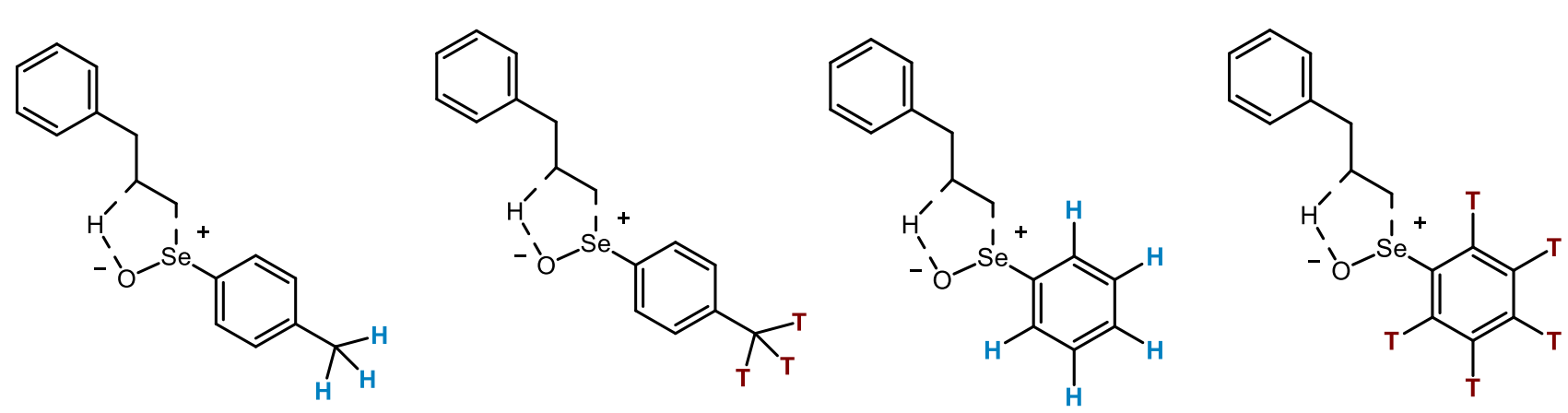

2b-TS **2b-t3-TS** **2c-TS** **2c-t5-TS**

Table S5. Vibrational frequencies and their differences for isotopic pairs, at B3LYP-D3(BJ)/def2-TZVP level of theory

2b-TS	2b-*t3*-TS[a]	diffr.	2c-TS	2c-*t5*-TS[a]	diffr.
**-1105.2**	**-1105.2**	**0**	**-1105.8**	**-1105.7**	**-0.1**
20.14	19.8	0.34	19.93	19.52	0.41
38.93	25.09	13.84	36.79	32.35	4.44
41.04	38.86	2.18	45.63	44.26	1.37
45.9	41.1	4.8	54.18	52.43	1.75
51.21	50.51	0.7	84.78	81.58	3.2
78.9	76.64	2.26	113.01	111.11	1.9
106.57	102.35	4.22	131.28	126.02	5.26
114.62	113.03	1.59	169.83	164.23	5.6
154.3	151.56	2.74	204.56	186.06	18.5
174.18	167.67	6.51	254	248.25	5.75
243.85	232.59	11.26	271.66	263.3	8.36
259.55	246.69	12.86	292.92	288.27	4.65
264.31	260.32	3.99	325.42	306.06	19.36
293.41	289.59	3.82	347.08	312.24	34.84
334.77	305.1	29.67	405.21	346.85	58.36
346.46	343.27	3.19	418.74	386.92	31.82

375.79	348.58	27.21	426.97	418.6	8.37
408.9	408.54	0.36	447.8	426.94	20.86
418.85	418.84	0.01	481.2	447.7	33.5
426.86	426.83	0.03	499.73	471.67	28.06
448.87	448.04	0.83	555.45	499.56	55.89
497.82	475.54	22.28	611.39	555.26	56.13
500.57	499.28	1.29	630.22	562.94	67.28
556.03	546.67	9.36	637.85	575.11	62.74
598.86	557.8	41.06	684.9	593.22	91.68
611.77	611.73	0.04	706.04	610.65	95.39
637.74	637.74	0	716.92	611.66	105.2
649.8	647.32	2.48	752.6	637.85	114.7
716.77	663.13	53.64	760.64	687.44	73.2
723.46	714.17	9.29	764.07	711.47	52.6
759.7	716.81	42.89	796.96	715.74	81.22
763.59	759.04	4.55	829.73	716.92	112.8
795.88	763.29	32.59	854.34	717.64	136.7
812.7	764.04	48.66	858.71	745.78	112.9
823.07	773.32	49.75	863.61	750.45	113.1
830.15	796.09	34.06	922.01	759.21	162.8
852.65	830.04	22.61	934.19	763.73	170.4
854.42	852.18	2.24	942.82	796.9	145.9
863.53	854.34	9.19	986.63	814.58	172.0
922.22	863.5	58.72	989.73	829.86	159.8
943.17	874.71	68.46	994.05	854.46	139.5
969.92	880.85	89.07	1004.49	863.48	141.0
986.53	882.61	103.9	1006.29	922.03	84.26
987.59	922.23	65.36	1008.12	926.58	81.54
994.71	943.18	51.53	1021.65	932.93	88.72
1004.82	959.67	45.15	1024.18	942.79	81.39
1007.13	976.84	30.29	1044.27	986.65	57.62
1008.98	986.59	22.39	1052.2	993.77	58.43
1024.14	988.09	36.05	1087.13	1003.66	83.47
1037.95	994.76	43.19	1096.5	1006.18	90.32
1052.27	1004.85	47.42	1098.98	1010.47	88.51
1065.21	1007.13	58.08	1102.25	1024.17	78.08
1089.78	1024.14	65.64	1172.46	1052.19	120.2
1099.18	1038.15	61.03	1174.06	1098.74	75.32
1102.14	1052.27	49.87	1193.14	1102.25	90.89
1134.21	1090.06	44.15	1194.83	1172.46	22.37
1172.43	1099.18	73.25	1202.92	1194.77	8.15
1194.76	1102.14	92.62	1227.44	1202.92	24.52
1200.38	1129.75	70.63	1264.5	1227.42	37.08

1203.24	1172.43	30.81	1302.89	1264.48	38.41
1227.29	1194.76	32.53	1317.53	1286.25	31.28
1234.48	1200.42	34.06	1319.06	1303.34	15.72
1264.47	1203.24	61.23	1340.37	1318.01	22.36
1302.9	1227.3	75.6	1347.96	1318.11	29.85
1317.22	1241.48	75.74	1361.88	1323.22	38.66
1318.62	1264.47	54.15	1410.2	1340.39	69.81
1336.13	1302.81	33.32	1470.85	1361.88	108.9
1340.26	1312.88	27.38	1484.95	1410.2	74.75
1361.93	1317.95	43.98	1486.26	1484.95	1.31
1407.39	1332.14	75.25	1509.5	1486.26	23.24
1410.05	1340.26	69.79	1515.97	1515.96	0.01
1423.05	1361.93	61.12	1529.52	1529.52	0
1466.55	1410.05	56.5	1545.24	1545.15	0.09
1474.69	1422.8	51.89	1617.21	1552.83	64.38
1484.89	1484.89	0	1618.4	1559.58	58.82
1486.25	1486.25	0	1623.65	1623.63	0.02
1516.04	1516.03	0.01	1644.06	1644.05	0.01
1524.52	1523.76	0.76	3019.55	2001.73	1017
1529.51	1529.5	0.01	3058.99	2012.04	1046
1545.15	1545.15	0	3077.28	2022.12	1055
1604.79	1599.09	5.7	3144.12	2029.99	1114
1623.51	1623.5	0.01	3159.43	2038.36	1121
1631.94	1631.17	0.77	3168.19	3019.55	148.6
1643.75	1643.75	0	3169.66	3058.99	110.6
3019.19	1816.96	1202	3173.9	3077.28	96.62
3026.55	1943.81	1082	3175.41	3144.12	31.29
3058.26	1970.63	1087	3182.41	3159.43	22.98
3073.87	3019.19	54.68	3183.16	3168.2	14.96
3076.53	3058.26	18.27	3190.6	3173.91	16.69
3107.18	3076.53	30.65	3193.57	3182.41	11.16
3144.33	3144.33	0	3196.25	3193.57	2.68
3159.3	3159.3	0	3227.92	3227.92	0
3162.46	3162.44	0.02			
3166.36	3166.31	0.05			
3167.91	3167.91	0			
3173.64	3173.64	0			
3182.11	3182.11	0			
3182.37	3182.35	0.02			
3188.52	3188.51	0.01			
3193.17	3193.17	0			
3227.96	3227.96	0			

a: Me (2b) or Ph (2c) hydrogens substituted with ^3T

- Vibrational quantization and partitioning

We first examined the aggregate thermochemical correction by evaluating $\Delta G^{\ddagger}-\Delta E^{\ddagger}$ across the substituent series. This quantity contains the standard corrections used to convert the electronic activation barrier into the activation free energy employed in the TST reference, including zero-point energy, thermal vibrational populations, and entropic contributions. If these quantum-statistical vibrational effects were responsible for the enhanced rate–driving-force responsiveness, $\Delta G^{\ddagger}-\Delta E^{\ddagger}$ would itself need to vary coherently and monotonically with thermodynamic driving force. Instead, the correction remains small and nearly constant, spanning only 0.30–0.36 kcal/mol, and shows no coherent monotonic dependence on driving force (**Figure S10a**). Thus, the combined vibrational and thermochemical corrections do not generate the additional responsiveness observed experimentally.

Importantly, zero-point quantization is already included within this correction and therefore within the calculated TST responsiveness. It is not an omitted contribution that should be added separately to α_{TST}. The relevant comparison is therefore between the observed responsiveness and a conventional TST reference that already contains standard vibrational quantization and partition-function effects. Because the aggregate correction shows no systematic driving-force dependence, these effects cannot account for the excess of α_{obs} over α_{TST}. Therefore, these corrections are not modulated coherently by the substituent series.

Vibrational quantization and partitioning

Is the effect systematically modulated by the remote driving-force perturbation?

• ΔG-coupled?	• Same-sign?	• Large enough?
No	N.A.	No

- Mode coupling

Remote para substitution was chosen to vary thermodynamic driving force while minimizing direct perturbation of the reaction center and its local dynamics. Even if spectator vibrations couple to the reaction coordinate to some extent, the relevant question is whether the remote substituents used to tune driving force systematically alter that coupling across the reaction series. This design should preferentially alter the electronic energetics of the reaction family rather than reorganize the reaction coordinate or its coupling to spectator modes. Before examining the actual substituent series, we first examine how readily is the reactive mode perturbed by large changes in the remote vibrational manifold.

To separate vibrational and mass effects from electronic barrier changes, we performed a same-electronic/different-mass control in which remote H atoms were replaced by isotope T, including conversion of the para-methyl group to CT_3 and t5-arene. Isotopic substitution leaves the electronic potential-energy surface and thermodynamic driving force essentially unchanged, while substantially shifting the frequencies of modes involving the substituted atoms. Many non-reactive modes consequently change by tens to more than 1000 cm^{-1} (**Table S5**). In contrast, the TS imaginary frequency remains essentially invariant: −1105.2 versus −1105.2 cm^{-1} for one isotopic pair and −1105.8 versus −1105.7 cm^{-1} for the other.

This control demonstrates that even substantial perturbations of the remote vibrational manifold are not measurably transmitted into the unstable mode defining the reaction coordinate. Thus, generic mode mixing is not readily induced in this reaction family. Consequently, the remote electronic substituents used to tune driving force are unlikely to generate the observed enhanced responsiveness merely through incidental vibrational coupling. The isotope-substitution analysis further supports this reaction-family assignment:

although remote aryl H/T substitution substantially shifts many local vibrational modes, the TS imaginary frequency and imaginary-mode displacement pattern remain essentially unchanged. Thus, the remote aryl modes do not appreciably mix into the imaginary reaction-coordinate mode, consistent with a conserved IRC across the perturbation.

mode coupling

Is the effect systematically modulated by the remote driving-force perturbation?

• ΔG-coupled?	• Same-sign?	• Large enough?
**N.A.**	**N.A.**	**No**

Although the present isotope control indicates that this is not the case for our system, para substitution is not the only possible means of varying thermodynamic driving force. In other reaction families, one can readily verify preservation of the intrinsic reaction coordinate. For example by monitoring the transition-state imaginary frequency, and if necessary, choose an alternative driving-force perturbation substituent site that minimizes changes in vibrational coupling.

- Dynamic transmission coefficient

As a qualitative probe of this possibility, we examined the transition-state imaginary frequency across the substituent series (**Figure S10b**). The imaginary frequency reflects the local curvature of the potential-energy surface along the unstable reaction coordinate and therefore serves as a convenient proxy for changes in the local barrier dynamics that may influence the dynamic transmission coefficient. Across the present reaction family, the imaginary frequency changes smoothly with driving force, corresponding to an effective responsiveness of only 0.082. Importantly, this modest curvature response is consistent with ordinary Hammond-interpolation of a preserved reaction barrier. As the same IRC evolves smoothly with driving force, a small accompanying change in local barrier curvature is expected. Thus, the observed imaginary-frequency trend is naturally interpreted as the normal evolution of the barrier itself rather than evidence for a distinct substituent-dependent dynamical contribution. If the enhanced responsiveness arose primarily from systematic modulation of the dynamic transmission coefficient, a substantially larger substituent-dependent response would be expected. The contrast with the experimental responsiveness is also evident in the quality of the correlation. The imaginary-frequency response shows appreciably more scatter than the experimental rate–driving-force relationship. Thus, the local curvature descriptor neither reproduces the magnitude nor the tight substituent dependence of the observed kinetic response.

We next made a deliberately conservative estimate by assuming that the entire variation in the TS imaginary frequency is transmitted into the dynamical transmission coefficient. This provides an upper-bound estimate for the corresponding contribution to the apparent Brønsted slope α_{TST}. In the **Kramers–Grote–Hynes (KGH)** framework, the dynamic transmission coefficient is κ_{KGH}. As a deliberately generous curvature-based estimate, we assumed that the curvature-dependent component of κ_{KGH} scales directly with $|\nu_{imag}|$, where $\kappa_{KGH} \propto |\nu_{imag}|$, giving $d(\ln \kappa_{KGH})/d(\ln |\nu_{imag}|) = 1$. then

$$k_{obs} = \kappa_{KGH} k_{TST} \text{ where } \kappa_{KGH} \propto |\nu_{imag}|$$

$$d(\hbar|\nu_{imag}|)/d(-\Delta G) = 0.082,\ \hbar|\nu_{imag}| = 3.13 \text{ kcal/mol, T = 294K}$$

$$d \ln |\nu_{imag}| / d(-\Delta G) = [d(\hbar|\nu_{imag}|)/d(-\Delta G)] / \hbar|\nu_{imag}| = 0.082 / 3.13 = 0.0262 \text{ (kcal/mol)}^{-1}$$

$$\boldsymbol{\Delta\alpha}_{\mathbf{KGH}} = -RT \cdot d \ln \kappa_{KGH} / d(-\Delta G) \leq -RT \cdot d \ln |\nu_{imag}| / d(-\Delta G) = 0.584 \times 0.0262 = 0.015$$

This is only 2% of the total increase in the Brønsted slope.

The local imaginary frequency cannot capture changes in the bath-coupling or the solvent friction. However, as we are studying a homologous series of para-substituted compounds in the **same solvent** and at the **same temperature**, the bulk solvent friction is effectively constant. Furthermore, because the substituent change is highly localized and far removed from the reaction center, the systematic variation in how the transition state couples to the solvent or internal vibrational modes is microscopically tiny. Even if the imaginary frequency has only captured, say 10% of the variation in κ_{KGH}, it would still be only 20% of the total increase in the Brønsted slope. A larger dynamical explanation would therefore require another component of κ_{KGH}, such as friction, bath coupling, or conformational gating, to vary monotonically with driving force and with much larger magnitude. Our design has intentionally tried to minimize such effects, and we find no structural or vibrational evidence for such a change.

dynamic transmission coefficient

Is the effect systematically modulated by the remote driving-force perturbation?

• Δ*G*-coupled?	• Same-sign?	• Large enough?
**yes/maybe**	**yes/maybe**	**No**

In summary, the distinction is therefore that tunneling is the only effect considered here whose responsiveness follows directly from the thermodynamic interpolation of the common reaction barrier. All other effects require an additional mechanism by which the remote driving-force perturbation also modulates that contribution. The comparison is summarized in **Table S6**.

Table S6. Summary of the role of non-classical effects in the rate-driving force relationship framework.

Effect	ΔG-coupled?	Same sign?	Large enough?	Conclusion
Vibrational partitioning	No	—	No	excluded
Mode coupling	not detected	—	No	unlikely
Dynamic transmission	weak	maybe	No	insufficient
Tunneling	Yes	Yes	Yes	consistent

NMR of selenides

*(4-methoxyphenyl)(3-phenylpropyl)selane (**1a**)*

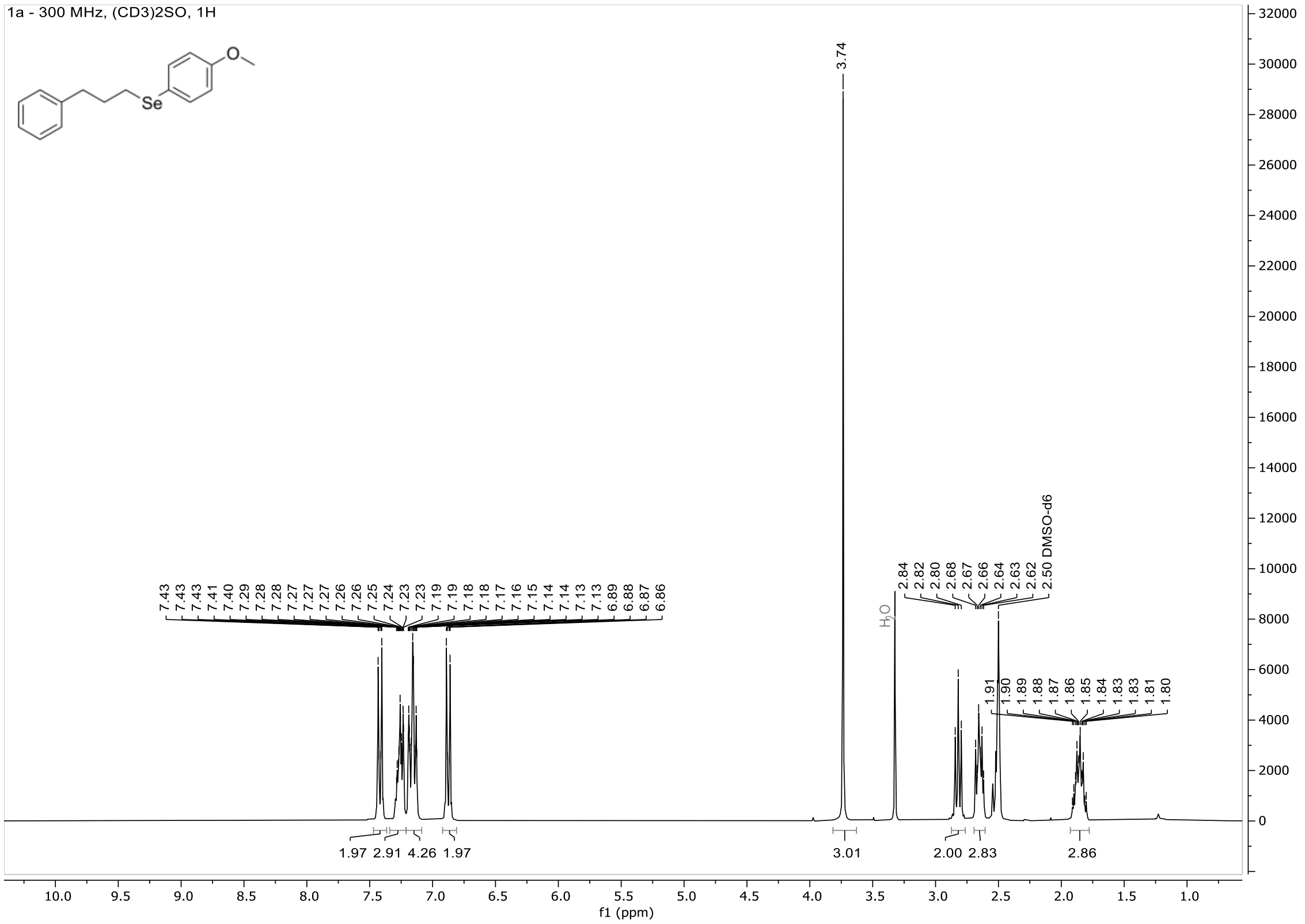

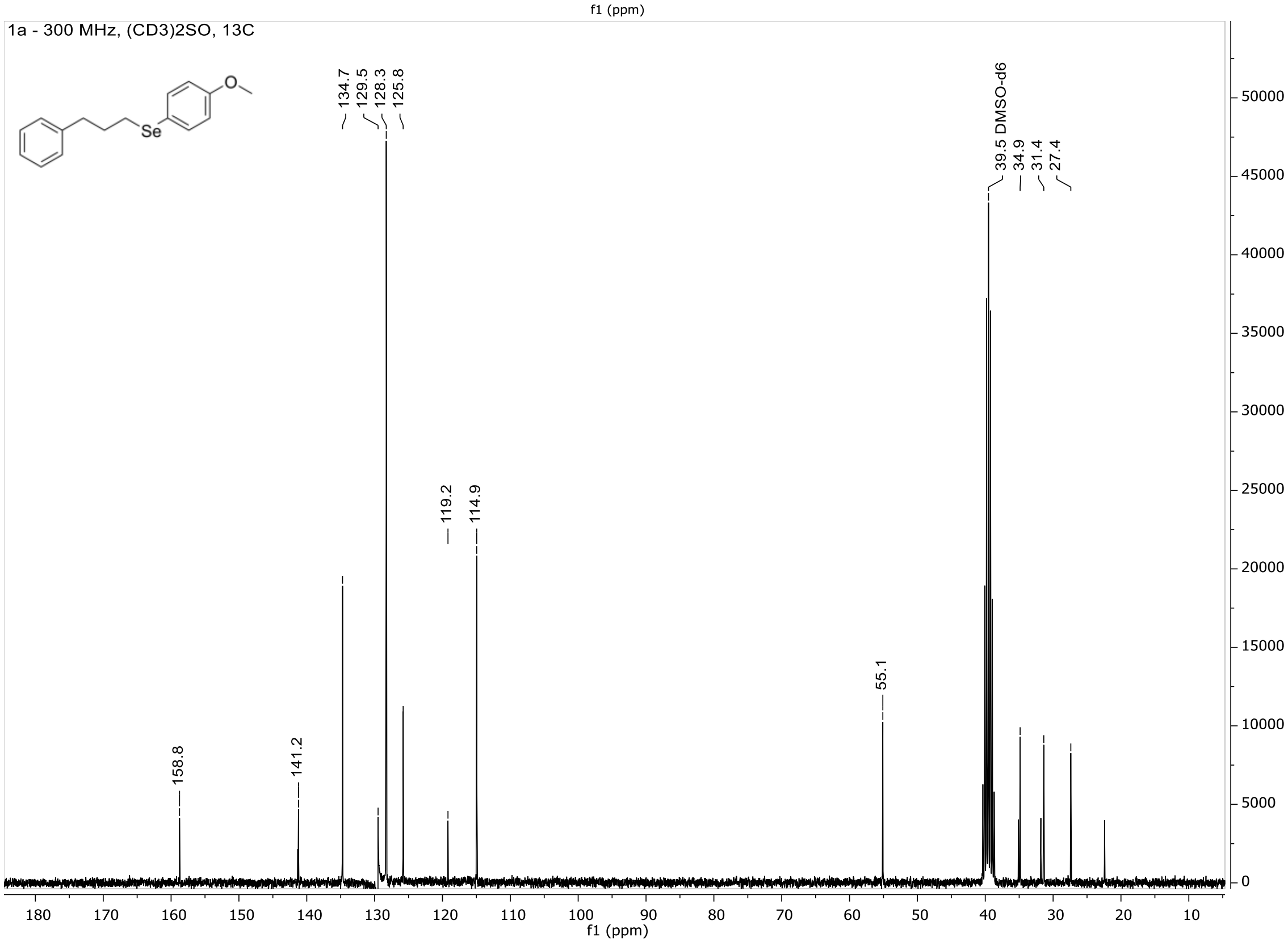

*(3-phenylpropyl)(p-tolyl)selane (**1b**)*

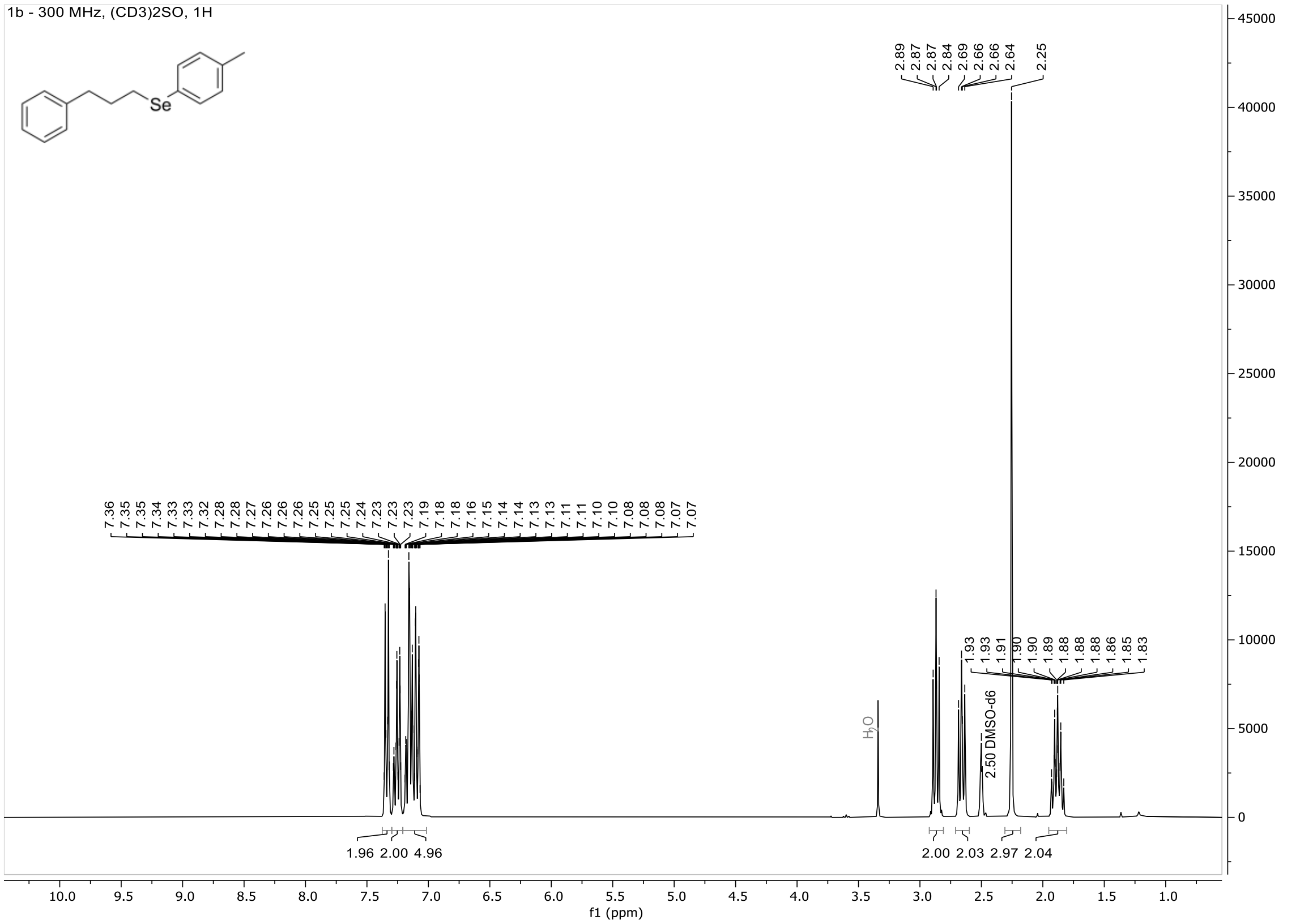

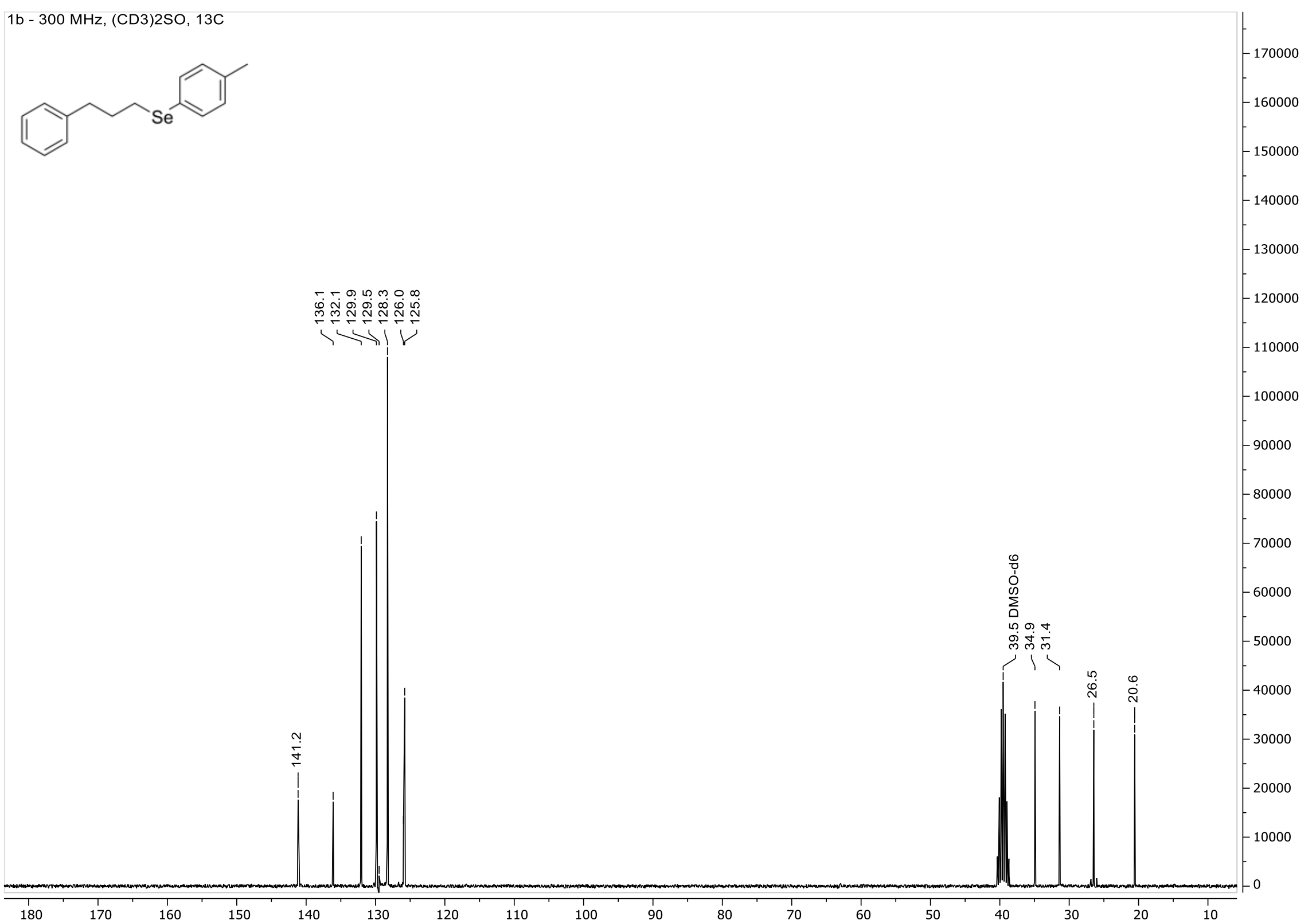

*phenyl(3-phenylpropyl)selane (**1c**)*

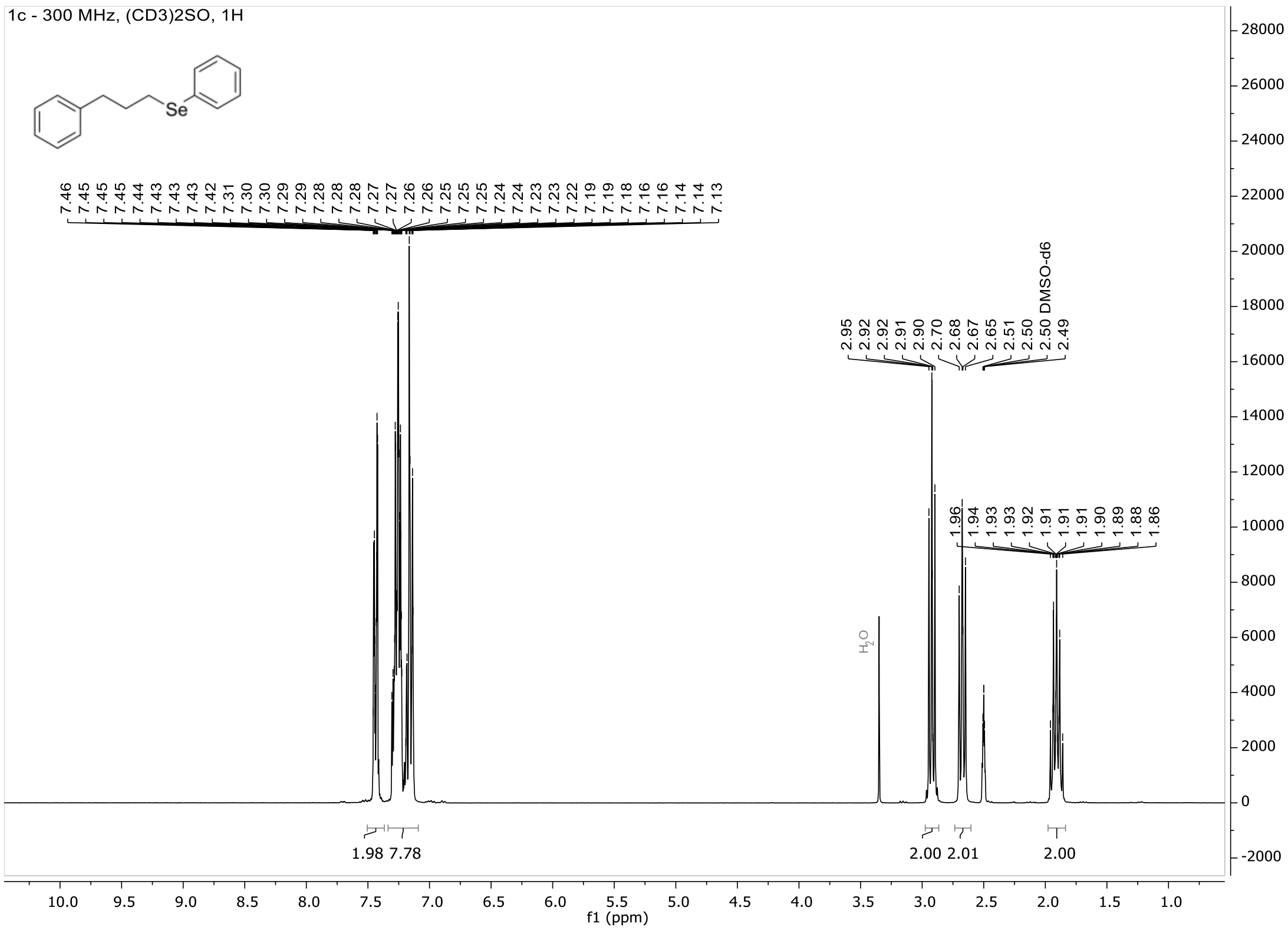

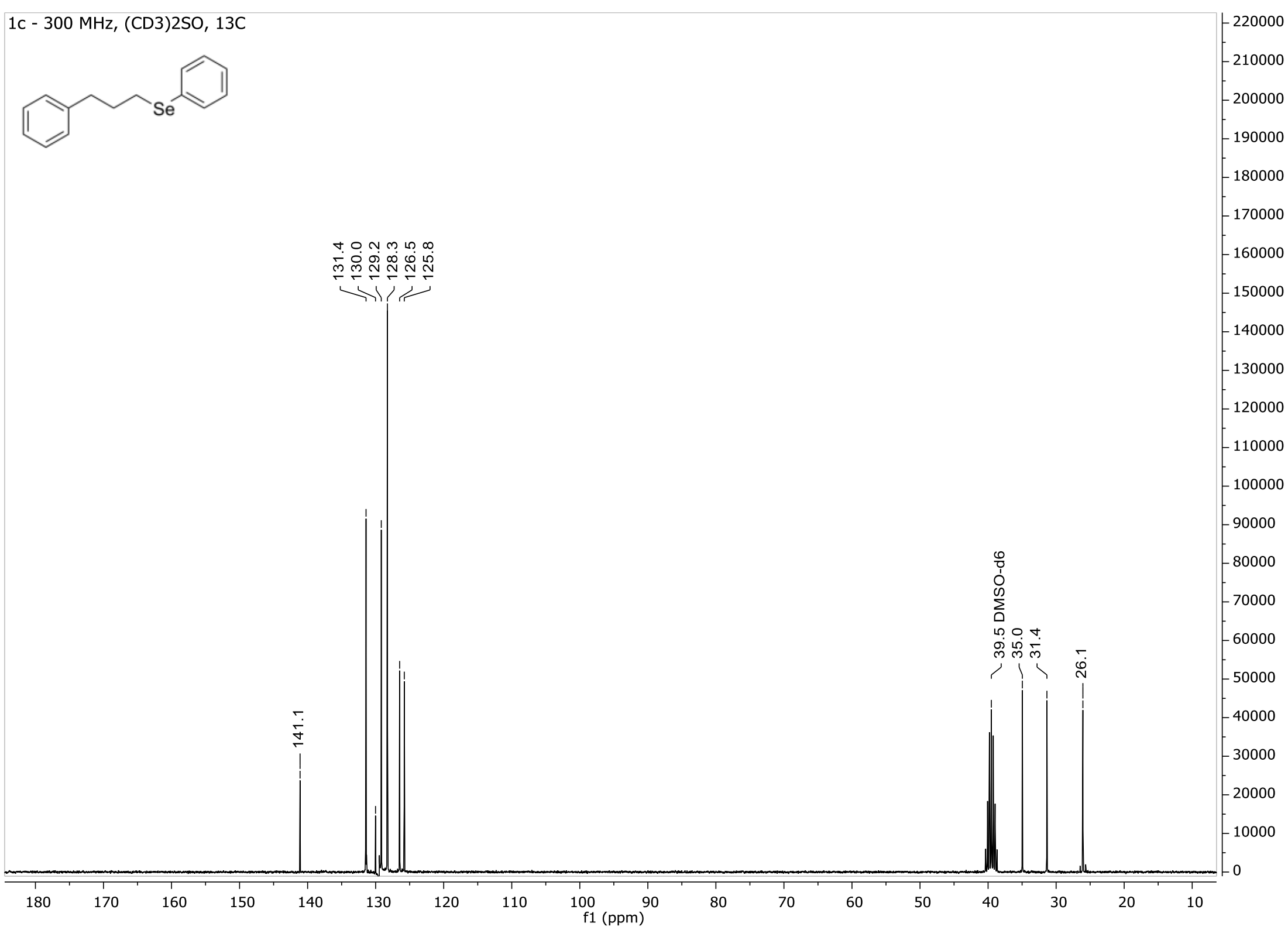

*(4-chlorophenyl)(3-phenylpropyl)selane (**1d**)*

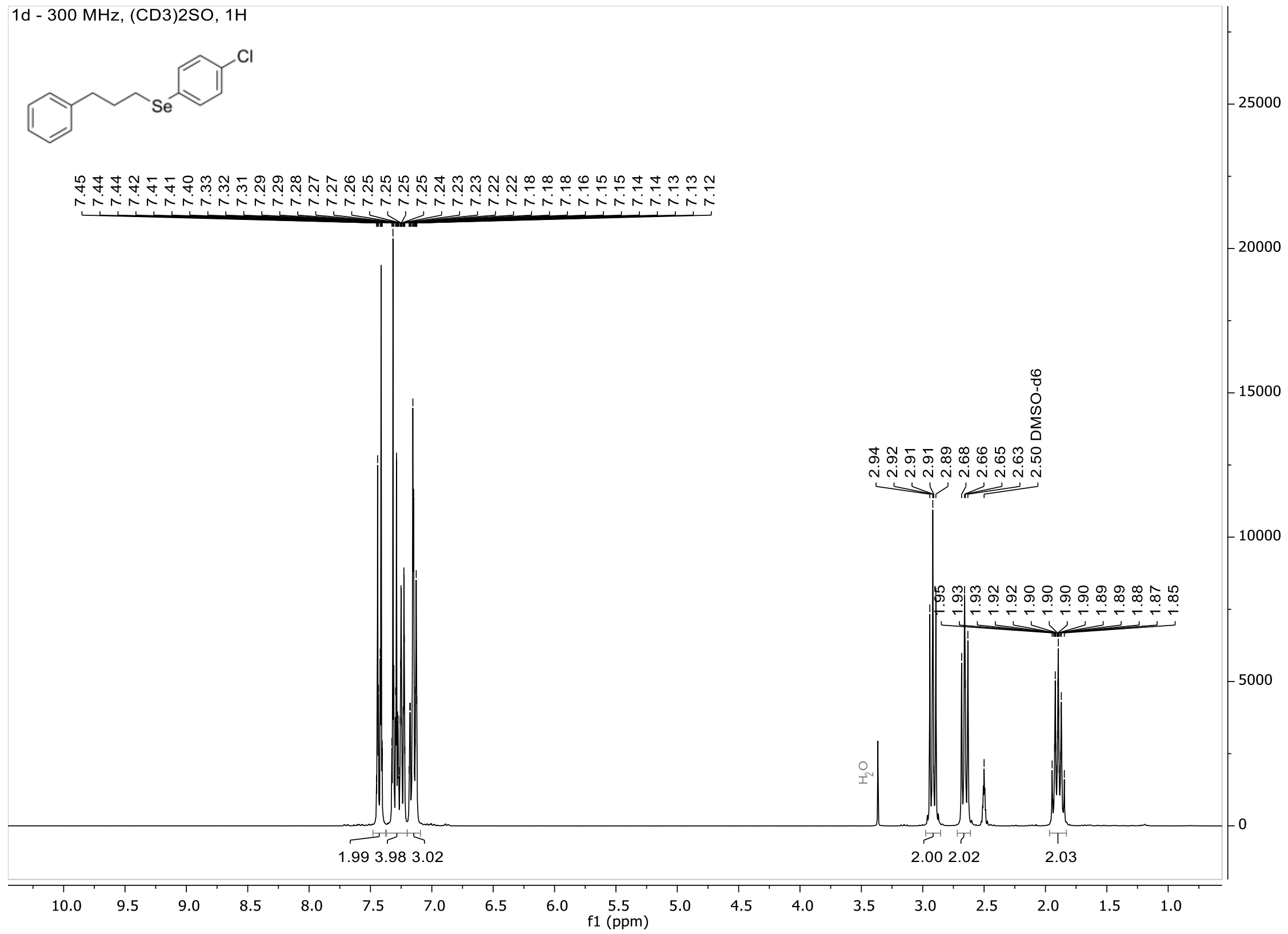

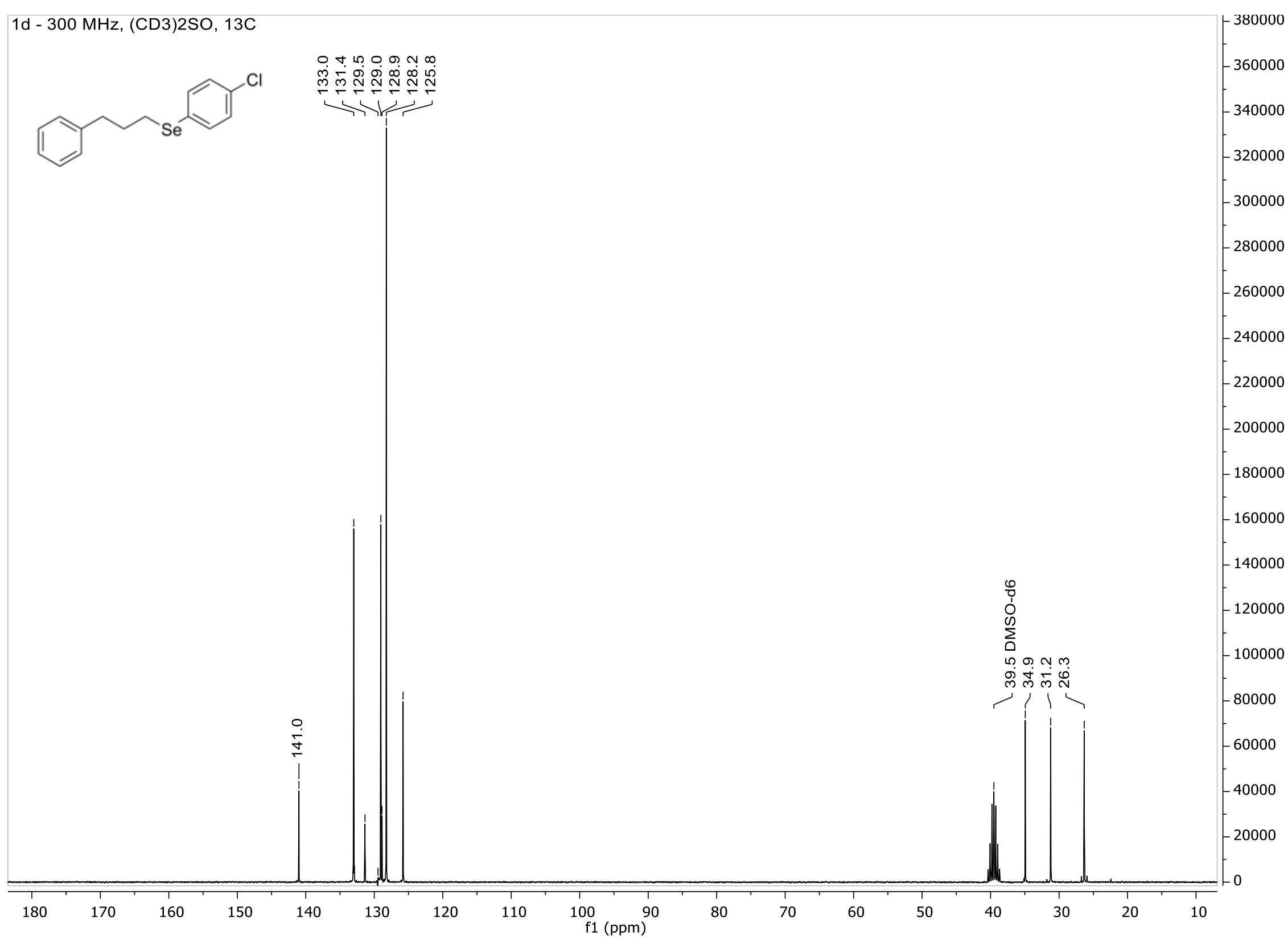

*(3-phenylpropyl)(4-(trifluoromethyl)phenyl)selane (**1e**)*

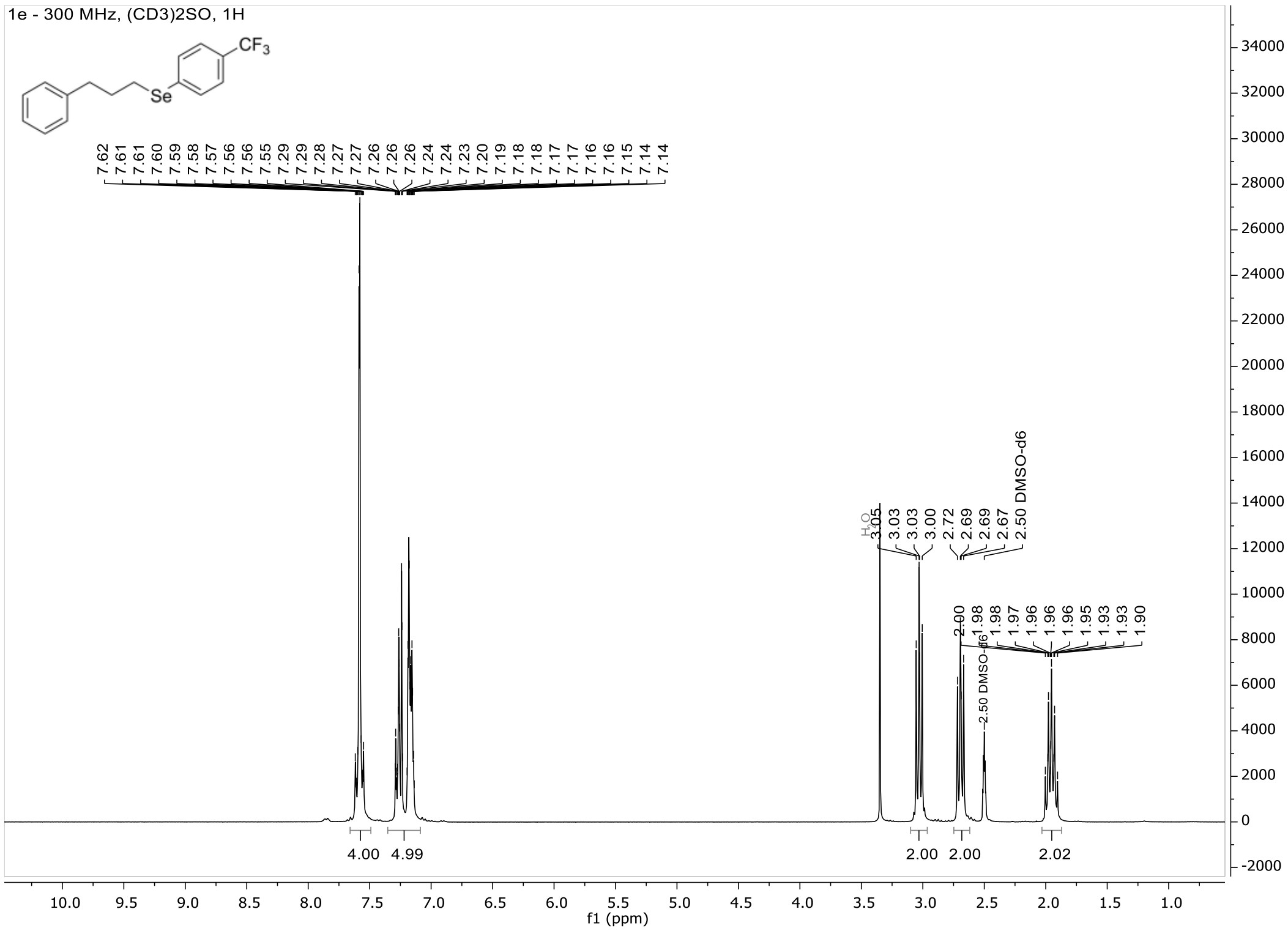

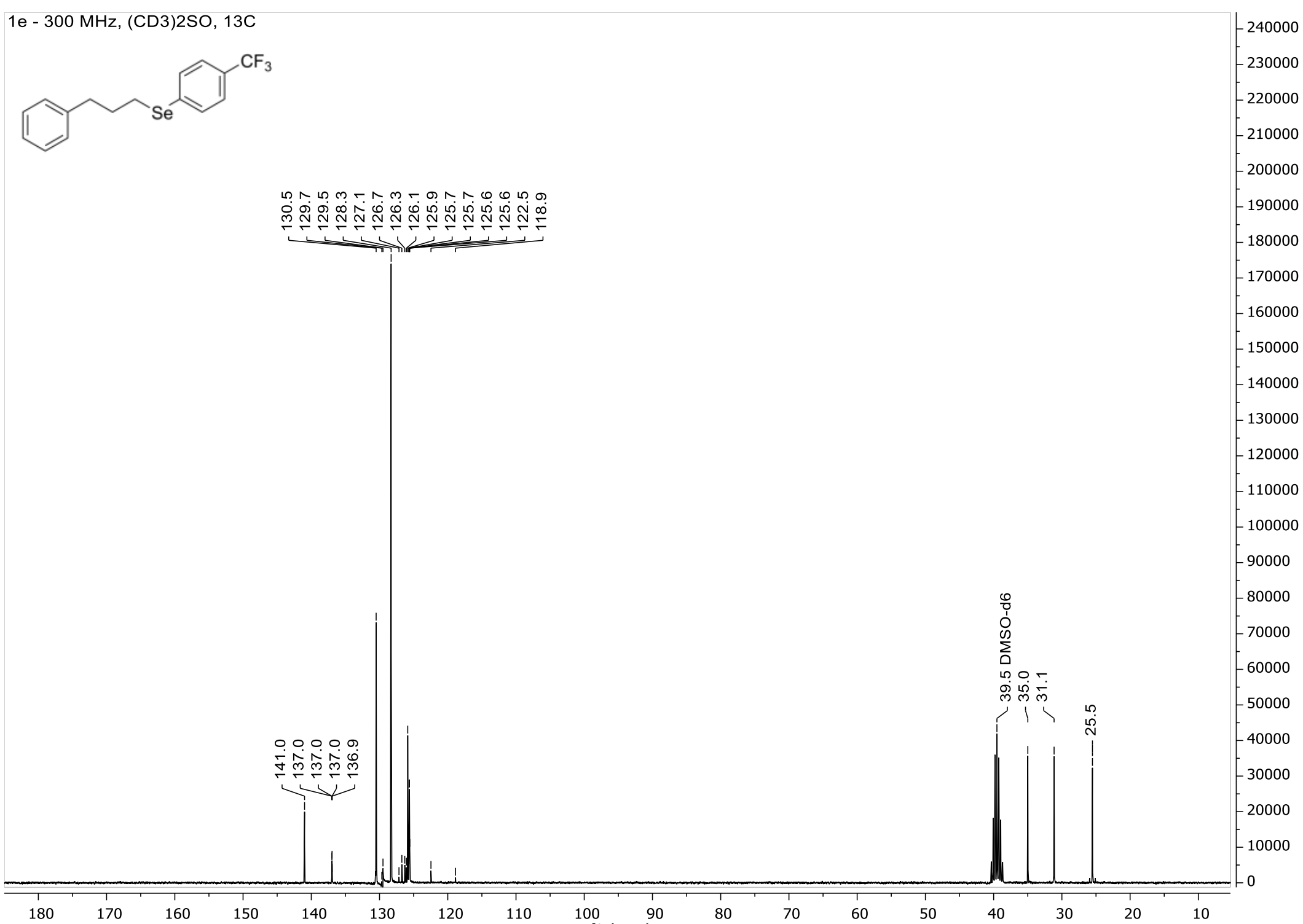

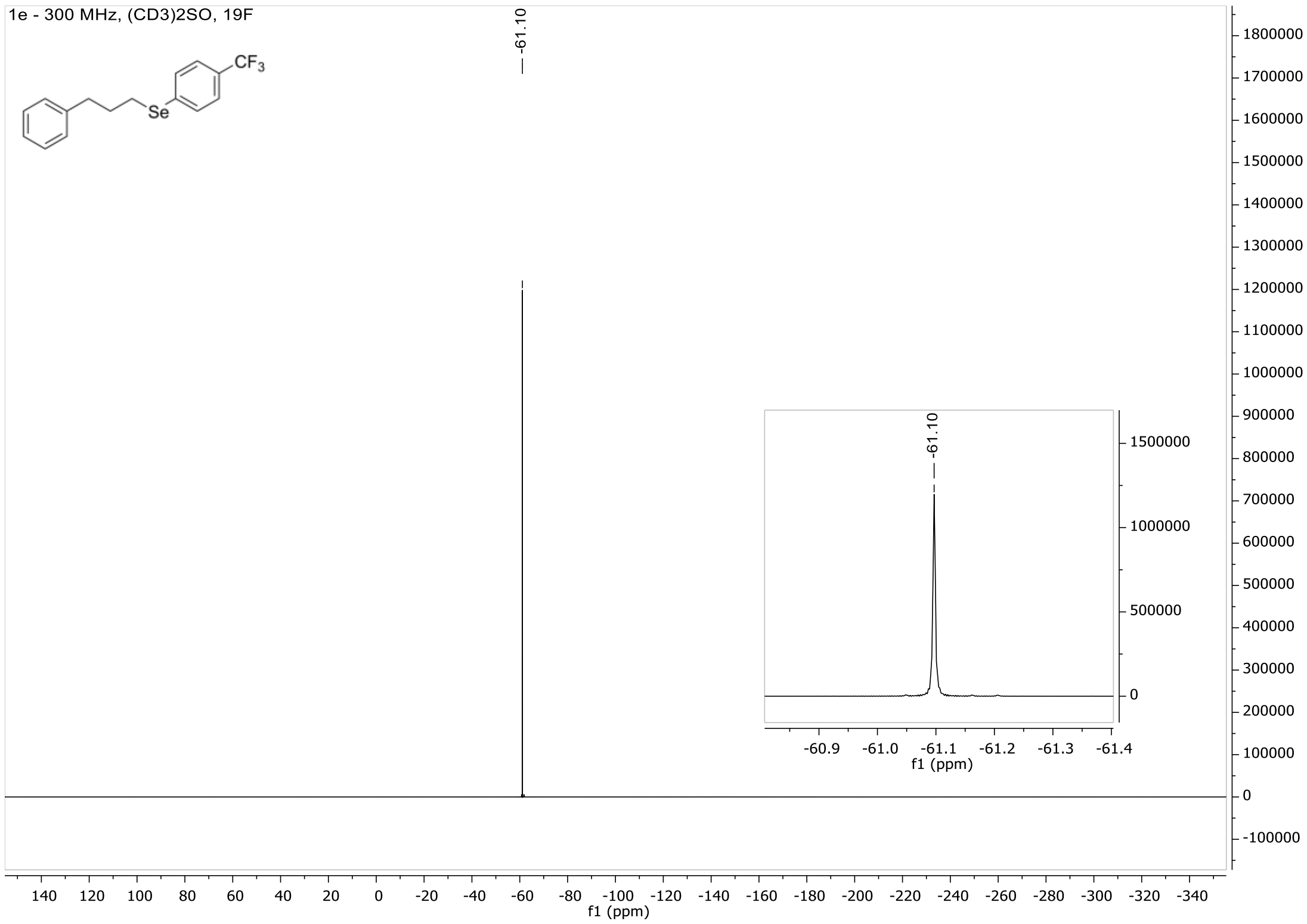
1e - 300 MHz, (CD3)2SO, 19F
CF3
Se
-61.10
f1 (ppm)

*4-((3-phenylpropyl)selanyl)benzonitrile (**1f**)*

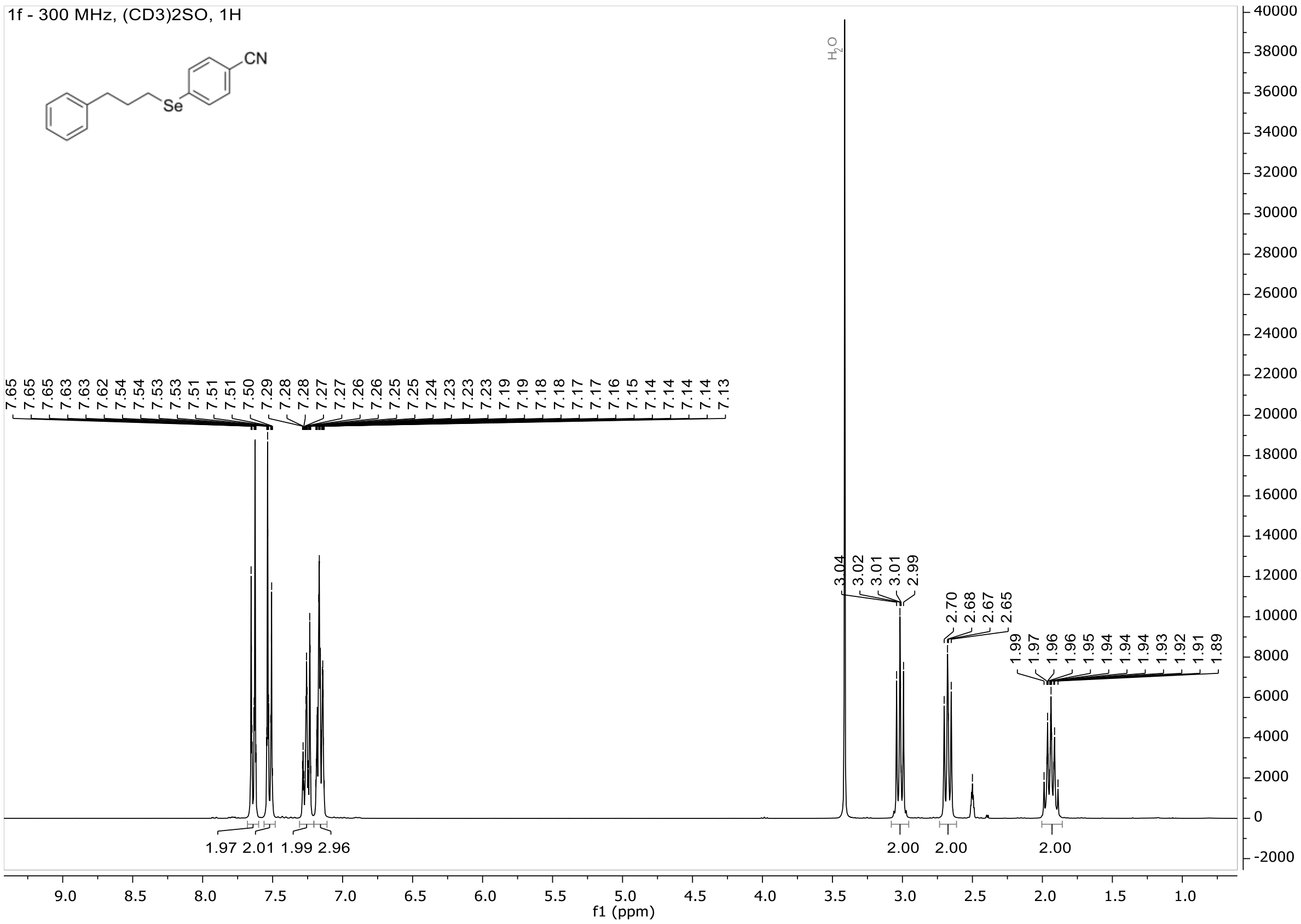

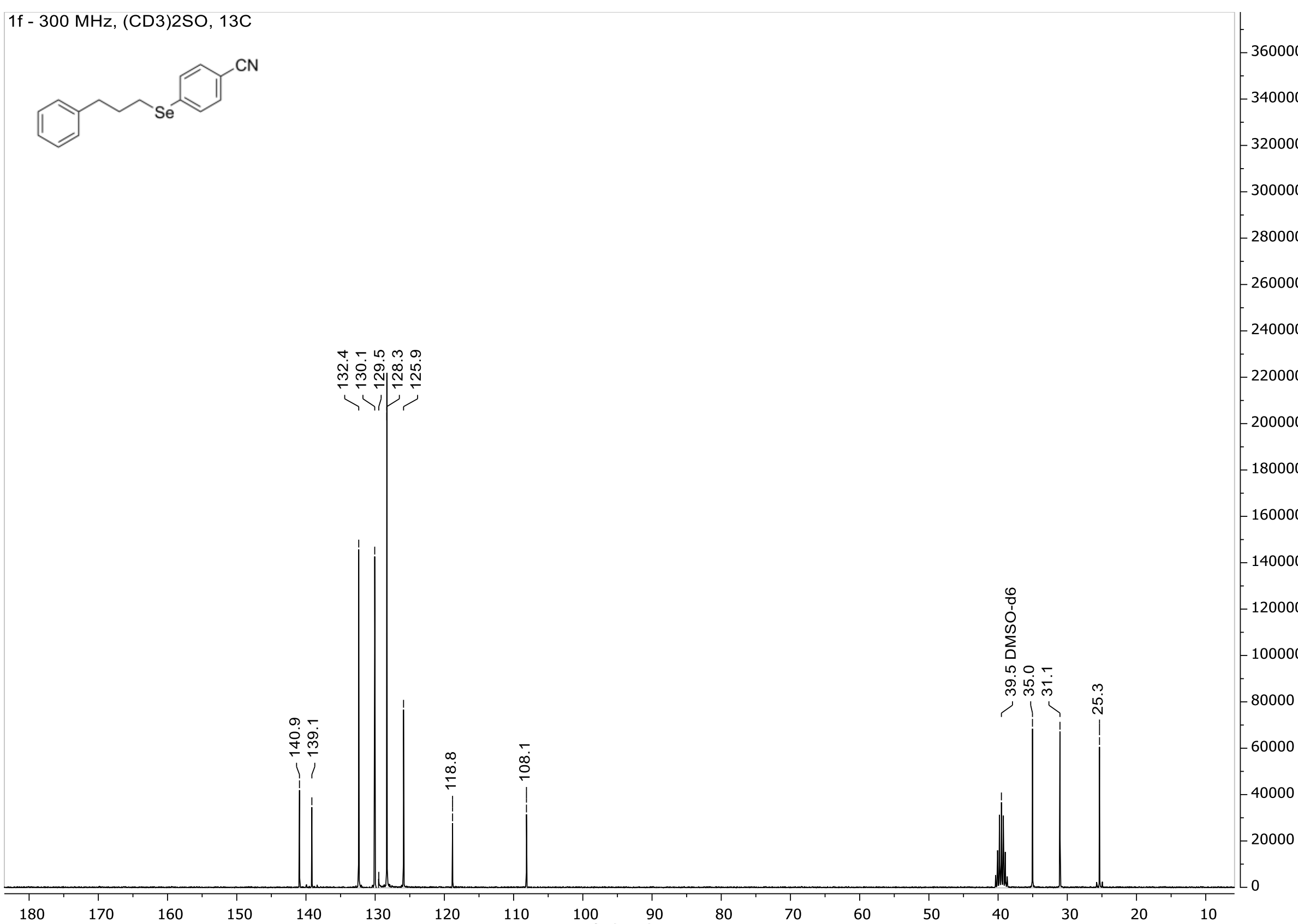

Computational coordinates

IRC point 483 (selenoxides)

R = OMe (**2a**)

Full E -3172.309658109975

C	-1.481156	0.472640	-5.884207
C	-0.896347	-0.098086	-4.749691
C	-0.711015	1.341187	-6.657123
C	0.414041	0.193262	-4.396186
C	0.604926	1.635837	-6.307669
C	1.171831	1.062923	-5.175889
H	-1.475253	-0.782133	-4.139891
H	-1.145081	1.793009	-7.541644
H	0.847333	-0.261646	-3.514218
H	1.186534	2.310662	-6.924031
H	2.197036	1.283546	-4.906127
C	-2.893881	0.109803	-6.266065
H	-3.258645	0.793143	-7.037530
H	-3.545520	0.229117	-5.396299
C	-3.012281	-1.344328	-6.756887
H	-2.552947	-2.001560	-6.016634
H	-4.062405	-1.629652	-6.808066
C	-2.367387	-1.547602	-8.118770
H	-3.073810	-1.434618	-8.940417
H	-1.520603	-0.881695	-8.294777
Se	-1.595056	-3.367816	-8.336624
O	-2.523401	-4.347074	-7.358589
C	-0.011924	-3.014765	-7.254620
C	0.105393	-3.609947	-6.012657
C	1.007226	-2.203096	-7.749879
C	1.242383	-3.391377	-5.236884
C	2.135929	-1.977270	-6.985468
C	2.259803	-2.569394	-5.722374
H	-0.695494	-4.242619	-5.650002
H	0.919215	-1.740200	-8.725689
H	1.318252	-3.855214	-4.264579
H	2.931927	-1.337918	-7.344550
O	3.398189	-2.281070	-5.045714
C	3.583662	-2.858357	-3.755300
H	2.802098	-2.538678	-3.061445
H	3.601623	-3.950008	-3.808678
H	4.547780	-2.498318	-3.402969

R = Me (**2b**)

Full E -3097.098171833848

C	-1.613275	0.567485	-6.009134
C	-1.115193	0.068949	-4.801476
C	-0.791479	1.399765	-6.768417
C	0.160104	0.397200	-4.362771
C	0.490045	1.731024	-6.333653
C	0.969968	1.231029	-5.129354
H	-1.734823	-0.587008	-4.201012
H	-1.157972	1.794816	-7.708937
H	0.525884	-0.002279	-3.425052
H	1.113096	2.377398	-6.939940
H	1.967917	1.482120	-4.792181
C	-2.989708	0.166870	-6.476261
H	-3.294287	0.791717	-7.319720
H	-3.708878	0.341011	-5.671068
C	-3.062963	-1.319120	-6.870913
H	-2.660678	-1.918612	-6.054059
H	-4.103889	-1.618336	-6.983543
C	-2.311410	-1.606630	-8.160624
H	-2.949455	-1.556642	-9.041273
H	-1.456476	-0.945619	-8.312711
Se	-1.514259	-3.428758	-8.194190
O	-2.500675	-4.352022	-7.218503
C	-0.014660	-2.979453	-7.023418
C	0.022762	-3.515353	-5.745945
C	1.016011	-2.172196	-7.486911
C	1.101784	-3.225573	-4.918440
C	2.086034	-1.886624	-6.648467
C	2.145663	-2.406377	-5.353783
H	-0.790181	-4.148251	-5.411797
H	0.988437	-1.761041	-8.489208
H	1.130373	-3.637472	-3.916022
H	2.884590	-1.245812	-7.002517
C	3.284383	-2.061015	-4.436639
H	2.961997	-1.346017	-3.673986
H	3.656241	-2.944187	-3.913034
H	4.112832	-1.611123	-4.984485

R = H (**2c**)

Full E -3057.789201429657

C	-1.674227	0.625796	-6.046774
C	-1.213443	0.148802	-4.815832
C	-0.830972	1.447574	-6.793876
C	0.048688	0.484172	-4.345136
C	0.436669	1.787199	-6.326384
C	0.881056	1.305612	-5.101103
H	-1.850361	-0.498558	-4.224216
H	-1.168896	1.825625	-7.751872
H	0.387167	0.099224	-3.391173
H	1.077053	2.425015	-6.923582
H	1.868450	1.562796	-4.738295
C	-3.027447	0.202225	-6.558010
H	-3.307133	0.809802	-7.421844
H	-3.779762	0.377379	-5.784231
C	-3.063910	-1.290547	-6.932271
H	-2.689620	-1.871414	-6.088597
H	-4.095345	-1.606784	-7.085869
C	-2.251858	-1.587440	-8.182307
H	-2.845535	-1.525800	-9.094180
H	-1.380360	-0.938964	-8.291913
Se	-1.485929	-3.423694	-8.180991
O	-2.525119	-4.325301	-7.240865
C	-0.023136	-3.000221	-6.949925
C	-0.079921	-3.499409	-5.658963
C	1.063722	-2.253348	-7.390397
C	0.970201	-3.234711	-4.783538
C	2.105909	-1.989807	-6.509652
C	2.059472	-2.481583	-5.207579
H	-0.938409	-4.085240	-5.354512
H	1.096937	-1.874158	-8.404924
H	0.933935	-3.614031	-3.769852
H	2.951819	-1.399310	-6.838193
H	2.872365	-2.273380	-4.523373

R = Cl (**2d**)

Full E -3517.358722788074

C	-1.563718	0.556623	-6.013070
C	-1.007353	0.064872	-4.828455
C	-0.787040	1.400239	-6.806607
C	0.280523	0.412166	-4.444470
C	0.506970	1.749955	-6.427220
C	1.044494	1.257765	-5.244352
H	-1.591262	-0.599858	-4.202444
H	-1.199570	1.789999	-7.729972
H	0.693639	0.017097	-3.524956
H	1.093816	2.405508	-7.059080
H	2.052487	1.521675	-4.949830
C	-2.953119	0.138701	-6.423340
H	-3.308463	0.776382	-7.235528
H	-3.636696	0.280148	-5.581150
C	-3.018421	-1.338910	-6.849641
H	-2.578970	-1.949796	-6.057405
H	-4.059333	-1.651547	-6.935713
C	-2.304734	-1.584515	-8.169499
H	-2.969341	-1.519932	-9.028960
H	-1.461644	-0.912260	-8.330486
Se	-1.493181	-3.395455	-8.278361
O	-2.475313	-4.365790	-7.347400
C	-0.013912	-2.973632	-7.067682
C	-0.010718	-3.537826	-5.803057
C	1.023361	-2.150869	-7.489980
C	1.040660	-3.268044	-4.932677
C	2.074975	-1.874758	-6.627687
C	2.068260	-2.438367	-5.357660
H	-0.830034	-4.179448	-5.503574
H	1.017555	-1.715524	-8.481817
H	1.054885	-3.692320	-3.938127
H	2.882921	-1.225758	-6.934719
Cl	3.386691	-2.077294	-4.264958

R = CF_3 (**2e**)

Full E -3394.868320671450

C	-1.565950	0.588549	-5.984194
C	-0.956861	0.109900	-4.820495
C	-0.836429	1.445753	-6.807415
C	0.338716	0.479996	-4.487216
C	0.465078	1.818466	-6.478951
C	1.056904	1.336326	-5.317972
H	-1.505126	-0.563352	-4.171613
H	-1.291527	1.825352	-7.714921
H	0.793002	0.095616	-3.583178
H	1.015235	2.483901	-7.133240
H	2.070566	1.618888	-5.063118
C	-2.956231	0.133834	-6.348040
H	-3.359762	0.766966	-7.141252
H	-3.613982	0.243106	-5.482278
C	-2.985446	-1.340501	-6.789228
H	-2.506873	-1.944692	-6.015762
H	-4.016938	-1.686096	-6.854553
C	-2.296060	-1.545036	-8.128021
H	-2.979149	-1.466641	-8.971733
H	-1.465423	-0.858357	-8.289373
Se	-1.470665	-3.341959	-8.303008
O	-2.426657	-4.348400	-7.384737
C	0.032108	-2.944035	-7.106449
C	0.074020	-3.570942	-5.873169
C	1.054673	-2.099132	-7.523152
C	1.161772	-3.353380	-5.033784
C	2.137581	-1.880346	-6.686432
C	2.193295	-2.518206	-5.447624
H	-0.737111	-4.225747	-5.580790
H	1.013163	-1.613419	-8.490054
H	1.204535	-3.839139	-4.068784
H	2.937054	-1.221807	-6.998140
C	3.370827	-2.260742	-4.553274
F	3.284105	-1.063205	-3.919243
F	3.508712	-3.191827	-3.588003
F	4.536046	-2.234325	-5.240742

R = CN (**2f**)

Full E -3150.030345010191

C	-1.553415	0.536725	-5.974926
C	-1.005526	0.034018	-4.790858
C	-0.772138	1.390000	-6.753564
C	0.277984	0.381518	-4.392206
C	0.517662	1.739283	-6.359785
C	1.046337	1.237301	-5.177007
H	-1.593471	-0.637508	-4.175837
H	-1.178187	1.788994	-7.675912
H	0.683643	-0.019742	-3.471922
H	1.107831	2.403098	-6.979844
H	2.050458	1.502211	-4.870366
C	-2.939030	0.121561	-6.400025
H	-3.294855	0.773809	-7.201791
H	-3.625711	0.238738	-5.559986
C	-2.994965	-1.346826	-6.858347
H	-2.553638	-1.971933	-6.079332
H	-4.033249	-1.662903	-6.953244
C	-2.279480	-1.557307	-8.183610
H	-2.947068	-1.474882	-9.041805
H	-1.438835	-0.879011	-8.329678
Se	-1.462861	-3.362207	-8.339056
O	-2.439423	-4.360967	-7.434739
C	0.008152	-2.954933	-7.107182
C	0.010553	-3.567908	-5.864518
C	1.030196	-2.097183	-7.498268
C	1.049807	-3.311987	-4.981687
C	2.069472	-1.835330	-6.622556
C	2.078861	-2.445762	-5.362585
H	-0.801678	-4.232044	-5.597640
H	1.016509	-1.627520	-8.473770
H	1.066121	-3.770787	-4.002427
H	2.866561	-1.160504	-6.901282
C	3.138535	-2.155404	-4.452262
N	3.993377	-1.909579	-3.718875

IRC point 242 (TS)

<u>R = OMe (**2a-TS**)</u>

Full E -3172.269476451928

C	-1.683156	0.330218	-6.001045
C	-1.154375	-0.445008	-4.968043
C	-0.865885	1.299778	-6.586357
C	0.148745	-0.251026	-4.524606
C	0.441308	1.492541	-6.151937
C	0.952568	0.717362	-5.116279
H	-1.771145	-1.208216	-4.509316
H	-1.262097	1.912149	-7.388275
H	0.539914	-0.861807	-3.721354
H	1.059193	2.249007	-6.620487
H	1.970846	0.861463	-4.777229
C	-3.093990	0.122238	-6.483076
H	-3.489936	1.081733	-6.829844
H	-3.719378	-0.187656	-5.642783
C	-3.255825	-0.904336	-7.615402
H	-3.149480	-2.085858	-7.049265
H	-4.283529	-0.964971	-7.975516
C	-2.280347	-1.054941	-8.619117
H	-2.569832	-1.308768	-9.629571
H	-1.298068	-0.611912	-8.518675
Se	-1.561648	-3.422088	-8.088125
O	-2.752331	-3.319308	-6.850768
C	0.076498	-3.060863	-7.131525
C	0.304293	-3.646652	-5.893525
C	1.063502	-2.267271	-7.714944
C	1.502176	-3.431634	-5.221155
C	2.262298	-2.055815	-7.056935
C	2.486284	-2.629339	-5.801601
H	-0.458914	-4.263094	-5.436615
H	0.893542	-1.800503	-8.677088
H	1.652763	-3.883256	-4.251868
H	3.028579	-1.426517	-7.490962
O	3.675925	-2.340979	-5.219150
C	3.944272	-2.880848	-3.926728
H	3.213331	-2.532284	-3.192691
H	3.950964	-3.973710	-3.946991
H	4.932607	-2.519302	-3.651423

<u>R = Me (**2b-TS**)</u>

Full E -3097.058292829905

C	-1.812830	0.400364	-6.090658
C	-1.369138	-0.319163	-4.980319
C	-0.951143	1.342099	-6.657085
C	-0.105580	-0.098681	-4.444690
C	0.316965	1.561609	-6.129621
C	0.743434	0.841468	-5.018601
H	-2.020381	-1.061077	-4.534433
H	-1.281525	1.911802	-7.518131
H	0.219648	-0.667008	-3.582806
H	0.970236	2.296286	-6.584628
H	1.730456	1.008322	-4.605672
C	-3.179847	0.160116	-6.673148
H	-3.542906	1.093994	-7.113141
H	-3.873632	-0.096357	-5.869317
C	-3.249648	-0.939164	-7.744370
H	-3.158499	-2.079332	-7.095657
H	-4.249866	-1.045167	-8.166053
C	-2.207386	-1.132404	-8.670259
H	-2.424484	-1.457421	-9.678418
H	-1.241245	-0.663793	-8.535078
Se	-1.487920	-3.447813	-7.941413
O	-2.752498	-3.287113	-6.787080
C	0.091516	-2.998076	-6.919581
C	0.265225	-3.517656	-5.639268
C	1.090266	-2.226070	-7.499582
C	1.434241	-3.249450	-4.943309
C	2.261218	-1.970096	-6.793003
C	2.451429	-2.472835	-5.506975
H	-0.513070	-4.118749	-5.187302
H	0.957704	-1.813022	-8.491813
H	1.560739	-3.647881	-3.942945
H	3.030910	-1.355886	-7.245134
C	3.699007	-2.166299	-4.728054
H	3.472367	-1.539387	-3.860780
H	4.165058	-3.079437	-4.350040
H	4.429664	-1.638535	-5.341548

R = H (**2c-TS**)

Full E -3057.749354624065

C	-1.831597	0.416148	-6.107073
C	-1.397477	-0.290476	-4.984947
C	-0.963251	1.347299	-6.680785
C	-0.135529	-0.069090	-4.446024
C	0.302975	1.568062	-6.149601
C	0.720717	0.859640	-5.027734
H	-2.054380	-1.023651	-4.532936
H	-1.286889	1.907343	-7.550669
H	0.183017	-0.628210	-3.575672
H	0.961665	2.294358	-6.610217
H	1.706555	1.026867	-4.612280
C	-3.194937	0.171501	-6.695866
H	-3.554049	1.100394	-7.149407
H	-3.894886	-0.075685	-5.894538
C	-3.255738	-0.940251	-7.754359
H	-3.159394	-2.071978	-7.091540
H	-4.253769	-1.058366	-8.177908
C	-2.208702	-1.138179	-8.673374
H	-2.419074	-1.478460	-9.677908
H	-1.245330	-0.663345	-8.539766
Se	-1.481391	-3.442647	-7.915513
O	-2.749447	-3.274964	-6.767176
C	0.096684	-2.987041	-6.889714
C	0.248610	-3.479234	-5.596878
C	1.109678	-2.246674	-7.490745
C	1.424546	-3.223656	-4.902087
C	2.286432	-2.000345	-6.790563
C	2.445509	-2.489125	-5.498463
H	-0.547360	-4.050124	-5.137009
H	0.981602	-1.856471	-8.492695
H	1.543537	-3.599211	-3.893341
H	3.072376	-1.415638	-7.251979
H	3.359622	-2.290600	-4.953332

R = Cl (**2d-TS**)

Full E -3517.318529862617

C	-1.773872	0.370471	-6.064873
C	-1.257943	-0.293604	-4.951752
C	-0.972033	1.325095	-6.695918
C	0.018193	-0.010162	-4.477443
C	0.307693	1.606480	-6.231068
C	0.807340	0.938796	-5.117139
H	-1.863357	-1.039863	-4.451438
H	-1.359145	1.854828	-7.558779
H	0.399460	-0.537067	-3.612302
H	0.913558	2.349087	-6.736037
H	1.804618	1.152483	-4.753256
C	-3.155682	0.067926	-6.580488
H	-3.606307	0.999780	-6.936001
H	-3.778632	-0.282984	-5.754968
C	-3.229526	-0.958534	-7.723615
H	-3.147006	-2.141435	-7.163782
H	-4.232009	-1.034181	-8.146537
C	-2.193942	-1.093392	-8.666784
H	-2.418874	-1.365741	-9.688859
H	-1.227265	-0.632175	-8.514205
Se	-1.452734	-3.439625	-8.074595
O	-2.743735	-3.376936	-6.943571
C	0.072428	-3.006673	-6.965648
C	0.136388	-3.480849	-5.659905
C	1.124252	-2.270665	-7.500659
C	1.251885	-3.211235	-4.878963
C	2.245401	-1.998797	-6.726452
C	2.292841	-2.470609	-5.422459
H	-0.687036	-4.046999	-5.245592
H	1.072482	-1.892160	-8.513712
H	1.305715	-3.564397	-3.858393
H	3.059802	-1.411333	-7.127157
Cl	3.683482	-2.094094	-4.428888

R = CF_3 (**2e-TS**)

Full E -3394.828612941195

C	-1.774401	0.388804	-6.065833
C	-1.208109	-0.283662	-4.983091
C	-1.015394	1.373129	-6.704263
C	0.076398	0.019963	-4.545037
C	0.272247	1.674722	-6.276158
C	0.822973	0.997963	-5.192012
H	-1.779799	-1.053964	-4.479476
H	-1.442112	1.909568	-7.544033
H	0.496947	-0.513589	-3.702813
H	0.844570	2.440403	-6.785753
H	1.825744	1.229404	-4.855993
C	-3.162599	0.060556	-6.546627
H	-3.647072	0.985918	-6.873154
H	-3.753235	-0.321224	-5.711148
C	-3.237073	-0.946884	-7.706172
H	-3.130124	-2.136346	-7.170302
H	-4.243011	-1.032111	-8.118878
C	-2.210664	-1.047699	-8.663487
H	-2.442734	-1.315025	-9.685365
H	-1.251381	-0.569132	-8.518222
Se	-1.420100	-3.387405	-8.116909
O	-2.705174	-3.374232	-6.979349
C	0.104534	-2.978723	-6.996773
C	0.139840	-3.438422	-5.684909
C	1.186366	-2.291827	-7.537764
C	1.269257	-3.217673	-4.911015
C	2.316168	-2.070904	-6.762690
C	2.359224	-2.542707	-5.454001
H	-0.711443	-3.960884	-5.270368
H	1.151485	-1.923646	-8.555175
H	1.300091	-3.573126	-3.890100
H	3.155791	-1.529375	-7.176705
C	3.560874	-2.259607	-4.603774
F	3.463998	-1.064971	-3.961963
F	3.746759	-3.188294	-3.641540
F	4.703542	-2.206681	-5.322656

R = CN (**2f-TS**)

Full E -3149.990546054587

C	-1.772427	0.357646	-6.042843
C	-1.245133	-0.291852	-4.926970
C	-0.980254	1.310059	-6.689905
C	0.033501	0.002255	-4.465739
C	0.302381	1.600274	-6.239577
C	0.814204	0.946034	-5.122872
H	-1.843691	-1.035575	-4.414535
H	-1.377054	1.831154	-7.553581
H	0.422629	-0.512059	-3.596557
H	0.900996	2.340068	-6.757104
H	1.813277	1.168200	-4.769128
C	-3.157110	0.042833	-6.542862
H	-3.627869	0.972799	-6.876549
H	-3.762156	-0.331628	-5.714598
C	-3.231714	-0.965070	-7.702033
H	-3.123494	-2.153902	-7.167982
H	-4.238579	-1.051429	-8.112339
C	-2.208552	-1.064977	-8.662005
H	-2.442948	-1.335032	-9.682616
H	-1.247637	-0.589172	-8.519030
Se	-1.415466	-3.407938	-8.115514
O	-2.698180	-3.394031	-6.975987
C	0.104513	-2.984074	-6.996843
C	0.129854	-3.419461	-5.675352
C	1.186250	-2.299724	-7.544430
C	1.245183	-3.173674	-4.893649
C	2.303667	-2.046618	-6.766233
C	2.335906	-2.487217	-5.438879
H	-0.722697	-3.939106	-5.260057
H	1.155474	-1.952876	-8.569380
H	1.275536	-3.502518	-3.863843
H	3.143649	-1.501404	-7.174394
C	3.474377	-2.204995	-4.627837
N	4.391881	-1.969016	-3.970397

IRC point 226 (tunneling product)

R = OMe (**3** and **4a**)

Full E -3172.288883774562

C	-1.681922	0.329394	-6.000437
C	-1.153481	-0.445062	-4.967728
C	-0.867120	1.300103	-6.586547
C	0.149229	-0.250709	-4.524010
C	0.440385	1.492375	-6.152222
C	0.952253	0.717408	-5.116502
H	-1.770569	-1.207084	-4.507099
H	-1.264031	1.912971	-7.387566
H	0.539521	-0.860703	-3.719967
H	1.058090	2.248986	-6.620675
H	1.970596	0.861951	-4.777896
C	-3.097182	0.117582	-6.469158
H	-3.526726	1.090162	-6.738175
H	-3.701331	-0.254111	-5.637760
C	-3.268646	-0.775349	-7.677860
H	-3.096222	-2.378615	-6.888462
H	-4.294619	-1.061966	-7.895947
C	-2.332676	-0.980081	-8.617969
H	-2.589914	-1.389161	-9.583287
H	-1.322782	-0.605784	-8.513679
Se	-1.544703	-3.444864	-8.095164
O	-2.776578	-3.316817	-6.815853
C	0.070895	-3.066544	-7.127871
C	0.307352	-3.649625	-5.889485
C	1.056661	-2.266865	-7.711231
C	1.502757	-3.429536	-5.218327
C	2.260071	-2.058283	-7.057570
C	2.486600	-2.628687	-5.801915
H	-0.448183	-4.275980	-5.433671
H	0.883365	-1.797715	-8.671528
H	1.655438	-3.881008	-4.249330
H	3.025424	-1.430054	-7.494947
O	3.676658	-2.340755	-5.219241
C	3.944920	-2.881015	-3.927016
H	3.214621	-2.533176	-3.192093
H	3.951792	-3.973892	-3.947480
H	4.933331	-2.519626	-3.651707

R = Me (**3** and **4b**)

Full E -3097.077658935644

C	-1.811613	0.399358	-6.089841
C	-1.367988	-0.319324	-4.979833
C	-0.952455	1.342346	-6.657451
C	-0.104648	-0.098517	-4.444173
C	0.315874	1.561557	-6.130130
C	0.743031	0.841872	-5.018926
H	-2.019683	-1.059917	-4.531984
H	-1.283637	1.912452	-7.517767
H	0.220910	-0.666910	-3.582500
H	0.968946	2.296384	-6.585095
H	1.729817	1.010722	-4.606543
C	-3.184347	0.156960	-6.659269
H	-3.585056	1.109638	-7.025947
H	-3.857011	-0.160086	-5.858551
C	-3.260958	-0.814586	-7.816356
H	-3.110079	-2.359988	-6.911985
H	-4.264930	-1.136794	-8.081804
C	-2.260757	-1.058801	-8.677396
H	-2.445332	-1.534248	-9.628819
H	-1.266499	-0.657472	-8.531265
Se	-1.470106	-3.470489	-7.945832
O	-2.778289	-3.283352	-6.753660
C	0.085655	-3.003936	-6.915818
C	0.267661	-3.519595	-5.634317
C	1.083486	-2.226495	-7.496559
C	1.434604	-3.247238	-4.940404
C	2.259049	-1.972914	-6.793711
C	2.451991	-2.472318	-5.507395
H	-0.503381	-4.129648	-5.182209
H	0.947391	-1.811139	-8.487249
H	1.563117	-3.644958	-3.940003
H	3.027988	-1.360172	-7.249271
C	3.699629	-2.166594	-4.728458
H	3.473918	-1.541099	-3.859972
H	4.165108	-3.080463	-4.351195
H	4.430911	-1.638825	-5.341236

R = H (**3** and **4c**)

Full E -3057.768714098193

C	-1.830664	0.415511	-6.106081
C	-1.396524	-0.290438	-4.984389
C	-0.964423	1.347513	-6.681093
C	-0.134780	-0.068882	-4.445447
C	0.302106	1.567815	-6.150045
C	0.720550	0.859602	-5.028089
H	-2.054012	-1.022103	-4.530326
H	-1.288655	1.907926	-7.550364
H	0.183805	-0.627706	-3.575012
H	0.960703	2.294043	-6.610814
H	1.706643	1.027076	-4.613391
C	-3.199707	0.168570	-6.681868
H	-3.597071	1.117048	-7.062698
H	-3.878376	-0.139043	-5.882621
C	-3.267478	-0.816615	-7.827927
H	-3.110072	-2.351233	-6.903428
H	-4.268430	-1.148144	-8.093256
C	-2.262462	-1.065167	-8.681841
H	-2.440191	-1.554153	-9.627736
H	-1.270760	-0.657110	-8.536590
Se	-1.463444	-3.465061	-7.919617
O	-2.774958	-3.271447	-6.733792
C	0.090827	-2.993570	-6.885926
C	0.251026	-3.480924	-5.591495
C	1.103085	-2.247730	-7.488071
C	1.425247	-3.221225	-4.899179
C	2.283946	-2.003245	-6.791455
C	2.446413	-2.488565	-5.499097
H	-0.537493	-4.061123	-5.130721
H	0.971366	-1.854874	-8.488402
H	1.546520	-3.596869	-3.890699
H	3.068839	-1.419777	-7.256361
H	3.361850	-2.290148	-4.956194

R = Cl (**3** and **4d**)

Full E -3517.338483785010

C	-1.772875	0.370138	-6.063996
C	-1.257284	-0.293370	-4.951281
C	-0.973340	1.325562	-6.696080
C	0.018618	-0.009711	-4.476938
C	0.306744	1.606349	-6.231362
C	0.807055	0.938839	-5.117468
H	-1.862914	-1.038679	-4.449739
H	-1.360762	1.855560	-7.558408
H	0.399798	-0.536124	-3.611568
H	0.912487	2.348897	-6.736493
H	1.804559	1.152637	-4.754315
C	-3.158226	0.062870	-6.568275
H	-3.646223	1.004629	-6.848269
H	-3.757883	-0.348951	-5.752618
C	-3.239928	-0.830455	-7.785736
H	-3.106734	-2.445785	-7.004420
H	-4.246014	-1.137176	-8.062080
C	-2.248254	-1.021097	-8.669071
H	-2.442506	-1.444224	-9.643286
H	-1.252773	-0.628390	-8.510991
Se	-1.434446	-3.461248	-8.079904
O	-2.770676	-3.376311	-6.911651
C	0.065907	-3.012972	-6.963138
C	0.138012	-3.482802	-5.655114
C	1.117325	-2.271147	-7.498450
C	1.251864	-3.208975	-4.876666
C	2.242910	-2.001718	-6.727295
C	2.293323	-2.470161	-5.422887
H	-0.677132	-4.058861	-5.238545
H	1.062351	-1.889201	-8.509925
H	1.307883	-3.562757	-3.856398
H	3.056216	-1.414795	-7.131214
Cl	3.683840	-2.093938	-4.428884

R = CF_3 (**3** and **4e**)

Full E -3394.848828546481

C	-1.773541	0.388607	-6.064731
C	-1.207396	-0.283483	-4.982605
C	-1.016790	1.373585	-6.704245
C	0.076958	0.020267	-4.544649
C	0.271238	1.674591	-6.276435
C	0.822713	0.997964	-5.192401
H	-1.778916	-1.053224	-4.477949
H	-1.443796	1.910043	-7.543614
H	0.497969	-0.513484	-3.702850
H	0.843414	2.440143	-6.786323
H	1.825761	1.229470	-4.857287
C	-3.164471	0.055176	-6.535152
H	-3.683780	0.987925	-6.787347
H	-3.732211	-0.385437	-5.711696
C	-3.250023	-0.818992	-7.765675
H	-3.082869	-2.448159	-7.015829
H	-4.253786	-1.141390	-8.032428
C	-2.267050	-0.976985	-8.664523
H	-2.465539	-1.392236	-9.641444
H	-1.277100	-0.567754	-8.514139
Se	-1.401366	-3.407896	-8.122631
O	-2.731347	-3.374916	-6.947058
C	0.097796	-2.986961	-6.994790
C	0.140968	-3.440029	-5.679510
C	1.179729	-2.293231	-7.535838
C	1.269140	-3.215284	-4.908757
C	2.313102	-2.073859	-6.763588
C	2.360069	-2.542533	-5.454235
H	-0.702314	-3.971781	-5.260977
H	1.141866	-1.921329	-8.551701
H	1.301720	-3.570918	-3.887944
H	3.151128	-1.532111	-7.180823
C	3.560622	-2.259605	-4.604583
F	3.464295	-1.064716	-3.961733
F	3.746718	-3.188146	-3.641524
F	4.703907	-2.206446	-5.322653

R = CN (**3** and **4f**)

Full E -3150.011021005601

C	-1.771478	0.357462	-6.041966
C	-1.244465	-0.291918	-4.926790
C	-0.981577	1.310728	-6.689882
C	0.033888	0.002477	-4.465305
C	0.301439	1.600177	-6.239722
C	0.813881	0.946004	-5.123087
H	-1.842808	-1.036525	-4.415241
H	-1.378596	1.832096	-7.553007
H	0.423255	-0.512123	-3.596459
H	0.899982	2.339876	-6.757396
H	1.813209	1.168150	-4.770094
C	-3.159190	0.037532	-6.531407
H	-3.665308	0.975610	-6.791019
H	-3.741617	-0.395326	-5.714344
C	-3.244571	-0.837258	-7.761560
H	-3.076571	-2.469233	-7.012699
H	-4.248725	-1.160146	-8.026381
C	-2.264261	-0.995135	-8.662684
H	-2.465137	-1.412537	-9.638258
H	-1.273319	-0.587430	-8.514888
Se	-1.396687	-3.428049	-8.121106
O	-2.724125	-3.395461	-6.943532
C	0.097327	-2.992450	-6.995346
C	0.130691	-3.420714	-5.670025
C	1.179774	-2.301321	-7.542785
C	1.244903	-3.171214	-4.891714
C	2.300374	-2.049565	-6.767256
C	2.336941	-2.486732	-5.439004
H	-0.713631	-3.949496	-5.250056
H	1.146257	-1.950492	-8.566201
H	1.276865	-3.500641	-3.862113
H	3.138551	-1.503880	-7.178752
C	3.474740	-2.204653	-4.628597
N	4.391979	-1.969069	-3.970109

References:

1. Neese, F. The ORCA program system. *Wiley Interdiscip. Rev. Comput. Mol. Sci.* **2**, 73–78 (2012).
2. Neese, F. Software Update: The ORCA Program System—Version 6.0. *Wiley Interdiscip. Rev. Comput. Mol. Sci.* **15**, e70019 (2025).
3. Lee, H. I. *et al.* Structure of the Modified Heme in Allylbenzene-Inactivated Chloroperoxidase Determined by Q-Band CW and Pulsed ENDOR. *J. Am. Chem. Soc.* **119**, 4059–4069 (1997).
4. Xiao, J., Li, Z. & Montgomery, J. Nickel-Catalyzed Decarboxylative Coupling of Redox-Active Esters with Aliphatic Aldehydes. *J. Am. Chem. Soc.* **143**, 21234–21240 (2021).
5. Singh, D. *et al.* An Efficient One-Pot Synthesis of Symmetrical Diselenides or Ditellurides from Halides with CuO Nanopowder/Se0 or Te0/Base. *Org. Lett.* **12**, 3288–3291 (2010).
6. Tricoire, M. *et al.* Electron Shuttle in N-Heteroaromatic Ni Catalysts for Alkene Isomerization. *JACS Au* **2**, 1881–1888 (2022).
7. 8.4. Compound Examples - ORCA 6.1 Manual. https://www.faccts.de/docs/orca/6.1/manual/contents/workflowsautomatization/compound_examples.html?q=compound&n=2#compound-examples.
